# Dynamics of genome change among *Legionella* species

**DOI:** 10.1038/srep33442

**Published:** 2016-09-16

**Authors:** Sandeep J. Joseph, Daniel Cox, Bernard Wolff, Shatavia S. Morrison, Natalia A. Kozak-Muiznieks, Michael Frace, Xavier Didelot, Santiago Castillo-Ramirez, Jonas Winchell, Timothy D. Read, Deborah Dean

**Affiliations:** 1 Department of Medicine, Division of Infectious Diseases, Emory University School of Medicine, Atlanta, Georgia, USA; 2 Department of Microbiology and Immunology, Emory University School of Medicine, Atlanta, Georgia, USA; 3 Respiratory Diseases Branch, Centers for Disease Control and Prevention, Atlanta, Georgia, USA; 4 Department of Infectious Disease Epidemiology, Imperial College, Norfolk Place, London, United Kingdom; 5 Programa de Genomica Evolutiva, Centro de Ciencias Genómicas, Universidad Nacional Autónoma de México, Cuernavaca, Morelos, México; 6 Department of Human Genetics, Emory University School of Medicine, Atlanta, Georgia, USA; 7 Department of Medicine and University of California, San Francisco, San Francisco, California, USA; 8 Department of Biomedical Engineering, University of California at San Francisco and Berkeley, San Francisco and Berkeley, California, USA; 9 ^9^Center for Immunobiology and Vaccine Development, UCSF Benioff Children’s Hospital Oakland Research Institute, Oakland, California, USA

## Abstract

*Legionella* species inhabit freshwater and soil ecosystems where they parasitize protozoa. *L. pneumonphila* (LP) serogroup-1 (Lp1) is the major cause of Legionnaires’ Disease (LD), a life-threatening pulmonary infection that can spread systemically. The increased global frequency of LD caused by Lp and non-Lp species underscores the need to expand our knowledge of evolutionary forces underlying disease pathogenesis. Whole genome analyses of 43 strains, including all known Lp serogroups 1–17 and 17 emergent LD-causing *Legionella* species (of which 33 were sequenced in this study) in addition to 10 publicly available genomes, resolved the strains into four phylogenetic clades along host virulence demarcations. Clade-specific genes were distinct for genetic exchange and signal-transduction, indicating adaptation to specific cellular and/or environmental niches. CRISPR spacer comparisons hinted at larger pools of accessory DNA sequences in Lp than predicted by the pan-genome analyses. While recombination within Lp was frequent and has been reported previously, population structure analysis identified surprisingly few DNA admixture events between species. In summary, diverse *Legionella* LD–causing species share a conserved core-genome, are genetically isolated from each other, and selectively acquire genes with potential for enhanced virulence.

Legionellae are gram-negative, facultative intracellular bacteria that are responsible for sporadic and epidemic outbreaks of atypical community-acquired pneumonia (CAP)^1^. The clinical presentation is “atypical” in that, in addition to pneumonia, there is often extrapulmonary organ infection and a noted resistance to Beta Lactam antibiotics. The atypical CAP presentation can also occur during infection with other bacterial pathogens such as *Chlamydia pneumoniae, Chlamydia psittaci, Mycoplasma pneumoniae, Francisella tularensis* and *Coxiella burnetii*. Accurate attribution of the correct etiologic agent can, therefore, be challenging.

The best-known legionellosis is a severe atypical CAP referred to as Legionnaire’s Disease (LD)^1^,^2^. The first report of LD was among attendees of the bicentennial celebration of the American Legion Auxiliary^2^ in 1976, where transmission occurred via the hotel air conditioning system. Another, lesser known, legionellosis is Pontiac Fever^3^ characterized by mild flu-like symptoms without pneumonia. Because legionellae inhabit freshwater ecosystems, they are often found in man-made water systems such as showers, spas, hospital heating systems, cooling towers, ventilation and air conditioning units, and decorative water fountains, which are the usual sources for aerosolization of the organism and subsequent clinical outbreaks. In addition, legionellae are able to form biofilms, and survive and replicate in the soil and within amoeba, which can shield them from environmental disinfectants, creating an even larger public health threat^4^,^5^.

*L. pneumophila* serogroup 1 (Lp1) is the leading cause of LD, accounting for up to 92% of clinically recognized legionellosis infections in the US and Europe^1^. Although Lp1 predominates as the cause of LD, it is not found at disproportionately higher rates in the environment than the 16 other Lp serogroups. *L. bozemanii* serogroups 1 and 2, *L. dumoffii*, and *L. micdadei,* account for most of the remaining human infections^6^,^7^. Other species of *Legionellae* rarely cause disease except for *L. longbeachae* where high rates of infection and disease have been reported in Australia and New Zealand^8^. According to the Centers for Disease Control and Prevention (CDC), the number of cases reported rose 217% while the incidence rate of legionellosis in the US increased almost 200% between 2000 and 2009^9^. In 2015, there were 15 outbreaks in the US and Europe with a 10% case fatality rate in addition to the growing number of cases caused by non- Lp species. There is also a recent report of probable person-to-person transmission^10^. These collective findings drive home the critical need to better characterize legionellae to improve our understanding of their biology and epidemiology to advance the design of strategic interventions.

While sequence-based typing (SBT) has been used for outbreak investigations because of its historically lower cost, whole genome sequencing (WGS) of eight Lp1 and Lp12 strains, in addition to a large number of Lp1 strains linked to geographic outbreaks^11^,^12^,^13^,^14^,^15^,^16^,^17^,^18^, has revealed how genetic exchange plays a role in shaping the virulence potential of the species. Genes encoding features such as drug resistance islands, secretion systems and a large repertoire of secreted effector proteins^19^ are part of the mobile accessory portion of the pan-genome.

These prior WGS studies have analyzed outbreaks within a specific area but none have evaluated the population genomics of all 17 Lp serogroups and the 18 other *Legionella* species associated with human disease, albeit rarely, to understand their evolutionary strategies. Here, we compared the dynamics of genome change both within Lp and across the *Legionella* genus. Our genomics analyses encompassed 43 species and serogroups of *Legionella*, including 33 strains (Lp serogroups 2 through 17, and 17 other *Legionella* species) sequenced in the present study along with nine Lp1 strains and *L. longbeachae* that were publically available.

## Results

### *Legionella* species fall into at least four clades

To improve our understanding of evolutionary forces acting on *Legionella* species, we performed comparative genomic analyses to elucidate population structure and estimate the effects of homologous recombination. The analysis set consisted of 10 published and 33 *Legionella* genomes sequenced in this study, including *Legionella* species occasionally reported as etiologic agents of human disease and Lp subtypes 2 through 17 (Table 1). Genomes were assembled using a hybrid assembly of Roche 454 and Illumina in this study where each genome project contained at least 3 million sequencing reads (combined Illumina and 454 sequencing technologies) (Supplementary Table 1).

**Figure 1:**
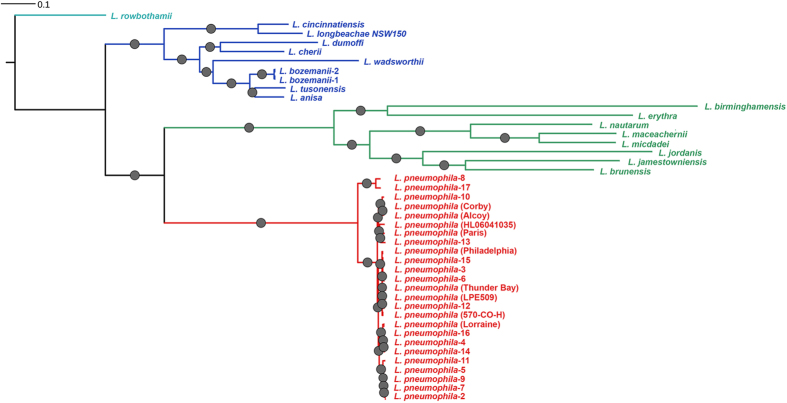
Whole genome phylogeny of all *Legionella* species. Whole genome nucleotide phylogeny of *Legionella* species. The tree was constructed using maximum likelihood approach with the RAxML method and the concatenated progressiveMAUVE alignment of 759,392 nucleotides of the 1140 protein-coding core genes. The protein phylogenetic tree as well as the tree inferred by ClonalFrame also showed the same topology. Clade 1 is shown in red, Clade 2 in green, Clade 3 in blue and Clade 4 (with a single isolate; *L. rowbothamii*) in cyan. The circles on the branches indicate branches with >90 bootstrap estimates out of a total of 100 generated to reconstruct the internal branches of the phylogeny.

**Figure 2:**
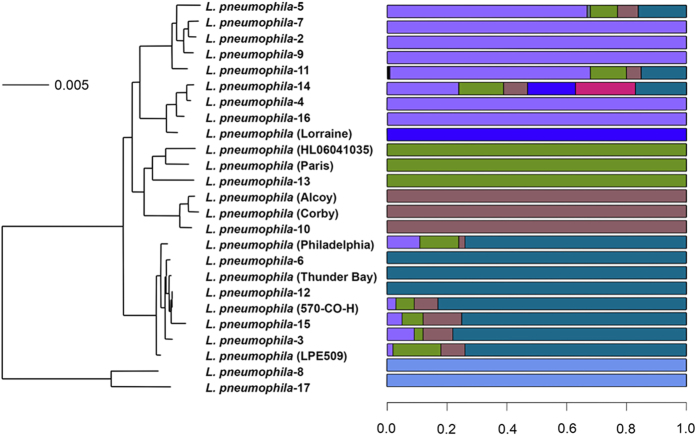
*L. pneumophila* (Lp) whole genome phylogeny and BAPS predicted population groups. All strains without a number following the species name are Lp serogroup 1 (Lp1) while those with numbers represent Lp serogroups. The Lp strains were a monophyletic group based on the whole genome phylogeny.

Contigs from assembled shotgun data were annotated for genes and RNA features using the PROKKA pipeline^20^. The average number of protein-coding genes across the genus was around 3,200 (Supplementary Table 1). Predicted genes were classified as either core or accessory within the *Legionella* genus pan-genome using OrthoMCL^21^. Genus phylogeny of whole genome data was inferred using maximum likelihood (ML) phylogeny of the concatenated progressiveMAUVE alignment^22^ of 759,392 nucleotides of the 1140 protein-coding genes (Fig. 1), the ML phylogeny of the concatenated alignment of 299,244 amino acid residues of the 1140 translated core genes (Supplementary Figure 1), and a ClonalFrame nucleotide-based phylogenetic analysis^23^. The latter has the advantage of removing most recombinant regions of the DNA alignment from consideration. All three approaches yielded similar phylogenetic tree topologies from which four clades were defined (Fig. 1): Clade 1 (red) contained only Lp strains, Clade 2 (green) contained eight *Legionella* species: *L. birminghamensis, L. erythra, L. nautarum, L. maceachernii, L. micdadei, L. jordanis, L. jamestowniensis* and *L. brunensis*; Clade 3 (blue) nine *Legionella* species: *L. cincinnatiensis, L. longbeachae NSW150, L. dumoffii, L. cherii, L. wadsworthii, L. bozemanii 1, L. bozemanii 2, L. tucsonensis* and *L. anisa,* and Clade 4 only *L. rowbothamii*. Lp strains were a monophyletic group based on the whole genome phylogeny (Fig. 2).

### Clade-specific accessory genes form a significant portion of the *Legionella* pan-genome

Based on the annotations described above, we enumerated the *Legionella* pan-genome and how accessory genes defined species relationships. We identified 12,977 ortholog gene families in all 43 genomes based on OrthoMCL clustering with a BLASTP identity cutoff of 10^−5^. Supplementary Figure 2A shows the barplot listing the number of gene clusters found in each genome. There were 1140 core gene families in all 43 genomes, representing the core genes in *Legionella* species, which were used in downstream phylogenetic, recombination and positive selection analyses.

The pan-genome rarefaction curve did not level out, indicating the pan-genome is “open” (i.e., not fully sampled) for the 19 *Legionella*, species (Supplementary Figure 2B). The Heaps law parameter **α**, a measure of the rate of novel information discovery^24^, was estimated to be 0.63. Values of **α** greater than 1 suggest a closed pan-genome. Using the matrix of gene family distribution across *Legionella*, we fitted a binomial mixture model implemented by the binomixEstimate function in the micropan R package^25^. This function estimated a series of mixture models with increasing complexity and used the Bayesian Information Criterion (BIC), estimating the optimum *Legionella* pan-genome size to be 30,275 genes (Supplementary Figure 2C).

To investigate patterns of shared gene content, we ran a principal component analysis (PCA) on the gene family distribution matrix using the *panpca* function (micropan R package). Around 34% of the total variation among the genomes was seen along the 2 principal components (Supplementary Figure 3). Genomes in the same clade formed non-overlapping groups. Species that formed clades (based on relatedness of core genome nucleotide sequences) were also more similar to each other in families of accessory genes.

We assigned functions to representative members of each family through BLASTX alignment to the BLAST2GO database^26^. Following the common trend in bacterial species pan-genomes, most core gene families mapped to a Gene Ontology (GO) category. Most of the accessory genes, however, could not be assigned with the exception of genes responsible for horizontal gene transfer (HGT) (e.g., transposition) and signal transduction (Supplementary Table 2a,b).

There were 1160 Clade 1 specific genes (Supplementary Table 3a), 3078 Clade 2 specific genes (Supplementary Table 3b), 2452 Clade 3 specific genes (Supplementary Table 3c), and 507 Clade 4 specific genes (Supplementary Table 3d). The tetracycline destructase gene was found in two Clade 2 species (*L. jordanis* and *L. nautarum*) and in Clade 3 species *L. longbeachae* but not in the other two clades.

*Legionella* systems for secreting effector proteins into the host cell environment are a key factor in intracellular survival and virulence^8^,^27^. Confirming and extending a previous finding^8^, we identified that the Dot/Icm type IVB secretion system (T4BSS) and Lsp type II secretion system (T2SS) belonged to the core genome of all *Legionella* species. However, we found that *Legionella* species are variable in their type IVA secretion systems (T4ASS) as they were present in different species except for the P-type, which was present in all species and codes for conjugative pili required for mating^8^,^28^ (Table 1). The F-type T4ASS also codes for pili but was missing from genomes Lp2, Lp3, Lp6 and *L. cherii* while the Lvh (*Legionella virB* homologue) T4ASS, which is involved in spread of infection from environmental niches^8^,^28^, was identified in only 10 *Legionella* genomes: 6 genomes from Clade 1; and 2 from Clade 3 (*L. bozemanii* serogroup 2 and *L. dumoffii*) (Table 1). Interestingly, Lp3 and Lp6 had Lvh T4ASS but not the F-type T4ASS in their genomes. Recently identified Genomic Islands (GI) T4SS (GI-T4SS), GI-T4SS-1 and GI-T4SS-2, which were found in Lp 130b and recognized to be involved in host adaptation^29^, were identified across Lp serogroups; 2 clusters each were present in Lp6, Lp11, Lp12, and Lp16 while a single cluster was present in Lp3, Lp7 Lp10, and Lp17 (Table 1). A single cluster of GI-T4SS (GI-T4SS-1) was also identified in non-Lp species *L. cherii* and *L. dumoffii,* both in Clade 3.

Because these data were from draft genome assemblies, we could not identify plasmid contigs with certainty. We looked for nine previously identified *Legionella* species plasmids available in GenBank (accessed 12/29/2016) in our genomes. *L. dumoffii* (Clade 3) and *L. jamestowniensis (*Clade 2), carry the Lp Lorraine plasmid pLELO (GI Accession: NC_018141) with 100% identity. Similarly, Lp13 and Lp5 had the Lp Lens plasmid pLPL. We also found partial matches to several other *Legionella* plasmids in 25 genomes (BLASTN identity >95%), suggesting that other strains contain plasmid genes.

### Positive selection analysis of core genes

We tested for clade-specific signatures of positive selection using PAML^30^, and identified 10 core genes (FDR p-value < 0.05) in Clade 1 compared to the other clades. These genes had no known relationship to *Legionella* virulence or interactions with each other based on searching the STRING database^31^. Clade 2 had the highest number of genes under positive selection (362 genes) followed by Clade 3 (170 genes) (Supplementary Table 4a,c,e). Eight genes total were under positive selection in all 3 clades sans Clade 4. Ninety-seven core genes showed evidence of positive selection in both Clades 2 and 3. T4SS protein IcmL was under positive selection in both Clades 2 and 3, while IcmC and IcmG were under positive selection only in Clade 3. Similarly, the T2SS protein LspJ was under selection only in Clade 3, while LspD was under selection only in Clade 2. There were 272 and 78 clade-specific core genes under selection in Clades 2 and 3, respectively.

Results of GO enrichment analysis of genes identified as under positive selection are shown in Supplementary Table 4b,d,f). Additionally, we performed GO enrichment analysis on genes estimated to be gained or lost on the terminal branches of each clade^32^ (Supplementary Table 5a–d). As expected most of the enriched GO terms mapped to functions associated with horizontally transferred elements such as CRISPRs (Clustered Regularly Interspaced Short Palindromic Repeats).

### CRISPR spacer comparisons hint at larger pool of DNA entering *Legionella* than predicted by comparative genomics

CRISPRs offer an alternative view on foreign DNA entering *Legionella* than the pan-genome estimated by comparative genomics. CRISPRs are bacterial adaptive immune systems for protection against DNA from non-hosts such as infecting phage^33^. They consist of *cas* genes and adjacent tandem arrays of short spacer DNA originating from the target organism. Cas-RNA spacer complexes destroy non-host DNA via specific binding. CRISPR spacer arrays are effectively a sampling of recently introduced foreign DNA. CRISPRs were previously identified in Lp Alcoy and Paris genomes^15^. Here, we identified another 22 ‘typical’ CRISPR sequences with at least two spacers in 13 *Legionella* genomes using CRISPRfinder^34^ (Supplementary Table 6). Some genomes had multiple CRISPR sequences: three were identified in Lp13 and Lp14, and two in Lp5, Lp7, and Lp11 (Supplementary Table 6; Supplementary Data 1). Lp5 had a CRISPR array with the highest number of spacers at 70. We also discovered questionable/putative CRISPRs that had one or few spacers (Supplementary Table 6; Supplementary Data 1). A CRISPR database search revealed the presence of four types of putative CRISPR-associated proteins: Cas large protein; Cas3; Cas1; and RAMP Csd1 family protein. Spacer sequences were specific for each strain. Of 555 spacer sequences in the *Legionella* genomes, 38 overlapped based on DNA sequence identity using the dnaclust tool^35^. Surprisingly, only 42/555 (7.6%) of the spacers were found to match a *Legionella* protein in the pan-genome database constructed using BLASTX with percent identity over the alignment length of >90%. Recently, Rao *et al*.^36^ identified 440 spacers in the *L. pneumophila* CRISPR-cas system, experimentally identifying the first known target of this system: a 30 kilobase episome (LME-1) of unknown function where interbacterial transfer is guarded against by CRISPR-Cas. Out of the 440 spacers identified, 277 spacers, of which 185 were unique, had exact matches with the 555 spacers identified in the present study. LME-1 was identified only in the Lp7 genome. These results suggest that a larger pool of foreign DNA infects *Legionella* than is reflected in the pan-genome, which counts only genes stably integrated into the genome.

### Recombination between *Legionella* clades and genus population structure

**Figure 3:**
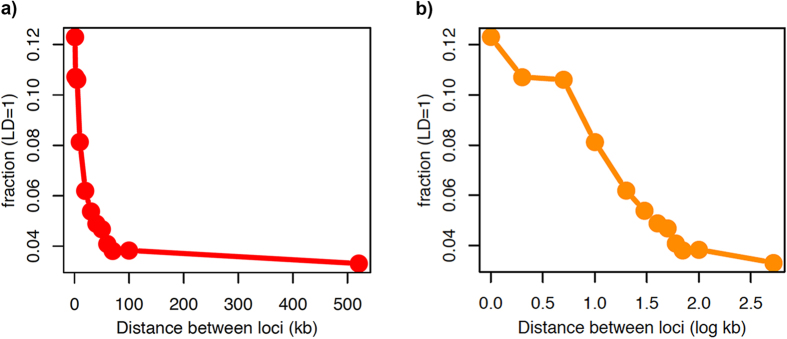
Illustration of distance-dependent decay in *Legionella* species core genome. (**a**) For pairs of loci separated by increasing genetic distance on a linear scale of the *Legionella* genome, the proportion of pairs in full linkage (number of pairs with DA’ = 1÷ total number of pairs in that distance bin) is plotted on the y-axis. (**b**) The same plot as in Figure **a** but the distance between loci is shown on a log scale (log kb).

Bacterial species are diverse in population structure, some being quite clonal (e.g., *Mycobacterium tuberculosis)*^37^ while others show high levels of recombination (e.g., *Neisseria gonorrhoeae* and *Legionella* spp.)^14^,^16^,^23^. Recombination in a bacterial species is predicted to result in linkage disequilibrium, decaying as the distance between loci in the genome increases^38^. Similar to the acquisition of accessory genes by HGT, homologous recombination is potentially a way for bacterial species to share fitness gains under environmental selection pressure^39^. We measured linkage disequilibrium between alleles of core genes in the genus and found strong linkage up to ~20 kb, which tailed off to background levels after 100 kb (Fig. 3a,b). Levels of linkage disequilibrium decay fell somewhere between that seen for *Chlamydia trachomatis* and *E. coli*, suggesting that significant levels of homologous recombination occur between core genes. Using three classic substitution analysis methods (PHI, NSS and Maximum-Chi^2^) implemented for detecting intragenic homologous recombination, 119 core genes showed significant evidence for recombination (FDR corrected p-value <0.1) in at least one method (Supplementary Table 7a–c).

**Figure 4:**
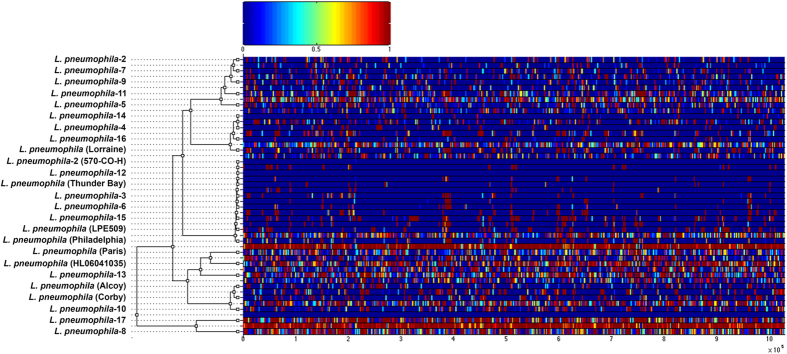
Results of the ClonalFrame analysis on an alignment of the 25 *L. pneumophila* genomes. The inferred clonal genealogy of *L. pneumophila* is shown on the left. Each branch of the tree corresponds to a row of the heat map, which is horizontally aligned according to the core MAUVE whole genome alignment. Each row of the heat map shows the posterior probability of recombination estimated by ClonalFrame on the corresponding branch (y axis) and along the positions of the alignment (x axis). The site-wise posterior probabilities of recombination are color-coded according to the legend shown at the top.

The impact of fixed recombination events was quantified by applying the ClonalFrame algorithm on the Lp genome alignment (intraspecific alignment; 1,028,806 bp). We detected recombination events on all branches of the clonal genealogy (Fig. 4). ClonalFrame estimated two values, *ρ*/*θ* and *r*/*m*, where the former measures the frequency of occurrence of recombination relative to mutation and the latter how important the effect of recombination is in genetic diversification relative to mutation. The ρ/θ was 0.124 (95% credibility interval of 0.177–0.133), implying that fixed recombination events have transferred relatively large portions of the genome between strains and/or serogroups. The *r/m* was 2.174 (95% credibility interval of 2.092–2.241), demonstrating that recombination events have played a larger role in shaping Lp genomes. The importance of recombination in Lp is supported by a study where 69 Lp1 genome sequenced samples isolated over 11 years in Spain showed that 98% of SNP diversity between strains was explained by only 16 recombination events^18^. The r/m value reported between these strains was 47.93.

We also estimated recombination based on a MAUVE alignment of all 43 *Legionella* genomes (759,392 bp): ρ/θ was estimated at 0.562 (95% credibility interval of 0.31–0.911) and the *r/m* value was 6.941 (95% credibility interval of 3.693–11.255) (Supplementary Figure 2). These values are not directly comparable to the values based on the intraspecific alignment, which is larger and covers more diverse regions of the conserved core genome, but they suggest recombination may be similarly important in other *Legionella* species.

**Figure 5:**
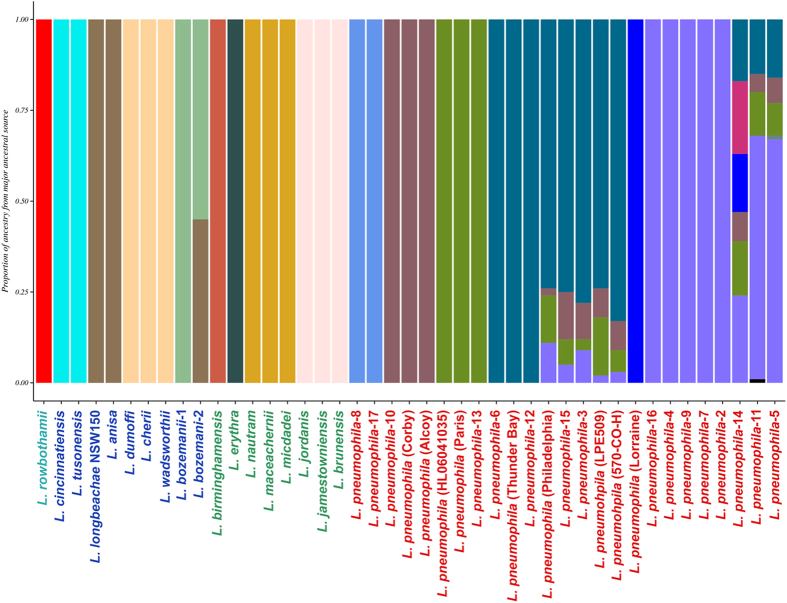
Admixture analysis of *Legionella* species. Mixture and admixture analyses were conducted using the software BAPS. This hierarchical Bayesian model-clustering based on core SNP alignment estimated 23 clusters/ancestral populations at the finest hierarchical level. Each color represents one of the genetically differentiated ancestral *Legionella* groups, and each vertical colored bar corresponds to one strain/species. When a vertical bar shows two or more colors, each color corresponds to one of the groups, showing evidence for admixture; the proportion of every color in the bar reflects the proportion of the isolate coming from the group represented by that particular color.

**Figure 6:**
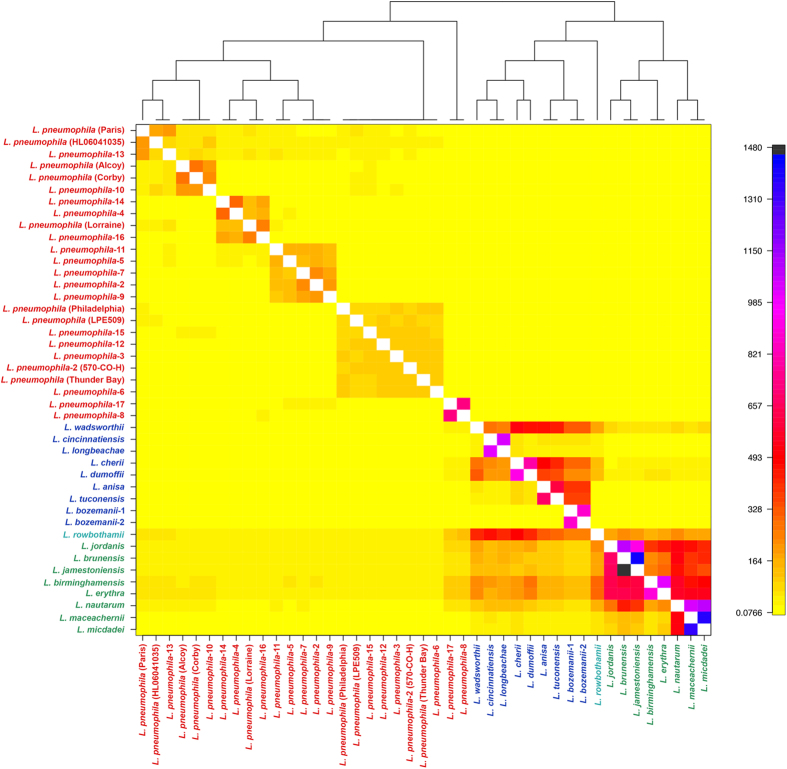
ChromoPainter co-ancestry matrix for *Legionella* species with population structure assignment based on fineSTRUCTURE analysis. The color of each cell of the matrix indicates the expected number of genetic material copied from a donor *Legionella* genome (x-axis) to a recipient genome (y-axis). The combined ChromoPainter and fineSTRUCTURE analysis assigned individual *Legionella* genomes to 21 populations.

Since ClonalFrame revealed that recombination has been a major factor in *Legionella* species evolution, we investigated how genetic variation is apportioned across species using BAPS^40^ and ChromoPainter + fineSTRUCTURE^41^. Both methods establish genetically differentiated groups and infer possible admixture occurring among those groups. As input, we used the 759,392 nucleotide MAUVE alignment based on conserved blocks of all 43 genomes. BAPS estimated the presence of 15 populations (Fig. 5) at the finest hierarchical level (within the Lp species), while ChromoPainter + fineSTRUCTURE assigned individual *Legionella* genomes to 21 populations (Fig. 6).

Overall, the population assignment of *Legionella* genomes was correlated to the groupings of ML-based phylogeny (Fig. 1a), BAPS, and fineSTRUCTURE, even though there were differences in population assignments at the lowest resolution within a clade, mainly due to methodological differences in the sensitivity of each method in assigning into populations. fineSTRUCTURE grouped Lp into 9 groups. However, BAPS clustered Lp serogroups into 7 groups. The main difference was that one large cluster assigned by BAPS included Lp2, 4, 5, 7, 9 11, 14, and 16 but were split into three groups by fineSTRUCTURE. In addition, fineSTRUCTURE grouped Lp1 Paris into a single cluster whereas BAPS grouped it with Lp HL06041035 and Lp13. While BAPS grouped species in Clade 2 into 4 groups, fineSTRUCTURE grouped them into 5. The main differences were that BAPs grouped *L. erythra* and *L. birminghamensis* into two separate populations while fineSTRUCTURE assigned them to a single population. Similarly, fineSTRUCTURE assigned *L. jordanis* and *L. nautram* into 2 singleton populations but BAPS grouped them together into 2 separate populations along with 2 other *Legionella* species in each of the populations. Clade 3 species were grouped into 4 populations in the BAPS analysis while fineSTRUCTURE grouped them into 6 populations by splitting the BAPS populations again into finer populations as well as re-assigning species into different populations. The single Clade 4 species, *L. rowbothamii*, was s distinct population in BAPS analysis (Fig. 5).

### Patterns of DNA admixture within the *Legionella* genus

Based on the fineSTRUCTURE coancestry matrix visualized as a heatmap (Fig. 6), there appeared to be genetic exchange events occurring within and across each *Legionella* species, especially between Clades 2 and 3 but few events between Lp and other species. The color of each cell of the matrix indicated the expected number of genetic markers imported from a donor genome (x-axis) to a recipient genome (y-axis). Clade 3 species *L. wadsworthii, L. cherri* and *L. dumoffii* were admixed based on fineSTRUCTURE analysis (Fig. 6), although BAPS (Fig. 5) showed little evidence of DNA imports into these three genomes. All *Legionella* genomes in Clade 2 except *L. maceachernii* and *L. micdadei* showed signs of admixtures across all genomes in Clade 3 according to fineSTRUCTURE (Fig. 6), but BAPS analysis did not reveal any admixtures. The only sign of admixture predicted by BAPS analysis, excluding Clade 1, was between Clade 3 species *L. anisa* and *L. bozemanii* serogroup 2, which was also found by fineSTRUCTURE. Of the 25 Lp genomes in Clade 1, BAPS analysis revealed admixture signals in eight genomes: Lp1 Philadelphia, Lp LPE509, Lp 570-CO-H, and Lp3, Lp5, Lp11, Lp14 and Lp15. fineSTRUCTURE analysis also indicated small levels of admixtures across some Clade 1 genomes, which was not as evident as in BAPS.

Both BAPS and fineSTRUCTURE analysis revealed the absence of DNA exchange between Clade 1 and the other clades, indicating a possible sexual isolation of Lp serogroups from other *Legionella* species. To understand the nature of recombination predicted by ClonalFrame analysis, we tried to assign the origin of each event by a BLASTN pipeline described in Methods. However, recombinant sequences could not be matched to a particular ancestor in any other *Legionella* species.

**Figure 7:**
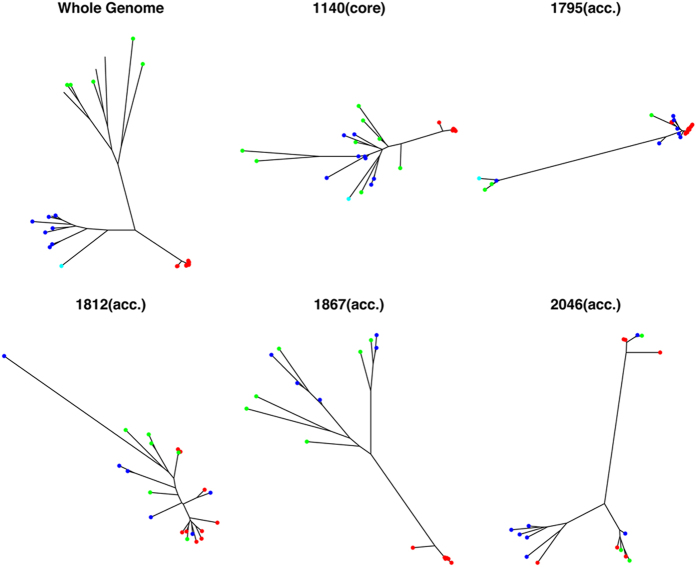
Examples of divergent gene family/cluster–based phylogenies that indicates clade specific gene transfer or Horizontal Gene Transfer (HGT) events. Clades 1 2, 3 and 4 are labeled with red, blue, green and cyan, respectively. There were a total of 129 core genes and 187 accessory genes that showed evidence of HGT events. From clockwise, top left: the whole genome *Legionella* species phylogeny, a core gene with incongruent phylogeny and 4 example accessory genes. The phylogenies shown are as follows: 1140 - peptide chain release factor 1; 2046 - integrating conjugative element protein PilL, PFGI-1 class; 1867 - Lysine-arginine-ornithine-binding periplasmic protein precursor; 1812 - Helix-destabilizing protein; and 1795 - RlpA-like protein precursor. The complete list and phylogenies of the incongruent/divergent core genes (Supplementary Figure 3a) and accessory genes (Supplementary Figure 3b) are shown in the Supplementary material.

As an alternative approach to identify potential admixture (and possible HGT), we created phylogenies for all gene families and then screened for phylogenies where there was an absence of congruity with the general pattern for the genome (see methods; Fig. 7). We screened for clade monophyly using the monophyletic function of the R *ape* package^42^ as well as the TOPD/FMTS^43^ tree congruence tool. Potentially incongruent trees were verified by manual inspection. For rapid processing, we used UPGMA distance based trees as we were comparing features of the gross topology^44^. Of the 1140 core genes, only 66 were found to have phylogenies with the whole genome (Supplementary Figure 4a). All of the inconsistencies fell in Clades 2 and 3: there was no evidence of an allele from another clade within the Lp species. For accessory genes, the rate was higher but these were still a relatively small proportion of the whole (145/2379) (Supplementary Figure 4b). In this case, 31 genes had a polyphyletic distribution. These were either genes that encoded hypothetical proteins, or functions associated with plasmids and phages.

## Discussion

Over the past 10 years, comparative genomics studies have been piecing together the ways in which individual bacterial genomes are related to larger taxonomic groups. Because bacteria are very diverse and every taxon has unique aspects to its ecology and genetics, this work has been mostly an empirical effort, aimed at estimating some of the basic parameters of evolution. Here, we characterized the genus level pan-genome, species-wide core genome and deciphered features of the natural history of *Legionella* based on comparative analyses of 43 genomes, encompassing all those that commonly or rarely cause human disease^6^.

We determined 1140 genes as the ‘perfect’ core genome in *Legionella* species. Previous estimates of core genome sizes are available only for Lp1 where 8^15^ and 27^45^ genome comparisons estimated the size to be 2405 and 2173 genes, respectively. A very recent study^18^ calculated the presence of 3120 core genes based on the comparison of 69 strains of Lp1, which were part of outbreaks occurring in Alcoy, Spain, over 11 years, in addition to nine Lp1 reference strains. This latter analysis did not capture the ‘true’ estimate of core genes because a distribution of *Legionella* species was not included.

One of the principal findings of our work is that *Legionella* phylogeny resolve into four distinct clades based, in general, on human host-virulence demarcations (Fig. 1A). The obvious question to ask is, what are the genetic features that distinguish these groups of species? Clade 1 comprises only the Lp serogroups, which are responsible for approximately 92% of LD cases. Clade 2 are rarely associated with human disease while Clade 3 species are associated with LD and Pontiac Fever with *L. longbeachae* causing ~30% of LD cases in Australia and New Zealand. Clade 4 contains the only legionella-like amoebal pathogen (LLAP) species in the database, *L. rowbothamii*, which is associated with co-infection with other pathogens and is unlikely to be the sole causative organism in human disease. We did not find evidence to suggest adaptation through acquisition of known metabolic pathways. However, the function of many accessory genes is unattributed at this time, so we may be missing some of the picture through our partial existing knowledge.

The four clades were distinguished by differences in genes that fell into GO categories such as DNA exchange (probably species-specific phages, transposons and plasmids) and signal transduction. Recent genome analysis has shown that the repertoire of secreted effector proteins with highly variable protein domains is largely species-specific^19^. Based on our PCA analysis, the species within each clade formed non-overlapping groups and were also more similar regarding accessory gene families. This suggests that *Legionella* as a group may use broadly similar and conserved machinery for intracellular infection and growth in humans as in a myriad of soil and water protists^5^ but differ from each other in the presence of genes that promote adaptations to specific niches by providing environmental sensing and shifts in virulence. For example, Clade 1 represents all Lp serogroups. For Lp1, a recent study of 21 clinical isolates (ST191) from an outbreak of LD in Scotland identified mutation, recombination and HGT that had occurred in the environmental *Legionella* population before human infection^46^. WGS of the isolates revealed three regions of high SNP density and four distinct subtypes. Included in the set of genes transferred horizontally were those encoding the Lvh T4ASS located on a plasmid-like element that has been implicated in host cell entry and intracellular replication^28^ at low temperature. Variation in clinical virulence was linked to strain-dependent differences in T4ASS^46^. In our study, the Lvh T4ASS was identified in only 10 genomes: 6 of 9 Clade 1 Lp1 genomes, and Clade 3 *L. bozemanii* serogroup 2 and *L. dumoffii*. Our findings suggest that Lvh T4ASS is not an essential virulence factor and confirms that the Lvh system is not widespread among *Legionella* species outside of Lp^47^,^48^. Additionally, we found that the P-type T4ASS was present in all *Legionella* genomes whereas the F-type T4ASS was missing in Lp2, 3 and 6, and in *L. cherii.* Such a heterogeneous distribution of these different T4ASS across the *Legionella* species is additional evidence of the plasticity of these genomes.

Clade 3 species include *L. longbeachae* that, along with Lp, accounts for over 95% of LD cases^8^. *L. longbeachae* was previously reported to contain an accessory gene, the tetracycline destructase, that was probably acquired by HGT from the soil, the natural habitat of this species^49^. The destructase confers tetracycline resistance to *L. longbeachae,* and has not been reported in Lp^50^ or any other *Legionella* species. While we found no evidence for the gene in any of the Lp1 or 16 other Lp serogroups, it was present in Clade 2 species *L. jordanis* and *L. nautarum* with 100% homology to the destructase in *L. longbeachae*. Since doxycycline is one of the first line drugs for treating legionellosis in the outpatient setting, presence of this gene may have led to the success of *L. longbeachae* in causing LD and to the success of the other species in causing human disease. *L. jordanis* has been association with human disease with a dozen or so recent cases reported^51^,^52^. The acquisition of the destructase coupled with these reports may indicate a shift in virulence and the tip of the iceberg in terms of their potential for increasing the prevalence of human disease.

Clade 4 includes *L. rowbothamii,* a species that was recognized in 2001^53^. The organism belongs to a group of species that were historically called legionella-like amoebal pathogens (LLAP) because of their obligate intracellular parasitism of protozoa and lack of growth in media that is used to isolate *Legionella*^54^. LLAPs have been isolated from various water supplies during investigations of LD^55^. However, while there is speculation and some data suggesting that LLAPs may be associated with human disease, they more often are found as a co-infection with other respiratory pathogens such as *Streptococcus pneumoniae* and respiratory syncytial virus^55^,^56^,^57^. *L. rowbothamii* is the only LLAP in our dataset and, therefore, it is not surprising that it forms a unique clade.

Adaptation to host species probably involves sensing the unique features of their intracellular environment and responding by producing secreted effectors that have actions, which are specific to their milieu. Because so little research has been done outside of the *L. pneumophila* species, we are unable to annotate most of the molecular machinery used in these other diverse *Legionella* species. *Legionella* host adaptation may parallel the situation found in *Chlamydiaceae*, another group of pathogens with a broad and diverse range of hosts, where adaptation is driven primarily by amino acid change in conserved proteins and secreted effectors under strong diversifying selection^58^. Indeed, a large number of diverse effectors have recently been identified among 38 *Legionella* species^19^.

In this study, we uncovered the patterns of DNA shuffling between *Legionella* species. We showed that, in the core genome, linkage disequilibrium breaks down with increasing genetic distance between markers and that each strain contained regions of its genome most likely to be affected by homologous recombination. Evidence for recombination within the Lp species has been noted previously^17^,^18^,^45^. As expected, the BAPs population structure analysis predicted admixture within Lp populations (Fig. 5). However, our analyses suggest that homologous recombination is almost exclusively an intraspecies event. We could not trace any sources of the potentially recombinant regions identified by ClonalFrame to a source outside of Lp. Population structure analysis in general offered little evidence of interspecies DNA admixture with the odd exception of the *L. bozemanii* serogroup 2 strain (Fig. 6). Although most accessory genes showed little evidence of interspecies transfer, a significant minority (144/2379) had a phylogenetic pattern consistent with HGT between *Legionella* spp. We also found near identical versions of two Lp plasmids in other *Legionella* clades. This pattern of gene flow, mostly occurring within species but with rare instances of sharing between congeners, has been termed ‘fuzzy species’^59^.

The mechanisms of recombination that have been shown for Lp include transformation and conjugation (reviewed in ref. 60). It is generally held that Lp1 strains that produce type IV pili are naturally competent for DNA transformation. While indigenous conjugative plasmids have been isolated from clinical and environmental *Legionella* and there is a recognized chromosome-based system for plasmid transfer, the majority of Lp1 strains do not contain these plasmids, as mentioned above, and are therefore unlikely to be necessary for virulence^60^. The CRISPR spacer analysis revealed that there is apparently a large ‘dark’ *Legionella* pan-genome, consisting of non-chromosomal DNA, presumably bacteriophage, suggesting that the true size of the pan-genome is considerably larger than the model-based estimation of 30,275. Interestingly, our GO and CRISPR findings for the monomorphic Lp serogroups, which are predicted to have an almost closed pan-genome and very few unique strain-specific genes, suggest that there is selective acquisition of genes that have enabled the organism to defend against phage attacks and to survive in both environmental and protozoan/eukaryotic hosts. These data indicate that the pool of foreign DNA in the environment able to be incorporated into the species is much greater than the number of genes that have actually been fixed in the chromosome.

## Methods

### Genome Sequencing of *Legionella* serogroups and species

In total, 43 genomes representing Lp1 (Philadelphia, Paris, Corby, Lorraine, LPE509, and Alcoy) and Lp serogroups Lp2-17 (including Lp12 strain 570-CO-H), *L. pneumophila* subspecies (Thunder Bay and HL06041035), and 17 other species *L. longbeachae, L. bozemanii* (serogroups 1 and 2)*, L. dumoffii, L. micdadei, L. birminghamensis, L. brunensis, L. cherii, L. cincinnatiensis, L. erythra, L. jamestowniensis, L. jordanis, L. maceachernii, L. nautarum, L. rowbothamii, L. tucsonensis, L. wadsworthii,* and *L. anisa* were used for comparative genomics analyses of which 33 were sequenced in this study (Supplementary Table 1). Genomic DNA from the 33 serogroups and species was sequenced with both 454 and Illumina (76 bp single end) technology. Burnstein *et al*.^19^ recently genome sequenced different strains from the current study including *L. anisa, L. bozemanii, L. brunensis, L. cherii, L. cincinnatiensis, L. dumoffii, L. erythra, L. jamestowniensis, L. jordanis, L. maceachernii, L. micdadei*, and *L. nautarum.*

### Hybrid De Novo Assembly of Contigs

The two sets of sequencing reads (454 and Illumina) from each sample were assembled using a hybrid *de novo* assembly strategy. Illumina reads were trimmed for base sequence quality using FASTQC (Andrews S: FastQC: A quality control tool for high throughput sequence data. [ http://www.bioinformatics.babraham.ac.uk/projects/fastqc/]). Per base sequence quality control (QC) cutoffs of 20% were used to trim illumina reads and then assembled into *de novo* contigs using VelvetOptimiser^61^, which is a wrapper that uses the Velvet denovo assembler and automatically estimates the optimal k-mer size that will produce the best possible assembly. The range of K-mer sizes specified were between 35 to 70. The best Illumina contigs obtained were then fragmented into shorter segments of ~400 bp, similar to the read length from 454. These 400 bp Illumina-fragmented contigs were then pooled with the reads generated from the 454 machine. A second *de novo* assembly using Newbler was then performed. The resulting *de novo* contigs were used for the downstream analysis mentioned below.

### Pan-genome analysis and genome alignment

Contigs from 10 published genomes and the 33 projects in this study were annotated using the Prokka^20^ prokaryotic annotation pipeline. For pan-genome analysis, the complete predicted protein coding sequences from all 43 genomes were searched against themselves using BLASTP with an e-value cutoff of 1e-05 for significance. The best BLASTP scores were used for identifying orthologous groups using the OrthoMCL algorithm^21^. A panmatrix using all orthologous gene was created and imported into the R-package, micropan, to generate visualizations to describe the *Legionella* pan-genome. MUSCLE^62^ was used with default settings to align genes and proteins within ortholog groups; each of the multiple sequence alignments (MSA) was filtered by GBLOCKS^63^ to remove gaps and highly divergent regions. Core genes are defined as the protein-coding gene clusters that have genes present from all 43 *Legionella* serogroups and species. All core protein coding MSA’s were concatenated to generate a super alignment for protein-based *Legionella* phylogeny. The whole genome nucleotide alignment was also generated using progressive MAUVE by concatenating all nucleotide sequences of each of the core genes^22^. This MAUVE alignment was used as input alignment for generating the whole genome nucleotide phylogeny, ClonalFrame, fineSTRUCTURE and BAPS analyses (see below). We also generated 2 separate MAUVE alignments: Lp only (intra-specific alignment; 1,028,806 bp); and all *Legionella* species (interspecific alignment; 759,392 bp) for a second version of ClonalFrame analysis (see below). The single nucleotide polymorphisms (SNPs) were extracted from the MAUVE core alignment based on reference strain Lp1 strain Philidelphia, and the core SNP positions were combined to prepare the genome-wide haplotype data. For all accessory gene clusters with at least 3 *Legionella* genomes present, MUSCLE MSAs were trimmed by GBLOCKS to remove gaps.

### Phylogenetic reconstruction

The ML phylogenetic reconstruction was implemented using RAxML^38^. Nucleotide phylogenies were reconstructed using the GTR (General Time Reversible) nucleotide substitution model, while the core protein coding phylogeny was generated using Jones-Taylor-Thornton (JTT) amino acid substitution model. For both substitution models the rate of heterogeneity was estimated with 4 discrete rate categories. Internal branches of the phylogeny were estimated with 100 bootstraps.

### Population structure using ChromoPainter, fineSTRUCTURE and BAPS

The ChromoPainter algorithm was applied to the genome-wide haplotype data using the linkage model^41^ to elucidate the population structure of *Legionella*. A recombination map file was created by specifying a uniform recombination rate per-site per-generation using a Perl script called makeuniformrecfile.pl. a preprocessing tool provided in ChromoPainter (http://www.paintmychromosomes.com). The output from ChromoPainter analysis is a co-ancestry matrix that summarizes the recombination-derived DNA imports and their donors across the 43 *Legionella* genomes. The fineSTRUCTURE algorithm uses the ChromoPainter generated co-ancestry matrix and performs model-based clustering using the Bayesian MCMC approach to explore population structure^41^. FineSTRUCTURE was run at a total of 400,000 iterations; the first 200,000 iterations were discarded as MCMC burn in. The thin interval was specified as 100.

To further gain insights into the population structure of these species, we used the BAPS software to establish the genetically differentiated groups and determine the amount of admixture between these groups. We ran the BAPS clustering model using the hierarchical manner to identify substructures inside the main clusters^64^. To find the optimal clustering, we ran five independent iterations with the prior upper bound of the number of clusters set to 35; clustering was performed with 4 levels in the hierarchy. The first level gave 10 clusters whereas the fourth yielded 20 clusters, for which we conducted a mixture analysis. We ran this analysis considering the minimum number of individuals for a population as one using parameters described by Castillo-Ramirez S. *et al*.^65^.

### Intragenic recombination analysis

To detect homologous intragenic recombination in the core gene clusters, we implemented the following three methods: (**1**) Pairwise Homoplasy Index (PHI); (**2**) Neighbor Similarity Score (NSS); and (**3**) Maximum^2^ using the PhiPack package^66^. For PHI, a window size of 50 nucleotides was used. For Maximum^2^, a fixed window size of two thirds of the number of polymorphic sites was used. P-values were estimated by employing 1,000 permutations for the three methods. Correction for multiple testing was performed using the Benjamini & Hochberg method implemented in the software Q-value^67^.

### Analysis of positive selection

Genes under positive selection were identified using codeml as implemented in PAML, version 4.8^30^. All gene clusters previously identified as having undergone recombination under any of the 3 intragenic recombination methods (described above) were removed for this analysis. We implemented the branch-site test2^68^ to identify genes under positive selection in each of the clades compared to other *Legionella* clades. For each core gene, the likelihood of a model that does not allow positive selection (null model) was compared to a model that allows positive selection (alternative model) using a Likelihood Ratio Test (LRT)^68^. One degree of freedom was used to calculate p-values, and correction for multiple testing was performed using the Benjamini and Hochberg method at a significance level of 0.05 implemented in the software Q-value^67^.

### Estimation of decay in linkage disequilibrium in the *Legionella* core genome

To illustrate the distance-dependent decay of linkage disequilibrium, we adopted the method implemented by Shapiro *et al*.^69^. We used the core orthologs (1140 genes) that are present in one copy per genome in each of the 43 *Legionella* serogroups and species, and each unique allele was assigned a unique allele number. We then selected pairs of loci separated by increasing distance in the *L. pneumophila* reference genome. Neighboring loci on the same strand were excluded. Linkage disequilibrium between 2 pairs of loci was estimated using the *D′A* metric^70^, which provides a summary measure of linkage disequilibrium between those two loci.

### Analysis of homologous recombination

ClonalFrame (version 1.2)^23^ was run at 40,000 iterations on the whole genome core nucleotide alignment identified by progressive MAUVE with all 43 genomes, and the initial half was discarded as Markov Chain Monte Carlo (MCMC) burn in. Good convergence and mixing properties were found between the four runs, making sure that they produced consistent estimates of the global parameters, clonal genealogy and location of recombination events. Additionally, for each reconstructed branch substitution event introduced by either mutation or recombination, the number of mutation events, and the number of recombination events were approximated. The relative effect of recombination and mutation on genetic change (*r*/*m*) and the relative rate of mutation and recombination (*ρ*/*θ*) were estimated. We also implemented two separate runs of ClonalFrame using the whole genome alignment of only the Lp serogroups (25 genomes) and non-Lp species (18 genomes) to obtain *L. pneumophila* specific recombination parameters.

We defined putative imports for each branch of the ClonalFrame tree as a genomic region for which the probability of recombination never goes below 80% and reaches 95% in at least one site. Such putative imported DNA segments were extracted from each of the genomes and, for each of them, we searched the GenBank nucleotide database for similarities with the highest normalized BLASTN score along with a percent identity (pcident) of at least 95% in the whole nucleotide BLAST database (updated February, 2016).

### CRISPR identification and spacer analysis

CRISPR sequences were identified using the online CRISPRs finding software (http://crispr.u-psud.fr/Server/). The software also searches the identified CRISPR sequences against the *cas* gene database. All spacer sequences were extracted and a sequence similarity clustering using dnaclust software was performed. BLASTN analysis against the NCBI nucleotide database as well as against the *Legionella* pan-genome proteome and the NCBI NR database were also performed.

### Reconstruction of gene gain and Loss

The analysis for the history of gene gain and loss was done by a stochastic mapping method implemented using the gainLoss program^32^. The program takes the patterns of absence or presence of gene clusters and analyzes the evolution of such observed phyletic patterns within the likelihood framework using a probabilistic evolutionary model that assumes the gain and loss of genes along the phylogeny that follow a Markovian continuous process, and infers probabilities and expectations for all gain and loss events in a per site per branch manner. The core genome phylogeny was used as the reference tree. The probabilistic evolutionary model used here assumed that the gain/loss ratio varies among sites (variable gain/loss ratio (mixture)). The total number of gains/losses for each branch was calculated as the probability of gain/loss for each gene cluster, summed across all the genes. The probability cutoff implemented to call a gene gained/lost at a branch of the phylogenetic tree was >0.98.

### GO Enrichment Analysis

GO terms were identified using BLAST2GO analysis^71^. In short, a single representative nucleotide gene sequence from each of the 12,977 gene clusters was selected and BLASTX was performed locally against the NR database (last updated February 2016). The BLASTX output file in XML format (E-value < = 1e-05) was then used to map the GO terms using BLAST2GO under default settings^71^. GO term enrichment analysis was performed for all the clade-specific gene clusters, genes gained along the terminal and internal branches, genes under recombination and genes under positive selection. GO enrichment analysis was tested using the GOEAST tool^72^ assuming our experiment was a customized microarray platform. Because there was only one species placed in Clade 4, we could not compute enrichment for this clade for positive selection analysis. The p-value of GO ID enrichment was calculated as the hypergeometric probability of getting a sample of genes (example: number of genes gained/under recombination/selection) under the null hypothesis that they were selected randomly from the total pool of 12,977 genes. To control error rates for multiple hypothesis testing, the p-values were adjusted using the Benjamini Hochberg method implemented in the software Q-value^67^ where a false discovery rate (FDR)-adjusted *p*-value < 0.05 was considered significant.

### Testing for potential Horizontal Gene Transfer (HGT) using gene phylogeny

All genes families with at least three members in each of Clades 1 to 3 were clustered using UPGMA. Each resulting tree was tested using the imonophyletic command of the R ape package^42^ and also using TOPD/FMTS (Version 3.3), where the former was implemented to determine if genes from the same clade formed monophyletic groups and the latter to check whether the topology of each of the accessory genes agrees with the topology of the *Legionella* species phylogenetic tree. For families that failed the screen, ML phylogenies were computed using RAxML^38^ and visually inspected for evidence of inter-clade gene movement.

## Additional Information

****Accession codes****: The data generated in this study has been deposited in the NCBI SRA database under the bioproject accession number SRP070825. All scripts are on figShare (link: https://figshare.com/projects/Dynamics_of_genome_change_among_Legionella_species/14567).

**How to cite this article**: Joseph, S. J. *et al*. Dynamics of genome change among *Legionella* species. *Sci. Rep.* **6**, 33442; doi: 10.1038/srep33442 (2016).

## Supplementary Material

Supplementary Information

## Figures and Tables

**Table 1:** Distribution of Type IV secretion systems in the 33 newly sequenced *Legionella* genomes in this study.

*Legionella* Species	Clade	Type IVB	Type IVA			GI-type*
		Dot/Icm	P-type	F-type	Lvh	
*L. pneumophila - 2 (D5762)*	1	X	X	—	—	—
*L. pneumophila - 3 (D5517)*	1	X	X	—	X	1
*L. pneumophila - 4 (D5739)*	1	X	X	X	X	—
*L. pneumophila - 5 (D5698)*	1	X	X	X	—	—
*L. pneumophila - 6 (D5864)*	1	X	X	—	X	2
*L. pneumophila - 7 (D5178)*	1	X	X	X	X	1
*L. pneumophila - 8 (D5744)*	1	X	X	X	X	—
*L. pneumophila - 9 (D4366)*	1	X	X	X	—	—
*L. pneumophila - 10 (D5602)*	1	X*	X	X	—*	1
*L. pneumophila - 11 (D797)*	1	X	X	X	X	2
*L. pneumophila - 12 (D4955)*	1	X	X	X	—	2
*L. pneumophila - 13 (D5677)*	1	X	X	X	X	—
*L. pneumophila - 14 (D4677)*	1	X	X	X	—	—
*L. pneumophila - 15 (D4613)*	1	X	X	X	—	—
*L. pneumophila - 16 (D5564)*	1	X	X	X	—	2
*L. pneumophila - 17 (D4954)*	1	X	X	X	X	1
*L. birminghamensis (D1407)*	2	X	X	X	—	—
*L. brunensis (D1635)*	2	X	X	X	—	—
*L. erythra (D3308)*	2	X	X	X	—	—
*L. jamestowniensis (D4855)*	2	X	X	X	—	—
*L. jordanis (D5875)*	2	X	X	X	—	—
*L. maceachernii (D5800)*	2	X	X	X	—	—
*L. micdadei (D5727)*	2	X	X	X	—	—
*L. nautarum (D4144)*	2	X	X	X	—	—
*L. anisa (D5641)*	3	X	X	X	—	—
*L. bozemanii - 1 (D5751)*	3	X	X	X	—	—
*L. bozemanii - 2 (D4398)*	3	X	X	X	X	—
*L. cherii (D3084)*	3	X	X	—	—	1
*L. cincinnatiensis (D3325)*	3	X	X	X	—	—
*L. dumoffii (D5637)*	3	X	X	X	X	1
*L. tucsonensis (D1087)*	3	X	X	X	—	—
*L. wadsworthii (D4735)*	3	X	X	X	—	—
*L. rowbothamii (D5054)*	4	X	X	X	—	—

*X indicates presence of that type of Type IV Secretion System.

*Indicates absence of that type of Type IV Secretion System.

*GI-Type is the genomic island associated Type IV Secretion System.
