# Supplementary material for: Dynamics of genome change among Legionella species
Source: Sci Rep. 2016 Sep 16;6:33442. doi: 10.1038/srep33442 (PMC5025774; doi:10.1038/srep33442)
Supplement: Supplementary Information [file srep33442-s1.pdf]

Supplementary Information

Dynamics of genome change among *Legionella* species

Sandeep J. Joseph[1], Daniel Cox[3], Bernard Wolff[4], Shatavia S. Morrison[4], Natalia A. Kozak-Muiznieks[4], Michael Frace[4], Xavier Didelot[5], Santiago Castillo-Ramirez[6], Jonas Winchell[4], Timothy D. Read[1,2,]* and Deborah Dean[,8,9,*,ǂ]

[1]Department of Medicine, Division of Infectious Diseases, [2]Department of Human Genetics and [3]Department of Microbiology and Immunology, Emory University School of Medicine, Atlanta, Georgia, USA

[4]Respiratory Diseases Branch, Centers for Disease Control and Prevention, Atlanta, Georgia, USA

[5]Department of Infectious Disease Epidemiology, Imperial College, Norfolk Place, London, United Kingdom

[6]Programa de Genomica Evolutiva, Centro de Ciencias Genómicas, Universidad Nacional Autónoma de México, Cuernavaca, Morelos, México[6];

[7]Center for Immunobiology and Vaccine Development, UCSF Benioff Children's Hospital Oakland Research Institute, Oakland, California, USA

[8]Department of Medicine and [9]Department of Biomedical Engineering, University of California, San Francisco, San Francisco, California, USA.

Supplementary Figures

Supplementary Figure 1. Whole genome protein phylogeny of *Legionella* species. The protein tree was constructed using maximum likelihood approach with the RAxML method using the concatenated MUSCLE alignment and GBLCOKS trimmed 299,244 amino acid residues of the 1140 translated core genes. The dark circles on the branches indicates branches with > 90 bootstrap estimates out of a total of 100 bootstrap estimates generated to reconstruct the internal branches of the phylogeny.

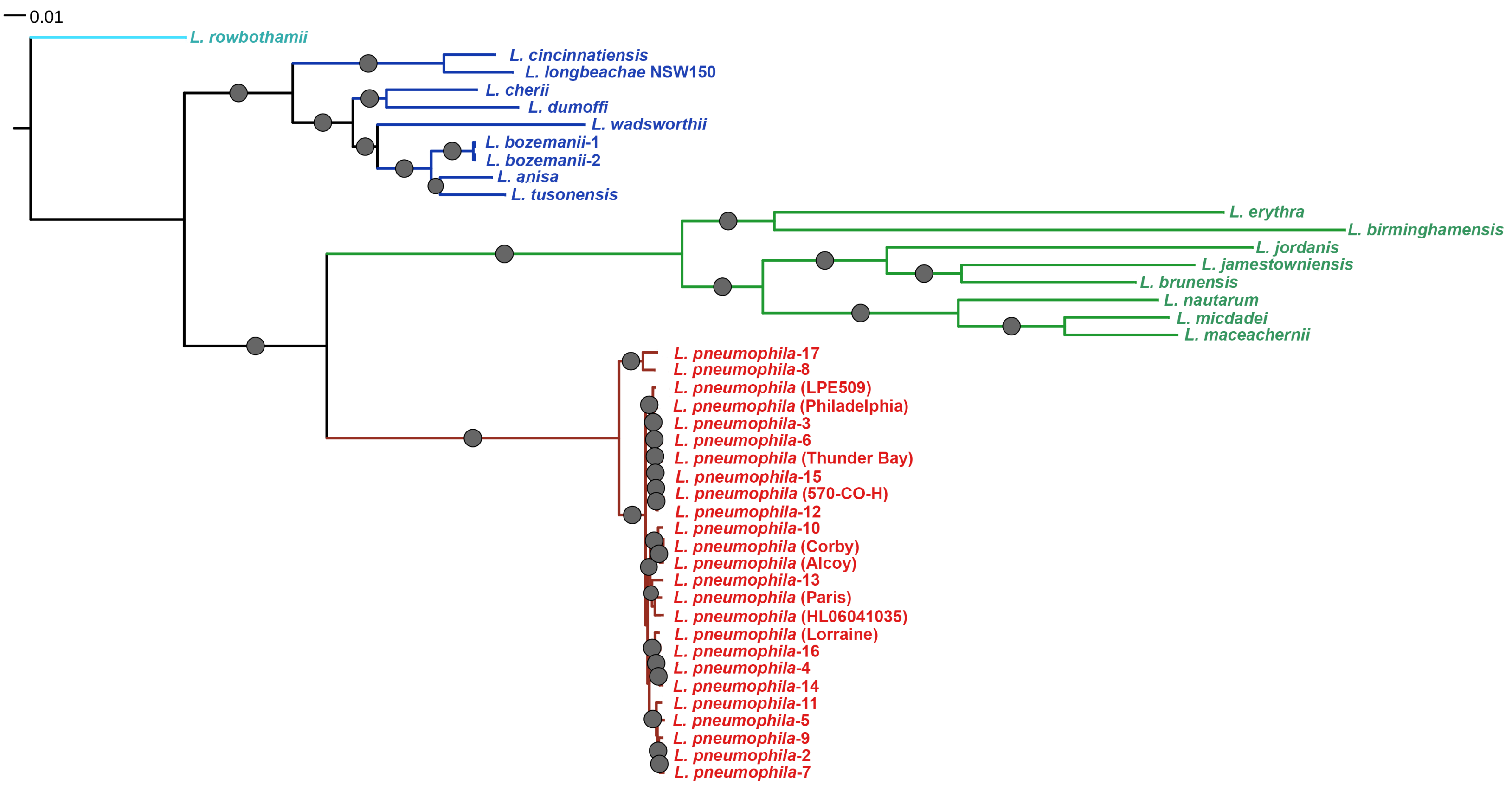

Supplementary Figure 2. Pan genome analysis of *Legionella* species. (**a**) Distribution of the presence or absence of gene clusters in *Legionella* species. Bar plot shows the number of gene clusters found in each of the 43 *Legionella* genomes used in this study. (**b**) Rarefaction curve of *Legionella* pan genome. The curve does not level out as more genomes are sequenced indicating an open pan genome for *Legionella* species. (**c**) Pie chart visualization of *Legionella* pan genome by proportion. This figure shows the pie-chart visualization of the fitted binomial mixture model, where gene clusters are assembled into groups having specific detection probabilities. The core gene clusters correspond to the darkest blue sector, with a detection probability of 1. There are also two other sectors with blue background color and detection probabilities close to 1, which are essentially core genes. The reason they are not present in all 43 genomes is because the genomes are mostly incomplete draft genomes. The greenish sectors represent the accessory gene clusters with a detection probability from 0.294 to 0.597, which are accessory genes. They are important in that they can be used to design typing schemes to identify *Legionella* at the species/strain level resolution. The orange/pink sectors are rarely occurring genes that are not conserved (detection probability ranges from 0.011 to 0.117).

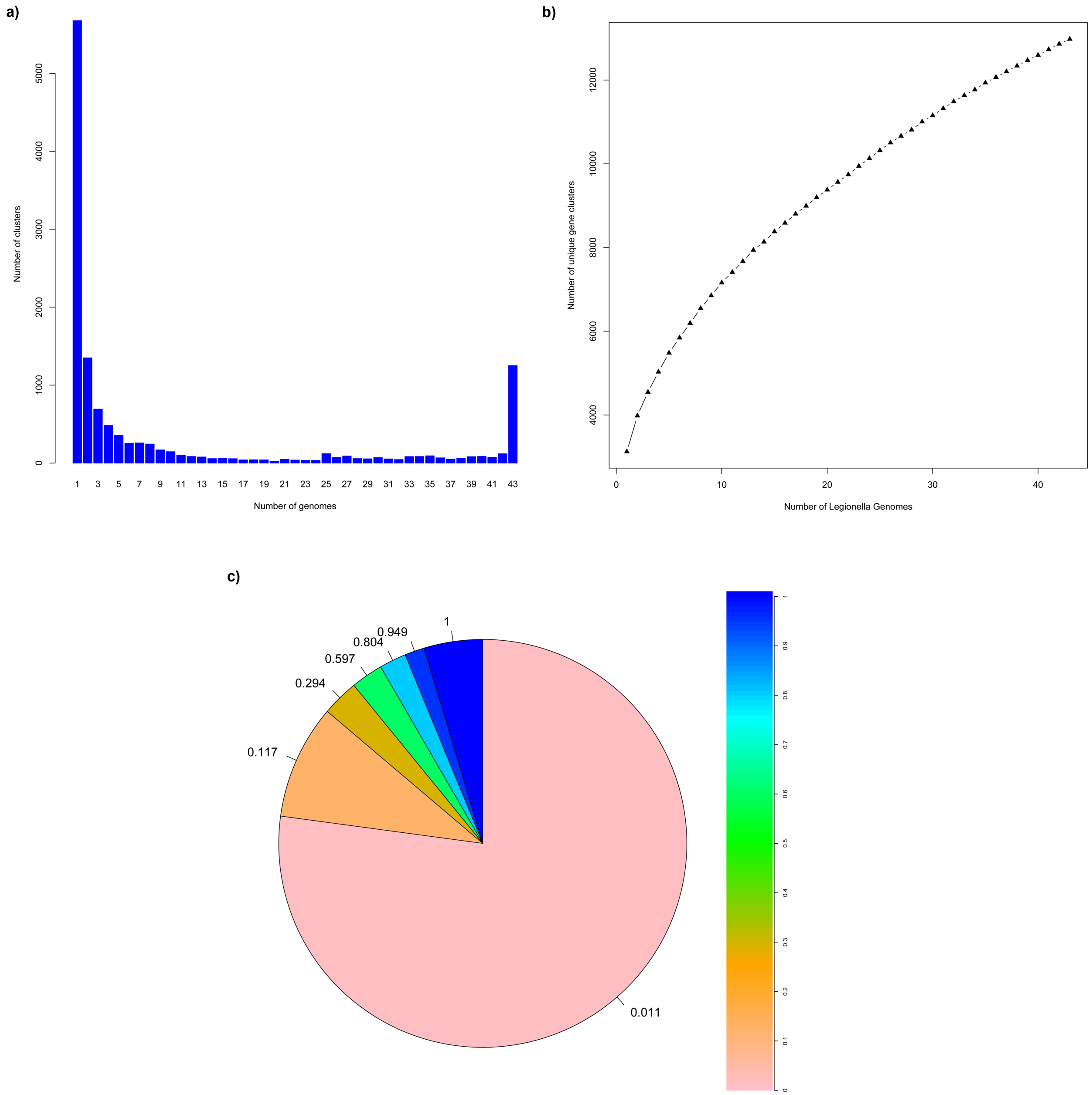

a)
Number of clusters
0
1000
2000
3000
4000
5000
1 3 5 7 9 11 13 15 17 19 21 23 25 27 29 31 33 35 37 39 41 43
Number of genomes
b)
Number of unique gene clusters
4000
6000
8000
10000
12000
0 10 20 30 40
Number of Legionella Genomes
c)
1
0.949
0.804
0.597
0.294
0.117
0.011

Supplementary Figure 3. Principal Component analysis (PCA) of the *Legionella* species gene family distribution matrix.

Each triangle represents a *Legionella* genome in the two first principal components of the pan genome matrix. The color indicates the 4 different clades identified in *Legionella* species; Clade 1 is shown in red, Clade 2 in green, Clade 3 in blue and Clade 4 (with a single species *L. rowbothamii*) in cyan. The percentages on the axis show how much of the total *Legionella* species pan genome-matrix variation is seen along each principal component.

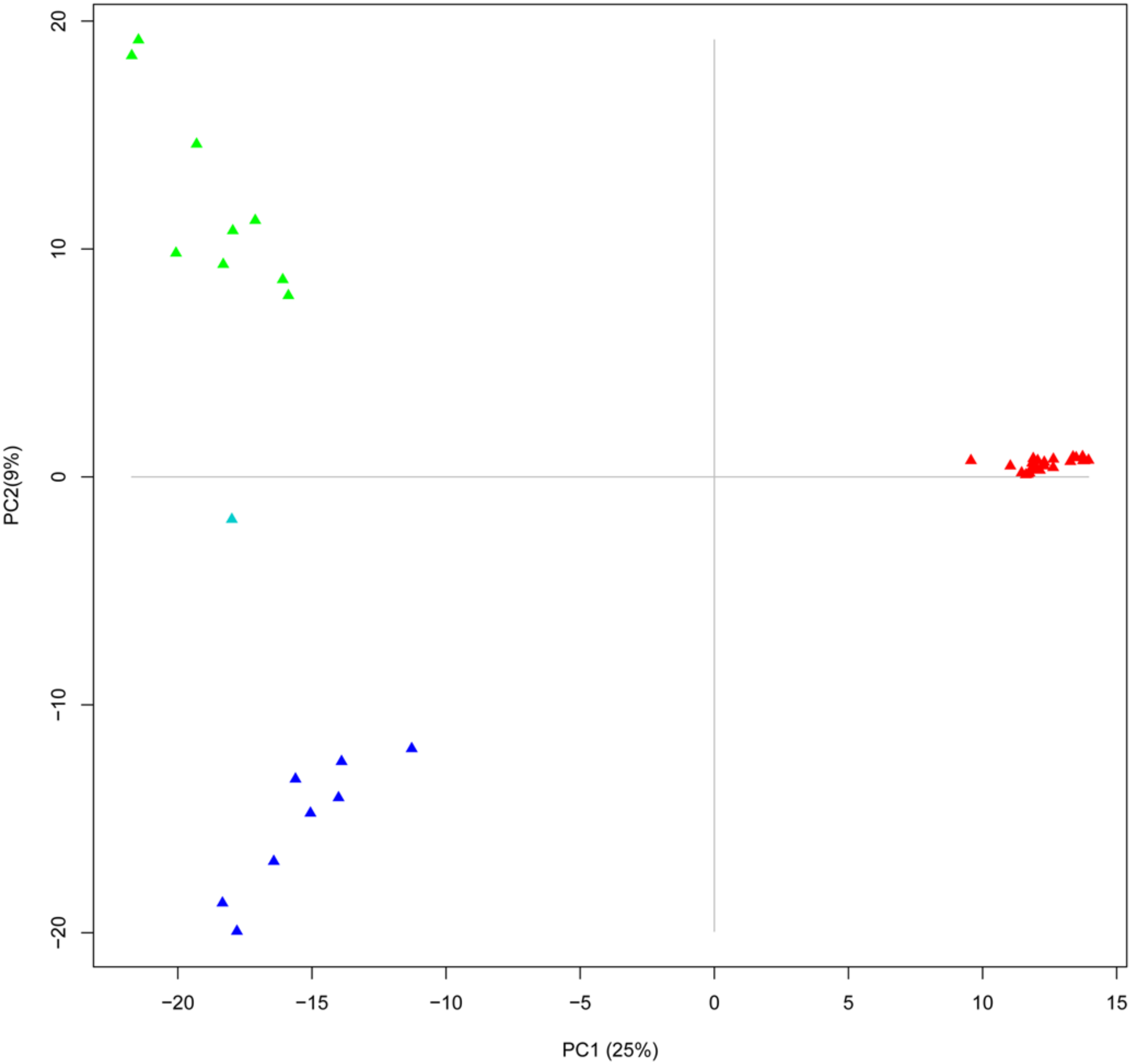

20
10
0
−10
−20
PC2(9%)
−20
−15
−10
−5
0
5
10
15
PC1 (25%)

Supplementary Figure 4A. **UPGMA phylogenies of all the divergent gene family/clusters identified.** *Legionella* core gene phylogenies that showed evidence of potential HGT using gene-based phylogeny**.**

Gene family 234 : putative DNA endonuclease SmrA

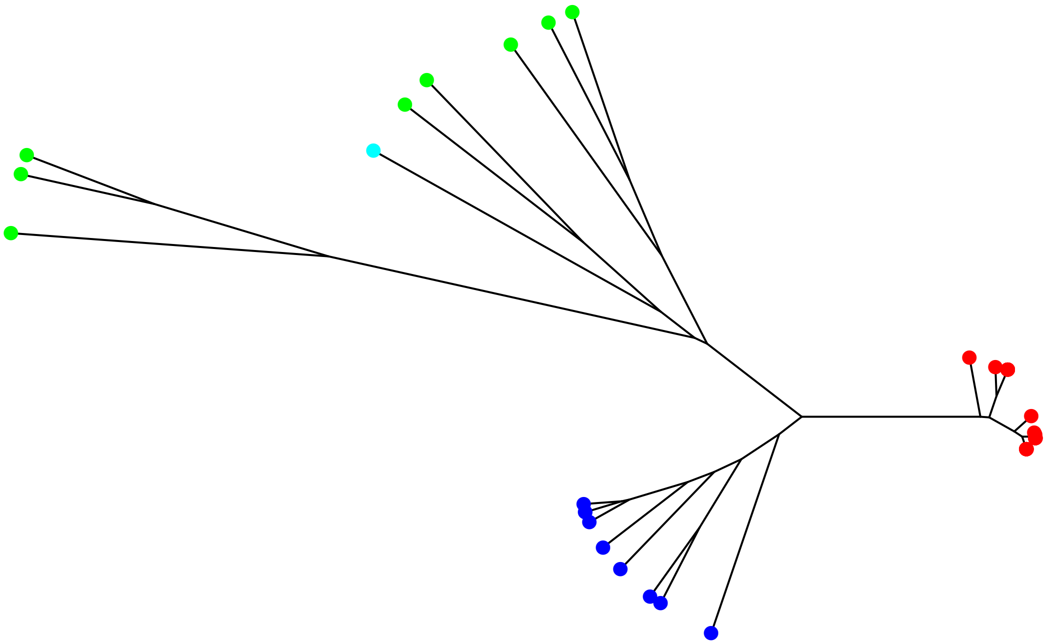

Gene family 360 : 50S ribosomal protein L32

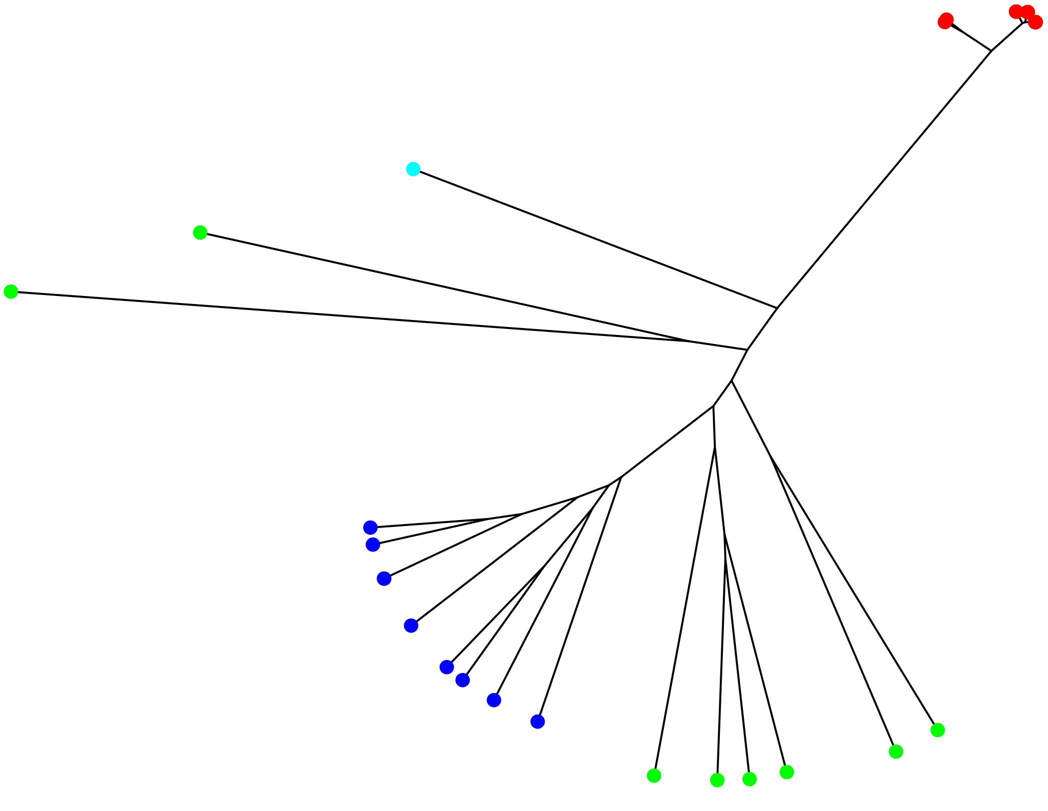

Gene family 366 : Ribonuclease HI

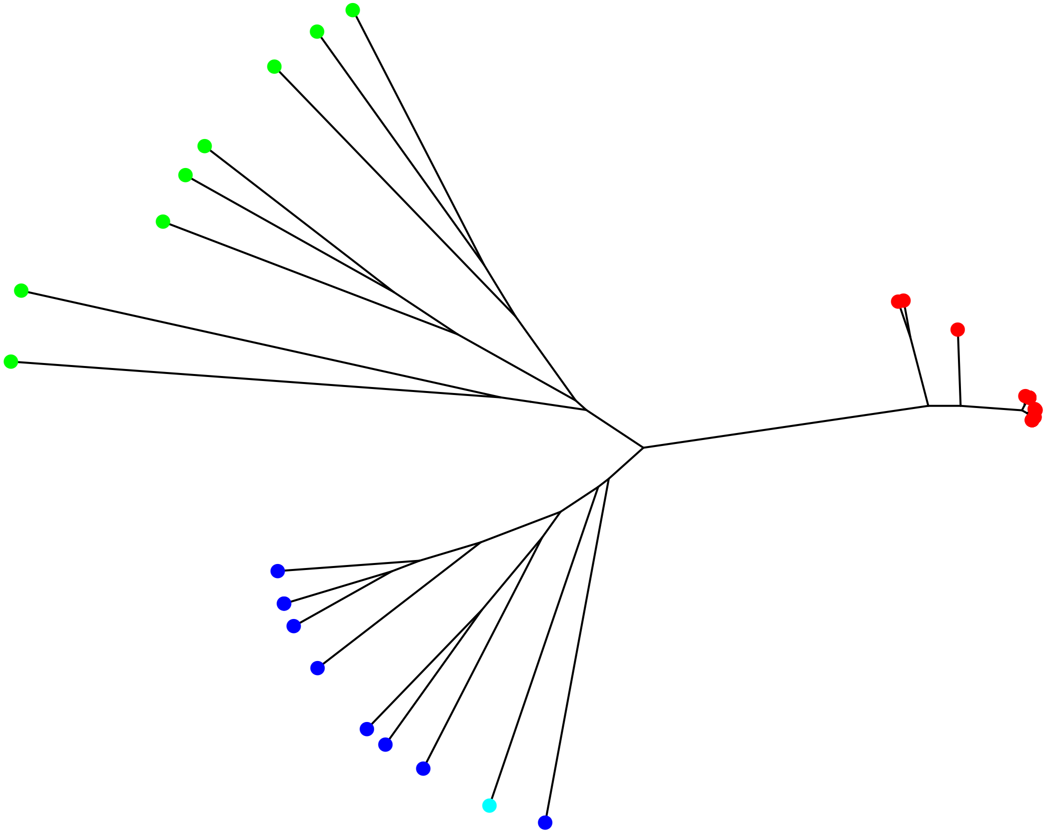

Gene family 373 : Dicarboxylic acid uptake system A

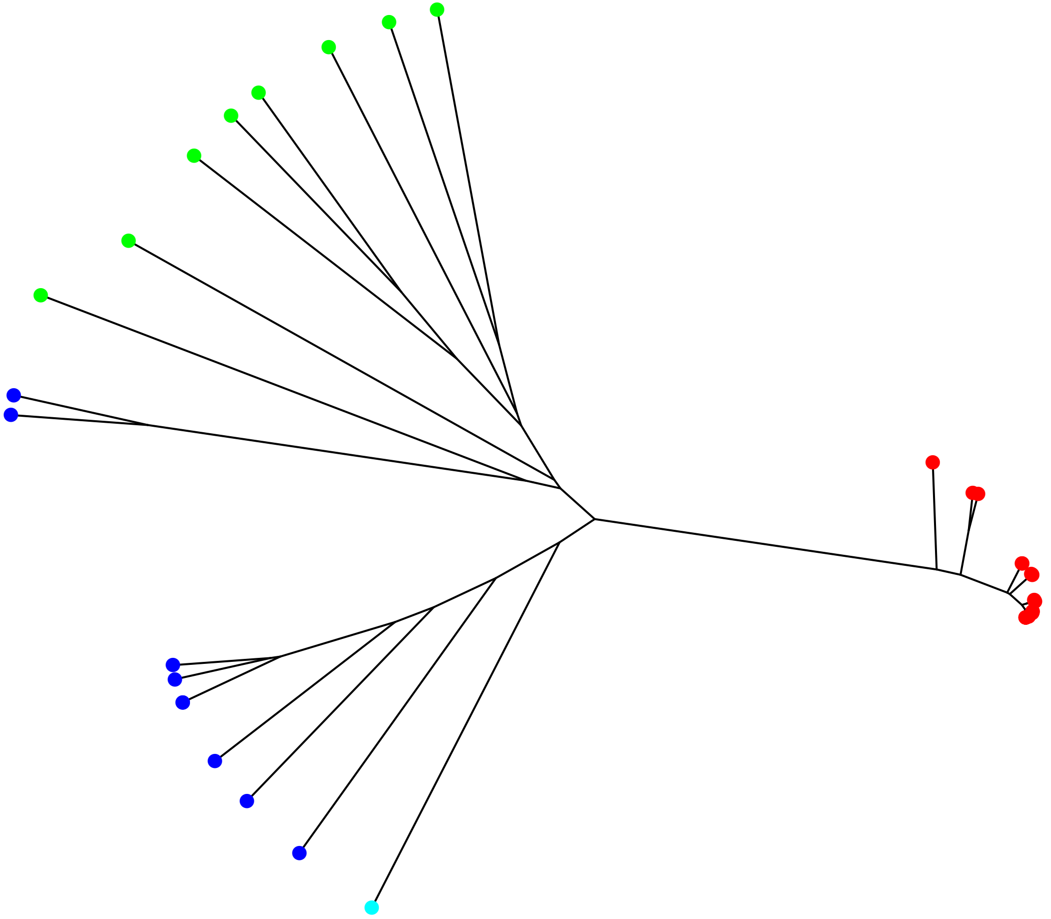

Gene family 380 : Rod shape−determining protein MreB

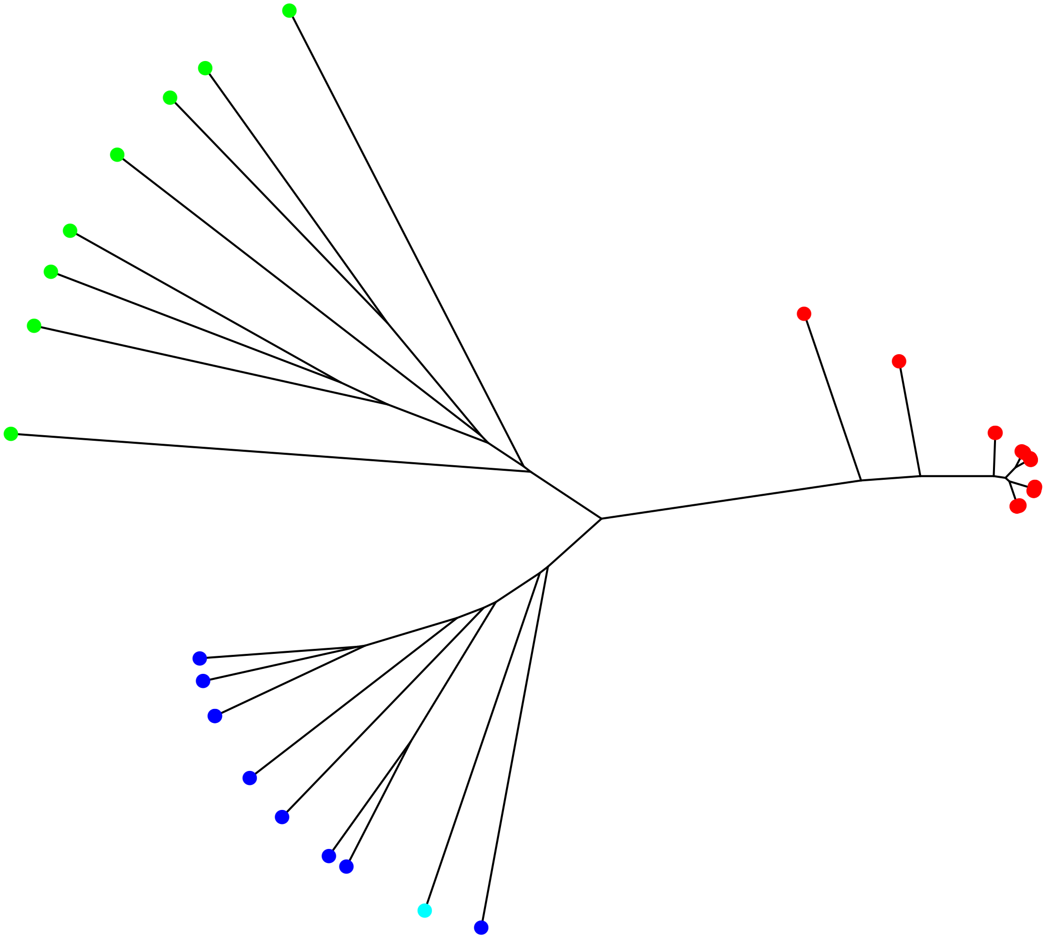

Gene family 399 : 3−deoxy−D−manno−octulosonate 8−phosphate phosphatase KdsC

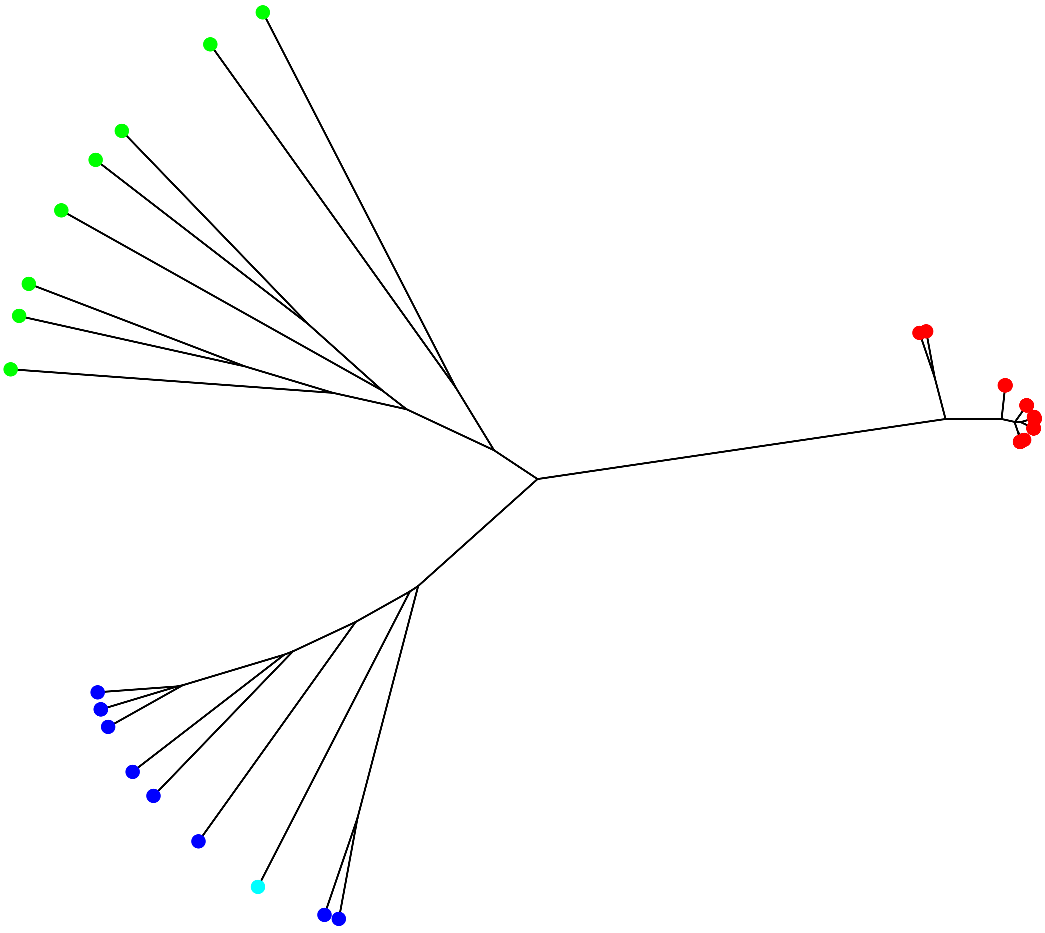

Gene family 411 : Cytochrome c−type biogenesis protein CcmB

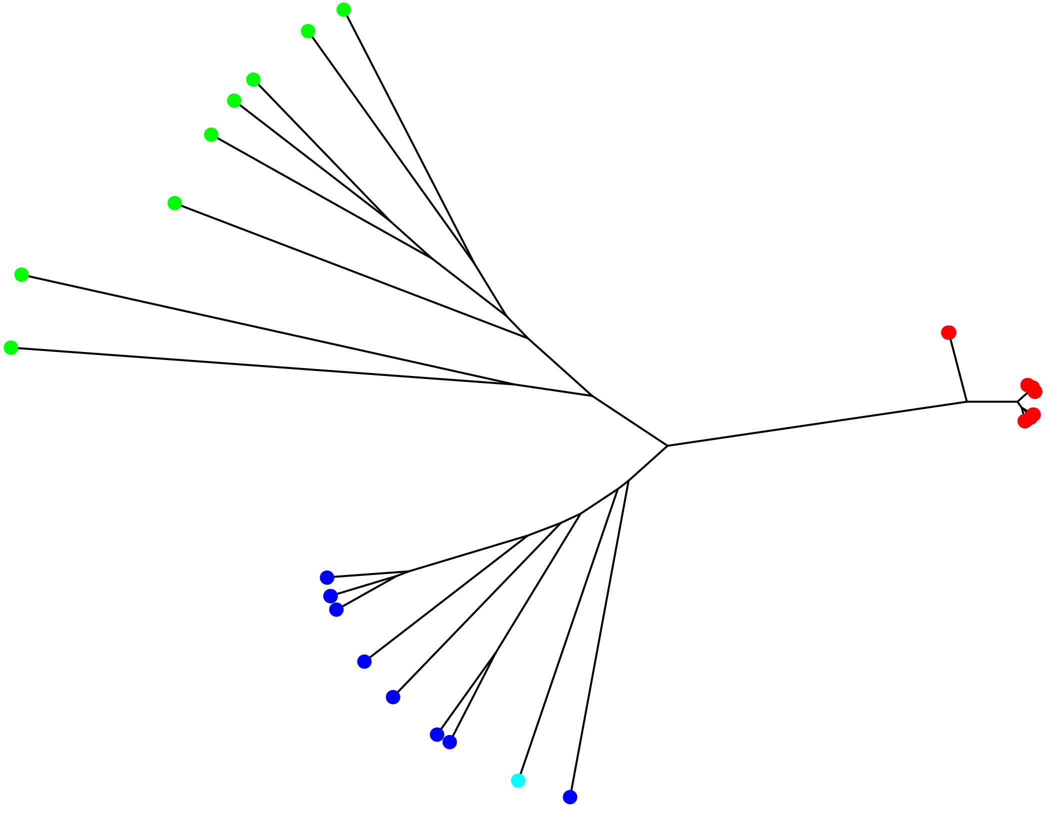

Gene family 443 : 50S ribosomal protein L9

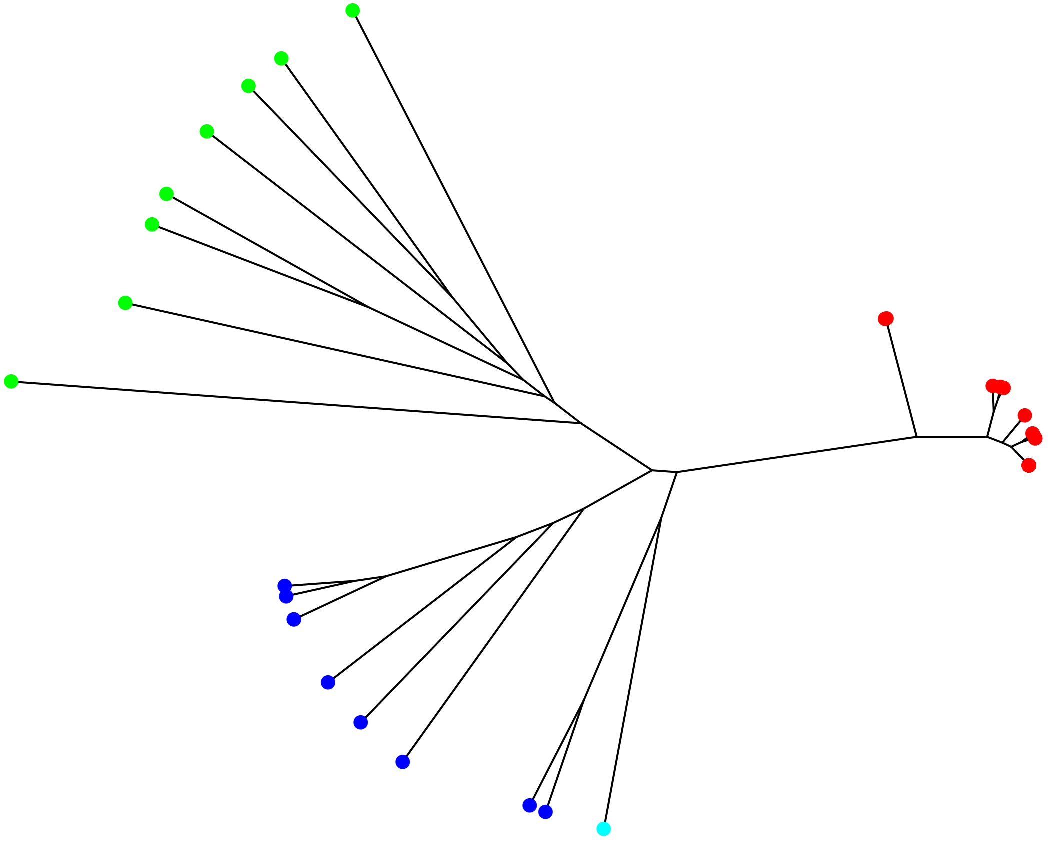

Gene family 476 : Rubredoxin−1

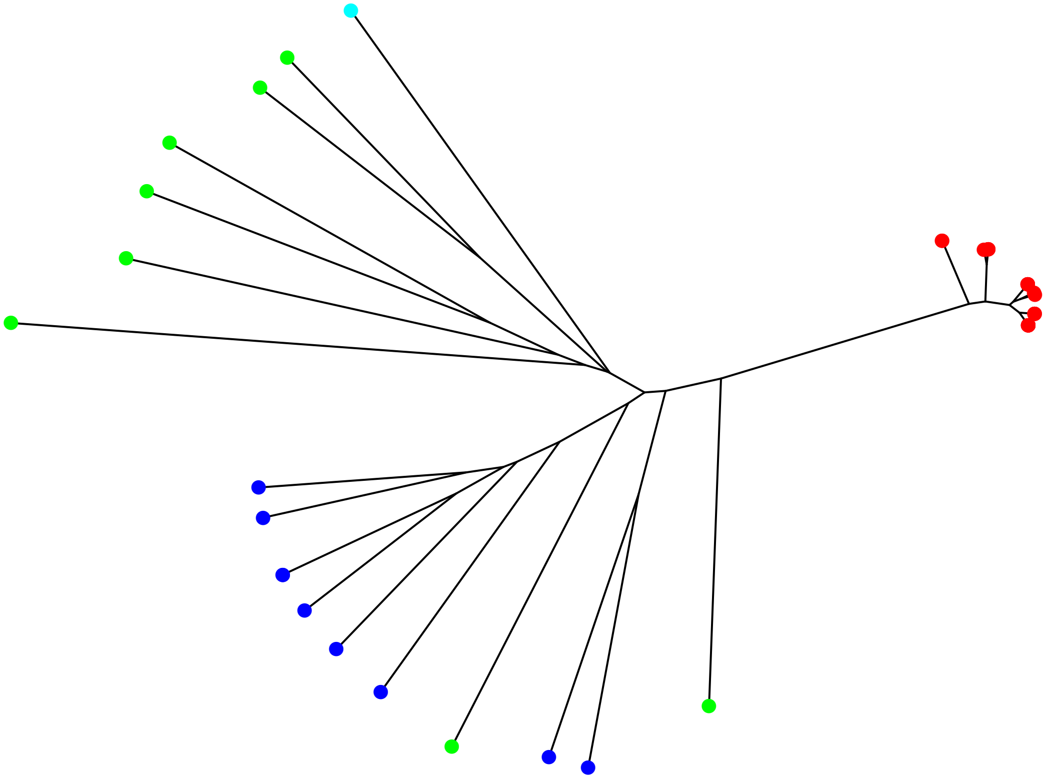

Gene family 485 : Ribosomal RNA large subunit methyltransferase M

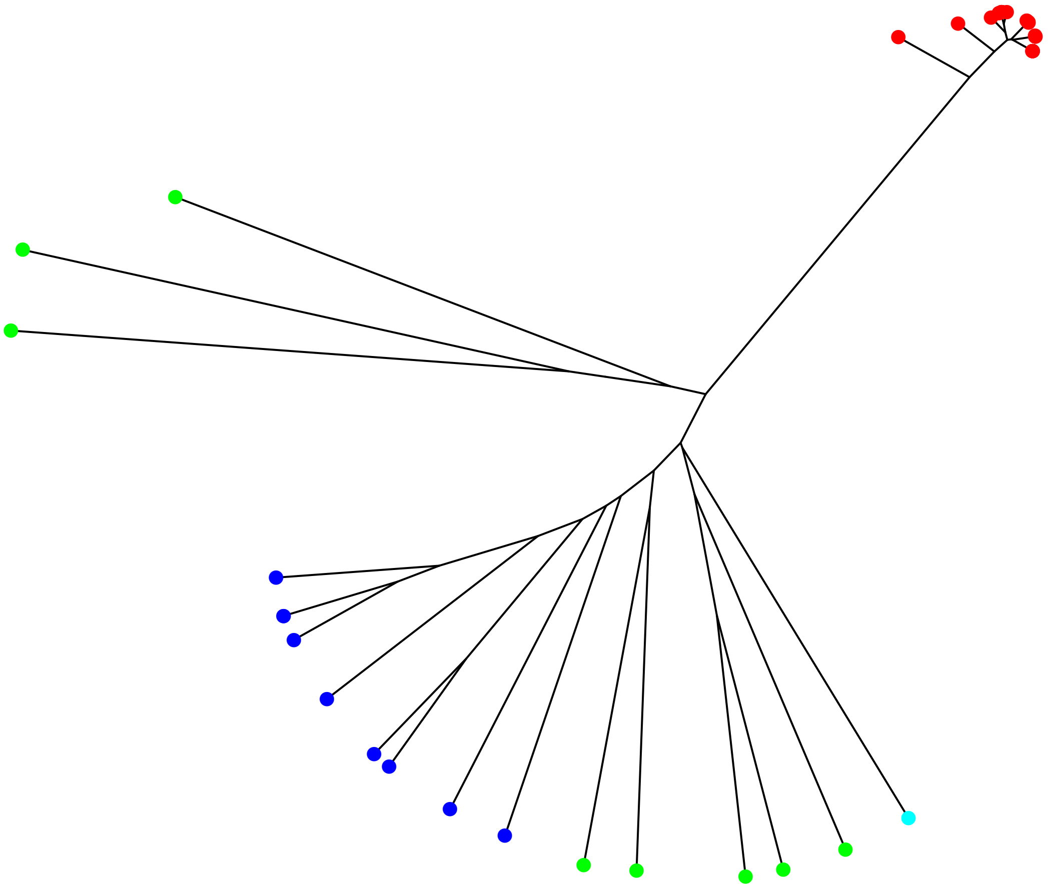

Gene family 486 : General secretion pathway protein F

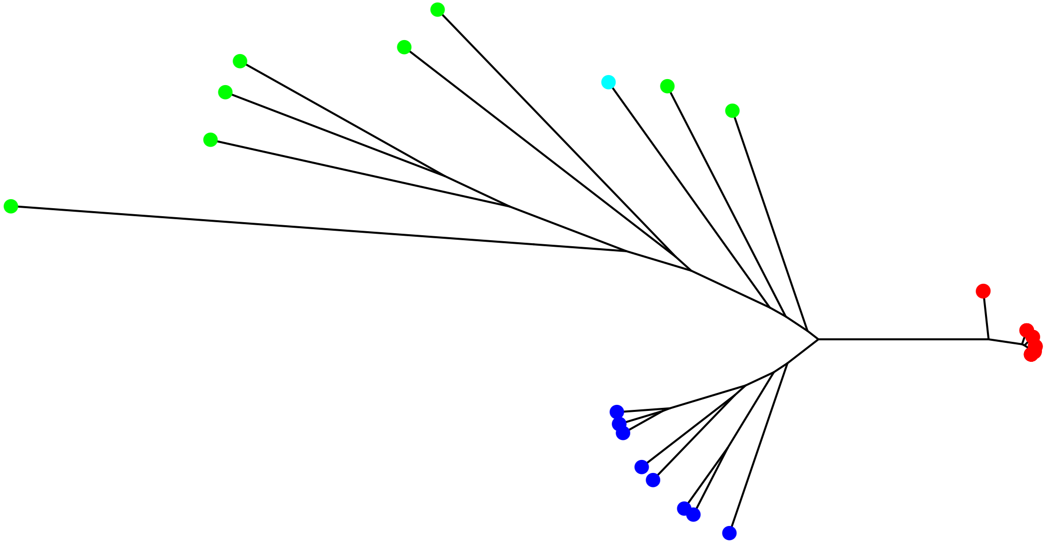

Gene family 487 : Type II traffic warden ATPase

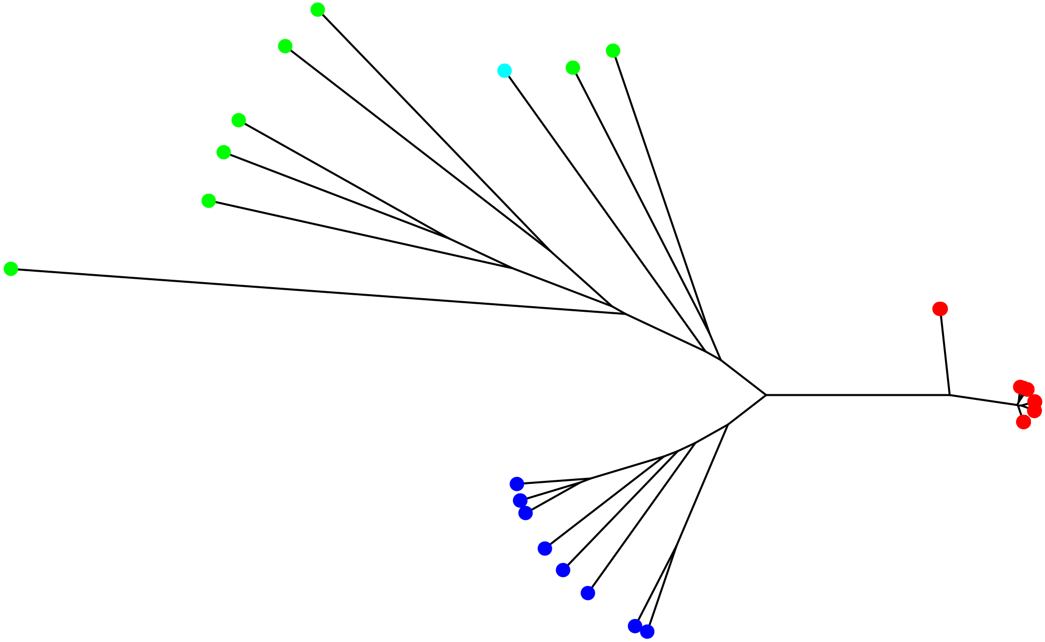

Gene family 491 : putative membrane protein

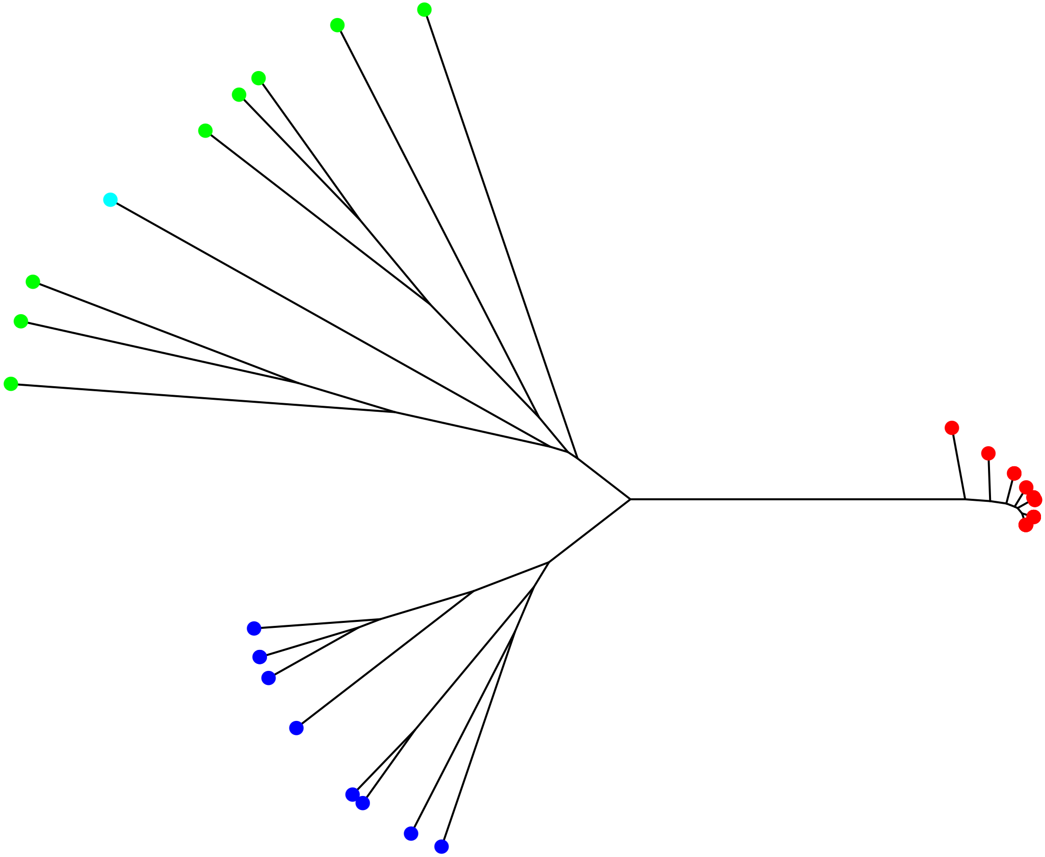

Gene family 536 : Nicotinate−nucleotide adenylyltransferase

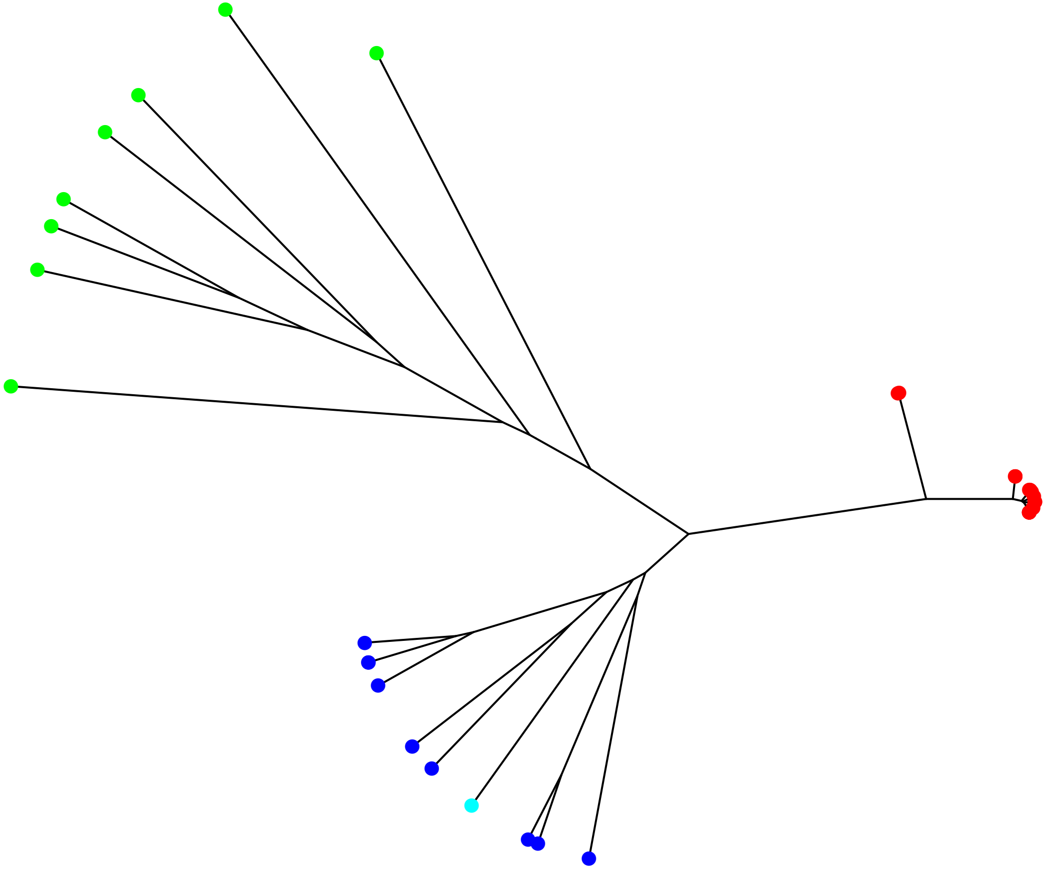

Gene family 556 : Iron−sulfur cluster assembly protein

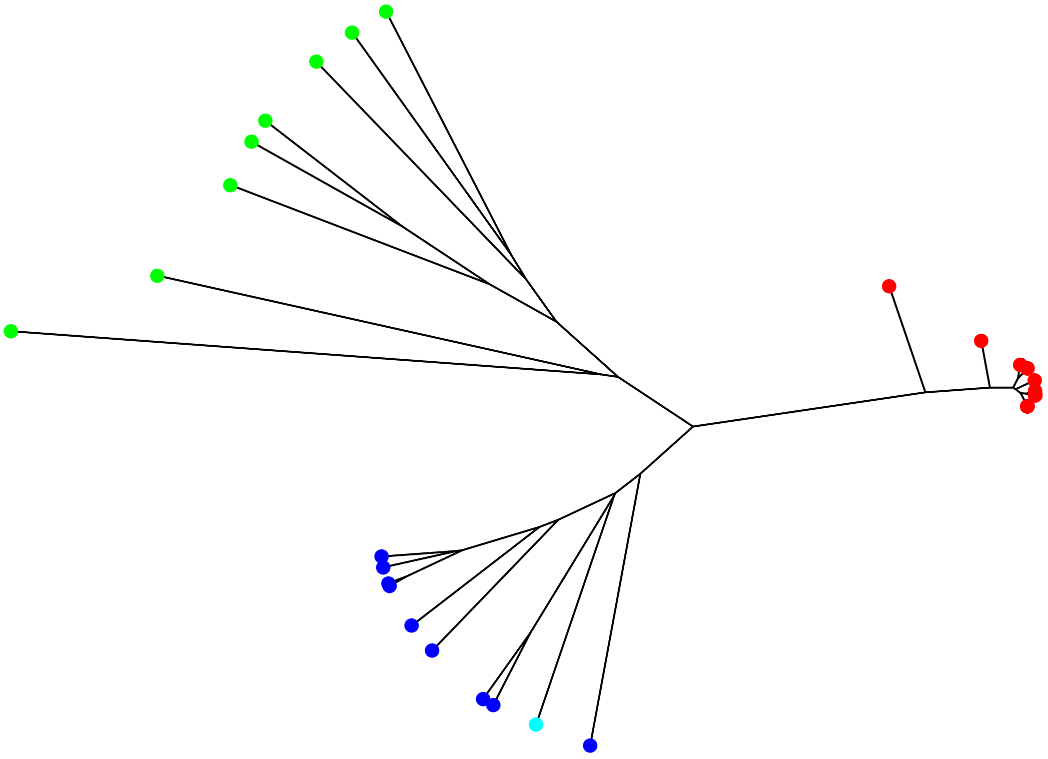

Gene family 561 : Arginine repressor

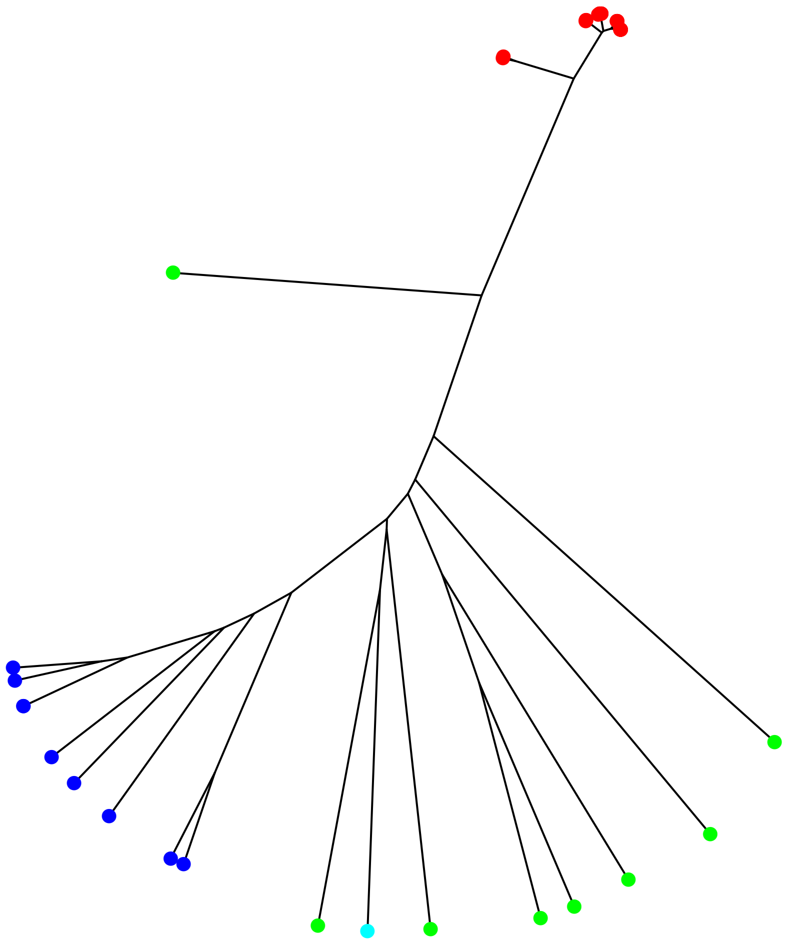

Gene family 602 : Host factor−I protein

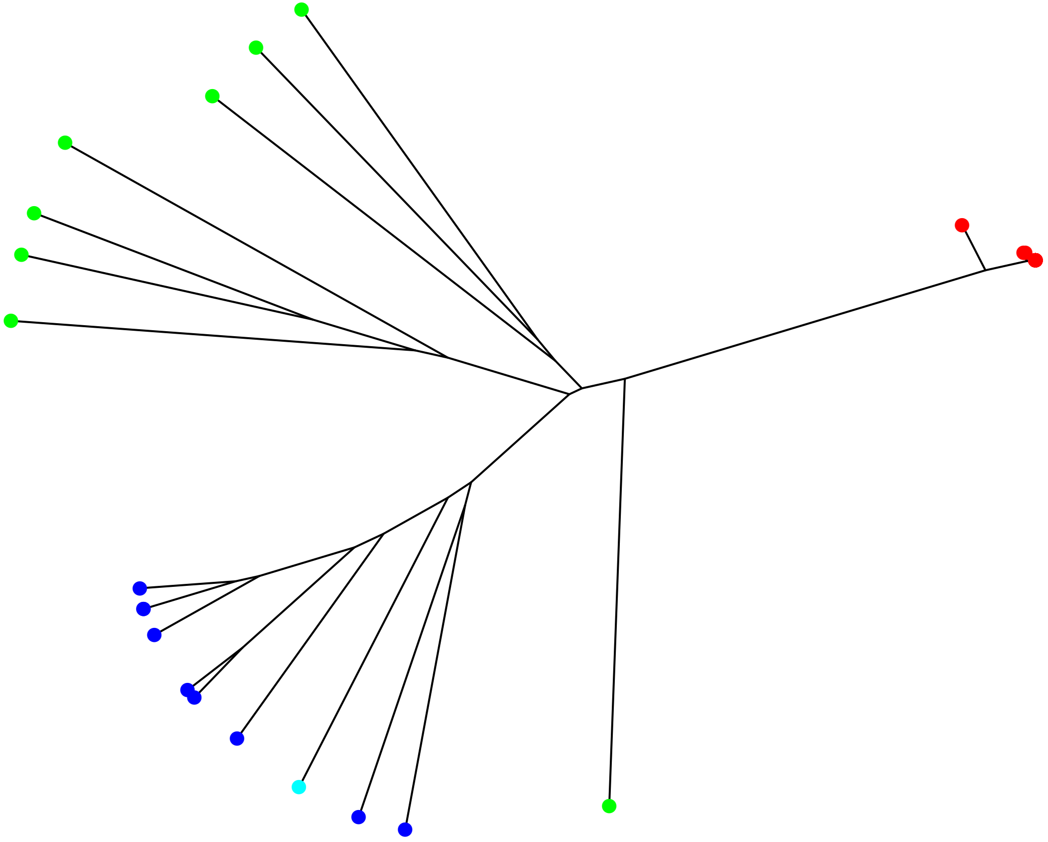

Gene family 611 : Low−affinity inorganic phosphate transporter 1

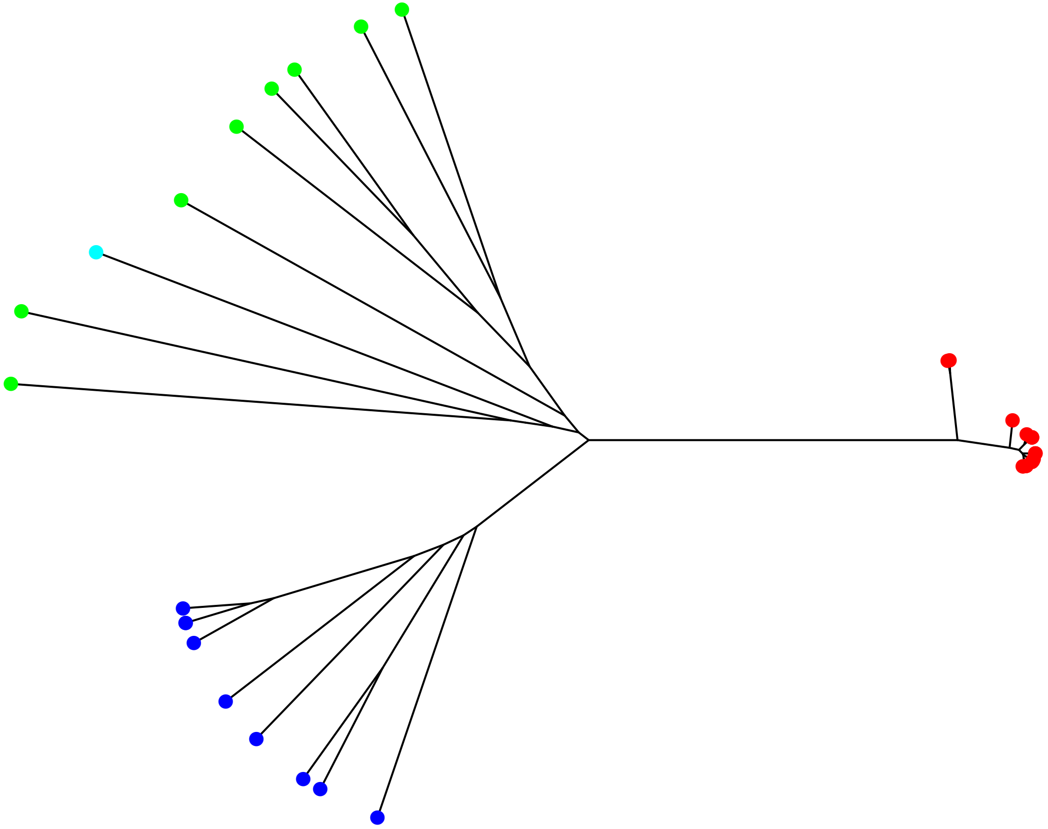

Gene family 621 : Modulator of FtsH protease HflK

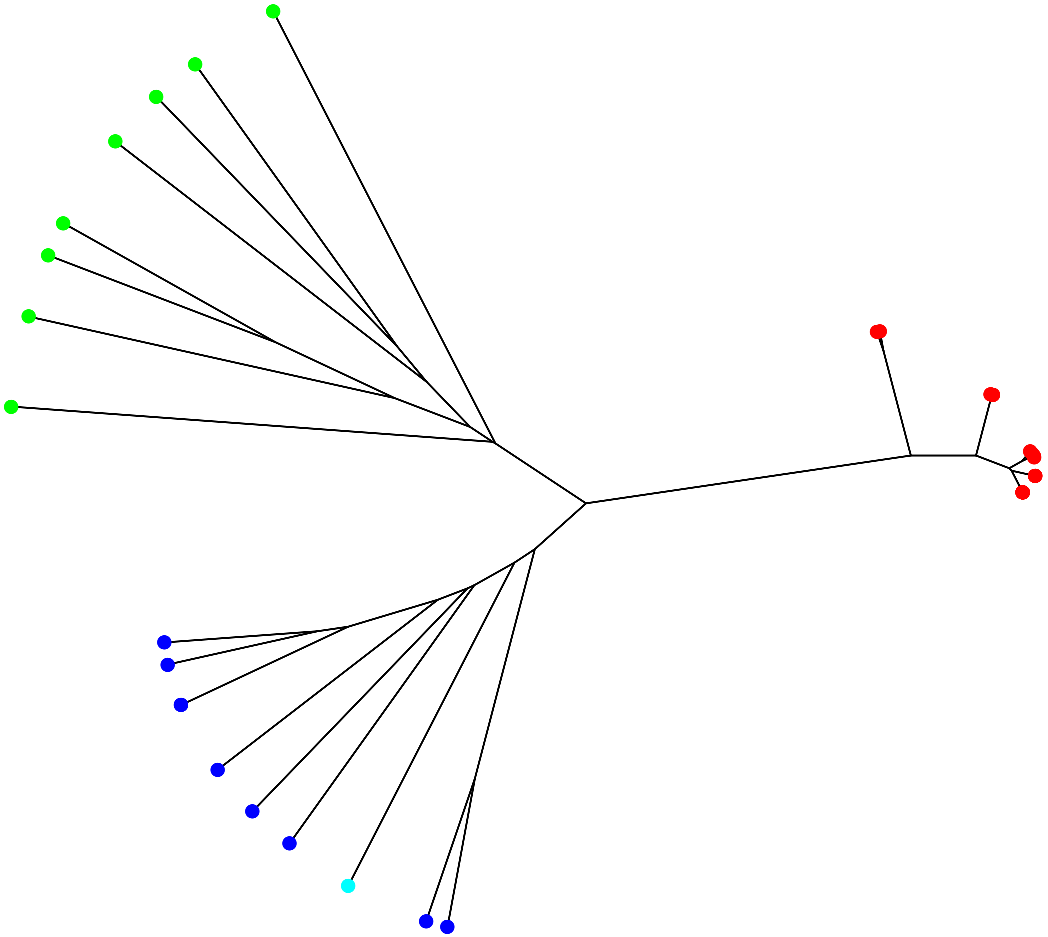

Gene family 622 : Deoxycytidine triphosphate deaminase

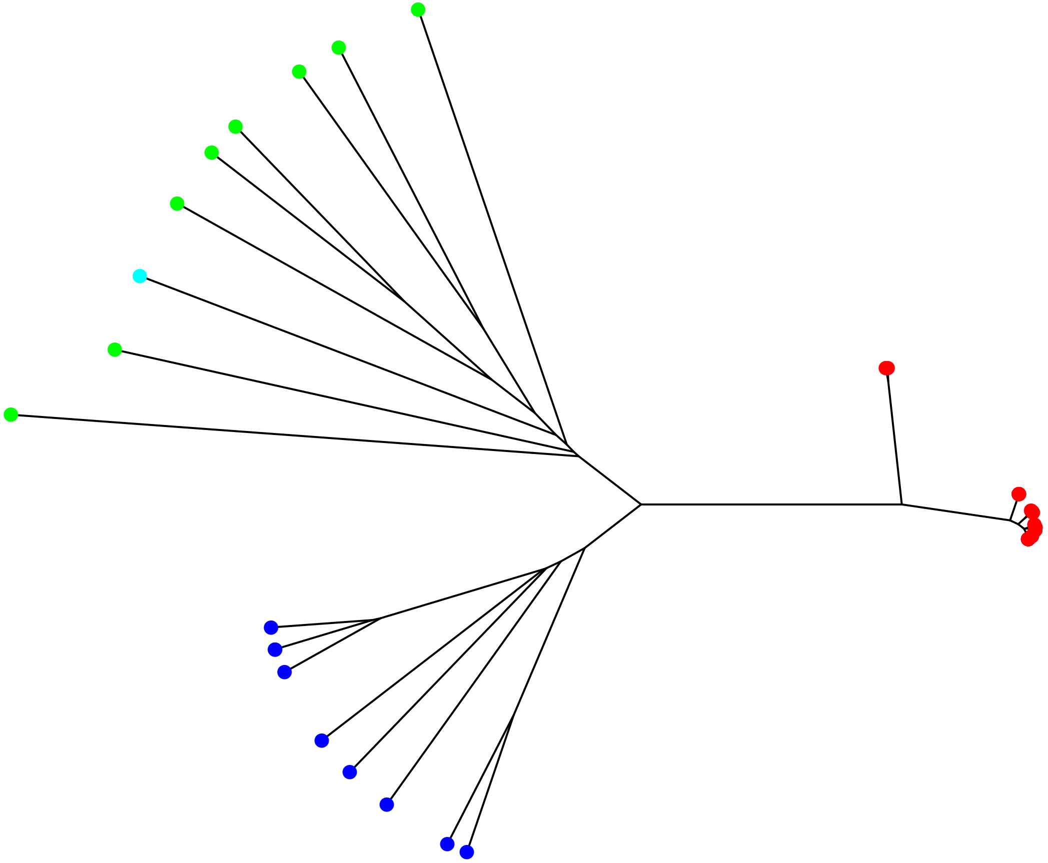

Gene family 633 : hypothetical protein

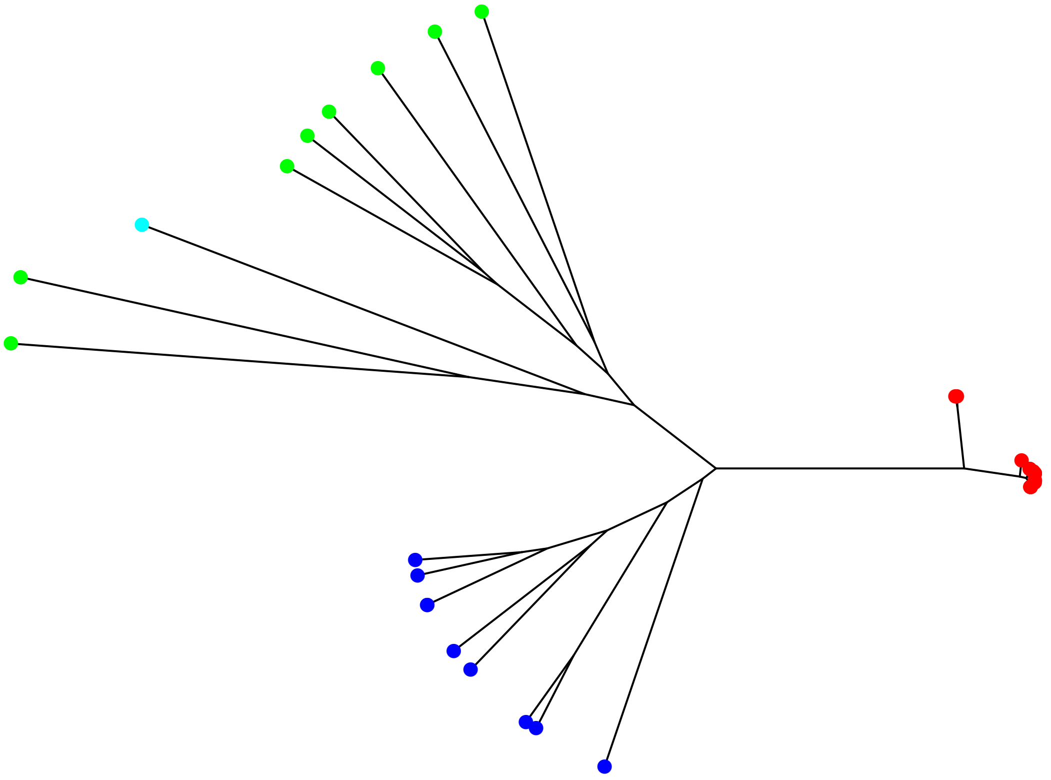

Gene family 667 : 50S ribosomal protein L33

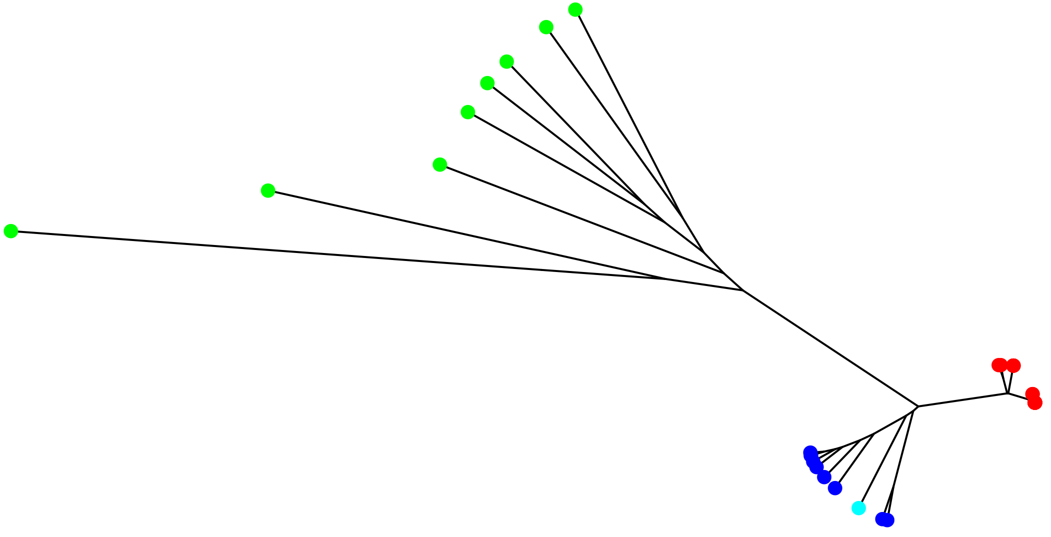

Gene family 695 : Zn−ribbon−containing, possibly RNA−binding protein and truncated derivatives

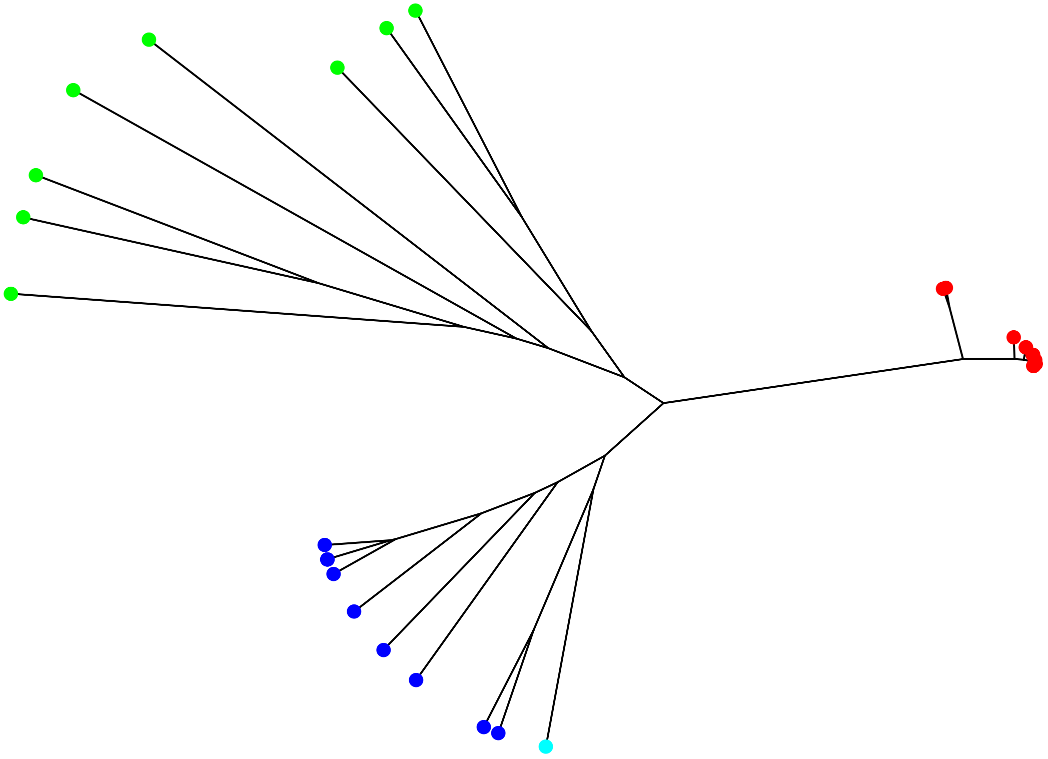

Gene family 732 : hypothetical protein

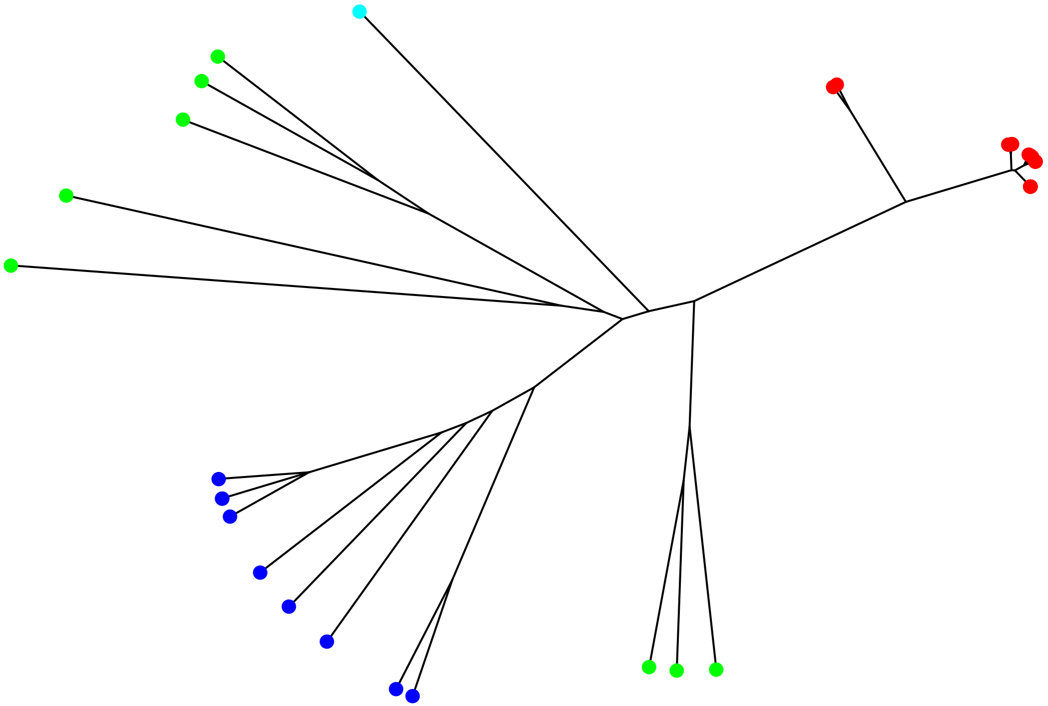

Gene family 748 : ProP effector

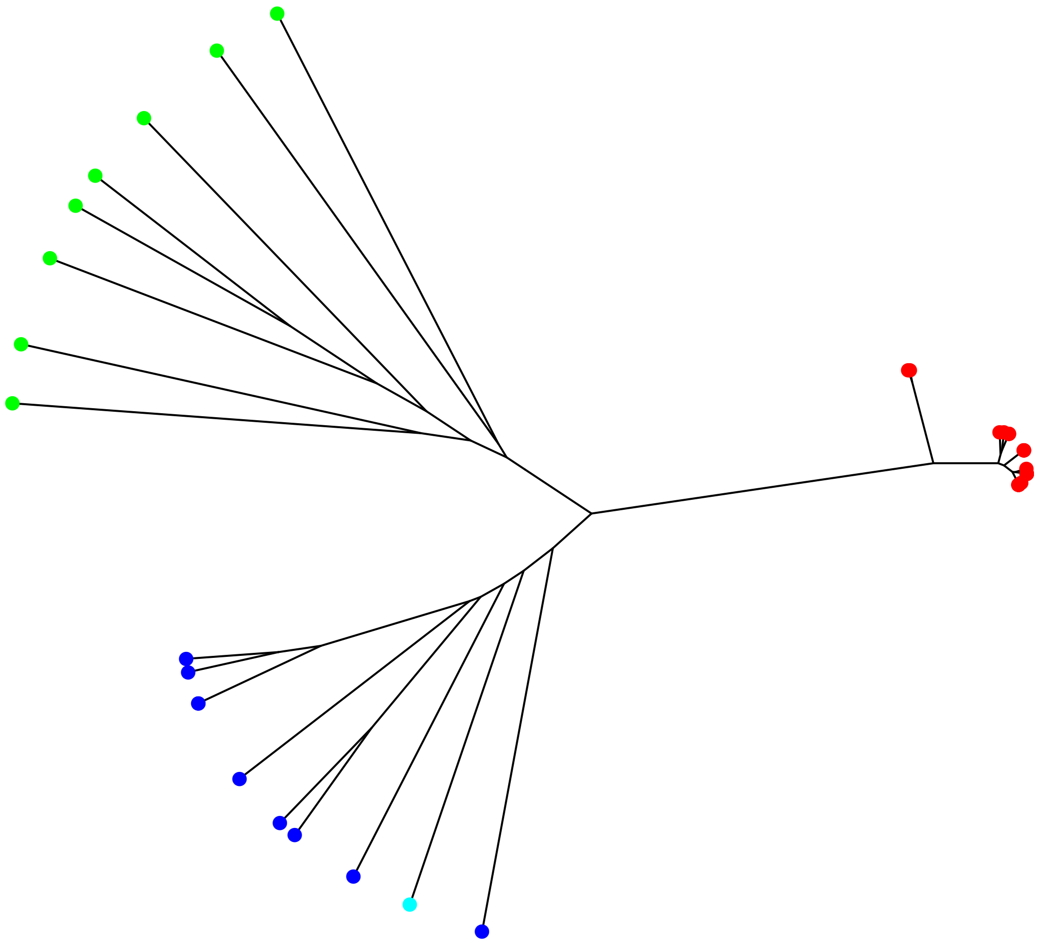

Gene family 770 : 50S ribosomal protein L19

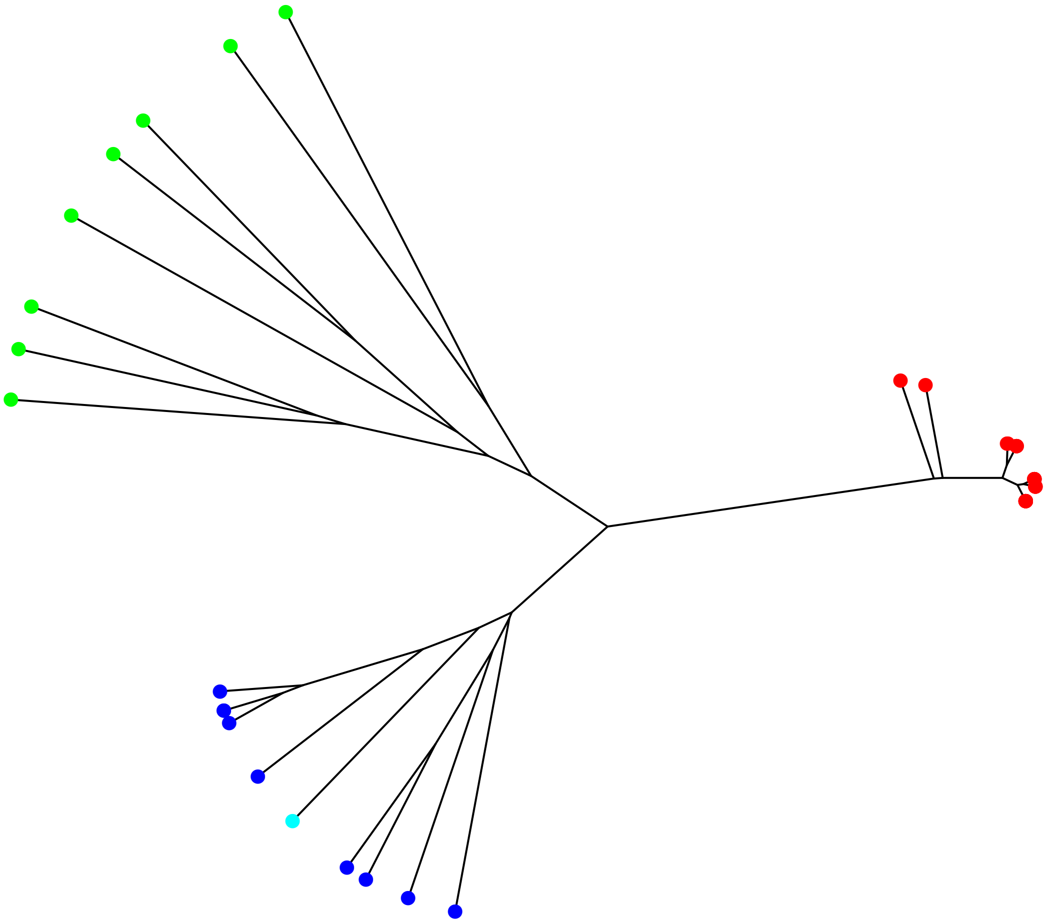

Gene family 796 : DNA−directed RNA polymerase subunit alpha

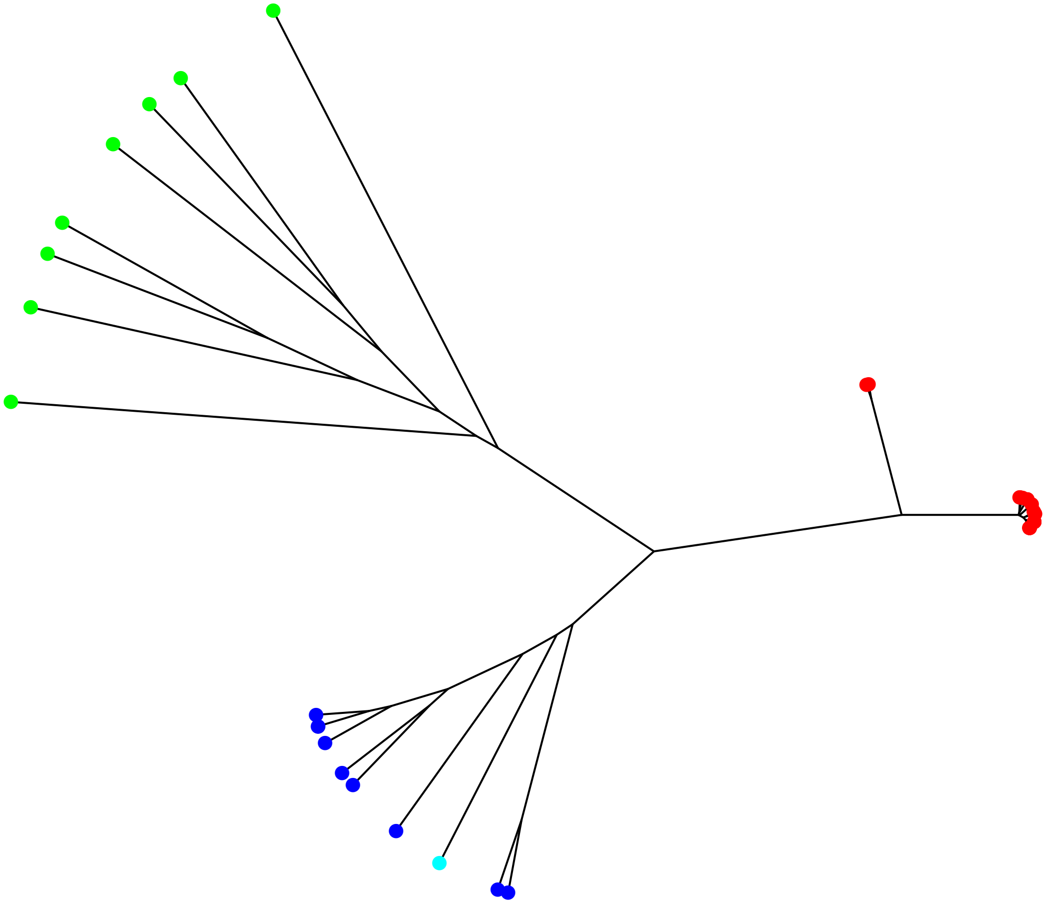

Gene family 801 : 50S ribosomal protein L15

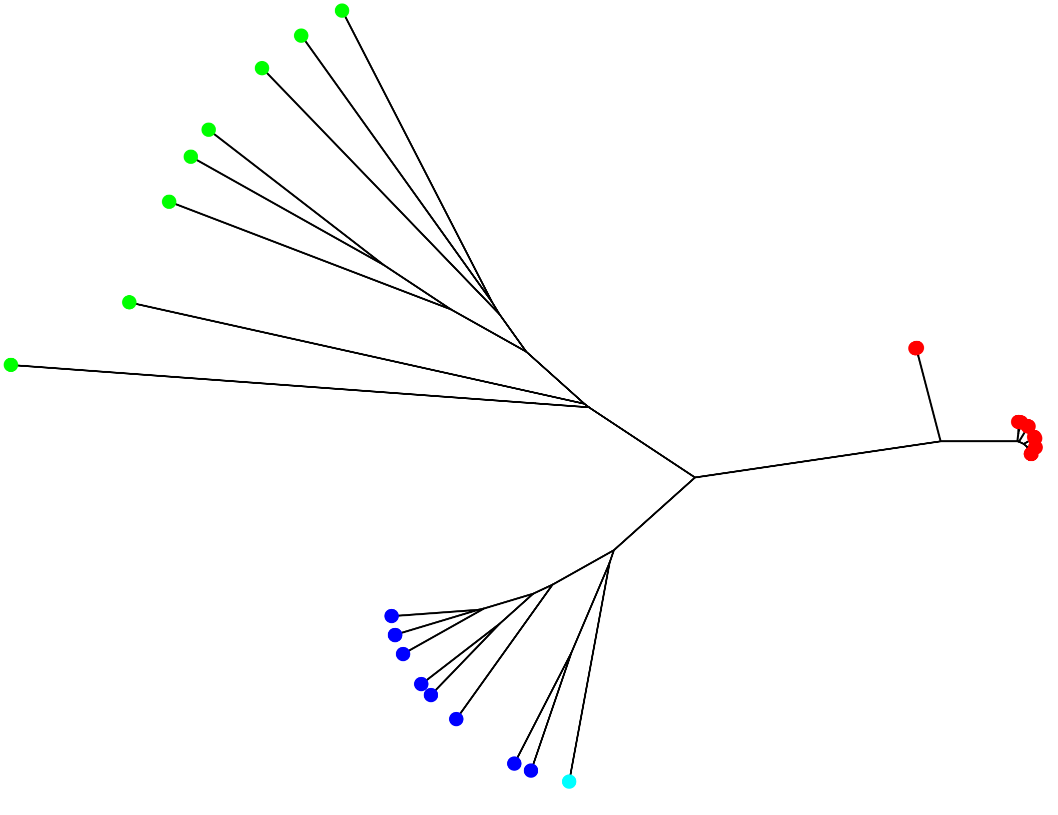

Gene family 809 : 50S ribosomal protein L14

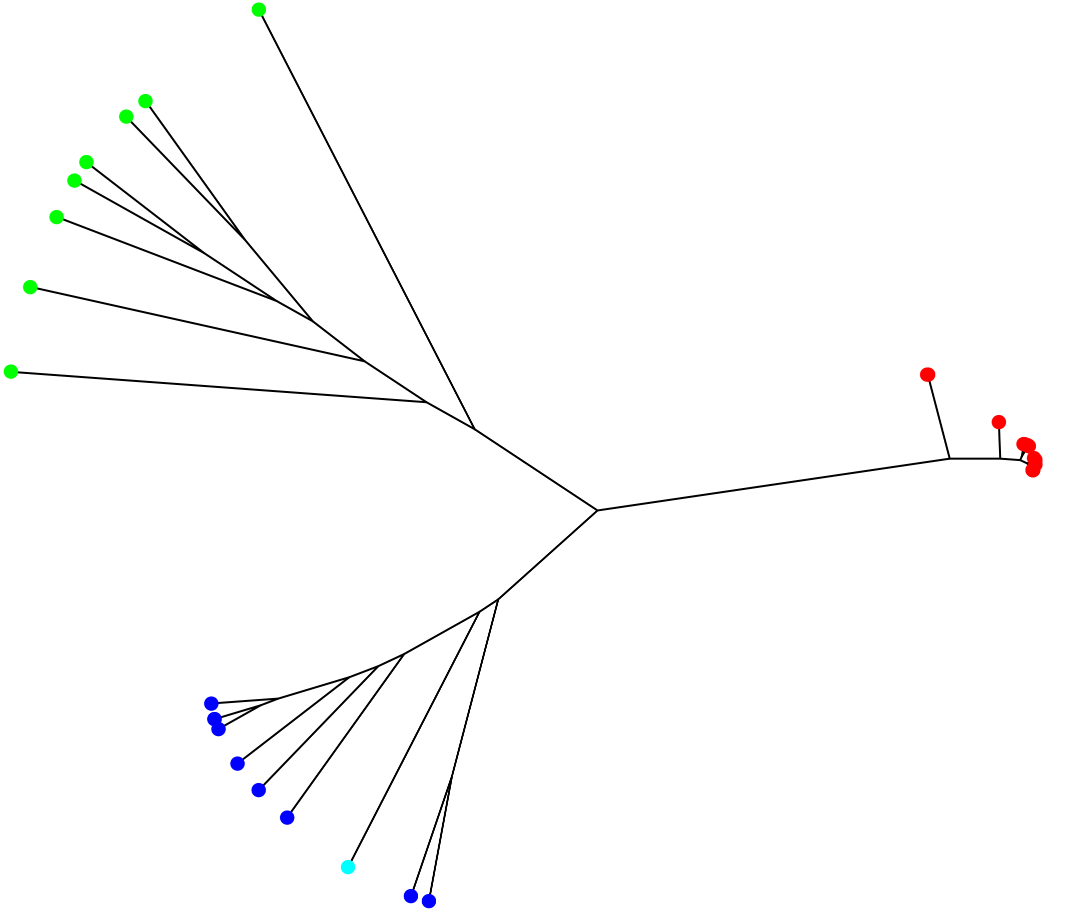

Gene family 810 : 30S ribosomal protein S17

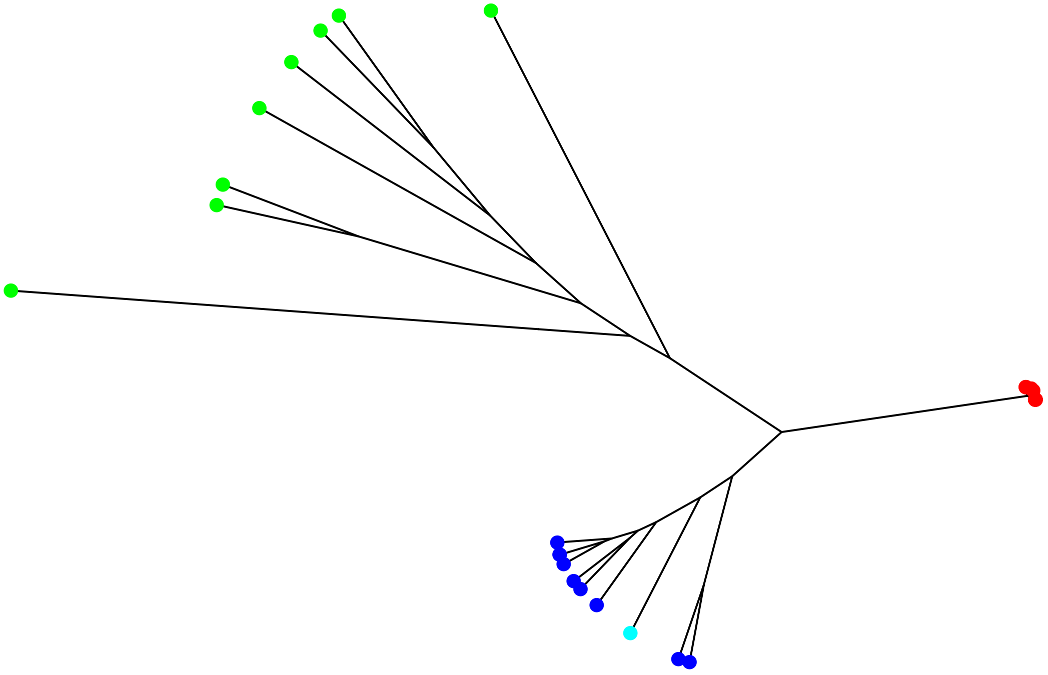

Gene family 818 : 50S ribosomal protein L3

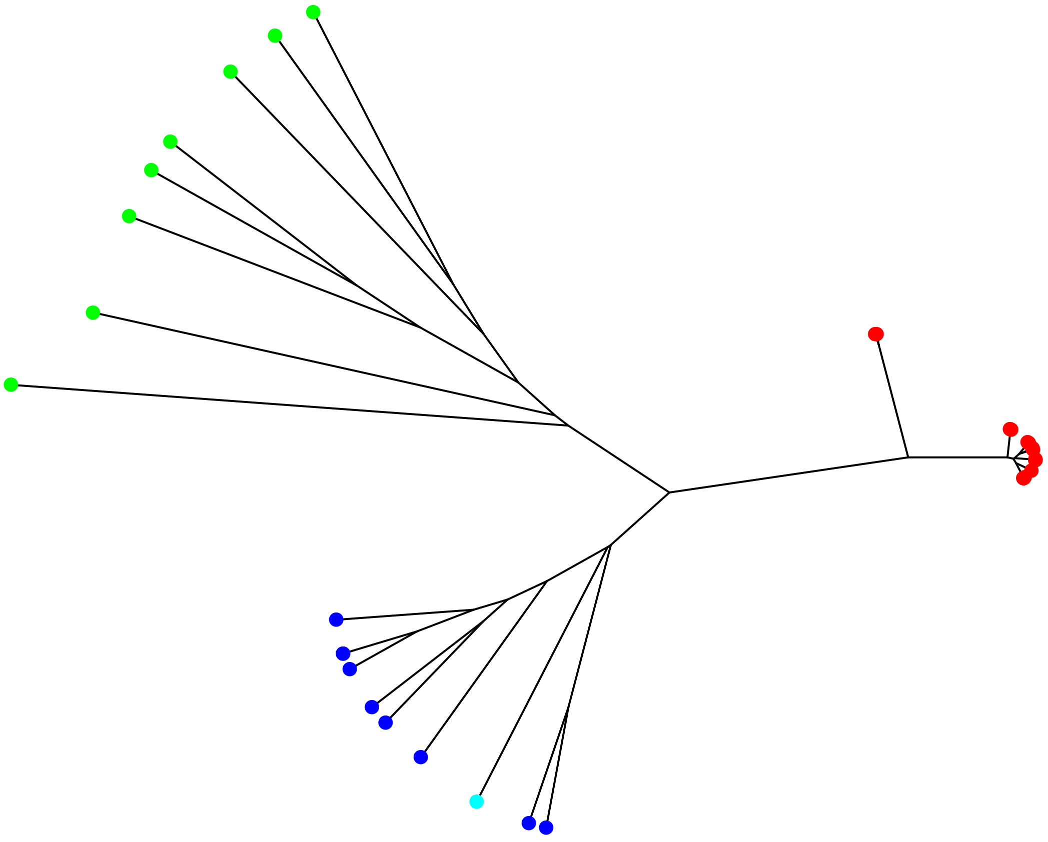

Gene family 828 : Succinyl−CoA ligase [ADP−forming] subunit alpha

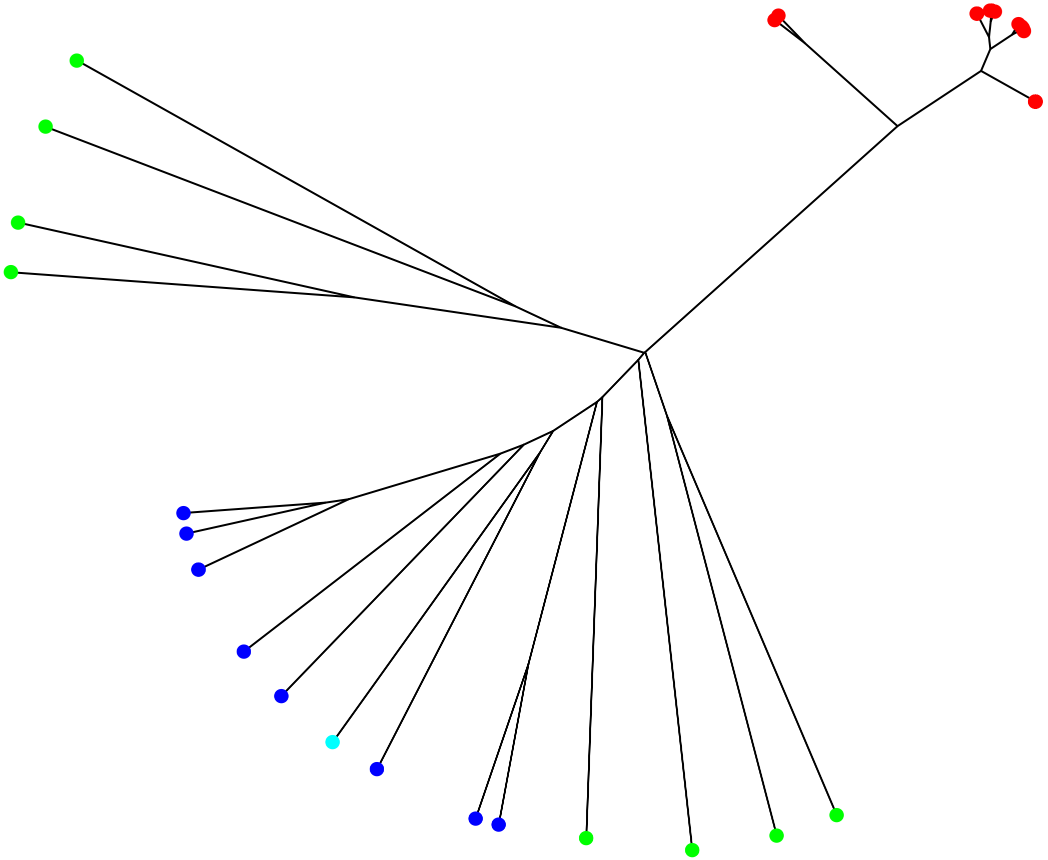

Gene family 835 : FeoC like transcriptional regulator

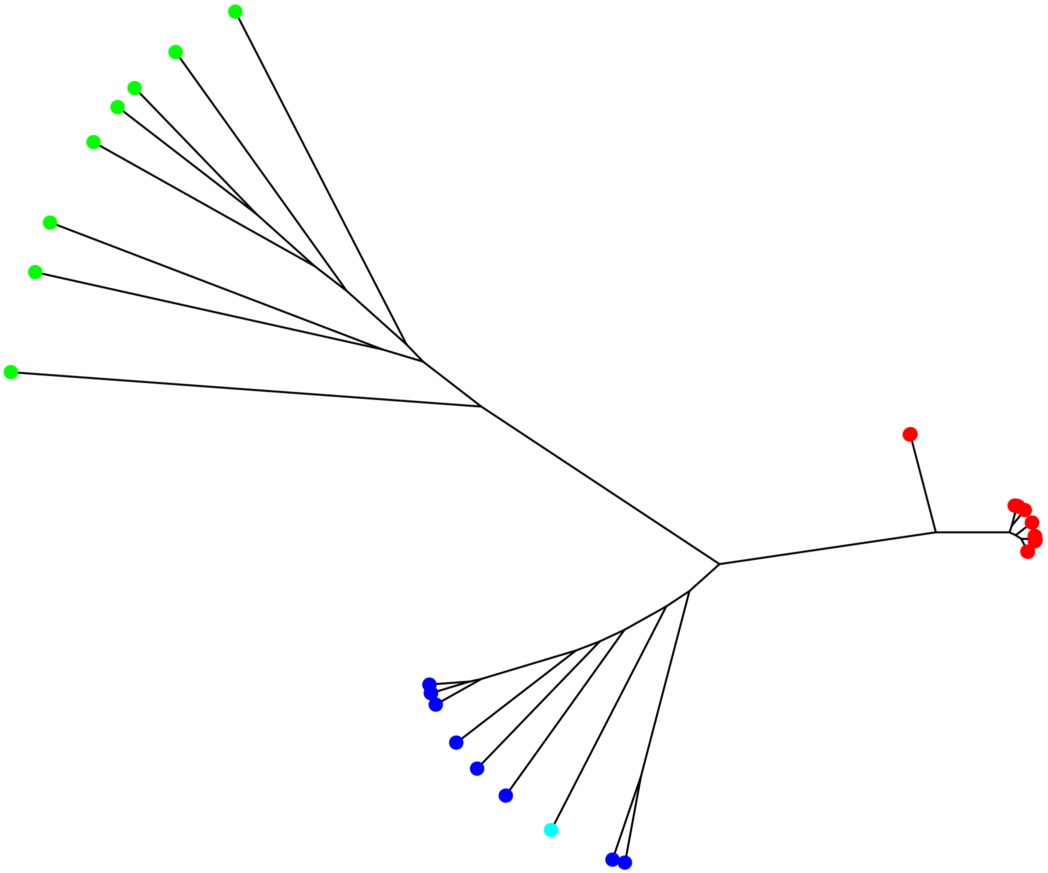

Gene family 850 : hypothetical protein

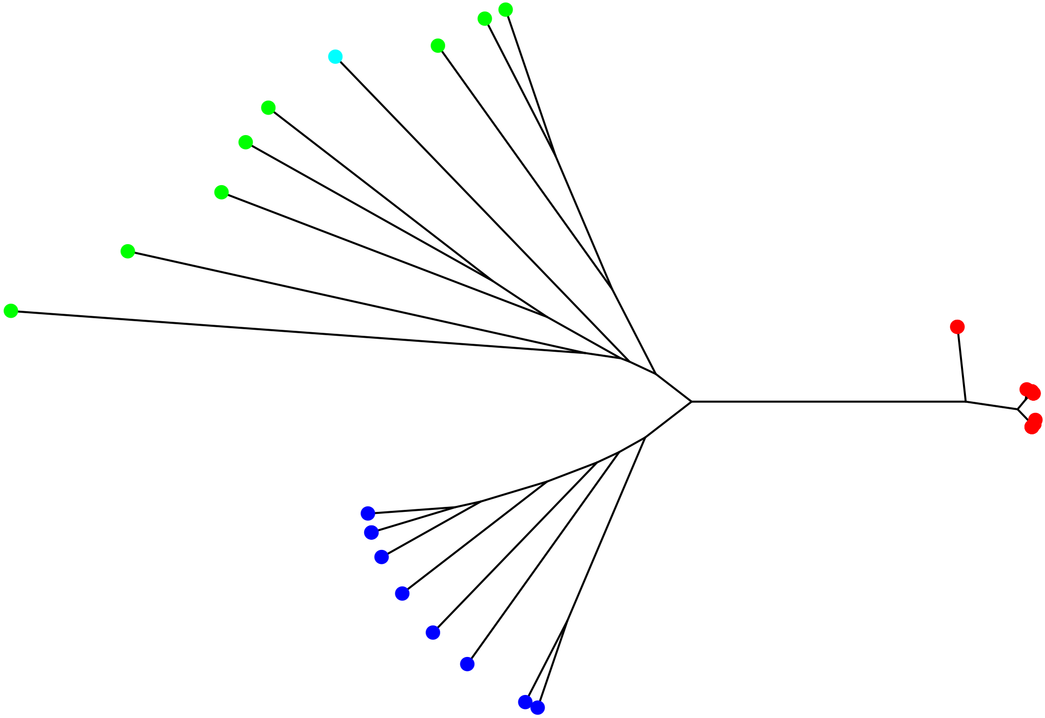

Gene family 853 : Phagosome trafficking protein DotA

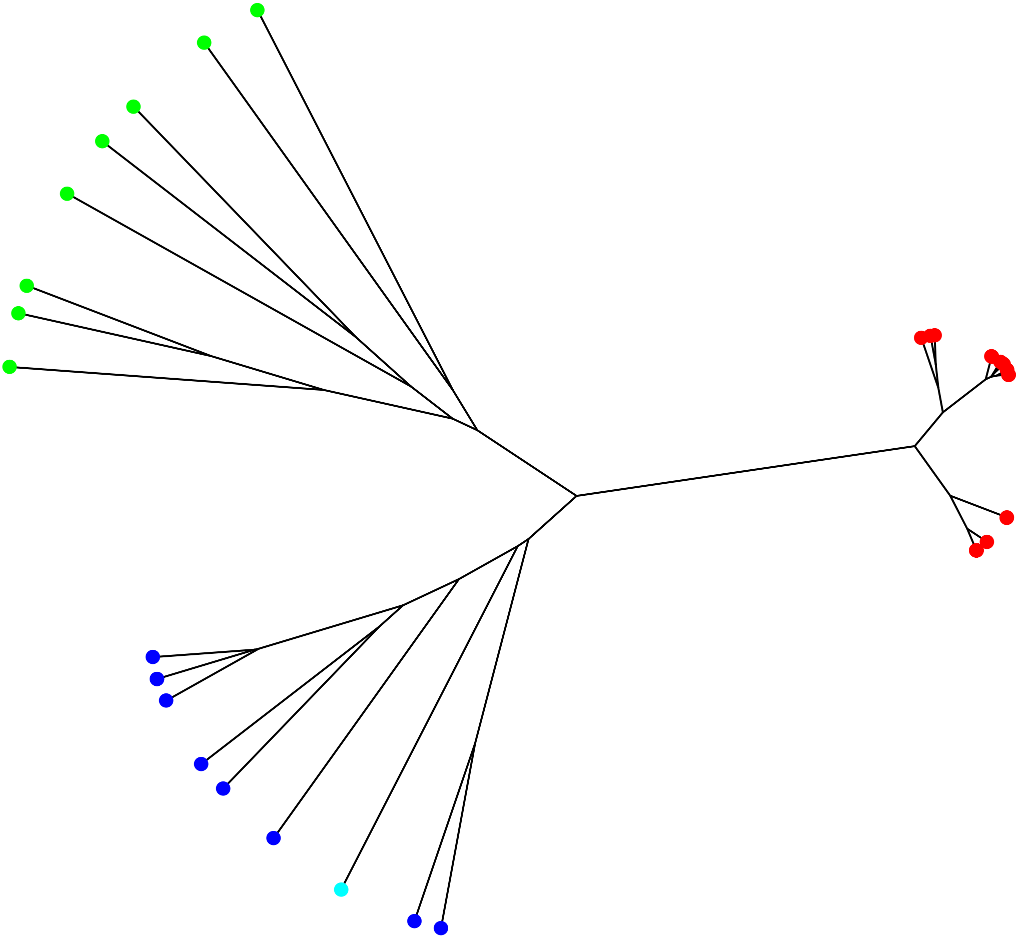

Gene family 875 : Translation initiation factor IF−3

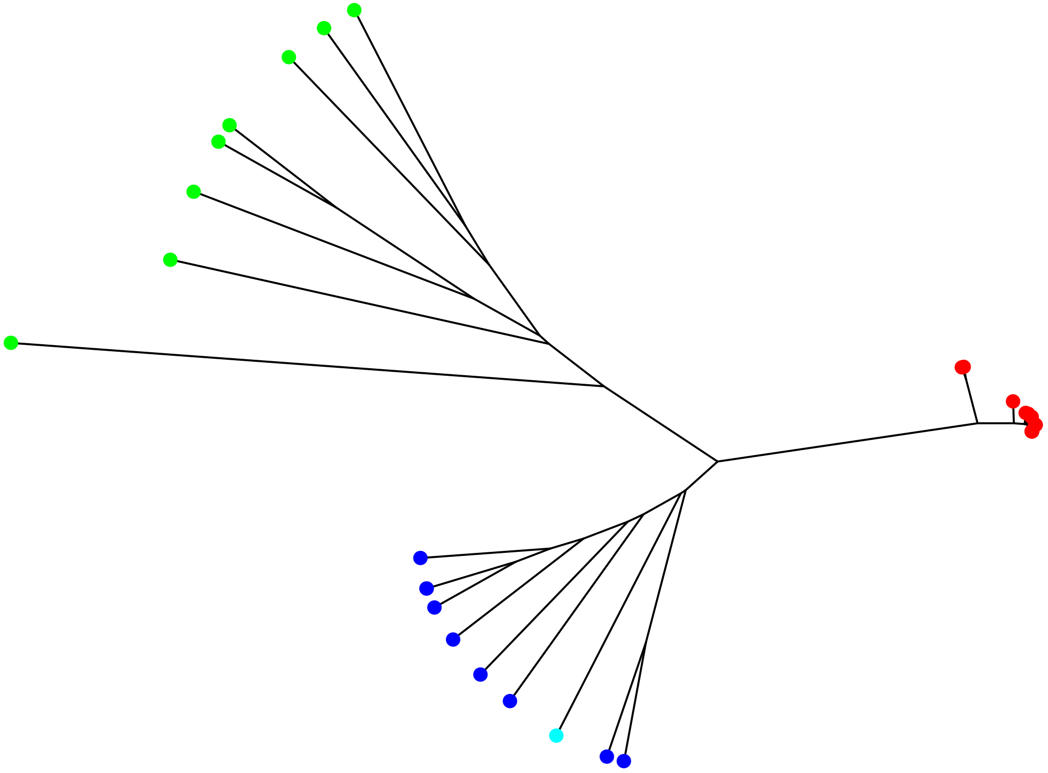

Gene family 908 : NADH−quinone oxidoreductase subunit I

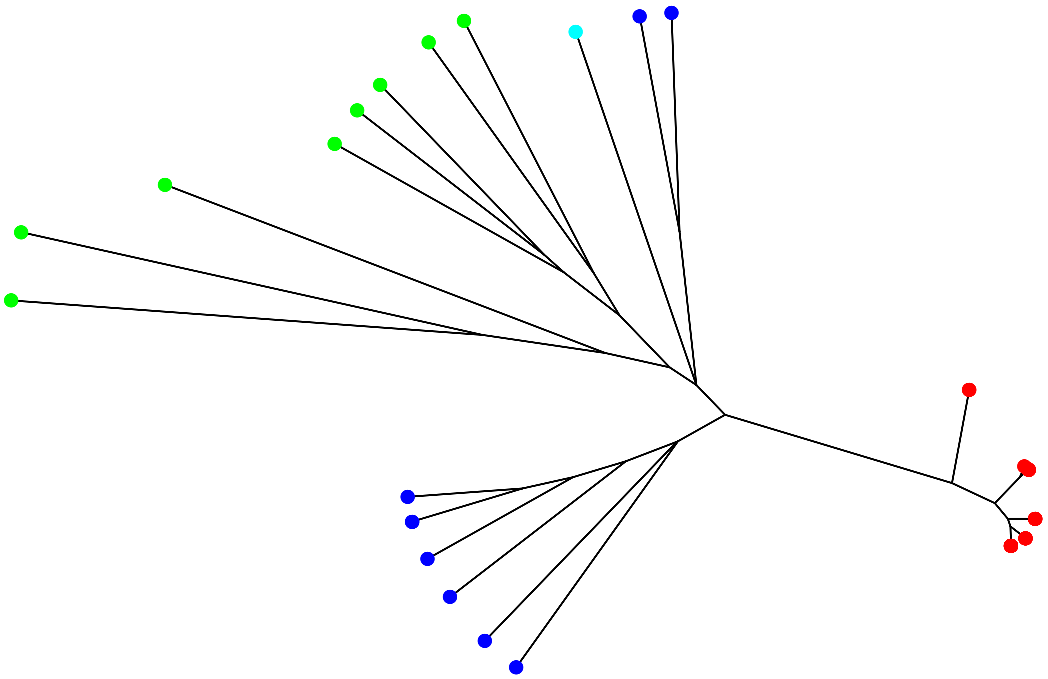

Gene family 911 : NADH−quinone oxidoreductase subunit F

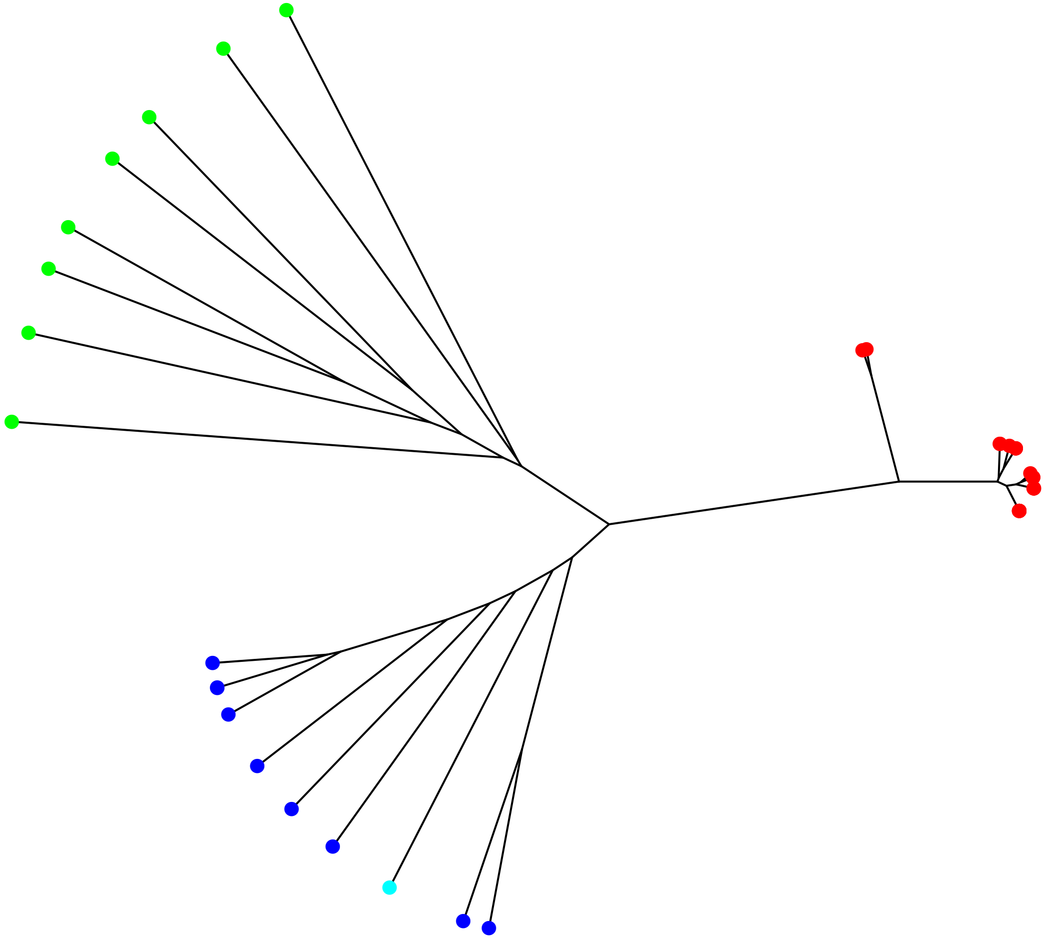

Gene family 915 : NAD(P)H−quinone oxidoreductase subunit 3

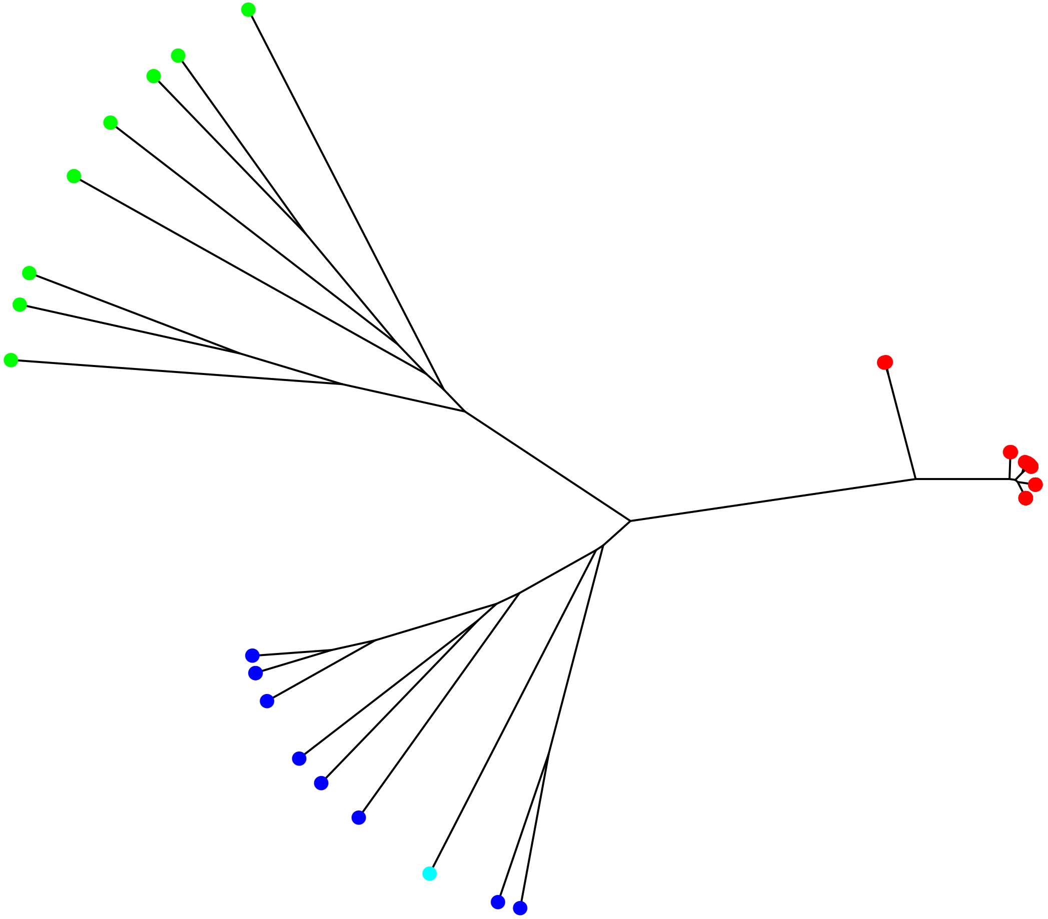

Gene family 930 : hypothetical protein

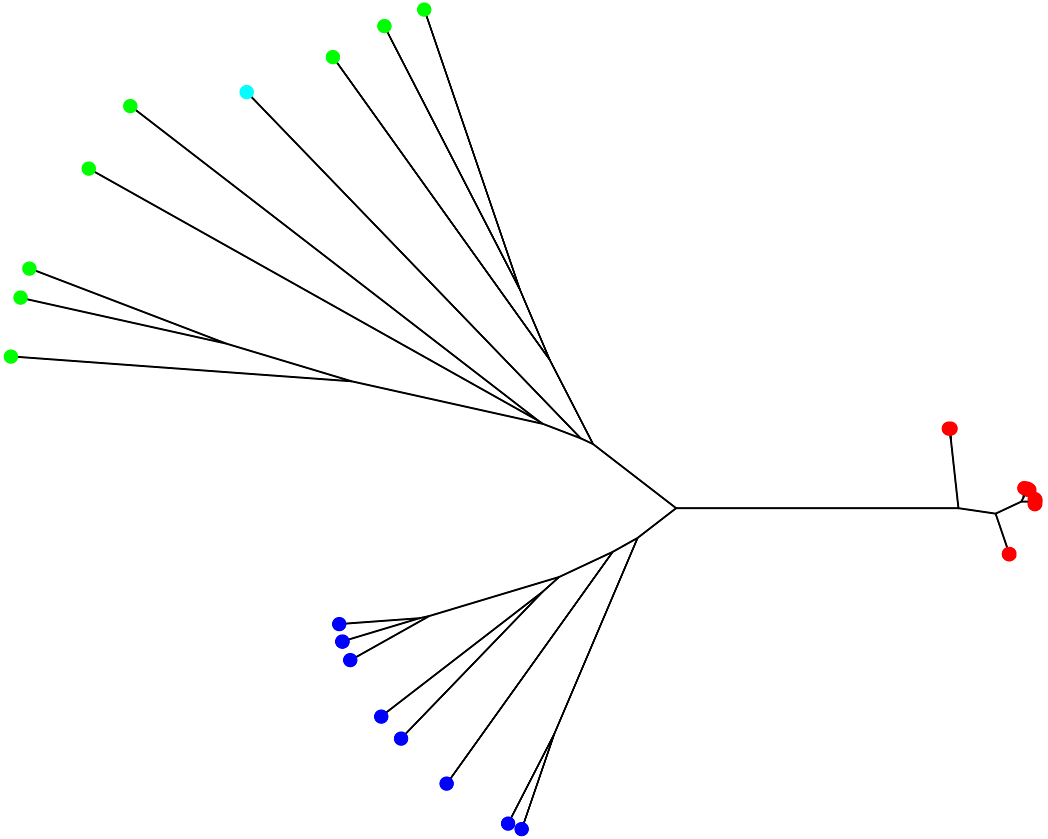

Gene family 966 : branched−chain alpha−keto acid dehydrogenase subunit E2

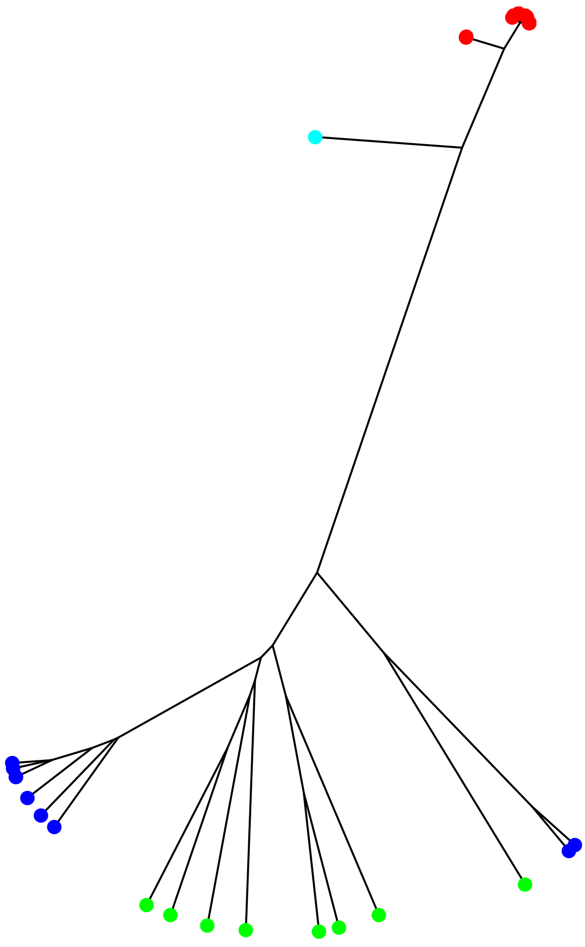

Gene family 1011 : ethanolamine utilization protein EutJ

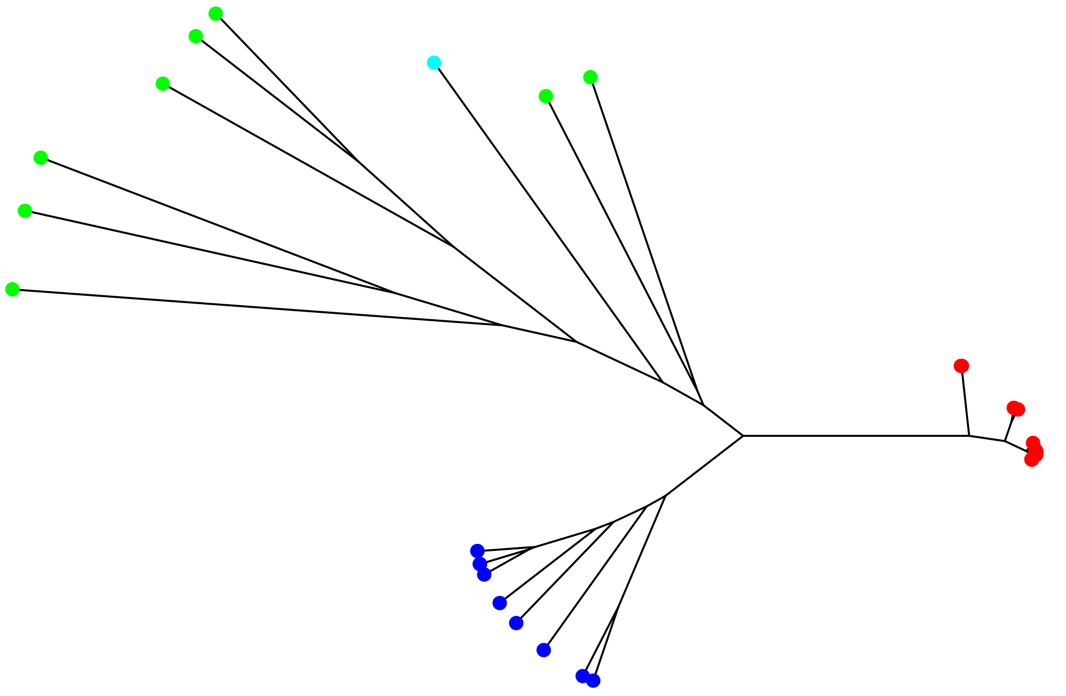

Gene family 1012 : Pilus assembly protein, PilO

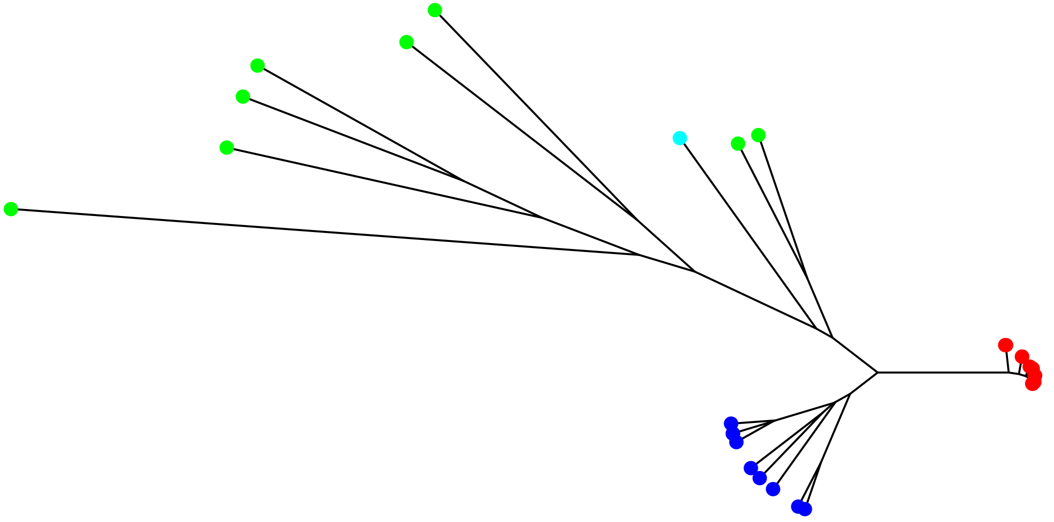

Gene family 1013 : Pilus assembly protein, PilP

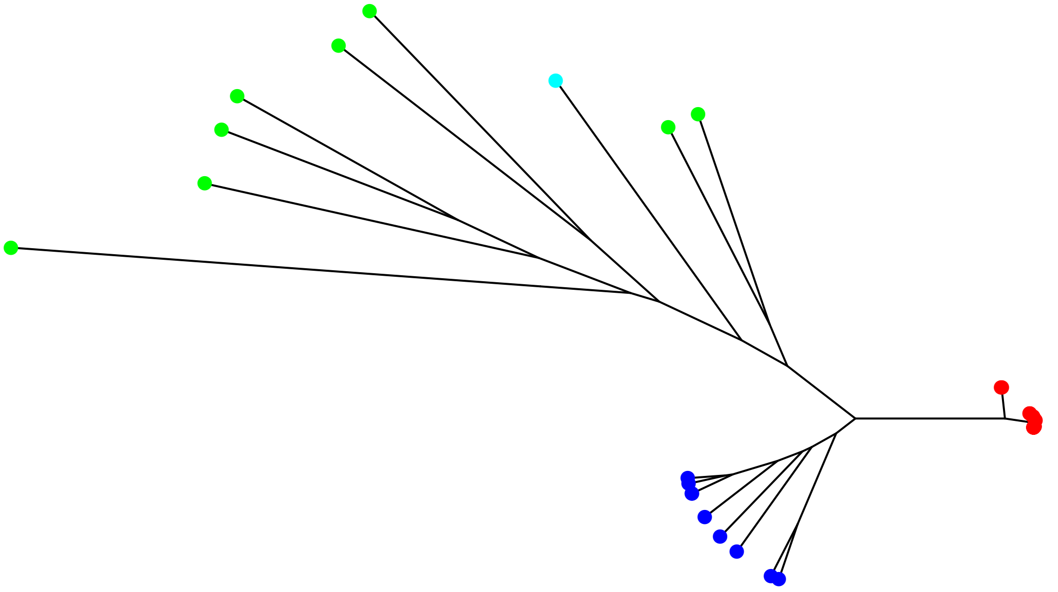

Gene family 1014 : Type IV pilus biogenesis and competence protein PilQ precursor

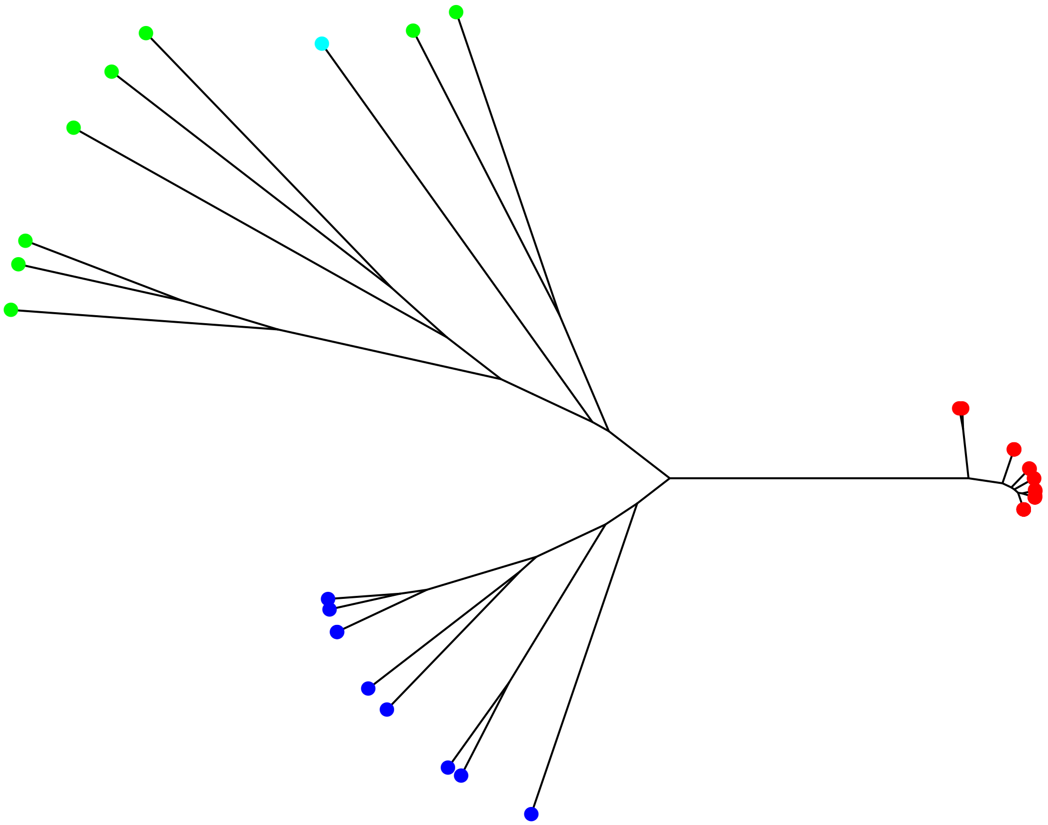

Gene family 1026 : pyridoxal phosphate enzyme, YggS family

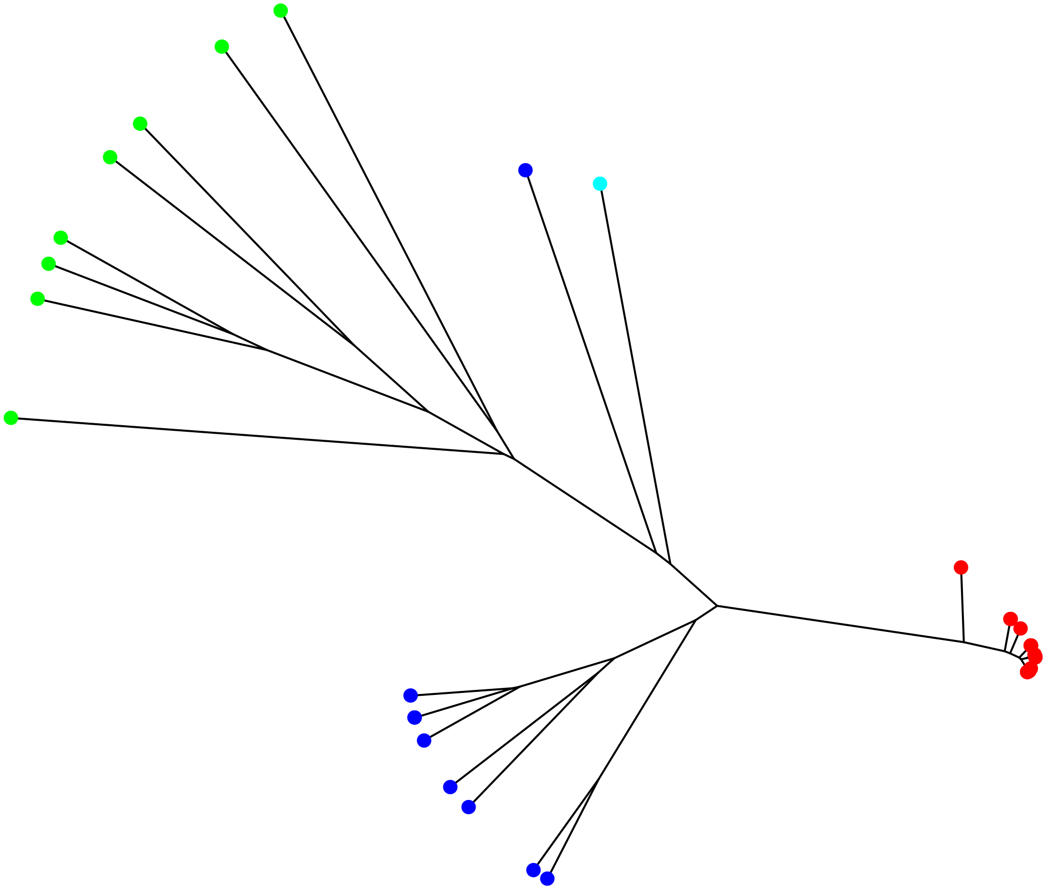

Gene family 1059 : lysozyme

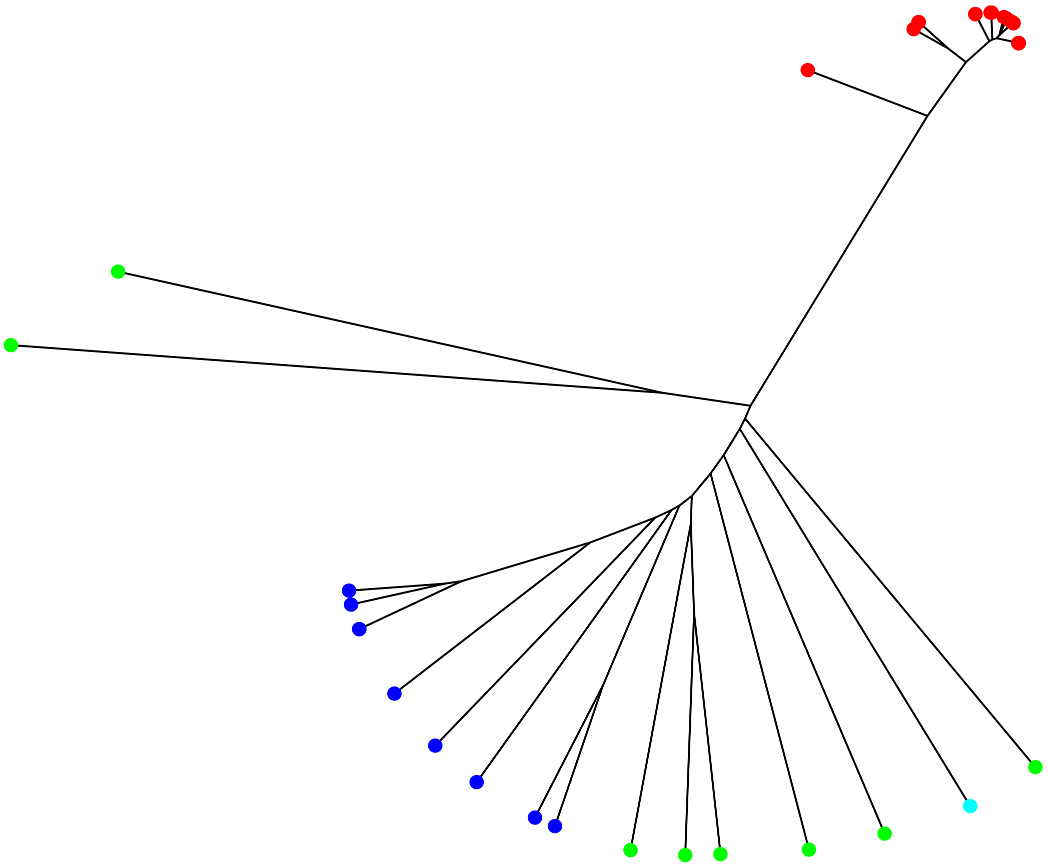

Gene family 1060 : hypothetical protein

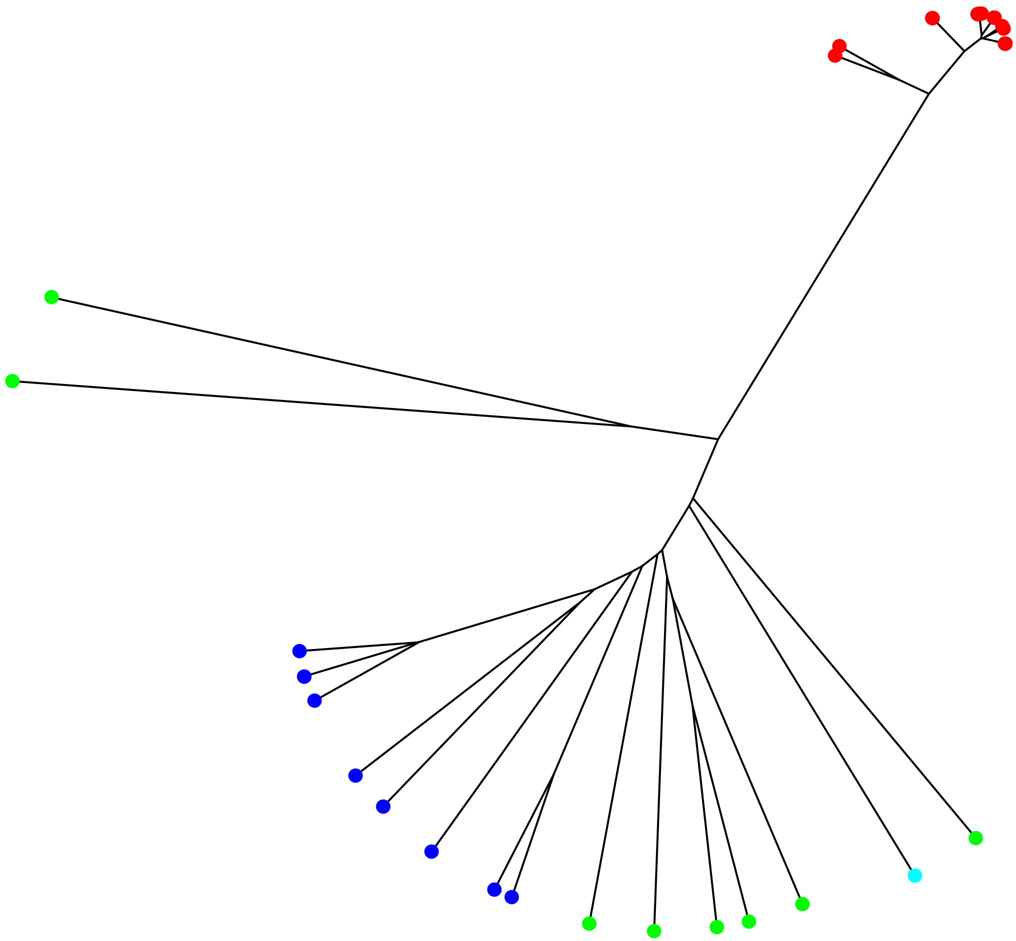

Gene family 1062 : hypothetical protein

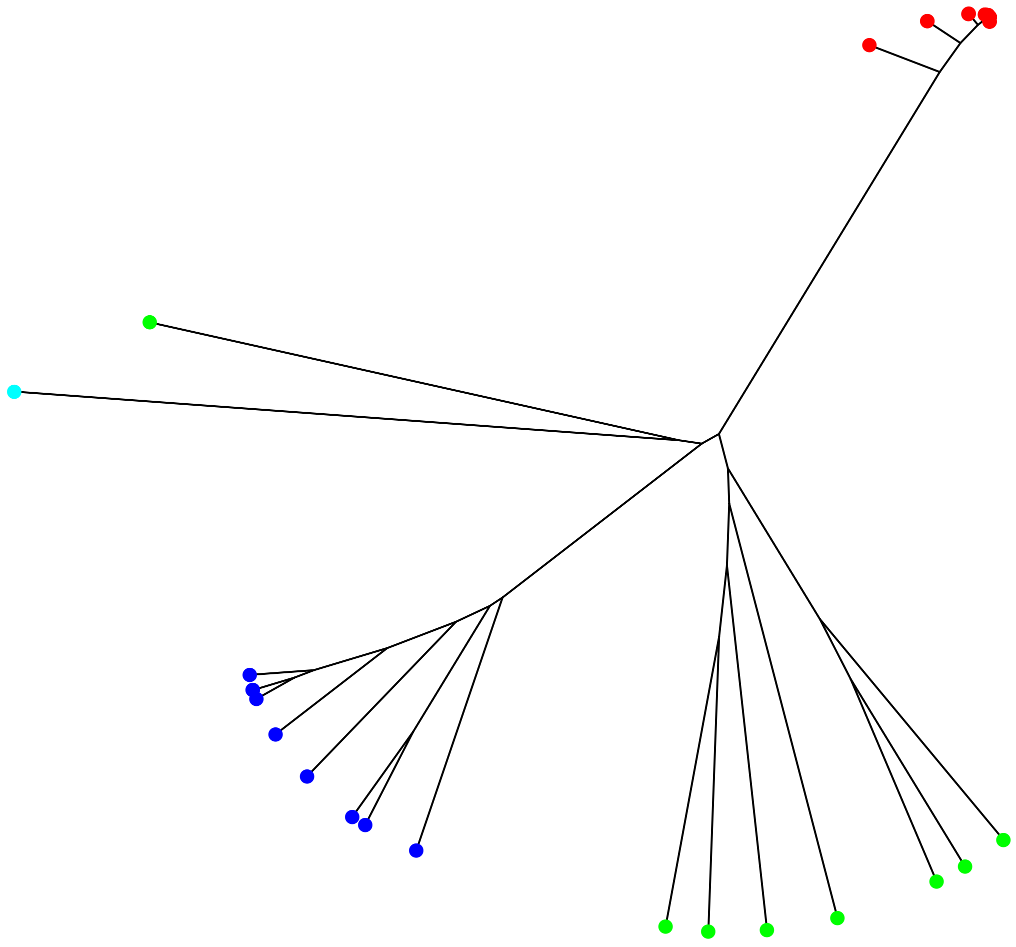

Gene family 1066 : hypothetical protein

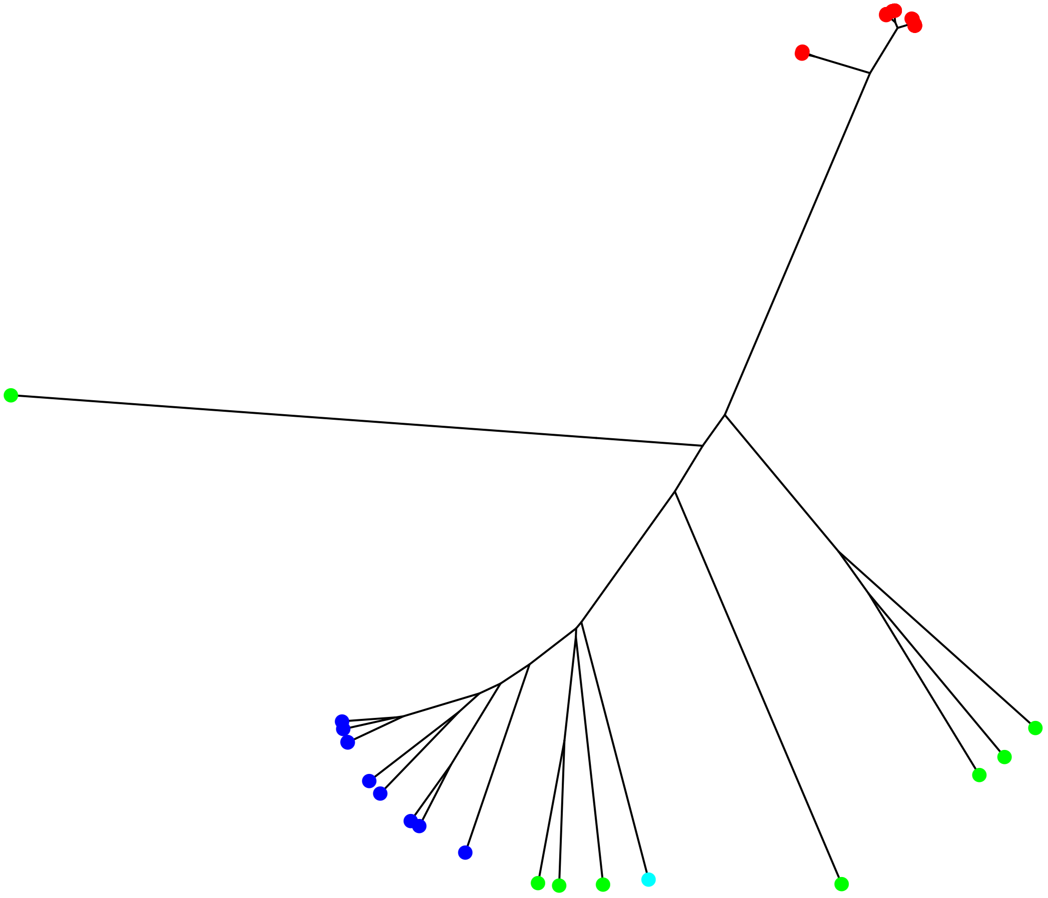

Gene family 1070 : Glutaminase

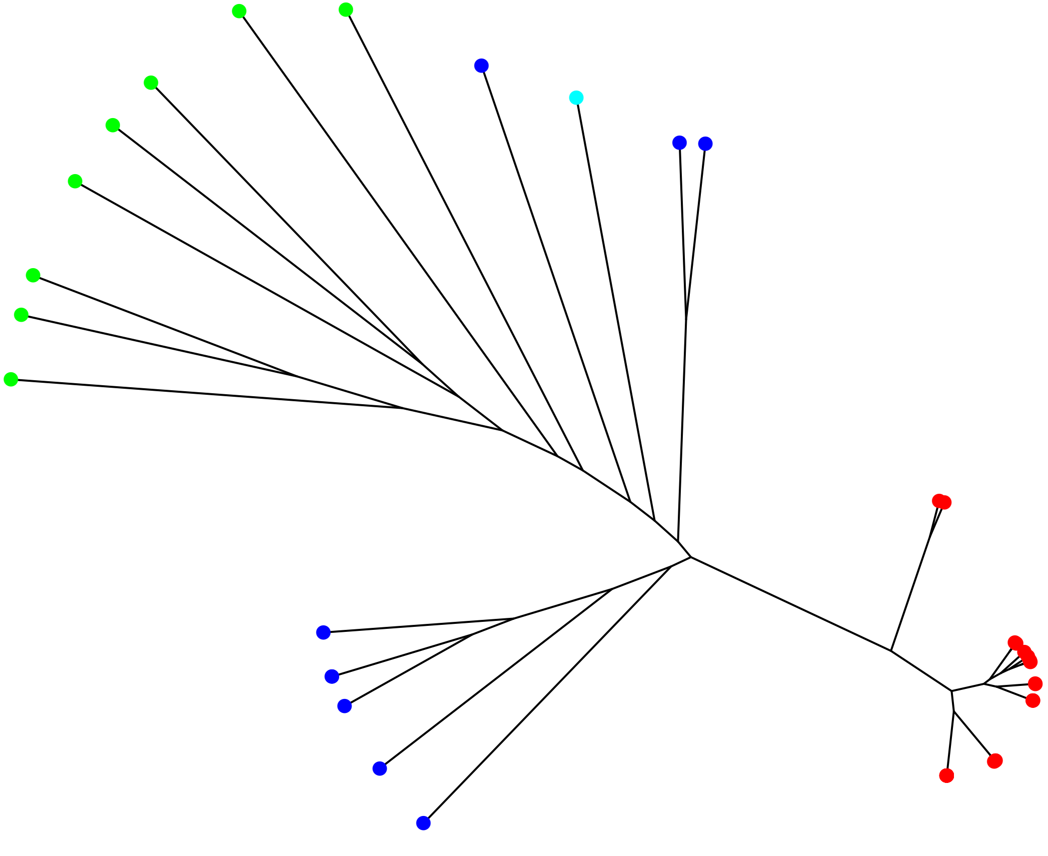

Gene family 1071 : Phenolic acid decarboxylase subunit B

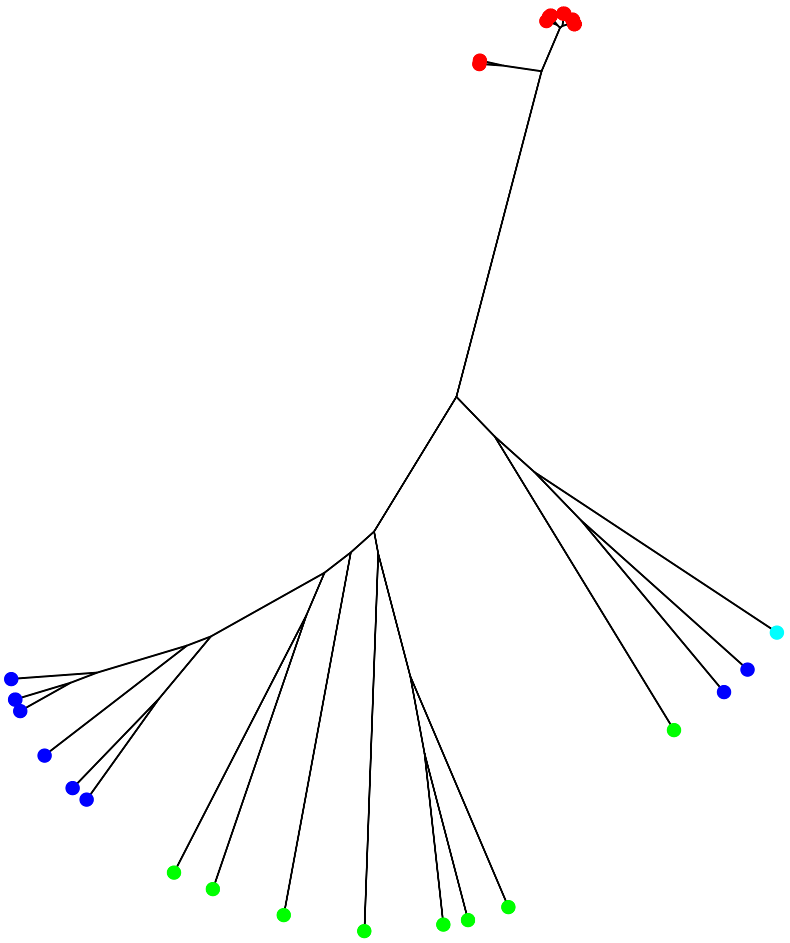

Gene family 1087 : hypothetical protein

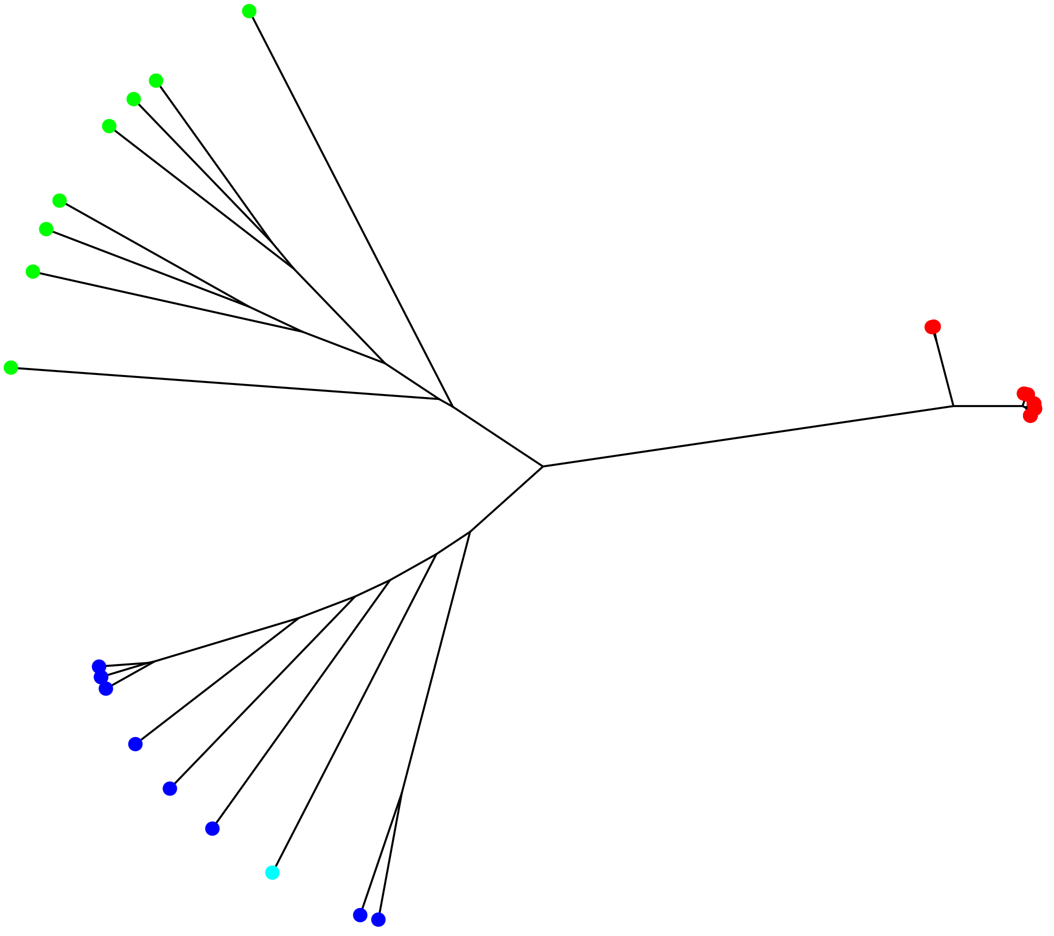

Gene family 1104 : hypothetical protein

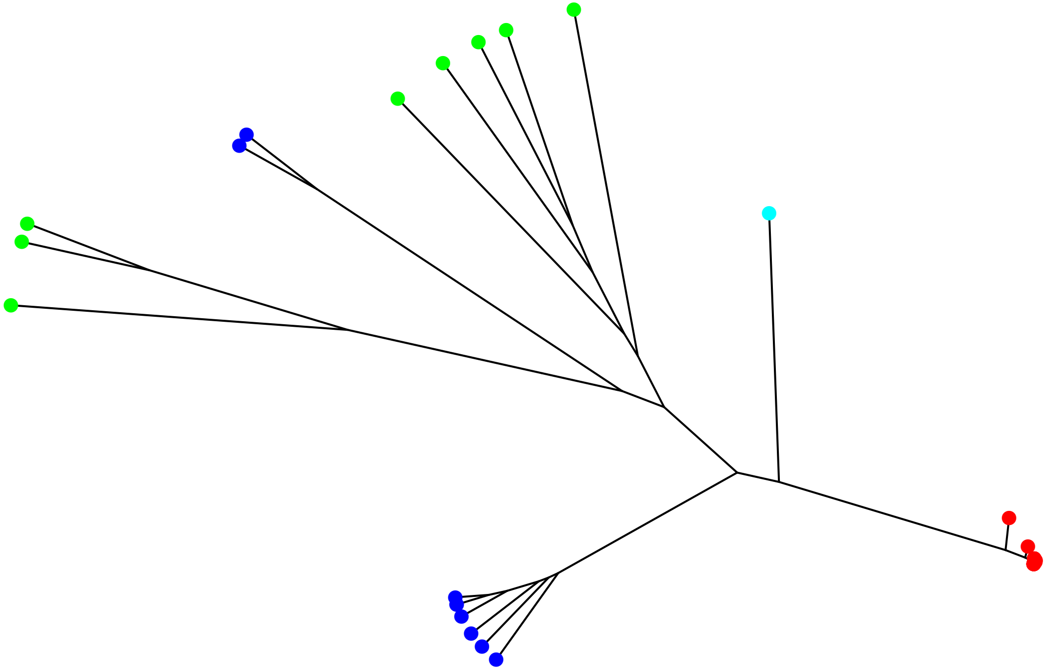

Gene family 1107 : Riboflavin synthase

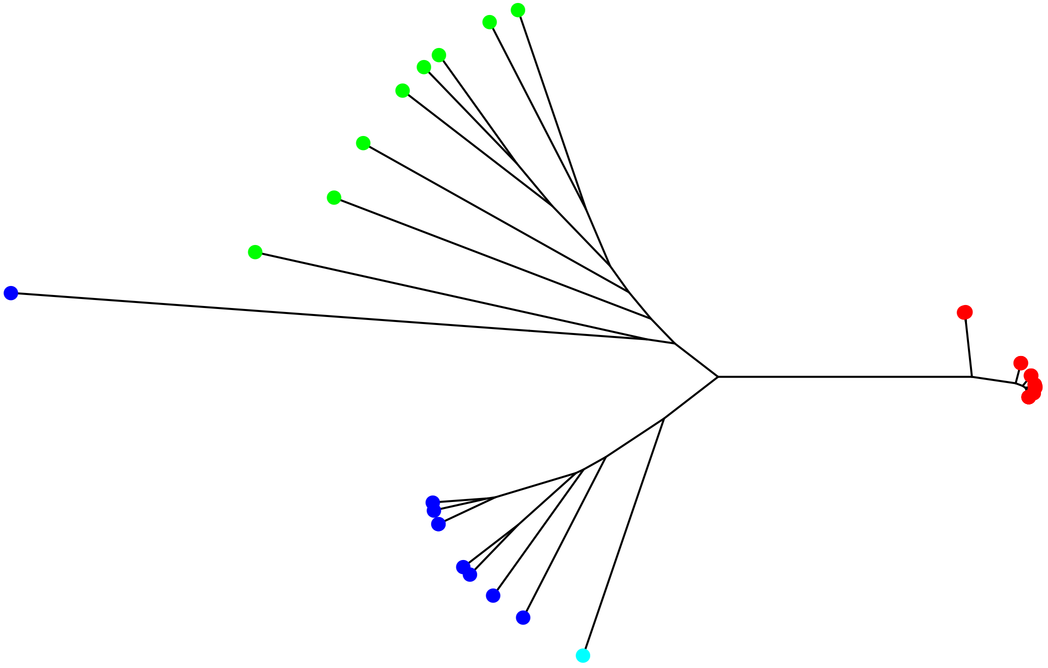

Gene family 1129 : queuosine biosynthesis protein QueD

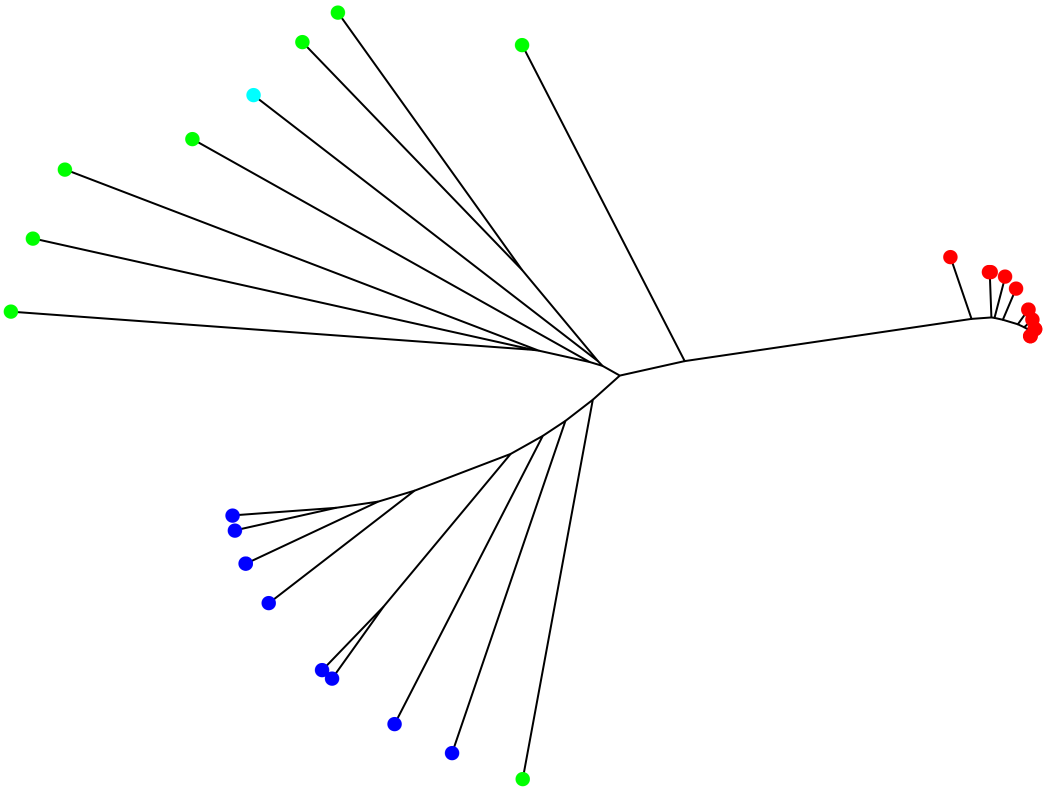

Gene family 1140 : Peptide chain release factor 1

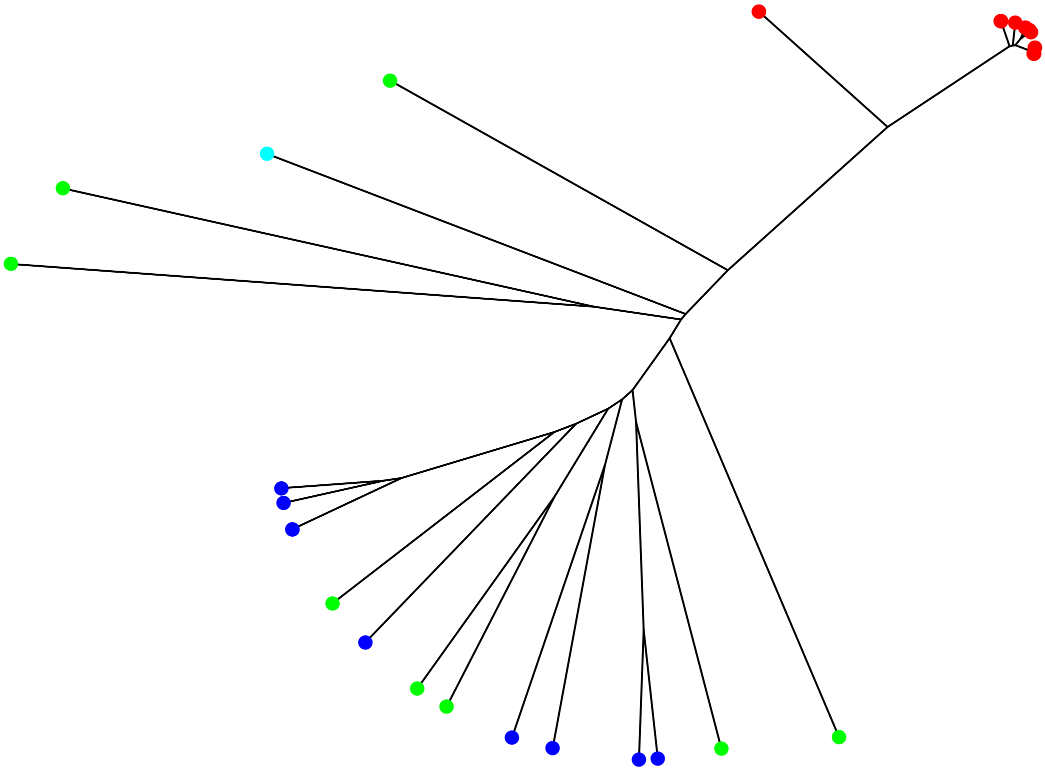

Gene family 1234 : Ribosomal RNA large subunit methyltransferase H

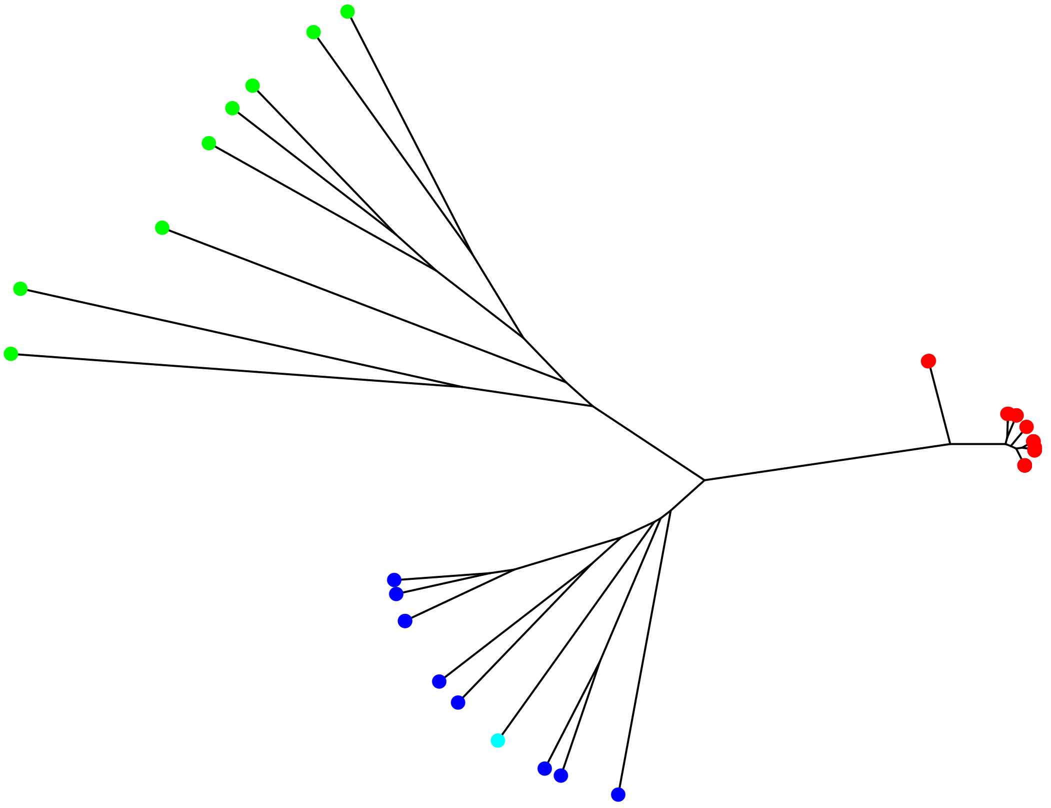

Gene family 1237 : Ribonuclease HII

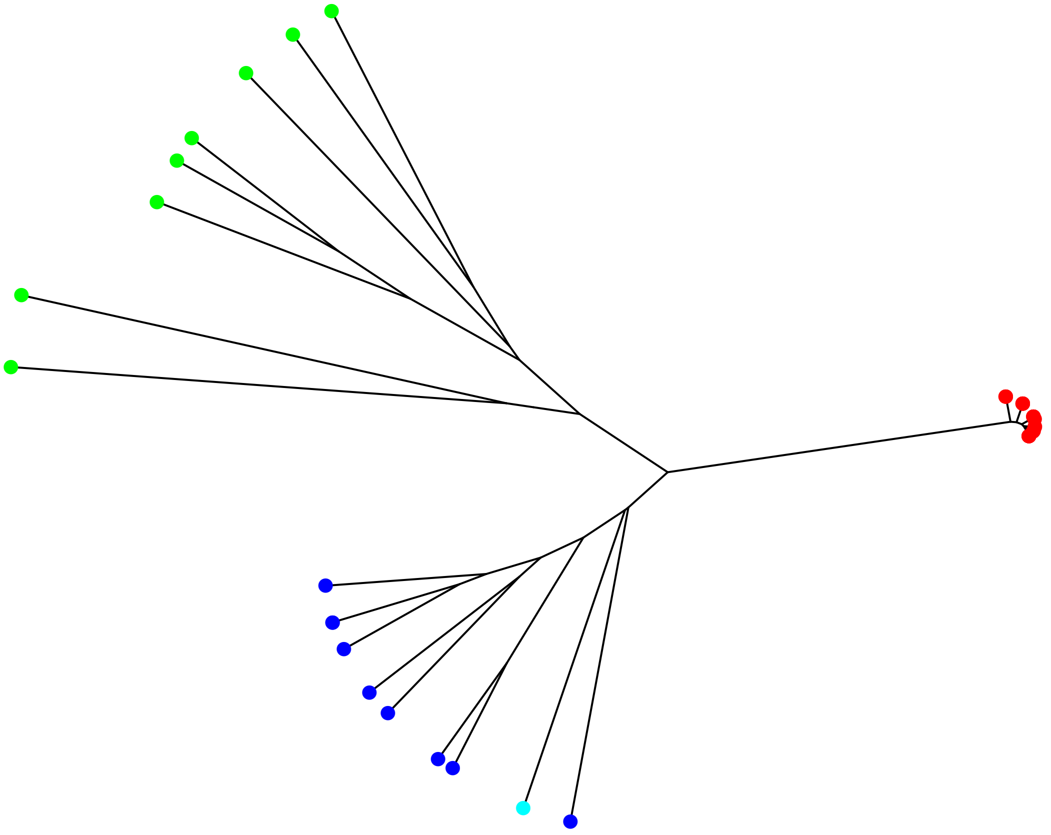

Gene family 1268 : Response regulator UvrY

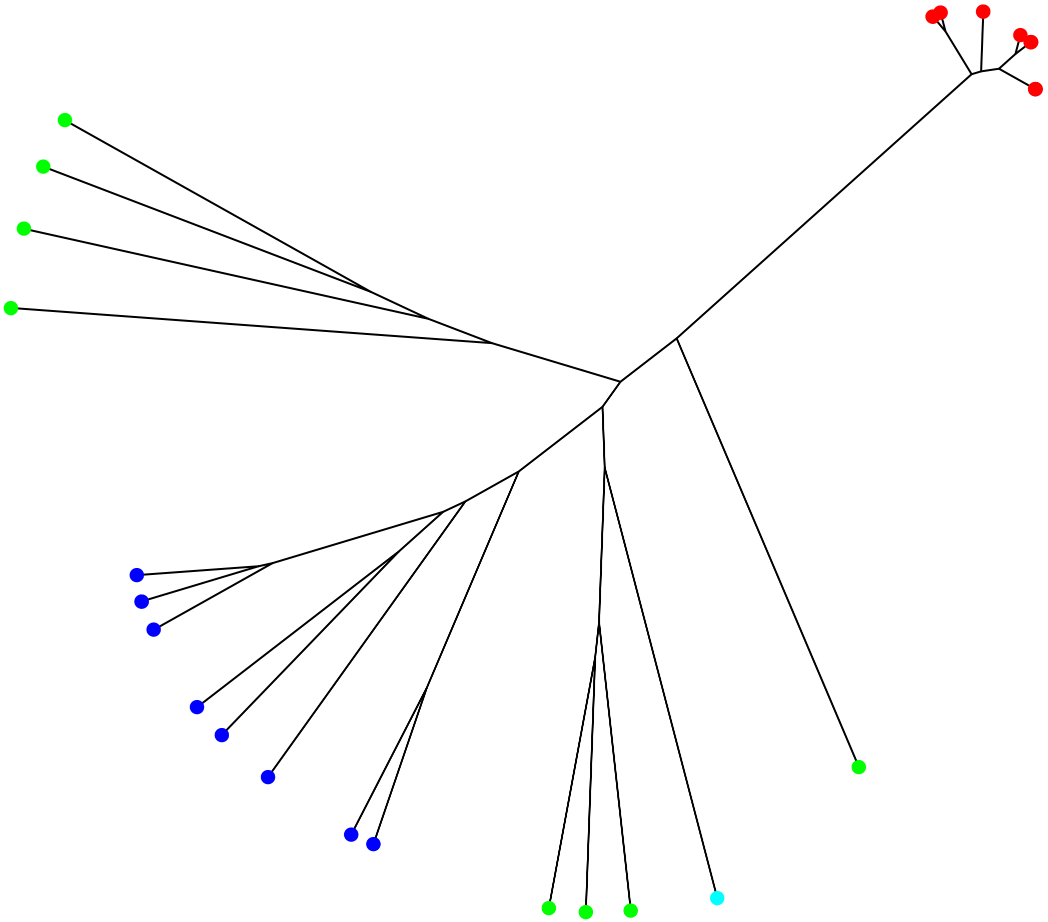

Gene family 1292 : polyhydroxyalkanoate synthesis repressor PhaR

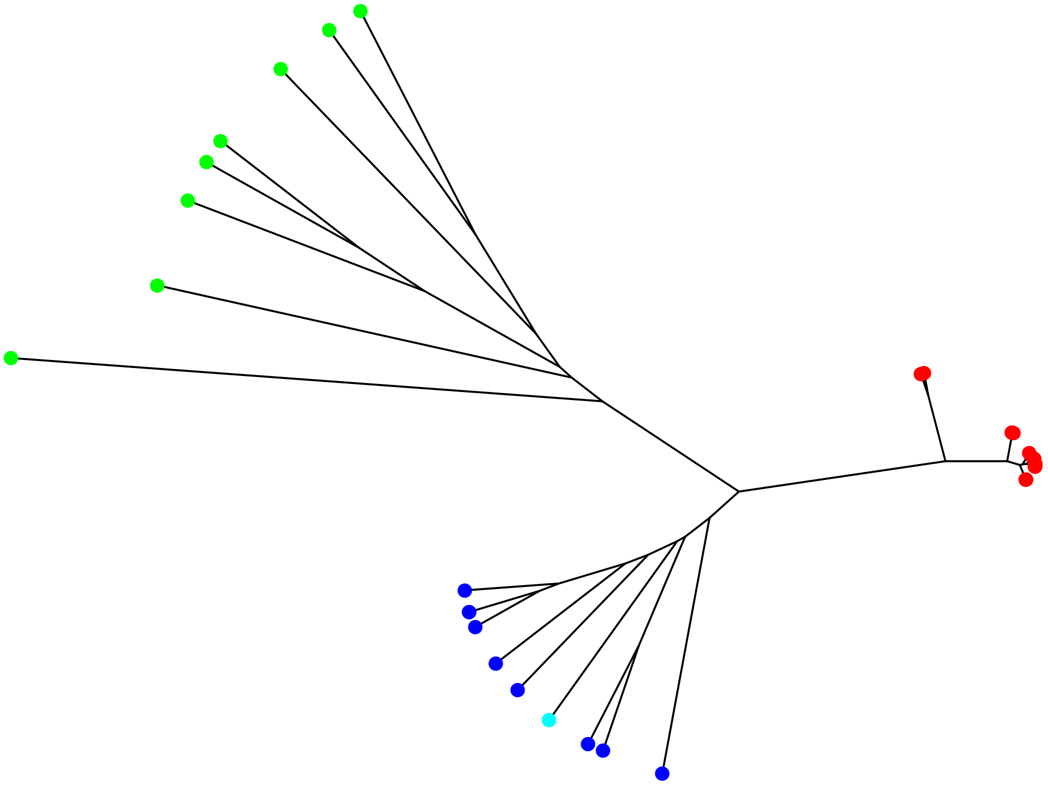

Gene family 1326 : Basal−body rod modification protein FlgD

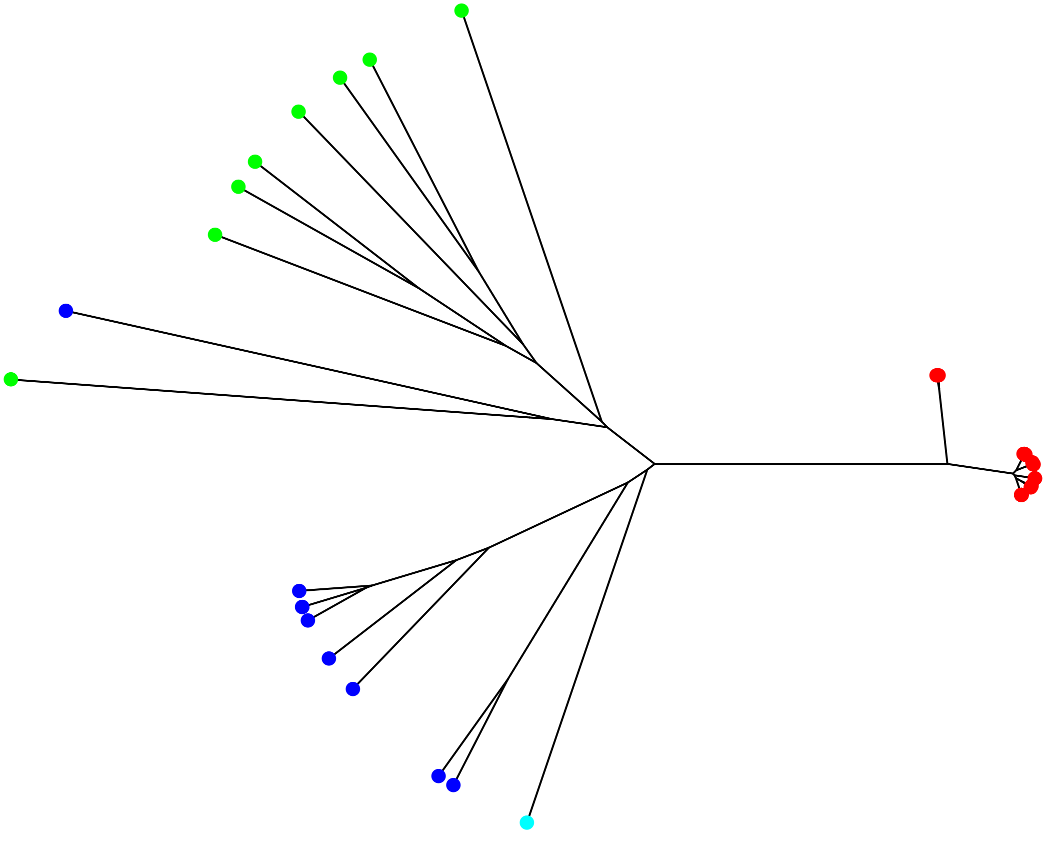

Gene family 1340 : 30S ribosomal protein S7

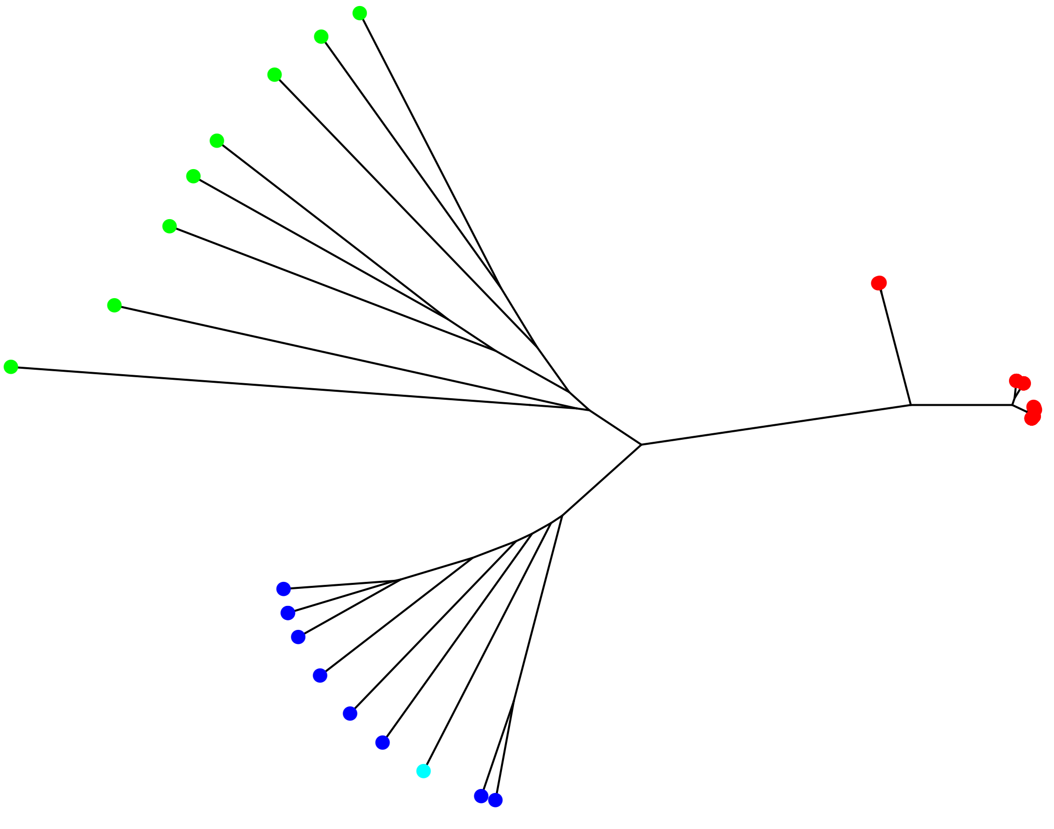

Gene family 1341 : 30S ribosomal protein S12

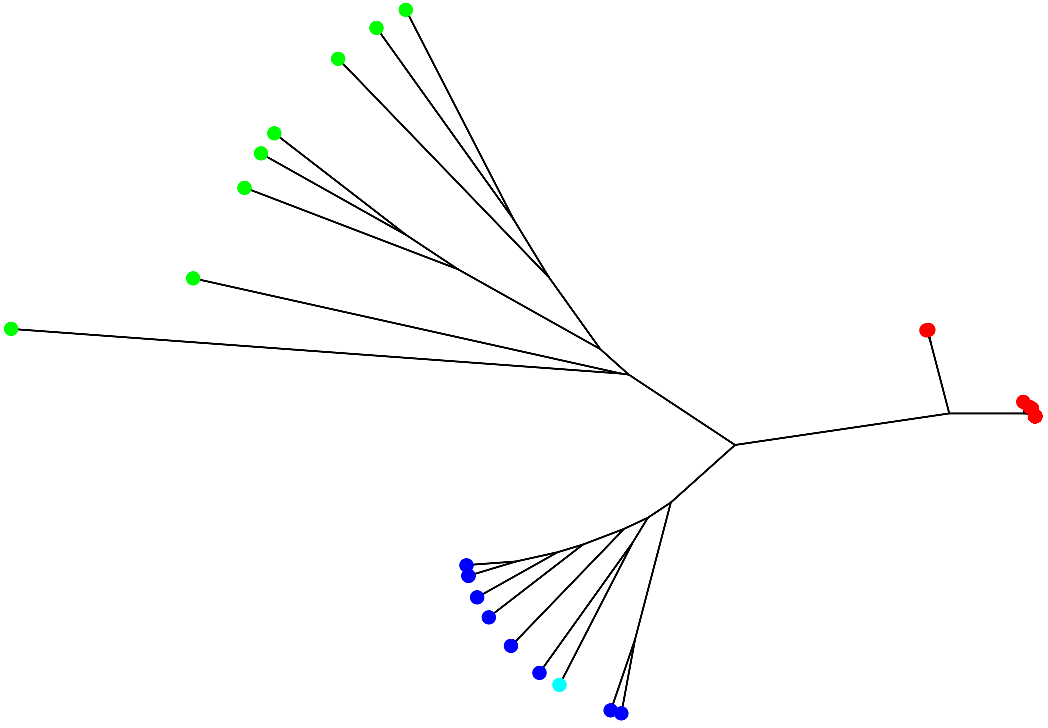

Gene family 1349 : acetoin dehydrogenase E2 subunit dihydrolipoyllysine−residue acetyltransferase

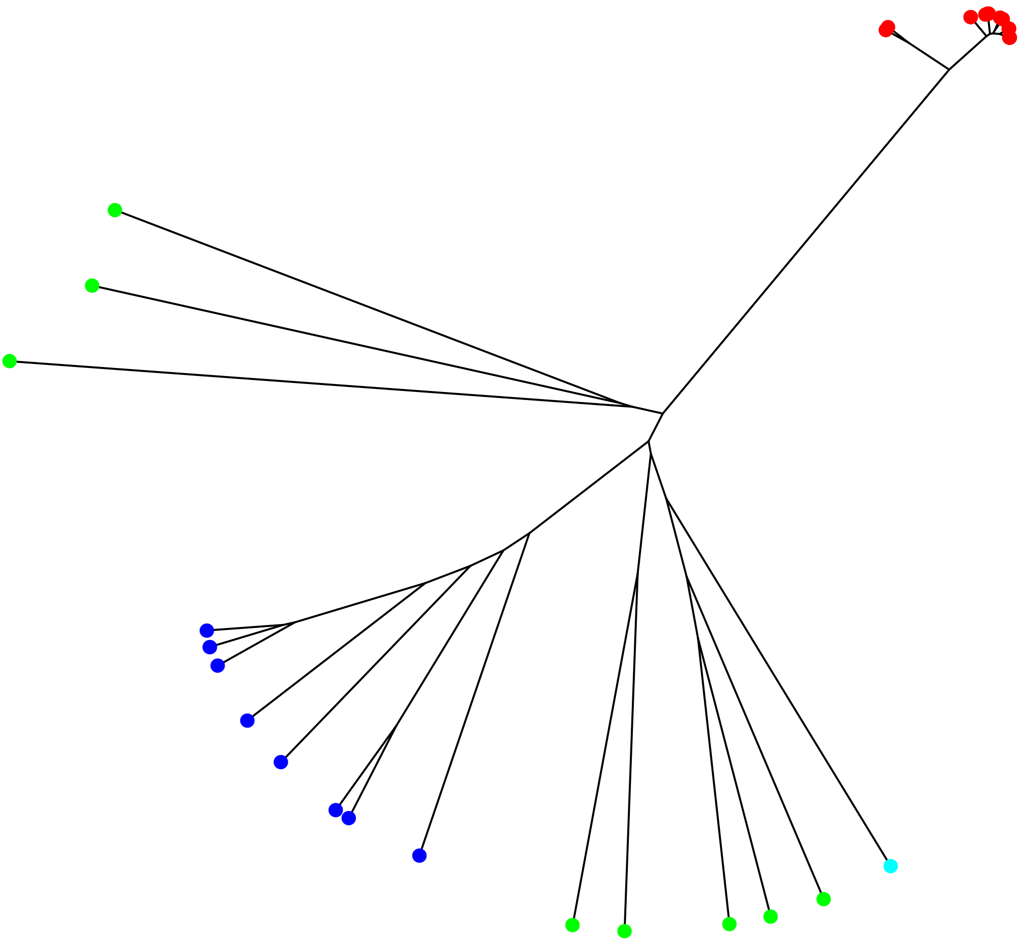

Gene family 1361 : Trp operon repressor

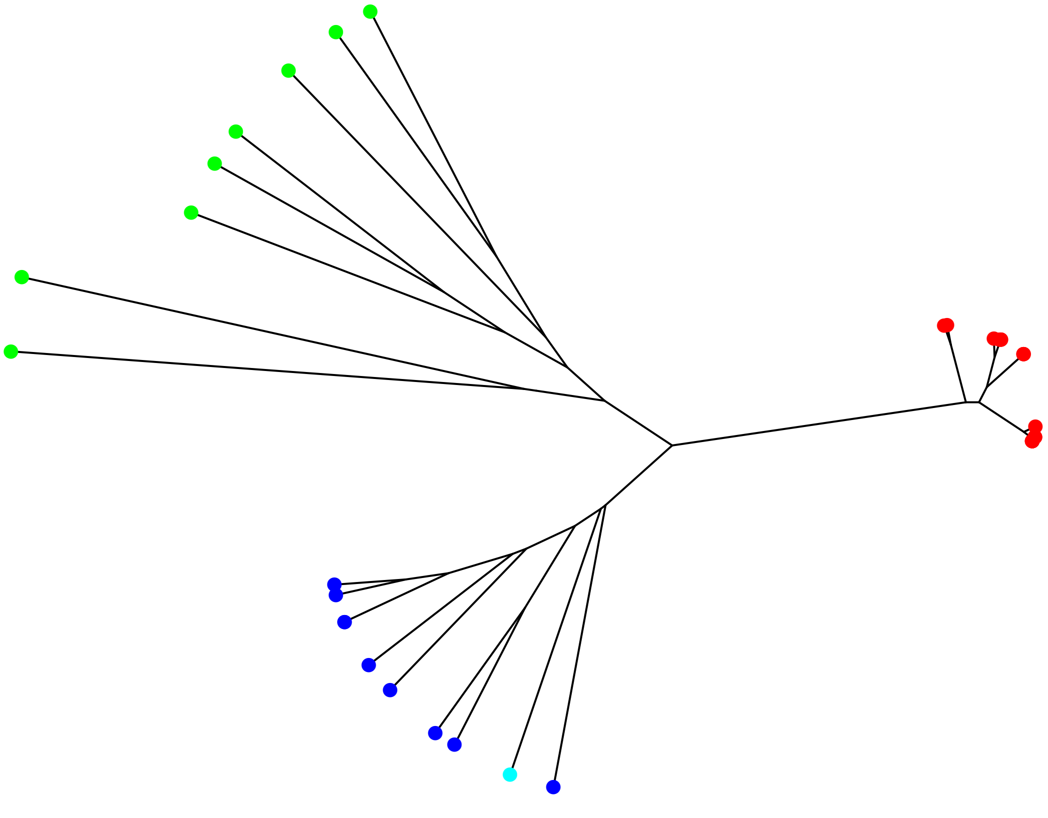

Supplementary Figure 4B. **UPGMA phylogenies of all the divergent gene family/clusters identified.** *Legionella* accessory gene phylogenies that showed evidence of potential HGT using gene-based phylogeny**.**

Gene family 234 : putative DNA endonuclease SmrA

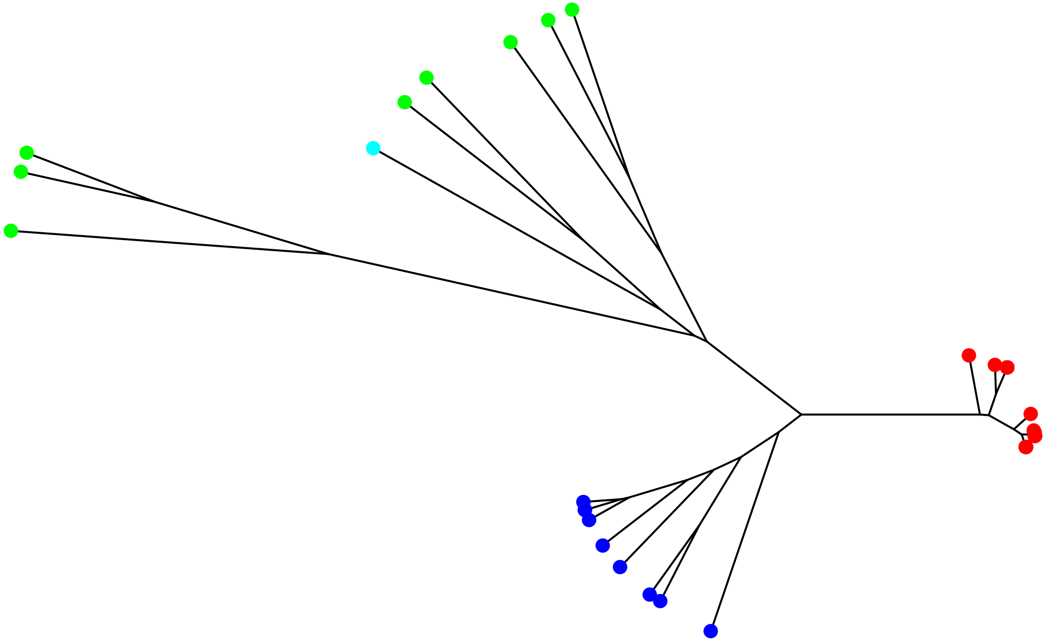

Gene family 360 : 50S ribosomal protein L32

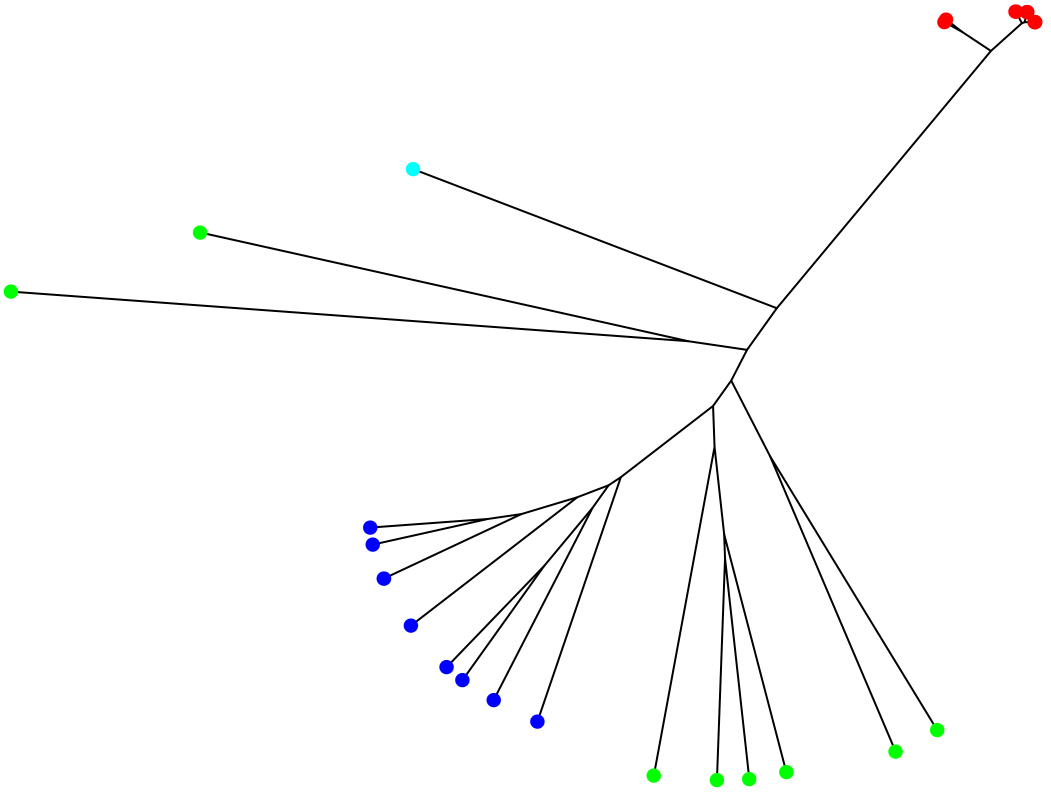

Gene family 366 : Ribonuclease HI

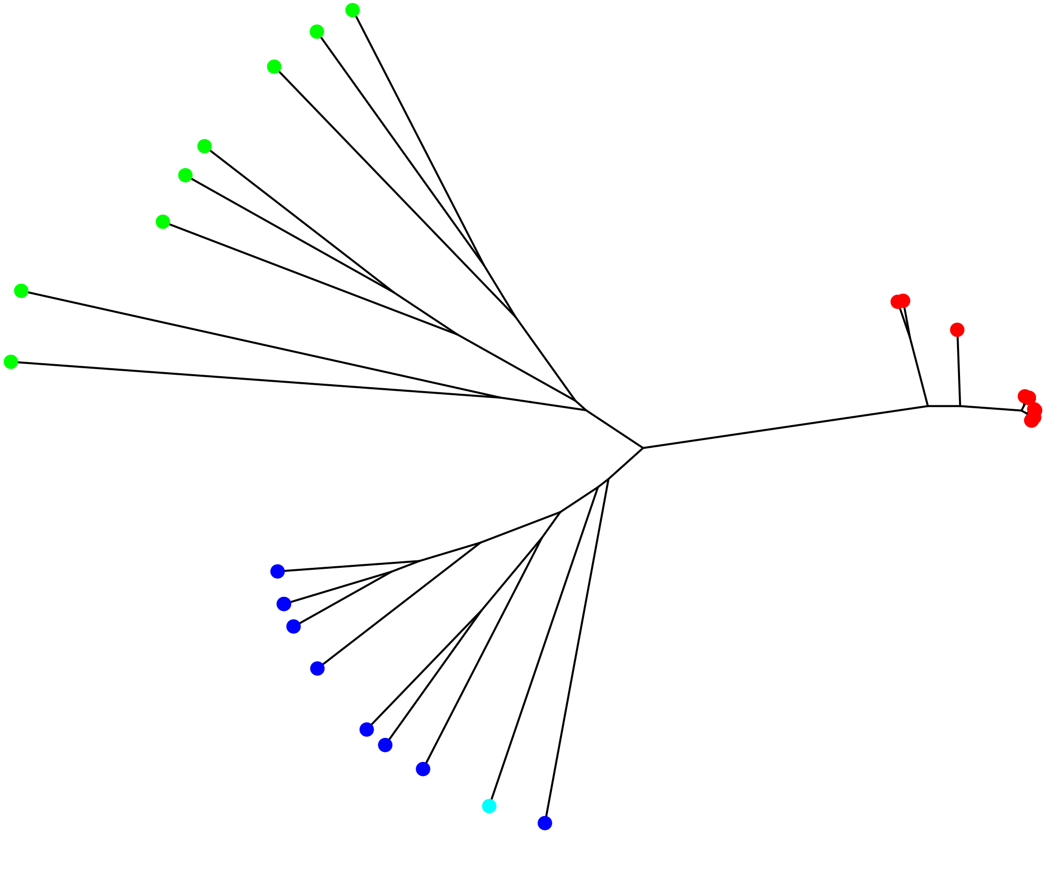

Gene family 373 : Dicarboxylic acid uptake system A

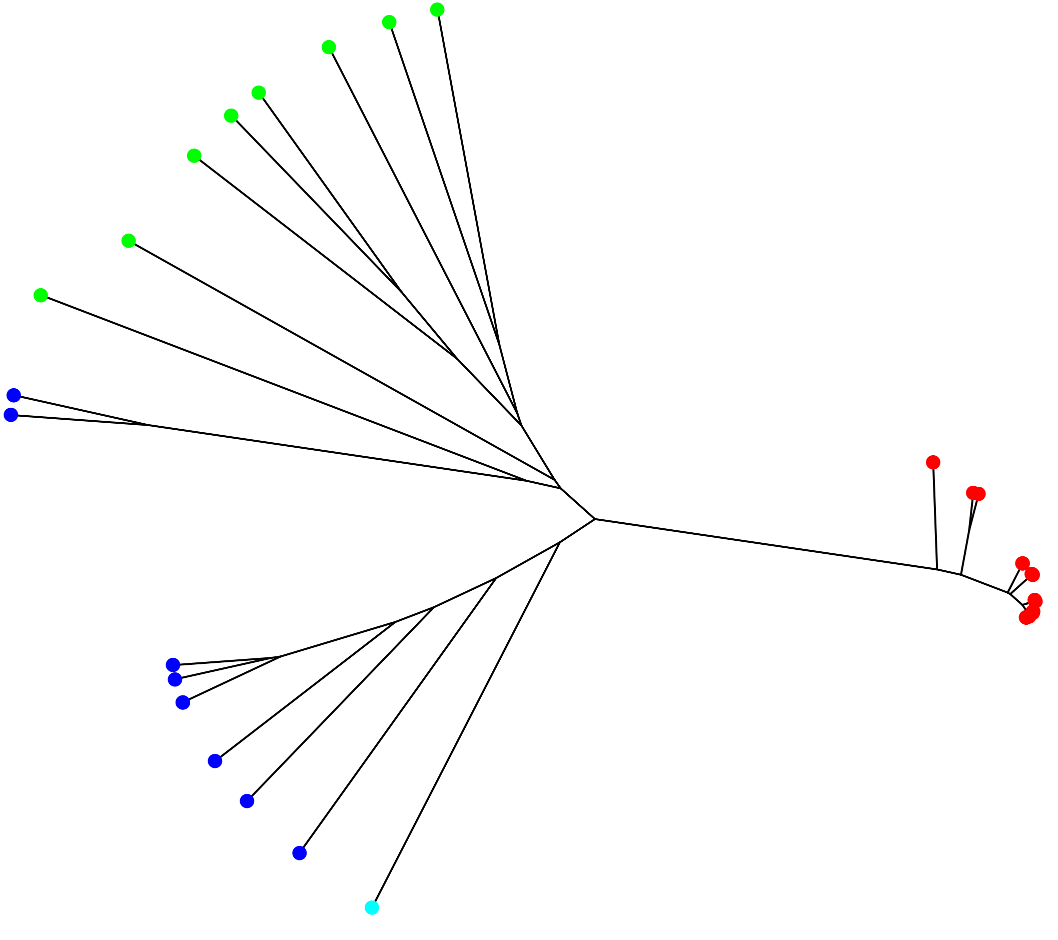

Gene family 380 : Rod shape−determining protein MreB

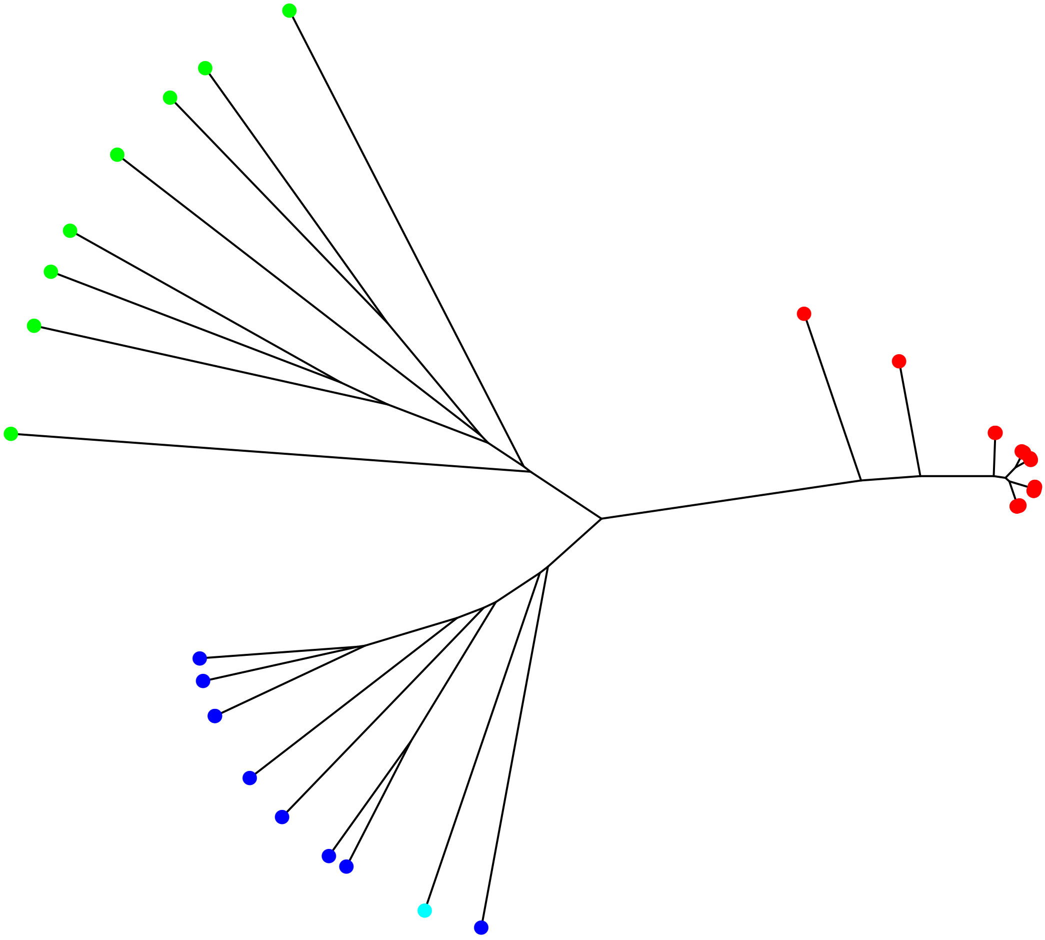

Gene family 399 : 3−deoxy−D−manno−octulosonate 8−phosphate phosphatase KdsC

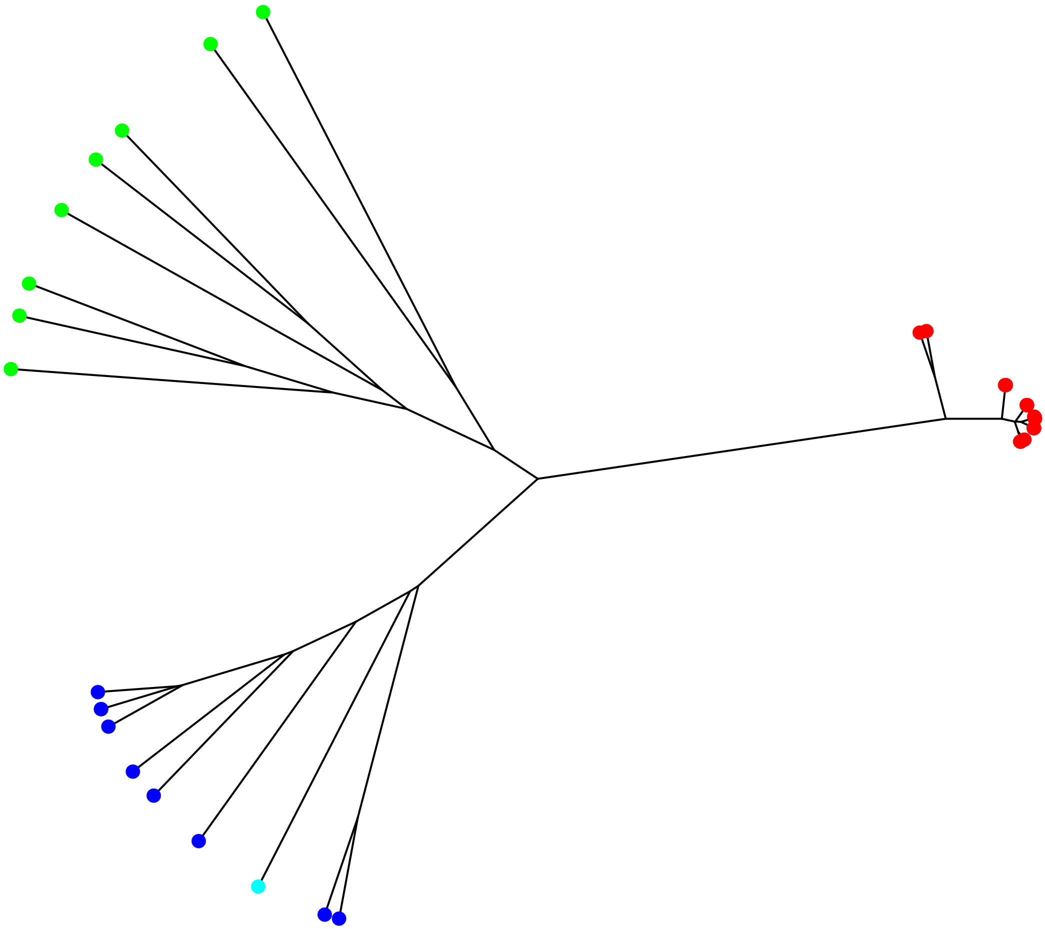

Gene family 411 : Cytochrome c−type biogenesis protein CcmB

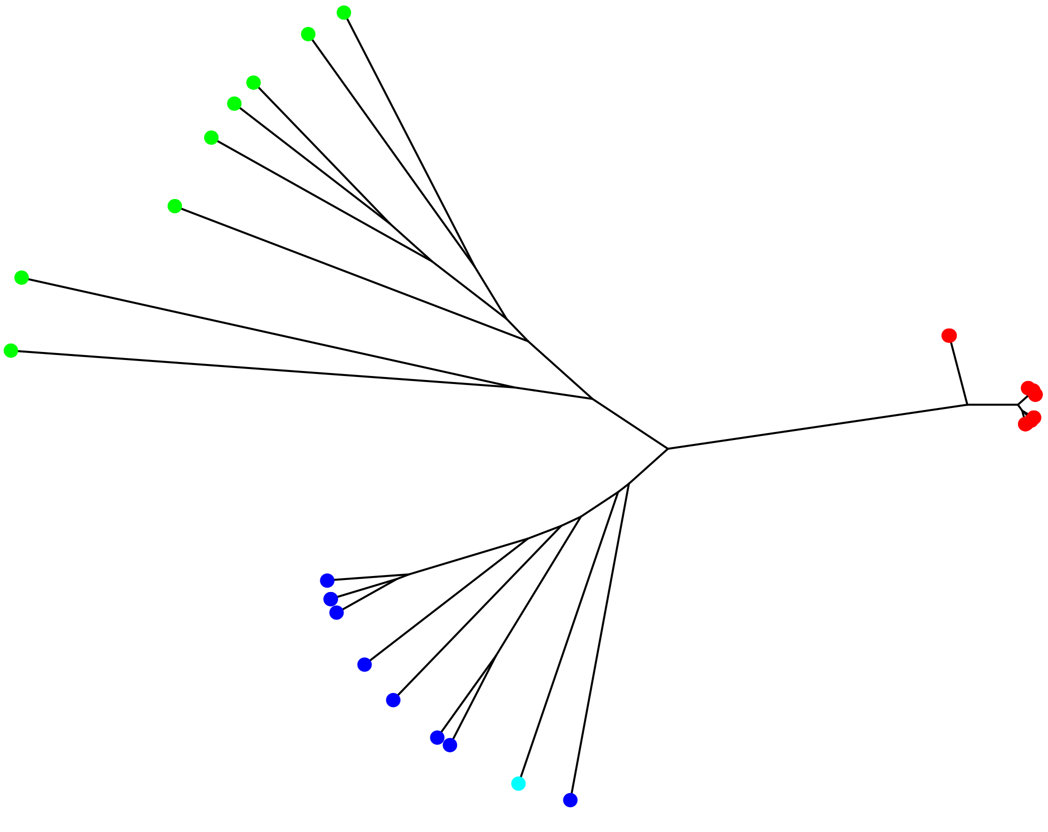

Gene family 443 : 50S ribosomal protein L9

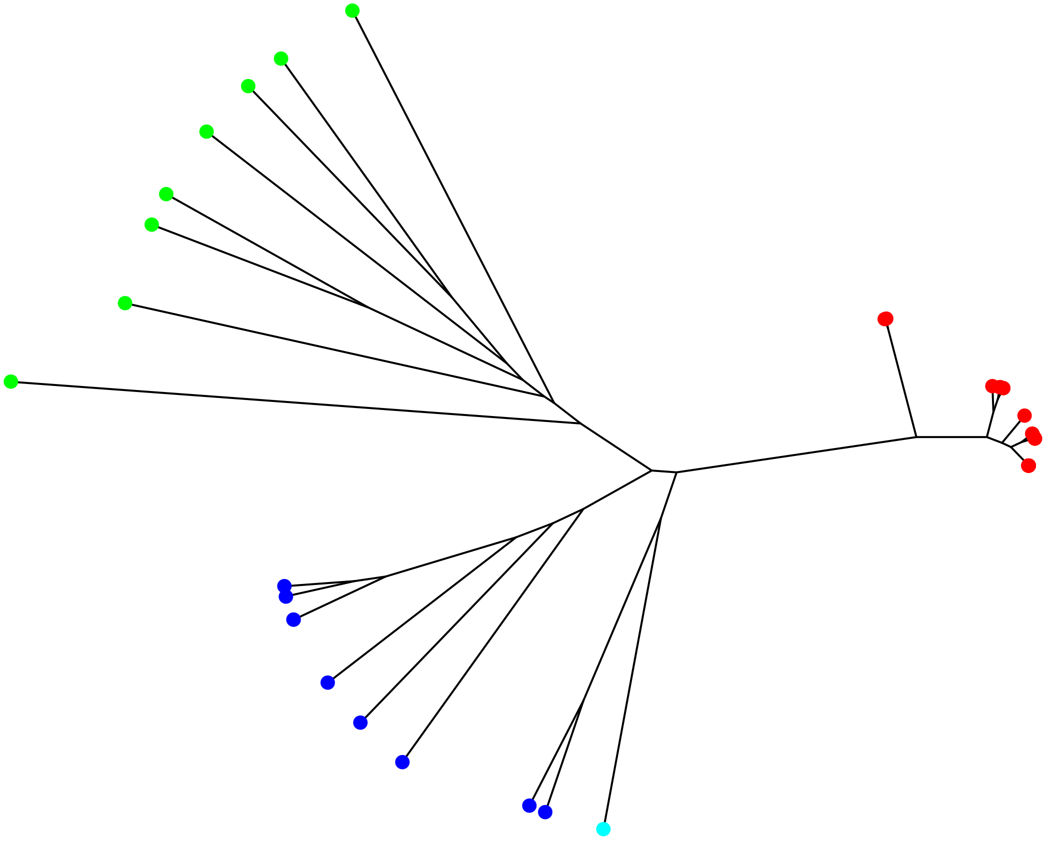

Gene family 476 : Rubredoxin−1

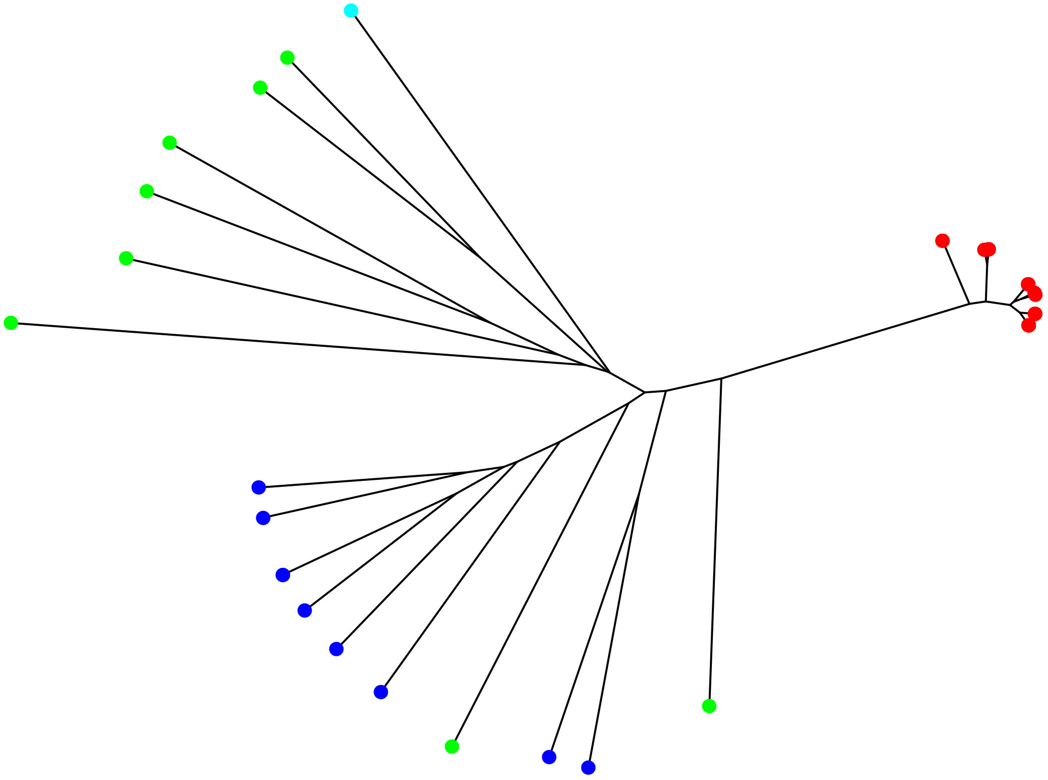

Gene family 485 : Ribosomal RNA large subunit methyltransferase M

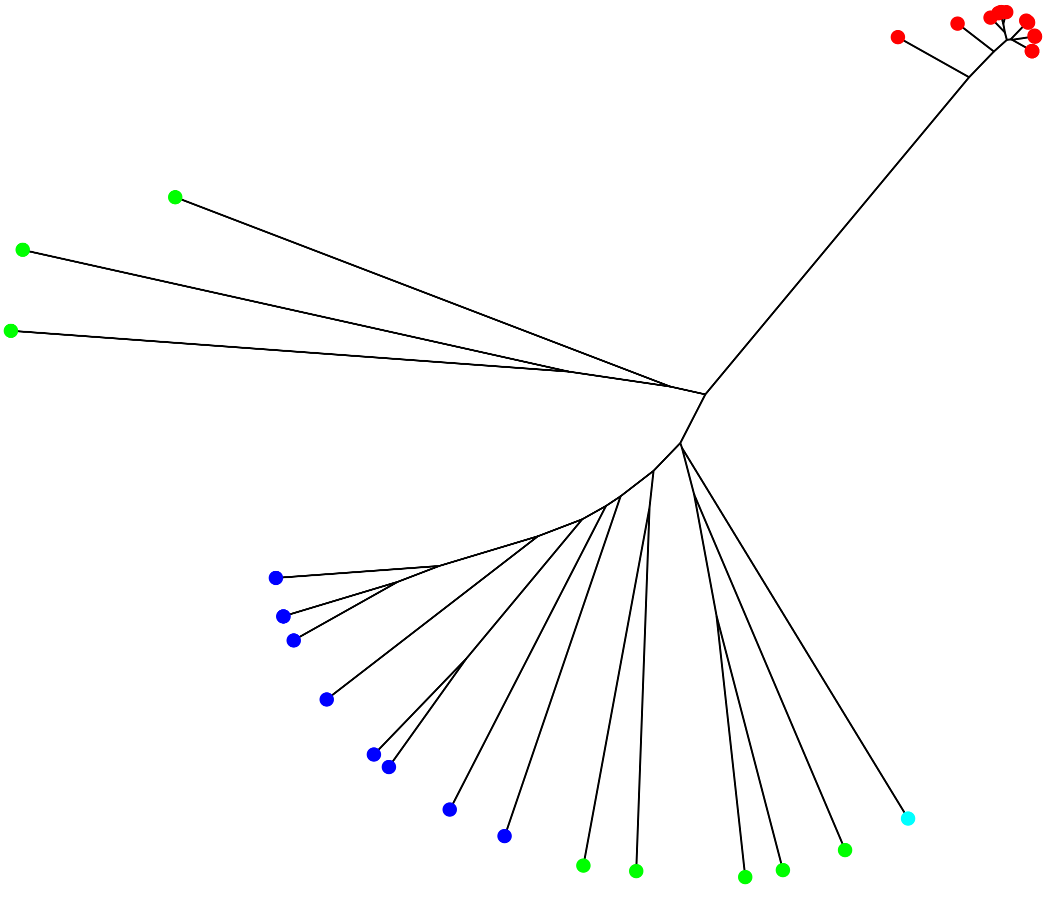

Gene family 486 : General secretion pathway protein F

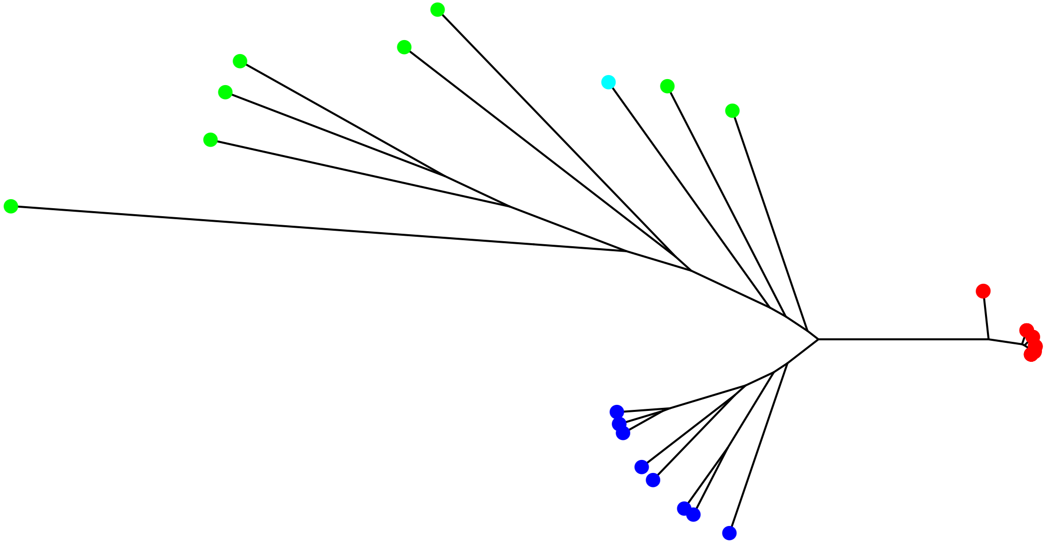

Gene family 487 : Type II traffic warden ATPase

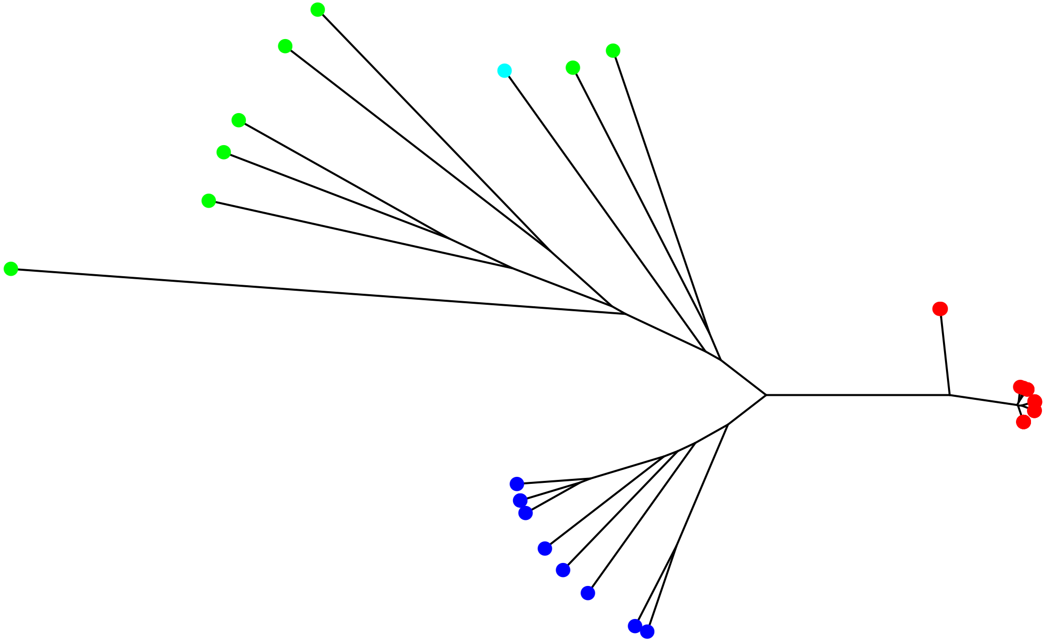

Gene family 491 : putative membrane protein

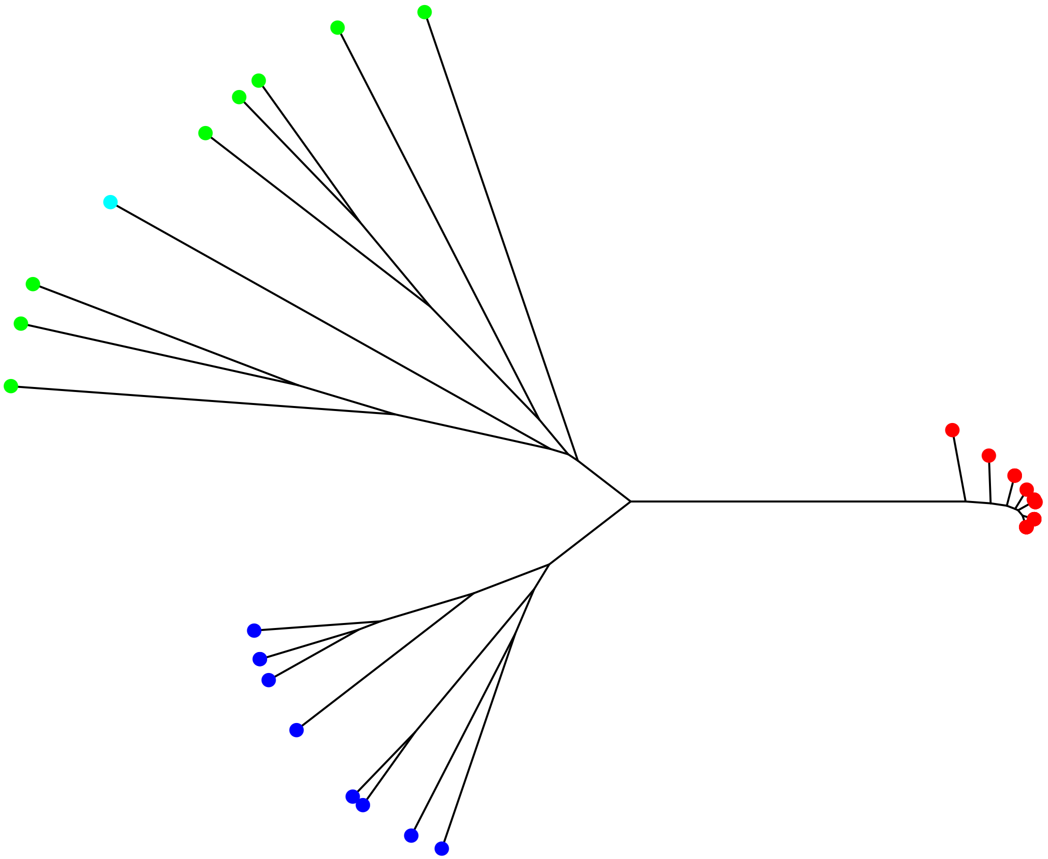

Gene family 536 : Nicotinate−nucleotide adenylyltransferase

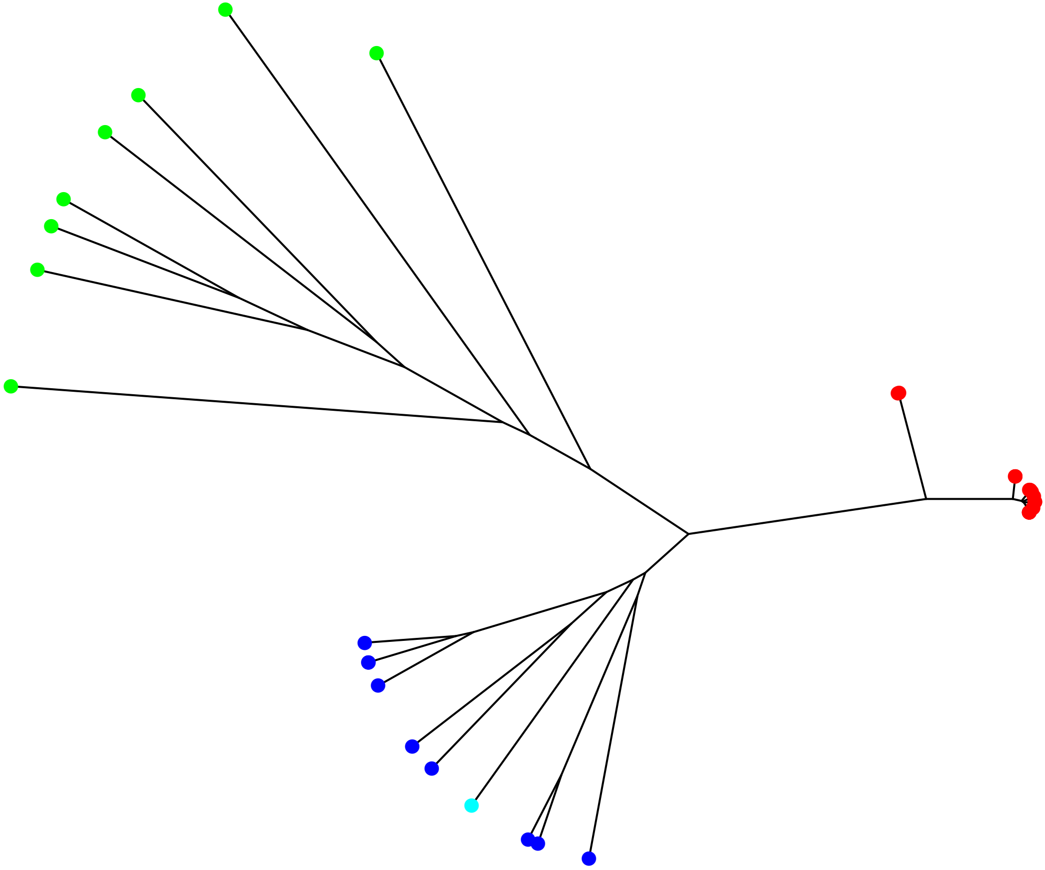

Gene family 556 : Iron−sulfur cluster assembly protein

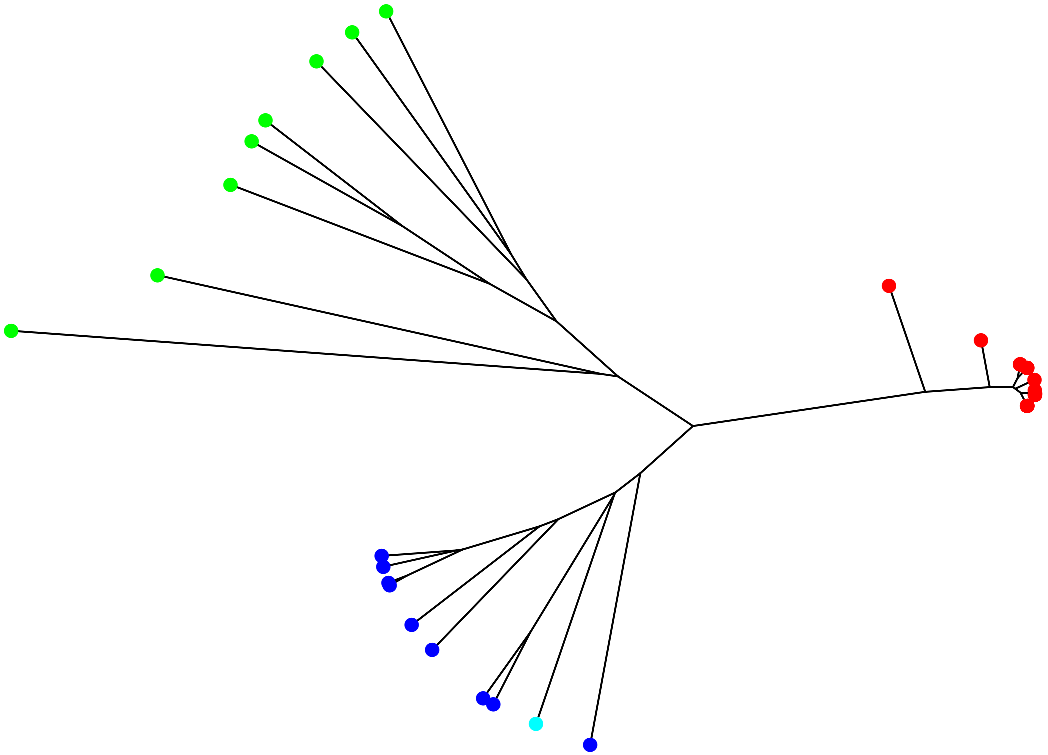

Gene family 561 : Arginine repressor

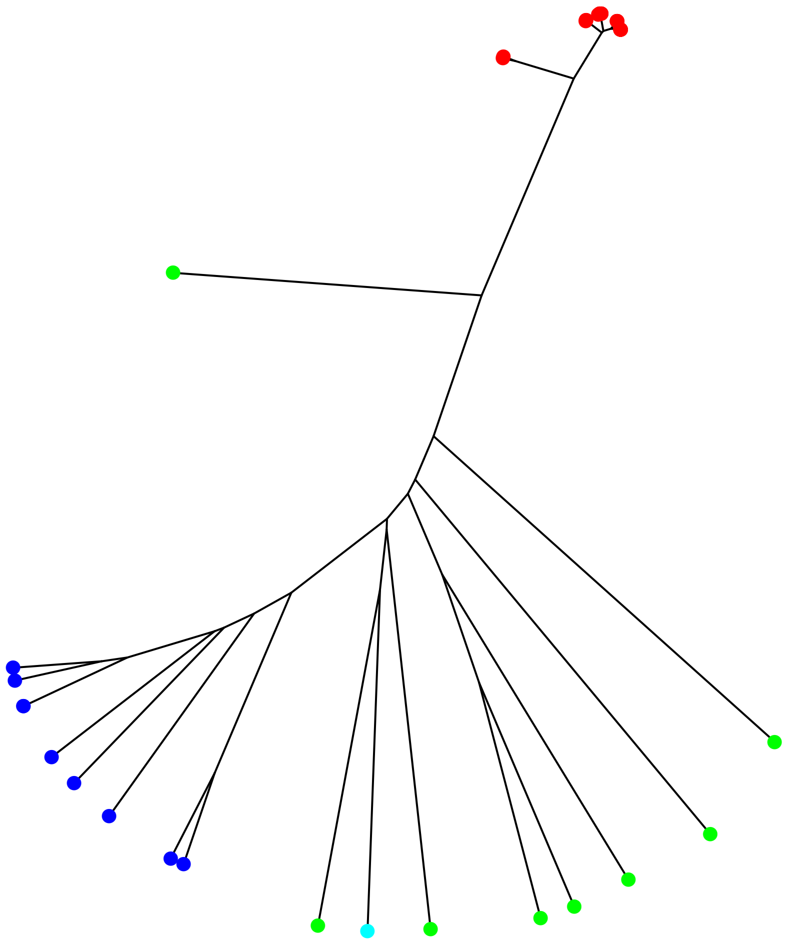

Gene family 602 : Host factor−I protein

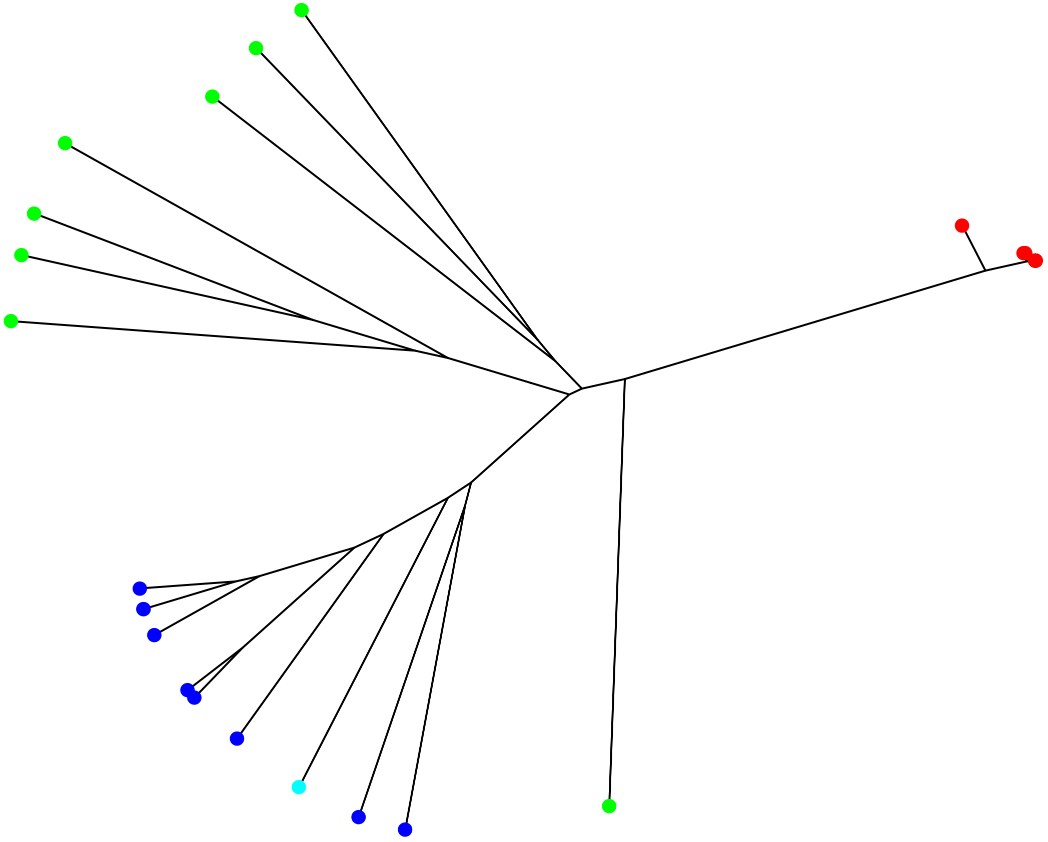

Gene family 611 : Low−affinity inorganic phosphate transporter 1

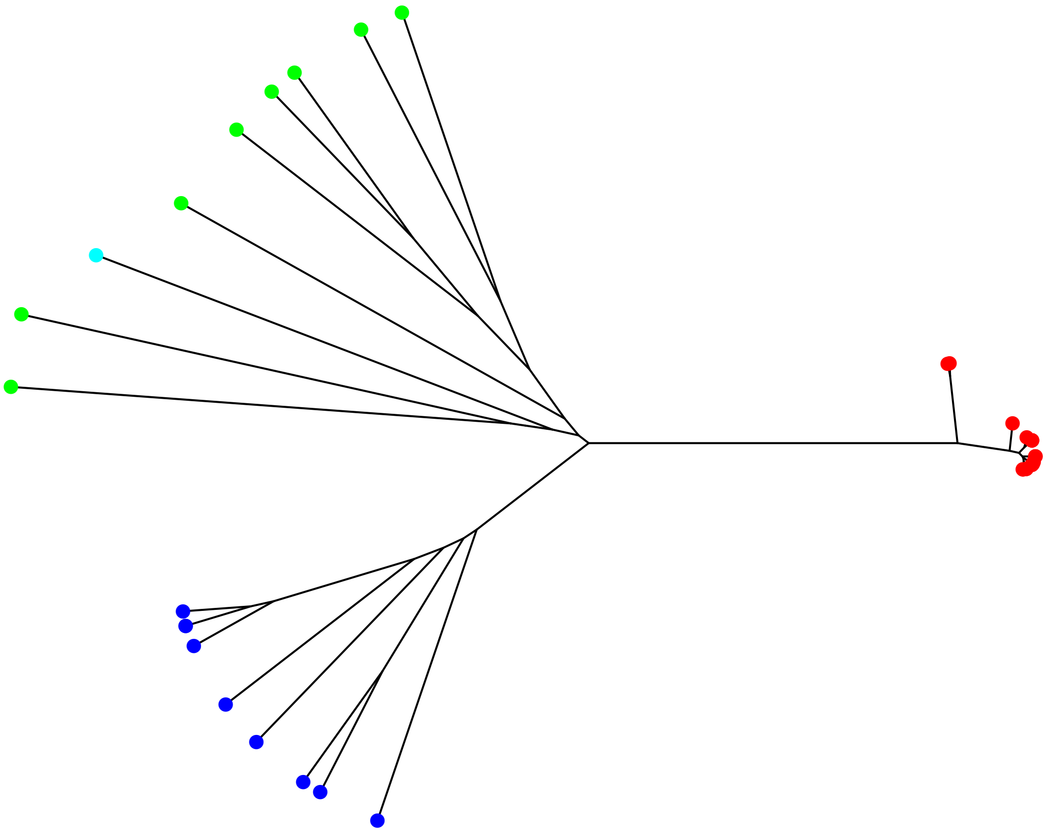

Gene family 621 : Modulator of FtsH protease HflK

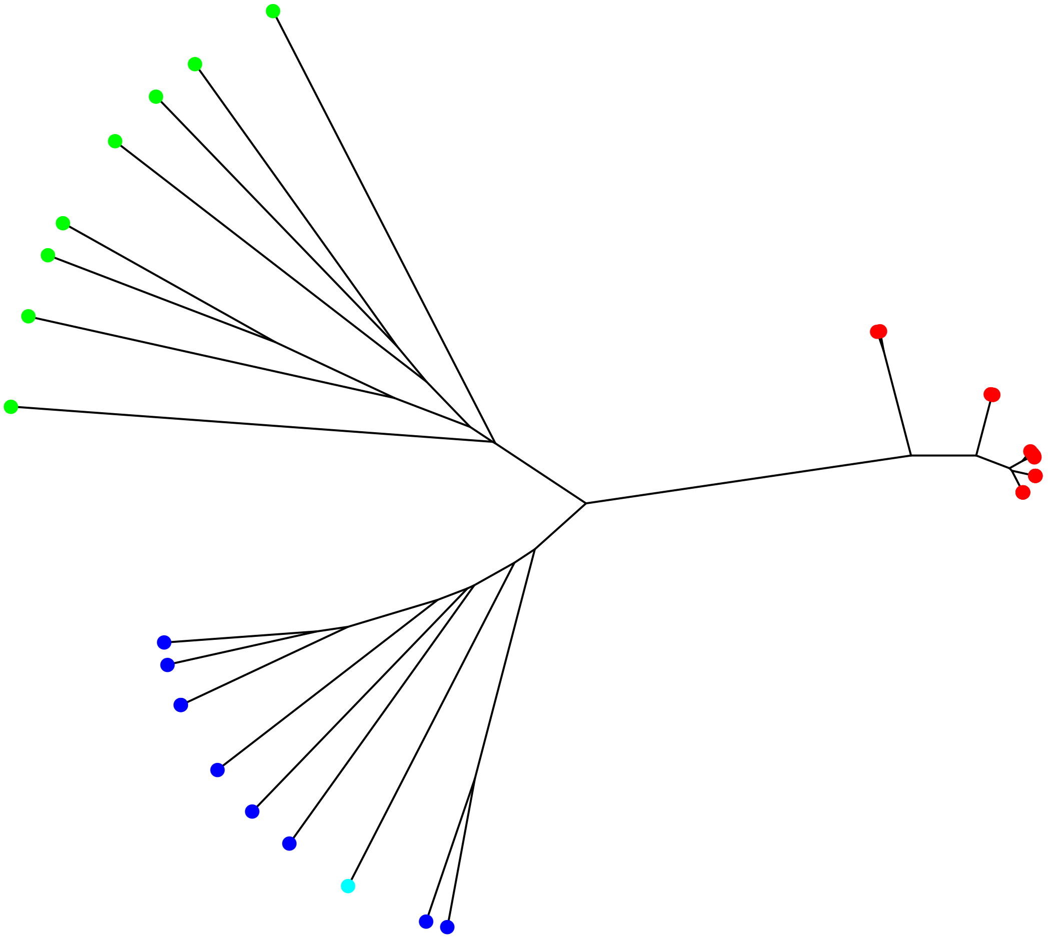

Gene family 622 : Deoxycytidine triphosphate deaminase

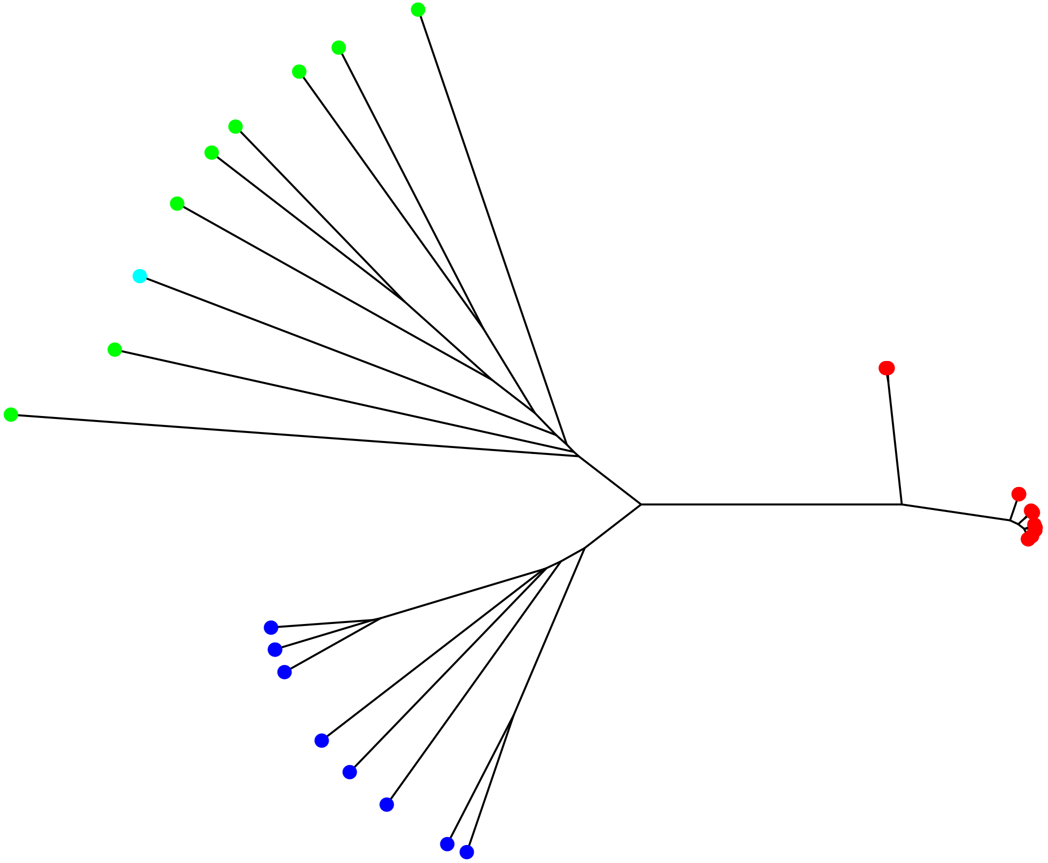

Gene family 633 : hypothetical protein

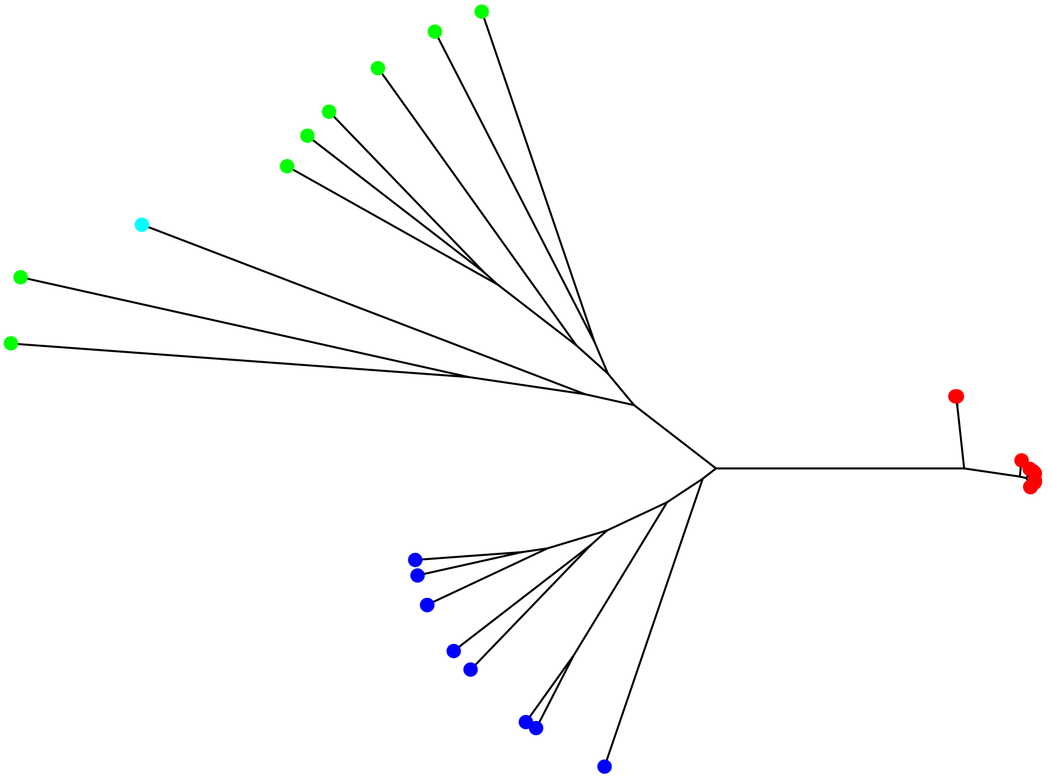

Gene family 667 : 50S ribosomal protein L33

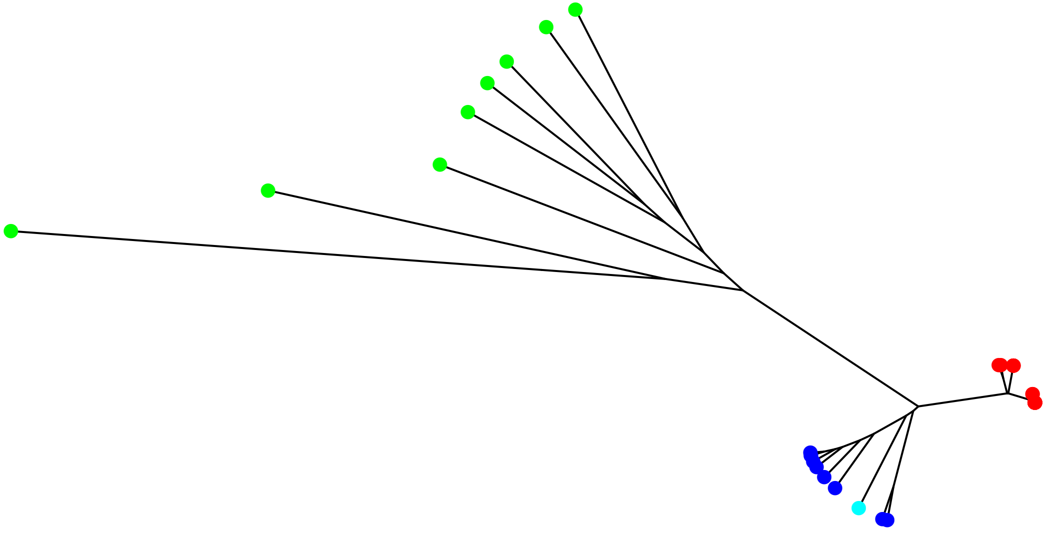

Gene family 695 : Zn−ribbon−containing, possibly RNA−binding protein and truncated derivatives

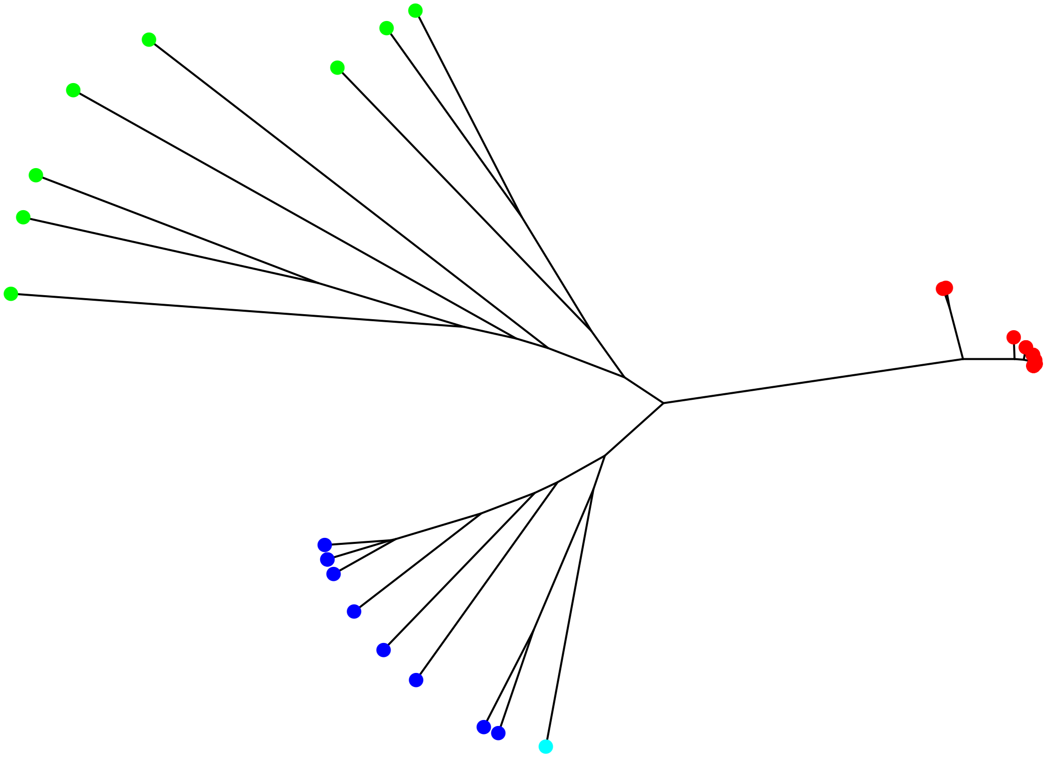

Gene family 732 : hypothetical protein

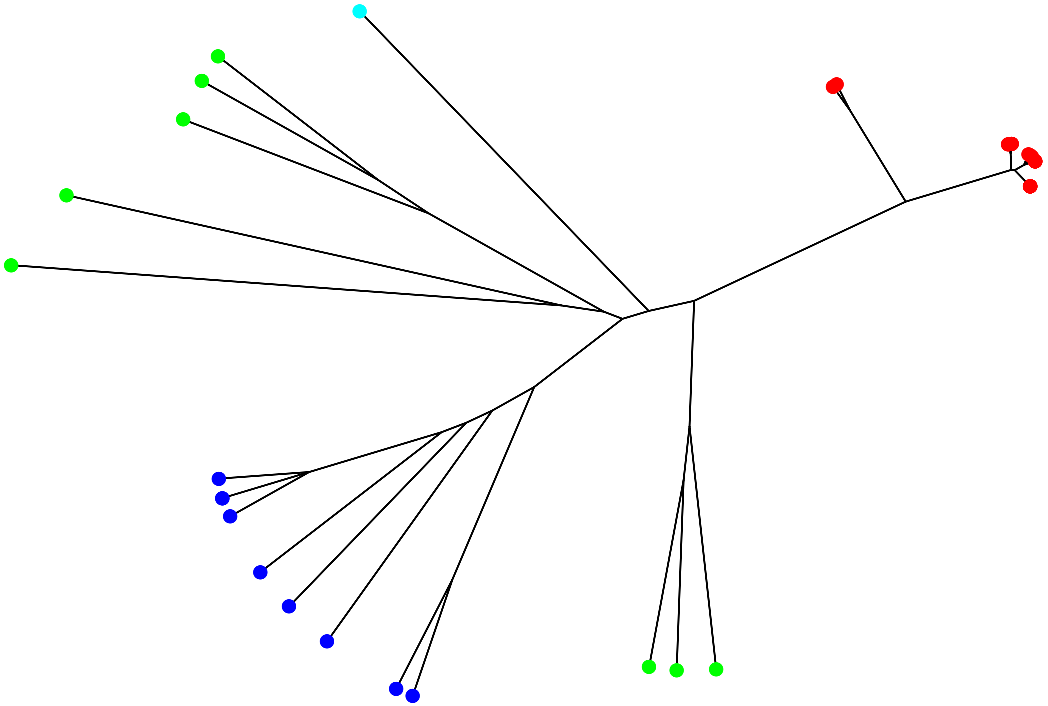

Gene family 748 : ProP effector

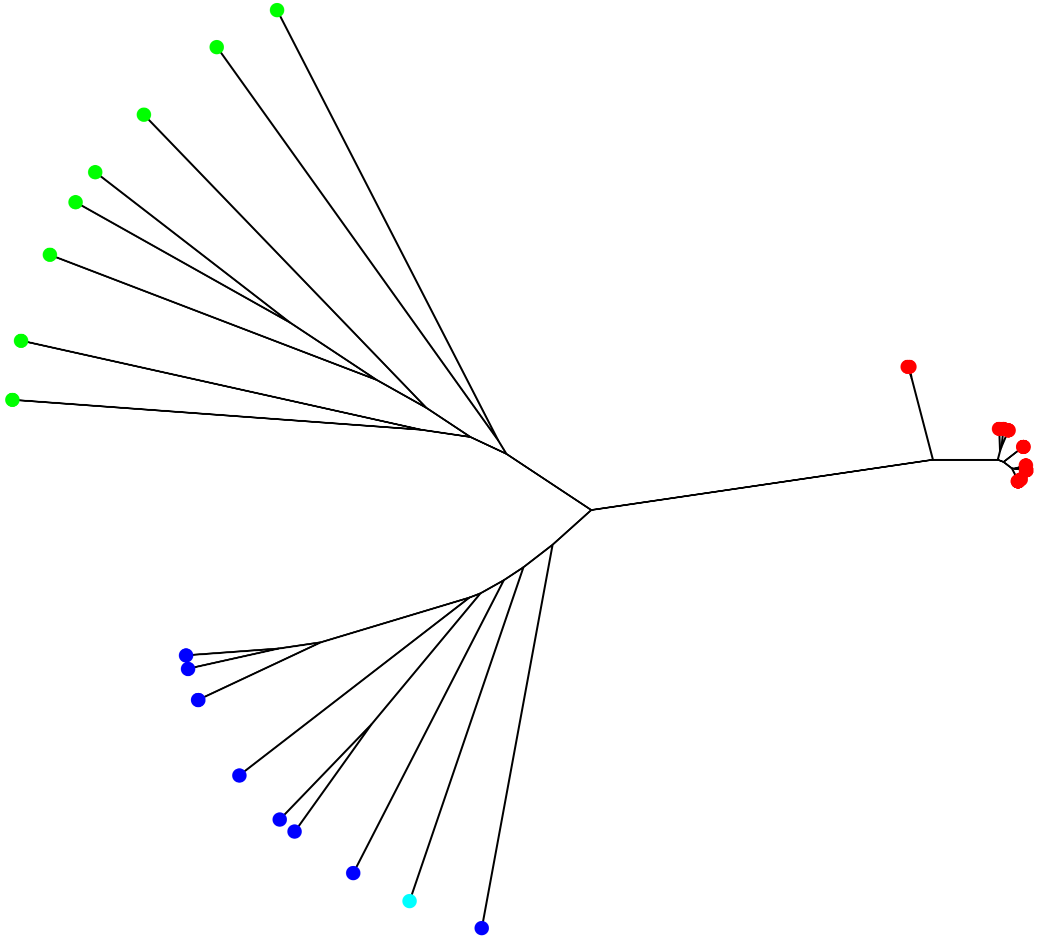

Gene family 770 : 50S ribosomal protein L19

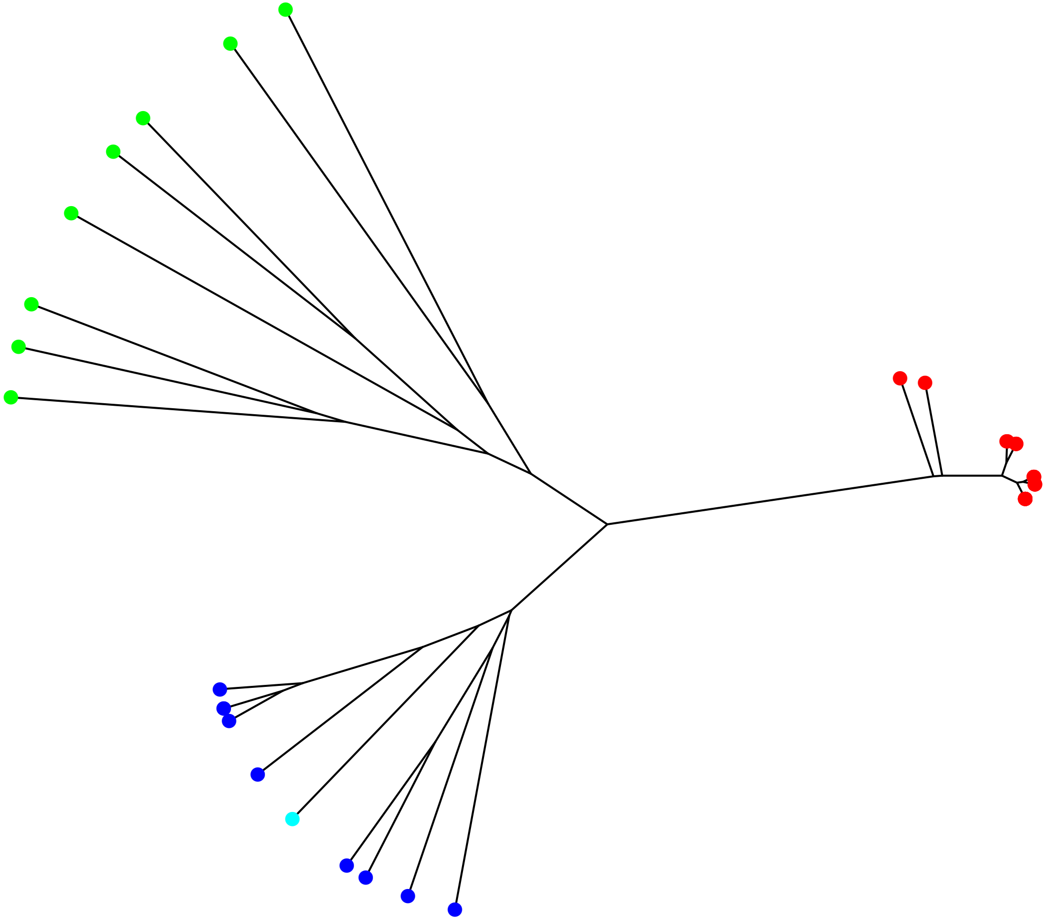

Gene family 796 : DNA−directed RNA polymerase subunit alpha

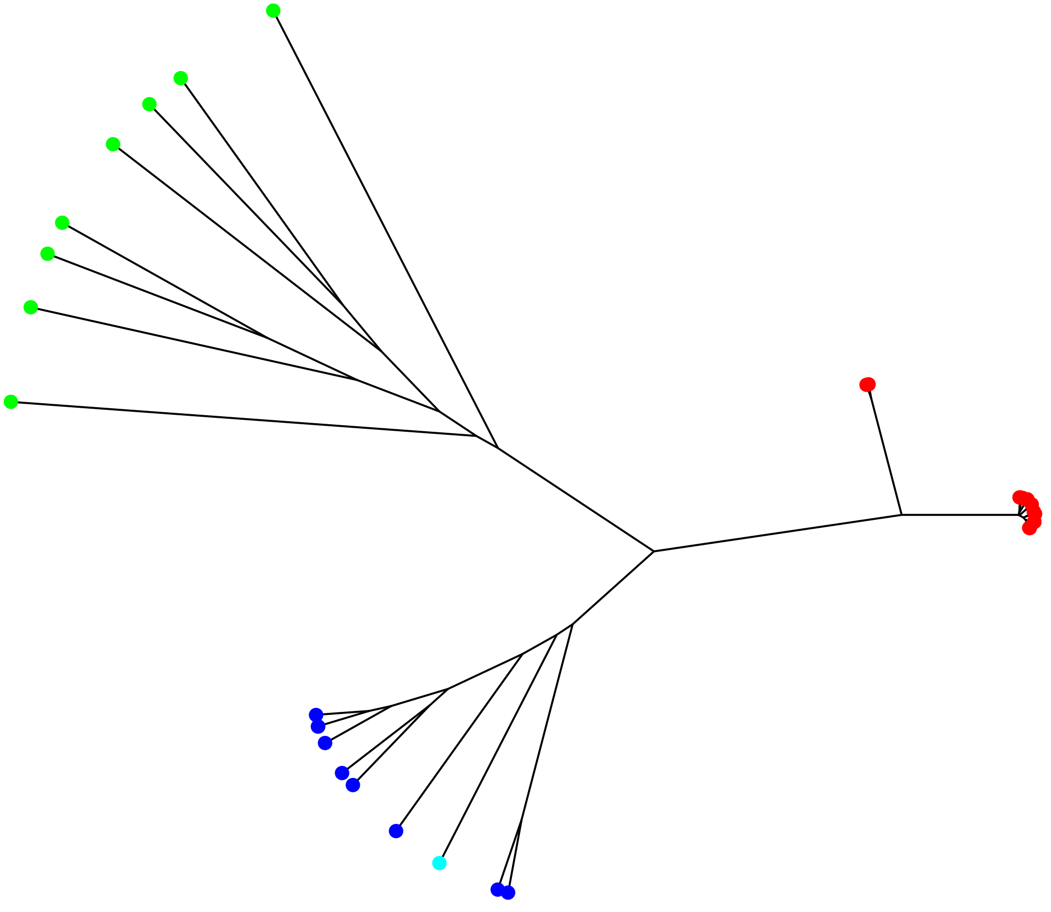

Gene family 801 : 50S ribosomal protein L15

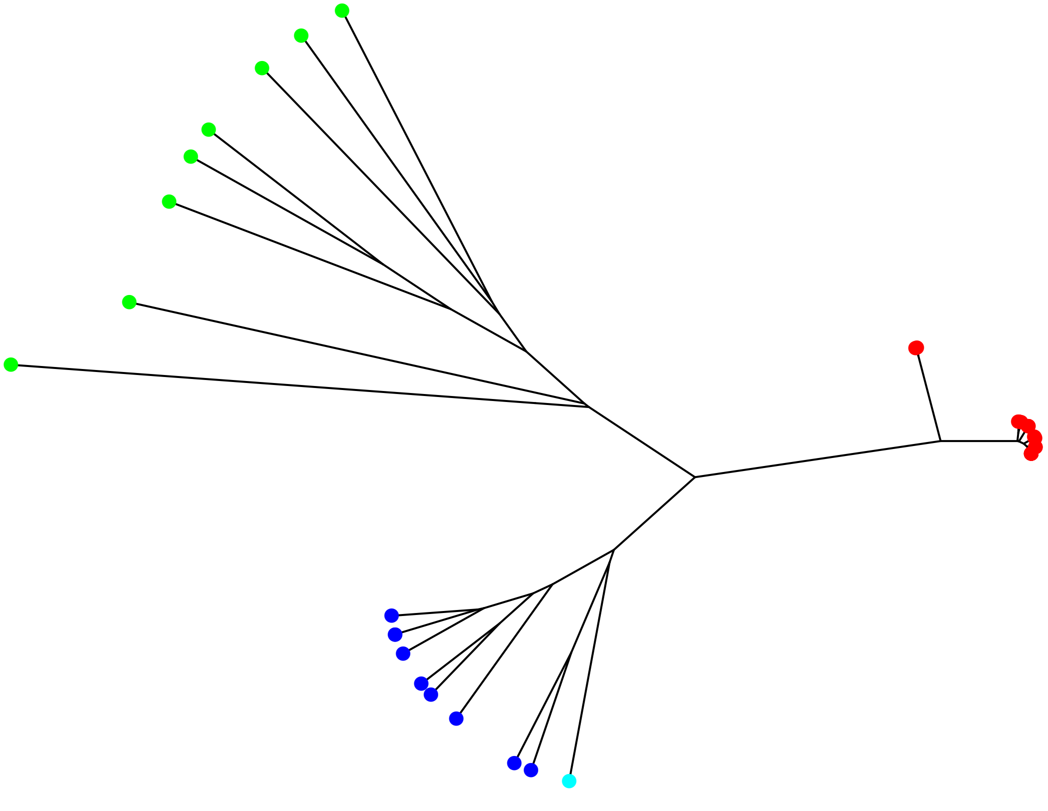

Gene family 809 : 50S ribosomal protein L14

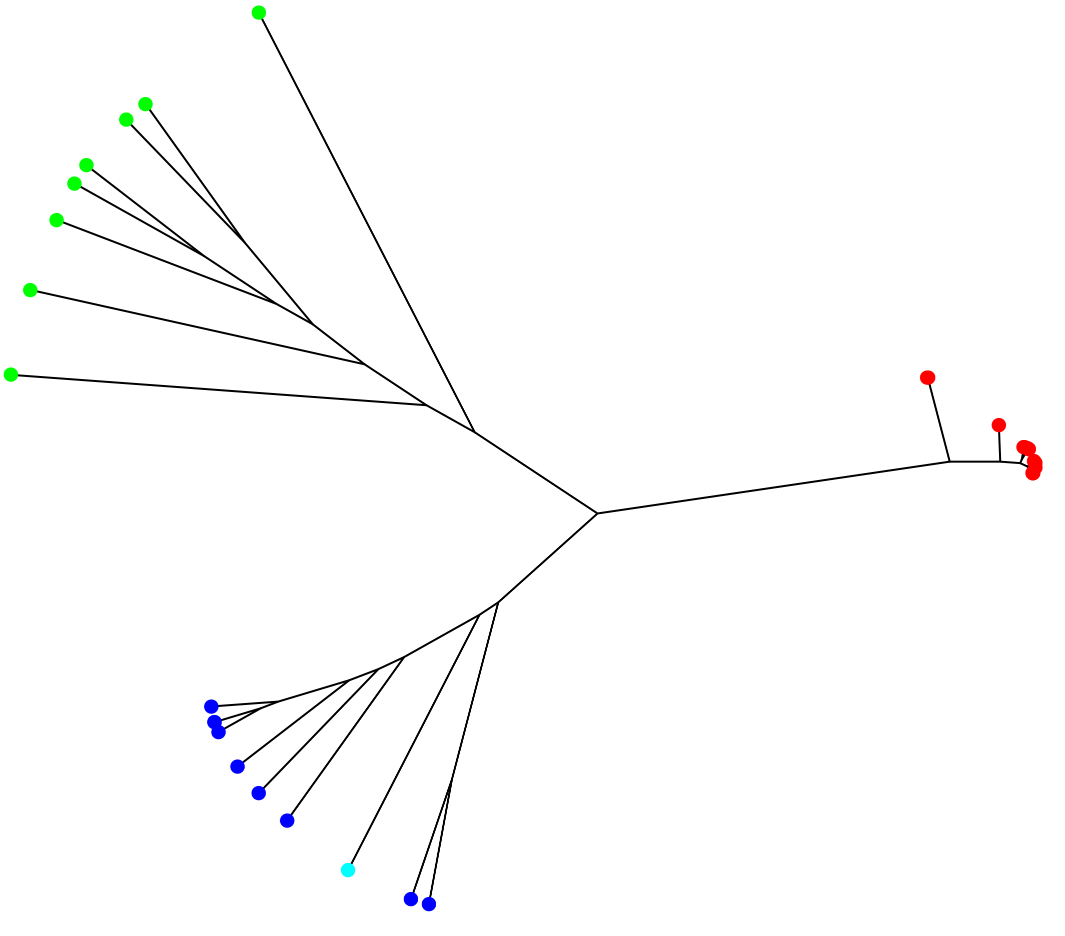

Gene family 810 : 30S ribosomal protein S17

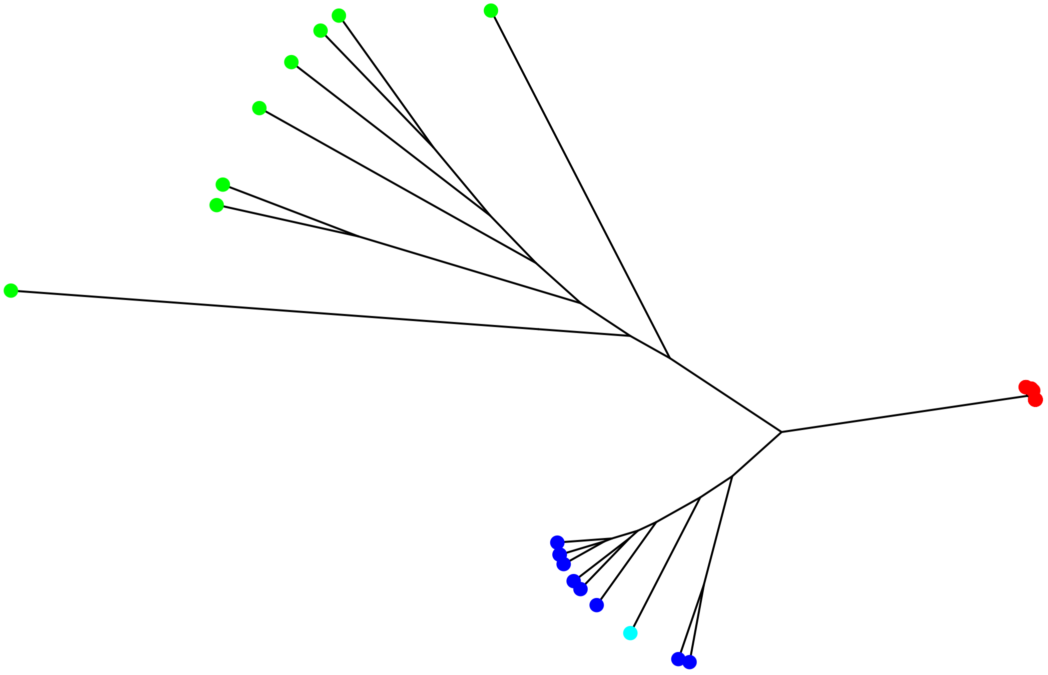

Gene family 818 : 50S ribosomal protein L3

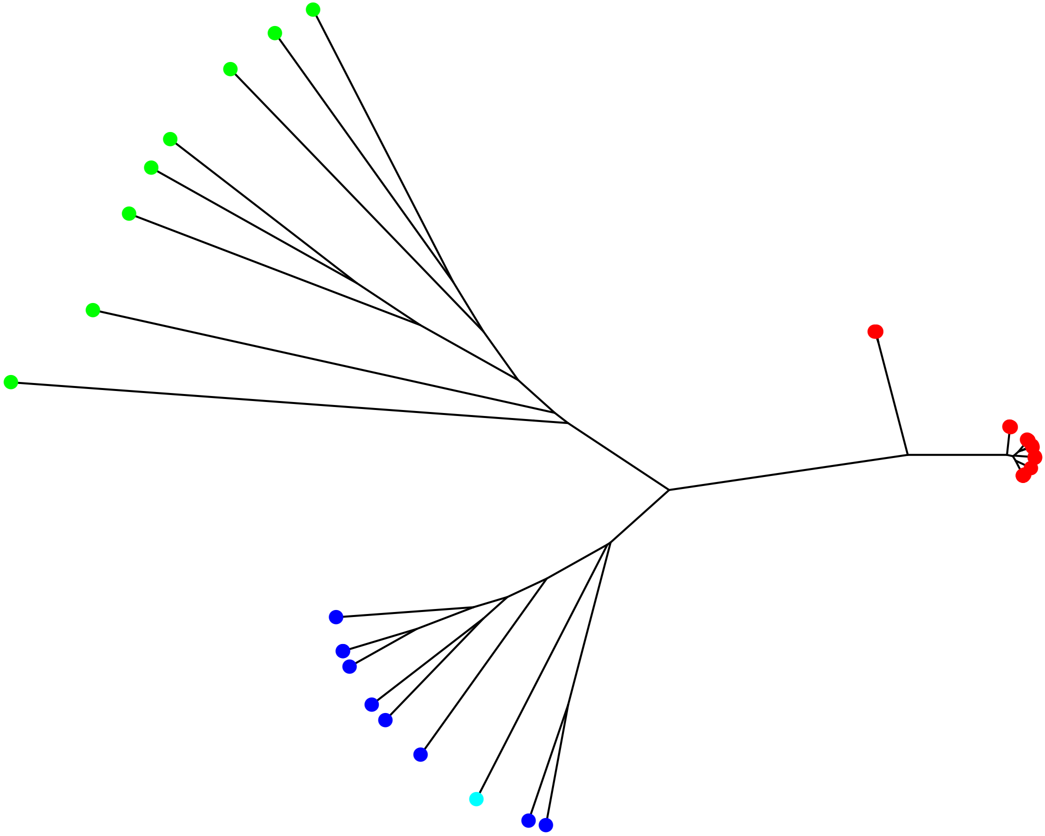

Gene family 828 : Succinyl−CoA ligase [ADP−forming] subunit alpha

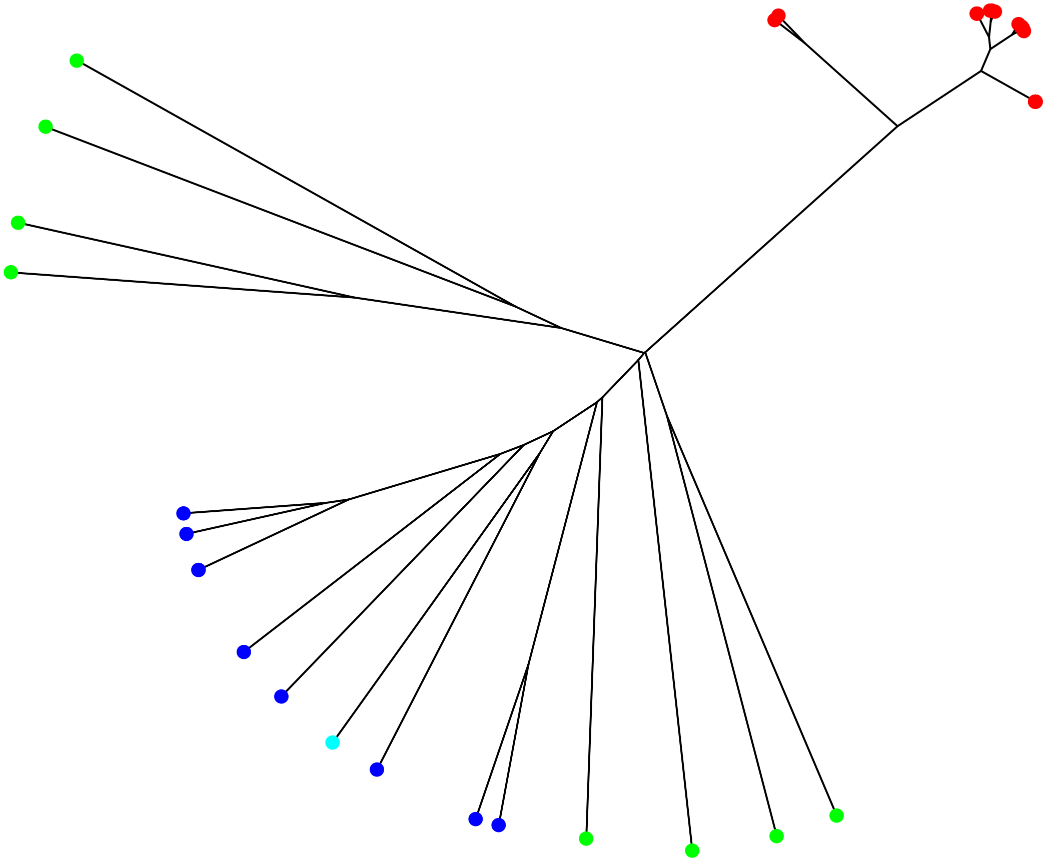

Gene family 835 : FeoC like transcriptional regulator

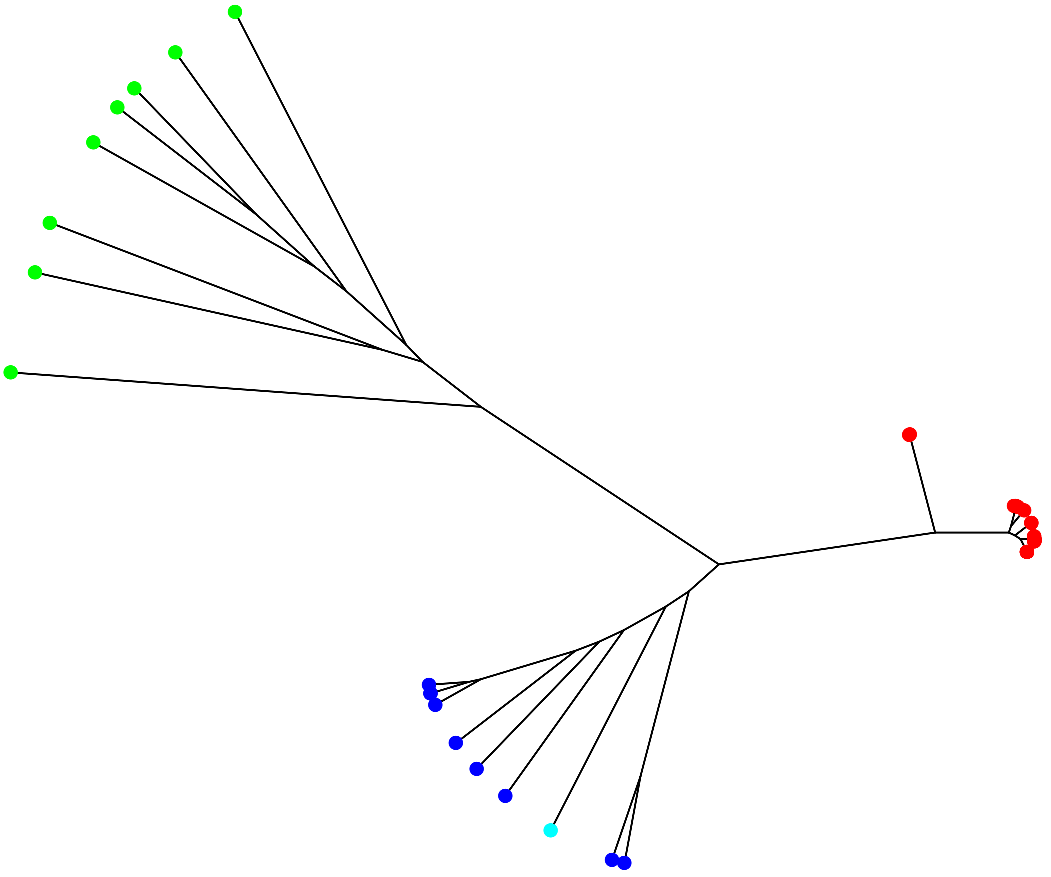

Gene family 850 : hypothetical protein

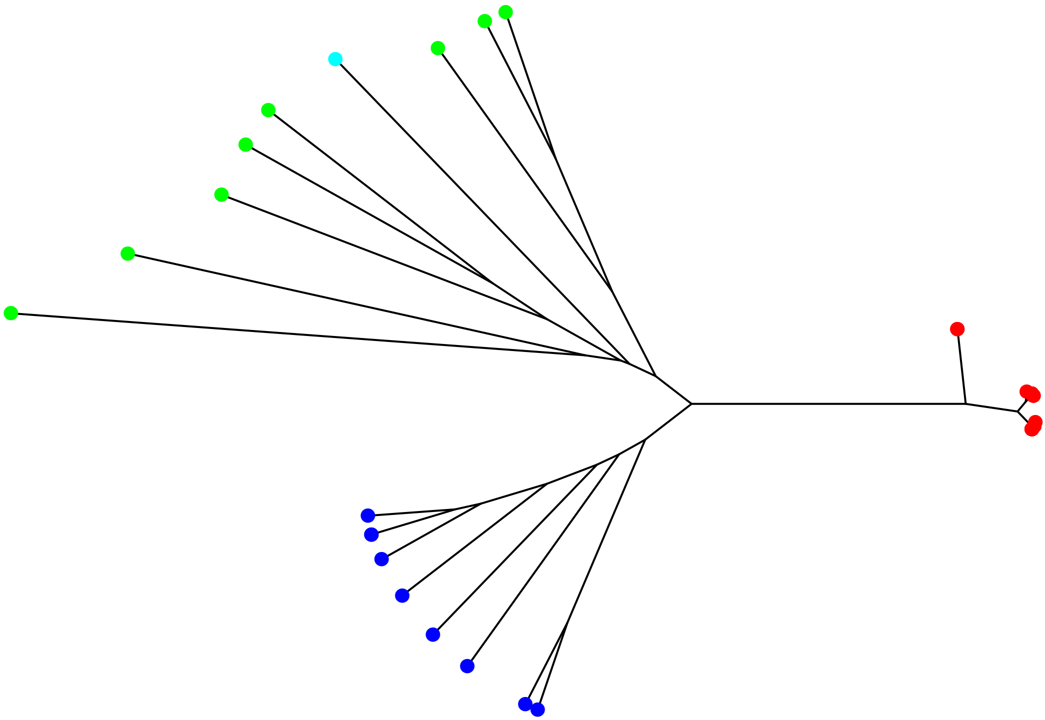

Gene family 853 : Phagosome trafficking protein DotA

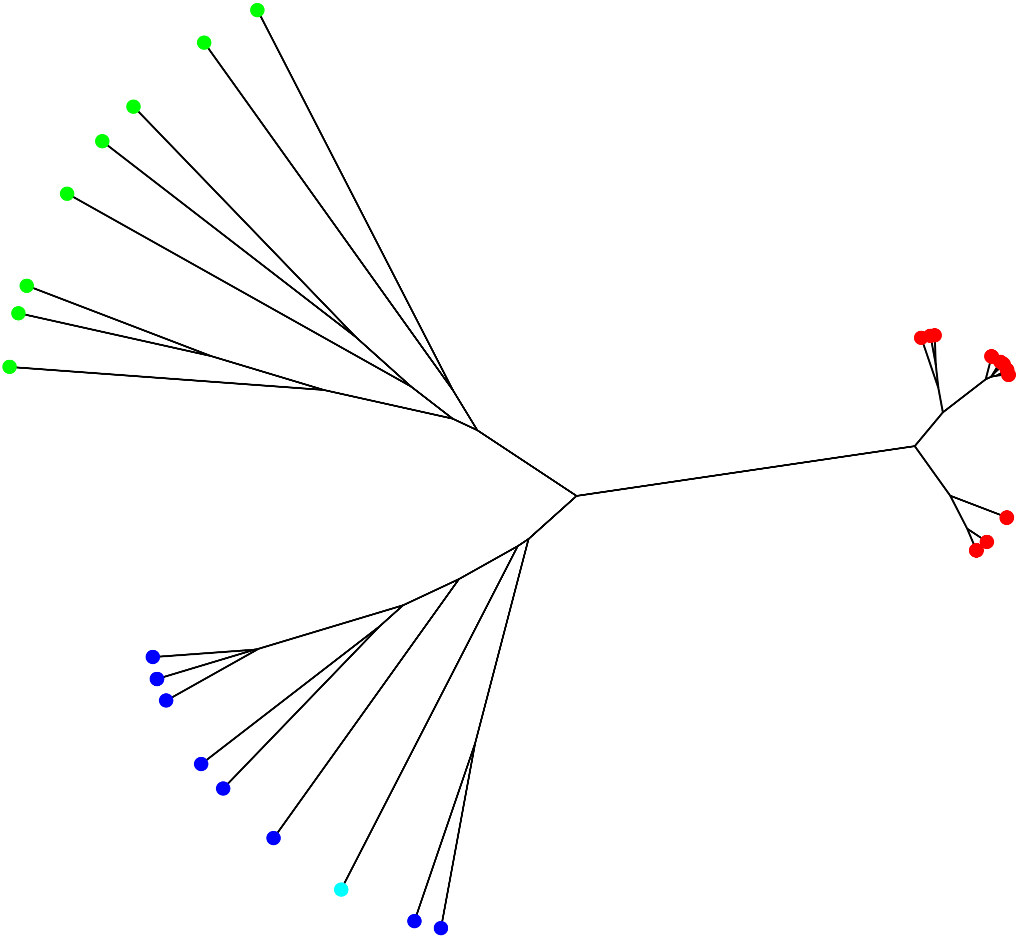

Gene family 875 : Translation initiation factor IF−3

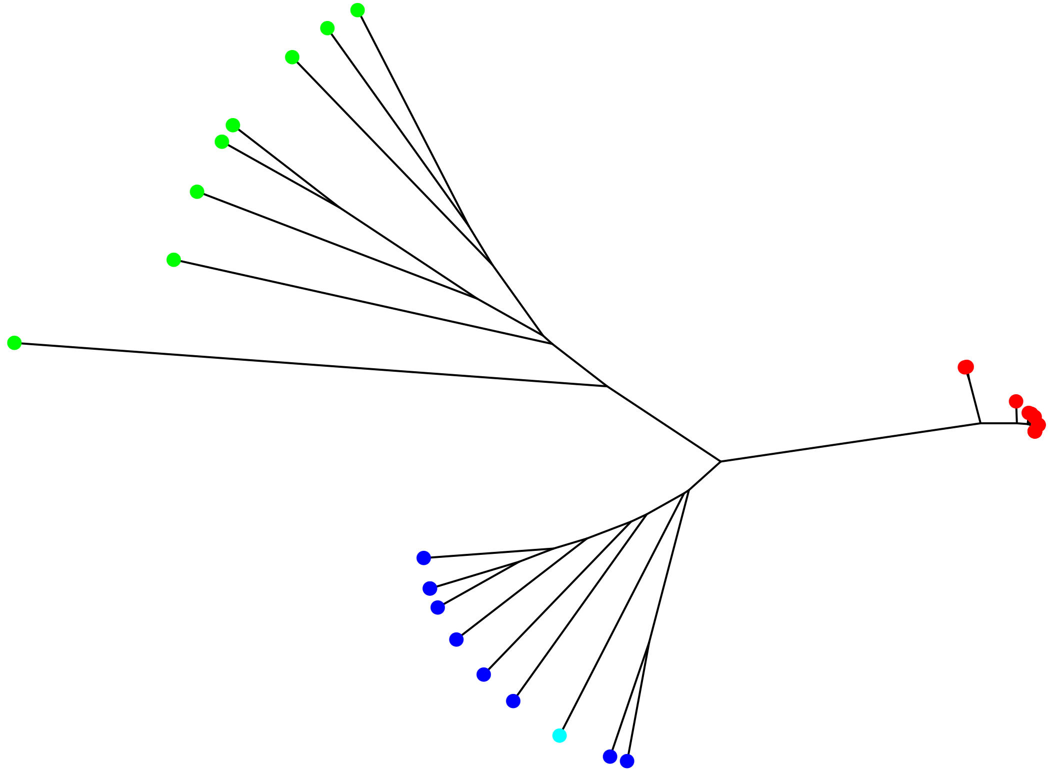

Gene family 908 : NADH−quinone oxidoreductase subunit I

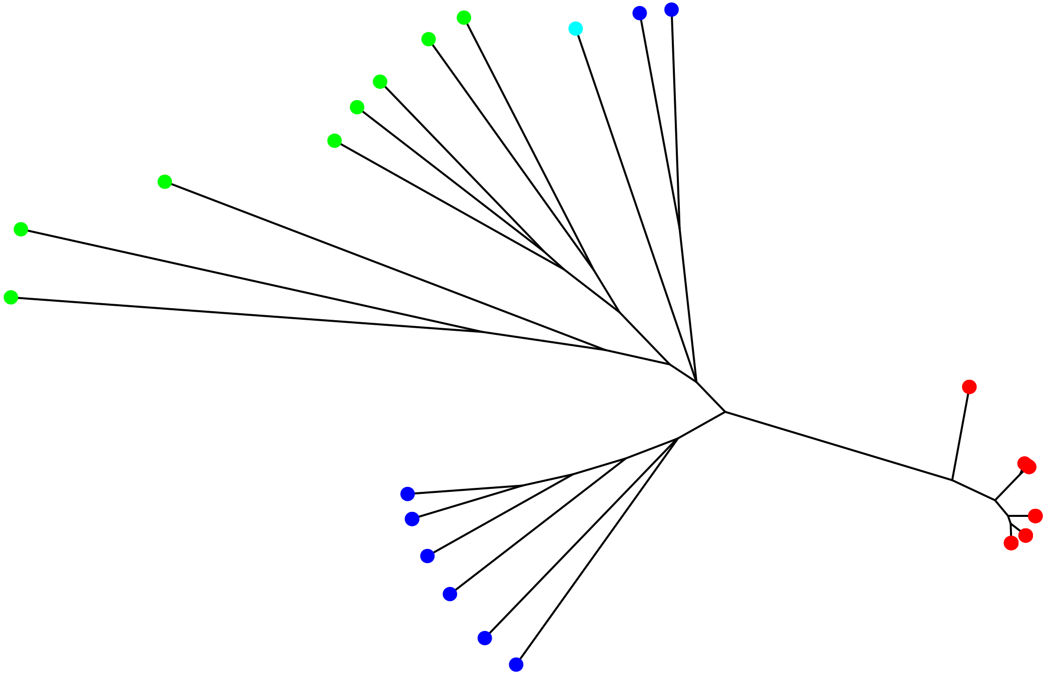

Gene family 911 : NADH−quinone oxidoreductase subunit F

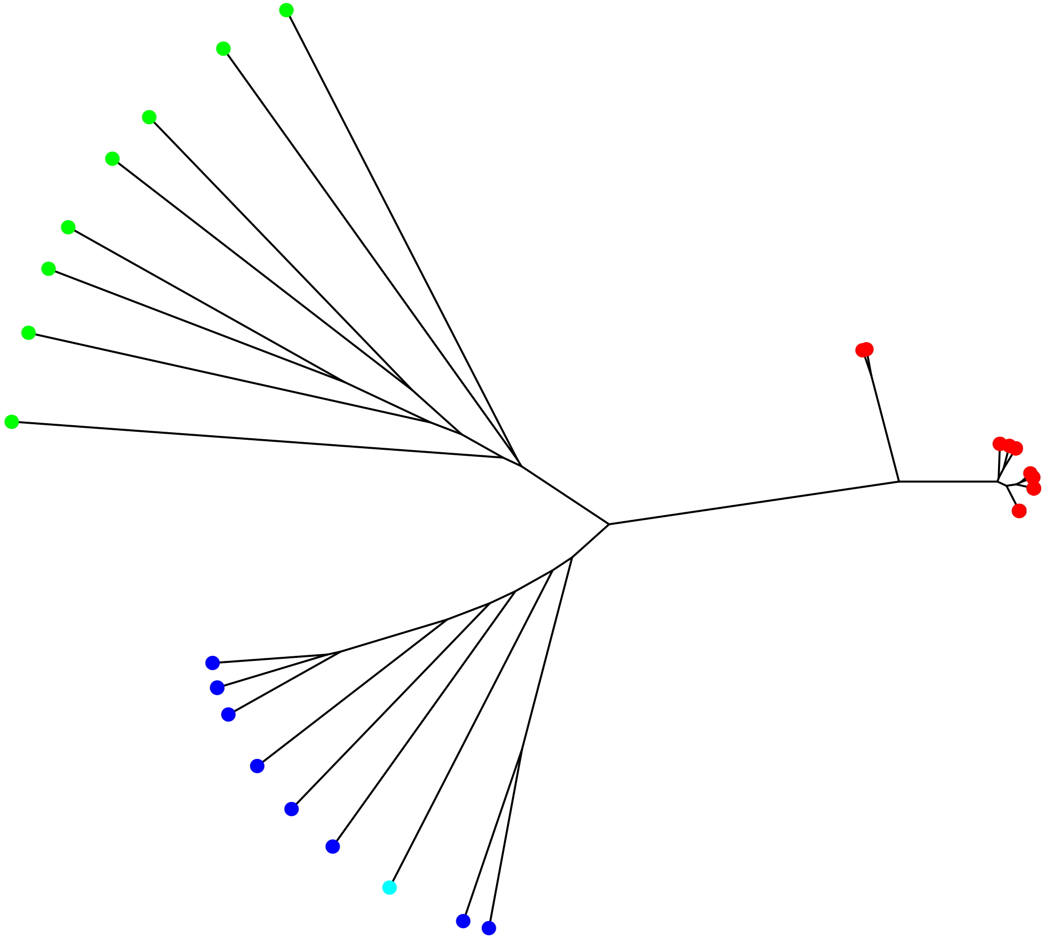

Gene family 915 : NAD(P)H−quinone oxidoreductase subunit 3

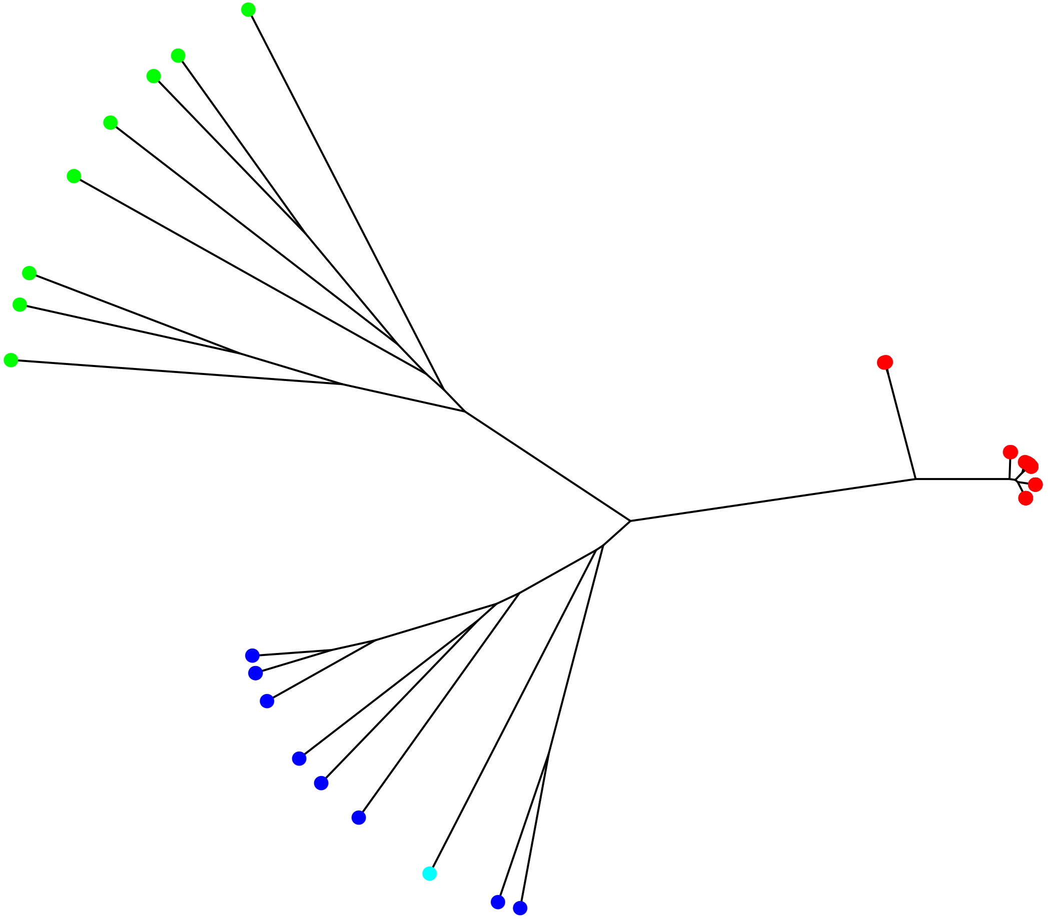

Gene family 930 : hypothetical protein

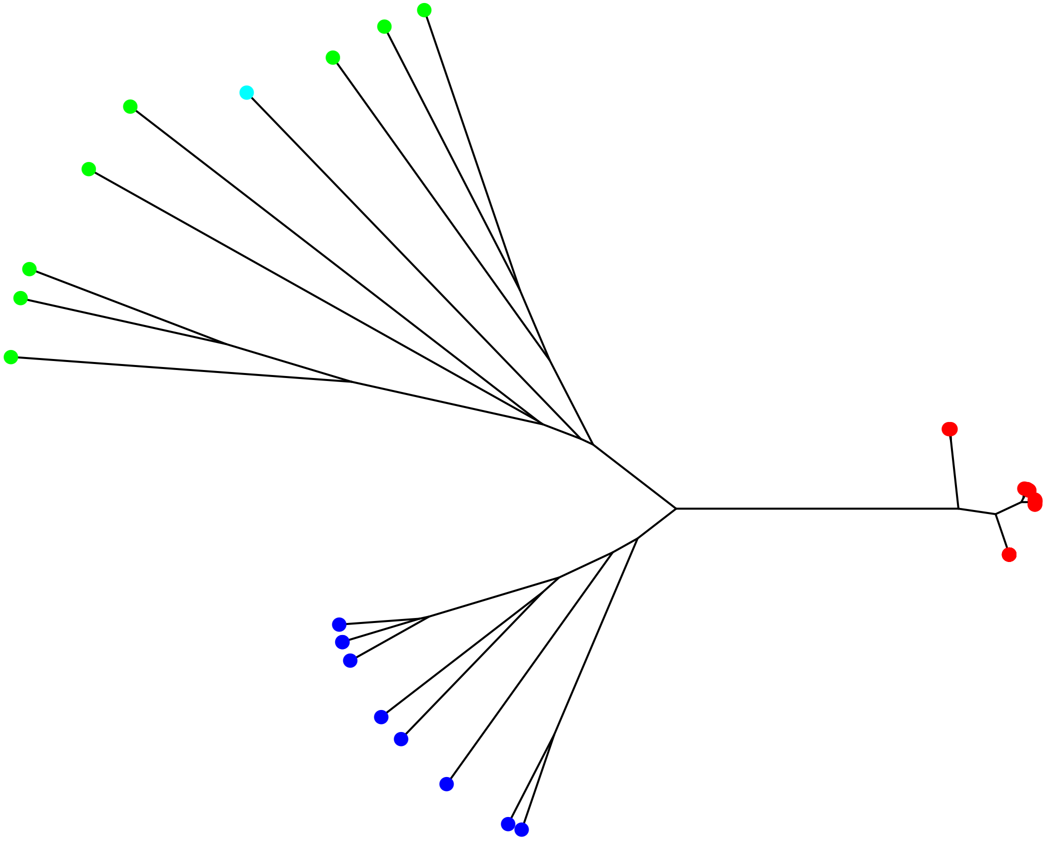

Gene family 966 : branched−chain alpha−keto acid dehydrogenase subunit E2

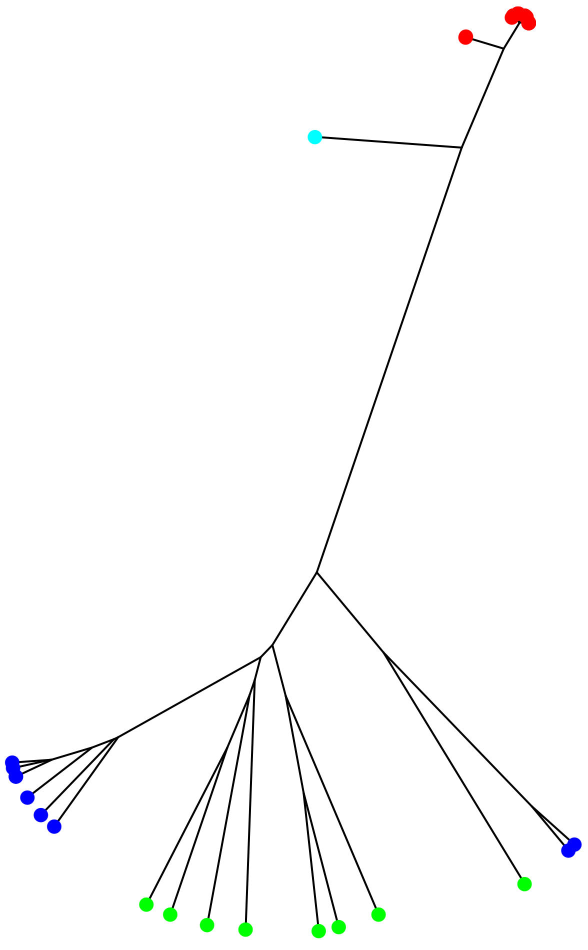

Gene family 1011 : ethanolamine utilization protein EutJ

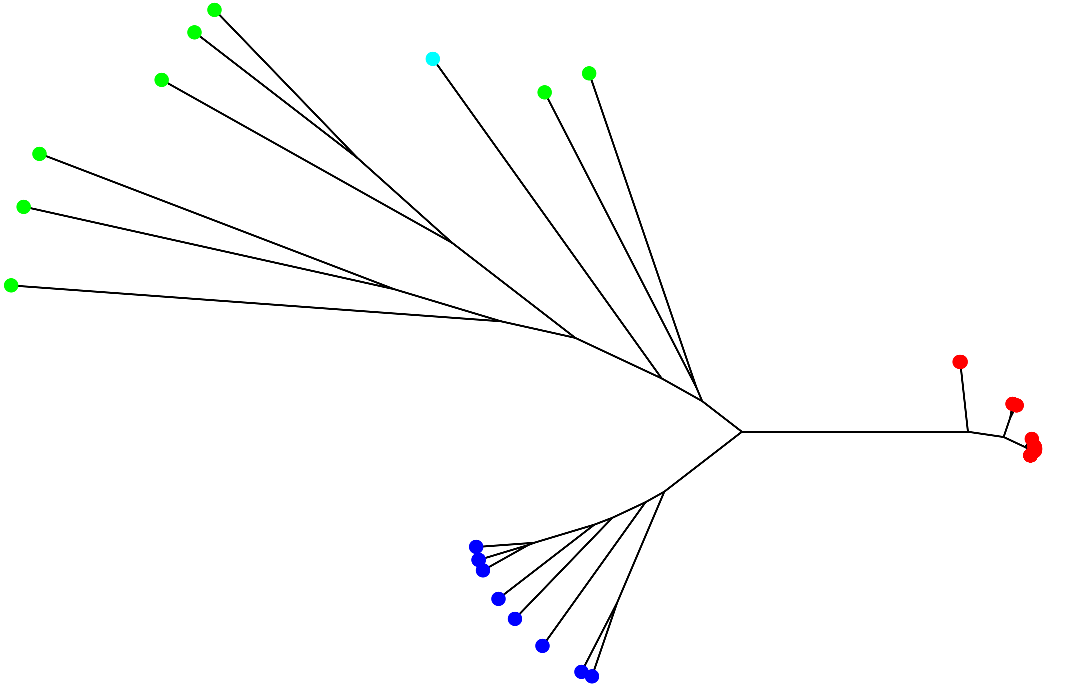

Gene family 1012 : Pilus assembly protein, PilO

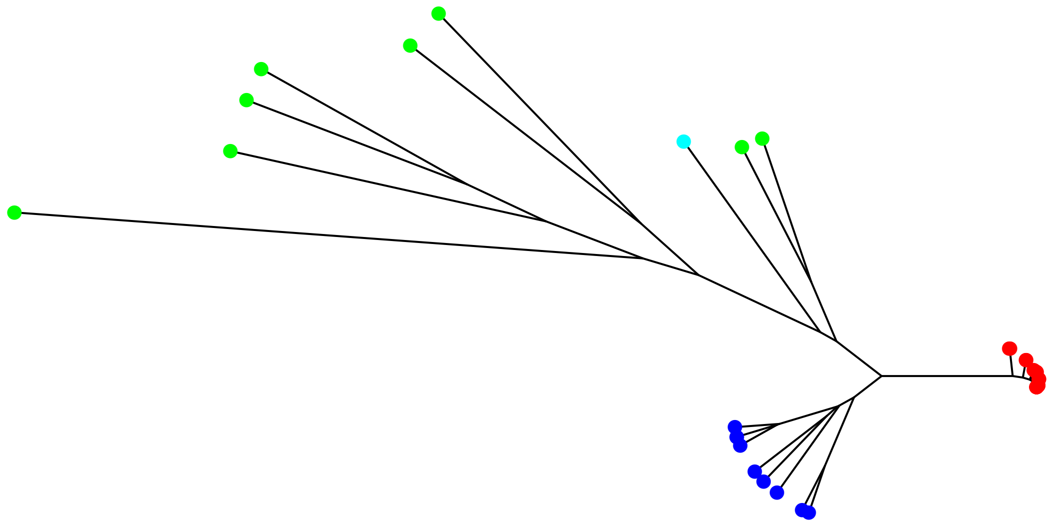

Gene family 1013 : Pilus assembly protein, PilP

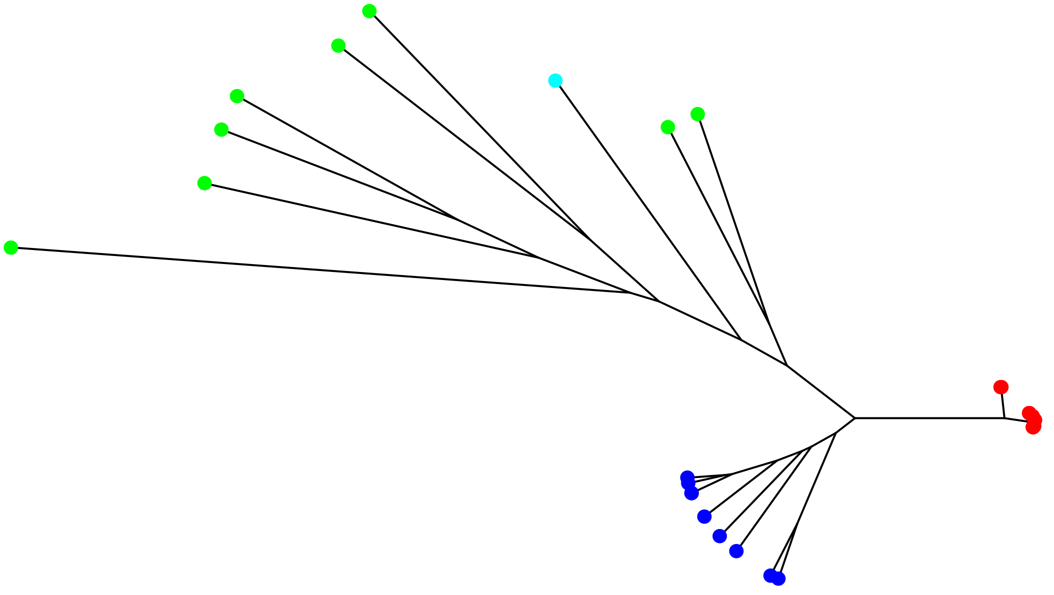

Gene family 1014 : Type IV pilus biogenesis and competence protein PilQ precursor

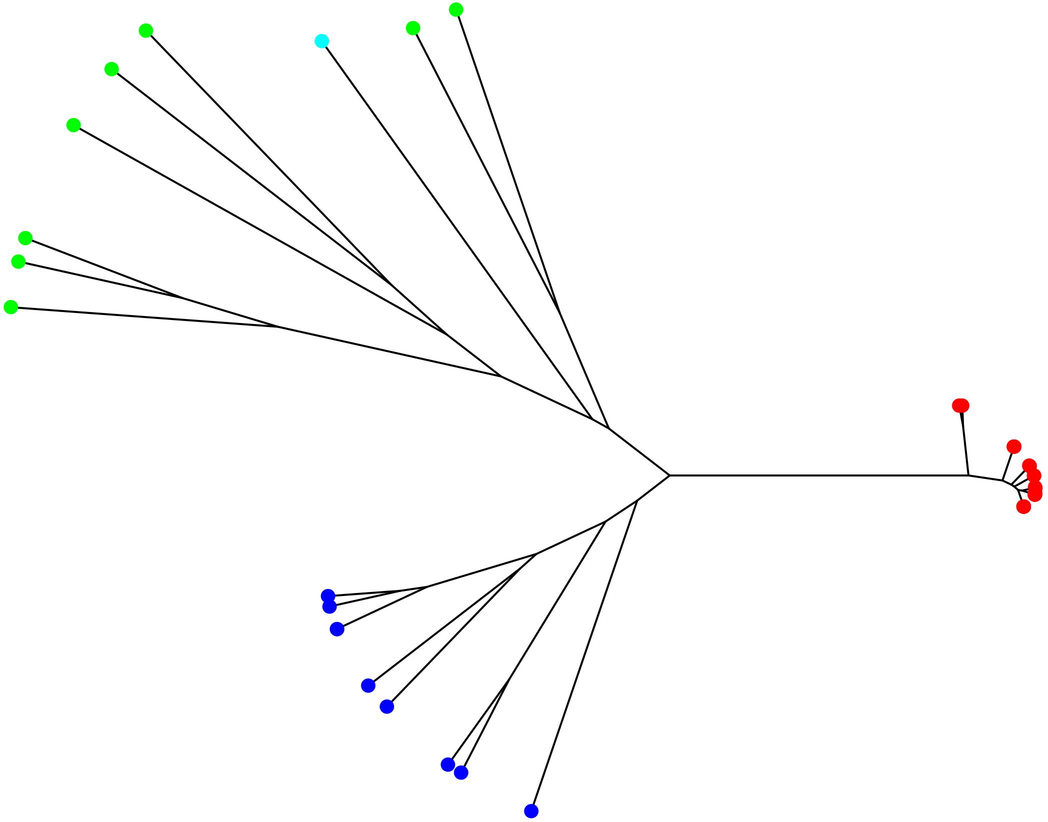

Gene family 1026 : pyridoxal phosphate enzyme, YggS family

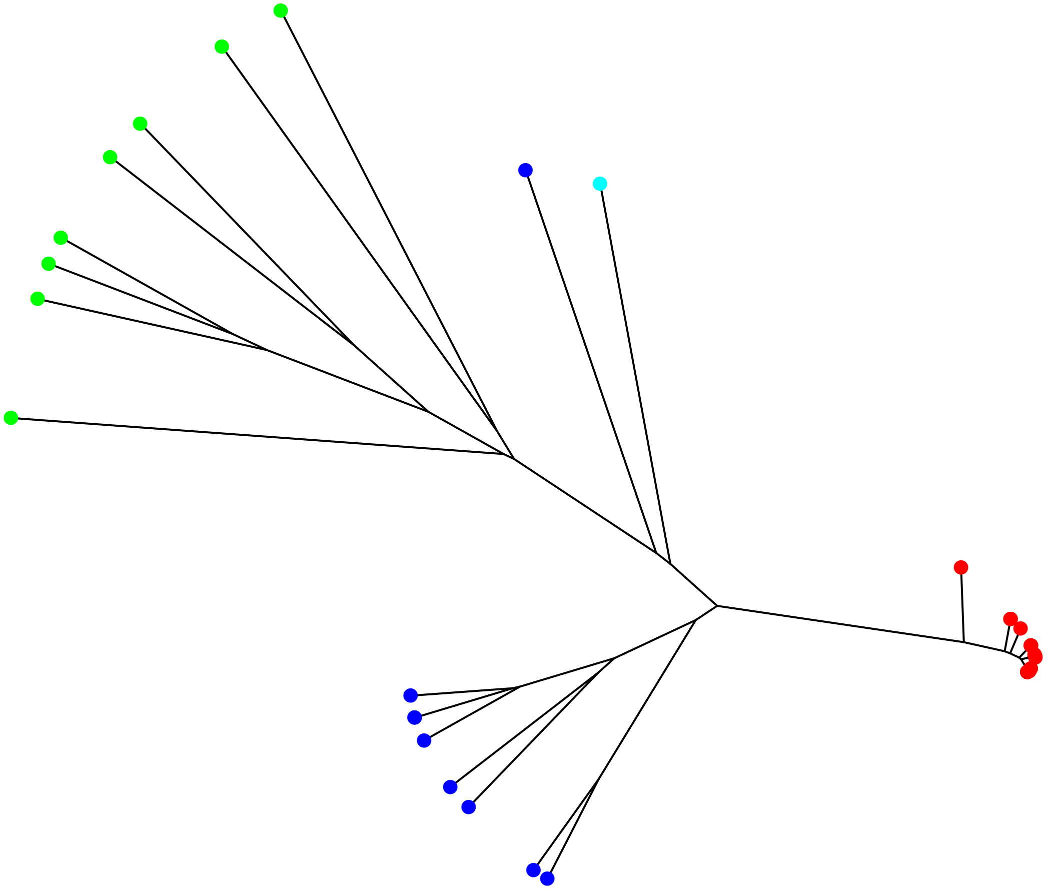

Gene family 1059 : lysozyme

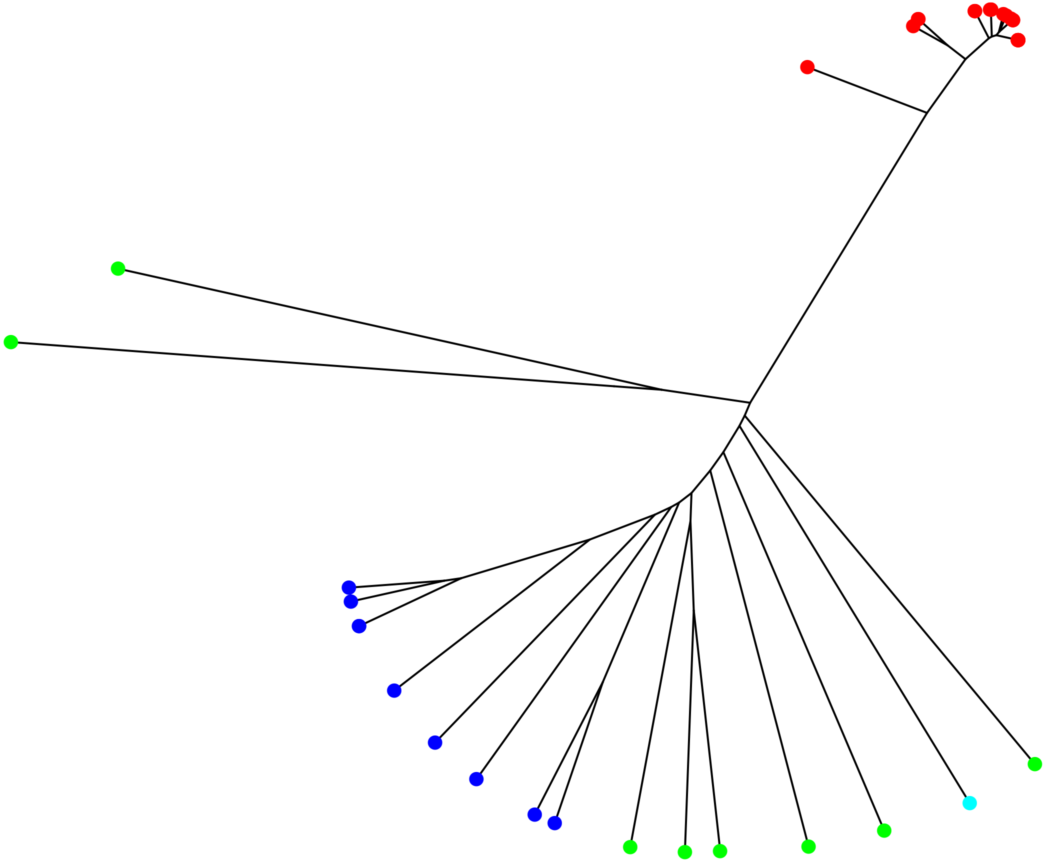

Gene family 1060 : hypothetical protein

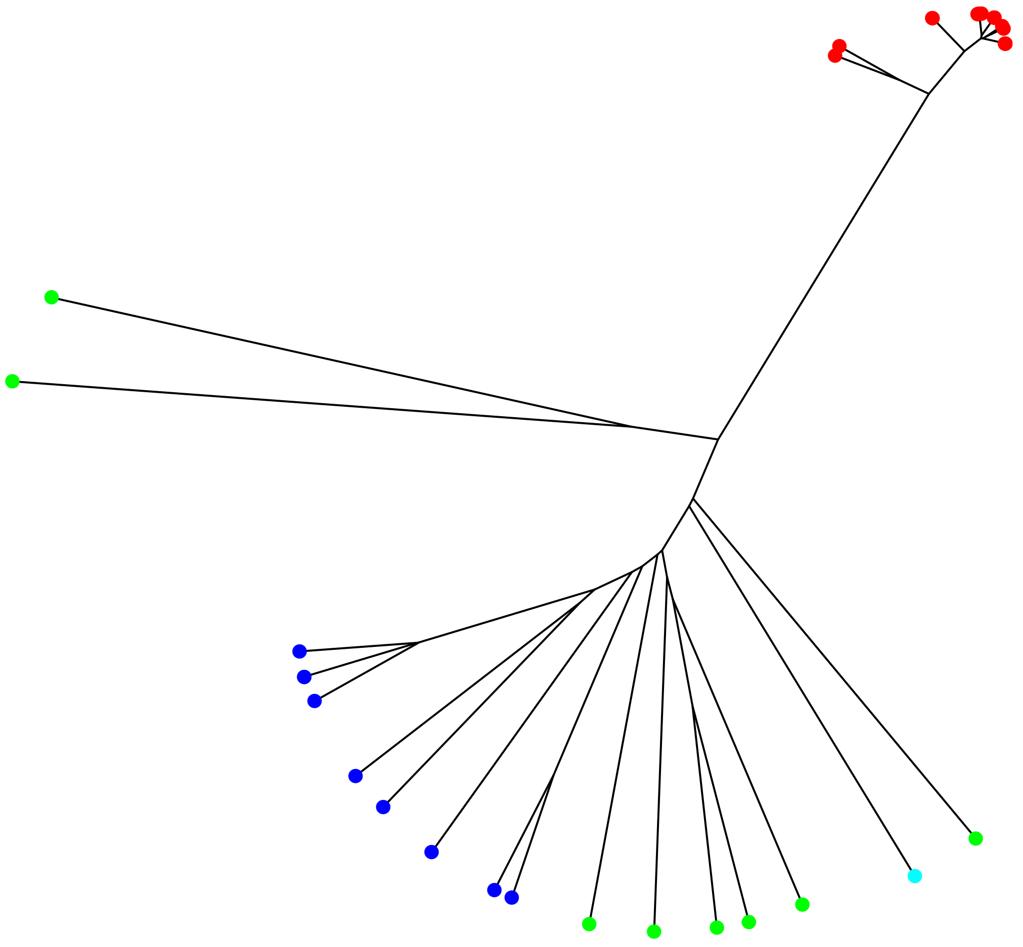

Gene family 1062 : hypothetical protein

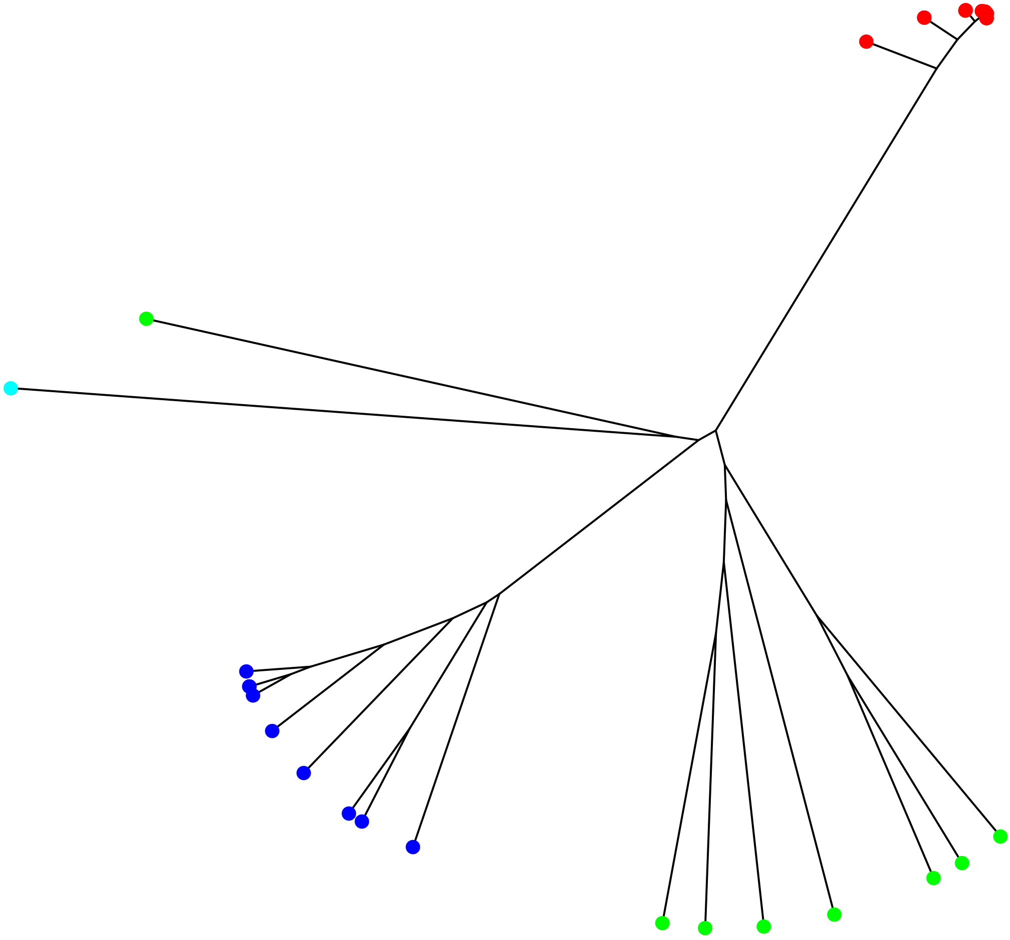

Gene family 1066 : hypothetical protein

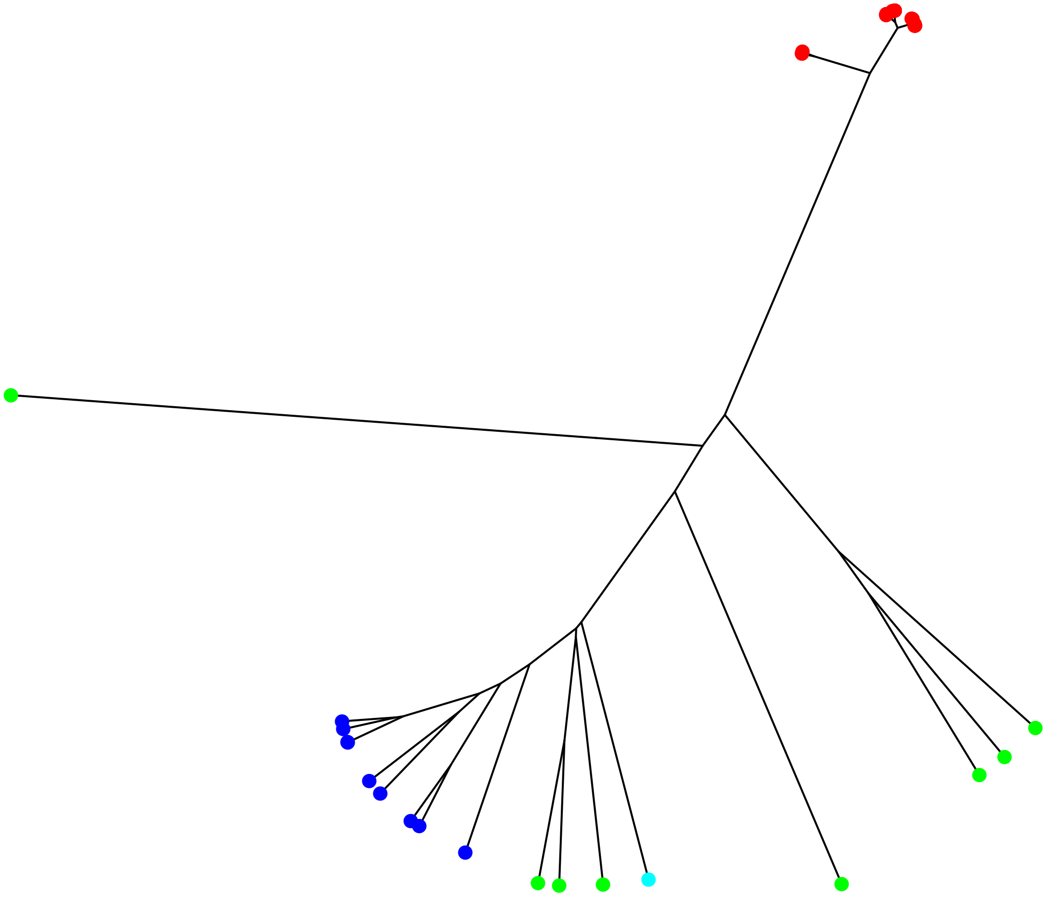

Gene family 1070 : Glutaminase

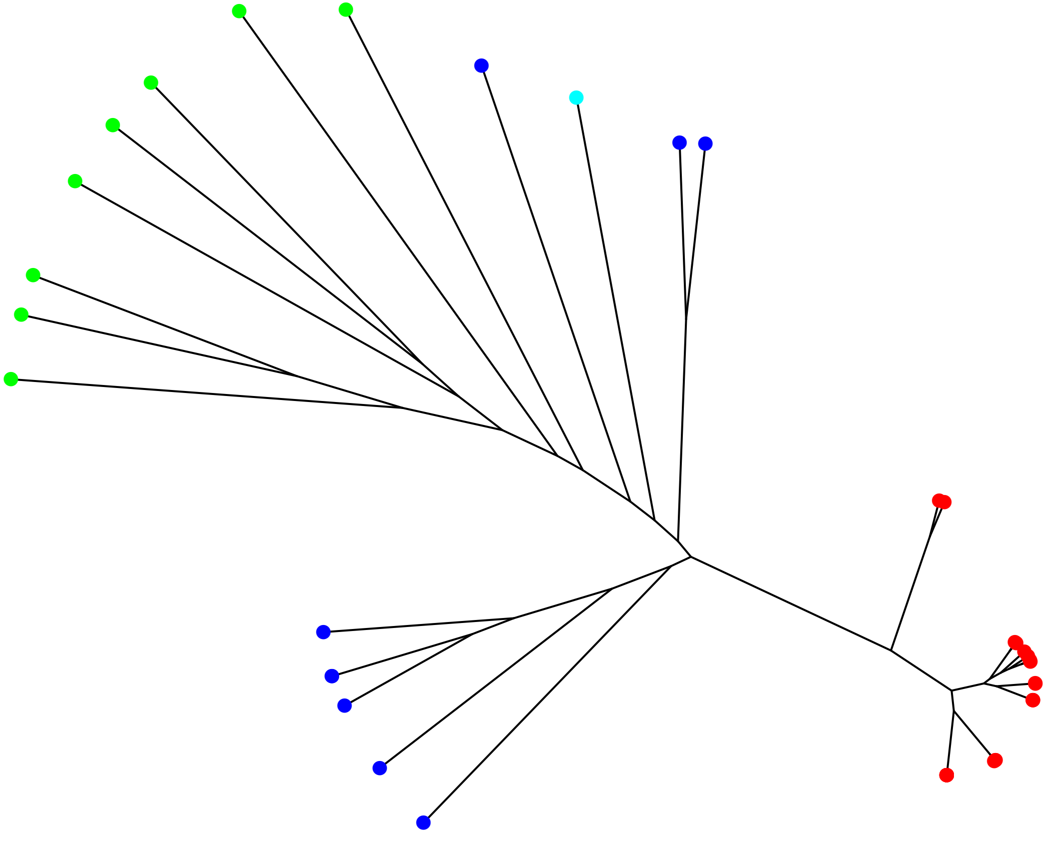

Gene family 1071 : Phenolic acid decarboxylase subunit B

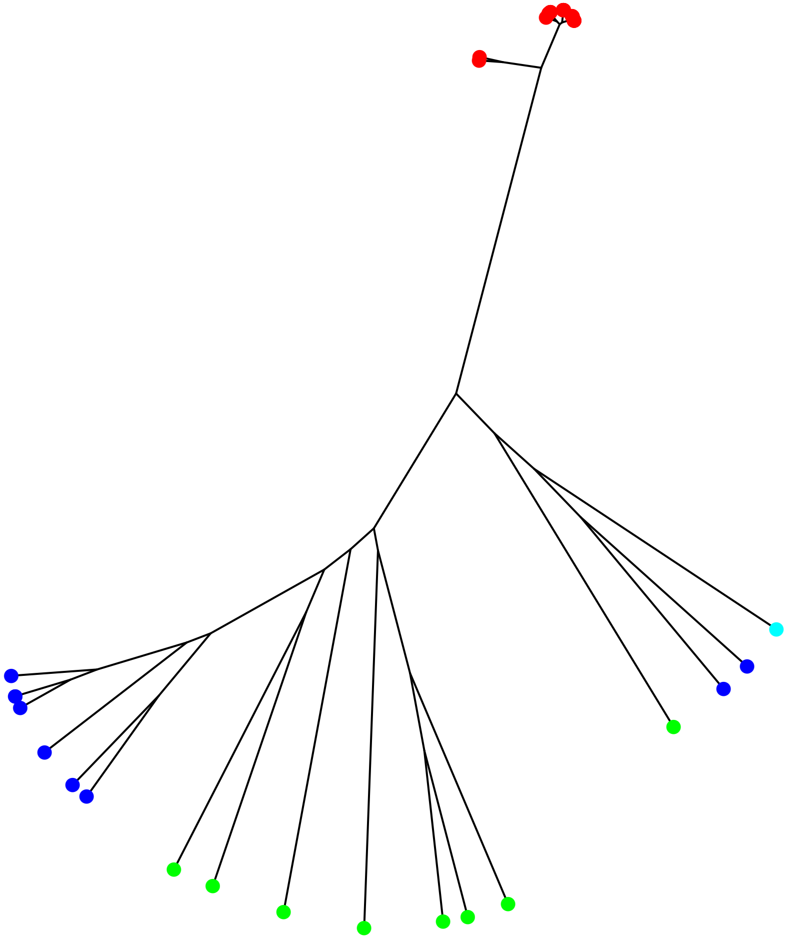

Gene family 1087 : hypothetical protein

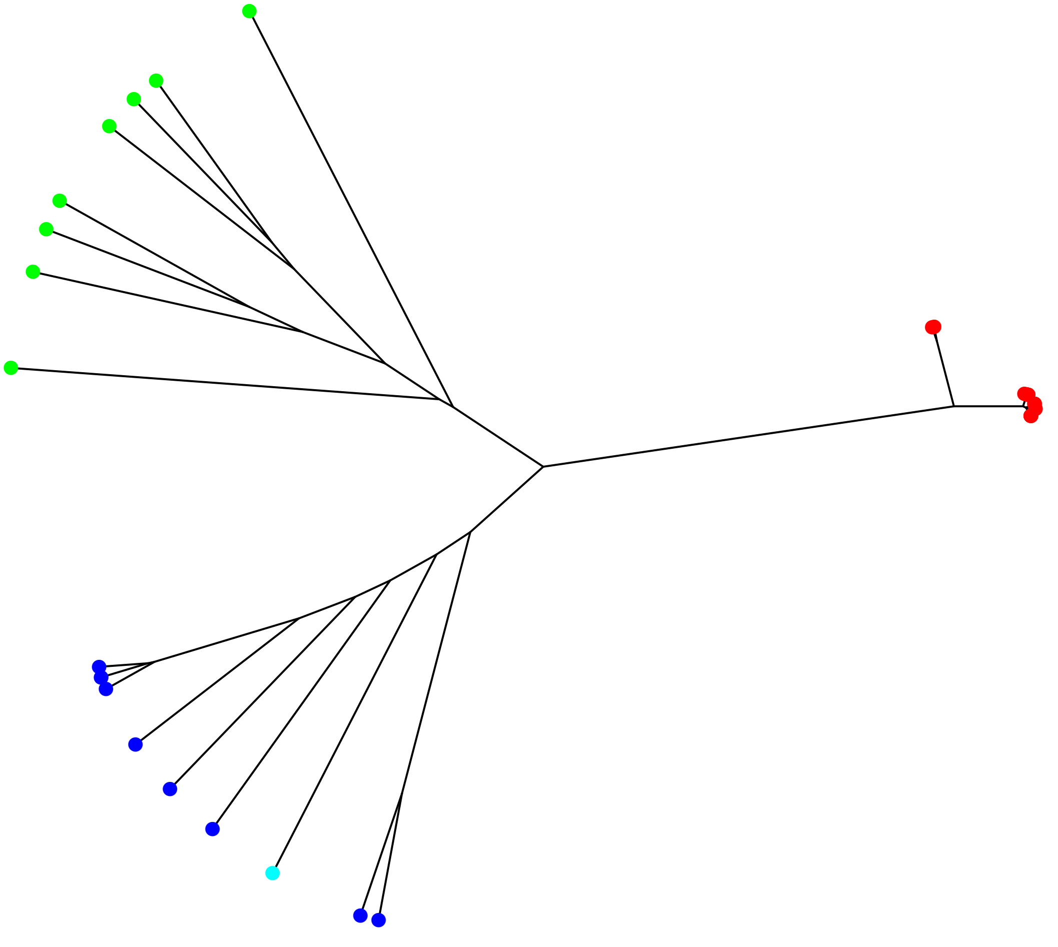

Gene family 1104 : hypothetical protein

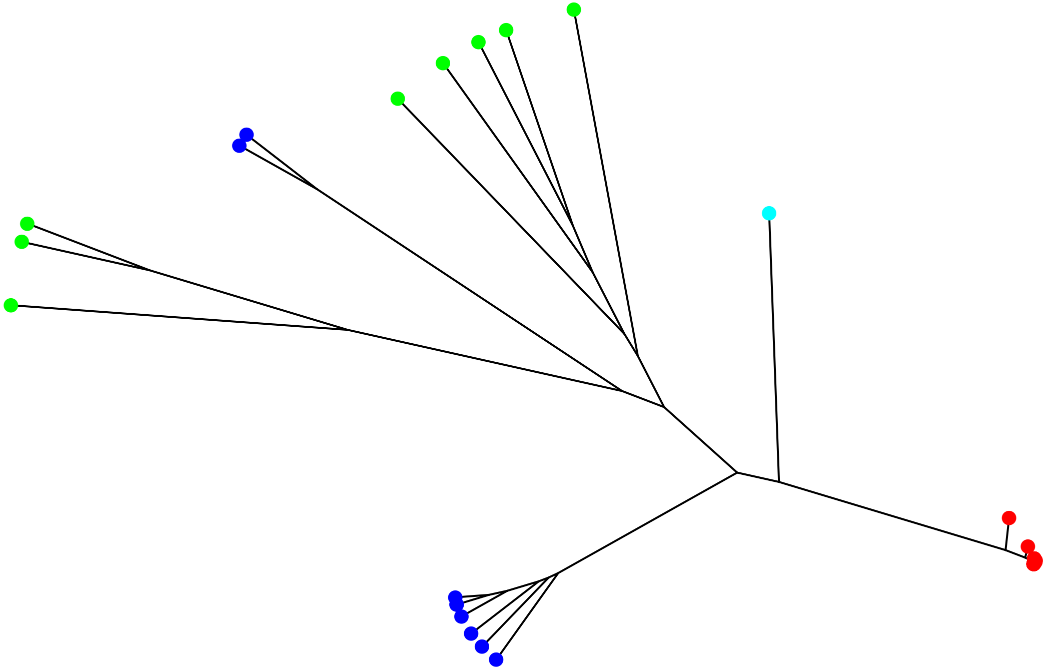

Gene family 1107 : Riboflavin synthase

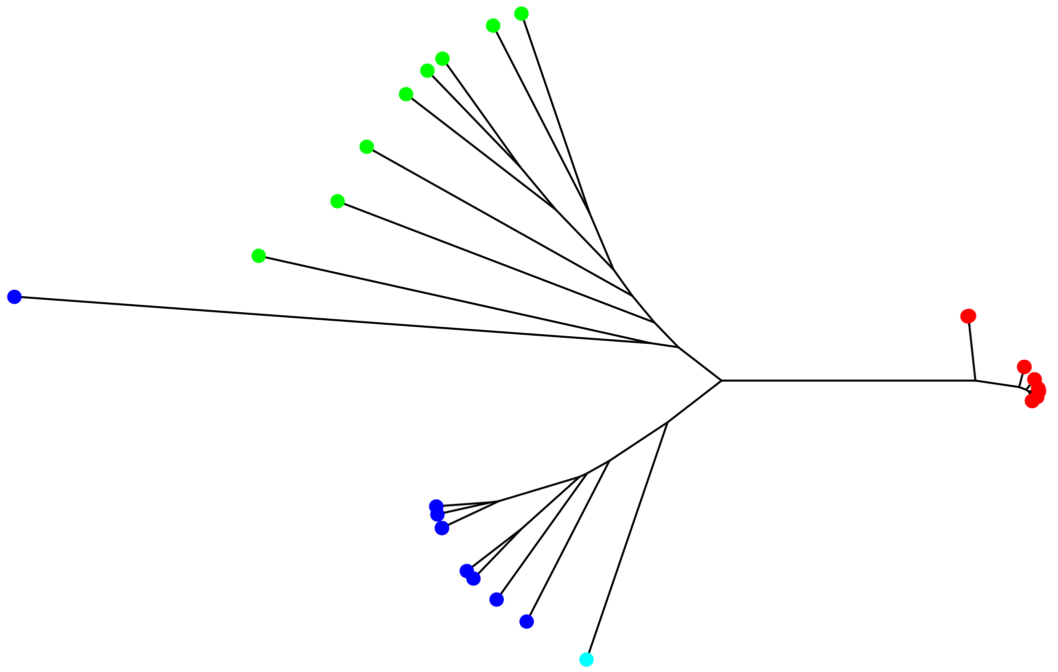

Gene family 1129 : queuosine biosynthesis protein QueD

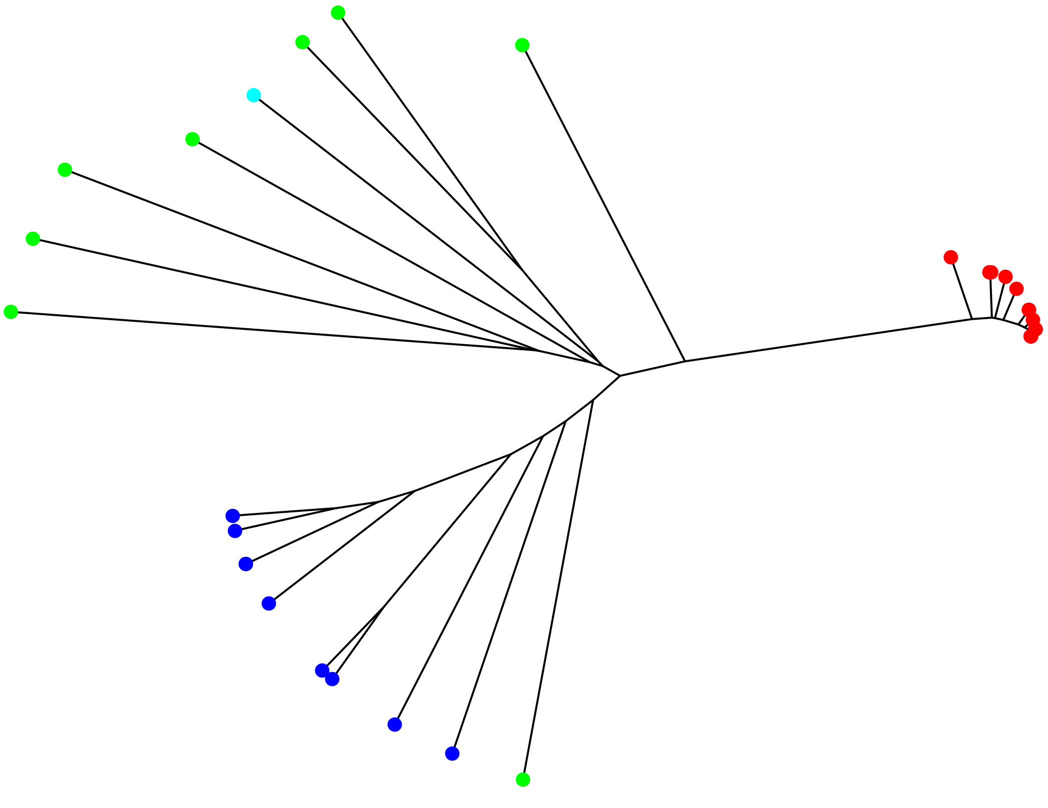

Gene family 1140 : Peptide chain release factor 1

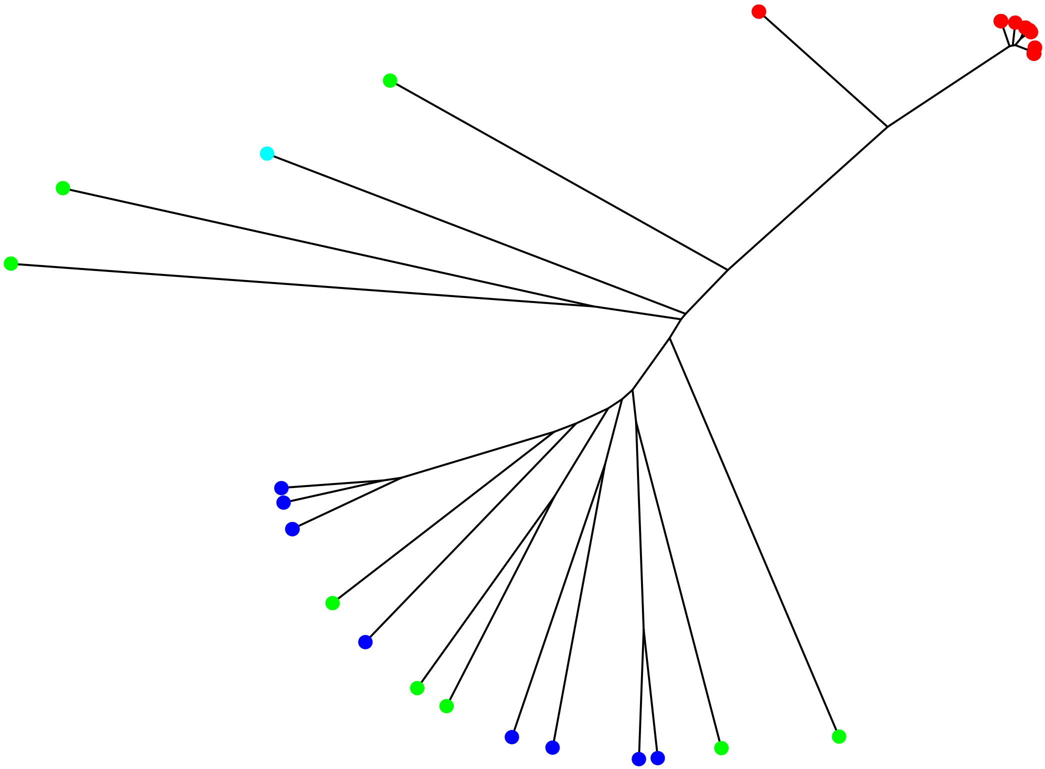

Gene family 1234 : Ribosomal RNA large subunit methyltransferase H

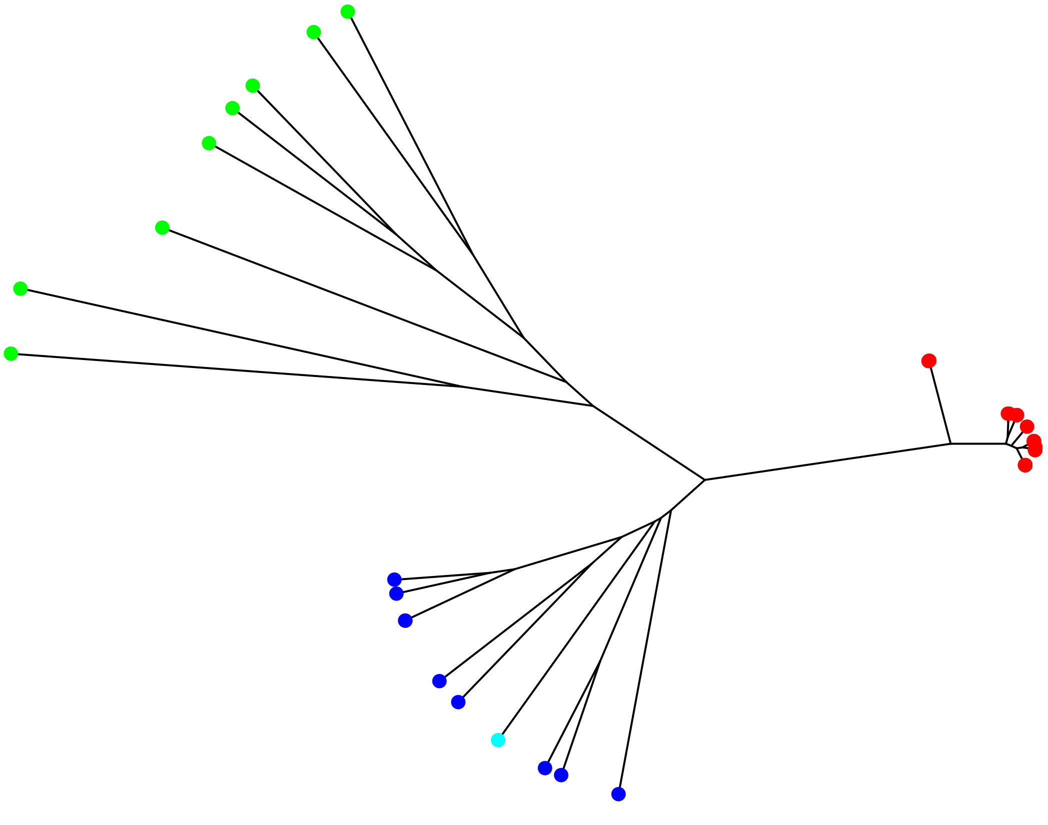

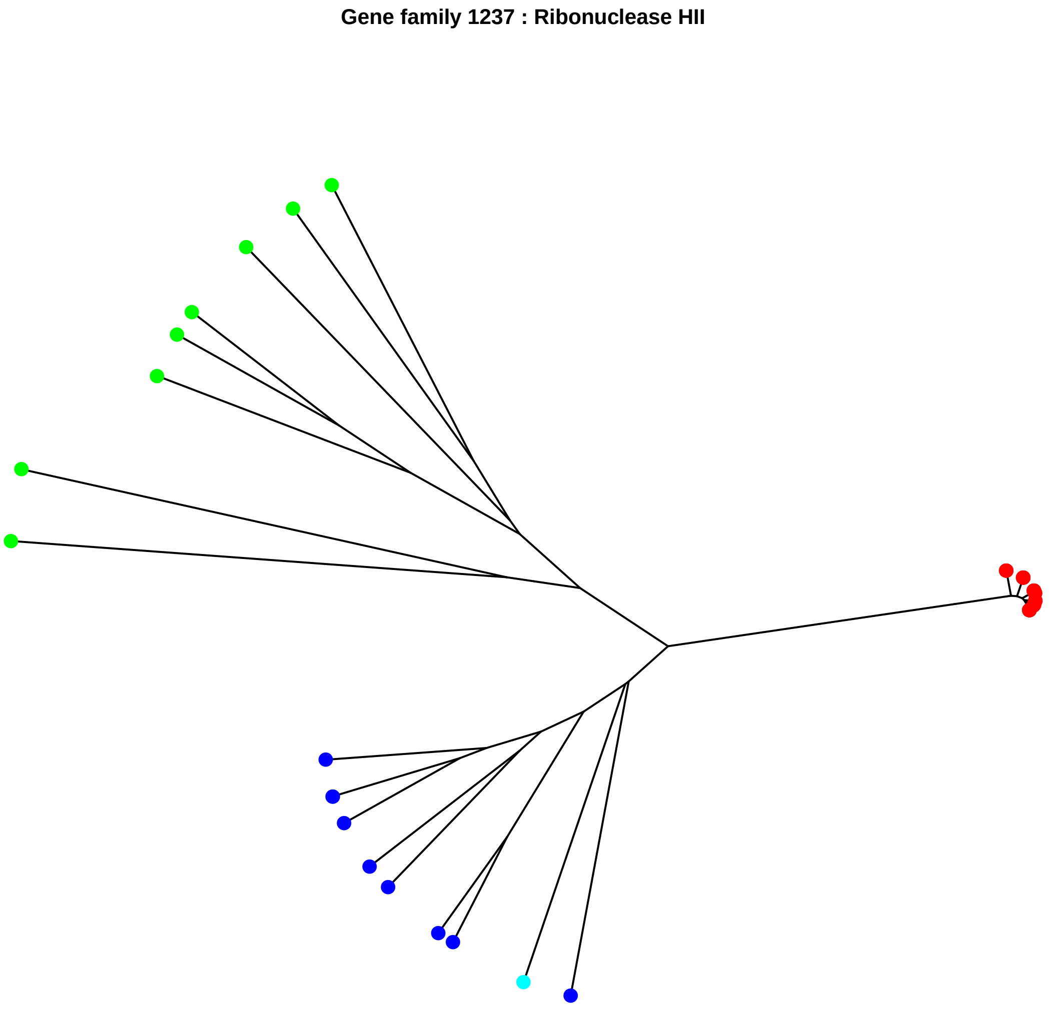

Gene family 1268 : Response regulator UvrY

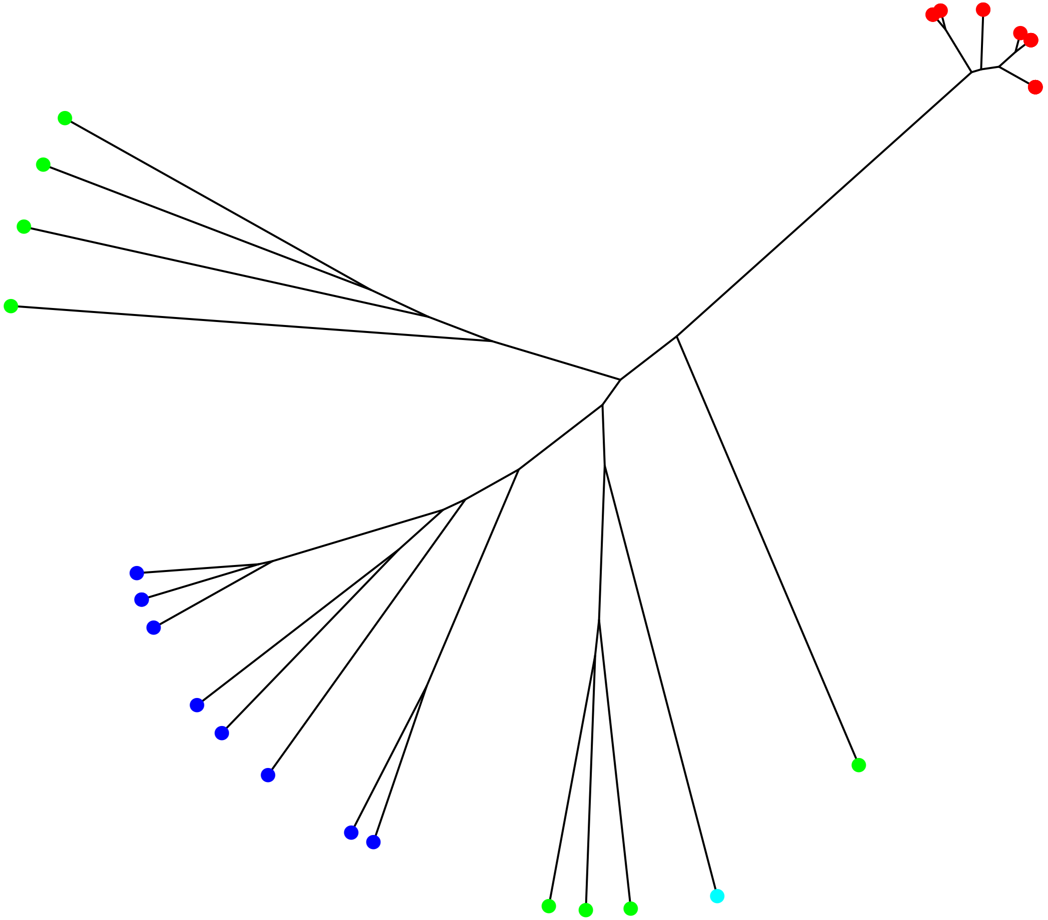

Gene family 1292 : polyhydroxyalkanoate synthesis repressor PhaR

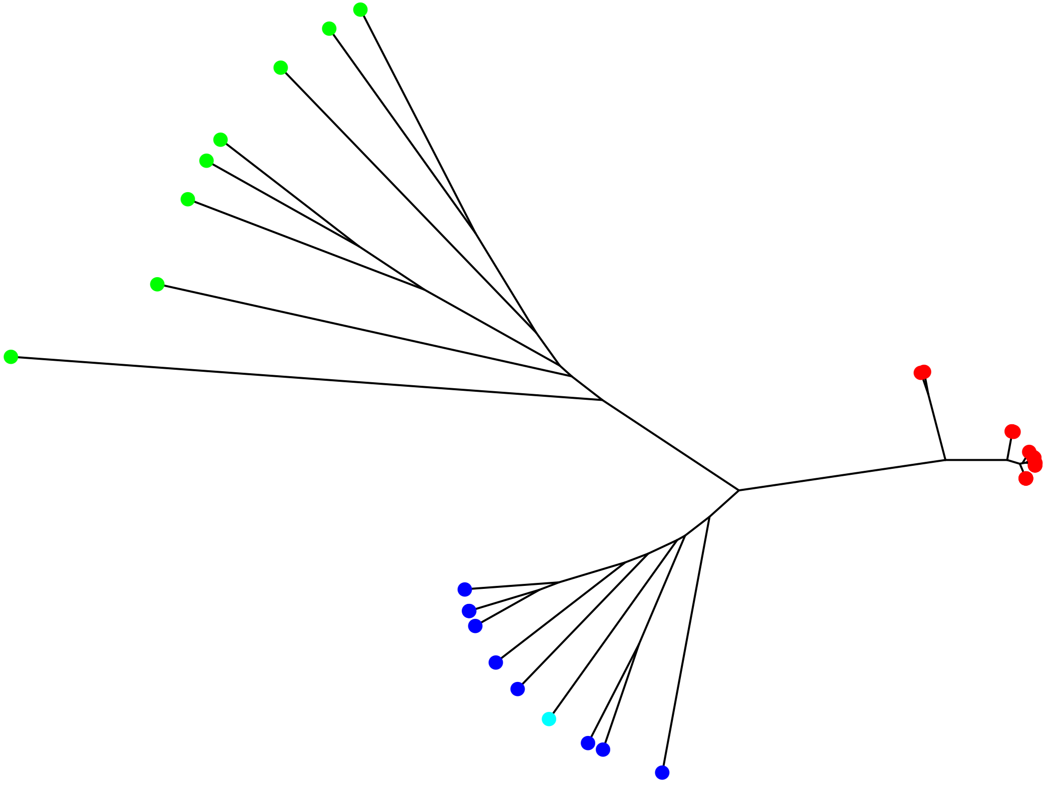

Gene family 1326 : Basal−body rod modification protein FlgD

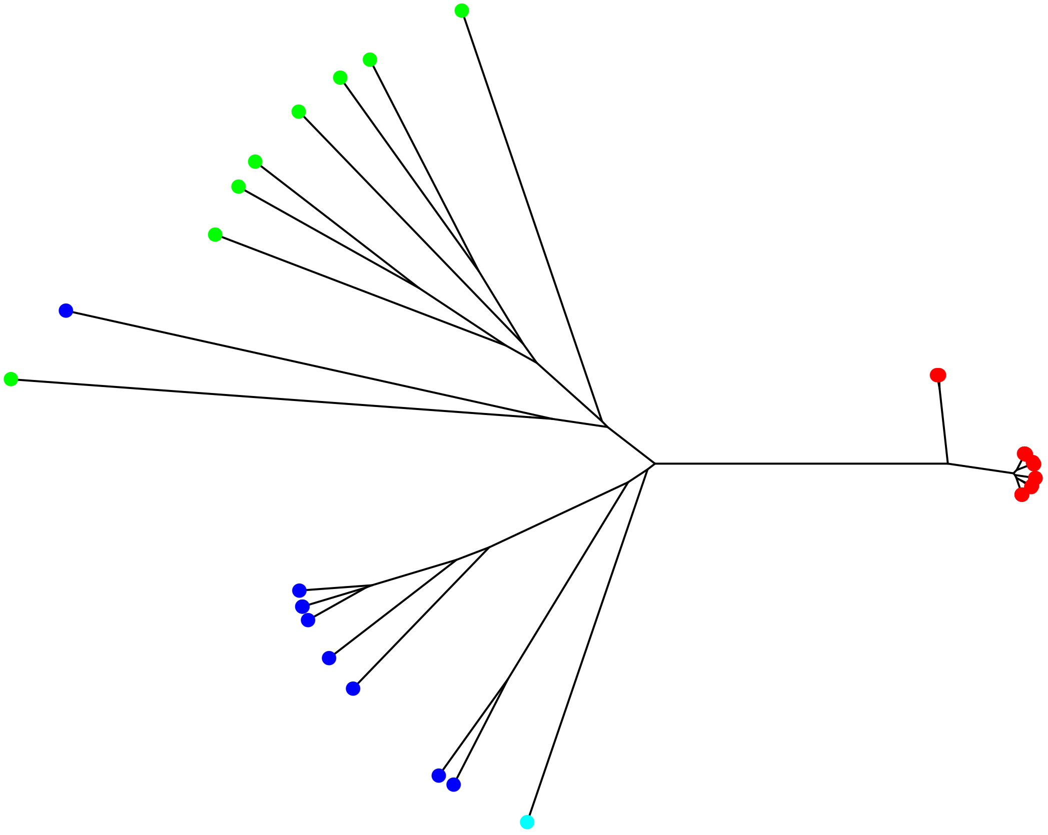

Gene family 1340 : 30S ribosomal protein S7

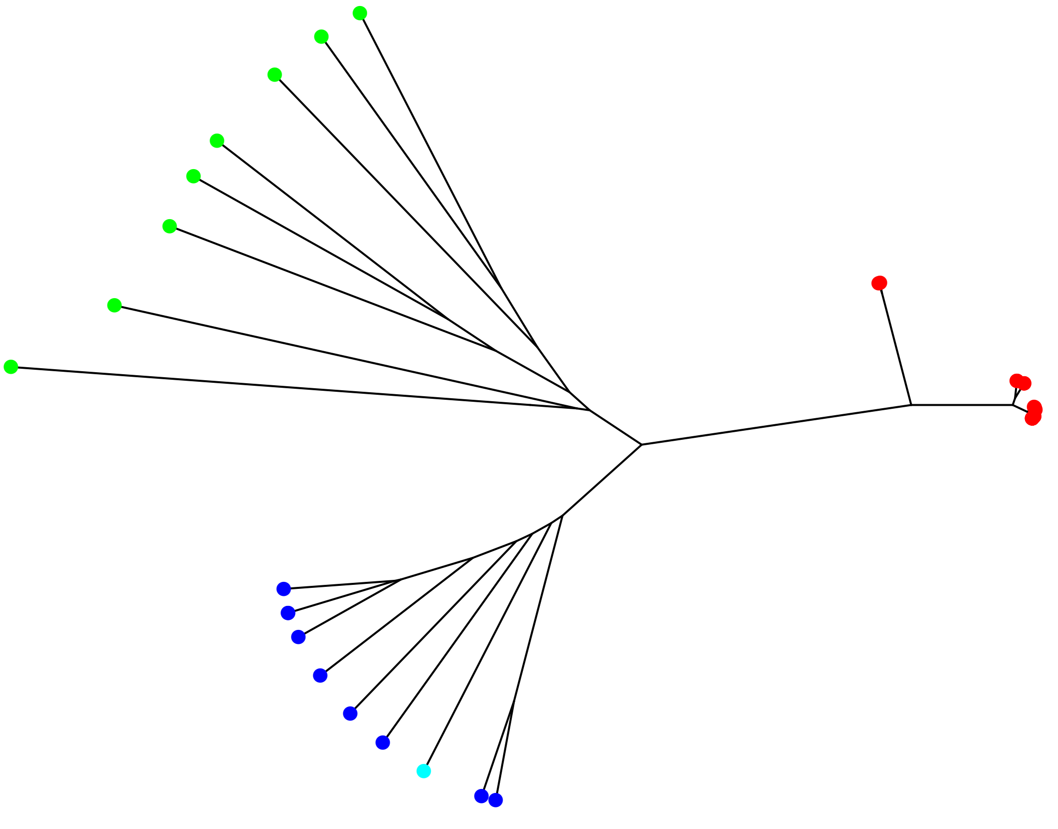

Gene family 1341 : 30S ribosomal protein S12

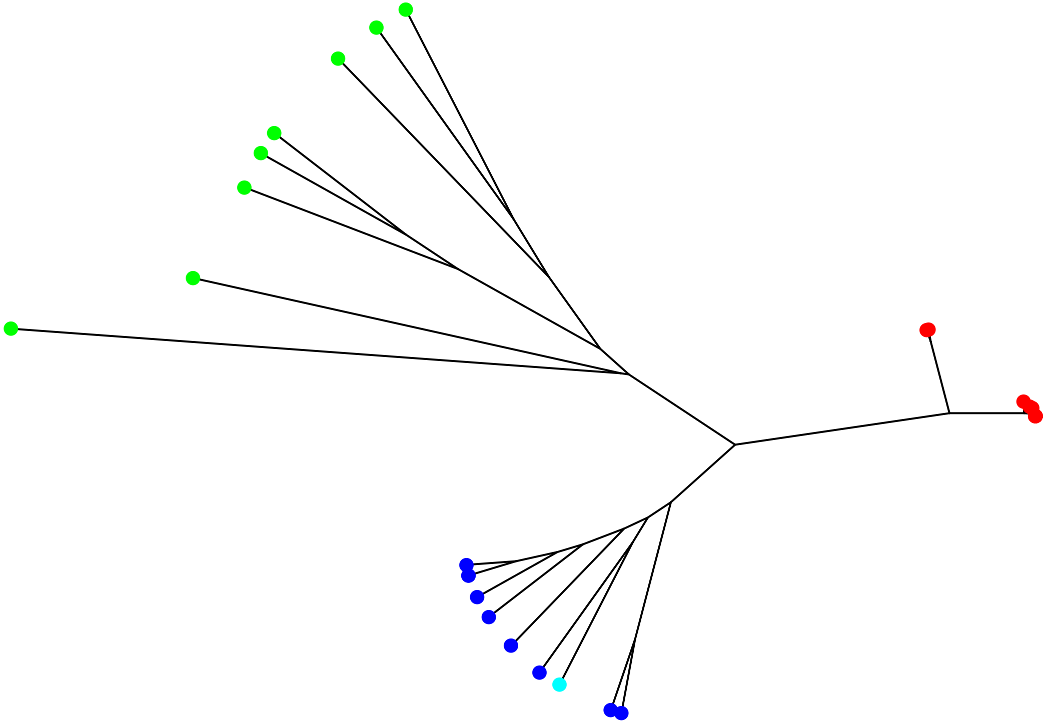

Gene family 1349 : acetoin dehydrogenase E2 subunit dihydrolipoyllysine−residue acetyltransferase

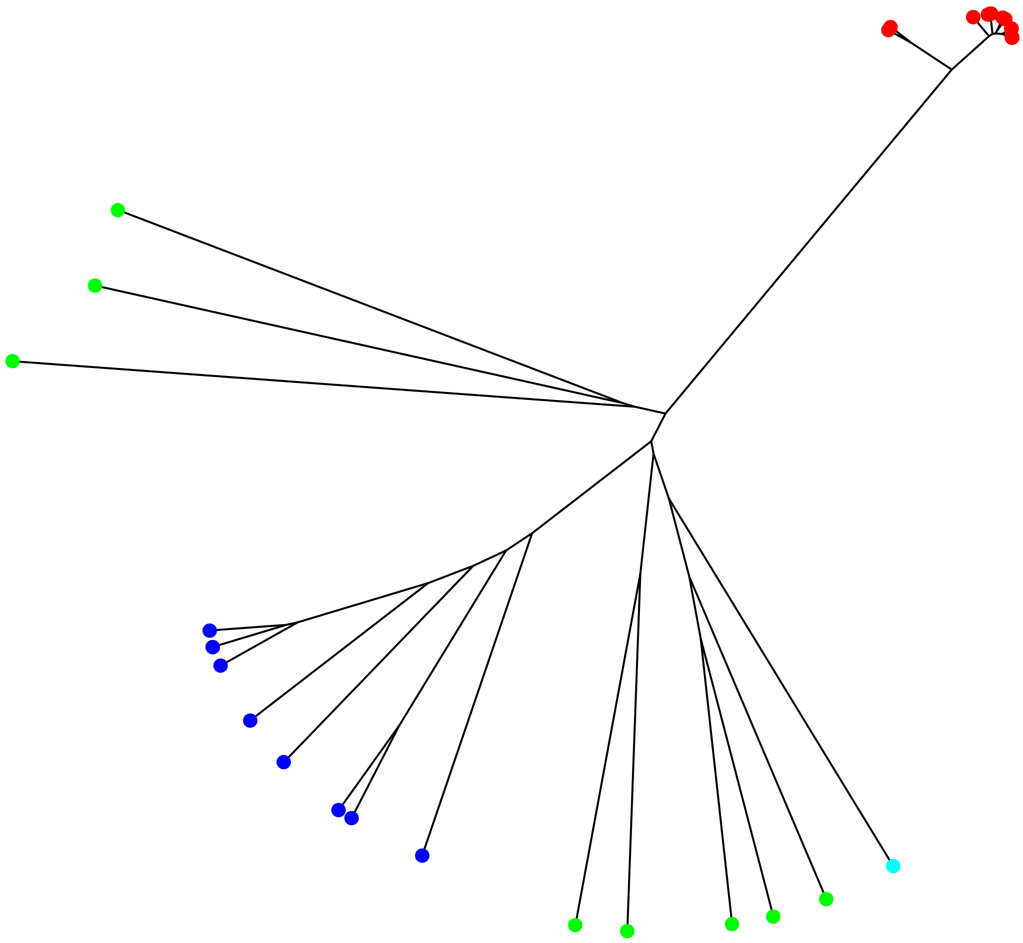

Gene family 1361 : Trp operon repressor

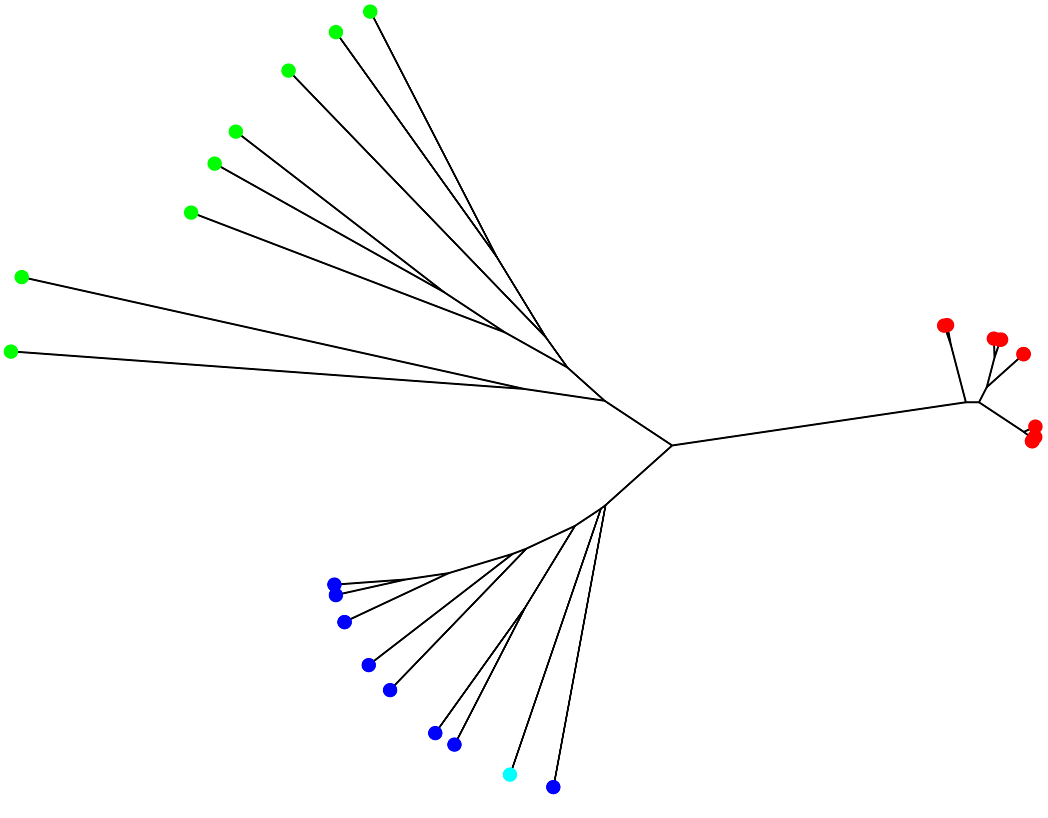

Supplementary Table 1. Characteristics of the *Legionella* genomes analyzed in this study.

Legionella species	Sg	No. of sequencing reads generated using both 454 and Illumina sequencing runs	No. of de novo contigs (> 500bp)	Largest Contig size in bp (number of reads used to form the contig)	Average GC%	No. of protein coding genes	No. of protein coding genes that were homologous to at least another *Legionella* gene	No. of unique genes present	Percentage of the protein coding genes that has at least one homologous *Legionella* gene	Clade	NCBI Accession Id
L. pneumophila - (Alcoy)	1	NA	NA	3516334*	38.38%	3127	3114	13	99.58%	1	NC_014125.1
L. pneumophila - (Corby)	1	NA	NA	3576470*	38.48%	3194	3177	17	99.46%	1	NC_009494.2
L. pneumophila - (HL06041035)	1	NA	NA	3492535*	38.35%	3113	3076	37	98.81%	1	FQ958211.1
L. pneumophila - (Lorraine)	1	NA	NA	3467254	38.36%	3130	3096	34	98.91%	1	FQ958210.1
L. pneumophila - (Paris)	1	NA	NA	3503610*	38.37%	3102	3064	38	98.77%	1	NC_006368.1
L. pneumophila - (Philadelphia)	1	NA	NA	3397754*	38.27%	3023	3020	3	99.90%	1	NC_002942.5
L. pneumophila - (Thunder Bay)	1	NA	NA	3455167*	38.23%	3116	3107	9	99.71%	1	NC_021350.1
L. pneumophila - (570-CO-H)	1	NA	NA	3359001	38.17%	2993	2993	0	100.00%	1	NC_016811.1
L. pneumophila - 2 (D5762)	*2*	12,644,433	77	216586 (10605)	38.36%	2986	2943	43	98.56%	1	
L. pneumophila - 3 (D5517)	*3*	12,539,147	40	439947 (28297)	37.67%	3041	3021	20	99.34%	1	
L. pneumophila - 4 (D5739)	*4*	9,010,736	85	175712 (27911)	37.71%	3143	3114	29	99.08%	1	
L. pneumophila - 5 [illegible]	*5*	13,708,986	34	697391 [illegible]	39.24%	3179	3149	30	99.06%	1	

(D5864)				(23676)							
L. pneumophila - 7 (D5178)	*7*	12,647,927	39	490823 (51022)	39.95%	3153	3085	68	97.84%	1	
L. pneumophila - 8 (D5744)	*8*	11,778,210	111	175201 (8012)	38.25%	3063	3021	42	98.63%	1	
L. pneumophila - 9 (D4366)	*9*	16,329,031	41	509167 (42,345)	39.86%	3051	3016	35	98.85%	1	
L. pneumophila - 10 (D5602)	*10*	3,637,672	83	369702 (33773)	38.04%	3181	3149	32	98.99%	1	
L. pneumophila - 11 (D797)	*11*	3,385,529	103	314201 (29554)	37.09%	3181	3118	63	98.02%	1	
L. pneumophila - 12 (D4955)	*12*	14,002,235	24	1178075 (119543)	40.60%	3049	3047	2	99.93%	1	
L. pneumophila - 13 (D5677)	*13*	10,606,713	101	273447 (27231)	39.00%	3104	3044	60	98.07%	1	
L. pneumophila - 14 (D4677)	*14*	11,255,691	165	130823 (8183)	37.18%	3200	3114	86	97.31%	1	
L. pneumophila - 15 (D4613)	*15*	12,443,216	57	248203 (9743)	38.56%	3059	3000	59	98.07%	1	
L. pneumophila - 16 (D5564)	*16*	2,475,679	124	109095 (7298)	38.45%	3231	3198	33	98.98%	1	
L. pneumophila - 17 (D4954)	*17*	11,220,305	74	552082 (35772)	38.81%	3242	3167	75	97.69%	1	
L. birminghamensis (D1407)		2,994,685	125	250412 (11618)	41.33%	3323	2874	449	86.49%	2	NC_0138 61.1
L. brunensis (D1635)		6,955,499	50	378315 (40895)	40.12%	3,312	3060	252	92.39%	2	
L. erythra (D3308)		4,650,940	150	267820 (15787)	41.99%	3279	2902	377	88.50%	2	
L. jamestowniensis (D4855)		9,436,629	36	719971 (58065)	39.63%	3322	3017	305	90.82%	2	
L. jordanis (D5875)		10,165,832	70	462056 (50345)	40.10%	2,961	2764	197	93.35%	2	
L. maceachernii (D5800)		12,176,672	86	261428 (19345)	40.04%	3,336	2939	397	88.10%	2	
L. micdadei (D5727)		44,586,161	43	261827 (34089)	40.94%	2,878	2752	126	95.62%	2	
L. nautarum (D4144)		7,059,586	75	284471 (36359)	39.53%	3,378	3044	334	90.11%	2	

L. anisa (D5641)		12,440,367	116	221876 (27235)	0.3914	3127	3114	13	99.58%	3	
L. bozemanii - 1 (D5751)	*1*	4,889,018	119	205860 (6389)	37.84%	3,628	3507	121	96.66%	3	
L. bozemanii - 2 (D4398)	2	4,373,003	90	265582 (38368)	37.60%	3,449	3399	50	98.55%	3	
L. cherii (D3084)		10,677,467	57	312463 (27548)	39.26%	3,223	3007	216	93.30%	3	
L. cincinnatiensis (D3325)		3,332,320	146	175844 (7158)	36.50%	3,501	3212	289	91.75%	3	
L. tusconensis (D1087)		8,211,209	31	703194 (48019)	38.64%	2999	2845	154	94.86%	3	
L. longbeachae (NSW150)		NA	NA	4077332*	37.11%	3473	3202	271	92.19%	3	NC_0138 61.1
L. dumoffii (D5637)		6,280,579	19	566306 (24927)	41.38%	3,446	3313	133	96.14%	3	
L. wadsworthii (D4735)		10,208,601	29	625783 (45679)	40.24%	3336	3150	186	94.42%	3	
L. rowbothamii (D5054)		13,499,246	55	1131520 (92829)	39.86%	3,559	3052	507	85.75%	4	

Supplementary Table 2. **GO term enrichment analysis of the core and accessory genomes identified in *Legionella* species.** (**a**) Results of the GO term enrichment analysis of the core genes. Performed hypergeometric probability distribution Test. (**b**) Results of the GO term enrichment analysis of all the accessory genes. Performed hypergeometric probability distribution Test.

Supplemtary Table 2a. Results of the GO term enrichment analysis of the core genes. Performed hypergeometric probability distribution Test

GOID	Ontology	Term	Level	q	m	t	k	log_odds_ratio	p
GO:0003674	molecular _function	molecular_function	0	853	5235	12977	1140	0.891279282	6.33E-133
GO:0003824	molecular _function	catalytic activity	1	641	3902	12977	1140	0.903033657	9.63E-81
GO:0016740	molecular _function	transferase activity	2	209	1400	12977	1140	0.764999193	1.41E-14
GO:0006139	biological	nucleobase-containing	4	237	1118	12977	1140	1.270889949	9.38E-40

	_process	compound metabolic process							
GO:0006213	biological _process	pyrimidine nucleoside metabolic process	7	11	23	12977	1140	2.444720835	7.15E-06
GO:0006220	biological _process	pyrimidine nucleotide metabolic process	8	16	26	12977	1140	2.808411455	2.89E-10
GO:0006221	biological _process	pyrimidine nucleotide biosynthetic process	9	15	24	12977	1140	2.830779268	8.21E-10
GO:0006241	biological _process	CTP biosynthetic process	11	4	7	12977	1140	2.701496251	0.005535782
GO:0006725	biological _process	cellular aromatic compound metabolic process	3	286	1244	12977	1140	1.387951739	5.32E-57
GO:0006753	biological _process	nucleoside phosphate metabolic process	6	59	189	12977	1140	1.829251798	1.73E-17
GO:0006793	biological _process	phosphorus metabolic process	3	114	592	12977	1140	1.132287821	4.38E-15
GO:0006796	biological _process	phosphate-containing compound metabolic process	4	113	588	12977	1140	1.12935779	6.82E-15
GO:0006807	biological _process	nitrogen compound metabolic process	2	352	1493	12977	1140	1.424284341	9.01E-76
GO:0008150	biological _process	biological_process	0	896	5467	12977	1140	0.899672435	3.15E-149
GO:0008152	biological _process	metabolic process	1	774	4493	12977	1140	0.971577582	4.66E-125
GO:0009058	biological _process	biosynthetic process	2	341	1051	12977	1140	1.884932148	4.57E-116
GO:0009116	biological _process	nucleoside metabolic process	6	46	123	12977	1140	2.089898623	3.95E-17
GO:0009117	biological _process	nucleotide metabolic process	7	57	184	12977	1140	1.818179231	9.53E-17
GO:0009119	biological _process	ribonucleoside metabolic process	7	41	102	12977	1140	2.193977835	1.24E-16
GO:0009141	biological _process	nucleoside triphosphate metabolic process	7	17	49	12977	1140	1.98160417	2.88E-06
GO:0009142	biological	nucleoside triphosphate	8	15	41	12977	1140	2.058189764	5.84E-06

	_process	biosynthetic process							
GO:0009147	biological _process	pyrimidine nucleoside triphosphate metabolic process	8	8	12	12977	1140	2.923888672	7.52E-06
GO:0009148	biological _process	pyrimidine nucleoside triphosphate biosynthetic process	9	7	11	12977	1140	2.856774476	5.19E-05
GO:0009163	biological _process	nucleoside biosynthetic process	7	35	76	12977	1140	2.390206676	1.69E-16
GO:0009165	biological _process	nucleotide biosynthetic process	8	47	133	12977	1140	2.008157589	2.13E-16
GO:0009199	biological _process	ribonucleoside triphosphate metabolic process	8	12	43	12977	1140	1.667548919	0.000975868
GO:0009201	biological _process	ribonucleoside triphosphate biosynthetic process	9	12	37	12977	1140	1.884360308	0.000218952
GO:0009208	biological _process	pyrimidine ribonucleoside triphosphate metabolic process	9	4	7	12977	1140	2.701496251	0.005535782
GO:0009209	biological _process	pyrimidine ribonucleoside triphosphate biosynthetic process	10	4	7	12977	1140	2.701496251	0.005535782
GO:0009218	biological _process	pyrimidine ribonucleotide metabolic process	9	12	19	12977	1140	2.84588616	5.17E-08
GO:0009220	biological _process	pyrimidine ribonucleotide biosynthetic process	10	12	19	12977	1140	2.84588616	5.17E-08
GO:0009259	biological _process	ribonucleotide metabolic process	8	35	109	12977	1140	1.869949865	5.65E-11
GO:0009260	biological _process	ribonucleotide biosynthetic process	9	32	87	12977	1140	2.065907677	7.32E-12
GO:0009987	biological _process	cellular process	1	628	2986	12977	1140	1.259473471	2.22E-132

GO:0018130	biological_process	heterocycle biosynthetic process	4	137	458	12977	1140	1.767679468	1.09E-38
GO:0019438	biological_process	aromatic compound biosynthetic process	4	132	426	12977	1140	1.818535672	4.79E-39
GO:0019637	biological_process	organophosphate metabolic process	4	85	255	12977	1140	1.923888672	1.89E-27
GO:0019693	biological_process	ribose phosphate metabolic process	5	37	111	12977	1140	1.923888672	4.09E-12
GO:0034641	biological_process	cellular nitrogen compound metabolic process	3	290	1271	12977	1140	1.377011948	3.88E-57
GO:0034654	biological_process	nucleobase-containing compound biosynthetic process	5	94	342	12977	1140	1.645587509	2.54E-23
GO:0042455	biological_process	ribonucleoside biosynthetic process	8	35	76	12977	1140	2.390206676	1.69E-16
GO:0044237	biological_process	cellular metabolic process	2	538	2220	12977	1140	1.463969574	8.85E-135
GO:0044238	biological_process	primary metabolic process	2	505	2261	12977	1140	1.346245474	1.37E-108
GO:0044249	biological_process	cellular biosynthetic process	3	321	891	12977	1140	2.035999038	4.73E-123
GO:0044271	biological_process	cellular nitrogen compound biosynthetic process	4	133	460	12977	1140	1.718643557	9.17E-36
GO:0044281	biological_process	small molecule metabolic process	3	216	658	12977	1140	1.901794901	8.08E-71
GO:0044710	biological_process	single-organism metabolic process	2	349	1380	12977	1140	1.525481847	4.86E-84
GO:0046036	biological_process	CTP metabolic process	10	4	7	12977	1140	2.701496251	0.005535782
GO:0046131	biological_process	pyrimidine ribonucleoside metabolic process	8	11	20	12977	1140	2.646354696	1.35E-06
GO:0046132	biological_process	pyrimidine ribonucleoside biosynthetic process	9	11	18	12977	1140	2.79835779	3.28E-07

GO:0046134	biological _process	pyrimidine nucleoside biosynthetic process	8	11	18	12977	1140	2.79835779	3.28E-07
GO:0046390	biological _process	ribose phosphate biosynthetic process	6	34	89	12977	1140	2.120580583	4.01E-13
GO:0046483	biological _process	heterocycle metabolic process	3	287	1260	12977	1140	1.374550081	2.54E-56
GO:0055086	biological _process	nucleobase-containing small molecule metabolic process	5	72	227	12977	1140	1.852227687	8.61E-22
GO:0071704	biological _process	organic substance metabolic process	2	558	2451	12977	1140	1.373817716	2.91E-128
GO:0072527	biological _process	pyrimidine-containing compound metabolic process	4	17	42	12977	1140	2.203996591	2.49E-07
GO:0072528	biological _process	pyrimidine-containing compound biosynthetic process	5	16	34	12977	1140	2.421388331	5.07E-08
GO:0090407	biological _process	organophosphate biosynthetic process	5	71	193	12977	1140	2.066141255	7.40E-26
GO:1901135	biological _process	carbohydrate derivative metabolic process	3	92	259	12977	1140	2.015604841	3.56E-32
GO:1901137	biological _process	carbohydrate derivative biosynthetic process	4	71	166	12977	1140	2.283558861	1.08E-30
GO:1901293	biological _process	nucleoside phosphate biosynthetic process	7	48	136	12977	1140	2.006350832	1.07E-16
GO:1901360	biological _process	organic cyclic compound metabolic process	3	297	1288	12977	1140	1.392253415	8.39E-60
GO:1901362	biological _process	organic cyclic compound biosynthetic process	4	144	476	12977	1140	1.783958411	2.33E-41
GO:1901564	biological _process	organonitrogen compound metabolic process	3	202	587	12977	1140	1.969845962	6.20E-70
GO:1901566	biological _process	organonitrogen compound biosynthetic process	4	134	359	12977	1140	2.087100329	4.38E-50
GO:1901576	biological _process	organic substance biosynthetic process	3	327	961	12977	1140	1.953605377	2.40E-117

GO:1901657	biological _process	glycosyl compound metabolic process	3	46	123	12977	1140	2.089898623	3.95E-17
GO:1901659	biological _process	glycosyl compound biosynthetic process	4	35	76	12977	1140	2.390206676	1.69E-16
GO:0006222	biological _process	UMP biosynthetic process	11	8	15	12977	1140	2.601960577	6.68E-05
GO:0009123	biological _process	nucleoside monophosphate metabolic process	7	19	47	12977	1140	2.202189834	4.41E-08
GO:0009124	biological _process	nucleoside monophosphate biosynthetic process	8	19	46	12977	1140	2.23321673	2.93E-08
GO:0009129	biological _process	pyrimidine nucleoside monophosphate metabolic process	8	11	18	12977	1140	2.79835779	3.28E-07
GO:0009130	biological _process	pyrimidine nucleoside monophosphate biosynthetic process	9	11	18	12977	1140	2.79835779	3.28E-07
GO:0009156	biological _process	ribonucleoside monophosphate biosynthetic process	9	16	42	12977	1140	2.11653375	1.52E-06
GO:0009161	biological _process	ribonucleoside monophosphate metabolic process	8	16	42	12977	1140	2.11653375	1.52E-06
GO:0009173	biological _process	pyrimidine ribonucleoside monophosphate metabolic process	9	8	15	12977	1140	2.601960577	6.68E-05
GO:0009174	biological _process	pyrimidine ribonucleoside monophosphate biosynthetic process	10	8	15	12977	1140	2.601960577	6.68E-05
GO:0046049	biological _process	UMP metabolic process	10	8	15	12977	1140	2.601960577	6.68E-05
GO:0000166	molecular _function	nucleotide binding	4	225	946	12977	1140	1.436935991	3.39E-46
GO:0005488	molecular	binding	1	496	2476	12977	1140	1.189251884	1.11E-86

	_function								
GO:0036094	molecular _function	small molecule binding	2	229	986	12977	1140	1.402611124	3.25E-45
GO:0097159	molecular _function	organic cyclic compound binding	2	383	1879	12977	1140	1.214302403	2.35E-64
GO:1901265	molecular _function	nucleoside phosphate binding	3	225	946	12977	1140	1.436935991	3.39E-46
GO:1901363	molecular _function	heterocyclic compound binding	2	383	1879	12977	1140	1.214302403	2.35E-64
GO:0005575	cellular_c omponent	cellular_component	0	491	2157	12977	1140	1.373619926	4.10E-108
GO:0005622	cellular_c omponent	intracellular	3	308	931	12977	1140	1.913000356	6.01E-106
GO:0005623	cellular_c omponent	cell	1	401	1249	12977	1140	1.869751838	5.24E-138
GO:0005737	cellular_c omponent	cytoplasm	5	274	572	12977	1140	2.447011919	3.81E-140
GO:0044424	cellular_c omponent	intracellular part	4	291	680	12977	1140	2.284335579	1.57E-133
GO:0044464	cellular_c omponent	cell part	2	394	1200	12977	1140	1.902084302	7.13E-139
GO:0016772	molecular _function	transferase activity, transferring phosphorus-containing groups	3	68	537	12977	1140	0.527535736	0.00442561
GO:0019205	molecular _function	nucleobase-containing compound kinase activity	5	7	17	12977	1140	2.228743253	0.001378086
GO:0001882	molecular _function	nucleoside binding	3	180	776	12977	1140	1.400791427	9.00E-35
GO:0001883	molecular _function	purine nucleoside binding	4	177	772	12977	1140	1.383999685	1.77E-33
GO:0005524	molecular _function	ATP binding	8	156	713	12977	1140	1.316495125	6.38E-27
GO:0017076	molecular _function	purine nucleotide binding	5	179	778	12977	1140	1.389040605	4.50E-34
GO:0030554	molecular	adenyl nucleotide	6	158	715	12977	1140	1.330832489	8.80E-28

	_function	binding							
GO:0032549	molecular _function	ribonucleoside binding	4	178	773	12977	1140	1.39026	6.32E-34
GO:0032550	molecular _function	purine ribonucleoside binding	5	177	772	12977	1140	1.383999685	1.77E-33
GO:0032553	molecular _function	ribonucleotide binding	5	184	801	12977	1140	1.386754696	5.42E-35
GO:0032555	molecular _function	purine ribonucleotide binding	6	178	777	12977	1140	1.382813815	1.28E-33
GO:0032559	molecular _function	adenyl ribonucleotide binding	7	157	714	12977	1140	1.323691658	2.35E-27
GO:0035639	molecular _function	purine ribonucleoside triphosphate binding	4	176	771	12977	1140	1.377695741	5.07E-33
GO:0043167	molecular _function	ion binding	2	327	1450	12977	1140	1.360160813	2.56E-64
GO:0043168	molecular _function	anion binding	3	229	1011	12977	1140	1.366487679	2.93E-43
GO:0055114	biological _process	oxidation-reduction process	3	138	712	12977	1140	1.141642198	1.47E-18
GO:0016491	molecular _function	oxidoreductase activity	2	129	680	12977	1140	1.110687492	1.49E-16
GO:0016627	molecular _function	oxidoreductase activity, acting on the CH-CH group of donors	3	17	49	12977	1140	1.98160417	2.88E-06
GO:0016628	molecular _function	oxidoreductase activity, acting on the CH-CH group of donors, NAD or NADP as acceptor	4	4	12	12977	1140	1.923888672	0.040422466
GO:0016746	molecular _function	transferase activity, transferring acyl groups	3	37	273	12977	1140	0.625547397	0.015347432
GO:0006082	biological _process	organic acid metabolic process	4	135	375	12977	1140	2.034919984	2.38E-48
GO:0006629	biological _process	lipid metabolic process	3	49	188	12977	1140	1.568972165	2.33E-11
GO:0006631	biological _process	fatty acid metabolic process	8	19	56	12977	1140	1.949423764	9.98E-07
GO:0006633	biological	fatty acid biosynthetic	9	13	36	12977	1140	2.039365889	3.32E-05

	_process	process							
GO:0008610	biological _process	lipid biosynthetic process	4	36	97	12977	1140	2.078863332	2.05E-13
GO:0016053	biological _process	organic acid biosynthetic process	5	69	194	12977	1140	2.017462787	3.88E-24
GO:0019752	biological _process	carboxylic acid metabolic process	6	134	370	12977	1140	2.043558903	2.45E-48
GO:0032787	biological _process	monocarboxylic acid metabolic process	7	31	79	12977	1140	2.159266735	2.31E-12
GO:0043436	biological _process	oxoacid metabolic process	5	135	372	12977	1140	2.046507959	8.37E-49
GO:0044255	biological _process	cellular lipid metabolic process	4	45	120	12977	1140	2.093813673	8.05E-17
GO:0044283	biological _process	small molecule biosynthetic process	4	85	248	12977	1140	1.964045798	2.08E-28
GO:0044711	biological _process	single-organism biosynthetic process	3	86	255	12977	1140	1.940762491	3.54E-28
GO:0046394	biological _process	carboxylic acid biosynthetic process	7	69	194	12977	1140	2.017462787	3.88E-24
GO:0072330	biological _process	monocarboxylic acid biosynthetic process	8	17	43	12977	1140	2.170049259	3.64E-07
GO:0005975	biological _process	carbohydrate metabolic process	3	42	264	12977	1140	0.856774476	0.000483766
GO:0008653	biological _process	lipopolysaccharide metabolic process	6	4	14	12977	1140	1.701496251	0.063270064
GO:0009059	biological _process	macromolecule biosynthetic process	4	169	502	12977	1140	1.938187055	2.54E-56
GO:0016051	biological _process	carbohydrate biosynthetic process	5	16	96	12977	1140	0.923888672	0.023668992
GO:0034645	biological _process	cellular macromolecule biosynthetic process	5	160	432	12977	1140	2.075891765	9.92E-60
GO:0043170	biological _process	macromolecule metabolic process	3	336	1597	12977	1140	1.260019998	1.85E-58
GO:0044260	biological _process	cellular macromolecule metabolic process	4	290	1235	12977	1140	1.418464936	5.05E-60
GO:0044262	biological _process	cellular carbohydrate metabolic process	4	12	63	12977	1140	1.11653375	0.020852667

GO:0044723	biological _process	single-organism carbohydrate metabolic process	4	25	131	12977	1140	1.119284361	0.000687605
GO:0016765	molecular _function	transferase activity, transferring alkyl or aryl (other than methyl) groups	3	13	39	12977	1140	1.923888672	8.36E-05
GO:0005996	biological _process	monosaccharide metabolic process	5	12	30	12977	1140	2.186923078	2.23E-05
GO:0046364	biological _process	monosaccharide biosynthetic process	6	5	10	12977	1140	2.508851173	0.003183723
GO:0016874	molecular _function	ligase activity	2	61	197	12977	1140	1.817536691	6.99E-18
GO:0006520	biological _process	cellular amino acid metabolic process	7	101	269	12977	1140	2.095600293	8.91E-38
GO:0006541	biological _process	glutamine metabolic process	9	7	30	12977	1140	1.409315499	0.033206109
GO:0009064	biological _process	glutamine family amino acid metabolic process	8	19	70	12977	1140	1.627495669	3.43E-05
GO:1901605	biological _process	alpha-amino acid metabolic process	8	60	171	12977	1140	1.997889253	1.20E-20
GO:0016879	molecular _function	ligase activity, forming carbon-nitrogen bonds	3	28	101	12977	1140	1.657994612	2.10E-07
GO:0016787	molecular _function	hydrolase activity	2	190	1220	12977	1140	0.826041349	5.40E-15
GO:0005525	molecular _function	GTP binding	8	21	60	12977	1140	1.994278	1.37E-07
GO:0019001	molecular _function	guanyl nucleotide binding	6	22	65	12977	1140	1.945914978	1.28E-07
GO:0032561	molecular _function	guanyl ribonucleotide binding	7	22	65	12977	1140	1.945914978	1.28E-07
GO:0008270	molecular _function	zinc ion binding	6	30	129	12977	1140	1.404514513	3.70E-06
GO:0043169	molecular _function	cation binding	3	150	612	12977	1140	1.48028202	1.63E-31
GO:0046872	molecular _function	metal ion binding	4	149	596	12977	1140	1.508851173	2.57E-32

GO:0046914	molecular _function	transition metal ion binding	5	61	240	12977	1140	1.532697915	1.74E-13
GO:0003933	molecular _function	GTP cyclohydrolase activity	6	3	7	12977	1140	2.286458751	0.043326175
GO:0016810	molecular _function	hydrolase activity, acting on carbon-nitrogen (but not peptide) bonds	3	22	138	12977	1140	0.859758335	0.013027895
GO:0016814	molecular _function	hydrolase activity, acting on carbon-nitrogen (but not peptide) bonds, in cyclic amidines	4	8	27	12977	1140	1.753963671	0.00554338
GO:0019238	molecular _function	cyclohydrolase activity	5	5	10	12977	1140	2.508851173	0.003183723
GO:0016829	molecular _function	lyase activity	2	53	213	12977	1140	1.502062007	2.12E-11
GO:0006766	biological _process	vitamin metabolic process	4	15	53	12977	1140	1.687821314	0.000162849
GO:0006767	biological _process	water-soluble vitamin metabolic process	5	15	53	12977	1140	1.687821314	0.000162849
GO:0009110	biological _process	vitamin biosynthetic process	5	14	50	12977	1140	1.672349905	0.000311958
GO:0042364	biological _process	water-soluble vitamin biosynthetic process	6	14	50	12977	1140	1.672349905	0.000311958
GO:0016830	molecular _function	carbon-carbon lyase activity	3	19	67	12977	1140	1.690689496	1.75E-05
GO:0030145	molecular _function	manganese ion binding	6	10	22	12977	1140	2.371347649	3.57E-05
GO:0000287	molecular _function	magnesium ion binding	5	39	109	12977	1140	2.026069067	7.16E-14
GO:0006508	biological _process	proteolysis	5	32	208	12977	1140	0.808411455	0.004325137
GO:0019538	biological _process	protein metabolic process	4	159	586	12977	1140	1.626977274	2.90E-39
GO:0016020	cellular_c omponent	membrane	1	184	1274	12977	1140	0.717263566	2.08E-11

GO:0004175	molecular _function	endopeptidase activity	5	16	83	12977	1140	1.133811741	0.006854102
GO:0004222	molecular _function	metalloendopeptidase activity	6	11	35	12977	1140	1.838999774	0.000570975
GO:0008233	molecular _function	peptidase activity	3	31	203	12977	1140	0.797711566	0.005535782
GO:0008237	molecular _function	metallopeptidase activity	5	16	66	12977	1140	1.464457053	0.000612899
GO:0070011	molecular _function	peptidase activity, acting on L-amino acid peptides	4	29	177	12977	1140	0.899226618	0.002765812
GO:0008144	molecular _function	drug binding	2	4	12	12977	1140	1.923888672	0.040422466
GO:0008658	molecular _function	penicillin binding	6	4	12	12977	1140	1.923888672	0.040422466
GO:0033293	molecular _function	monocarboxylic acid binding	5	4	12	12977	1140	1.923888672	0.040422466
GO:0007049	biological _process	cell cycle	3	27	47	12977	1140	2.709149823	6.30E-16
GO:0044699	biological _process	single-organism process	1	189	1366	12977	1140	0.655351829	4.12E-10
GO:0044763	biological _process	single-organism cellular process	2	169	1252	12977	1140	0.619711762	3.20E-08
GO:0016043	biological _process	cellular component organization	2	60	133	12977	1140	2.360459333	1.82E-27
GO:0022607	biological _process	cellular component assembly	3	35	61	12977	1140	2.707396852	1.69E-20
GO:0044085	biological _process	cellular component biogenesis	2	72	119	12977	1140	2.783958411	3.15E-44
GO:0071840	biological _process	cellular component organization or biogenesis	1	83	176	12977	1140	2.424458985	8.49E-40
GO:0006810	biological _process	transport	3	110	802	12977	1140	0.64275246	7.18E-06
GO:0008104	biological _process	protein localization	3	34	140	12977	1140	1.467030997	2.64E-07
GO:0009306	biological	protein secretion	7	12	50	12977	1140	1.449957484	0.003656386

	_process									
GO:0015031	biological _process	protein transport	5	30	133	12977	1140	1.360459333	6.96E-06
GO:0032940	biological _process	secretion by cell	6	12	50	12977	1140	1.449957484	0.003656386
GO:0033036	biological _process	macromolecule localization	2	37	149	12977	1140	1.499136018	3.88E-08
GO:0044765	biological _process	single-organism transport	4	83	592	12977	1140	0.674437238	5.90E-05
GO:0045184	biological _process	establishment of protein localization	4	30	133	12977	1140	1.360459333	6.96E-06
GO:0046903	biological _process	secretion	5	12	50	12977	1140	1.449957484	0.003656386
GO:0051179	biological _process	localization	1	114	835	12977	1140	0.636108799	5.84E-06
GO:0051234	biological _process	establishment of localization	2	110	803	12977	1140	0.640954709	7.52E-06
GO:0051641	biological _process	cellular localization	3	23	108	12977	1140	1.277525627	0.000241584
GO:0051649	biological _process	establishment of localization in cell	4	18	100	12977	1140	1.034919984	0.007928409
GO:0071702	biological _process	organic substance transport	4	54	240	12977	1140	1.356848079	7.14E-10
GO:0009279	cellular_c omponent	cell outer membrane	6	9	20	12977	1140	2.356848079	0.000106793
GO:0019867	cellular_c omponent	outer membrane	2	13	48	12977	1140	1.62432839	0.000763119
GO:0030312	cellular_c omponent	external encapsulating structure	4	14	35	12977	1140	2.186923078	3.92E-06
GO:0030313	cellular_c omponent	cell envelope	4	14	29	12977	1140	2.4582251	2.72E-07
GO:0031975	cellular_c omponent	envelope	3	14	29	12977	1140	2.4582251	2.72E-07
GO:0044462	cellular_c omponent	external encapsulating structure part	5	13	28	12977	1140	2.401935969	1.36E-06
GO:0071944	cellular_c omponent	cell periphery	3	90	273	12977	1140	1.907947128	1.19E-28

GO:0005215	molecular _function	transporter activity	1	70	488	12977	1140	0.707396852	0.000131355
GO:0008565	molecular _function	protein transporter activity	3	19	38	12977	1140	2.508851173	5.50E-10
GO:0022892	molecular _function	substrate-specific transporter activity	2	56	293	12977	1140	1.12144924	1.54E-07
GO:0009423	biological _process	chorismate biosynthetic process	9	6	12	12977	1140	2.508851173	0.001078378
GO:0043648	biological _process	dicarboxylic acid metabolic process	7	26	68	12977	1140	2.12182805	3.38E-10
GO:0043650	biological _process	dicarboxylic acid biosynthetic process	8	14	27	12977	1140	2.561318593	9.02E-08
GO:0046417	biological _process	chorismate metabolic process	8	6	15	12977	1140	2.186923078	0.003944172
GO:0008652	biological _process	cellular amino acid biosynthetic process	8	50	135	12977	1140	2.075891765	2.22E-18
GO:0009072	biological _process	aromatic amino acid family metabolic process	8	15	39	12977	1140	2.130339549	2.95E-06
GO:0009073	biological _process	aromatic amino acid family biosynthetic process	9	11	25	12977	1140	2.324426602	1.87E-05
GO:0016835	molecular _function	carbon-oxygen lyase activity	3	22	72	12977	1140	1.79835779	8.93E-07
GO:0016853	molecular _function	isomerase activity	2	29	121	12977	1140	1.447968931	2.95E-06
GO:0016860	molecular _function	intramolecular oxidoreductase activity	3	6	17	12977	1140	2.006350832	0.007830073
GO:0016861	molecular _function	intramolecular oxidoreductase activity, interconverting aldoses and ketoses	4	5	13	12977	1140	2.130339549	0.011011531
GO:0006006	biological _process	glucose metabolic process	7	11	27	12977	1140	2.213395289	4.39E-05
GO:0006007	biological _process	glucose catabolic process	8	8	22	12977	1140	2.049419554	0.001408711
GO:0006091	biological	generation of precursor	3	26	70	12977	1140	2.080007874	6.96E-10

	_process	metabolites and energy							
GO:0006096	biological _process	glycolysis	9	7	16	12977	1140	2.316206095	0.000934959
GO:0009056	biological _process	catabolic process	2	56	191	12977	1140	1.738777267	2.69E-15
GO:0016052	biological _process	carbohydrate catabolic process	4	10	35	12977	1140	1.701496251	0.002401936
GO:0019318	biological _process	hexose metabolic process	6	11	27	12977	1140	2.213395289	4.39E-05
GO:0019320	biological _process	hexose catabolic process	7	8	22	12977	1140	2.049419554	0.001408711
GO:0044724	biological _process	single-organism carbohydrate catabolic process	5	9	29	12977	1140	1.820795179	0.002299916
GO:0046365	biological _process	monosaccharide catabolic process	6	8	22	12977	1140	2.049419554	0.001408711
GO:1901575	biological _process	organic substance catabolic process	3	56	177	12977	1140	1.848600545	6.50E-17
GO:0006094	biological _process	gluconeogenesis	8	4	8	12977	1140	2.508851173	0.009538302
GO:0019319	biological _process	hexose biosynthetic process	7	4	8	12977	1140	2.508851173	0.009538302
GO:0016462	molecular _function	pyrophosphatase activity	5	66	323	12977	1140	1.217854937	5.22E-10
GO:0016817	molecular _function	hydrolase activity, acting on acid anhydrides	3	67	329	12977	1140	1.212996589	4.39E-10
GO:0016818	molecular _function	hydrolase activity, acting on acid anhydrides, in phosphorus-containing anhydrides	4	67	326	12977	1140	1.226212209	2.93E-10
GO:0017111	molecular _function	nucleoside-triphosphatase activity	6	60	314	12977	1140	1.121121019	5.17E-08
GO:0008094	molecular _function	DNA-dependent ATPase activity	9	8	38	12977	1140	1.260923659	0.038675319
GO:0016887	molecular	ATPase activity	7	40	217	12977	1140	1.069228035	2.85E-05

	_function								
GO:0042623	molecular _function	ATPase activity, coupled	8	24	153	12977	1140	0.836425831	0.011163314
GO:0006259	biological _process	DNA metabolic process	6	59	531	12977	1140	0.338926171	0.075601361
GO:0090304	biological _process	nucleic acid metabolic process	5	172	902	12977	1140	1.118132304	1.34E-22
GO:0006260	biological _process	DNA replication	7	18	80	12977	1140	1.356848079	0.000667309
GO:0003676	molecular _function	nucleic acid binding	3	187	1016	12977	1140	1.067060946	8.23E-23
GO:0071103	biological _process	DNA conformation change	7	12	51	12977	1140	1.421388331	0.004353077
GO:0006281	biological _process	DNA repair	7	26	98	12977	1140	1.594581047	1.48E-06
GO:0006950	biological _process	response to stress	2	37	177	12977	1140	1.250698988	3.31E-06
GO:0006974	biological _process	response to DNA damage stimulus	5	26	98	12977	1140	1.594581047	1.48E-06
GO:0033554	biological _process	cellular response to stress	4	30	114	12977	1140	1.582851754	2.45E-07
GO:0009432	biological _process	SOS response	5	8	16	12977	1140	2.508851173	0.000119965
GO:0009991	biological _process	response to extracellular stimulus	3	8	18	12977	1140	2.338926171	0.000307904
GO:0031668	biological _process	cellular response to extracellular stimulus	4	8	18	12977	1140	2.338926171	0.000307904
GO:0071496	biological _process	cellular response to external stimulus	3	8	18	12977	1140	2.338926171	0.000307904
GO:0003995	molecular _function	acyl-CoA dehydrogenase activity	4	5	16	12977	1140	1.830779268	0.025564747
GO:0048037	molecular _function	cofactor binding	2	68	281	12977	1140	1.461887694	7.32E-14
GO:0050660	molecular _function	flavin adenine dinucleotide binding	5	21	73	12977	1140	1.711344037	4.51E-06
GO:0050662	molecular _function	coenzyme binding	3	43	188	12977	1140	1.380527076	3.36E-08

GO:0016836	molecular _function	hydro-lyase activity	4	16	52	12977	1140	1.808411455	3.19E-05
GO:0016779	molecular _function	nucleotidyltransferase activity	4	28	149	12977	1140	1.097037574	0.000395176
GO:0000049	molecular _function	tRNA binding	5	14	21	12977	1140	2.923888672	9.93E-10
GO:0003723	molecular _function	RNA binding	4	88	162	12977	1140	2.628432788	9.94E-49
GO:0016070	biological _process	RNA metabolic process	6	112	318	12977	1140	2.003323139	7.23E-39
GO:0090305	biological _process	nucleic acid phosphodiester bond hydrolysis	6	31	149	12977	1140	1.243878963	2.61E-05
GO:0090501	biological _process	RNA phosphodiester bond hydrolysis	7	11	21	12977	1140	2.575965369	2.43E-06
GO:0004518	molecular _function	nuclease activity	4	32	155	12977	1140	1.232726767	2.21E-05
GO:0004540	molecular _function	ribonuclease activity	5	11	21	12977	1140	2.575965369	2.43E-06
GO:0016788	molecular _function	hydrolase activity, acting on ester bonds	3	52	285	12977	1140	1.054472782	1.98E-06
GO:0006396	biological _process	RNA processing	7	48	83	12977	1140	2.718774242	3.05E-28
GO:0006399	biological _process	tRNA metabolic process	8	51	86	12977	1140	2.75501176	9.53E-31
GO:0008033	biological _process	tRNA processing	9	30	51	12977	1140	2.743316426	5.12E-18
GO:0010467	biological _process	gene expression	4	166	358	12977	1140	2.400074827	1.04E-79
GO:0034470	biological _process	ncRNA processing	8	45	73	12977	1140	2.81087971	3.58E-28
GO:0034660	biological _process	ncRNA metabolic process	7	66	108	12977	1140	2.79835779	6.45E-41
GO:0046939	biological _process	nucleotide phosphorylation	8	6	11	12977	1140	2.634382055	0.000612152
GO:0016776	molecular _function	phosphotransferase activity, phosphate	4	8	14	12977	1140	2.701496251	3.57E-05

		group as acceptor							
GO:0019201	molecular _function	nucleotide kinase activity	6	5	10	12977	1140	2.508851173	0.003183723
GO:0006163	biological _process	purine nucleotide metabolic process	8	26	92	12977	1140	1.685728935	4.02E-07
GO:0072521	biological _process	purine-containing compound metabolic process	4	30	104	12977	1140	1.71530205	2.70E-08
GO:0006351	biological _process	transcription, DNA-dependent	8	31	148	12977	1140	1.253594117	2.26E-05
GO:0032774	biological _process	RNA biosynthetic process	7	32	161	12977	1140	1.177934295	4.81E-05
GO:0003899	molecular _function	DNA-directed RNA polymerase activity	6	8	21	12977	1140	2.11653375	0.001028167
GO:0034062	molecular _function	RNA polymerase activity	5	8	21	12977	1140	2.11653375	0.001028167
GO:0003677	molecular _function	DNA binding	4	78	691	12977	1140	0.361711491	0.030422894
GO:0016778	molecular _function	diphosphotransferase activity	4	4	10	12977	1140	2.186923078	0.020984057
GO:0009150	biological _process	purine ribonucleotide metabolic process	9	23	88	12977	1140	1.57298151	8.13E-06
GO:0033865	biological _process	nucleoside bisphosphate metabolic process	7	9	22	12977	1140	2.219344555	0.000244224
GO:0033875	biological _process	ribonucleoside bisphosphate metabolic process	8	9	22	12977	1140	2.219344555	0.000244224
GO:0034032	biological _process	purine nucleoside bisphosphate metabolic process	8	9	22	12977	1140	2.219344555	0.000244224
GO:0042278	biological _process	purine nucleoside metabolic process	7	27	75	12977	1140	2.034919984	6.96E-10
GO:0046128	biological _process	purine ribonucleoside metabolic process	8	27	75	12977	1140	2.034919984	6.96E-10
GO:1901068	biological _process	guanosine-containing compound metabolic process	9	5	16	12977	1140	1.830779268	0.025564747

GO:0006400	biological _process	tRNA modification	10	22	37	12977	1140	2.758829426	1.75E-13
GO:0008616	biological _process	queuosine biosynthetic process	11	4	8	12977	1140	2.508851173	0.009538302
GO:0009451	biological _process	RNA modification	7	39	61	12977	1140	2.863516054	2.60E-25
GO:0043412	biological _process	macromolecule modification	4	67	314	12977	1140	1.280319614	5.53E-11
GO:0046116	biological _process	queuosine metabolic process	8	4	8	12977	1140	2.508851173	0.009538302
GO:0016757	molecular _function	transferase activity, transferring glycosyl groups	3	16	79	12977	1140	1.205070425	0.00422592
GO:0016763	molecular _function	transferase activity, transferring pentosyl groups	4	10	31	12977	1140	1.876582957	0.000880882
GO:0016021	cellular_c omponent	integral to membrane	4	115	716	12977	1140	0.870525446	7.47E-10
GO:0031224	cellular_c omponent	intrinsic to membrane	3	116	719	12977	1140	0.876984207	4.78E-10
GO:0044425	cellular_c omponent	membrane part	2	131	779	12977	1140	0.936794656	1.28E-12
GO:0006605	biological _process	protein targeting	7	6	22	12977	1140	1.634382055	0.025544762
GO:0034613	biological _process	cellular protein localization	5	11	57	12977	1140	1.135392777	0.02534832
GO:0070727	biological _process	cellular macromolecule localization	4	11	57	12977	1140	1.135392777	0.02534832
GO:0008320	molecular _function	protein transmembrane transporter activity	5	5	6	12977	1140	3.245816767	0.000142659
GO:0022804	molecular _function	active transmembrane transporter activity	3	23	159	12977	1140	0.719530173	0.029792641
GO:0022857	molecular _function	transmembrane transporter activity	2	41	301	12977	1140	0.632783501	0.009564158
GO:0022884	molecular _function	macromolecule transmembrane transporter activity	4	5	8	12977	1140	2.830779268	0.00096793

GO:0022891	molecular_function	substrate-specific transmembrane transporter activity	3	37	241	12977	1140	0.805415202	0.002170013
GO:0055085	biological_process	transmembrane transport	5	55	436	12977	1140	0.522026561	0.011630285
GO:0071806	biological_process	protein transmembrane transport	6	13	34	12977	1140	2.12182805	1.65E-05
GO:0065002	biological_process	intracellular protein transmembrane transport	7	4	8	12977	1140	2.508851173	0.009538302
GO:0005886	cellular_component	plasma membrane	4	78	240	12977	1140	1.887362796	2.28E-24
GO:0004659	molecular_function	prenyltransferase activity	4	4	11	12977	1140	2.049419554	0.029792641
GO:0005342	molecular_function	organic acid transmembrane transporter activity	4	7	36	12977	1140	1.146281093	0.075095565
GO:0008509	molecular_function	anion transmembrane transporter activity	5	12	58	12977	1140	1.235832678	0.011886246
GO:0008514	molecular_function	organic anion transmembrane transporter activity	6	8	37	12977	1140	1.299397807	0.033206109
GO:0015075	molecular_function	ion transmembrane transporter activity	4	31	218	12977	1140	0.694863158	0.014916365
GO:0046943	molecular_function	carboxylic acid transmembrane transporter activity	7	7	36	12977	1140	1.146281093	0.075095565
GO:0003333	biological_process	amino acid transmembrane transport	10	7	30	12977	1140	1.409315499	0.033206109
GO:0006811	biological_process	ion transport	5	31	221	12977	1140	0.675144924	0.017487037
GO:0006820	biological_process	anion transport	6	17	74	12977	1140	1.386860648	0.000764406
GO:0006865	biological_process	amino acid transport	9	7	36	12977	1140	1.146281093	0.075095565
GO:0015711	biological	organic anion transport	7	11	50	12977	1140	1.324426602	0.010636146

	_process								
GO:0015849	biological _process	organic acid transport	5	9	46	12977	1140	1.155214218	0.04165388
GO:0034220	biological _process	ion transmembrane transport	6	17	116	12977	1140	0.738333019	0.054831012
GO:0046942	biological _process	carboxylic acid transport	8	9	46	12977	1140	1.155214218	0.04165388
GO:0071705	biological _process	nitrogen compound transport	4	16	77	12977	1140	1.242064632	0.003224942
GO:0016645	molecular _function	oxidoreductase activity, acting on the CH-NH group of donors	3	5	19	12977	1140	1.582851754	0.049139564
GO:0006544	biological _process	glycine metabolic process	9	6	7	12977	1140	3.286458751	1.73E-05
GO:0009069	biological _process	serine family amino acid metabolic process	8	9	21	12977	1140	2.286458751	0.000162849
GO:0009070	biological _process	serine family amino acid biosynthetic process	9	4	11	12977	1140	2.049419554	0.029792641
GO:1901607	biological _process	alpha-amino acid biosynthetic process	9	38	98	12977	1140	2.142068842	7.50E-15
GO:0006575	biological _process	cellular modified amino acid metabolic process	8	17	47	12977	1140	2.041725162	1.52E-06
GO:0006732	biological _process	coenzyme metabolic process	4	41	101	12977	1140	2.208191695	8.44E-17
GO:0006760	biological _process	folic acid-containing compound metabolic process	9	8	12	12977	1140	2.923888672	7.52E-06
GO:0009108	biological _process	coenzyme biosynthetic process	5	36	80	12977	1140	2.356848079	1.51E-16
GO:0009396	biological _process	folic acid-containing compound biosynthetic process	10	6	8	12977	1140	3.093813673	5.80E-05
GO:0042398	biological _process	cellular modified amino acid biosynthetic process	9	12	31	12977	1140	2.139617363	3.29E-05
GO:0042558	biological _process	pteridine-containing compound metabolic	4	10	15	12977	1140	2.923888672	4.12E-07

		process							
GO:0042559	biological _process	pteridine-containing compound biosynthetic process	5	8	10	12977	1140	3.186923078	9.96E-07
GO:0046653	biological _process	tetrahydrofolate metabolic process	10	5	7	12977	1140	3.023424346	0.000409099
GO:0051186	biological _process	cofactor metabolic process	3	57	143	12977	1140	2.18186985	8.48E-23
GO:0051188	biological _process	cofactor biosynthetic process	4	47	114	12977	1140	2.23055001	1.52E-19
GO:0006730	biological _process	one-carbon metabolic process	4	6	10	12977	1140	2.771885579	0.000313267
GO:0050661	molecular _function	NADP binding	5	7	25	12977	1140	1.672349905	0.013783745
GO:0051287	molecular _function	NAD binding	5	13	41	12977	1140	1.851738886	0.000145438
GO:0072524	biological _process	pyridine-containing compound metabolic process	4	10	32	12977	1140	1.830779268	0.001114317
GO:0072525	biological _process	pyridine-containing compound biosynthetic process	5	8	24	12977	1140	1.923888672	0.002598833
GO:0016614	molecular _function	oxidoreductase activity, acting on CH-OH group of donors	3	27	139	12977	1140	1.144797602	0.000300357
GO:0016616	molecular _function	oxidoreductase activity, acting on the CH-OH group of donors, NAD or NADP as acceptor	4	24	120	12977	1140	1.186923078	0.000445112
GO:0010468	biological _process	regulation of gene expression	5	48	248	12977	1140	1.139617363	9.58E-07
GO:0010608	biological _process	posttranscriptional regulation of gene expression	6	11	22	12977	1140	2.508851173	4.22E-06
GO:0019222	biological _process	regulation of metabolic process	3	57	339	12977	1140	0.936599724	7.49E-06
GO:0050789	biological	regulation of biological	2	89	658	12977	1140	0.62264083	0.00011292

	_process	process							
GO:0060255	biological _process	regulation of macromolecule metabolic process	4	51	264	12977	1140	1.136882395	4.30E-07
GO:0065007	biological _process	biological regulation	1	90	683	12977	1140	0.584962501	0.000248865
GO:0065008	biological _process	regulation of biological quality	2	31	91	12977	1140	1.955252843	1.64E-10
GO:0016044	biological _process	cellular membrane organization	4	8	14	12977	1140	2.701496251	3.57E-05
GO:0043163	biological _process	cell envelope organization	4	7	7	12977	1140	3.508851173	3.23E-07
GO:0043165	biological _process	Gram-negative-bacterium-type cell outer membrane assembly	6	7	7	12977	1140	3.508851173	3.23E-07
GO:0044091	biological _process	membrane biogenesis	3	7	8	12977	1140	3.316206095	2.04E-06
GO:0045229	biological _process	external encapsulating structure organization	3	23	37	12977	1140	2.822959763	1.14E-14
GO:0061024	biological _process	membrane organization	3	8	14	12977	1140	2.701496251	3.57E-05
GO:0071709	biological _process	membrane assembly	5	7	8	12977	1140	3.316206095	2.04E-06
GO:0003755	molecular _function	peptidyl-prolyl cis-trans isomerase activity	4	3	9	12977	1140	1.923888672	0.08055066
GO:0016859	molecular _function	cis-trans isomerase activity	3	3	9	12977	1140	1.923888672	0.08055066
GO:0042597	cellular_c omponent	periplasmic space	3	10	24	12977	1140	2.245816767	8.49E-05
GO:0030288	cellular_c omponent	outer membrane-bounded periplasmic space	6	4	8	12977	1140	2.508851173	0.009538302
GO:0006457	biological _process	protein folding	6	13	29	12977	1140	2.351309896	2.14E-06
GO:0044267	biological _process	cellular protein metabolic process	5	129	382	12977	1140	1.9426496	1.24E-42

GO:0000413	biological _process	protein peptidyl-prolyl isomerization	9	3	9	12977	1140	1.923888672	0.08055066
GO:0006464	biological _process	cellular protein modification process	6	28	220	12977	1140	0.534846381	0.06417215
GO:0018208	biological _process	peptidyl-proline modification	8	3	9	12977	1140	1.923888672	0.08055066
GO:0036211	biological _process	protein modification process	5	28	220	12977	1140	0.534846381	0.06417215
GO:0005515	molecular _function	protein binding	2	18	79	12977	1140	1.374995426	0.000570975
GO:0051082	molecular _function	unfolded protein binding	3	10	23	12977	1140	2.307217312	5.64E-05
GO:1901264	biological _process	carbohydrate derivative transport	5	5	9	12977	1140	2.660854266	0.00186774
GO:0006635	biological _process	fatty acid beta-oxidation	10	3	7	12977	1140	2.286458751	0.043326175
GO:0009062	biological _process	fatty acid catabolic process	9	5	11	12977	1140	2.371347649	0.005197577
GO:0016042	biological _process	lipid catabolic process	4	6	14	12977	1140	2.286458751	0.002689376
GO:0016054	biological _process	organic acid catabolic process	5	24	57	12977	1140	2.260923659	1.82E-10
GO:0019395	biological _process	fatty acid oxidation	9	3	7	12977	1140	2.286458751	0.043326175
GO:0030258	biological _process	lipid modification	5	5	13	12977	1140	2.130339549	0.011011531
GO:0034440	biological _process	lipid oxidation	6	3	7	12977	1140	2.286458751	0.043326175
GO:0044242	biological _process	cellular lipid catabolic process	5	6	12	12977	1140	2.508851173	0.001078378
GO:0044248	biological _process	cellular catabolic process	3	39	136	12977	1140	1.70679055	1.73E-10
GO:0044282	biological _process	small molecule catabolic process	4	26	66	12977	1140	2.164896771	1.65E-10
GO:0044712	biological _process	single-organism catabolic process	3	26	66	12977	1140	2.164896771	1.65E-10
GO:0046395	biological	carboxylic acid catabolic	7	24	57	12977	1140	2.260923659	1.82E-10

	_process	process							
GO:0072329	biological _process	monocarboxylic acid catabolic process	8	6	12	12977	1140	2.508851173	0.001078378
GO:0015698	biological _process	inorganic anion transport	7	6	24	12977	1140	1.508851173	0.038029961
GO:0032991	cellular_c omponent	macromolecular complex	1	101	224	12977	1140	2.359707733	2.62E-46
GO:0043234	cellular_c omponent	protein complex	2	50	150	12977	1140	1.923888672	3.23E-16
GO:0006412	biological _process	translation	6	90	131	12977	1140	2.967281267	1.29E-62
GO:0006414	biological _process	translational elongation	7	4	13	12977	1140	1.808411455	0.049501873
GO:0006518	biological _process	peptide metabolic process	5	5	15	12977	1140	1.923888672	0.020262755
GO:0043603	biological _process	cellular amide metabolic process	4	15	45	12977	1140	1.923888672	2.09E-05
GO:0043604	biological _process	amide biosynthetic process	5	9	26	12977	1140	1.978336456	0.000990091
GO:0003746	molecular _function	translation elongation factor activity	6	4	13	12977	1140	1.808411455	0.049501873
GO:0008135	molecular _function	translation factor activity, nucleic acid binding	5	11	22	12977	1140	2.508851173	4.22E-06
GO:0004470	molecular _function	malic enzyme activity	5	3	8	12977	1140	2.093813673	0.059179072
GO:0016615	molecular _function	malate dehydrogenase activity	4	4	9	12977	1140	2.338926171	0.015264401
GO:0006108	biological _process	malate metabolic process	8	3	7	12977	1140	2.286458751	0.043326175
GO:0004471	molecular _function	malate dehydrogenase (decarboxylating) activity	6	3	8	12977	1140	2.093813673	0.059179072
GO:0000270	biological _process	peptidoglycan metabolic process	6	21	44	12977	1140	2.441736977	1.82E-10
GO:0006022	biological _process	aminoglycan metabolic process	4	21	49	12977	1140	2.286458751	1.97E-09

GO:0009057	biological _process	macromolecule catabolic process	4	17	64	12977	1140	1.596314014	0.000129089
GO:0030203	biological _process	glycosaminoglycan metabolic process	5	21	45	12977	1140	2.409315499	2.94E-10
GO:1901136	biological _process	carbohydrate derivative catabolic process	4	6	22	12977	1140	1.634382055	0.025544762
GO:1901565	biological _process	organonitrogen compound catabolic process	4	20	69	12977	1140	1.722254811	7.00E-06
GO:0009308	biological _process	amine metabolic process	4	9	36	12977	1140	1.508851173	0.009854463
GO:0016651	molecular _function	oxidoreductase activity, acting on NAD(P)H	3	18	43	12977	1140	2.252511419	5.64E-08
GO:0016655	molecular _function	oxidoreductase activity, acting on NAD(P)H, quinone or similar compound as acceptor	4	13	22	12977	1140	2.749859272	3.83E-08
GO:0003916	molecular _function	DNA topoisomerase activity	5	5	11	12977	1140	2.371347649	0.005197577
GO:0003918	molecular _function	DNA topoisomerase type II (ATP-hydrolyzing) activity	10	4	7	12977	1140	2.701496251	0.005535782
GO:0061505	molecular _function	DNA topoisomerase II activity	6	4	7	12977	1140	2.701496251	0.005535782
GO:0006265	biological _process	DNA topological change	8	5	11	12977	1140	2.371347649	0.005197577
GO:0005694	cellular_c omponent	chromosome	7	6	17	12977	1140	2.006350832	0.007830073
GO:0043226	cellular_c omponent	organelle	1	57	149	12977	1140	2.122572666	9.34E-22
GO:0043228	cellular_c omponent	non-membrane-bounded organelle	2	57	130	12977	1140	2.319373374	2.55E-25
GO:0043229	cellular_c omponent	intracellular organelle	5	57	148	12977	1140	2.132287821	6.41E-22
GO:0043232	cellular_c omponent	intracellular non-membrane-bounded organelle	6	57	130	12977	1140	2.319373374	2.55E-25

GO:0007059	biological _process	chromosome segregation	3	5	12	12977	1140	2.245816767	0.007834784
GO:0009063	biological _process	cellular amino acid catabolic process	8	18	44	12977	1140	2.219344555	8.57E-08
GO:1901606	biological _process	alpha-amino acid catabolic process	9	17	42	12977	1140	2.203996591	2.49E-07
GO:0009055	molecular _function	electron carrier activity	1	18	58	12977	1140	1.820795179	7.70E-06
GO:0046983	molecular _function	protein dimerization activity	3	6	12	12977	1140	2.508851173	0.001078378
GO:0008360	biological _process	regulation of cell shape	6	18	27	12977	1140	2.923888672	2.50E-12
GO:0022603	biological _process	regulation of anatomical structure morphogenesis	4	18	28	12977	1140	2.871421252	6.26E-12
GO:0022604	biological _process	regulation of cell morphogenesis	5	18	28	12977	1140	2.871421252	6.26E-12
GO:0050793	biological _process	regulation of developmental process	3	18	28	12977	1140	2.871421252	6.26E-12
GO:0050794	biological _process	regulation of cellular process	3	80	608	12977	1140	0.582851754	0.000625263
GO:0051128	biological _process	regulation of cellular component organization	4	23	36	12977	1140	2.862488127	4.86E-15
GO:0051301	biological _process	cell division	3	27	43	12977	1140	2.83747392	2.82E-17
GO:0016881	molecular _function	acid-amino acid ligase activity	4	11	46	12977	1140	1.444720835	0.005661369
GO:0071554	biological _process	cell wall organization or biogenesis	3	18	41	12977	1140	2.32122417	2.35E-08
GO:0071555	biological _process	cell wall organization	4	16	30	12977	1140	2.601960577	5.02E-09
GO:0006023	biological _process	aminoglycan biosynthetic process	5	16	26	12977	1140	2.808411455	2.89E-10
GO:0006024	biological _process	glycosaminoglycan biosynthetic process	6	16	26	12977	1140	2.808411455	2.89E-10
GO:0009252	biological _process	peptidoglycan biosynthetic process	8	16	26	12977	1140	2.808411455	2.89E-10

GO:0009273	biological _process	peptidoglycan-based cell wall biogenesis	5	16	26	12977	1140	2.808411455	2.89E-10
GO:0042546	biological _process	cell wall biogenesis	4	16	26	12977	1140	2.808411455	2.89E-10
GO:0044036	biological _process	cell wall macromolecule metabolic process	5	17	35	12977	1140	2.467030997	9.58E-09
GO:0044038	biological _process	cell wall macromolecule biosynthetic process	7	16	26	12977	1140	2.808411455	2.89E-10
GO:0070589	biological _process	cellular component macromolecule biosynthetic process	6	16	26	12977	1140	2.808411455	2.89E-10
GO:0000003	biological _process	reproduction	1	5	8	12977	1140	2.830779268	0.00096793
GO:0000910	biological _process	cytokinesis	5	11	16	12977	1140	2.968282791	6.03E-08
GO:0019954	biological _process	asexual reproduction	2	5	7	12977	1140	3.023424346	0.000409099
GO:0022402	biological _process	cell cycle process	4	12	26	12977	1140	2.393373955	3.92E-06
GO:0032505	biological _process	reproduction of a single-celled organism	2	5	7	12977	1140	3.023424346	0.000409099
GO:0043093	biological _process	cytokinesis by binary fission	6	5	7	12977	1140	3.023424346	0.000409099
GO:0032153	cellular_c omponent	cell division site	3	5	6	12977	1140	3.245816767	0.000142659
GO:0005887	cellular_c omponent	integral to plasma membrane	7	10	17	12977	1140	2.743316426	2.02E-06
GO:0031226	cellular_c omponent	intrinsic to plasma membrane	6	12	21	12977	1140	2.701496251	2.34E-07
GO:0044459	cellular_c omponent	plasma membrane part	5	13	23	12977	1140	2.685728935	7.75E-08
GO:0008168	molecular _function	methyltransferase activity	4	36	195	12977	1140	1.07144586	7.37E-05
GO:0016741	molecular _function	transferase activity, transferring one-carbon groups	3	39	212	12977	1140	1.066332937	3.79E-05
GO:0006364	biological	rRNA processing	9	17	25	12977	1140	2.952457824	6.99E-12

	_process								
GO:0016072	biological _process	rRNA metabolic process	8	17	25	12977	1140	2.952457824	6.99E-12
GO:0022613	biological _process	ribonucleoprotein complex biogenesis	3	24	35	12977	1140	2.964530656	9.99E-17
GO:0042254	biological _process	ribosome biogenesis	4	24	35	12977	1140	2.964530656	9.99E-17
GO:0008170	molecular _function	N-methyltransferase activity	5	8	21	12977	1140	2.11653375	0.001028167
GO:0008173	molecular _function	RNA methyltransferase activity	5	16	24	12977	1140	2.923888672	5.29E-11
GO:0008649	molecular _function	rRNA methyltransferase activity	6	12	18	12977	1140	2.923888672	2.18E-08
GO:0008757	molecular _function	S-adenosylmethionine-dependent methyltransferase activity	5	20	53	12977	1140	2.102858813	6.96E-08
GO:0000154	biological _process	rRNA modification	10	12	18	12977	1140	2.923888672	2.18E-08
GO:0001510	biological _process	RNA methylation	8	16	25	12977	1140	2.864994983	1.32E-10
GO:0031167	biological _process	rRNA methylation	11	12	18	12977	1140	2.923888672	2.18E-08
GO:0032259	biological _process	methylation	2	36	195	12977	1140	1.07144586	7.37E-05
GO:0043414	biological _process	macromolecule methylation	5	22	53	12977	1140	2.240362337	1.56E-09
GO:0070475	biological _process	rRNA base methylation	12	4	7	12977	1140	2.701496251	0.005535782
GO:0006355	biological _process	regulation of transcription, DNA-dependent	8	36	212	12977	1140	0.95085572	0.000393361
GO:0009889	biological _process	regulation of biosynthetic process	4	48	237	12977	1140	1.205070425	2.45E-07
GO:0010556	biological _process	regulation of macromolecule biosynthetic process	5	46	233	12977	1140	1.168226984	9.55E-07

GO:0019219	biological _process	regulation of nucleobase-containing compound metabolic process	5	39	261	12977	1140	0.766347395	0.002572604
GO:0031323	biological _process	regulation of cellular metabolic process	4	48	290	12977	1140	0.913904583	6.78E-05
GO:0031326	biological _process	regulation of cellular biosynthetic process	5	47	235	12977	1140	1.186923078	4.75E-07
GO:0051171	biological _process	regulation of nitrogen compound metabolic process	4	39	261	12977	1140	0.766347395	0.002572604
GO:0051252	biological _process	regulation of RNA metabolic process	6	36	212	12977	1140	0.95085572	0.000393361
GO:0080090	biological _process	regulation of primary metabolic process	4	49	311	12977	1140	0.842790247	0.000192225
GO:2000112	biological _process	regulation of cellular macromolecule biosynthetic process	6	46	233	12977	1140	1.168226984	9.55E-07
GO:2001141	biological _process	regulation of RNA biosynthetic process	7	36	212	12977	1140	0.95085572	0.000393361
GO:0006164	biological _process	purine nucleotide biosynthetic process	9	21	67	12977	1140	1.835079405	1.04E-06
GO:0009152	biological _process	purine ribonucleotide biosynthetic process	10	20	66	12977	1140	1.786385148	3.40E-06
GO:0015936	biological _process	coenzyme A metabolic process	10	7	13	12977	1140	2.615766377	0.000200448
GO:0015937	biological _process	coenzyme A biosynthetic process	11	6	8	12977	1140	3.093813673	5.80E-05
GO:0033866	biological _process	nucleoside bisphosphate biosynthetic process	8	6	8	12977	1140	3.093813673	5.80E-05
GO:0034030	biological _process	ribonucleoside bisphosphate biosynthetic process	9	6	8	12977	1140	3.093813673	5.80E-05
GO:0034033	biological _process	purine nucleoside bisphosphate biosynthetic process	9	6	8	12977	1140	3.093813673	5.80E-05
GO:0042451	biological	purine nucleoside	8	21	51	12977	1140	2.228743253	4.83E-09

	_process	biosynthetic process							
GO:0046129	biological _process	purine ribonucleoside biosynthetic process	9	21	51	12977	1140	2.228743253	4.83E-09
GO:0072522	biological _process	purine-containing compound biosynthetic process	5	22	73	12977	1140	1.778458232	1.14E-06
GO:0020037	molecular _function	heme binding	4	10	48	12977	1140	1.245816767	0.020618562
GO:0046906	molecular _function	tetrapyrrole binding	3	10	49	12977	1140	1.216069423	0.023642924
GO:0005506	molecular _function	iron ion binding	6	21	71	12977	1140	1.751421476	2.86E-06
GO:0030170	molecular _function	pyridoxal phosphate binding	4	17	70	12977	1140	1.467030997	0.000389065
GO:0019877	biological _process	diaminopimelate biosynthetic process	9	5	5	12977	1140	3.508851173	2.96E-05
GO:0046451	biological _process	diaminopimelate metabolic process	8	7	13	12977	1140	2.615766377	0.000200448
GO:0006553	biological _process	lysine metabolic process	9	7	14	12977	1140	2.508851173	0.000350044
GO:0009066	biological _process	aspartate family amino acid metabolic process	8	10	32	12977	1140	1.830779268	0.001114317
GO:0009067	biological _process	aspartate family amino acid biosynthetic process	9	8	28	12977	1140	1.701496251	0.007136688
GO:0009085	biological _process	lysine biosynthetic process	10	7	13	12977	1140	2.615766377	0.000200448
GO:0009089	biological _process	lysine biosynthetic process via diaminopimelate	11	7	13	12977	1140	2.615766377	0.000200448
GO:0047661	molecular _function	amino-acid racemase activity	6	3	8	12977	1140	2.093813673	0.059179072
GO:0016311	biological _process	dephosphorylation	5	9	40	12977	1140	1.356848079	0.01934326
GO:0016791	molecular _function	phosphatase activity	5	8	38	12977	1140	1.260923659	0.038675319
GO:0042578	molecular	phosphoric ester	4	10	56	12977	1140	1.023424346	0.050371631

	_function	hydrolase activity						
GO:0006644	biological _process	phospholipid metabolic process	5	20	37	12977	1140	2.621325902	2.97E-11
GO:0006650	biological _process	glycerophospholipid metabolic process	6	5	7	12977	1140	3.023424346	0.000409099
GO:0008654	biological _process	phospholipid biosynthetic process	6	19	35	12977	1140	2.627495669	9.35E-11
GO:0046486	biological _process	glycerolipid metabolic process	5	5	7	12977	1140	3.023424346	0.000409099
GO:0046434	biological _process	organophosphate catabolic process	5	6	13	12977	1140	2.393373955	0.001754878
GO:0006790	biological _process	sulfur compound metabolic process	3	16	50	12977	1140	1.864994983	1.84E-05
GO:0044272	biological _process	sulfur compound biosynthetic process	4	13	37	12977	1140	1.999837525	4.57E-05
GO:0006353	biological _process	DNA-dependent transcription, termination	9	4	5	12977	1140	3.186923078	0.001096945
GO:0009890	biological _process	negative regulation of biosynthetic process	5	4	9	12977	1140	2.338926171	0.015264401
GO:0009892	biological _process	negative regulation of metabolic process	4	4	10	12977	1140	2.186923078	0.020984057
GO:0010558	biological _process	negative regulation of macromolecule biosynthetic process	6	4	9	12977	1140	2.338926171	0.015264401
GO:0010605	biological _process	negative regulation of macromolecule metabolic process	5	4	10	12977	1140	2.186923078	0.020984057
GO:0031324	biological _process	negative regulation of cellular metabolic process	5	4	10	12977	1140	2.186923078	0.020984057
GO:0031327	biological _process	negative regulation of cellular biosynthetic process	6	4	9	12977	1140	2.338926171	0.015264401
GO:0045934	biological _process	negative regulation of nucleobase-containing compound metabolic	6	3	8	12977	1140	2.093813673	0.059179072

		process							
GO:0048519	biological _process	negative regulation of biological process	3	4	13	12977	1140	1.808411455	0.049501873
GO:0048523	biological _process	negative regulation of cellular process	4	4	13	12977	1140	1.808411455	0.049501873
GO:0051172	biological _process	negative regulation of nitrogen compound metabolic process	5	3	8	12977	1140	2.093813673	0.059179072
GO:2000113	biological _process	negative regulation of cellular macromolecule biosynthetic process	7	4	9	12977	1140	2.338926171	0.015264401
GO:0030246	molecular _function	carbohydrate binding	2	5	20	12977	1140	1.508851173	0.058475892
GO:0008483	molecular _function	transaminase activity	4	9	37	12977	1140	1.469322808	0.011725054
GO:0016769	molecular _function	transferase activity, transferring nitrogenous groups	3	10	39	12977	1140	1.545377049	0.005335546
GO:0016209	molecular _function	antioxidant activity	1	9	33	12977	1140	1.634382055	0.00553806
GO:0052689	molecular _function	carboxylic ester hydrolase activity	4	6	29	12977	1140	1.235832678	0.080434319
GO:0090502	biological _process	RNA phosphodiester bond hydrolysis, endonucleolytic	8	5	11	12977	1140	2.371347649	0.005197577
GO:0004519	molecular _function	endonuclease activity	5	16	97	12977	1140	0.90893833	0.025838952
GO:0004521	molecular _function	endoribonuclease activity	6	5	11	12977	1140	2.371347649	0.005197577
GO:0006418	biological _process	tRNA aminoacylation for protein translation	10	22	36	12977	1140	2.79835779	8.02E-14
GO:0043038	biological _process	amino acid activation	8	22	37	12977	1140	2.758829426	1.75E-13
GO:0043039	biological _process	tRNA aminoacylation	9	22	37	12977	1140	2.758829426	1.75E-13
GO:0004812	molecular _function	aminoacyl-tRNA ligase activity	5	22	37	12977	1140	2.758829426	1.75E-13

GO:0016875	molecular _function	ligase activity, forming carbon-oxygen bonds	3	22	38	12977	1140	2.720355278	3.74E-13
GO:0016876	molecular _function	ligase activity, forming aminoacyl-tRNA and related compounds	4	22	38	12977	1140	2.720355278	3.74E-13
GO:0019104	molecular _function	DNA N-glycosylase activity	5	3	9	12977	1140	1.923888672	0.08055066
GO:0006284	biological _process	base-excision repair	8	3	9	12977	1140	1.923888672	0.08055066
GO:0051536	molecular _function	iron-sulfur cluster binding	3	22	72	12977	1140	1.79835779	8.93E-07
GO:0051539	molecular _function	4 iron, 4 sulfur cluster binding	4	12	26	12977	1140	2.393373955	3.92E-06
GO:0051540	molecular _function	metal cluster binding	2	22	72	12977	1140	1.79835779	8.93E-07
GO:0006643	biological _process	membrane lipid metabolic process	5	10	19	12977	1140	2.582851754	7.18E-06
GO:0006664	biological _process	glycolipid metabolic process	6	10	17	12977	1140	2.743316426	2.02E-06
GO:0009245	biological _process	lipid A biosynthetic process	8	10	17	12977	1140	2.743316426	2.02E-06
GO:0009247	biological _process	glycolipid biosynthetic process	7	10	17	12977	1140	2.743316426	2.02E-06
GO:0009311	biological _process	oligosaccharide metabolic process	5	10	26	12977	1140	2.130339549	0.000181893
GO:0009312	biological _process	oligosaccharide biosynthetic process	6	10	24	12977	1140	2.245816767	8.49E-05
GO:0046467	biological _process	membrane lipid biosynthetic process	6	10	18	12977	1140	2.660854266	3.92E-06
GO:0046493	biological _process	lipid A metabolic process	7	10	17	12977	1140	2.743316426	2.02E-06
GO:1901269	biological _process	lipooligosaccharide metabolic process	6	10	17	12977	1140	2.743316426	2.02E-06
GO:1901271	biological _process	lipooligosaccharide biosynthetic process	7	10	17	12977	1140	2.743316426	2.02E-06
GO:0006040	biological _process	amino sugar metabolic process	4	4	14	12977	1140	1.701496251	0.063270064

GO:0009226	biological _process	nucleotide-sugar biosynthetic process	9	4	7	12977	1140	2.701496251	0.005535782
GO:0046349	biological _process	amino sugar biosynthetic process	5	3	5	12977	1140	2.771885579	0.016850698
GO:0000902	biological _process	cell morphogenesis	5	3	5	12977	1140	2.771885579	0.016850698
GO:0009653	biological _process	anatomical structure morphogenesis	3	3	6	12977	1140	2.508851173	0.027997418
GO:0032502	biological _process	developmental process	1	3	7	12977	1140	2.286458751	0.043326175
GO:0032989	biological _process	cellular component morphogenesis	4	3	5	12977	1140	2.771885579	0.016850698
GO:0044767	biological _process	single-organism developmental process	2	3	7	12977	1140	2.286458751	0.043326175
GO:0048856	biological _process	anatomical structure development	2	3	7	12977	1140	2.286458751	0.043326175
GO:0048869	biological _process	cellular developmental process	3	3	6	12977	1140	2.508851173	0.027997418
GO:0009200	biological _process	deoxyribonucleoside triphosphate metabolic process	8	4	5	12977	1140	3.186923078	0.001096945
GO:0009211	biological _process	pyrimidine deoxyribonucleoside triphosphate metabolic process	9	4	5	12977	1140	3.186923078	0.001096945
GO:0009219	biological _process	pyrimidine deoxyribonucleotide metabolic process	10	4	5	12977	1140	3.186923078	0.001096945
GO:0009221	biological _process	pyrimidine deoxyribonucleotide biosynthetic process	11	4	5	12977	1140	3.186923078	0.001096945
GO:0009262	biological _process	deoxyribonucleotide metabolic process	8	6	7	12977	1140	3.286458751	1.73E-05
GO:0009263	biological _process	deoxyribonucleotide biosynthetic process	9	4	5	12977	1140	3.186923078	0.001096945
GO:0009265	biological _process	2'-deoxyribonucleotide biosynthetic process	10	4	5	12977	1140	3.186923078	0.001096945

GO:0009394	biological _process	2'-deoxyribonucleotide metabolic process	9	4	5	12977	1140	3.186923078	0.001096945
GO:0019692	biological _process	deoxyribose phosphate metabolic process	5	5	6	12977	1140	3.245816767	0.000142659
GO:0046385	biological _process	deoxyribose phosphate biosynthetic process	6	4	5	12977	1140	3.186923078	0.001096945
GO:0009162	biological _process	deoxyribonucleoside monophosphate metabolic process	8	3	5	12977	1140	2.771885579	0.016850698
GO:0003747	molecular _function	translation release factor activity	7	4	5	12977	1140	3.186923078	0.001096945
GO:0008079	molecular _function	translation termination factor activity	6	4	5	12977	1140	3.186923078	0.001096945
GO:0006415	biological _process	translational termination	7	5	6	12977	1140	3.245816767	0.000142659
GO:0022411	biological _process	cellular component disassembly	3	5	6	12977	1140	3.245816767	0.000142659
GO:0032984	biological _process	macromolecular complex disassembly	4	5	6	12977	1140	3.245816767	0.000142659
GO:0043241	biological _process	protein complex disassembly	5	5	6	12977	1140	3.245816767	0.000142659
GO:0043624	biological _process	cellular protein complex disassembly	6	5	6	12977	1140	3.245816767	0.000142659
GO:0043933	biological _process	macromolecular complex subunit organization	3	18	27	12977	1140	2.923888672	2.50E-12
GO:0071822	biological _process	protein complex subunit organization	4	15	24	12977	1140	2.830779268	8.21E-10
GO:0030488	biological _process	tRNA methylation	11	3	6	12977	1140	2.508851173	0.027997418
GO:0008175	molecular _function	tRNA methyltransferase activity	6	4	7	12977	1140	2.701496251	0.005535782
GO:0006417	biological _process	regulation of translation	7	10	20	12977	1140	2.508851173	1.28E-05
GO:0032268	biological _process	regulation of cellular protein metabolic process	6	10	30	12977	1140	1.923888672	0.000655405

GO:0051246	biological _process	regulation of protein metabolic process	5	10	30	12977	1140	1.923888672	0.000655405
GO:0003924	molecular _function	GTPase activity	7	14	30	12977	1140	2.409315499	4.45E-07
GO:0008324	molecular _function	cation transmembrane transporter activity	5	21	159	12977	1140	0.58828564	0.081515821
GO:0015077	molecular _function	monovalent inorganic cation transmembrane transporter activity	7	18	95	12977	1140	1.108920566	0.004808997
GO:0022890	molecular _function	inorganic cation transmembrane transporter activity	6	19	118	12977	1140	0.874135637	0.019748914
GO:0003887	molecular _function	DNA-directed DNA polymerase activity	6	9	30	12977	1140	1.771885579	0.002848916
GO:0034061	molecular _function	DNA polymerase activity	5	9	45	12977	1140	1.186923078	0.036865819
GO:0071897	biological _process	DNA biosynthetic process	7	9	32	12977	1140	1.678776174	0.004556084
GO:0006749	biological _process	glutathione metabolic process	9	4	9	12977	1140	2.338926171	0.015264401
GO:0016667	molecular _function	oxidoreductase activity, acting on a sulfur group of donors	3	12	32	12977	1140	2.093813673	4.68E-05
GO:0016668	molecular _function	oxidoreductase activity, acting on a sulfur group of donors, NAD(P) as acceptor	4	4	14	12977	1140	1.701496251	0.063270064
GO:0045454	biological _process	cell redox homeostasis	5	7	24	12977	1140	1.731243594	0.010932801
GO:0015036	molecular _function	disulfide oxidoreductase activity	4	8	14	12977	1140	2.701496251	3.57E-05
GO:0016831	molecular _function	carboxy-lyase activity	4	11	38	12977	1140	1.720355278	0.001178371
GO:0043190	cellular_c omponent	ATP-binding cassette (ABC) transporter complex	3	11	19	12977	1140	2.720355278	6.92E-07
GO:0006206	biological	pyrimidine nucleobase	7	5	10	12977	1140	2.508851173	0.003183723

	_process	metabolic process						
GO:0006207	biological _process	'de novo' pyrimidine nucleobase biosynthetic process	9	4	5	12977	1140	3.186923078	0.001096945
GO:0009112	biological _process	nucleobase metabolic process	6	5	18	12977	1140	1.660854266	0.041196971
GO:0019856	biological _process	pyrimidine nucleobase biosynthetic process	8	5	6	12977	1140	3.245816767	0.000142659
GO:0046112	biological _process	nucleobase biosynthetic process	7	5	11	12977	1140	2.371347649	0.005197577
GO:0044205	biological _process	'de novo' UMP biosynthetic process	12	7	8	12977	1140	3.316206095	2.04E-06
GO:0008171	molecular _function	O-methyltransferase activity	5	6	20	12977	1140	1.771885579	0.017070369
GO:0008276	molecular _function	protein methyltransferase activity	5	5	14	12977	1140	2.023424346	0.015444873
GO:0006479	biological _process	protein methylation	8	5	15	12977	1140	1.923888672	0.020262755
GO:0008213	biological _process	protein alkylation	7	5	15	12977	1140	1.923888672	0.020262755
GO:0016408	molecular _function	C-acyltransferase activity	5	3	8	12977	1140	2.093813673	0.059179072
GO:0006547	biological _process	histidine metabolic process	9	13	24	12977	1140	2.62432839	1.48E-07
GO:0006548	biological _process	histidine catabolic process	10	3	9	12977	1140	1.923888672	0.08055066
GO:0009075	biological _process	histidine family amino acid metabolic process	8	13	24	12977	1140	2.62432839	1.48E-07
GO:0009077	biological _process	histidine family amino acid catabolic process	9	3	9	12977	1140	1.923888672	0.08055066
GO:0015942	biological _process	formate metabolic process	8	3	8	12977	1140	2.093813673	0.059179072
GO:0019439	biological _process	aromatic compound catabolic process	4	17	73	12977	1140	1.406489455	0.000649824
GO:0019557	biological _process	histidine catabolic process to glutamate	11	3	8	12977	1140	2.093813673	0.059179072

		and formate							
GO:0044270	biological _process	cellular nitrogen compound catabolic process	4	15	68	12977	1140	1.328278927	0.002504848
GO:0046700	biological _process	heterocycle catabolic process	4	16	71	12977	1140	1.359104053	0.001381114
GO:0052803	biological _process	imidazole-containing compound metabolic process	4	13	24	12977	1140	2.62432839	1.48E-07
GO:0052805	biological _process	imidazole-containing compound catabolic process	5	3	9	12977	1140	1.923888672	0.08055066
GO:1901361	biological _process	organic cyclic compound catabolic process	4	18	75	12977	1140	1.449957484	0.000294749
GO:0019556	biological _process	histidine catabolic process to glutamate and formamide	11	3	9	12977	1140	1.923888672	0.08055066
GO:0043606	biological _process	formamide metabolic process	5	3	9	12977	1140	1.923888672	0.08055066
GO:0006448	biological _process	regulation of translational elongation	8	4	10	12977	1140	2.186923078	0.020984057
GO:0006450	biological _process	regulation of translational fidelity	9	4	10	12977	1140	2.186923078	0.020984057
GO:0002161	molecular _function	aminoacyl-tRNA editing activity	5	3	9	12977	1140	1.923888672	0.08055066
GO:0016840	molecular _function	carbon-nitrogen lyase activity	3	4	13	12977	1140	1.808411455	0.049501873
GO:0044444	cellular_c omponent	cytoplasmic part	6	63	125	12977	1140	2.520346812	2.47E-32
GO:0003989	molecular _function	acetyl-CoA carboxylase activity	5	3	5	12977	1140	2.771885579	0.016850698
GO:0016421	molecular _function	CoA carboxylase activity	4	3	7	12977	1140	2.286458751	0.043326175
GO:0016885	molecular _function	ligase activity, forming carbon-carbon bonds	3	3	9	12977	1140	1.923888672	0.08055066
GO:0016780	molecular _function	phosphotransferase activity, for other	4	5	13	12977	1140	2.130339549	0.011011531

		substituted phosphate groups						
GO:0006401	biological _process	RNA catabolic process	7	6	28	12977	1140	1.286458751	0.06931593
GO:0034655	biological _process	nucleobase-containing compound catabolic process	5	12	44	12977	1140	1.634382055	0.001173122
GO:0044265	biological _process	cellular macromolecule catabolic process	5	11	40	12977	1140	1.646354696	0.001875238
GO:0016782	molecular _function	transferase activity, transferring sulfur-containing groups	3	6	17	12977	1140	2.006350832	0.007830073
GO:0016783	molecular _function	sulfurtransferase activity	4	6	13	12977	1140	2.393373955	0.001754878
GO:0006720	biological _process	isoprenoid metabolic process	5	4	13	12977	1140	1.808411455	0.049501873
GO:0008299	biological _process	isoprenoid biosynthetic process	6	4	13	12977	1140	1.808411455	0.049501873
GO:0006066	biological _process	alcohol metabolic process	4	4	13	12977	1140	1.808411455	0.049501873
GO:0006071	biological _process	glycerol metabolic process	7	3	5	12977	1140	2.771885579	0.016850698
GO:0019400	biological _process	alditol metabolic process	6	3	5	12977	1140	2.771885579	0.016850698
GO:0019751	biological _process	polyol metabolic process	5	4	11	12977	1140	2.049419554	0.029792641
GO:1901615	biological _process	organic hydroxy compound metabolic process	3	5	15	12977	1140	1.923888672	0.020262755
GO:1901292	biological _process	nucleoside phosphate catabolic process	7	3	8	12977	1140	2.093813673	0.059179072
GO:0034622	biological _process	cellular macromolecular complex assembly	5	11	17	12977	1140	2.88081995	1.48E-07
GO:0065003	biological _process	macromolecular complex assembly	4	13	21	12977	1140	2.816973468	1.78E-08
GO:0016891	molecular _function	endoribonuclease activity, producing 5'-	7	3	6	12977	1140	2.508851173	0.027997418

		phosphomonoesters							
GO:0016893	molecular _function	endonuclease activity, active with either ribo- or deoxyribonucleic acids and producing 5'-phosphomonoesters	6	4	12	12977	1140	1.923888672	0.040422466
GO:0009084	biological _process	glutamine family amino acid biosynthetic process	9	8	25	12977	1140	1.864994983	0.003401109
GO:0015627	cellular_c omponent	type II protein secretion system complex	3	6	13	12977	1140	2.393373955	0.001754878
GO:0015628	biological _process	protein secretion by the type II secretion system	8	6	13	12977	1140	2.393373955	0.001754878
GO:0003735	molecular _function	structural constituent of ribosome	2	49	58	12977	1140	3.265580022	1.39E-41
GO:0005198	molecular _function	structural molecule activity	1	51	68	12977	1140	3.093813673	1.76E-38
GO:0030529	cellular_c omponent	ribonucleoprotein complex	5	51	73	12977	1140	2.991451956	6.31E-36
GO:0005840	cellular_c omponent	ribosome	7	50	71	12977	1140	3.002960243	1.68E-35
GO:0019843	molecular _function	rRNA binding	5	37	43	12977	1140	3.292039784	4.86E-32
GO:0004520	molecular _function	endodeoxyribonuclease activity	6	5	13	12977	1140	2.130339549	0.011011531
GO:0004536	molecular _function	deoxyribonuclease activity	5	7	19	12977	1140	2.068278581	0.002802318
GO:0009381	molecular _function	excinuclease ABC activity	7	3	5	12977	1140	2.771885579	0.016850698
GO:0006289	biological _process	nucleotide-excision repair	8	4	13	12977	1140	1.808411455	0.049501873
GO:0043021	molecular _function	ribonucleoprotein complex binding	2	3	8	12977	1140	2.093813673	0.059179072
GO:0043022	molecular _function	ribosome binding	3	3	8	12977	1140	2.093813673	0.059179072
GO:0016226	biological _process	iron-sulfur cluster assembly	5	6	6	12977	1140	3.508851173	3.05E-06

GO:0031163	biological _process	metallo-sulfur cluster assembly	4	6	6	12977	1140	3.508851173	3.05E-06
GO:0006525	biological _process	arginine metabolic process	9	9	16	12977	1140	2.678776174	1.17E-05
GO:0006526	biological _process	arginine biosynthetic process	10	6	10	12977	1140	2.771885579	0.000313267
GO:0016884	molecular _function	carbon-nitrogen ligase activity, with glutamine as amido-N-donor	4	7	30	12977	1140	1.409315499	0.033206109
GO:0003684	molecular _function	damaged DNA binding	5	4	15	12977	1140	1.601960577	0.07957662
GO:0030163	biological _process	protein catabolic process	5	5	14	12977	1140	2.023424346	0.015444873
GO:0006778	biological _process	porphyrin-containing compound metabolic process	5	11	32	12977	1140	1.968282791	0.000244224
GO:0006779	biological _process	porphyrin-containing compound biosynthetic process	6	10	29	12977	1140	1.972798272	0.000483766
GO:0006783	biological _process	heme biosynthetic process	7	7	21	12977	1140	1.923888672	0.005206757
GO:0033013	biological _process	tetrapyrrole metabolic process	4	12	34	12977	1140	2.006350832	9.07E-05
GO:0033014	biological _process	tetrapyrrole biosynthetic process	5	11	31	12977	1140	2.014086481	0.000180591
GO:0042168	biological _process	heme metabolic process	6	8	24	12977	1140	1.923888672	0.002598833
GO:0042440	biological _process	pigment metabolic process	3	9	33	12977	1140	1.634382055	0.00553806
GO:0046148	biological _process	pigment biosynthetic process	4	8	30	12977	1140	1.601960577	0.010637465
GO:0006782	biological _process	protoporphyrinogen IX biosynthetic process	8	4	15	12977	1140	1.601960577	0.07957662
GO:0046501	biological _process	protoporphyrinogen IX metabolic process	6	4	15	12977	1140	1.601960577	0.07957662
GO:0016435	molecular _function	rRNA (guanine) methyltransferase	7	5	6	12977	1140	3.245816767	0.000142659

		activity							
GO:0015078	molecular _function	hydrogen ion transmembrane transporter activity	8	15	66	12977	1140	1.371347649	0.00186774
GO:0015980	biological _process	energy derivation by oxidation of organic compounds	4	18	51	12977	1140	2.006350832	1.06E-06
GO:0022900	biological _process	electron transport chain	4	9	24	12977	1140	2.093813673	0.000506538
GO:0022904	biological _process	respiratory electron transport chain	6	6	18	12977	1140	1.923888672	0.010193637
GO:0045333	biological _process	cellular respiration	5	18	50	12977	1140	2.034919984	7.79E-07
GO:0009060	biological _process	aerobic respiration	6	13	34	12977	1140	2.12182805	1.65E-05
GO:0070469	cellular_c omponent	respiratory chain	3	4	9	12977	1140	2.338926171	0.015264401
GO:0015935	cellular_c omponent	small ribosomal subunit	9	6	7	12977	1140	3.286458751	1.73E-05
GO:0044391	cellular_c omponent	ribosomal subunit	8	12	15	12977	1140	3.186923078	7.37E-10
GO:0044422	cellular_c omponent	organelle part	2	12	62	12977	1140	1.139617363	0.01978985
GO:0044446	cellular_c omponent	intracellular organelle part	6	12	34	12977	1140	2.006350832	9.07E-05
GO:0015934	cellular_c omponent	large ribosomal subunit	9	6	8	12977	1140	3.093813673	5.80E-05
GO:0051205	biological _process	protein insertion into membrane	6	5	6	12977	1140	3.245816767	0.000142659
GO:0051668	biological _process	localization within membrane	4	5	6	12977	1140	3.245816767	0.000142659
GO:0000105	biological _process	histidine biosynthetic process	10	10	15	12977	1140	2.923888672	4.12E-07
GO:0009076	biological _process	histidine family amino acid biosynthetic process	9	10	15	12977	1140	2.923888672	4.12E-07
GO:0015939	biological	pantothenate metabolic	9	3	6	12977	1140	2.508851173	0.027997418

	_process	process							
GO:0010181	molecular _function	FMN binding	6	6	24	12977	1140	1.508851173	0.038029961
GO:0016624	molecular _function	oxidoreductase activity, acting on the aldehyde or oxo group of donors, disulfide as acceptor	4	3	8	12977	1140	2.093813673	0.059179072
GO:0016903	molecular _function	oxidoreductase activity, acting on the aldehyde or oxo group of donors	3	11	42	12977	1140	1.575965369	0.002782239
GO:0006413	biological _process	translational initiation	7	4	6	12977	1140	2.923888672	0.002782239
GO:0006461	biological _process	protein complex assembly	5	10	18	12977	1140	2.660854266	3.92E-06
GO:0017004	biological _process	cytochrome complex assembly	7	7	13	12977	1140	2.615766377	0.000200448
GO:0043623	biological _process	cellular protein complex assembly	6	8	14	12977	1140	2.701496251	3.57E-05
GO:0070271	biological _process	protein complex biogenesis	3	10	18	12977	1140	2.660854266	3.92E-06
GO:0006733	biological _process	oxidoreduction coenzyme metabolic process	5	16	35	12977	1140	2.379568156	8.28E-08
GO:0006739	biological _process	NADP metabolic process	10	3	8	12977	1140	2.093813673	0.059179072
GO:0019362	biological _process	pyridine nucleotide metabolic process	8	7	20	12977	1140	1.994278	0.003856469
GO:0046496	biological _process	nicotinamide nucleotide metabolic process	9	7	20	12977	1140	1.994278	0.003856469
GO:0015748	biological _process	organophosphate ester transport	5	3	5	12977	1140	2.771885579	0.016850698
GO:0042954	molecular _function	lipoprotein transporter activity	4	3	5	12977	1140	2.771885579	0.016850698
GO:0042953	biological _process	lipoprotein transport	6	3	5	12977	1140	2.771885579	0.016850698
GO:0001522	biological _process	pseudouridine synthesis	8	6	9	12977	1140	2.923888672	0.000148189

GO:0009982	molecular _function	pseudouridine synthase activity	4	6	8	12977	1140	3.093813673	5.80E-05
GO:0016866	molecular _function	intramolecular transferase activity	3	8	30	12977	1140	1.601960577	0.010637465
GO:0009309	biological _process	amine biosynthetic process	5	8	17	12977	1140	2.421388331	0.000196277
GO:0016620	molecular _function	oxidoreductase activity, acting on the aldehyde or oxo group of donors, NAD or NADP as acceptor	4	8	29	12977	1140	1.650870178	0.008660364
GO:0015035	molecular _function	protein disulfide oxidoreductase activity	5	6	12	12977	1140	2.508851173	0.001078378
GO:0006072	biological _process	glycerol-3-phosphate metabolic process	6	3	7	12977	1140	2.286458751	0.043326175
GO:0052646	biological _process	alditol phosphate metabolic process	5	3	7	12977	1140	2.286458751	0.043326175
GO:0004527	molecular _function	exonuclease activity	5	13	48	12977	1140	1.62432839	0.000763119
GO:0016796	molecular _function	exonuclease activity, active with either ribo- or deoxyribonucleic acids and producing 5'-phosphomonoesters	6	6	10	12977	1140	2.771885579	0.000313267
GO:0006308	biological _process	DNA catabolic process	7	3	5	12977	1140	2.771885579	0.016850698
GO:0006768	biological _process	biotin metabolic process	8	4	5	12977	1140	3.186923078	0.001096945
GO:0009102	biological _process	biotin biosynthetic process	9	4	5	12977	1140	3.186923078	0.001096945
GO:0009266	biological _process	response to temperature stimulus	3	3	7	12977	1140	2.286458751	0.043326175
GO:0009628	biological _process	response to abiotic stimulus	2	3	9	12977	1140	1.923888672	0.08055066
GO:0006099	biological _process	tricarboxylic acid cycle	7	10	26	12977	1140	2.130339549	0.000181893
GO:0006352	biological	DNA-dependent	9	5	11	12977	1140	2.371347649	0.005197577

	_process	transcription, initiation						
GO:0000988	molecular _function	protein binding transcription factor activity	1	5	10	12977	1140	2.508851173	0.003183723
GO:0000990	molecular _function	core RNA polymerase binding transcription factor activity	2	5	10	12977	1140	2.508851173	0.003183723
GO:0000996	molecular _function	core DNA-dependent RNA polymerase binding promoter specificity activity	3	5	10	12977	1140	2.508851173	0.003183723
GO:0016987	molecular _function	sigma factor activity	4	5	10	12977	1140	2.508851173	0.003183723
GO:0006298	biological _process	mismatch repair	8	3	5	12977	1140	2.771885579	0.016850698
GO:0009065	biological _process	glutamine family amino acid catabolic process	9	4	12	12977	1140	1.923888672	0.040422466
GO:0006743	biological _process	ubiquinone metabolic process	6	9	15	12977	1140	2.771885579	5.84E-06
GO:0006744	biological _process	ubiquinone biosynthetic process	7	9	15	12977	1140	2.771885579	5.84E-06
GO:0042180	biological _process	cellular ketone metabolic process	4	9	21	12977	1140	2.286458751	0.000162849
GO:0042181	biological _process	ketone biosynthetic process	5	9	16	12977	1140	2.678776174	1.17E-05
GO:0042375	biological _process	quinone cofactor metabolic process	4	9	15	12977	1140	2.771885579	5.84E-06
GO:0045426	biological _process	quinone cofactor biosynthetic process	6	9	15	12977	1140	2.771885579	5.84E-06
GO:1901661	biological _process	quinone metabolic process	5	9	15	12977	1140	2.771885579	5.84E-06
GO:1901663	biological _process	quinone biosynthetic process	6	9	15	12977	1140	2.771885579	5.84E-06
GO:0008169	molecular _function	C-methyltransferase activity	5	3	6	12977	1140	2.508851173	0.027997418
GO:0006527	biological _process	arginine catabolic process	10	3	6	12977	1140	2.508851173	0.027997418

GO:0016832	molecular _function	aldehyde-lyase activity	4	4	8	12977	1140	2.508851173	0.009538302
GO:0016833	molecular _function	oxo-acid-lyase activity	4	4	10	12977	1140	2.186923078	0.020984057
GO:0046912	molecular _function	transferase activity, transferring acyl groups, acyl groups converted into alkyl on transfer	4	3	7	12977	1140	2.286458751	0.043326175
GO:0006167	biological _process	AMP biosynthetic process	11	3	6	12977	1140	2.508851173	0.027997418
GO:0009126	biological _process	purine nucleoside monophosphate metabolic process	8	6	23	12977	1140	1.570251717	0.031135247
GO:0009127	biological _process	purine nucleoside monophosphate biosynthetic process	9	6	23	12977	1140	1.570251717	0.031135247
GO:0009167	biological _process	purine ribonucleoside monophosphate metabolic process	9	6	23	12977	1140	1.570251717	0.031135247
GO:0009168	biological _process	purine ribonucleoside monophosphate biosynthetic process	10	6	23	12977	1140	1.570251717	0.031135247
GO:0046033	biological _process	AMP metabolic process	10	3	6	12977	1140	2.508851173	0.027997418
GO:0009435	biological _process	NAD biosynthetic process	11	4	11	12977	1140	2.049419554	0.029792641
GO:0019359	biological _process	nicotinamide nucleotide biosynthetic process	10	5	12	12977	1140	2.245816767	0.007834784
GO:0019363	biological _process	pyridine nucleotide biosynthetic process	9	5	12	12977	1140	2.245816767	0.007834784
GO:0019674	biological _process	NAD metabolic process	10	5	12	12977	1140	2.245816767	0.007834784
GO:0000162	biological _process	tryptophan biosynthetic process	10	4	7	12977	1140	2.701496251	0.005535782
GO:0006568	biological _process	tryptophan metabolic process	9	4	13	12977	1140	1.808411455	0.049501873
GO:0006576	biological	cellular biogenic amine	6	6	15	12977	1140	2.186923078	0.003944172

	_process	metabolic process							
GO:0006586	biological _process	indolalkylamine metabolic process	7	4	13	12977	1140	1.808411455	0.049501873
GO:0042401	biological _process	cellular biogenic amine biosynthetic process	7	6	9	12977	1140	2.923888672	0.000148189
GO:0042430	biological _process	indole-containing compound metabolic process	4	4	13	12977	1140	1.808411455	0.049501873
GO:0042435	biological _process	indole-containing compound biosynthetic process	5	4	7	12977	1140	2.701496251	0.005535782
GO:0044106	biological _process	cellular amine metabolic process	5	6	18	12977	1140	1.923888672	0.010193637
GO:0046219	biological _process	indolalkylamine biosynthetic process	8	4	7	12977	1140	2.701496251	0.005535782
GO:0015232	molecular _function	heme transporter activity	3	4	7	12977	1140	2.701496251	0.005535782
GO:0051184	molecular _function	cofactor transporter activity	2	4	8	12977	1140	2.508851173	0.009538302
GO:0015886	biological _process	heme transport	6	4	8	12977	1140	2.508851173	0.009538302
GO:0051181	biological _process	cofactor transport	5	4	9	12977	1140	2.338926171	0.015264401
GO:1901678	biological _process	iron coordination entity transport	5	4	12	12977	1140	1.923888672	0.040422466
GO:0008408	molecular _function	3'-5' exonuclease activity	6	5	13	12977	1140	2.130339549	0.011011531
GO:0016877	molecular _function	ligase activity, forming carbon-sulfur bonds	3	5	10	12977	1140	2.508851173	0.003183723
GO:0051726	biological _process	regulation of cell cycle	4	3	9	12977	1140	1.923888672	0.08055066
GO:0000917	biological _process	barrier septum assembly	8	8	12	12977	1140	2.923888672	7.52E-06
GO:0032506	biological _process	cytokinetic process	6	8	12	12977	1140	2.923888672	7.52E-06
GO:0090529	biological _process	cell septum assembly	7	8	12	12977	1140	2.923888672	7.52E-06

GO:0009144	biological _process	purine nucleoside triphosphate metabolic process	8	9	37	12977	1140	1.469322808	0.011725054
GO:0009145	biological _process	purine nucleoside triphosphate biosynthetic process	9	9	31	12977	1140	1.724579864	0.003621545
GO:0009205	biological _process	purine ribonucleoside triphosphate metabolic process	9	9	37	12977	1140	1.469322808	0.011725054
GO:0009206	biological _process	purine ribonucleoside triphosphate biosynthetic process	10	9	31	12977	1140	1.724579864	0.003621545
GO:0006754	biological _process	ATP biosynthetic process	11	8	30	12977	1140	1.601960577	0.010637465
GO:0006818	biological _process	hydrogen transport	5	8	37	12977	1140	1.299397807	0.033206109
GO:0015985	biological _process	energy coupled proton transport, down electrochemical gradient	9	8	29	12977	1140	1.650870178	0.008660364
GO:0015986	biological _process	ATP synthesis coupled proton transport	12	8	29	12977	1140	1.650870178	0.008660364
GO:0015992	biological _process	proton transport	8	8	37	12977	1140	1.299397807	0.033206109
GO:0046034	biological _process	ATP metabolic process	10	8	32	12977	1140	1.508851173	0.015679127
GO:0016469	cellular_c omponent	proton-transporting two-sector ATPase complex	3	8	32	12977	1140	1.508851173	0.015679127
GO:0033178	cellular_c omponent	proton-transporting two-sector ATPase complex, catalytic domain	4	5	19	12977	1140	1.582851754	0.049139564
GO:0045259	cellular_c omponent	proton-transporting ATP synthase complex	5	8	27	12977	1140	1.753963671	0.00554338
GO:0045261	cellular_c omponent	proton-transporting ATP synthase complex, catalytic core F(1)	6	5	16	12977	1140	1.830779268	0.025564747

GO:0044769	molecular _function	ATPase activity, coupled to transmembrane movement of ions, rotational mechanism	12	7	27	12977	1140	1.561318593	0.020262755
GO:0046933	molecular _function	proton-transporting ATP synthase activity, rotational mechanism	13	7	26	12977	1140	1.615766377	0.016850698
GO:0042777	biological _process	plasma membrane ATP synthesis coupled proton transport	13	7	17	12977	1140	2.228743253	0.001378086
GO:0051052	biological _process	regulation of DNA metabolic process	6	3	7	12977	1140	2.286458751	0.043326175
GO:0043566	molecular _function	structure-specific DNA binding	5	5	17	12977	1140	1.743316426	0.032772471
GO:0004532	molecular _function	exoribonuclease activity	6	4	6	12977	1140	2.923888672	0.002782239
GO:0016896	molecular _function	exoribonuclease activity, producing 5'-phosphomonoesters	7	4	6	12977	1140	2.923888672	0.002782239
GO:0090503	biological _process	RNA phosphodiester bond hydrolysis, exonucleolytic	8	4	6	12977	1140	2.923888672	0.002782239
GO:0016878	molecular _function	acid-thiol ligase activity	4	4	6	12977	1140	2.923888672	0.002782239
GO:0006119	biological _process	oxidative phosphorylation	6	5	12	12977	1140	2.245816767	0.007834784
GO:0042773	biological _process	ATP synthesis coupled electron transport	7	5	12	12977	1140	2.245816767	0.007834784
GO:0003954	molecular _function	NADH dehydrogenase activity	4	11	17	12977	1140	2.88081995	1.48E-07
GO:0008137	molecular _function	NADH dehydrogenase (ubiquinone) activity	6	7	13	12977	1140	2.615766377	0.000200448
GO:0050136	molecular _function	NADH dehydrogenase (quinone) activity	5	11	17	12977	1140	2.88081995	1.48E-07
GO:0048038	molecular _function	quinone binding	3	6	16	12977	1140	2.093813673	0.005540491
**Supplementary Table 2b. Results of the GO term enrichment analysis of all the accessory genes. Performed**									

hypergeometric probability distribution Test									
**GOID**	**Ontology**	**Term**	**Level**	**q**	**m**	**t**	**k**	**log_odds_ratio**	**p**
GO:0004803	molecular _function	transposase activity	2	131	131	12977	1183 8	0.13253154	0.006004821
GO:0006313	biological _process	transposition, DNA-mediated	8	132	132	12977	1183 8	0.13253154	0.006004821
GO:0032196	biological _process	transposition	3	132	132	12977	1183 8	0.13253154	0.006004821
GO:0007165	biological _process	signal transduction	4	299	308	12977	1183 8	0.089746674	0.014509967
GO:0023052	biological _process	signaling	1	299	308	12977	1183 8	0.089746674	0.014509967
GO:0044700	biological _process	single organism signaling	2	299	308	12977	1183 8	0.089746674	0.014509967
GO:0004871	molecular _function	signal transducer activity	2	189	193	12977	1183 8	0.102316927	0.039897051
GO:0060089	molecular _function	molecular transducer activity	1	189	193	12977	1183 8	0.102316927	0.039897051
GO:0015074	biological _process	DNA integration	7	133	134	12977	1183 8	0.121724785	0.027346048

Supplementary Table 3. Unique gene families/clusters identified in each of the 4 *Legionella* clades. (**a**) Unique genes/gene clusters found only in the Clade 1 *L. pneumophila* strains. (**b**) Unique genes/gene clusters found only in the clade 2 *Legionella* species. (**c**) Unique genes/gene clusters found only in the clade 3 *Legionella* species. (**d**) Unique genes/gene clusters found only in the Clade 4 *Legionella* species.

LPE509_01	LPE509_01153 hypothetical protein

1<br>3<br>9	
LPE509_01397	LPE509_01421 hypothetical protein
L<br>P<br>E<br>5<br>0<br>9<br>_<br>0<br>2<br>2<br>5<br>9	LPE509_02290 GIY-YIG nuclease superfamily protein
LPE509_00370	LPE509_00375 Collagen triple helix repeat (20 copies)
LPE509_01138	LPE509_01152 hypothetical protein
LPE509_02286	LPE509_02318 hypothetical protein
Lorraine00037	Lorraine_00037 hypothetical protein
LPE509_00541	LPE509_00547 hypothetical protein
LPE509_00564	LPE509_00570 Heme NO binding protein
LPE509_01157	LPE509_01171 hypothetical protein
LPE509_02111	LPE509_02141 hypothetical protein
LPE509_00100	LPE509_00101 Histamine oxidase
LPE509_00107	LPE509_00108 hypothetical protein
LPE509_00111	LPE509_00112 hypothetical protein
LPE509_00144	LPE509_00145 hypothetical protein
LPE509_00170	LPE509_00171 putative PEP-CTERM system TPR-repeat lipoprotein
LPE509_00173	LPE509_00174 hypothetical protein
LPE509_00192	LPE509_00193 hypothetical protein

LPE509_00320	LPE509_00325 Malonyl-CoA O-methyltransferase BioC
LPE509_00336	LPE509_00341 Endonuclease YhcR precursor
LPE509_00350	LPE509_00355 putative ATPase/kinase involved in NAD metabolism
LPE509_00430	LPE509_00435 hypothetical protein
LPE509_00474	LPE509_00480 hypothetical protein
LPE509_00477	LPE509_00483 hypothetical protein
LPE509_00480	LPE509_00486 hypothetical protein
LPE509_00491	LPE509_00497 Alpha amylase, catalytic domain
LPE509_00510	LPE509_00516 Dot/Icm substrate protein
LPE509_00511	LPE509_00517 hypothetical protein
LPE509_00533	LPE509_00539 hypothetical protein
LPE509_00581	LPE509_00587 hypothetical protein
LPE509_00590	LPE509_00596 hypothetical protein
LPE509_00593	LPE509_00599 Alkaline phosphatase precursor
LPE509_00617	LPE509_00623 Patatin-like phospholipase
LPE509_00618	LPE509_00624 hypothetical protein
LPE509_00631	LPE509_00637 putative peptidase
LPE509_00635	LPE509_00641 Ran GTPase-activating protein (RanGAP) involved in mRNA processing and transport
LPE509_00636	LPE509_00642 hypothetical protein
LPE509_00705	LPE509_00712 hypothetical protein
LPE509_00747	LPE509_00757 hypothetical protein
LPE509_00794	LPE509_00804 Putative multidrug export ATP-binding/permease protein
LPE509_00835	LPE509_00845 SnoaL-like domain protein
LPE509_00841	LPE509_00851 16 kDa heat shock protein A
LPE509_00847	LPE509_00857 Taurine catabolism dioxygenase TauD, TfdA family
LPE509_00881	LPE509_00892 hypothetical protein
LPE509_00883	LPE509_00894 hypothetical protein
LPE509_00893	LPE509_00904 hypothetical protein
LPE509_01080	LPE509_01094 Virulence-regulating protein VirS
LPE509_01126	LPE509_01140 Regulator of chromosome condensation (RCC1) repeat

LPE509_01172	LPE509_01186 hypothetical protein
LPE509_01190	LPE509_01205 Pilin
LPE509_01219	LPE509_01234 hypothetical protein
LPE509_01274	LPE509_01298 hypothetical protein
LPE509_01426	LPE509_01450 Alpha amylase, catalytic domain
LPE509_01428	LPE509_01452 malto-oligosyltrehalose trehalohydrolase
LPE509_01431	LPE509_01455 hypothetical protein
LPE509_01432	LPE509_01456 IgA Peptidase M64
LPE509_01437	LPE509_01461 multidrug resistance protein
LPE509_01439	LPE509_01463 Ran GTPase-activating protein (RanGAP) involved in mRNA processing and transport
LPE509_01443	LPE509_01467 glutathione synthetase
LPE509_01444	LPE509_01468 Virulence metalloprotease precursor
LPE509_01482	LPE509_01506 cytochrome c oxidase, cbb3-type, subunit III
LPE509_01509	LPE509_01535 hypothetical protein
LPE509_01611	LPE509_01637 hypothetical protein
LPE509_01619	LPE509_01645 hypothetical protein
LPE509_01809	LPE509_01836 hypothetical protein
LPE509_01825	LPE509_01852 hypothetical protein
LPE509_01884	LPE509_01913 Putative beta-lactamase HcpC precursor
LPE509_01886	LPE509_01915 Pyruvate formate-lyase 1-activating enzyme
LPE509_01887	LPE509_01916 hypothetical protein
LPE509_01894	LPE509_01923 hypothetical protein
LPE509_01897	LPE509_01926 carboxylate/amino acid/amine transporter
LPE509_01908	LPE509_01937 hypothetical protein
LPE509_01911	LPE509_01940 hypothetical protein
LPE509_01936	LPE509_01965 Membrane-bound lytic murein transglycosylase D precursor
LPE509_02107	LPE509_02137 Golgi nucleoside diphosphatase
LPE509_02110	LPE509_02140 hypothetical protein
LPE509_02181	LPE509_02211 hypothetical protein
LPE509_02295	LPE509_02327 N-acetylglucosaminyl-diphospho-decaprenol L-rhamnosyltransferase

LPE509_02296	LPE509_02328 N-acetylglucosaminyl-diphospho-decaprenol L-rhamnosyltransferase
LPE509_02297	LPE509_02329 hypothetical protein
LPE509_02409	LPE509_02441 Outer membrane protein OprM precursor
LPE509_02410	LPE509_02442 Inner membrane transport permease YbhR
LPE509_02411	LPE509_02443 Inner membrane transport permease YbhS
LPE509_02514	LPE509_02547 hypothetical protein
LPE509_02519	LPE509_02553 Gamma-glutamylputrescine oxidoreductase
LPE509_02567	LPE509_02603 hypothetical protein
LPE509_02624	LPE509_02660 hypothetical protein
LPE509_02691	LPE509_02727 hypothetical protein
LPE509_02702	LPE509_02738 hypothetical protein
LPE509_02788	LPE509_02830 5'-nucleotidase
LPE509_02796	LPE509_02838 hypothetical protein
LPE509_02797	LPE509_02839 hypothetical protein
LPE509_02801	LPE509_02843 hypothetical protein
LPE509_02844	LPE509_02886 hypothetical protein
LPE509_02858	LPE509_02900 hypothetical protein
LPE509_02899	LPE509_02941 hypothetical protein
LPE509_02993	LPE509_03037 putative transmembrane sensor domain protein
LPE509_02997	LPE509_03041 Peptidase C39 family protein
LPE509_03003	LPE509_03047 hypothetical protein
LPE509_03024	LPE509_03068 hypothetical protein
LPE509_03025	LPE509_03069 Cyclic di-GMP phosphodiesterase Gmr
LPE509_00475	LPE509_00481 hypothetical protein
LPE509_00867	LPE509_00878 hypothetical protein
LPE509_00880	LPE509_00891 hypothetical protein
LPE509_01130	LPE509_01144 hypothetical protein
LPE509_01605	LPE509_01631 hypothetical protein
LPE509_01900	LPE509_01929 Bacterial leucyl aminopeptidase precursor
LPE509_01960	LPE509_01989 Defects in Rab1 recruitment protein A

LPE509_02285	LPE509_02317 D-alanyl-lipoteichoic acid biosynthesis protein DltB
LPE509_02699	LPE509_02735 hypothetical protein
LPE509_02771	LPE509_02813 hypothetical protein
LPE509_02897	LPE509_02939 hypothetical protein
LPE509_03009	LPE509_03053 hypothetical protein
LPE509_00097	LPE509_00098 hypothetical protein
LPE509_00099	LPE509_00100 hypothetical protein
LPE509_00267	LPE509_00271 hypothetical protein
LPE509_00687	LPE509_00694 hypothetical protein
LPE509_00698	LPE509_00705 hypothetical protein
LPE509_01358	LPE509_01382 hypothetical protein
LPE509_01415	LPE509_01439 hypothetical protein
LPE509_02176	LPE509_02206 hypothetical protein
LPE509_02287	LPE509_02319 Transposase
LPE509_00472	LPE509_00478 hypothetical protein
LPE509_00656	LPE509_00662 hypothetical protein
LPE509_03004	LPE509_03048 hypothetical protein
LPE509_00657	LPE509_00663 hypothetical protein
LPE509_01650	LPE509_01676 2OG-Fe(II) oxygenase superfamily protein
LPE509_01963	LPE509_01992 hypothetical protein
LPE509_02552	LPE509_02588 hypothetical protein
LPE509_02565	LPE509_02601 hypothetical protein
LPE509_02871	LPE509_02913 hypothetical protein
LPE509_03016	LPE509_03060 hypothetical protein
LPE509_00620	LPE509_00626 hypothetical protein
LPE509_00882	LPE509_00893 hypothetical protein
LPE509_01651	LPE509_01677 hypothetical protein
LPE509_02307	LPE509_02339 hypothetical protein
LPE509_00089	LPE509_00090 putative nucleotidyltransferases
LPE509_00090	LPE509_00091 nucleotidyltransferase substrate binding protein, family

LPE509_00612	LPE509_00618 hypothetical protein
LPE509_00888	LPE509_00899 hypothetical protein
LPE509_01948	LPE509_01977 hypothetical protein
LPE509_02300	LPE509_02332 hypothetical protein
LPE509_03040	LPE509_03084 hypothetical protein
LPE509_00606	LPE509_00612 beta-lactamase/D-alanine carboxypeptidase
LPE509_00607	LPE509_00613 Opacity protein antigens
LPE509_00624	LPE509_00630 hypothetical protein
LPE509_01429	LPE509_01453 hypothetical protein
LPE509_01947	LPE509_01976 hypothetical protein
Lorraine01041	Lorraine_01056 hypothetical protein
Lorraine02111	Lorraine_02145 hypothetical protein
Lorraine02389	Lorraine_02427 hypothetical protein
Lorraine02961	Lorraine_03004 hypothetical protein
LPE509_00056	LPE509_00057 hypothetical protein
LPE509_00057	LPE509_00058 hypothetical protein
LPE509_00059	LPE509_00060 Asparagine synthetase [glutamine-hydrolyzing] 3
LPE509_00061	LPE509_00062 hypothetical protein
LPE509_00062	LPE509_00063 Heterocyst differentiation ATP-binding protein HepA
LPE509_00063	LPE509_00064 hypothetical protein
LPE509_00718	LPE509_00725 hypothetical protein
Lorraine00499	Lorraine_00507 hypothetical protein
LPE509_01880	LPE509_01909 hypothetical protein
LPE509_02303	LPE509_02335 Pseudaminic acid synthase
LPE509_02305	LPE509_02337 hypothetical protein
LPE509_02867	LPE509_02909 Ribose transport system permease protein RbsC
LPE509_02868	LPE509_02910 Ribose import ATP-binding protein RbsA
LPE509_02869	LPE509_02911 D-ribose-binding periplasmic protein precursor
Lorraine01391	Lorraine_01409 Pathogenicity locus
Alcoy00213	Alcoy_00215 hypothetical protein

p_43290_00773	pneu_43290_00785 Oxidoreductase family, NAD-binding Rossmann fold
p_43290_00774	pneu_43290_00786 Oxidoreductase family, NAD-binding Rossmann fold
LPE509_00580	LPE509_00586 hypothetical protein
LPE509_00614	LPE509_00620 hypothetical protein
LPE509_02981	LPE509_03025 putative DMT superfamily transporter inner membrane protein
Lorraine02004	Lorraine_02034 hypothetical protein
LPE509_00157	LPE509_00158 hypothetical protein
LPE509_00287	LPE509_00291 hypothetical protein
LPE509_00840	LPE509_00850 Spore protein SP21
LPE509_02302	LPE509_02334 Demethylmenaquinone methyltransferase
LPE509_02304	LPE509_02336 serine/threonine protein kinase
LPE509_02984	LPE509_03028 hypothetical protein
LPE509_02985	LPE509_03029 hypothetical protein
Alcoy02350	Alcoy_02387 hypothetical protein
LPE509_00058	LPE509_00059 hypothetical protein
LPE509_00070	LPE509_00071 putative neuraminidase (sialidase)
LPE509_00071	LPE509_00072 hypothetical protein
LPE509_01609	LPE509_01635 hypothetical protein
LPE509_01746	LPE509_01772 hypothetical protein
LPE509_01808	LPE509_01835 hypothetical protein
LPE509_02902	LPE509_02944 putative P-loop ATPase
Lorraine02370	Lorraine_02408 hypothetical protein
Alcoy02658	Alcoy_02696 Heat shock protein J
Corby02937	Corby_02978 hypothetical protein
p_43290_00753	pneu_43290_00765 hypothetical protein
sg10_00507	L_pne_10_00519 putative transmembrane sensor domain protein
LPE509_00661	LPE509_00667 hypothetical protein
LPE509_02306	LPE509_02338 hypothetical protein
LPE509_03010	LPE509_03054 Fatty acid hydroxylase superfamily protein
Lorraine00286	Lorraine_00288 hypothetical protein

Lorraine00288	Lorraine_00290 hypothetical protein
Lorraine01350	Lorraine_01368 hypothetical protein
Alcoy01186	Alcoy_01201 hypothetical protein
Alcoy02179	Alcoy_02212 hypothetical protein
p_43290_00752	pneu_43290_00764 D-alanyl-lipoteichoic acid biosynthesis protein DltB
p_43290_00785	pneu_43290_00797 CDP-Glycerol:Poly(glycerophosphate) glycerophosphotransferase
LPE509_00290	LPE509_00294 hypothetical protein
LPE509_00444	LPE509_00450 hypothetical protein
LPE509_00808	LPE509_00818 Regulator of chromosome condensation (RCC1) repeat
LPE509_01786	LPE509_01813 hypothetical protein
LPE509_02336	LPE509_02368 hypothetical protein
Lorraine02043	Lorraine_02076 hypothetical protein
Lorraine02044	Lorraine_02077 Cyn operon transcriptional activator
Lorraine02049	Lorraine_02082 Ankyrin repeats (3 copies)
Lorraine02095	Lorraine_02128 hypothetical protein
Lorraine02096	Lorraine_02129 hypothetical protein
Lorraine02445	Lorraine_02483 hypothetical protein
Alcoy00411	Alcoy_00419 choline dehydrogenase
Alcoy01257	Alcoy_01272 hypothetical protein
pHL00831	pneu_HL_00843 hypothetical protein
sg10_01290	L_pne_10_01307 N-acylneuraminate cytidylyltransferase
LPE509_00449	LPE509_00455 hypothetical protein
LPE509_00516	LPE509_00522 hypothetical protein
LPE509_00521	LPE509_00527 hypothetical protein
LPE509_00649	LPE509_00655 putative protein related to capsule biosynthesis enzymes
LPE509_00744	LPE509_00754 Quercetin 2,3-dioxygenase
LPE509_00786	LPE509_00796 hypothetical protein
LPE509_00986	LPE509_00997 hypothetical protein
LPE509_01169	LPE509_01183 hypothetical protein
LPE509_01489	LPE509_01513 hypothetical protein

LPE509_01979	LPE509_02008 hypothetical protein
Lorraine00452	Lorraine_00460 hypothetical protein
Lorraine01526	Lorraine_01546 hypothetical protein
Lorraine02388	Lorraine_02426 SNARE domain protein
Alcoy00788	Alcoy_00800 hypothetical protein
pHL03086	pneu_HL_03130 Fungalysin/Thermolysin Propeptide Motif
p_43290_00756	pneu_43290_00768 Glutamate-1-semialdehyde 2,1-aminomutase
sg10_02626	L_pne_10_02655 p48
sg11_01034	LP_11__01054 hypothetical protein
LPE509_00054	LPE509_00055 hypothetical protein
LPE509_00442	LPE509_00448 hypothetical protein
LPE509_00443	LPE509_00449 hypothetical protein
LPE509_00457	LPE509_00463 hypothetical protein
LPE509_00476	LPE509_00482 hypothetical protein
LPE509_00494	LPE509_00500 F-box-like protein
LPE509_00577	LPE509_00583 putative endonuclease containing a URI domain protein
LPE509_00608	LPE509_00614 hypothetical protein
LPE509_00648	LPE509_00654 putative DNA-binding transcriptional regulator
LPE509_00650	LPE509_00656 hypothetical protein
LPE509_00668	LPE509_00674 hypothetical protein
LPE509_00874	LPE509_00885 hypothetical protein
LPE509_00900	LPE509_00911 hypothetical protein
LPE509_00901	LPE509_00912 hypothetical protein
LPE509_01108	LPE509_01122 hypothetical protein
LPE509_01121	LPE509_01135 hypothetical protein
LPE509_01127	LPE509_01141 hypothetical protein
LPE509_01430	LPE509_01454 hypothetical protein
LPE509_01782	LPE509_01809 hypothetical protein
LPE509_01914	LPE509_01943 hypothetical protein
LPE509_01980	LPE509_02009 hypothetical protein

LPE509_01983	LPE509_02012 Deoxyguanosinetriphosphate triphosphohydrolase
LPE509_01988	LPE509_02017 hypothetical protein
LPE509_02352	LPE509_02384 hypothetical protein
LPE509_02901	LPE509_02943 hypothetical protein
LPE509_02903	LPE509_02945 hypothetical protein
LPE509_02904	LPE509_02946 hypothetical protein
LPE509_02905	LPE509_02947 hypothetical protein
LPE509_03048	LPE509_03092 hypothetical protein
Lorraine00287	Lorraine_00289 hypothetical protein
Lorraine02036	Lorraine_02069 hypothetical protein
Lorraine02387	Lorraine_02425 Opacity protein antigens
p_43290_00765	pneu_43290_00777 hypothetical protein
p_43290_00766	pneu_43290_00778 hypothetical protein
Paris02435	Paris_02473 hypothetical protein
LPE509_00102	LPE509_00103 hypothetical protein
LPE509_00576	LPE509_00582 Transposase
LPE509_02018	LPE509_02047 hypothetical protein
LPE509_02301	LPE509_02333 hypothetical protein
LPE509_02986	LPE509_03030 hypothetical protein
Lorraine00166	Lorraine_00168 hypothetical protein
Lorraine00809	Lorraine_00821 hypothetical protein
Lorraine01249	Lorraine_01267 putative endonuclease containing a URI domain protein
Lorraine02376	Lorraine_02414 hypothetical protein
Lorraine02377	Lorraine_02415 Rieske [2Fe-2S] domain protein
Lorraine02378	Lorraine_02416 hypothetical protein
Lorraine02478	Lorraine_02516 hypothetical protein
Lorraine02561	Lorraine_02599 hypothetical protein
Lorraine03007	Lorraine_03050 acetyl-CoA acetyltransferase
Lorraine03008	Lorraine_03051 hypothetical protein
Alcoy00642	Alcoy_00654 UDP-N-acetylglucosamine 1-carboxyvinyltransferase

Alcoy02005	Alcoy_02035 hypothetical protein
Alcoy02092	Alcoy_02122 hypothetical protein
Alcoy02994	Alcoy_03037 hypothetical protein
Corby02693	Corby_02731 hypothetical protein
p_43290_00021	pneu_43290_00021 hypothetical protein
p_43290_00759	pneu_43290_00771 Imidazole glycerol phosphate synthase subunit HisF
p_43290_00760	pneu_43290_00772 Imidazole glycerol phosphate synthase subunit HisH 1
p_43290_00761	pneu_43290_00773 N-acetyl sugar amidotransferase
p_43290_00762	pneu_43290_00774 General stress protein 69
Paris00157	Paris_00159 CRISPR-associated protein Cas9/Csx12, subtype II-B/NMENI
Paris00159	Paris_00161 CRISPR-associated endoribonuclease Cas2
Paris00160	Paris_00162 CRISPR-associated protein Cas4
sg10_01937	L_pne_10_01958 hypothetical protein
sg10_03039	L_pne_10_03074 hypothetical protein
sg10_03043	L_pne_10_03078 Oxidoreductase family, NAD-binding Rossmann fold
sg10_03045	L_pne_10_03080 N-acylneuraminate cytidylyltransferase
sg11_00071	LP_11__00081 hypothetical protein
sg11_02024	LP_11__02053 hypothetical protein
sg13_00212	LP_13__00213 Glutathionyl-hydroquinone reductase YqjG
sg13_00487	LP_13__00491 hypothetical protein
LPE509_00952	LPE509_00963 hypothetical protein
LPE509_02431	LPE509_02463 hypothetical protein
Lorraine00035	Lorraine_00035 GIY-YIG nuclease superfamily protein
Lorraine00167	Lorraine_00169 hypothetical protein
Lorraine00393	Lorraine_00401 hypothetical protein
Lorraine00793	Lorraine_00805 Acyltransferase family protein
Lorraine01045	Lorraine_01060 hypothetical protein
Lorraine01049	Lorraine_01064 GIY-YIG nuclease superfamily protein
Lorraine01056	Lorraine_01071 hypothetical protein
Lorraine02448	Lorraine_02486 Aspartate aminotransferase

Lorraine02666	Lorraine_02705 hypothetical protein
Alcoy01131	Alcoy_01146 hypothetical protein
Alcoy01691	Alcoy_01711 Fe-S oxidoreductase
Alcoy03052	Alcoy_03095 hypothetical protein
Alcoy03056	Alcoy_03099 hypothetical protein
pHL02278	pneu_HL_02312 hypothetical protein
pHL02293	pneu_HL_02327 hypothetical protein
p_43290_02963	pneu_43290_03007 hypothetical protein
sg10_01291	L_pne_10_01308 N-acetyl sugar amidotransferase
sg10_03084	L_pne_10_03119 hypothetical protein
sg11_01861	LP_11__01886 hypothetical protein
sg13_00298	LP_13__00299 putative N-formylglutamate amidohydrolase
sg13_00805	LP_13__00813 hypothetical protein
sg14_01411	LP_14__01429 hypothetical protein
LPE509_02094	LPE509_02123 hypothetical protein
LPE509_02098	LPE509_02127 hypothetical protein
Lorraine00068	Lorraine_00068 hypothetical protein
Lorraine00711	Lorraine_00723 Opacity protein antigens
Lorraine01389	Lorraine_01407 hypothetical protein
Lorraine01643	Lorraine_01663 hypothetical protein
Lorraine01645	Lorraine_01665 hypothetical protein
Lorraine01997	Lorraine_02027 hypothetical protein
Alcoy00873	Alcoy_00886 hypothetical protein
Alcoy01138	Alcoy_01153 Transposase, TnpA family
Alcoy01788	Alcoy_01808 hypothetical protein
Alcoy02017	Alcoy_02047 hypothetical protein
Alcoy02234	Alcoy_02268 hypothetical protein
Alcoy02468	Alcoy_02506 hypothetical protein
Alcoy02995	Alcoy_03038 putative transcriptional regulator
Corby02048	Corby_02078 hypothetical protein

p_43290_00769	pneu_43290_00781 hypothetical protein
Paris00156	Paris_00158 hypothetical protein
sg10_01358	L_pne_10_01375 Formimidoylglutamase
sg10_01917	L_pne_10_01938 hypothetical protein
sg11_00275	LP_11__00285 hypothetical protein
sg11_00533	LP_11__00546 Aspartate aminotransferase
sg11_00711	LP_11__00724 hypothetical protein
sg11_00818	LP_11__00832 hypothetical protein
sg11_02302	LP_11__02332 Ankyrin repeats (3 copies)
sg11_02466	LP_11__02498 hypothetical protein
sg11_02774	LP_11__02809 Fic/DOC family protein
sg11_02808	LP_11__02843 hypothetical protein
sg12_00839	L_pne_12_D4955_00854 hypothetical protein
sg13_01156	LP_13__01167 CAI-1 autoinducer sensor kinase/phosphatase CqsS
sg14_00576	LP_14__00577 DNA polymerase I
sg14_00907	LP_14__00918 hypothetical protein
sg14_02664	LP_14__02694 hypothetical protein
sg14_02692	LP_14__02722 Trans-aconitate methyltransferase
sg14_02693	LP_14__02723 L-threonine 3-dehydrogenase
sg14_02841	LP_14__02873 Cyanophycin synthetase
sg14_02941	LP_14__02974 Trifunctional NAD biosynthesis/regulator protein NadR
sg14_02942	LP_14__02975 L-glutamyl-[BtrI acyl-carrier protein] decarboxylase
sg14_02944	LP_14__02977 Putative N-acetyl-LL-diaminopimelate aminotransferase
sg14_02948	LP_14__02981 hypothetical protein
sg2_02986	LP_02_03028 hypothetical protein
LPE509_01025	LPE509_01036 Propionate kinase
LPE509_01038	LPE509_01049 Cold shock-like protein CspE
LPE509_02908	LPE509_02950 hypothetical protein
Lorraine00150	Lorraine_00152 hypothetical protein
Lorraine00280	Lorraine_00282 hypothetical protein

Lorraine00292	Lorraine_00294 hypothetical protein
Lorraine00540	Lorraine_00548 hypothetical protein
Lorraine00856	Lorraine_00869 hypothetical protein
Lorraine00987	Lorraine_01002 hypothetical protein
Lorraine00988	Lorraine_01003 ATP-dependent helicase HepA
Lorraine00989	Lorraine_01004 hypothetical protein
Lorraine00990	Lorraine_01005 putative outer membrane lipoprotein
Lorraine00991	Lorraine_01006 chromosome segregation protein
Lorraine01054	Lorraine_01069 hypothetical protein
Lorraine01644	Lorraine_01664 hypothetical protein
Lorraine01780	Lorraine_01800 hypothetical protein
Lorraine02053	Lorraine_02086 Dot/Icm substrate protein
Lorraine02372	Lorraine_02410 hypothetical protein
Lorraine02435	Lorraine_02473 hypothetical protein
Alcoy00151	Alcoy_00153 hypothetical protein
Alcoy00155	Alcoy_00157 Pertussis toxin liberation protein H
Alcoy00160	Alcoy_00162 conjugal transfer protein TrbG
Alcoy00162	Alcoy_00164 Type IV secretion system protein virB10
Alcoy00170	Alcoy_00172 50S ribosomal protein L22/unknown domain fusion protein
Alcoy00171	Alcoy_00173 hypothetical protein
Alcoy00177	Alcoy_00179 hypothetical protein
Alcoy00191	Alcoy_00193 hypothetical protein
Alcoy00204	Alcoy_00206 hypothetical protein
Alcoy00206	Alcoy_00208 Inosine-uridine preferring nucleoside hydrolase
Alcoy02003	Alcoy_02033 hypothetical protein
Alcoy02058	Alcoy_02088 hypothetical protein
Alcoy02495	Alcoy_02533 hypothetical protein
Alcoy02665	Alcoy_02703 hypothetical protein
Alcoy02993	Alcoy_03036 hypothetical protein
Alcoy02996	Alcoy_03039 hypothetical protein

Alcoy02997	Alcoy_03040 hypothetical protein
Corby01184	Corby_01199 hypothetical protein
pHL00207	pneu_HL_00209 hypothetical protein
pHL00212	pneu_HL_00214 Transposase
p_43290_00822	pneu_43290_00835 Transposase DDE domain protein
Philadelphia01227	Philadelphia_01243 Eco47II restriction endonuclease
Philadelphia01251	Philadelphia_01267 hypothetical protein
Philadelphia01259	Philadelphia_01275 hypothetical protein
Philadelphia01264	Philadelphia_01280 hypothetical protein
Philadelphia01265	Philadelphia_01281 hypothetical protein
sg10_02166	L_pne_10_02191 hypothetical protein
sg10_03050	L_pne_10_03085 hypothetical protein
sg11_00321	LP_11__00331 hypothetical protein
sg11_02405	LP_11__02437 hypothetical protein
sg11_02699	LP_11__02734 hypothetical protein
sg12_01888	L_pne_12_D4955_01915 hypothetical protein
sg12_01891	L_pne_12_D4955_01918 deoxyguanosinetriphosphate triphosphohydrolase-like protein
sg12_01892	L_pne_12_D4955_01919 hypothetical protein
sg13_00899	LP_13__00907 putative decaprenylphosphoryl-beta-D-ribose oxidase
sg13_02992	LP_13__03023 hypothetical protein
sg14_00242	LP_14__00242 sporadically distributed protein
sg14_01199	LP_14__01211 hypothetical protein
sg14_02053	LP_14__02075 hypothetical protein
sg14_02581	LP_14__02611 hypothetical protein
sg15_02995	LP_15__03031 hypothetical protein
sg17_02737	LP_17__02769 hypothetical protein
sg2_00705	LP_02_00716 hypothetical protein
sg2_00929	LP_02_00943 hypothetical protein
sg2_02843	LP_02_02879 hypothetical protein
sg2_02887	LP_02_02924 hypothetical protein

LPE509_02087	LPE509_02116 hypothetical protein
LPE509_02088	LPE509_02117 hypothetical protein
LPE509_02089	LPE509_02118 conjugative coupling factor TraD, PFGI-1 class
LPE509_02090	LPE509_02119 hypothetical protein
Lorraine00310	Lorraine_00312 hypothetical protein
Lorraine01028	Lorraine_01043 GIY-YIG nuclease superfamily protein
Lorraine01050	Lorraine_01065 hypothetical protein
Lorraine01245	Lorraine_01263 hypothetical protein
Lorraine02327	Lorraine_02365 hypothetical protein
Lorraine02328	Lorraine_02366 hypothetical protein
Lorraine02335	Lorraine_02373 hypothetical protein
Lorraine02338	Lorraine_02376 hypothetical protein
Lorraine02350	Lorraine_02388 putative hydrophobic domain protein
Lorraine02647	Lorraine_02685 hypothetical protein
Lorraine03026	Lorraine_03069 hypothetical protein
Alcoy00064	Alcoy_00064 hypothetical protein
Alcoy00065	Alcoy_00065 Bacteriophytochrome cph2
Alcoy00194	Alcoy_00196 hypothetical protein
Alcoy00196	Alcoy_00198 hypothetical protein
Alcoy00198	Alcoy_00200 hypothetical protein
Alcoy00200	Alcoy_00202 SWIM/SEC-C metal-binding motif protein, family
Alcoy00201	Alcoy_00203 hypothetical protein
Alcoy01174	Alcoy_01189 hypothetical protein
Alcoy01200	Alcoy_01215 hypothetical protein
Alcoy01220	Alcoy_01235 circadian clock protein KaiC
Alcoy01234	Alcoy_01249 hypothetical protein
Alcoy01235	Alcoy_01250 hypothetical protein
Alcoy01776	Alcoy_01796 hypothetical protein
Alcoy01789	Alcoy_01809 hypothetical protein
Alcoy01790	Alcoy_01810 Glucan endo-1,3-beta-glucosidase A1 precursor

Alcoy01803	Alcoy_01823 hypothetical protein
Alcoy02004	Alcoy_02034 hypothetical protein
Alcoy02053	Alcoy_02083 hypothetical protein
Alcoy02152	Alcoy_02185 hypothetical protein
Alcoy02210	Alcoy_02243 hypothetical protein
Alcoy02280	Alcoy_02314 hypothetical protein
Alcoy02597	Alcoy_02635 hypothetical protein
Alcoy02607	Alcoy_02645 hypothetical protein
Alcoy03027	Alcoy_03070 hypothetical protein
Alcoy03029	Alcoy_03072 hypothetical protein
Corby00330	Corby_00332 hypothetical protein
Corby00847	Corby_00859 hypothetical protein
pHL00193	pneu_HL_00195 hypothetical protein
pHL00208	pneu_HL_00210 hypothetical protein
pHL02191	pneu_HL_02224 phosphoglycerate transporter family protein
pHL02199	pneu_HL_02232 hypothetical protein
p_43290_01270	pneu_43290_01288 hypothetical protein
Paris00203	Paris_00205 hypothetical protein
sg10_00513	L_pne_10_00525 Anthranilate synthase component II
sg10_00854	L_pne_10_00867 hypothetical protein
sg10_01377	L_pne_10_01394 DNA topoisomerase 4 subunit B
sg10_02007	L_pne_10_02028 hypothetical protein
sg10_02184	L_pne_10_02209 Pseudooxynicotine oxidase
sg10_02687	L_pne_10_02717 hypothetical protein
sg10_03163	L_pne_10_03204 hypothetical protein
sg11_00036	LP_11__00037 hypothetical protein
sg11_00099	LP_11__00109 enoyl-CoA hydratase
sg11_00578	LP_11__00591 Cytochrome d ubiquinol oxidase subunit 1
sg11_01596	LP_11__01619 hypothetical protein
sg11_01631	LP_11__01655 Cytochrome c-type biogenesis protein CcmF

sg11_01854	LP_11__01879 hypothetical protein
sg11_02417	LP_11__02449 hypothetical protein
sg11_02985	LP_11__03020 hypothetical protein
sg12_00133	L_pne_12_D4955_00134 putative isomerase YddE
sg13_02319	LP_13__02348 hypothetical protein
sg13_02952	LP_13__02983 hypothetical protein
sg13_02972	LP_13__03003 nicotinate phosphoribosyltransferase
sg14_00900	LP_14__00911 hypothetical protein
sg14_02058	LP_14__02080 hypothetical protein
sg14_02491	LP_14__02520 hypothetical protein
sg14_02535	LP_14__02564 hypothetical protein
sg15_00669	LP_15__00675 Acyl-CoA dehydrogenase
sg15_01078	LP_15__01094 Phosphocholine transferase AnkX
sg15_01174	LP_15__01190 hypothetical protein
sg15_01834	LP_15__01856 Inner membrane protein YrbG
sg15_01900	LP_15__01922 hypothetical protein
sg15_02119	LP_15__02143 hypothetical protein
sg15_02120	LP_15__02144 KAP family P-loop domain protein
sg15_02292	LP_15__02318 hypothetical protein
sg15_02977	LP_15__03013 deoxyguanosinetriphosphate triphosphohydrolase-like protein
sg16_00193	LP_16_00194 hypothetical protein
sg16_01315	LP_16_01327 hypothetical protein
sg16_01637	LP_16_01651 hypothetical protein
sg16_02610	LP_16_02640 hypothetical protein
sg17_01327	LP_17__01347 hypothetical protein
sg17_02280	LP_17__02310 Phosphocholine transferase AnkX
sg17_03235	LP_17__03271 Transposase
sg2_02367	LP_02_02397 Xaa-Pro aminopeptidase
sg2_02710	LP_02_02745 Transposase IS66 family protein
sg2_02711	LP_02_02746 Transposase

sg3_00018	sg3_00020 hypothetical protein
LPE509_00118	LPE509_00119 hypothetical protein
LPE509_01608	LPE509_01634 hypothetical protein
LPE509_02047	LPE509_02076 hypothetical protein
LPE509_02051	LPE509_02080 short chain dehydrogenase
LPE509_02062	LPE509_02091 hypothetical protein
Lorraine00056	Lorraine_00056 GIY-YIG nuclease superfamily protein
Lorraine00147	Lorraine_00149 hypothetical protein
Lorraine00164	Lorraine_00166 hypothetical protein
Lorraine00416	Lorraine_00424 GIY-YIG nuclease superfamily protein
Lorraine00465	Lorraine_00473 hypothetical protein
Lorraine01220	Lorraine_01237 hypothetical protein
Lorraine01599	Lorraine_01619 GIY-YIG nuclease superfamily protein
Lorraine01703	Lorraine_01723 hypothetical protein
Lorraine01881	Lorraine_01911 hypothetical protein
Lorraine01883	Lorraine_01913 hypothetical protein
Lorraine02332	Lorraine_02370 hypothetical protein
Lorraine02333	Lorraine_02371 hypothetical protein
Lorraine02353	Lorraine_02391 hypothetical protein
Lorraine02414	Lorraine_02452 hypothetical protein
Lorraine02846	Lorraine_02887 putative endonuclease containing a URI domain protein
Alcoy00309	Alcoy_00311 hypothetical protein
Alcoy00841	Alcoy_00853 Tellurite methyltransferase
Alcoy00858	Alcoy_00870 hypothetical protein
Alcoy00859	Alcoy_00871 hypothetical protein
Alcoy01172	Alcoy_01187 hypothetical protein
Alcoy01787	Alcoy_01807 four helix bundle protein
Alcoy01791	Alcoy_01811 hypothetical protein
Alcoy01793	Alcoy_01813 four helix bundle protein
Alcoy02002	Alcoy_02032 hypothetical protein

Alcoy02150	Alcoy_02183 hypothetical protein
Alcoy02174	Alcoy_02207 hypothetical protein
Alcoy02422	Alcoy_02460 hypothetical protein
Alcoy02664	Alcoy_02702 hypothetical protein
Alcoy02666	Alcoy_02704 Helix-turn-helix domain protein
Alcoy02667	Alcoy_02705 hypothetical protein
Alcoy02670	Alcoy_02708 hypothetical protein
Alcoy02835	Alcoy_02875 CAI-1 autoinducer sensor kinase/phosphatase CqsS
Alcoy03098	Alcoy_03142 Pseudolysin precursor
Corby00205	Corby_00207 hypothetical protein
Corby01177	Corby_01192 Superfamily II helicase
Corby01178	Corby_01193 Superfamily II helicase
Corby01587	Corby_01605 CAAX amino terminal protease self- immunity
Corby02457	Corby_02495 putative cadmium-transporting ATPase
Corby02469	Corby_02507 Retron-type reverse transcriptase
Corby02506	Corby_02544 hypothetical protein
Corby02511	Corby_02549 hypothetical protein
pHL00189	pneu_HL_00191 hypothetical protein
pHL00575	pneu_HL_00583 hypothetical protein
pHL01503	pneu_HL_01521 hypothetical protein
pHL02296	pneu_HL_02330 hypothetical protein
pHL02850	pneu_HL_02891 hypothetical protein
pHL03042	pneu_HL_03085 Dolichyl-phosphate-mannose-protein mannosyltransferase
pHL03043	pneu_HL_03086 Lipopolysaccharide kinase (Kdo/WaaP) family protein
pHL03044	pneu_HL_03087 Beta-xylosidase
pHL03056	pneu_HL_03099 methionine biosynthesis protein MetW
p_43290_01014	pneu_43290_01029 hypothetical protein
Paris00039	Paris_00039 Transposase
Paris00161	Paris_00163 hypothetical protein
Paris00180	Paris_00182 hypothetical protein

Paris00209	Paris_00211 Transposase
Paris00826	Paris_00838 hypothetical protein
Paris00856	Paris_00868 Transposase
Paris01887	Paris_01916 hypothetical protein
Paris01967	Paris_01997 hypothetical protein
Paris02136	Paris_02169 hypothetical protein
Paris02260	Paris_02297 hypothetical protein
Paris02433	Paris_02471 hypothetical protein
Paris02467	Paris_02505 acetoacetyl-CoA reductase
Paris02631	Paris_02669 hypothetical protein
sg10_00133	L_pne_10_00134 hypothetical protein
sg10_00164	L_pne_10_00166 Peptide methionine sulfoxide reductase MsrA 2
sg10_00310	L_pne_10_00313 hypothetical protein
sg10_00660	L_pne_10_00673 PemK-like protein
sg10_00756	L_pne_10_00769 Cyclic di-GMP phosphodiesterase Gmr
sg10_01484	L_pne_10_01501 hypothetical protein
sg10_01508	L_pne_10_01525 transcriptional regulator, y4mF family
sg10_01512	L_pne_10_01529 conjugal transfer protein TraL
sg10_01514	L_pne_10_01531 hypothetical protein
sg10_01516	L_pne_10_01533 hypothetical protein
sg10_01521	L_pne_10_01538 hypothetical protein
sg10_01527	L_pne_10_01544 3-oxoacyl-[acyl-carrier-protein] reductase FabG
sg10_01528	L_pne_10_01545 Acetyl-CoA acetyltransferase
sg10_02049	L_pne_10_02073 hypothetical protein
sg10_02735	L_pne_10_02765 putative DNA helicase
sg10_02832	L_pne_10_02864 hypothetical protein
sg11_00332	LP_11__00342 hypothetical protein
sg11_00357	LP_11__00367 hypothetical protein
sg11_00619	LP_11__00632 hypothetical protein
sg11_00620	LP_11__00633 hypothetical protein

sg11_01016	LP_11__01035 hypothetical protein
sg11_01075	LP_11__01095 ribose-5-phosphate isomerase A
sg11_01255	LP_11__01276 hypothetical protein
sg11_01361	LP_11__01383 hypothetical protein
sg11_01446	LP_11__01468 hypothetical protein
sg11_01725	LP_11__01750 hypothetical protein
sg11_01858	LP_11__01883 hypothetical protein
sg11_01896	LP_11__01921 hypothetical protein
sg11_02019	LP_11__02048 ribosomal-protein-L7/L12-serine acetyltransferase
sg11_02167	LP_11__02197 hypothetical protein
sg11_02229	LP_11__02259 hypothetical protein
sg11_02276	LP_11__02306 Malonyl-CoA O-methyltransferase BioC
sg11_02282	LP_11__02312 putative dienelactone hydrolase
sg11_02607	LP_11__02642 deoxyguanosinetriphosphate triphosphohydrolase-like protein
sg11_02872	LP_11__02907 (R)-stereoselective amidase
sg11_02876	LP_11__02911 Pseudaminic acid synthase
sg11_02971	LP_11__03006 hypothetical protein
sg11_02992	LP_11__03027 Bacteriophytochrome cph2
sg11_03053	LP_11__03089 CRISPR-associated protein Cas9/Csx12, subtype II-B/NMENI
sg11_03069	LP_11__03105 hypothetical protein
sg11_03074	LP_11__03110 hypothetical protein
sg11_03077	LP_11__03113 hypothetical protein
sg11_03081	LP_11__03117 Reverse transcriptase (RNA-dependent DNA polymerase)
sg11_03082	LP_11__03118 hypothetical protein
sg11_03083	LP_11__03119 hypothetical protein
sg11_03118	LP_11__03155 hypothetical protein
sg11_03132	LP_11__03169 hypothetical protein
sg11_03133	LP_11__03170 Spore coat polysaccharide biosynthesis protein SpsE
sg11_03144	LP_11__03181 hypothetical protein
sg11_03178	LP_11__03215 hypothetical protein

sg12_02816	L_pne_12_D4955_02852 hypothetical protein
sg13_00217	LP_13__00218 hypothetical protein
sg13_00687	LP_13__00694 hypothetical protein
sg13_01033	LP_13__01044 preprotein translocase subunit SecA
sg13_01071	LP_13__01082 hypothetical protein
sg13_01445	LP_13__01466 periplasmic multidrug efflux lipoprotein precursor
sg13_01542	LP_13__01565 hypothetical protein
sg13_01617	LP_13__01640 hypothetical protein
sg13_01827	LP_13__01850 hypothetical protein
sg13_02017	LP_13__02044 hypothetical protein
sg13_02023	LP_13__02050 hypothetical protein
sg13_02024	LP_13__02051 TIR domain protein
sg13_02368	LP_13__02397 hypothetical protein
sg13_02537	LP_13__02567 hypothetical protein
sg13_02541	LP_13__02571 hypothetical protein
sg13_02627	LP_13__02657 hypothetical protein
sg13_02653	LP_13__02683 hypothetical protein
sg13_02654	LP_13__02684 carbon storage regulator
sg13_02721	LP_13__02751 hypothetical protein
sg13_02909	LP_13__02939 CRISPR-associated protein Csy3
sg13_02953	LP_13__02984 hypothetical protein
sg13_02955	LP_13__02986 hypothetical protein
sg13_02956	LP_13__02987 hypothetical protein
sg13_02957	LP_13__02988 hypothetical protein
sg13_03050	LP_13__03081 hypothetical protein
sg13_03062	LP_13__03093 hypothetical protein
sg14_00159	LP_14__00159 RNA polymerase sigma-54 factor 2
sg14_00282	LP_14__00282 hypothetical protein
sg14_00319	LP_14__00319 C4-dicarboxylate transport protein
sg14_00397	LP_14__00397 hypothetical protein

sg14_00566	LP_14__00567 hypothetical protein
sg14_00685	LP_14__00687 aspartate aminotransferase
sg14_00711	LP_14__00713 hypothetical protein
sg14_00942	LP_14__00954 hypothetical protein
sg14_00991	LP_14__01003 Penicillin-binding protein 4 precursor
sg14_00996	LP_14__01008 hypothetical protein
sg14_01047	LP_14__01059 hypothetical protein
sg14_01132	LP_14__01144 hypothetical protein
sg14_01183	LP_14__01195 hypothetical protein
sg14_01263	LP_14__01275 tyramine oxidase
sg14_01302	LP_14__01314 hypothetical protein
sg14_01637	LP_14__01655 Spore maturation protein B
sg14_01775	LP_14__01796 thymidylate kinase
sg14_01837	LP_14__01858 NAD-dependent malic enzyme
sg14_01913	LP_14__01935 Dot/Icm substrate protein
sg14_01999	LP_14__02021 hypothetical protein
sg14_02267	LP_14__02294 hypothetical protein
sg14_02287	LP_14__02314 hypothetical protein
sg14_02328	LP_14__02355 50S ribosomal protein L3
sg14_02427	LP_14__02455 hypothetical protein
sg14_02586	LP_14__02616 hypothetical protein
sg14_02588	LP_14__02618 hypothetical protein
sg14_02590	LP_14__02620 Superfamily II helicase
sg14_02691	LP_14__02721 Putative asparagine synthetase [glutamine-hydrolyzing]
sg14_02770	LP_14__02801 hypothetical protein
sg14_02792	LP_14__02823 hypothetical protein
sg14_02795	LP_14__02826 hypothetical protein
sg14_02811	LP_14__02842 hypothetical protein
sg14_02812	LP_14__02843 hypothetical protein
sg14_02813	LP_14__02844 hypothetical protein

sg14_02814	LP_14__02845 hypothetical protein
sg14_02820	LP_14__02851 hypothetical protein
sg14_02943	LP_14__02976 hypothetical protein
sg14_02986	LP_14__03020 Golgi nucleoside diphosphatase
sg14_03063	LP_14__03099 hypothetical protein
sg14_03090	LP_14__03126 Chaperone protein ClpB
sg14_03091	LP_14__03127 Major Facilitator Superfamily protein
sg14_03144	LP_14__03180 hypothetical protein
sg14_03166	LP_14__03203 dTDP-3-amino-3,4,6-trideoxy-alpha-D-glucopyranose
sg15_00039	LP_15__00041 hypothetical protein
sg15_00184	LP_15__00186 Beta-lactamase
sg15_00292	LP_15__00295 Patatin-like phospholipase
sg15_00457	LP_15__00462 malonate decarboxylase, alpha subunit
sg15_00727	LP_15__00734 hypothetical protein
sg15_01006	LP_15__01022 hypothetical protein
sg15_01121	LP_15__01137 hypothetical protein
sg15_01212	LP_15__01231 hypothetical protein
sg15_01359	LP_15__01378 Deoxyribodipyrimidine photo-lyase
sg15_01452	LP_15__01471 hypothetical protein
sg15_01750	LP_15__01772 Methionine import ATP-binding protein MetN
sg15_01875	LP_15__01897 cell division protein DamX
sg15_02038	LP_15__02061 hypothetical protein
sg15_02074	LP_15__02098 hypothetical protein
sg15_02867	LP_15__02902 Fatty acid desaturase
sg15_02868	LP_15__02903 hypothetical protein
sg15_03057	LP_15__03094 hypothetical protein
sg16_00235	LP_16_00236 tRNA 5-methylaminomethyl-2-thiouridine biosynthesis bifunctional protein MnmC
sg16_00362	LP_16_00363 hypothetical protein
sg16_01387	LP_16_01399 Macrophage killing protein with similarity to conjugation protein
sg16_01554	LP_16_01567 hypothetical protein

sg16_01609	LP_16_01622 amidase
sg16_02108	LP_16_02133 cytochrome C oxidase assembly protein
sg16_02562	LP_16_02589 lipoprotein releasing system, ATP-binding protein
sg16_02744	LP_16_02776 hypothetical protein
sg16_02774	LP_16_02808 hypothetical protein
sg16_03012	LP_16_03048 hypothetical protein
sg17_00097	LP_17__00098 hypothetical protein
sg17_00553	LP_17__00560 hypothetical protein
sg17_00747	LP_17__00757 hypothetical protein
sg17_00748	LP_17__00758 hypothetical protein
sg17_00758	LP_17__00768 Sulfonamide resistance protein
sg17_00759	LP_17__00769 Multidrug resistance protein D
sg17_00775	LP_17__00785 hypothetical protein
sg17_00842	LP_17__00861 hypothetical protein
sg17_01568	LP_17__01592 hypothetical protein
sg17_01603	LP_17__01628 Dot/Icm substrate protein
sg17_01615	LP_17__01640 hypothetical protein
sg17_01761	LP_17__01787 hypothetical protein
sg17_02054	LP_17__02084 hypothetical protein
sg17_02435	LP_17__02465 hypothetical protein
sg17_02659	LP_17__02691 hypothetical protein
sg17_02799	LP_17__02833 hypothetical protein
sg17_02815	LP_17__02850 hypothetical protein
sg17_02854	LP_17__02889 hypothetical protein
sg17_02916	LP_17__02951 hypothetical protein
sg17_03052	LP_17__03087 hypothetical protein
sg17_03214	LP_17__03250 Legionella pneumophila major outer membrane protein precursor
sg17_03215	LP_17__03251 carboxylate/amino acid/amine transporter
sg2_00002	LP_02_00002 hypothetical protein
sg2_00337	LP_02_00343 Superfamily II helicase

sg2_00338	LP_02_00344 Superfamily II helicase
sg2_00340	LP_02_00346 hypothetical protein
sg2_00343	LP_02_00349 hypothetical protein
sg2_00350	LP_02_00356 hypothetical protein
sg2_00351	LP_02_00357 hypothetical protein
sg2_00352	LP_02_00358 hypothetical protein
sg2_00621	LP_02_00632 hypothetical protein
sg2_01067	LP_02_01083 Isoleucine--tRNA ligase
sg2_02283	LP_02_02313 hypothetical protein
sg2_02320	LP_02_02350 biotin biosynthesis protein BioC
sg2_02324	LP_02_02354 hypothetical protein
sg2_02424	LP_02_02454 ComEC family competence protein
sg2_02528	LP_02_02560 C-20 methyltransferase BchU
sg2_02953	LP_02_02990 Response regulator UvrY
sg3_00599	sg3_00613 hypothetical protein
sg3_00710	sg3_00725 hypothetical protein
sg3_00772	sg3_00787 Dicarboxylic acid uptake system A
sg3_01311	sg3_01335 hypothetical protein
sg3_02756	sg3_02790 Membrane-bound lytic murein transglycosylase D precursor
sg4_03038	LP_04_03072 hypothetical protein
sg5_01241	LP_05_01254 hypothetical protein
sg5_01800	LP_05_01828 hypothetical protein
sg5_01830	LP_05_01859 putative phage-associated protein
sg5_03031	LP_05_03068 hypothetical protein
sg5_03168	LP_05_03206 hypothetical protein
sg6_00292	LP_06_00301 Gamma-glutamyl phosphate reductase
sg7_00846	sg7_00864 hypothetical protein
sg7_00847	sg7_00865 Spore coat polysaccharide biosynthesis protein SpsE
sg8_00183	sg8_00183 hypothetical protein
Alcoy01051	Alcoy_01066 hypothetical protein

Alcoy01055	Alcoy_01070 hypothetical protein
Alcoy01072	Alcoy_01087 integrating conjugative element protein, family
Alcoy01184	Alcoy_01199 hypothetical protein
Alcoy01185	Alcoy_01200 hypothetical protein
Alcoy02245	Alcoy_02279 hypothetical protein
Alcoy02421	Alcoy_02459 Transposase
Alcoy02426	Alcoy_02464 hypothetical protein
Alcoy02430	Alcoy_02468 hypothetical protein
Alcoy02433	Alcoy_02471 hypothetical protein
Alcoy02513	Alcoy_02551 Small integral membrane protein
Alcoy02923	Alcoy_02966 hypothetical protein
Alcoy03024	Alcoy_03067 hypothetical protein
Lorraine00036	Lorraine_00036 hypothetical protein
Lorraine00038	Lorraine_00038 hypothetical protein
Lorraine00057	Lorraine_00057 hypothetical protein
Lorraine00135	Lorraine_00136 hypothetical protein
Lorraine00244	Lorraine_00246 putative endonuclease containing a URI domain protein
Lorraine00245	Lorraine_00247 putative endonuclease containing a URI domain protein
Lorraine00395	Lorraine_00403 hypothetical protein
Lorraine00453	Lorraine_00461 hypothetical protein
Lorraine00787	Lorraine_00799 hypothetical protein
Lorraine00995	Lorraine_01010 hypothetical protein
Lorraine01120	Lorraine_01136 hypothetical protein
Lorraine01172	Lorraine_01189 hypothetical protein
Lorraine01174	Lorraine_01191 GIY-YIG nuclease superfamily protein
Lorraine01219	Lorraine_01236 GIY-YIG nuclease superfamily protein
Lorraine01247	Lorraine_01265 hypothetical protein
Lorraine01248	Lorraine_01266 putative endonuclease containing a URI domain protein
Lorraine01285	Lorraine_01303 GIY-YIG nuclease superfamily protein
Lorraine01394	Lorraine_01412 hypothetical protein

Lorraine01397	Lorraine_01415 Transposase IS116/IS110/IS902 family protein
Lorraine01399	Lorraine_01417 Transposase
Lorraine01533	Lorraine_01553 GIY-YIG nuclease superfamily protein
Lorraine01546	Lorraine_01566 hypothetical protein
Lorraine01547	Lorraine_01567 GIY-YIG nuclease superfamily protein
Lorraine01597	Lorraine_01617 hypothetical protein
Lorraine01598	Lorraine_01618 hypothetical protein
Lorraine01860	Lorraine_01889 hypothetical protein
Lorraine01922	Lorraine_01952 hypothetical protein
Lorraine02359	Lorraine_02397 hypothetical protein
Lorraine02537	Lorraine_02575 hypothetical protein
Lorraine02680	Lorraine_02719 hypothetical protein
Lorraine02681	Lorraine_02720 putative endonuclease containing a URI domain protein
Lorraine02845	Lorraine_02886 hypothetical protein
Lorraine02936	Lorraine_02979 hypothetical protein
Lorraine03075	Lorraine_03118 GIY-YIG nuclease superfamily protein
LPE509_02428	LPE509_02460 hypothetical protein
Paris00081	Paris_00081 hypothetical protein
Paris00082	Paris_00082 Protein kinase domain protein
Paris00083	Paris_00083 hypothetical protein
Paris00198	Paris_00200 hypothetical protein
Paris00204	Paris_00206 Site-specific recombinase XerD
Paris00208	Paris_00210 hypothetical protein
Paris00212	Paris_00214 hypothetical protein
Paris00265	Paris_00267 hypothetical protein
Paris00288	Paris_00290 hypothetical protein
Paris00291	Paris_00293 hypothetical protein
Paris00292	Paris_00294 Ubiquinol oxidase subunit 2 precursor
Paris00293	Paris_00295 Ubiquinol oxidase subunit 1
Paris00294	Paris_00296 Cytochrome o ubiquinol oxidase subunit 3

Paris00295	Paris_00297 Cytochrome o ubiquinol oxidase protein CyoD
Paris00296	Paris_00298 hypothetical protein
Paris00297	Paris_00299 hypothetical protein
Paris00299	Paris_00301 putative periplasmic ligand-binding sensor domain protein
Paris00331	Paris_00333 GIY-YIG nuclease superfamily protein
Paris00771	Paris_00783 Transposase
Paris00775	Paris_00787 hypothetical protein
Paris00839	Paris_00851 N-acetylglucosaminyl-diphospho-decaprenol L-rhamnosyltransferase
Paris01566	Paris_01586 hypothetical protein
Paris01824	Paris_01844 hypothetical protein
Paris01876	Paris_01905 hypothetical protein
Paris01877	Paris_01906 hypothetical protein
Paris01951	Paris_01981 hypothetical protein
Paris02028	Paris_02058 hypothetical protein
Paris02053	Paris_02086 hypothetical protein
Paris02099	Paris_02132 hypothetical protein
Paris02134	Paris_02167 hypothetical protein
Paris02142	Paris_02175 hypothetical protein
Paris02446	Paris_02484 hypothetical protein
Paris02512	Paris_02550 hypothetical protein
Paris02882	Paris_02925 4-hydroxy-tetrahydrodipicolinate synthase
Paris02883	Paris_02926 gentisate 1,2-dioxygenase
Paris02884	Paris_02927 Cyn operon transcriptional activator
Paris02885	Paris_02928 hypothetical protein
Paris03010	Paris_03053 hypothetical protein
Philadelphia00753	Philadelphia_00765 N-acetylglucosaminyl-diphospho-decaprenol L-rhamnosyltransferase
Philadelphia01508	Philadelphia_01526 Heterocyst differentiation ATP-binding protein HepA
Philadelphia02732	Philadelphia_02772 hypothetical protein
pHL00168	pneu_HL_00170 hypothetical protein
pHL00185	pneu_HL_00187 hypothetical protein

pHL00186	pneu_HL_00188 hypothetical protein
pHL00323	pneu_HL_00325 GIY-YIG nuclease superfamily protein
pHL00689	pneu_HL_00701 hypothetical protein
pHL00691	pneu_HL_00703 hypothetical protein
pHL00692	pneu_HL_00704 hypothetical protein
pHL00820	pneu_HL_00832 pyrroloquinoline quinone biosynthesis protein PqqE
pHL00821	pneu_HL_00833 hypothetical protein
pHL01109	pneu_HL_01124 Poly-beta-hydroxybutyrate polymerase
pHL01110	pneu_HL_01125 Poly-beta-hydroxybutyrate polymerase
pHL01121	pneu_HL_01136 hypothetical protein
pHL01122	pneu_HL_01137 Antitoxin HicB
pHL01123	pneu_HL_01138 YcfA-like protein
pHL01464	pneu_HL_01482 hypothetical protein
pHL01619	pneu_HL_01639 Bacteriophytochrome cph2
pHL01655	pneu_HL_01675 hypothetical protein
pHL01666	pneu_HL_01686 hypothetical protein
pHL01667	pneu_HL_01687 hypothetical protein
pHL01713	pneu_HL_01733 hypothetical protein
pHL01782	pneu_HL_01802 hypothetical protein
pHL01810	pneu_HL_01830 Undecaprenyl phosphate-alpha-4-amino-4-deoxy-L-arabinose arabinosyl transferase
pHL02043	pneu_HL_02073 hypothetical protein
pHL02054	pneu_HL_02084 hypothetical protein
pHL02084	pneu_HL_02114 hypothetical protein
pHL02103	pneu_HL_02133 intracellular protease, PfpI family
pHL02229	pneu_HL_02262 hypothetical protein
pHL02319	pneu_HL_02353 hypothetical protein
pHL02438	pneu_HL_02476 hypothetical protein
pHL02441	pneu_HL_02479 hypothetical protein
pHL02445	pneu_HL_02483 hypothetical protein
pHL02456	pneu_HL_02494 hypothetical protein

pHL02534	pneu_HL_02572 hypothetical protein
pHL02647	pneu_HL_02685 hypothetical protein
pHL02761	pneu_HL_02800 2-oxoglutarate carboxylase small subunit
pHL03012	pneu_HL_03055 hypothetical protein
pHL03089	pneu_HL_03133 hypothetical protein
sg10_00038	L_pne_10_00039 hypothetical protein
sg10_00064	L_pne_10_00065 hypothetical protein
sg10_00101	L_pne_10_00102 hypothetical protein
sg10_00105	L_pne_10_00106 hypothetical protein
sg10_00106	L_pne_10_00107 hypothetical protein
sg10_00120	L_pne_10_00121 hypothetical protein
sg10_00132	L_pne_10_00133 hypothetical protein
sg10_00272	L_pne_10_00275 hypothetical protein
sg10_00307	L_pne_10_00310 hypothetical protein
sg10_00589	L_pne_10_00601 hypothetical protein
sg10_00659	L_pne_10_00672 hypothetical protein
sg10_00662	L_pne_10_00675 hypothetical protein
sg10_00934	L_pne_10_00947 hypothetical protein
sg10_01030	L_pne_10_01045 Kynurenine 3-monooxygenase
sg10_01172	L_pne_10_01189 hypothetical protein
sg10_01294	L_pne_10_01311 hypothetical protein
sg10_01488	L_pne_10_01505 hypothetical protein
sg10_01554	L_pne_10_01571 conjugal transfer protein TrbA
sg10_01555	L_pne_10_01572 hypothetical protein
sg10_01751	L_pne_10_01770 hypothetical protein
sg10_01945	L_pne_10_01966 hypothetical protein
sg10_02026	L_pne_10_02047 Chorismate synthase
sg10_02062	L_pne_10_02086 Legionella pneumophila major outer membrane protein precursor
sg10_02063	L_pne_10_02087 Legionella pneumophila major outer membrane protein precursor
sg10_02373	L_pne_10_02401 hypothetical protein

sg10_02703	L_pne_10_02733 hypothetical protein
sg10_02726	L_pne_10_02756 hypothetical protein
sg10_02877	L_pne_10_02911 Integrase core domain protein
sg10_02964	L_pne_10_02998 hypothetical protein
sg10_02999	L_pne_10_03034 hypothetical protein
sg10_03042	L_pne_10_03077 hypothetical protein
sg10_03141	L_pne_10_03182 hypothetical protein
sg11_00003	LP_11__00004 hypothetical protein
sg11_00108	LP_11__00118 hypothetical protein
sg11_00175	LP_11__00185 hypothetical protein
sg11_00318	LP_11__00328 hypothetical protein
sg11_00436	LP_11__00446 arginine repressor
sg11_00525	LP_11__00538 Ankyrin repeats (3 copies)
sg11_00846	LP_11__00861 hypothetical protein
sg11_00847	LP_11__00862 hypothetical protein
sg11_00852	LP_11__00867 Na(+)/dicarboxylate symporter
sg11_00904	LP_11__00919 hypothetical protein
sg11_00905	LP_11__00920 hypothetical protein
sg11_01099	LP_11__01120 hypothetical protein
sg11_01151	LP_11__01172 hypothetical protein
sg11_01325	LP_11__01347 hypothetical protein
sg11_01439	LP_11__01461 3-oxoacyl-[acyl-carrier-protein] synthase 3
sg11_01523	LP_11__01546 hypothetical protein
sg11_01536	LP_11__01559 hypothetical protein
sg11_01671	LP_11__01695 hypothetical protein
sg11_01737	LP_11__01762 hypothetical protein
sg11_01745	LP_11__01770 hypothetical protein
sg11_01754	LP_11__01779 hypothetical protein
sg11_01781	LP_11__01806 hypothetical protein
sg11_01895	LP_11__01920 hypothetical protein

sg11_01947	LP_11__01973 hypothetical protein
sg11_02001	LP_11__02030 hypothetical protein
sg11_02061	LP_11__02090 hypothetical protein
sg11_02098	LP_11__02127 hypothetical protein
sg11_02118	LP_11__02148 hypothetical protein
sg11_02168	LP_11__02198 hypothetical protein
sg11_02224	LP_11__02254 hypothetical protein
sg11_02242	LP_11__02272 conjugative transfer ATPase, family
sg11_02266	LP_11__02296 hypothetical protein
sg11_02273	LP_11__02303 hypothetical protein
sg11_02387	LP_11__02419 hypothetical protein
sg11_02448	LP_11__02480 hypothetical protein
sg11_02495	LP_11__02527 hypothetical protein
sg11_02498	LP_11__02532 D-alanyl-lipoteichoic acid biosynthesis protein DltB
sg11_02524	LP_11__02559 hypothetical protein
sg11_02546	LP_11__02581 hypothetical protein
sg11_02548	LP_11__02583 hypothetical protein
sg11_02586	LP_11__02621 hypothetical protein
sg11_02598	LP_11__02633 hypothetical protein
sg11_02867	LP_11__02902 hypothetical protein
sg11_02869	LP_11__02904 hypothetical protein
sg11_02879	LP_11__02914 Transposase
sg11_02953	LP_11__02988 hypothetical protein
sg11_02957	LP_11__02992 hypothetical protein
sg11_02981	LP_11__03016 hypothetical protein
sg11_03018	LP_11__03054 hypothetical protein
sg11_03070	LP_11__03106 Transposase
sg11_03071	LP_11__03107 Transposase
sg11_03108	LP_11__03145 ubiquinone/menaquinone biosynthesis methyltransferase
sg11_03131	LP_11__03168 hypothetical protein

sg11_03154	LP_11__03191 hypothetical protein
sg11_03156	LP_11__03193 Dipeptide and tripeptide permease A
sg11_03157	LP_11__03194 hypothetical protein
sg11_03158	LP_11__03195 hypothetical protein
sg11_03161	LP_11__03198 hypothetical protein
sg11_03163	LP_11__03200 hypothetical protein
sg11_03164	LP_11__03201 hypothetical protein
sg11_03165	LP_11__03202 Transposase
sg11_03169	LP_11__03206 hypothetical protein
sg11_03176	LP_11__03213 hypothetical protein
sg12_00840	L_pne_12_D4955_00855 hypothetical protein
sg12_02887	L_pne_12_D4955_02923 hypothetical protein
sg13_00073	LP_13__00074 hypothetical protein
sg13_00074	LP_13__00075 hypothetical protein
sg13_00135	LP_13__00136 hypothetical protein
sg13_00200	LP_13__00201 hypothetical protein
sg13_00436	LP_13__00437 Transposase DDE domain protein
sg13_00684	LP_13__00691 hypothetical protein
sg13_00685	LP_13__00692 hypothetical protein
sg13_00686	LP_13__00693 hypothetical protein
sg13_00713	LP_13__00720 hypothetical protein
sg13_00914	LP_13__00922 Alcohol dehydrogenase
sg13_01048	LP_13__01059 Cyclic di-GMP phosphodiesterase Gmr
sg13_01143	LP_13__01154 hypothetical protein
sg13_01255	LP_13__01267 hypothetical protein
sg13_01256	LP_13__01268 hypothetical protein
sg13_01403	LP_13__01424 hypothetical protein
sg13_01462	LP_13__01484 hypothetical protein
sg13_01538	LP_13__01561 putative deoxyribonuclease YjjV
sg13_01539	LP_13__01562 hypothetical protein

sg13_01540	LP_13__01563 hypothetical protein
sg13_01541	LP_13__01564 putative P-loop ATPase
sg13_01546	LP_13__01569 hypothetical protein
sg13_01548	LP_13__01571 Type IV secretion system protein VirB11
sg13_01551	LP_13__01574 Pertussis toxin liberation protein E
sg13_01560	LP_13__01583 hypothetical protein
sg13_01563	LP_13__01586 hypothetical protein
sg13_01642	LP_13__01665 Dipeptide permease D
sg13_01643	LP_13__01666 hypothetical protein
sg13_01698	LP_13__01721 hypothetical protein
sg13_01919	LP_13__01942 thiazole synthase
sg13_02115	LP_13__02142 GIY-YIG nuclease superfamily protein
sg13_02166	LP_13__02193 hypothetical protein
sg13_02172	LP_13__02199 hypothetical protein
sg13_02217	LP_13__02244 hypothetical protein
sg13_02237	LP_13__02265 hypothetical protein
sg13_02282	LP_13__02311 hypothetical protein
sg13_02372	LP_13__02401 hypothetical protein
sg13_02373	LP_13__02402 hypothetical protein
sg13_02374	LP_13__02403 hypothetical protein
sg13_02429	LP_13__02458 hypothetical protein
sg13_02630	LP_13__02660 YcfA-like protein
sg13_02658	LP_13__02688 hypothetical protein
sg13_02711	LP_13__02741 hypothetical protein
sg13_02744	LP_13__02774 hypothetical protein
sg13_02894	LP_13__02924 hypothetical protein
sg13_02948	LP_13__02979 hypothetical protein
sg13_02986	LP_13__03017 hypothetical protein
sg13_02988	LP_13__03019 hypothetical protein
sg13_02989	LP_13__03020 hypothetical protein

sg13_03023	LP_13__03054 hypothetical protein
sg13_03047	LP_13__03078 hypothetical protein
sg13_03051	LP_13__03082 hypothetical protein
sg13_03056	LP_13__03087 putative secretion ATPase, PEP-CTERM locus subfamily
sg13_03057	LP_13__03088 Integrase core domain protein
sg13_03059	LP_13__03090 hypothetical protein
sg13_03064	LP_13__03095 hypothetical protein
sg13_03079	LP_13__03113 hypothetical protein
sg13_03080	LP_13__03114 N-hydroxyarylamine O-acetyltransferase
sg13_03085	LP_13__03119 hypothetical protein
sg13_03090	LP_13__03124 Opacity protein antigens
sg13_03104	LP_13__03142 S23 ribosomal protein
sg14_00004	LP_14__00004 hypothetical protein
sg14_00028	LP_14__00028 Efflux pump membrane transporter BepE
sg14_00038	LP_14__00038 Imidazolonepropionase
sg14_00139	LP_14__00139 type VI secretion protein IcmF
sg14_00177	LP_14__00177 Argininosuccinate lyase
sg14_00226	LP_14__00226 Conjugal transfer protein TraG
sg14_00232	LP_14__00232 hypothetical protein
sg14_00252	LP_14__00252 Beta-ketoacyl-acyl-carrier-protein synthase I
sg14_00395	LP_14__00395 hypothetical protein
sg14_00424	LP_14__00424 Threonine--tRNA ligase
sg14_00458	LP_14__00459 hypothetical protein
sg14_00578	LP_14__00579 DNA polymerase I
sg14_00726	LP_14__00728 Putative Mg2+ and Co2+ transporter CorB
sg14_00762	LP_14__00764 hypothetical protein
sg14_00817	LP_14__00819 Oxygen sensor protein DosP
sg14_00829	LP_14__00834 putative general secretion pathway protein YghD
sg14_00841	LP_14__00850 ATP-dependent Clp protease ATP-binding subunit ClpX
sg14_00925	LP_14__00937 DNA primase

sg14_01004	LP_14__01016 Galactose transporter
sg14_01066	LP_14__01078 hypothetical protein
sg14_01086	LP_14__01098 hypothetical protein
sg14_01100	LP_14__01112 hypothetical protein
sg14_01134	LP_14__01146 hypothetical protein
sg14_01150	LP_14__01162 hypothetical protein
sg14_01173	LP_14__01185 hypothetical protein
sg14_01215	LP_14__01227 hypothetical protein
sg14_01369	LP_14__01384 hypothetical protein
sg14_01456	LP_14__01474 hypothetical protein
sg14_01486	LP_14__01504 hypothetical protein
sg14_01494	LP_14__01512 Cation efflux system protein CzcA
sg14_01496	LP_14__01514 Cation efflux system protein CzcB
sg14_01539	LP_14__01557 Glutamate-1-semialdehyde 2,1-aminomutase
sg14_01561	LP_14__01579 ABC transporter arginine-binding protein 1 precursor
sg14_01611	LP_14__01629 hypothetical protein
sg14_01613	LP_14__01631 hypothetical protein
sg14_01659	LP_14__01679 hypothetical protein
sg14_01691	LP_14__01712 2-oxoglutarate oxidoreductase subunit KorA
sg14_01766	LP_14__01787 hypothetical protein
sg14_01813	LP_14__01834 hypothetical protein
sg14_01842	LP_14__01864 hypothetical protein
sg14_01908	LP_14__01930 hypothetical protein
sg14_01921	LP_14__01943 hypothetical protein
sg14_02034	LP_14__02056 hypothetical protein
sg14_02124	LP_14__02147 Glutamine--tRNA ligase
sg14_02194	LP_14__02220 Glucose-6-phosphate 1-dehydrogenase
sg14_02254	LP_14__02281 hypothetical protein
sg14_02384	LP_14__02411 hypothetical protein
sg14_02396	LP_14__02424 Distal rod protein

sg14_02445	LP_14__02473 hypothetical protein
sg14_02471	LP_14__02500 hypothetical protein
sg14_02478	LP_14__02507 putative acyltransferase YihG
sg14_02619	LP_14__02649 hypothetical protein
sg14_02684	LP_14__02714 hypothetical protein
sg14_02694	LP_14__02724 Linear gramicidin synthase subunit D
sg14_02722	LP_14__02752 hypothetical protein
sg14_02727	LP_14__02757 hypothetical protein
sg14_02763	LP_14__02794 hypothetical protein
sg14_02764	LP_14__02795 hypothetical protein
sg14_02765	LP_14__02796 Type I restriction-modification system methyltransferase subunit
sg14_02766	LP_14__02797 Eco57I restriction-modification methylase
sg14_02767	LP_14__02798 T5orf172 domain protein
sg14_02768	LP_14__02799 hypothetical protein
sg14_02777	LP_14__02808 hypothetical protein
sg14_02778	LP_14__02809 hypothetical protein
sg14_02784	LP_14__02815 hypothetical protein
sg14_02807	LP_14__02838 hypothetical protein
sg14_02816	LP_14__02847 hypothetical protein
sg14_02817	LP_14__02848 hypothetical protein
sg14_02824	LP_14__02855 putative transcriptional regulator
sg14_02825	LP_14__02856 Helix-turn-helix domain protein
sg14_02827	LP_14__02858 hypothetical protein
sg14_02836	LP_14__02868 Ran GTPase-activating protein (RanGAP) involved in mRNA processing and transport
sg14_02882	LP_14__02915 hypothetical protein
sg14_02892	LP_14__02925 Phosphoenolpyruvate carboxylase
sg14_02966	LP_14__02999 EamA-like transporter family protein
sg14_03057	LP_14__03093 hypothetical protein
sg14_03061	LP_14__03097 hypothetical protein
sg14_03088	LP_14__03124 hypothetical protein

sg14_03124	LP_14__03160 hypothetical protein
sg14_03133	LP_14__03169 hypothetical protein
sg14_03145	LP_14__03181 hypothetical protein
sg14_03152	LP_14__03188 hypothetical protein
sg14_03164	LP_14__03201 hypothetical protein
sg14_03165	LP_14__03202 hypothetical protein
sg14_03176	LP_14__03213 hypothetical protein
sg14_03186	LP_14__03223 hypothetical protein
sg15_00052	LP_15__00054 hypothetical protein
sg15_00058	LP_15__00060 Aminopeptidase N
sg15_00145	LP_15__00147 hypothetical protein
sg15_00187	LP_15__00189 Macrolide export ATP-binding/permease protein MacB
sg15_00302	LP_15__00305 hypothetical protein
sg15_00318	LP_15__00321 hypothetical protein
sg15_00506	LP_15__00511 hypothetical protein
sg15_00630	LP_15__00636 hypothetical protein
sg15_00649	LP_15__00655 hypothetical protein
sg15_00703	LP_15__00710 hypothetical protein
sg15_00748	LP_15__00764 hypothetical protein
sg15_00771	LP_15__00787 hypothetical protein
sg15_00778	LP_15__00794 Penicillin-binding protein 2B
sg15_00959	LP_15__00975 hypothetical protein
sg15_01024	LP_15__01040 hypothetical protein
sg15_01138	LP_15__01154 Imidazole glycerol phosphate synthase subunit HisH
sg15_01172	LP_15__01188 hypothetical protein
sg15_01236	LP_15__01255 hypothetical protein
sg15_01258	LP_15__01277 hypothetical protein
sg15_01354	LP_15__01373 hypothetical protein
sg15_01439	LP_15__01458 Aspartyl/glutamyl-tRNA(Asn/Gln) amidotransferase subunit B
sg15_01687	LP_15__01709 hypothetical protein

sg15_01689	LP_15__01711 hypothetical protein
sg15_01770	LP_15__01792 Morphology and auto-aggregation control protein
sg15_01774	LP_15__01796 Diaminopimelate decarboxylase
sg15_01784	LP_15__01806 hypothetical protein
sg15_01818	LP_15__01840 hypothetical protein
sg15_01849	LP_15__01871 hypothetical protein
sg15_01850	LP_15__01872 L,D-transpeptidase catalytic domain
sg15_01906	LP_15__01928 hypothetical protein
sg15_01979	LP_15__02002 Defects in Rab1 recruitment protein A
sg15_01993	LP_15__02016 hypothetical protein
sg15_02056	LP_15__02079 hypothetical protein
sg15_02063	LP_15__02086 2-oxoisovalerate dehydrogenase subunit alpha
sg15_02065	LP_15__02088 hypothetical protein
sg15_02152	LP_15__02176 hypothetical protein
sg15_02157	LP_15__02181 GTP-binding protein EngA
sg15_02179	LP_15__02203 hypothetical protein
sg15_02403	LP_15__02433 3D-(3,5/4)-trihydroxycyclohexane-1,2-dione hydrolase
sg15_02430	LP_15__02460 hypothetical protein
sg15_02495	LP_15__02526 succinate dehydrogenase, hydrophobic membrane anchor protein
sg15_02544	LP_15__02575 4-hydroxythreonine-4-phosphate dehydrogenase
sg15_02578	LP_15__02609 hypothetical protein
sg15_02598	LP_15__02629 integral membrane protein MviN
sg15_02706	LP_15__02737 hypothetical protein
sg15_02756	LP_15__02788 Cytochrome d ubiquinol oxidase subunit 2
sg15_02769	LP_15__02801 Coproporphyrinogen-III oxidase, aerobic
sg15_02826	LP_15__02859 hypothetical protein
sg15_02836	LP_15__02869 Bacteriophytochrome cph2
sg15_02839	LP_15__02872 hypothetical protein
sg15_02870	LP_15__02905 short chain dehydrogenase
sg15_02916	LP_15__02951 hypothetical protein

sg15_02924	LP_15__02959 hypothetical protein
sg15_02926	LP_15__02961 Fungalysin/Thermolysin Propeptide Motif
sg15_02927	LP_15__02962 hypothetical protein
sg15_02943	LP_15__02978 Cation efflux system protein CusA
sg15_02944	LP_15__02979 hypothetical protein
sg15_02982	LP_15__03018 hypothetical protein
sg15_03027	LP_15__03064 glutamine ABC transporter periplasmic protein
sg16_00237	LP_16_00238 Glutamate-1-semialdehyde 2,1-aminomutase
sg16_00398	LP_16_00399 hypothetical protein
sg16_00478	LP_16_00482 hypothetical protein
sg16_00527	LP_16_00531 hypothetical protein
sg16_00710	LP_16_00719 hypothetical protein
sg16_00734	LP_16_00743 glutathione-regulated potassium-efflux system protein KefC
sg16_00834	LP_16_00843 Phosphate transporter family protein
sg16_00848	LP_16_00857 acylglycerophosphoethanolamine acyltransferase
sg16_01238	LP_16_01250 hypothetical protein
sg16_01429	LP_16_01442 hypothetical protein
sg16_01442	LP_16_01455 Aerobic glycerol-3-phosphate dehydrogenase
sg16_01465	LP_16_01478 Phosphate acyltransferase
sg16_01523	LP_16_01536 L-serine dehydratase 1
sg16_01619	LP_16_01633 hypothetical protein
sg16_01651	LP_16_01665 hypothetical protein
sg16_01682	LP_16_01696 hypothetical protein
sg16_01697	LP_16_01711 hypothetical protein
sg16_01712	LP_16_01726 hypothetical protein
sg16_01752	LP_16_01766 hypothetical protein
sg16_01902	LP_16_01917 hypothetical protein
sg16_02255	LP_16_02281 Fimbrial assembly protein (PilN)
sg16_02325	LP_16_02351 hypothetical protein
sg16_02463	LP_16_02489 hypothetical protein

sg16_02614	LP_16_02644 hypothetical protein
sg16_02655	LP_16_02685 hypothetical protein
sg16_02739	LP_16_02771 hypothetical protein
sg16_02751	LP_16_02783 hypothetical protein
sg16_02811	LP_16_02845 hypothetical protein
sg16_02909	LP_16_02943 hypothetical protein
sg16_02914	LP_16_02948 hypothetical protein
sg16_02967	LP_16_03002 hypothetical protein
sg16_03001	LP_16_03037 hypothetical protein
sg16_03167	LP_16_03203 hypothetical protein
sg17_00285	LP_17__00288 sporadically distributed protein
sg17_00290	LP_17__00293 hypothetical protein
sg17_00291	LP_17__00294 hypothetical protein
sg17_00292	LP_17__00295 hypothetical protein
sg17_00294	LP_17__00297 hypothetical protein
sg17_00312	LP_17__00315 hypothetical protein
sg17_00327	LP_17__00330 hypothetical protein
sg17_00418	LP_17__00422 hypothetical protein
sg17_00513	LP_17__00519 hypothetical protein
sg17_00577	LP_17__00584 PAS domain S-box protein
sg17_00581	LP_17__00588 hypothetical protein
sg17_00760	LP_17__00770 Beta-lactamase
sg17_00761	LP_17__00771 hypothetical protein
sg17_01156	LP_17__01176 hypothetical protein
sg17_01161	LP_17__01181 hypothetical protein
sg17_01204	LP_17__01224 hypothetical protein
sg17_01326	LP_17__01346 hypothetical protein
sg17_01408	LP_17__01432 T5orf172 domain protein
sg17_01409	LP_17__01433 DNA phosphorothioation system restriction enzyme
sg17_01411	LP_17__01435 hypothetical protein

sg17_01413	LP_17__01437 hypothetical protein
sg17_01521	LP_17__01545 hypothetical protein
sg17_01604	LP_17__01629 hypothetical protein
sg17_01731	LP_17__01757 hypothetical protein
sg17_01732	LP_17__01758 hypothetical protein
sg17_01741	LP_17__01767 hypothetical protein
sg17_01764	LP_17__01790 hypothetical protein
sg17_01846	LP_17__01873 hypothetical protein
sg17_01916	LP_17__01943 hypothetical protein
sg17_01971	LP_17__01998 hypothetical protein
sg17_02010	LP_17__02040 hypothetical protein
sg17_02011	LP_17__02041 hypothetical protein
sg17_02033	LP_17__02063 Type IV secretion system protein virB4
sg17_02034	LP_17__02064 Pertussis toxin liberation protein C
sg17_02066	LP_17__02096 hypothetical protein
sg17_02068	LP_17__02098 hypothetical protein
sg17_02077	LP_17__02107 hypothetical protein
sg17_02081	LP_17__02111 hypothetical protein
sg17_02082	LP_17__02112 hypothetical protein
sg17_02346	LP_17__02376 hypothetical protein
sg17_02355	LP_17__02385 hypothetical protein
sg17_02356	LP_17__02386 hypothetical protein
sg17_02517	LP_17__02547 NAD-dependent dihydropyrimidine dehydrogenase subunit PreA
sg17_02620	LP_17__02652 Virulence sensor protein BvgS precursor
sg17_02691	LP_17__02723 hypothetical protein
sg17_02695	LP_17__02727 hypothetical protein
sg17_02697	LP_17__02729 SEC-C motif
sg17_02701	LP_17__02733 hypothetical protein
sg17_02702	LP_17__02734 Sel1 repeat
sg17_02703	LP_17__02735 hypothetical protein

sg17_02704	LP_17__02736 hypothetical protein
sg17_02728	LP_17__02760 serine/threonine protein kinase
sg17_02781	LP_17__02815 hypothetical protein
sg17_02782	LP_17__02816 hypothetical protein
sg17_02785	LP_17__02819 hypothetical protein
sg17_02786	LP_17__02820 hypothetical protein
sg17_02794	LP_17__02828 hypothetical protein
sg17_02795	LP_17__02829 hypothetical protein
sg17_02796	LP_17__02830 hypothetical protein
sg17_02858	LP_17__02893 Fatty acid desaturase
sg17_02860	LP_17__02895 hypothetical protein
sg17_02879	LP_17__02914 hypothetical protein
sg17_02940	LP_17__02975 efflux transporter, RND family, MFP subunit
sg17_03073	LP_17__03108 hypothetical protein
sg17_03074	LP_17__03109 hypothetical protein
sg17_03075	LP_17__03110 hypothetical protein
sg17_03101	LP_17__03136 hypothetical protein
sg17_03102	LP_17__03137 hypothetical protein
sg17_03104	LP_17__03139 hypothetical protein
sg17_03131	LP_17__03167 hypothetical protein
sg17_03213	LP_17__03249 hypothetical protein
sg17_03222	LP_17__03258 hypothetical protein
sg17_03223	LP_17__03259 hypothetical protein
sg17_03227	LP_17__03263 hypothetical protein
sg17_03234	LP_17__03270 hypothetical protein
sg2_00248	LP_02_00254 PAS domain S-box protein
sg2_00341	LP_02_00347 hypothetical protein
sg2_00359	LP_02_00365 hypothetical protein
sg2_00374	LP_02_00380 Inner membrane protein YejM
sg2_00378	LP_02_00384 hypothetical protein

sg2_00492	LP_02_00499 hypothetical protein
sg2_00701	LP_02_00712 hypothetical protein
sg2_00762	LP_02_00773 bifunctional regulator KidO
sg2_00766	LP_02_00777 methionyl-tRNA formyltransferase
sg2_00774	LP_02_00785 hypothetical protein
sg2_00775	LP_02_00786 Phytanoyl-CoA dioxygenase (PhyH)
sg2_00777	LP_02_00788 hypothetical protein
sg2_00909	LP_02_00923 hypothetical protein
sg2_01003	LP_02_01018 putative membrane protein
sg2_01189	LP_02_01205 hypothetical protein
sg2_01239	LP_02_01255 hypothetical protein
sg2_01366	LP_02_01383 putative protein involved in outer membrane biogenesis
sg2_01413	LP_02_01430 hypothetical protein
sg2_01673	LP_02_01693 hypothetical protein
sg2_01706	LP_02_01726 hypothetical protein
sg2_01754	LP_02_01775 hypothetical protein
sg2_01841	LP_02_01862 Dual-specificity RNA methyltransferase RlmN
sg2_01867	LP_02_01888 General secretion pathway protein F
sg2_01942	LP_02_01963 50S ribosomal protein L9
sg2_02039	LP_02_02067 Sodium:dicarboxylate symporter family protein
sg2_02041	LP_02_02069 hypothetical protein
sg2_02059	LP_02_02087 Outer membrane protein TolC precursor
sg2_02171	LP_02_02200 PhoH-like protein
sg2_02180	LP_02_02209 hypothetical protein
sg2_02244	LP_02_02273 hypothetical protein
sg2_02245	LP_02_02274 hypothetical protein
sg2_02446	LP_02_02476 putative NTPase (NACHT family)
sg2_02457	LP_02_02487 hypothetical protein
sg2_02475	LP_02_02505 secondary thiamine-phosphate synthase enzyme
sg2_02624	LP_02_02656 hypothetical protein

sg2_02645	LP_02_02677 hypothetical protein
sg2_02738	LP_02_02773 oxidoreductase
sg2_02747	LP_02_02782 Phosphoribosylaminoimidazole-succinocarboxamide synthase
sg2_02767	LP_02_02803 hypothetical protein
sg2_02874	LP_02_02911 putative metabolite transport protein CsbC
sg2_02891	LP_02_02928 hypothetical protein
sg2_02968	LP_02_03006 hypothetical protein
sg2_02982	LP_02_03020 hypothetical protein
sg3_00003	sg3_00003 Fatty acid oxidation complex subunit alpha
sg3_00005	sg3_00005 multifunctional fatty acid oxidation complex subunit alpha
sg3_00024	sg3_00026 hypothetical protein
sg3_00386	sg3_00398 hypothetical protein
sg3_00573	sg3_00587 Transposase, TnpA family
sg3_00708	sg3_00723 hypothetical protein
sg3_00910	sg3_00926 hypothetical protein
sg3_01030	sg3_01049 Efflux pump membrane transporter BepG
sg3_01156	sg3_01176 hypothetical protein
sg3_01158	sg3_01178 hypothetical protein
sg3_01285	sg3_01308 23S rRNA (guanosine-2'-O-)-methyltransferase RlmB
sg3_01319	sg3_01343 hypothetical protein
sg3_01669	sg3_01695 Dot/Icm substrate protein
sg3_01713	sg3_01739 hypothetical protein
sg3_01900	sg3_01927 hypothetical protein
sg3_02149	sg3_02179 Biotin carboxylase
sg3_02280	sg3_02310 hypothetical protein
sg3_02488	sg3_02522 multidrug resistance protein MdtN
sg3_02789	sg3_02823 hypothetical protein
sg3_02872	sg3_02907 hypothetical protein
sg4_00420	LP_04_00423 hypothetical protein
sg4_00677	LP_04_00682 hypothetical protein

sg4_00792	LP_04_00798 hypothetical protein
sg4_01129	LP_04_01137 hypothetical protein
sg4_01131	LP_04_01139 hypothetical protein
sg4_01133	LP_04_01141 hypothetical protein
sg4_01136	LP_04_01144 hypothetical protein
sg4_01139	LP_04_01147 hypothetical protein
sg4_01140	LP_04_01148 hypothetical protein
sg4_01141	LP_04_01149 hypothetical protein
sg4_01142	LP_04_01150 hypothetical protein
sg4_01146	LP_04_01154 hypothetical protein
sg4_01912	LP_04_01937 hypothetical protein
sg4_01932	LP_04_01957 Methyltransferase domain protein
sg4_01944	LP_04_01969 hypothetical protein
sg4_01955	LP_04_01980 hypothetical protein
sg4_01968	LP_04_01993 Periplasmic mercury ion-binding protein
sg4_02311	LP_04_02337 hypothetical protein
sg4_02313	LP_04_02339 hypothetical protein
sg4_02468	LP_04_02495 hypothetical protein
sg4_02487	LP_04_02514 hypothetical protein
sg4_02494	LP_04_02521 hypothetical protein
sg4_02569	LP_04_02596 hypothetical protein
sg4_02652	LP_04_02680 Diaminopimelate epimerase
sg4_03032	LP_04_03066 hypothetical protein
sg4_03040	LP_04_03074 hypothetical protein
sg4_03042	LP_04_03076 hypothetical protein
sg4_03044	LP_04_03078 hypothetical protein
sg4_03084	LP_04_03119 hypothetical protein
sg5_00114	LP_05_00115 hypothetical protein
sg5_00181	LP_05_00183 Isoleucine--tRNA ligase
sg5_00185	LP_05_00187 hypothetical protein

sg5_00835	LP_05_00843 hypothetical protein
sg5_00841	LP_05_00849 hypothetical protein
sg5_00843	LP_05_00851 hypothetical protein
sg5_00935	LP_05_00943 Hydrogenase expression/formation protein HypE
sg5_01262	LP_05_01275 hypothetical protein
sg5_01277	LP_05_01290 short chain dehydrogenase
sg5_01427	LP_05_01443 hypothetical protein
sg5_01498	LP_05_01514 Methionine aminopeptidase
sg5_01697	LP_05_01723 Endoglucanase C precursor
sg5_01709	LP_05_01736 hypothetical protein
sg5_01801	LP_05_01829 hypothetical protein
sg5_01831	LP_05_01860 hypothetical protein
sg5_01945	LP_05_01975 putative cation-transporting ATPase F
sg5_01970	LP_05_02000 hypothetical protein
sg5_02015	LP_05_02045 hypothetical protein
sg5_02031	LP_05_02061 hypothetical protein
sg5_02155	LP_05_02185 p-hydroxybenzoic acid efflux pump subunit AaeA
sg5_02182	LP_05_02212 hypothetical protein
sg5_02406	LP_05_02441 hypothetical protein
sg5_02797	LP_05_02834 guanine deaminase
sg5_02825	LP_05_02862 Histamine oxidase
sg5_02859	LP_05_02896 hypothetical protein
sg5_02972	LP_05_03009 hypothetical protein
sg5_03010	LP_05_03047 hypothetical protein
sg5_03145	LP_05_03183 trans-aconitate 2-methyltransferase
sg5_03152	LP_05_03190 putative acyl transferase
sg5_03153	LP_05_03191 hypothetical protein
sg6_00054	LP_06_00063 2-oxoglutarate carboxylase large subunit
sg6_00078	LP_06_00087 Delta-aminolevulinic acid dehydratase
sg6_00126	LP_06_00135 putative response regulatory protein

sg6_00400	LP_06_00411 Pyruvate dehydrogenase E1 component
sg6_00533	LP_06_00544 hypothetical protein
sg6_00746	LP_06_00761 CDP-Glycerol:Poly(glycerophosphate) glycerophosphotransferase
sg6_00773	LP_06_00789 ATP-dependent Clp protease ATP-binding subunit ClpA
sg6_00948	LP_06_00966 conjugative transfer ATPase, family
sg6_01013	LP_06_01031 hypothetical protein
sg6_01040	LP_06_01058 Putative prophage CPS-53 integrase
sg6_01052	LP_06_01070 hypothetical protein
sg6_01246	LP_06_01266 hypothetical protein
sg6_01361	LP_06_01381 hypothetical protein
sg6_01381	LP_06_01401 hypothetical protein
sg6_01412	LP_06_01432 hypothetical protein
sg6_01422	LP_06_01442 hypothetical protein
sg6_01442	LP_06_01462 tRNA-dihydrouridine synthase A
sg6_01539	LP_06_01559 D-cysteine desulfhydrase
sg6_01550	LP_06_01570 Type II secretory pathway, component PulK
sg6_01558	LP_06_01578 putative 3-hydroxyacyl-CoA dehydrogenase
sg6_01583	LP_06_01603 hypothetical protein
sg6_01698	LP_06_01718 Outer membrane protein OprM precursor
sg6_01844	LP_06_01864 hypothetical protein
sg6_01939	LP_06_01962 Modulator of FtsH protease HflC
sg6_02083	LP_06_02108 hypothetical protein
sg6_02084	LP_06_02109 hypothetical protein
sg6_02085	LP_06_02110 hypothetical protein
sg6_02106	LP_06_02131 type I secretion outer membrane protein, TolC family
sg6_02107	LP_06_02132 Cation efflux system protein CzcC
sg6_02306	LP_06_02335 hypothetical protein
sg6_02343	LP_06_02372 hypothetical protein
sg6_02464	LP_06_02494 DNA topoisomerase 1
sg6_02631	LP_06_02663 hypothetical protein

sg6_02676	LP_06_02709 (Dimethylallyl)adenosine tRNA methylthiotransferase MiaB
sg6_02696	LP_06_02729 hypothetical protein
sg6_02757	LP_06_02790 Major Facilitator Superfamily protein
sg6_02809	LP_06_02843 putative 3-hydroxybutyryl-CoA dehydrogenase
sg6_02814	LP_06_02848 Low-affinity cAMP phosphodiesterase
sg6_02825	LP_06_02859 hypothetical protein
sg6_02828	LP_06_02862 hypothetical protein
sg6_02838	LP_06_02872 hypothetical protein
sg6_02946	LP_06_02980 Bacterial leucyl aminopeptidase precursor
sg6_02947	LP_06_02981 hypothetical protein
sg6_02953	LP_06_02987 hypothetical protein
sg6_02978	LP_06_03012 hypothetical protein
sg6_03007	LP_06_03041 ApaLI-like restriction endonuclease
sg6_03010	LP_06_03044 hypothetical protein
sg6_03038	LP_06_03072 hypothetical protein
sg6_03054	LP_06_03088 Dot/Icm substrate protein
sg6_03099	LP_06_03133 hypothetical protein
sg6_03105	LP_06_03140 hypothetical protein
sg7_00012	sg7_00013 hypothetical protein
sg7_00298	sg7_00309 hypothetical protein
sg7_00537	sg7_00550 Adenosine monophosphate-protein hydrolase SidD
sg7_00584	sg7_00597 hypothetical protein
sg7_00655	sg7_00668 hypothetical protein
sg7_00849	sg7_00867 Aldehyde dehydrogenase, thermostable
sg7_00850	sg7_00868 1-deoxy-D-xylulose-5-phosphate synthase
sg7_01013	sg7_01033 hypothetical protein
sg7_01081	sg7_01102 hypothetical protein
sg7_01084	sg7_01105 Cation efflux system protein CzcA
sg7_01085	sg7_01106 Cation efflux system protein CzcA
sg7_01191	sg7_01212 Transposase DDE domain protein

sg7_01748	sg7_01777 hypothetical protein
sg7_01924	sg7_01955 Protein of unknown function (Hypoth_ymh)
sg7_01925	sg7_01956 hypothetical protein
sg7_02017	sg7_02048 hypothetical protein
sg7_02021	sg7_02052 hypothetical protein
sg7_02022	sg7_02053 hypothetical protein
sg7_02582	sg7_02619 hypothetical protein
sg7_02597	sg7_02634 hypothetical protein
sg7_02640	sg7_02677 hypothetical protein
sg7_02941	sg7_02978 hypothetical protein
sg7_02942	sg7_02979 hypothetical protein
sg7_02945	sg7_02982 hypothetical protein
sg7_02947	sg7_02984 hypothetical protein
sg7_02949	sg7_02986 hypothetical protein
sg7_02950	sg7_02987 hypothetical protein
sg7_02951	sg7_02988 Exodeoxyribonuclease 8
sg7_02952	sg7_02989 hypothetical protein
sg7_02953	sg7_02990 hypothetical protein
sg7_02954	sg7_02991 hypothetical protein
sg7_02955	sg7_02992 Terminase-like family protein
sg7_02956	sg7_02993 Crossover junction endodeoxyribonuclease RusA
sg7_02957	sg7_02994 Peptidase M15
sg7_02958	sg7_02995 hypothetical protein
sg7_02959	sg7_02996 hypothetical protein
sg7_02960	sg7_02997 hypothetical protein
sg7_02961	sg7_02998 hypothetical protein
sg7_02962	sg7_02999 chromosome segregation protein
sg7_02963	sg7_03000 hypothetical protein
sg7_02964	sg7_03001 hypothetical protein
sg7_02965	sg7_03002 hypothetical protein

sg7_02966	sg7_03003 hypothetical protein
sg7_02967	sg7_03004 hypothetical protein
sg7_02968	sg7_03005 hypothetical protein
sg7_02969	sg7_03006 hypothetical protein
sg7_02970	sg7_03007 hypothetical protein
sg7_02971	sg7_03008 Bacteriophage head to tail connecting protein
sg7_02972	sg7_03009 hypothetical protein
sg7_02973	sg7_03010 hypothetical protein
sg7_02974	sg7_03011 hypothetical protein
sg7_02975	sg7_03012 hypothetical protein
sg7_02976	sg7_03013 hypothetical protein
sg7_02977	sg7_03014 hypothetical protein
sg7_02978	sg7_03015 hypothetical protein
sg7_02979	sg7_03016 hypothetical protein
sg7_02980	sg7_03017 hypothetical protein
sg7_02981	sg7_03018 hypothetical protein
sg7_02983	sg7_03020 hypothetical protein
sg7_03081	sg7_03118 hypothetical protein
sg7_03083	sg7_03120 hypothetical protein
sg7_03084	sg7_03121 hypothetical protein
sg7_03130	sg7_03168 Macrocin-O-methyltransferase (TylF)
sg7_03131	sg7_03169 HAD phosphoserine phosphatase-like hydrolase, family IB
sg7_03132	sg7_03170 4-hydroxy-tetrahydrodipicolinate synthase
sg7_03133	sg7_03171 N-acylneuraminate cytidylyltransferase
sg7_03136	sg7_03174 hypothetical protein
sg7_03140	sg7_03178 hypothetical protein
sg8_00005	sg8_00005 hypothetical protein
sg8_00017	sg8_00017 hypothetical protein
sg8_00180	sg8_00180 hypothetical protein
sg8_00186	sg8_00186 T5orf172 domain protein

sg8_00187	sg8_00187 hypothetical protein
sg8_00280	sg8_00282 hypothetical protein
sg8_00556	sg8_00560 hypothetical protein
sg8_00559	sg8_00563 hypothetical protein
sg8_00601	sg8_00605 hypothetical protein
sg8_00621	sg8_00625 hypothetical protein
sg8_00820	sg8_00828 Site-specific recombinase XerD
sg8_01118	sg8_01127 Chitinase
sg8_01145	sg8_01154 hypothetical protein
sg8_01146	sg8_01155 hypothetical protein
sg8_01147	sg8_01156 hypothetical protein
sg8_01246	sg8_01257 hypothetical protein
sg8_01247	sg8_01258 hypothetical protein
sg8_01362	sg8_01373 hypothetical protein
sg8_01576	sg8_01591 hypothetical protein
sg8_01589	sg8_01604 hypothetical protein
sg8_01784	sg8_01800 hypothetical protein
sg8_01942	sg8_01958 hypothetical protein
sg8_02132	sg8_02155 hypothetical protein
sg8_02141	sg8_02164 hypothetical protein
sg8_02208	sg8_02231 hypothetical protein
sg8_02258	sg8_02283 hypothetical protein
sg8_02282	sg8_02307 hypothetical protein
sg8_02383	sg8_02408 Dot/Icm substrate protein
sg8_02458	sg8_02487 ATP-dependent DNA helicase PcrA
sg8_02459	sg8_02488 hypothetical protein
sg8_02481	sg8_02510 hypothetical protein
sg8_02597	sg8_02628 2-oxoglutarate carboxylase small subunit
sg8_02688	sg8_02720 Transposase Tn5 dimerisation domain protein
sg8_02689	sg8_02721 hypothetical protein

sg8_02708	sg8_02740 hypothetical protein
sg8_02709	sg8_02741 hypothetical protein
sg8_02843	sg8_02876 hypothetical protein
sg8_02844	sg8_02877 hypothetical protein
sg8_02921	sg8_02959 hypothetical protein
sg8_02932	sg8_02970 hypothetical protein
sg8_02985	sg8_03023 Site-specific recombinase XerD
sg8_03046	sg8_03084 hypothetical protein
sg9_00094	sg9_00095 hypothetical protein
sg9_00102	sg9_00103 conjugal transfer protein TrbD
sg9_00229	sg9_00233 hypothetical protein
sg9_00230	sg9_00234 hypothetical protein
sg9_00444	sg9_00448 hypothetical protein
sg9_00814	sg9_00820 hypothetical protein
sg9_00980	sg9_00987 hypothetical protein
sg9_01068	sg9_01075 nuclease NucT
sg9_01069	sg9_01076 hypothetical protein
sg9_01070	sg9_01077 hypothetical protein
sg9_01073	sg9_01080 hypothetical protein
sg9_01074	sg9_01081 hypothetical protein
sg9_01078	sg9_01085 hypothetical protein
sg9_01100	sg9_01107 hypothetical protein
sg9_01138	sg9_01145 Conjugal transfer protein TraG
sg9_01258	sg9_01267 conjugal transfer protein TraL
sg9_01260	sg9_01269 Relaxosome protein
sg9_01261	sg9_01270 conjugal transfer relaxase TraI
sg9_01264	sg9_01273 hypothetical protein
sg9_01266	sg9_01275 Conjugal transfer protein TraG
sg9_01499	sg9_01513 hypothetical protein
sg9_01948	sg9_01973 hypothetical protein

sg9_02003	sg9_02030 Growth regulator
sg9_02004	sg9_02031 mRNA interferase MazF
sg9_02105	sg9_02133 hypothetical protein
sg9_02148	sg9_02176 hypothetical protein
sg9_02321	sg9_02351 hypothetical protein
sg9_02515	sg9_02548 FG-GAP repeat
sg9_02518	sg9_02551 Transposase IS66 family protein
sg9_02519	sg9_02552 hypothetical protein
sg9_02632	sg9_02665 tyramine oxidase
sg9_02772	sg9_02810 Bacterial regulatory proteins, luxR family
sg9_02806	sg9_02844 hypothetical protein
sg9_02810	sg9_02848 hypothetical protein
sg9_02821	sg9_02859 tropinone reductase
Thunder Bay00781	Thunder Bay_00787 Transposase for transposon Tn5
Thunder Bay01307	Thunder Bay_01318 hypothetical protein
Thunder Bay01308	Thunder Bay_01319 hypothetical protein
Thunder Bay01938	Thunder Bay_01961 hypothetical protein
Thunder Bay01939	Thunder Bay_01962 putative transposase OrfB
Thunder Bay02133	Thunder Bay_02160 hypothetical protein
Thunder Bay02192	Thunder Bay_02219 Transposase
Thunder Bay02242	Thunder Bay_02269 hypothetical protein
Thunder Bay02848	Thunder Bay_02882 hypothetical protein

Supplementary Table 3b. Unique genes/gene clusters found only in the Clade 2 Legionella species

Birmingham01178	PROKKA_01198 hypothetical protein
Birmingham00121	PROKKA_00121 hypothetical protein
Jordan01398	Jordan_01416 hypothetical protein
mac00435	mac_D5800_00447 hypothetical protein
Birmingham00417	PROKKA_00421 hypothetical protein
Birmingham00832	PROKKA_00848 Inner membrane protein YdcO

Birmingham00982	PROKKA_00999 homoserine kinase
Birmingham01003	PROKKA_01020 Histidine-specific methyltransferase EgtD
Birmingham01131	PROKKA_01150 Multidrug transporter MdtA
Birmingham01132	PROKKA_01151 Efflux pump membrane transporter BepE
Birmingham01413	PROKKA_01435 Undecaprenyl phosphate-alpha-4-amino-4-deoxy-L-arabinose arabinosyl transferase
Birmingham01437	PROKKA_01459 Phosphate acetyltransferase
Birmingham01517	PROKKA_01539 EVE domain protein
Birmingham01610	PROKKA_01632 Trk system potassium uptake protein TrkH
Birmingham01611	PROKKA_01633 Trk system potassium uptake protein TrkA
Birmingham01747	PROKKA_01769 hypothetical protein
Birmingham02146	PROKKA_02175 Nitrogen regulatory protein
Birmingham02449	PROKKA_02478 nicotinamide riboside transporter PnuC
Birmingham02574	PROKKA_02605 hypothetical protein
Birmingham02944	PROKKA_02977 hypothetical protein
Jamestown01890	PROKKA_01917 hypothetical protein
Birmingham00093	PROKKA_00093 hypothetical protein
Birmingham00764	PROKKA_00780 hypothetical protein
Birmingham00782	PROKKA_00798 Polysaccharide biosynthesis protein
Birmingham01006	PROKKA_01023 PAP2 superfamily protein
Birmingham02126	PROKKA_02155 hypothetical protein
Birmingham02295	PROKKA_02324 hypothetical protein
Birmingham03311	PROKKA_03348 Transposase
Jamestown00033	PROKKA_00033 hypothetical protein
Jamestown00445	PROKKA_00448 Histone deacetylase-like amidohydrolase
Jamestown00713	PROKKA_00718 hypothetical protein
Jamestown00780	PROKKA_00786 Alpha-ketoglutarate permease
Jamestown01195	PROKKA_01208 hypothetical protein
Jamestown01576	PROKKA_01601 LysR substrate binding domain protein
Jamestown02226	PROKKA_02256 hypothetical protein
Jamestown02812	PROKKA_02846 Branched-chain-amino-acid aminotransferase

Jamestown02814	PROKKA_02848 hypothetical protein
Jordan00241	Jordan_00241 hypothetical protein
mac00602	mac_D5800_00616 Ankyrin repeats (3 copies)
mac03158	mac_D5800_03194 hypothetical protein
Birmingham01186	PROKKA_01206 2,4-dichlorophenol 6-monooxygenase
Birmingham01191	PROKKA_01211 Endoglucanase precursor
Birmingham01338	PROKKA_01360 hypothetical protein
Birmingham01845	PROKKA_01868 hypothetical protein
Birmingham01957	PROKKA_01983 hypothetical protein
Birmingham02971	PROKKA_03004 hypothetical protein
Jamestown00282	PROKKA_00284 Thioredoxin-related protein
Jamestown00407	PROKKA_00410 Response regulator of citrate/malate metabolism
Jamestown00673	PROKKA_00678 hypothetical protein
Jamestown00679	PROKKA_00684 hypothetical protein
Jamestown00835	PROKKA_00841 hypothetical protein
Jamestown00903	PROKKA_00910 ADP-ribose pyrophosphatase
Jamestown01191	PROKKA_01204 P-protein
Jamestown01432	PROKKA_01454 Putative molybdenum carrier
Jamestown01534	PROKKA_01559 hypothetical protein
Jamestown02194	PROKKA_02224 CBS domain protein
Jamestown02286	PROKKA_02317 Sulfurtransferase TusA
Jamestown02287	PROKKA_02318 tRNA-specific 2-thiouridylase MnmA
Jamestown02572	PROKKA_02605 Ribokinase
Jamestown03171	PROKKA_03206 Aspartokinase
Jamestown03302	PROKKA_03339 conjugal transfer protein TrbP
Birmingham00164	PROKKA_00165 hypothetical protein
Birmingham00226	PROKKA_00227 hypothetical protein
Birmingham00649	PROKKA_00664 biotin biosynthesis protein BioC
Birmingham00679	PROKKA_00694 hypothetical protein
Birmingham01135	PROKKA_01154 hypothetical protein

Birmingham01521	PROKKA_01543 hypothetical protein
Birmingham02084	PROKKA_02113 hypothetical protein
Birmingham02235	PROKKA_02264 hypothetical protein
Birmingham02517	PROKKA_02548 Acyl-CoA thioester hydrolase YbgC
Birmingham02935	PROKKA_02968 IS2 repressor TnpA
Birmingham03074	PROKKA_03109 hypothetical protein
Jamestown00127	PROKKA_00127 hypothetical protein
Jamestown00252	PROKKA_00254 hypothetical protein
Jamestown00311	PROKKA_00313 L,D-transpeptidase catalytic domain
Jamestown00797	PROKKA_00803 hypothetical protein
Jamestown01022	PROKKA_01029 Beta-phosphoglucomutase
Jamestown01078	PROKKA_01086 hypothetical protein
Jamestown01676	PROKKA_01701 hypothetical protein
Jamestown02675	PROKKA_02708 hypothetical protein
Jamestown02885	PROKKA_02919 Linear gramicidin synthase subunit D
Jamestown03250	PROKKA_03285 NADP-dependent 3-hydroxy acid dehydrogenase YdfG
Jordan00273	Jordan_00273 Phosphoadenosine phosphosulfate reductase family protein
Jordan00504	Jordan_00515 hypothetical protein
brunen01892	PROKKA_01917 hypothetical protein
mac03063	mac_D5800_03098 hypothetical protein
Birmingham00007	PROKKA_00007 Ran GTPase-activating protein (RanGAP) involved in mRNA processing and transport
Birmingham00098	PROKKA_00098 hypothetical protein
Birmingham00135	PROKKA_00135 hypothetical protein
Birmingham00141	PROKKA_00141 hypothetical protein
Birmingham00238	PROKKA_00239 hypothetical protein
Birmingham00351	PROKKA_00355 Acriflavine resistance protein B
Birmingham00352	PROKKA_00356 Acriflavine resistance protein A precursor
Birmingham00357	PROKKA_00361 putative acetyltransferase
Birmingham00607	PROKKA_00621 putative integral membrane protein
Birmingham00801	PROKKA_00817 Acetyl-CoA acetyltransferase

Birmingham00877	PROKKA_00893 hypothetical protein
Birmingham00888	PROKKA_00904 DNA-binding transcriptional dual regulator Crp
Birmingham01001	PROKKA_01018 hypothetical protein
Birmingham01086	PROKKA_01105 Serine/threonine-protein kinase B
Birmingham01557	PROKKA_01579 hypothetical protein
Birmingham01717	PROKKA_01739 3'(2'),5'-bisphosphate nucleotidase CysQ
Birmingham01984	PROKKA_02013 hypothetical protein
Birmingham02238	PROKKA_02267 hypothetical protein
Birmingham02274	PROKKA_02303 sucrose/H+ symporter
Birmingham02711	PROKKA_02742 8-oxoguanine deaminase
Birmingham02965	PROKKA_02998 Lipoprotein signal peptidase
Jamestown00082	PROKKA_00082 Glycine/sarcosine/dimethylglycine N-methyltransferase
Jamestown00111	PROKKA_00111 hypothetical protein
Jamestown00188	PROKKA_00188 hypothetical protein
Jamestown00234	PROKKA_00235 hypothetical protein
Jamestown00299	PROKKA_00301 hypothetical protein
Jamestown00422	PROKKA_00425 hypothetical protein
Jamestown00533	PROKKA_00536 hypothetical protein
Jamestown00563	PROKKA_00566 hypothetical protein
Jamestown00589	PROKKA_00592 hypothetical protein
Jamestown00602	PROKKA_00605 Antitoxin igA-2
Jamestown00638	PROKKA_00641 lipoprotein NlpI
Jamestown01073	PROKKA_01081 hypothetical protein
Jamestown01158	PROKKA_01166 hypothetical protein
Jamestown01166	PROKKA_01175 hybrid sensory histidine kinase BarA
Jamestown01173	PROKKA_01185 hypothetical protein
Jamestown01215	PROKKA_01228 hypothetical protein
Jamestown01240	PROKKA_01253 hypothetical protein
Jamestown01481	PROKKA_01506 carboxylate/amino acid/amine transporter
Jamestown01777	PROKKA_01803 hypothetical protein

Jamestown01882	PROKKA_01909 Osmotically-inducible protein Y precursor
Jamestown01994	PROKKA_02023 isoaspartyl dipeptidase
Jamestown02384	PROKKA_02416 hypothetical protein
Jamestown02465	PROKKA_02498 type 2 lantibiotic biosynthesis protein LanM
Jamestown02744	PROKKA_02778 hypothetical protein
Jamestown02820	PROKKA_02854 hypothetical protein
Jamestown02895	PROKKA_02929 hypothetical protein
Jamestown03058	PROKKA_03093 hypothetical protein
Jordan00033	Jordan_00033 Aspartyl/Asparaginyl beta-hydroxylase
Jordan00925	Jordan_00940 Acetyltransferase Pat
Jordan01747	Jordan_01768 hypothetical protein
Jordan02741	Jordan_02776 ABC-type uncharacterized transport system, periplasmic component
brunen01342	PROKKA_01354 hypothetical protein
brunen02332	PROKKA_02365 Alpha/beta hydrolase family protein
brunen02567	PROKKA_02600 NADP-dependent alcohol dehydrogenase C 2
erythra00418	PROKKA_00421 hypothetical protein
erythra01024	PROKKA_01032 hypothetical protein
mac02702	mac_D5800_02736 Phosphocholine transferase AnkX
Birmingham00043	PROKKA_00043 hypothetical protein
Birmingham00125	PROKKA_00125 hypothetical protein
Birmingham00155	PROKKA_00156 hypothetical protein
Birmingham00287	PROKKA_00291 hypothetical protein
Birmingham00481	PROKKA_00486 hypothetical protein
Birmingham00771	PROKKA_00787 hypothetical protein
Birmingham01123	PROKKA_01142 3-phytase (myo-inositol-hexaphosphate 3-phosphohydrolase)
Birmingham01220	PROKKA_01240 hypothetical protein
Birmingham01362	PROKKA_01384 hypothetical protein
Birmingham01433	PROKKA_01455 Transaldolase
Birmingham01491	PROKKA_01513 Ran GTPase-activating protein (RanGAP) involved in mRNA processing and transport
Birmingham01492	PROKKA_01514 Defects in Rab1 recruitment protein A

Birmingham01505	PROKKA_01527 Modulator of FtsH protease HflK
Birmingham01554	PROKKA_01576 hypothetical protein
Birmingham01725	PROKKA_01747 hypothetical protein
Birmingham01738	PROKKA_01760 Phosphatidylcholine-sterol acyltransferase precursor
Birmingham02165	PROKKA_02194 hypothetical protein
Birmingham02247	PROKKA_02276 hypothetical protein
Birmingham02650	PROKKA_02681 hypothetical protein
Birmingham02718	PROKKA_02749 Patatin
Birmingham02906	PROKKA_02939 hypothetical protein
Birmingham03108	PROKKA_03143 hypothetical protein
Birmingham03139	PROKKA_03174 hypothetical protein
Birmingham03273	PROKKA_03309 hypothetical protein
Jamestown00005	PROKKA_00005 hypothetical protein
Jamestown00060	PROKKA_00060 homoserine kinase
Jamestown00159	PROKKA_00159 hypothetical protein
Jamestown00251	PROKKA_00253 hypothetical protein
Jamestown00285	PROKKA_00287 Ribose-phosphate pyrophosphokinase
Jamestown00398	PROKKA_00401 hypothetical protein
Jamestown00459	PROKKA_00462 hypothetical protein
Jamestown00726	PROKKA_00731 putative acetyltransferase
Jamestown00864	PROKKA_00870 hypothetical protein
Jamestown00884	PROKKA_00891 hypothetical protein
Jamestown00907	PROKKA_00914 hypothetical protein
Jamestown00913	PROKKA_00920 hypothetical protein
Jamestown00986	PROKKA_00993 hypothetical protein
Jamestown01013	PROKKA_01020 hypothetical protein
Jamestown01084	PROKKA_01092 hypothetical protein
Jamestown01164	PROKKA_01173 NADH:flavorubredoxin oxidoreductase
Jamestown01179	PROKKA_01192 hypothetical protein
Jamestown01311	PROKKA_01324 hypothetical protein

Jamestown01330	PROKKA_01343 hypothetical protein
Jamestown01360	PROKKA_01373 hypothetical protein
Jamestown01382	PROKKA_01398 hypothetical protein
Jamestown01473	PROKKA_01498 Molybdenum cofactor biosynthesis protein C
Jamestown01625	PROKKA_01650 (R)-stereoselective amidase
Jamestown01667	PROKKA_01692 Sensory/regulatory protein RpfC
Jamestown01889	PROKKA_01916 hypothetical protein
Jametowns01902	PROKKA_01929 hypothetical protein
Jamestown01905	PROKKA_01932 hypothetical protein
Jamestown01906	PROKKA_01933 Voltage-gated ClC-type chloride channel ClcB
Jamestown01934	PROKKA_01961 Phasin protein
Jamestown01946	PROKKA_01973 hypothetical protein
Jamestown02228	PROKKA_02258 hypothetical protein
Jamestown02314	PROKKA_02345 hypothetical protein
Jamestown02432	PROKKA_02465 chaperone protein DnaJ
Jamestown02507	PROKKA_02540 hypothetical protein
Jamestown02685	PROKKA_02718 hypothetical protein
Jamestown02745	PROKKA_02779 Histidine ammonia-lyase
Jamestown02751	PROKKA_02785 hypothetical protein
Jamestown02792	PROKKA_02826 hypothetical protein
Jamestown03069	PROKKA_03104 hypothetical protein
Jamestown03139	PROKKA_03174 hypothetical protein
Jamestown03239	PROKKA_03274 Bacterioferritin (cytochrome b1)
Jordan00191	Jordan_00191 poly(R)-hydroxyalkanoic acid synthase, class III, PhaC subunit
Jordan00206	Jordan_00206 Subversion of eukaryotic vesicle trafficking A
Jordan00243	Jordan_00243 hypothetical protein
Jordan00905	Jordan_00920 hypothetical protein
Jordan01053	Jordan_01068 hypothetical protein
Jordan01136	Jordan_01152 hypothetical protein
Jordan01908	Jordan_01930 Chondramide synthase cmdD

Jordan01994	Jordan_02016 hypothetical protein
Jordan02162	Jordan_02185 mRNA interferase HigB
brunen00245	PROKKA_00248 Toxin HigB-2
brunen00658	PROKKA_00663 hypothetical protein
brunen01042	PROKKA_01048 hypothetical protein
brunen01144	PROKKA_01151 hypothetical protein
brunen02429	PROKKA_02462 putative protein involved in outer membrane biogenesis
brunen03103	PROKKA_03139 Helix-turn-helix
erythra00448	PROKKA_00451 hypothetical protein
erythra00672	PROKKA_00677 hypothetical protein
erythra00955	PROKKA_00963 Phosphocholine transferase AnkX
erythra01315	PROKKA_01325 hypothetical protein
erythra02520	PROKKA_02535 hypothetical protein
erythra02844	PROKKA_02872 hypothetical protein
erythra02946	PROKKA_02976 hypothetical protein
mac00069	mac_D5800_00069 hypothetical protein
mac00176	mac_D5800_00185 hypothetical protein
mac00330	mac_D5800_00339 Putative beta-lactamase HcpC precursor
mac00407	mac_D5800_00419 hypothetical protein
mac00445	mac_D5800_00457 hypothetical protein
mac00475	mac_D5800_00488 Multidrug resistance outer membrane protein MdtP precursor
mac00545	mac_D5800_00559 hypothetical protein
mac00566	mac_D5800_00580 Acyltransferase family protein
mac00574	mac_D5800_00588 putative glycosyl transferase
mac00575	mac_D5800_00589 hypothetical protein
mac00576	mac_D5800_00590 Chondroitin polymerase
mac00648	mac_D5800_00663 NAD(P)H azoreductase
mac00651	mac_D5800_00666 hypothetical protein
mac00670	mac_D5800_00685 hypothetical protein
mac00678	mac_D5800_00693 Carbamoyl-phosphate synthase small chain

mac00679	mac_D5800_00694 Carbamoyl-phosphate synthase large chain
mac00731	mac_D5800_00746 hypothetical protein
mac00754	mac_D5800_00769 hypothetical protein
mac00767	mac_D5800_00782 N-formylglutamate deformylase
mac00890	mac_D5800_00906 Tetratricopeptide repeat
mac00908	mac_D5800_00924 polysaccharide deacetylase family sporulation protein PdaB
mac00914	mac_D5800_00930 hypothetical protein
mac00955	mac_D5800_00972 hypothetical protein
mac00977	mac_D5800_00994 hypothetical protein
mac01041	mac_D5800_01059 hypothetical protein
mac01078	mac_D5800_01098 hypothetical protein
mac01079	mac_D5800_01099 Hyaluronan synthase
mac01081	mac_D5800_01101 Alginate biosynthesis protein AlgA
mac01097	mac_D5800_01117 hypothetical protein
mac01098	mac_D5800_01118 hypothetical protein
mac01107	mac_D5800_01127 dTDP-glucose 4,6-dehydratase
mac01109	mac_D5800_01129 UDP-4-amino-4-deoxy-L-arabinose--oxoglutarate aminotransferase
mac01111	mac_D5800_01131 glucans biosynthesis protein
mac01117	mac_D5800_01137 Sensor kinase protein RcsC
mac01179	mac_D5800_01199 Na(+)/serine-threonine symporter
mac01245	mac_D5800_01266 hypothetical protein
mac01259	mac_D5800_01280 hypothetical protein
mac01261	mac_D5800_01282 putative protein tyrosine phosphatase
mac01265	mac_D5800_01286 hypothetical protein
mac01314	mac_D5800_01335 hypothetical protein
mac01411	mac_D5800_01433 hypothetical protein
mac01486	mac_D5800_01508 hypothetical protein
mac01565	mac_D5800_01587 hypothetical protein
mac01567	mac_D5800_01589 hypothetical protein
mac01622	mac_D5800_01645 hypothetical protein

mac01980	mac_D5800_02007 integral membrane protein, YccS/YhfK family
mac02005	mac_D5800_02032 putative amino-acid-binding protein YxeM precursor
mac02026	mac_D5800_02053 Ribulose bisphosphate carboxylase large chain
mac02028	mac_D5800_02055 hypothetical protein
mac02029	mac_D5800_02056 hypothetical protein
mac02068	mac_D5800_02095 hypothetical protein
mac02090	mac_D5800_02117 hypothetical protein
mac02098	mac_D5800_02125 Small integral membrane protein
mac02100	mac_D5800_02127 hypothetical protein
mac02162	mac_D5800_02189 hypothetical protein
mac02219	mac_D5800_02249 RasGEF domain protein
mac02238	mac_D5800_02268 Putative universal stress protein
mac02339	mac_D5800_02371 hypothetical protein
mac02357	mac_D5800_02389 hypothetical protein
mac02361	mac_D5800_02393 Creatinine amidohydrolase
mac02385	mac_D5800_02418 small GTP-binding protein domain protein
mac02425	mac_D5800_02459 Putative cyclase
mac02427	mac_D5800_02461 Oxalate decarboxylase OxdC
mac02475	mac_D5800_02509 hypothetical protein
mac02634	mac_D5800_02668 hypothetical protein
mac02645	mac_D5800_02679 hypothetical protein
mac02646	mac_D5800_02680 hypothetical protein
mac02663	mac_D5800_02697 hypothetical protein
mac02686	mac_D5800_02720 Endonuclease V
mac02802	mac_D5800_02837 hypothetical protein
mac02880	mac_D5800_02915 TQO small subunit DoxD
mac02885	mac_D5800_02920 Inner membrane protein YejM
mac03009	mac_D5800_03044 hypothetical protein
mac03135	mac_D5800_03171 hypothetical protein
mac03299	mac_D5800_03336 ankyrin repeat protein

micdadei00829	micdadei_00846 hypothetical protein
micdadei01794	micdadei_01822 Transcriptional regulatory protein AfsQ1
Birmingham00001	PROKKA_00001 hypothetical protein
Birmingham00009	PROKKA_00009 hypothetical protein
Birmingham00011	PROKKA_00011 hypothetical protein
Birmingham00046	PROKKA_00046 HEXXH motif domain protein
Birmingham00072	PROKKA_00072 hypothetical protein
Birmingham00127	PROKKA_00127 DNA alkylation repair enzyme
Birmingham00229	PROKKA_00230 Acyltransferase family protein
Birmingham00324	PROKKA_00328 Proline iminopeptidase
Birmingham00350	PROKKA_00354 Ras family protein
Birmingham00373	PROKKA_00377 hypothetical protein
Birmingham00376	PROKKA_00380 hypothetical protein
Birmingham00479	PROKKA_00484 hypothetical protein
Birmingham00655	PROKKA_00670 Inner membrane protein YqaA
Birmingham00669	PROKKA_00684 Acyltransferase family protein
Birmingham00854	PROKKA_00870 hypothetical protein
Birmingham00913	PROKKA_00929 hypothetical protein
Birmingham00917	PROKKA_00933 hypothetical protein
Birmingham00927	PROKKA_00943 hypothetical protein
Birmingham00948	PROKKA_00964 hypothetical protein
Birmingham00976	PROKKA_00993 3-hydroxybenzoate 6-hydroxylase
Birmingham01082	PROKKA_01101 Bacterial protein of unknown function (Gcw_chp)
Birmingham01276	PROKKA_01296 putative HD phosphohydrolase
Birmingham01301	PROKKA_01322 hypothetical protein
Birmingham01321	PROKKA_01342 hypothetical protein
Birmingham01425	PROKKA_01447 Phosphatidylcholine-sterol acyltransferase precursor
Birmingham01478	PROKKA_01500 hypothetical protein
Birmingham01494	PROKKA_01516 hypothetical protein
Birmingham01506	PROKKA_01528 Inner membrane protein YbbJ

Birmingham01620	PROKKA_01642 putative diguanylate cyclase YegE
Birmingham01626	PROKKA_01648 chromosome segregation protein SMC
Birmingham01673	PROKKA_01695 FAD dependent oxidoreductase
Birmingham01684	PROKKA_01706 Uridine phosphorylase
Birmingham01712	PROKKA_01734 lipopolysaccharide 1,2-N-acetylglucosaminetransferase
Birmingham01714	PROKKA_01736 hypothetical protein
Birmingham01716	PROKKA_01738 hypothetical protein
Birmingham01721	PROKKA_01743 hypothetical protein
Birmingham01948	PROKKA_01974 hypothetical protein
Birmingham01949	PROKKA_01975 hypothetical protein
Birmingham02102	PROKKA_02131 Cytochrome P450
Birmingham02163	PROKKA_02192 Tryptophan--tRNA ligase 2
Birmingham02175	PROKKA_02204 Glutamyl-tRNA reductase
Birmingham02194	PROKKA_02223 hypothetical protein
Birmingham02201	PROKKA_02230 Alpha-hemolysin translocation ATP-binding protein HlyB
Birmingham02284	PROKKA_02313 Alpha-amylase precursor
Birmingham02287	PROKKA_02316 hypothetical protein
Birmingham02298	PROKKA_02327 hypothetical protein
Birmingham02380	PROKKA_02409 putative membrane protein YjcC
Birmingham02475	PROKKA_02506 ribosomal-protein-alanine N-acetyltransferase
Birmingham02551	PROKKA_02582 hypothetical protein
Birmingham02639	PROKKA_02670 hypothetical protein
Birmingham02685	PROKKA_02716 hypothetical protein
Birmingham02686	PROKKA_02717 hypothetical protein
Birmingham02689	PROKKA_02720 hypothetical protein
Birmingham02721	PROKKA_02752 cation diffusion facilitator family transporter
Birmingham02745	PROKKA_02777 hypothetical protein
Birmingham02784	PROKKA_02816 Blue-light-activated protein
Birmingham02793	PROKKA_02825 Uridine kinase
Birmingham02885	PROKKA_02918 hypothetical protein

Birmingham02960	PROKKA_02993 transcriptional regulator, y4mF family
Birmingham02961	PROKKA_02994 hypothetical protein
Birmingham02967	PROKKA_03000 hypothetical protein
Birmingham03174	PROKKA_03209 Arginine--tRNA ligase
Birmingham03184	PROKKA_03219 Cyclic di-GMP phosphodiesterase response regulator RpfG
Birmingham03210	PROKKA_03245 hypothetical protein
Birmingham03218	PROKKA_03253 hypothetical protein
Birmingham03243	PROKKA_03279 Superfamily II helicase
Birmingham03305	PROKKA_03342 hypothetical protein
Birmingham03317	PROKKA_03358 Transposase
Jamestown00059	PROKKA_00059 hypothetical protein
Jamestown00131	PROKKA_00131 prevent-host-death family protein
Jamestown00182	PROKKA_00182 hypothetical protein
Jamestown00184	PROKKA_00184 Putative multidrug export ATP-binding/permease protein
Jamestown00197	PROKKA_00197 Aerobic respiration control sensor protein ArcB
Jamestown00253	PROKKA_00255 hypothetical protein
Jamestown00270	PROKKA_00272 Phage-related protein
Jamestown00279	PROKKA_00281 hypothetical protein
Jamestown00293	PROKKA_00295 2-methylcitrate dehydratase
Jamestown00296	PROKKA_00298 hypothetical protein
Jamestown00332	PROKKA_00334 hypothetical protein
Jamestown00354	PROKKA_00357 HTH-type transcriptional repressor YcgE
Jamestown00408	PROKKA_00411 hypothetical protein
Jamestown00520	PROKKA_00523 hypothetical protein
Jamestown00523	PROKKA_00526 pheromone autoinducer 2 transporter
Jamestown00551	PROKKA_00554 hypothetical protein
Jamestown00705	PROKKA_00710 K(+)/H(+) antiporter
Jamestown00740	PROKKA_00745 hypothetical protein
Jamestown00826	PROKKA_00832 membrane protein insertase
Jamestown00919	PROKKA_00926 ankyrin repeat protein

Jamestown00925	PROKKA_00932 putative MFS-type transporter YcaD
Jamestown01042	PROKKA_01049 Avirulence protein AvrBs3
Jamestown01043	PROKKA_01050 hypothetical protein
Jamestown01057	PROKKA_01065 hypothetical protein
Jamestown01083	PROKKA_01091 Alpha-aminoadipate--LysW ligase LysX
Jamestown01135	PROKKA_01143 HAD superfamily (subfamily IG) hydrolase, 5'-nucleotidase
Jamestown01143	PROKKA_01151 hypothetical protein
Jamestown01148	PROKKA_01156 Soluble aldose sugar dehydrogenase YliI precursor
Jamestown01180	PROKKA_01193 Endonuclease/Exonuclease/phosphatase family protein
Jamestown01242	PROKKA_01255 hypothetical protein
Jamestown01257	PROKKA_01270 Chloramphenicol resistance pump Cmr
Jamestown01268	PROKKA_01281 hypothetical protein
Jamestown01348	PROKKA_01361 PIN domain protein
Jamestown01358	PROKKA_01371 phosphosulfolactate synthase
Jamestown01471	PROKKA_01496 Molybdopterin synthase catalytic subunit
Jamestown01474	PROKKA_01499 Cyclic pyranopterin monophosphate synthase
Jamestown01480	PROKKA_01505 Fumarate hydratase class I, anaerobic
Jamestown01511	PROKKA_01536 hypothetical protein
Jamestown01519	PROKKA_01544 hypothetical protein
Jamestown01523	PROKKA_01548 hypothetical protein
Jamestown01677	PROKKA_01702 hypothetical protein
Jamestown01776	PROKKA_01802 hypothetical protein
Jamestown01867	PROKKA_01894 hypothetical protein
Jamestown01925	PROKKA_01952 hypothetical protein
Jamestown02173	PROKKA_02202 hypothetical protein
Jamestown02187	PROKKA_02217 hypothetical protein
Jamestown02195	PROKKA_02225 hypothetical protein
Jamestown02222	PROKKA_02252 hypothetical protein
Jamestown02292	PROKKA_02323 hypothetical protein
Jamestown02317	PROKKA_02348 TDP-fucosamine acetyltransferase

Jamestown02366	PROKKA_02398 hypothetical protein
Jamestown02373	PROKKA_02405 putative O-linked N-acetylglucosamine transferase, SPINDLY family
Jamestown02433	PROKKA_02466 hypothetical protein
Jamestown02476	PROKKA_02509 hypothetical protein
Jamestown02501	PROKKA_02534 hypothetical protein
Jamestown02567	PROKKA_02600 hypothetical protein
Jamestown02630	PROKKA_02663 hypothetical protein
Jamestown02633	PROKKA_02666 hypothetical protein
Jamestown02635	PROKKA_02668 O-acetylserine/cysteine export protein
Jamestown02666	PROKKA_02699 hypothetical protein
Jamestown02850	PROKKA_02884 Putative amidoligase enzyme
Jamestown02851	PROKKA_02885 Putative glutamine amidotransferasec
Jamestown02894	PROKKA_02928 hypothetical protein
Jamestown02929	PROKKA_02963 hypothetical protein
Jamestown03037	PROKKA_03072 hypothetical protein
Jamestown03038	PROKKA_03073 hypothetical protein
Jamestown03074	PROKKA_03109 hypothetical protein
Jamestown03110	PROKKA_03145 hypothetical protein
Jamestown03132	PROKKA_03167 hypothetical protein
Jamestown03135	PROKKA_03170 DNA primase TraC
Jamestown03165	PROKKA_03200 hypothetical protein
Jamestown03180	PROKKA_03215 Ran GTPase-activating protein (RanGAP) involved in mRNA processing and transport
Jamestown03248	PROKKA_03283 hypothetical protein
Jamestown03249	PROKKA_03284 hypothetical protein
Jamestown03287	PROKKA_03323 Chain length determinant protein
Jordan00597	Jordan_00609 hypothetical protein
Jordan00626	Jordan_00638 hypothetical protein
Jordan00770	Jordan_00783 hypothetical protein
Jordan00874	Jordan_00889 hypothetical protein
Jordan00950	Jordan_00965 hypothetical protein

Jordan01051	Jordan_01066 hypothetical protein
Jordan01099	Jordan_01115 periplasmic protein
Jordan01179	Jordan_01196 hypothetical protein
Jordan01337	Jordan_01355 hypothetical protein
Jordan01350	Jordan_01368 Glycosyl hydrolase family 81
Jordan01378	Jordan_01396 Tetratricopeptide repeat
Jordan01408	Jordan_01426 hypothetical protein
Jordan01666	Jordan_01687 hypothetical protein
Jordan01680	Jordan_01701 Signal peptidase I
Jordan01685	Jordan_01706 hypothetical protein
Jordan02189	Jordan_02212 hypothetical protein
Jordan02190	Jordan_02213 hypothetical protein
Jordan02224	Jordan_02248 Alpha-acetolactate decarboxylase precursor
Jordan02308	Jordan_02332 hypothetical protein
Jordan02351	Jordan_02375 hypothetical protein
Jordan02609	Jordan_02643 hypothetical protein
Jordan02610	Jordan_02644 hypothetical protein
Jordan02639	Jordan_02673 3-dehydroquinate synthase
Jordan02879	Jordan_02916 putative lyase
brunen00075	PROKKA_00076 preprotein translocase subunit SecA
brunen00221	PROKKA_00224 hypothetical protein
brunen00398	PROKKA_00401 Hydantoin racemase
brunen00671	PROKKA_00676 hypothetical protein
brunen00798	PROKKA_00803 putative lactoylglutathione lyase
brunen01107	PROKKA_01114 hypothetical protein
brunen01200	PROKKA_01211 hypothetical protein
brunen01308	PROKKA_01320 hypothetical protein
brunen01565	PROKKA_01579 hypothetical protein
brunen01711	PROKKA_01726 hypothetical protein
brunen01714	PROKKA_01729 hypothetical protein

brunen01801	PROKKA_01825 hypothetical protein
brunen01925	PROKKA_01953 hypothetical protein
brunen01956	PROKKA_01984 Lipase (class 3)
brunen01990	PROKKA_02018 hypothetical protein
brunen02043	PROKKA_02072 hypothetical protein
brunen02135	PROKKA_02165 hypothetical protein
brunen02136	PROKKA_02166 hypothetical protein
brunen02137	PROKKA_02167 hypothetical protein
brunen02194	PROKKA_02225 hypothetical protein
brunen02214	PROKKA_02245 hypothetical protein
brunen02227	PROKKA_02258 acetoin dehydrogenase E2 subunit dihydrolipoyllysine-residue acetyltransferase
brunen02562	PROKKA_02595 hypothetical protein
brunen02677	PROKKA_02711 putative protein tyrosine phosphatase
brunen02678	PROKKA_02712 hypothetical protein
brunen02730	PROKKA_02764 hypothetical protein
brunen02780	PROKKA_02814 ankyrin repeat protein
brunen02846	PROKKA_02880 hypothetical protein
brunen02896	PROKKA_02930 Enamine/imine deaminase
brunen03167	PROKKA_03203 hypothetical protein
brunen03260	PROKKA_03297 hypothetical protein
brunen03277	PROKKA_03314 hypothetical protein
erythra00121	PROKKA_00124 hypothetical protein
erythra00129	PROKKA_00132 hypothetical protein
erythra00397	PROKKA_00400 Cyclic di-GMP phosphodiesterase response regulator RpfG
erythra00937	PROKKA_00945 Magnesium-chelatase 60 kDa subunit
erythra01060	PROKKA_01068 hypothetical protein
erythra01337	PROKKA_01347 L-2-hydroxyglutarate oxidase LhgO
erythra01349	PROKKA_01359 hypothetical protein
erythra01805	PROKKA_01818 hypothetical protein
erythra01962	PROKKA_01976 Sulfate adenylyltransferase subunit 2

erythra01977	PROKKA_01991 hypothetical protein
erythra02773	PROKKA_02792 mevalonate kinase
erythra02948	PROKKA_02978 hypothetical protein
erythra03022	PROKKA_03056 hypothetical protein
erythra03058	PROKKA_03092 fumarylacetoacetase
mac00024	mac_D5800_00024 hypothetical protein
mac00028	mac_D5800_00028 hypothetical protein
mac00037	mac_D5800_00037 hypothetical protein
mac00151	mac_D5800_00160 hypothetical protein
mac00213	mac_D5800_00222 hypothetical protein
mac00306	mac_D5800_00315 hypothetical protein
mac00375	mac_D5800_00387 hypothetical protein
mac00437	mac_D5800_00449 hypothetical protein
mac00457	mac_D5800_00470 hypothetical protein
mac00502	mac_D5800_00515 hypothetical protein
mac00557	mac_D5800_00571 hypothetical protein
mac00638	mac_D5800_00653 hypothetical protein
mac00667	mac_D5800_00682 hypothetical protein
mac00691	mac_D5800_00706 hypothetical protein
mac00698	mac_D5800_00713 hypothetical protein
mac00712	mac_D5800_00727 hypothetical protein
mac00717	mac_D5800_00732 Transcriptional regulatory protein BasR
mac00740	mac_D5800_00755 ankyrin repeat protein
mac00811	mac_D5800_00826 hypothetical protein
mac00824	mac_D5800_00839 hypothetical protein
mac00846	mac_D5800_00861 Sulfate starvation-induced protein 7
mac00862	mac_D5800_00877 Metallo-beta-lactamase superfamily protein
mac00891	mac_D5800_00907 Avirulence protein AvrBs3
mac01089	mac_D5800_01109 Galactokinase
mac01101	mac_D5800_01121 hypothetical protein

mac01131	mac_D5800_01151 serine/threonine protein kinase
mac01183	mac_D5800_01203 Glycine betaine/carnitine/choline transport system permease protein OpuCB
mac01208	mac_D5800_01228 hypothetical protein
mac01251	mac_D5800_01272 serine protease inhibitor-like protein
mac01252	mac_D5800_01273 hypothetical protein
mac01263	mac_D5800_01284 hypothetical protein
mac01267	mac_D5800_01288 hypothetical protein
mac01450	mac_D5800_01472 hypothetical protein
mac01453	mac_D5800_01475 hypothetical protein
mac01488	mac_D5800_01510 Putative zinc metalloproteasec/MT2700
mac01490	mac_D5800_01512 hypothetical protein
mac01491	mac_D5800_01513 hypothetical protein
mac01540	mac_D5800_01562 hypothetical protein
mac01552	mac_D5800_01574 hypothetical protein
mac01553	mac_D5800_01575 hypothetical protein
mac01765	mac_D5800_01788 hypothetical protein
mac01838	mac_D5800_01864 putative isomerase YddE
mac01912	mac_D5800_01939 Sugar-specific transcriptional regulator TrmB
mac01913	mac_D5800_01940 Orotate phosphoribosyltransferase
mac01914	mac_D5800_01941 Sulfite exporter TauE/SafE
mac01921	mac_D5800_01948 Aspartyl/Asparaginyl beta-hydroxylase
mac01933	mac_D5800_01960 hypothetical protein
mac01963	mac_D5800_01990 L-asparaginase
mac01965	mac_D5800_01992 Proline iminopeptidase
mac01971	mac_D5800_01998 Aerobic respiration control sensor protein ArcB
mac01972	mac_D5800_01999 nuclease NucT
mac01974	mac_D5800_02001 hypothetical protein
mac01986	mac_D5800_02013 3-oxoacyl-[acyl-carrier-protein] synthase 2
mac01987	mac_D5800_02014 hypothetical protein
mac02001	mac_D5800_02028 PQQ-dependent catabolism-associated beta-propeller protein

mac02055	mac_D5800_02082 Toxin YoeB
mac02056	mac_D5800_02083 Antitoxin YefM
mac02108	mac_D5800_02135 hypothetical protein
mac02194	mac_D5800_02224 Clavaminate synthase 2
mac02195	mac_D5800_02225 putative amino-acid metabolite efflux pump
mac02200	mac_D5800_02230 Karyopherin (importin) beta
mac02205	mac_D5800_02235 hypothetical protein
mac02243	mac_D5800_02274 hypothetical protein
mac02309	mac_D5800_02340 glutamine ABC transporter periplasmic protein
mac02365	mac_D5800_02397 hypothetical protein
mac02413	mac_D5800_02446 hypothetical protein
mac02414	mac_D5800_02447 hypothetical protein
mac02438	mac_D5800_02472 hypothetical protein
mac02441	mac_D5800_02475 hypothetical protein
mac02465	mac_D5800_02499 hypothetical protein
mac02563	mac_D5800_02597 Polysaccharide biosynthesis protein
mac02619	mac_D5800_02653 hypothetical protein
mac02635	mac_D5800_02669 hypothetical protein
mac02706	mac_D5800_02740 hypothetical protein
mac02713	mac_D5800_02747 hypothetical protein
mac02757	mac_D5800_02792 Gramicidin S synthase II
mac02764	mac_D5800_02799 hypothetical protein
mac02831	mac_D5800_02866 recombination regulator RecX
mac02834	mac_D5800_02869 hypothetical protein
mac02879	mac_D5800_02914 hypothetical protein
mac02883	mac_D5800_02918 hypothetical protein
mac02890	mac_D5800_02925 hypothetical protein
mac02913	mac_D5800_02948 hypothetical protein
mac02973	mac_D5800_03008 hypothetical protein
mac03042	mac_D5800_03077 hypothetical protein

mac03043	mac_D5800_03078 hypothetical protein
mac03068	mac_D5800_03103 hypothetical protein
mac03070	mac_D5800_03105 hypothetical protein
mac03071	mac_D5800_03106 hypothetical protein
mac03147	mac_D5800_03183 3,4-dihydroxy-2-butanone 4-phosphate synthase
mac03168	mac_D5800_03204 hypothetical protein
mac03319	mac_D5800_03356 hypothetical protein
micdadei00169	micdadei_00169 ankyrin repeat protein
micdadei00462	micdadei_00469 hypothetical protein
micdadei00478	micdadei_00485 hypothetical protein
micdadei00614	micdadei_00622 hypothetical protein
micdadei00890	micdadei_00908 putative transcriptional regulatory protein pdtaR
micdadei00926	micdadei_00944 hypothetical protein
micdadei01616	micdadei_01641 hypothetical protein
micdadei01812	micdadei_01840 polysaccharide deacetylase family sporulation protein PdaB
micdadei02170	micdadei_02204 hypothetical protein
micdadei02172	micdadei_02206 hypothetical protein
micdadei02270	micdadei_02304 hypothetical protein
micdadei02809	micdadei_02848 3-oxoacyl-[acyl-carrier-protein] reductase FabG
nautram00156	PROKKA_00159 hypothetical protein
nautram00170	PROKKA_00173 hypothetical protein
nautram00177	PROKKA_00180 hypothetical protein
nautram00190	PROKKA_00193 hypothetical protein
nautram00227	PROKKA_00230 Ankyrin repeats (3 copies)
nautram00268	PROKKA_00271 Ankyrin repeats (3 copies)
nautram00859	PROKKA_00868 hypothetical protein
nautram01180	PROKKA_01193 glycogen synthase, Corynebacterium family
nautram01367	PROKKA_01390 Tyrocidine synthase I
nautram01988	PROKKA_02018 Methyltransferase domain protein
nautram02213	PROKKA_02243 hypothetical protein

nautram02249	PROKKA_02279 hypothetical protein
nautram02725	PROKKA_02757 Carbon storage regulator
nautram02906	PROKKA_02940 hypothetical protein
nautram02937	PROKKA_02971 Sporulation initiation inhibitor protein soj
nautram02947	PROKKA_02981 hypothetical protein
Birmingham00002	PROKKA_00002 hypothetical protein
Birmingham00003	PROKKA_00003 Ankyrin repeats (3 copies)
Birmingham00014	PROKKA_00014 hypothetical protein
Birmingham00034	PROKKA_00034 hypothetical protein
Birmingham00045	PROKKA_00045 hypothetical protein
Birmingham00048	PROKKA_00048 hypothetical protein
Birmingham00052	PROKKA_00052 High temperature protein G
Birmingham00094	PROKKA_00094 hypothetical protein
Birmingham00105	PROKKA_00105 2-dehydro-3-deoxyphosphooctonate aldolase
Birmingham00111	PROKKA_00111 hypothetical protein
Birmingham00138	PROKKA_00138 hypothetical protein
Birmingham00140	PROKKA_00140 hypothetical protein
Birmingham00156	PROKKA_00157 hypothetical protein
Birmingham00158	PROKKA_00159 hypothetical protein
Birmingham00180	PROKKA_00181 Succinate semialdehyde dehydrogenase [NAD(P)+] Sad
Birmingham00221	PROKKA_00222 GTP-binding protein Era
Birmingham00224	PROKKA_00225 Bacteriophytochrome cph2
Birmingham00232	PROKKA_00233 hypothetical protein
Birmingham00246	PROKKA_00247 hypothetical protein
Birmingham00252	PROKKA_00253 3-oxoadipate enol-lactonase 2
Birmingham00266	PROKKA_00267 hypothetical protein
Birmingham00293	PROKKA_00297 hypothetical protein
Birmingham00294	PROKKA_00298 murein hydrolase B
Birmingham00299	PROKKA_00303 hypothetical protein
Birmingham00300	PROKKA_00304 hypothetical protein

Birmingham00309	PROKKA_00313 hypothetical protein
Birmingham00310	PROKKA_00314 hypothetical protein
Birmingham00323	PROKKA_00327 hypothetical protein
Birmingham00327	PROKKA_00331 preprotein translocase subunit SecA
Birmingham00328	PROKKA_00332 hypothetical protein
Birmingham00333	PROKKA_00337 hypothetical protein
Birmingham00342	PROKKA_00346 hypothetical protein
Birmingham00346	PROKKA_00350 hypothetical protein
Birmingham00347	PROKKA_00351 hypothetical protein
Birmingham00349	PROKKA_00353 Curved DNA-binding protein
Birmingham00354	PROKKA_00358 hypothetical protein
Birmingham00358	PROKKA_00362 hypothetical protein
Birmingham00360	PROKKA_00364 hypothetical protein
Birmingham00361	PROKKA_00365 hypothetical protein
Birmingham00365	PROKKA_00369 hypothetical protein
Birmingham00369	PROKKA_00373 Chaperone protein DnaJ
Birmingham00371	PROKKA_00375 hypothetical protein
Birmingham00372	PROKKA_00376 hypothetical protein
Birmingham00379	PROKKA_00383 hypothetical protein
Birmingham00384	PROKKA_00388 hypothetical protein
Birmingham00385	PROKKA_00389 hypothetical protein
Birmingham00393	PROKKA_00397 hypothetical protein
Birmingham00394	PROKKA_00398 hypothetical protein
Birmingham00427	PROKKA_00431 hypothetical protein
Birmingham00439	PROKKA_00444 Cell-division control histidine kinase PdhS
Birmingham00450	PROKKA_00455 hypothetical protein
Birmingham00455	PROKKA_00460 hypothetical protein
Birmingham00462	PROKKA_00467 hypothetical protein
Birmingham00464	PROKKA_00469 hypothetical protein
Birmingham00465	PROKKA_00470 (R)-specific enoyl-CoA hydratase

Birmingham00469	PROKKA_00474 hypothetical protein
Birmingham00480	PROKKA_00485 hypothetical protein
Birmingham00495	PROKKA_00500 hypothetical protein
Birmingham00511	PROKKA_00516 hypothetical protein
Birmingham00527	PROKKA_00535 hypothetical protein
Birmingham00541	PROKKA_00549 glutamate--cysteine ligase
Birmingham00558	PROKKA_00566 hypothetical protein
Birmingham00559	PROKKA_00567 hypothetical protein
Birmingham00564	PROKKA_00572 hypothetical protein
Birmingham00569	PROKKA_00577 hypothetical protein
Birmingham00570	PROKKA_00578 hypothetical protein
Birmingham00579	PROKKA_00587 Chaperone protein ClpB
Birmingham00580	PROKKA_00588 Heat shock protein F84.1
Birmingham00581	PROKKA_00589 hypothetical protein
Birmingham00595	PROKKA_00607 Lon protease
Birmingham00608	PROKKA_00622 hydrolase CocE/NonD family protein
Birmingham00609	PROKKA_00623 Cocaine esterase
Birmingham00610	PROKKA_00624 hypothetical protein
Birmingham00619	PROKKA_00633 hypothetical protein
Birmingham00637	PROKKA_00651 Chloramphenicol resistance pump Cmr
Birmingham00638	PROKKA_00652 Chorismate synthase
Birmingham00639	PROKKA_00653 3-deoxy-D-manno-octulosonate 8-phosphate phosphatase KdsC
Birmingham00666	PROKKA_00681 hypothetical protein
Birmingham00683	PROKKA_00698 Phosphoserine aminotransferase
Birmingham00689	PROKKA_00704 tetratricopeptide repeat protein
Birmingham00723	PROKKA_00738 Multicopper oxidase
Birmingham00731	PROKKA_00746 ATP-dependent zinc metalloprotease FtsH 2
Birmingham00732	PROKKA_00747 hypothetical protein
Birmingham00733	PROKKA_00748 hypothetical protein
Birmingham00736	PROKKA_00751 hypothetical protein

Birmingham00738	PROKKA_00753 hypothetical protein
Birmingham00739	PROKKA_00754 hypothetical protein
Birmingham00748	PROKKA_00764 putative diguanylate cyclase YedQ
Birmingham00787	PROKKA_00803 hypothetical protein
Birmingham00792	PROKKA_00808 hypothetical protein
Birmingham00794	PROKKA_00810 ubiquinone biosynthesis hydroxylase family protein
Birmingham00802	PROKKA_00818 hypothetical protein
Birmingham00840	PROKKA_00856 hypothetical protein
Birmingham00851	PROKKA_00867 hypothetical protein
Birmingham00852	PROKKA_00868 hypothetical protein
Birmingham00853	PROKKA_00869 hypothetical protein
Birmingham00863	PROKKA_00879 hypothetical protein
Birmingham00865	PROKKA_00881 putative outer membrane protein
Birmingham00866	PROKKA_00882 hypothetical protein
Birmingham00867	PROKKA_00883 hypothetical protein
Birmingham00892	PROKKA_00908 hypothetical protein
Birmingham00908	PROKKA_00924 hypothetical protein
Birmingham00929	PROKKA_00945 hypothetical protein
Birmingham00949	PROKKA_00965 hypothetical protein
Birmingham00955	PROKKA_00972 hypothetical protein
Birmingham00961	PROKKA_00978 hypothetical protein
Birmingham00967	PROKKA_00984 hypothetical protein
Birmingham00975	PROKKA_00992 hypothetical protein
Birmingham00991	PROKKA_01008 hypothetical protein
Birmingham01008	PROKKA_01025 hypothetical protein
Birmingham01041	PROKKA_01060 hypothetical protein
Birmingham01049	PROKKA_01068 Ferric iron reductase FhuF-like transporter
Birmingham01053	PROKKA_01072 hypothetical protein
Birmingham01063	PROKKA_01082 Thioredoxin reductase
Birmingham01072	PROKKA_01091 hypothetical protein

Birmingham01073	PROKKA_01092 hypothetical protein
Birmingham01081	PROKKA_01100 Sensor protein FixL
Birmingham01083	PROKKA_01102 Sporulation initiation phosphotransferase F
Birmingham01084	PROKKA_01103 Bacteriophytochrome cph2
Birmingham01095	PROKKA_01114 hypothetical protein
Birmingham01116	PROKKA_01135 pyridoxal phosphate enzyme, YggS family
Birmingham01121	PROKKA_01140 hypothetical protein
Birmingham01129	PROKKA_01148 hypothetical protein
Birmingham01144	PROKKA_01163 hypothetical protein
Birmingham01199	PROKKA_01219 hypothetical protein
Birmingham01221	PROKKA_01241 outer membrane biogenesis protein BamB
Birmingham01240	PROKKA_01260 small GTP-binding protein domain protein
Birmingham01245	PROKKA_01265 N-succinylglutamate 5-semialdehyde dehydrogenase
Birmingham01274	PROKKA_01294 hypothetical protein
Birmingham01293	PROKKA_01313 hypothetical protein
Birmingham01298	PROKKA_01318 hypothetical protein
Birmingham01299	PROKKA_01320 hypothetical protein
Birmingham01314	PROKKA_01335 hypothetical protein
Birmingham01361	PROKKA_01383 hypothetical protein
Birmingham01363	PROKKA_01385 hypothetical protein
Birmingham01374	PROKKA_01396 chaperone protein DnaJ
Birmingham01387	PROKKA_01409 Spermidine/putrescine-binding periplasmic protein precursor
Birmingham01391	PROKKA_01413 hypothetical protein
Birmingham01400	PROKKA_01422 hypothetical protein
Birmingham01416	PROKKA_01438 hypothetical protein
Birmingham01422	PROKKA_01444 Multidrug resistance ABC transporter ATP-binding/permease protein BmrA
Birmingham01423	PROKKA_01445 ankyrin repeat protein
Birmingham01424	PROKKA_01446 Proline iminopeptidase
Birmingham01468	PROKKA_01490 hypothetical protein
Birmingham01473	PROKKA_01495 hypothetical protein

Birmingham01477	PROKKA_01499 hypothetical protein
Birmingham01483	PROKKA_01505 Heterocyst differentiation ATP-binding protein HepA
Birmingham01484	PROKKA_01506 putative ABC transporter ATP-binding protein
Birmingham01487	PROKKA_01509 hypothetical protein
Birmingham01507	PROKKA_01529 hypothetical protein
Birmingham01508	PROKKA_01530 hypothetical protein
Birmingham01522	PROKKA_01544 hypothetical protein
Birmingham01525	PROKKA_01547 hypothetical protein
Birmingham01528	PROKKA_01550 hypothetical protein
Birmingham01547	PROKKA_01569 hypothetical protein
Birmingham01559	PROKKA_01581 PAP2 superfamily protein
Birmingham01560	PROKKA_01582 Non-motile and phage-resistance protein
Birmingham01561	PROKKA_01583 hypothetical protein
Birmingham01564	PROKKA_01586 hypothetical protein
Birmingham01565	PROKKA_01587 hypothetical protein
Birmingham01566	PROKKA_01588 hypothetical protein
Birmingham01567	PROKKA_01589 hypothetical protein
Birmingham01608	PROKKA_01630 hypothetical protein
Birmingham01609	PROKKA_01631 hypothetical protein
Birmingham01623	PROKKA_01645 hypothetical protein
Birmingham01630	PROKKA_01652 hypothetical protein
Birmingham01652	PROKKA_01674 hypothetical protein
Birmingham01653	PROKKA_01675 hypothetical protein
Birmingham01654	PROKKA_01676 hypothetical protein
Birmingham01657	PROKKA_01679 Aerobic respiration control sensor protein ArcB
Birmingham01658	PROKKA_01680 Virulence sensor protein BvgS precursor
Birmingham01659	PROKKA_01681 hypothetical protein
Birmingham01660	PROKKA_01682 hypothetical protein
Birmingham01661	PROKKA_01683 hypothetical protein
Birmingham01662	PROKKA_01684 hypothetical protein

Birmingham01664	PROKKA_01686 hypothetical protein
Birmingham01672	PROKKA_01694 Aerobic respiration control sensor protein ArcB
Birmingham01683	PROKKA_01705 hypothetical protein
Birmingham01689	PROKKA_01711 Multidrug resistance ABC transporter ATP-binding and permease protein
Birmingham01711	PROKKA_01733 hypothetical protein
Birmingham01720	PROKKA_01742 hypothetical protein
Birmingham01722	PROKKA_01744 hypothetical protein
Birmingham01726	PROKKA_01748 hypothetical protein
Birmingham01734	PROKKA_01756 hypothetical protein
Birmingham01756	PROKKA_01778 Zinc carboxypeptidase
Birmingham01757	PROKKA_01779 hypothetical protein
Birmingham01765	PROKKA_01787 hypothetical protein
Birmingham01774	PROKKA_01796 hypothetical protein
Birmingham01792	PROKKA_01814 hypothetical protein
Birmingham01802	PROKKA_01825 hypothetical protein
Birmingham01822	PROKKA_01845 hypothetical protein
Birmingham01836	PROKKA_01859 hypothetical protein
Birmingham01841	PROKKA_01864 Retron-type reverse transcriptase
Birmingham01842	PROKKA_01865 Homeobox domain protein
Birmingham01851	PROKKA_01874 TDP-fucosamine acetyltransferase
Birmingham01873	PROKKA_01897 hypothetical protein
Birmingham01890	PROKKA_01914 hypothetical protein
Birmingham01892	PROKKA_01916 hypothetical protein
Birmingham01919	PROKKA_01944 hypothetical protein
Birmingham01926	PROKKA_01951 hypothetical protein
Birmingham01955	PROKKA_01981 Acriflavine resistance protein B
Birmingham01958	PROKKA_01984 hypothetical protein
Birmingham01966	PROKKA_01992 Polar-differentiation response regulator DivK
Birmingham01969	PROKKA_01995 hypothetical protein
Birmingham01978	PROKKA_02004 hypothetical protein

Birmingham02004	PROKKA_02033 hypothetical protein
Birmingham02023	PROKKA_02052 Methionine aminotransferase
Birmingham02027	PROKKA_02056 carboxylate/amino acid/amine transporter
Birmingham02031	PROKKA_02060 hypothetical protein
Birmingham02036	PROKKA_02065 hypothetical protein
Birmingham02049	PROKKA_02078 hypothetical protein
Birmingham02071	PROKKA_02100 Betaine aldehyde dehydrogenase
Birmingham02072	PROKKA_02101 Aldehyde dehydrogenase PuuC
Birmingham02073	PROKKA_02102 Riboflavin biosynthesis protein RibD
Birmingham02074	PROKKA_02103 Riboflavin synthase
Birmingham02079	PROKKA_02108 3-hydroxy-3-methylglutaryl-coenzyme A reductase
Birmingham02090	PROKKA_02119 hypothetical protein
Birmingham02096	PROKKA_02125 hypothetical protein
Birmingham02098	PROKKA_02127 hypothetical protein
Birmingham02099	PROKKA_02128 hypothetical protein
Birmingham02110	PROKKA_02139 hypothetical protein
Birmingham02144	PROKKA_02173 hypothetical protein
Birmingham02157	PROKKA_02186 Abortive infection bacteriophage resistance protein
Birmingham02160	PROKKA_02189 hypothetical protein
Birmingham02161	PROKKA_02190 hypothetical protein
Birmingham02167	PROKKA_02196 Heme oxygenase
Birmingham02169	PROKKA_02198 Glutamate-1-semialdehyde 2,1-aminomutase
Birmingham02171	PROKKA_02200 hypothetical protein
Birmingham02180	PROKKA_02209 hypothetical protein
Birmingham02182	PROKKA_02211 hypothetical protein
Birmingham02183	PROKKA_02212 hypothetical protein
Birmingham02184	PROKKA_02213 Putative beta-lactamase HcpC precursor
Birmingham02191	PROKKA_02220 hypothetical protein
Birmingham02200	PROKKA_02229 hypothetical protein
Birmingham02216	PROKKA_02245 Arabinose transporter

Birmingham02222	PROKKA_02251 hypothetical protein
Birmingham02223	PROKKA_02252 hypothetical protein
Birmingham02226	PROKKA_02255 putative symporter YodF
Birmingham02227	PROKKA_02256 hypothetical protein
Birmingham02233	PROKKA_02262 hypothetical protein
Birmingham02250	PROKKA_02279 hypothetical protein
Birmingham02255	PROKKA_02284 hypothetical protein
Birmingham02259	PROKKA_02288 Demethylspheroidene O-methyltransferase
Birmingham02266	PROKKA_02295 hypothetical protein
Birmingham02268	PROKKA_02297 hypothetical protein
Birmingham02275	PROKKA_02304 Alpha-1,4-glucan:maltose-1-phosphate maltosyltransferase 1
Birmingham02282	PROKKA_02311 Maltooligosyl trehalose synthase
Birmingham02283	PROKKA_02312 Maltooligosyl trehalose synthase
Birmingham02285	PROKKA_02314 hypothetical protein
Birmingham02291	PROKKA_02320 hypothetical protein
Birmingham02294	PROKKA_02323 hypothetical protein
Birmingham02296	PROKKA_02325 hypothetical protein
Birmingham02297	PROKKA_02326 hypothetical protein
Birmingham02299	PROKKA_02328 Alpha/beta hydrolase family protein
Birmingham02302	PROKKA_02331 hypothetical protein
Birmingham02325	PROKKA_02354 Aquaporin Z 2
Birmingham02330	PROKKA_02359 tRNA modification GTPase MnmE
Birmingham02336	PROKKA_02365 DNA replication and repair protein RecF
Birmingham02347	PROKKA_02376 hypothetical protein
Birmingham02348	PROKKA_02377 hypothetical protein
Birmingham02349	PROKKA_02378 hypothetical protein
Birmingham02350	PROKKA_02379 hypothetical protein
Birmingham02355	PROKKA_02384 hypothetical protein
Birmingham02356	PROKKA_02385 hypothetical protein
Birmingham02364	PROKKA_02393 Fluffing protein

Birmingham02367	PROKKA_02396 hypothetical protein
Birmingham02374	PROKKA_02403 hypothetical protein
Birmingham02383	PROKKA_02412 hypothetical protein
Birmingham02393	PROKKA_02422 methylmalonyl-CoA epimerase
Birmingham02396	PROKKA_02425 hypothetical protein
Birmingham02405	PROKKA_02434 hypothetical protein
Birmingham02409	PROKKA_02438 ATP-independent RNA helicase DbpA
Birmingham02410	PROKKA_02439 pH-dependent sodium/proton antiporter
Birmingham02432	PROKKA_02461 hypothetical protein
Birmingham02456	PROKKA_02485 sn-glycerol-3-phosphate transport system permease protein UgpA
Birmingham02474	PROKKA_02505 hypothetical protein
Birmingham02479	PROKKA_02510 hypothetical protein
Birmingham02481	PROKKA_02512 putative malonic semialdehyde reductase RutE
Birmingham02482	PROKKA_02513 hypothetical protein
Birmingham02485	PROKKA_02516 hypothetical protein
Birmingham02486	PROKKA_02517 conjugal transfer protein TraM
Birmingham02490	PROKKA_02521 conjugal transfer relaxase TraI
Birmingham02491	PROKKA_02522 conjugal transfer relaxase TraI
Birmingham02498	PROKKA_02529 Conjugal transfer protein TraG
Birmingham02499	PROKKA_02530 Conjugal transfer protein TraG
Birmingham02501	PROKKA_02532 conjugal transfer protein TrbL
Birmingham02507	PROKKA_02538 conjugal transfer protein TrbF
Birmingham02509	PROKKA_02540 conjugal transfer protein TrbD
Birmingham02510	PROKKA_02541 conjugal transfer protein TrbC
Birmingham02512	PROKKA_02543 Carbon storage regulator
Birmingham02516	PROKKA_02547 hypothetical protein
Birmingham02520	PROKKA_02551 hypothetical protein
Birmingham02521	PROKKA_02552 hypothetical protein
Birmingham02522	PROKKA_02553 putative methanogenesis marker protein 1
Birmingham02523	PROKKA_02554 hypothetical protein

Birmingham02524	PROKKA_02555 hypothetical protein
Birmingham02543	PROKKA_02574 hypothetical protein
Birmingham02549	PROKKA_02580 hypothetical protein
Birmingham02565	PROKKA_02596 hypothetical protein
Birmingham02568	PROKKA_02599 Antibiotic biosynthesis monooxygenase
Birmingham02579	PROKKA_02610 hypothetical protein
Birmingham02583	PROKKA_02614 hypothetical protein
Birmingham02602	PROKKA_02633 Nitric oxide reductase large subunit
Birmingham02611	PROKKA_02642 RasGEF domain protein
Birmingham02646	PROKKA_02677 hypothetical protein
Birmingham02654	PROKKA_02685 hypothetical protein
Birmingham02662	PROKKA_02693 hypothetical protein
Birmingham02663	PROKKA_02694 hypothetical protein
Birmingham02672	PROKKA_02703 hypothetical protein
Birmingham02681	PROKKA_02712 hypothetical protein
Birmingham02682	PROKKA_02713 hypothetical protein
Birmingham02690	PROKKA_02721 hypothetical protein
Birmingham02692	PROKKA_02723 hypothetical protein
Birmingham02697	PROKKA_02728 hypothetical protein
Birmingham02698	PROKKA_02729 hypothetical protein
Birmingham02699	PROKKA_02730 hypothetical protein
Birmingham02700	PROKKA_02731 hypothetical protein
Birmingham02701	PROKKA_02732 Phosphoenolpyruvate synthase
Birmingham02707	PROKKA_02738 hypothetical protein
Birmingham02710	PROKKA_02741 MscS family inner membrane protein YnaI
Birmingham02714	PROKKA_02745 Pilin
Birmingham02725	PROKKA_02756 hypothetical protein
Birmingham02726	PROKKA_02757 hypothetical protein
Birmingham02734	PROKKA_02766 hypothetical protein
Birmingham02735	PROKKA_02767 hypothetical protein

Birmingham02749	PROKKA_02781 hypothetical protein
Birmingham02751	PROKKA_02783 gloeo_Verruco repeat
Birmingham02752	PROKKA_02784 hypothetical protein
Birmingham02757	PROKKA_02789 hypothetical protein
Birmingham02773	PROKKA_02805 Bicarbonate transport system permease protein CmpB
Birmingham02796	PROKKA_02828 hypothetical protein
Birmingham02817	PROKKA_02850 DNA primase
Birmingham02828	PROKKA_02861 Biotin carboxylase
Birmingham02830	PROKKA_02863 hypothetical protein
Birmingham02833	PROKKA_02866 hypothetical protein
Birmingham02835	PROKKA_02868 Aerobic respiration control sensor protein ArcB
Birmingham02836	PROKKA_02869 hypothetical protein
Birmingham02841	PROKKA_02874 hypothetical protein
Birmingham02842	PROKKA_02875 chromosome segregation protein SMC
Birmingham02843	PROKKA_02876 hypothetical protein
Birmingham02844	PROKKA_02877 hypothetical protein
Birmingham02866	PROKKA_02899 hypothetical protein
Birmingham02869	PROKKA_02902 hypothetical protein
Birmingham02887	PROKKA_02920 Glutamate synthase [NADPH] large chain precursor
Birmingham02891	PROKKA_02924 hypothetical protein
Birmingham02893	PROKKA_02926 hypothetical protein
Birmingham02897	PROKKA_02930 hypothetical protein
Birmingham02898	PROKKA_02931 hypothetical protein
Birmingham02907	PROKKA_02940 hypothetical protein
Birmingham02911	PROKKA_02944 hypothetical protein
Birmingham02913	PROKKA_02946 phosphoribose diphosphate:decaprenyl-phosphate phosphoribosyltransferase
Birmingham02914	PROKKA_02947 putative membrane protein
Birmingham02922	PROKKA_02955 hypothetical protein
Birmingham02924	PROKKA_02957 hypothetical protein
Birmingham02925	PROKKA_02958 hypothetical protein

Birmingham02928	PROKKA_02961 hypothetical protein
Birmingham02930	PROKKA_02963 ankyrin repeat protein
Birmingham02932	PROKKA_02965 hypothetical protein
Birmingham02933	PROKKA_02966 Deoxyguanosinetriphosphate triphosphohydrolase
Birmingham02937	PROKKA_02970 Guanylate kinase
Birmingham02938	PROKKA_02971 DNA-invertase hin
Birmingham02939	PROKKA_02972 hypothetical protein
Birmingham02940	PROKKA_02973 Transposon Tn3 resolvase
Birmingham02943	PROKKA_02976 hypothetical protein
Birmingham02945	PROKKA_02978 hypothetical protein
Birmingham02946	PROKKA_02979 hypothetical protein
Birmingham02947	PROKKA_02980 hypothetical protein
Birmingham02951	PROKKA_02984 hypothetical protein
Birmingham02953	PROKKA_02986 putative transposase OrfB
Birmingham02956	PROKKA_02989 hypothetical protein
Birmingham02957	PROKKA_02990 hypothetical protein
Birmingham02958	PROKKA_02991 hypothetical protein
Birmingham02962	PROKKA_02995 hypothetical protein
Birmingham02975	PROKKA_03008 Signal transduction histidine kinase
Birmingham02976	PROKKA_03009 hypothetical protein
Birmingham02977	PROKKA_03010 Ribbon-helix-helix protein, copG family
Birmingham02978	PROKKA_03011 Toxin RelE2
Birmingham02979	PROKKA_03012 putative transposase OrfB
Birmingham02980	PROKKA_03013 Transposase
Birmingham02986	PROKKA_03019 Pilin
Birmingham02987	PROKKA_03020 hypothetical protein
Birmingham02994	PROKKA_03028 hypothetical protein
Birmingham03005	PROKKA_03039 hypothetical protein
Birmingham03007	PROKKA_03041 Tfp pilus assembly protein, tip-associated adhesin PilY1
Birmingham03018	PROKKA_03052 hypothetical protein

Birmingham03021	PROKKA_03055 hypothetical protein
Birmingham03037	PROKKA_03071 hypothetical protein
Birmingham03048	PROKKA_03083 Outer membrane protein transport protein (OMPP1/FadL/TodX)
Birmingham03063	PROKKA_03098 queuosine biosynthesis protein QueD
Birmingham03066	PROKKA_03101 poly-beta-1,6 N-acetyl-D-glucosamine synthase
Birmingham03072	PROKKA_03107 Nuclease NucM precursor
Birmingham03080	PROKKA_03115 hypothetical protein
Birmingham03088	PROKKA_03123 Silver exporting P-type ATPase
Birmingham03093	PROKKA_03128 hypothetical protein
Birmingham03106	PROKKA_03141 hypothetical protein
Birmingham03111	PROKKA_03146 hypothetical protein
Birmingham03112	PROKKA_03147 hypothetical protein
Birmingham03117	PROKKA_03152 hypothetical protein
Birmingham03127	PROKKA_03162 hypothetical protein
Birmingham03129	PROKKA_03164 hypothetical protein
Birmingham03131	PROKKA_03166 hypothetical protein
Birmingham03132	PROKKA_03167 hypothetical protein
Birmingham03134	PROKKA_03169 hypothetical protein
Birmingham03137	PROKKA_03172 hypothetical protein
Birmingham03138	PROKKA_03173 hypothetical protein
Birmingham03140	PROKKA_03175 hypothetical protein
Birmingham03148	PROKKA_03183 hypothetical protein
Birmingham03149	PROKKA_03184 Efflux pump membrane transporter BepG
Birmingham03166	PROKKA_03201 hypothetical protein
Birmingham03172	PROKKA_03207 hypothetical protein
Birmingham03176	PROKKA_03211 conjugal transfer protein TraL
Birmingham03177	PROKKA_03212 conjugal transfer protein TrbG
Birmingham03179	PROKKA_03214 Type IV secretion system protein virB4
Birmingham03186	PROKKA_03221 hypothetical protein
Birmingham03187	PROKKA_03222 hypothetical protein

Birmingham03189	PROKKA_03224 hypothetical protein
Birmingham03194	PROKKA_03229 Sodium/proton antiporter NhaA
Birmingham03202	PROKKA_03237 hypothetical protein
Birmingham03203	PROKKA_03238 hypothetical protein
Birmingham03220	PROKKA_03255 hypothetical protein
Birmingham03221	PROKKA_03256 hypothetical protein
Birmingham03224	PROKKA_03259 Malate synthase A
Birmingham03234	PROKKA_03269 hypothetical protein
Birmingham03242	PROKKA_03278 Superfamily II helicase
Birmingham03246	PROKKA_03282 hypothetical protein
Birmingham03247	PROKKA_03283 hypothetical protein
Birmingham03248	PROKKA_03284 acetoin dehydrogenase E2 subunit dihydrolipoyllysine-residue acetyltransferase
Birmingham03251	PROKKA_03287 Superfamily II helicase
Birmingham03257	PROKKA_03293 hypothetical protein
Birmingham03258	PROKKA_03294 hypothetical protein
Birmingham03260	PROKKA_03296 hypothetical protein
Birmingham03261	PROKKA_03297 PQQ-dependent catabolism-associated beta-propeller protein
Birmingham03263	PROKKA_03299 hypothetical protein
Birmingham03264	PROKKA_03300 hypothetical protein
Birmingham03268	PROKKA_03304 Phage envelope protein
Birmingham03271	PROKKA_03307 hypothetical protein
Birmingham03272	PROKKA_03308 Excinuclease cho
Birmingham03288	PROKKA_03325 SagB-type dehydrogenase domain protein
Birmingham03289	PROKKA_03326 hypothetical protein
Birmingham03290	PROKKA_03327 hypothetical protein
Birmingham03291	PROKKA_03328 hypothetical protein
Birmingham03293	PROKKA_03330 SagB-type dehydrogenase domain protein
Birmingham03295	PROKKA_03332 hypothetical protein
Birmingham03302	PROKKA_03339 hypothetical protein
Birmingham03304	PROKKA_03341 hypothetical protein

Birmingham03306	PROKKA_03343 hypothetical protein
Birmingham03308	PROKKA_03345 hypothetical protein
Birmingham03309	PROKKA_03346 hypothetical protein
Birmingham03316	PROKKA_03357 hypothetical protein
brunen00002	PROKKA_00002 hypothetical protein
brunen00027	PROKKA_00027 hypothetical protein
brunen00037	PROKKA_00038 hypothetical protein
brunen00038	PROKKA_00039 hypothetical protein
brunen00084	PROKKA_00085 hypothetical protein
brunen00085	PROKKA_00086 hypothetical protein
brunen00086	PROKKA_00087 hypothetical protein
brunen00087	PROKKA_00088 hypothetical protein
brunen00093	PROKKA_00094 hypothetical protein
brunen00094	PROKKA_00095 Tyrosine recombinase XerC
brunen00095	PROKKA_00096 hypothetical protein
brunen00103	PROKKA_00104 SPFH domain / Band 7 family protein
brunen00109	PROKKA_00110 hypothetical protein
brunen00117	PROKKA_00118 hypothetical protein
brunen00144	PROKKA_00145 hypothetical protein
brunen00184	PROKKA_00185 hypothetical protein
brunen00188	PROKKA_00189 hypothetical protein
brunen00222	PROKKA_00225 hypothetical protein
brunen00246	PROKKA_00249 hypothetical protein
brunen00250	PROKKA_00253 hypothetical protein
brunen00267	PROKKA_00270 hypothetical protein
brunen00278	PROKKA_00281 hypothetical protein
brunen00286	PROKKA_00289 hypothetical protein
brunen00289	PROKKA_00292 hypothetical protein
brunen00304	PROKKA_00307 Quercetin 2,3-dioxygenase
brunen00318	PROKKA_00321 hypothetical protein

brunen00329	PROKKA_00332 hypothetical protein
brunen00345	PROKKA_00348 Xylene monooxygenase electron transfer component
brunen00349	PROKKA_00352 hypothetical protein
brunen00350	PROKKA_00353 hypothetical protein
brunen00382	PROKKA_00385 hypothetical protein
brunen00383	PROKKA_00386 hypothetical protein
brunen00384	PROKKA_00387 hypothetical protein
brunen00401	PROKKA_00404 hypothetical protein
brunen00402	PROKKA_00405 hypothetical protein
brunen00414	PROKKA_00417 3-isopropylmalate dehydratase small subunit
brunen00445	PROKKA_00448 hypothetical protein
brunen00479	PROKKA_00482 hypothetical protein
brunen00484	PROKKA_00487 hypothetical protein
brunen00497	PROKKA_00500 Linear gramicidin synthase subunit D
brunen00498	PROKKA_00501 hypothetical protein
brunen00506	PROKKA_00509 putative ATPase (AAA+ superfamily)
brunen00507	PROKKA_00510 hypothetical protein
brunen00508	PROKKA_00511 poly(A) polymerase I
brunen00519	PROKKA_00522 hypothetical protein
brunen00531	PROKKA_00535 DNA primase
brunen00532	PROKKA_00536 DNA primase (bacterial type)
brunen00536	PROKKA_00540 putative PEP-CTERM system TPR-repeat lipoprotein
brunen00537	PROKKA_00541 MORN repeat variant
brunen00548	PROKKA_00552 hypothetical protein
brunen00553	PROKKA_00557 haloacid dehalogenase-like hydrolase
brunen00555	PROKKA_00559 6'''-hydroxyparomomycin C oxidase
brunen00572	PROKKA_00577 hypothetical protein
brunen00574	PROKKA_00579 RasGEF domain protein
brunen00582	PROKKA_00587 hypothetical protein
brunen00609	PROKKA_00614 Polyketide biosynthesis protein PksE

brunen00610	PROKKA_00615 4-hydroxyacetophenone monooxygenase
brunen00612	PROKKA_00617 3-oxoacyl-[acyl-carrier-protein] synthase 3
brunen00614	PROKKA_00619 Long-chain-fatty-acid--CoA ligase
brunen00616	PROKKA_00621 Beta-ketoacyl-acyl-carrier-protein synthase I
brunen00617	PROKKA_00622 Polyketide synthase PksL
brunen00656	PROKKA_00661 serine/threonine protein kinase
brunen00657	PROKKA_00662 hypothetical protein
brunen00659	PROKKA_00664 hypothetical protein
brunen00667	PROKKA_00672 hypothetical protein
brunen00715	PROKKA_00720 hypothetical protein
brunen00732	PROKKA_00737 hypothetical protein
brunen00786	PROKKA_00791 O-antigen ligase RfaL
brunen00795	PROKKA_00800 hypothetical protein
brunen00807	PROKKA_00812 NADPH-dependent 7-cyano-7-deazaguanine reductase
brunen00847	PROKKA_00852 hypothetical protein
brunen00848	PROKKA_00853 hypothetical protein
brunen00867	PROKKA_00872 Putative ribosomal N-acetyltransferase YdaF
brunen00917	PROKKA_00922 Protein tyrosine kinase
brunen00918	PROKKA_00923 hypothetical protein
brunen00966	PROKKA_00972 hypothetical protein
brunen01016	PROKKA_01022 hypothetical protein
brunen01019	PROKKA_01025 hypothetical protein
brunen01027	PROKKA_01033 hypothetical protein
brunen01038	PROKKA_01044 hypothetical protein
brunen01056	PROKKA_01062 hypothetical protein
brunen01060	PROKKA_01066 hypothetical protein
brunen01072	PROKKA_01079 hypothetical protein
brunen01077	PROKKA_01084 hypothetical protein
brunen01086	PROKKA_01093 hypothetical protein
brunen01090	PROKKA_01097 hypothetical protein

brunen01098	PROKKA_01105 hypothetical protein
brunen01100	PROKKA_01107 hypothetical protein
brunen01127	PROKKA_01134 D-malate degradation protein R
brunen01132	PROKKA_01139 hypothetical protein
brunen01137	PROKKA_01144 hypothetical protein
brunen01160	PROKKA_01170 Penicillin V acylase
brunen01167	PROKKA_01178 bZIP transcription factor
brunen01171	PROKKA_01182 hypothetical protein
brunen01173	PROKKA_01184 hypothetical protein
brunen01182	PROKKA_01193 hypothetical protein
brunen01189	PROKKA_01200 hypothetical protein
brunen01236	PROKKA_01247 hypothetical protein
brunen01238	PROKKA_01249 D-beta-hydroxybutyrate dehydrogenase
brunen01239	PROKKA_01250 3-oxoacyl-[acyl-carrier-protein] reductase FabG
brunen01241	PROKKA_01253 hypothetical protein
brunen01242	PROKKA_01254 Opacity protein antigens
brunen01243	PROKKA_01255 Legionella pneumophila major outer membrane protein precursor
brunen01253	PROKKA_01265 hypothetical protein
brunen01257	PROKKA_01269 3-deoxy-D-manno-octulosonic acid kinase
brunen01259	PROKKA_01271 Lipid A 3-O-deacylase (PagL)
brunen01260	PROKKA_01272 ADP-heptose--LPS heptosyltransferase 2
brunen01263	PROKKA_01275 hypothetical protein
brunen01264	PROKKA_01276 hypothetical protein
brunen01272	PROKKA_01284 hypothetical protein
brunen01280	PROKKA_01292 hypothetical protein
brunen01298	PROKKA_01310 hypothetical protein
brunen01299	PROKKA_01311 hypothetical protein
brunen01301	PROKKA_01313 hypothetical protein
brunen01305	PROKKA_01317 hypothetical protein
brunen01309	PROKKA_01321 hypothetical protein

brunen01320	PROKKA_01332 Beta-lactamase
brunen01323	PROKKA_01335 Plastocyanin precursor
brunen01329	PROKKA_01341 hypothetical protein
brunen01332	PROKKA_01344 Bifunctional xylanase/deacetylase precursor
brunen01347	PROKKA_01359 hypothetical protein
brunen01351	PROKKA_01363 hypothetical protein
brunen01354	PROKKA_01366 Guanine deaminase
brunen01356	PROKKA_01368 LPS biosynthesis protein
brunen01379	PROKKA_01391 hypothetical protein
brunen01380	PROKKA_01392 hypothetical protein
brunen01381	PROKKA_01393 hypothetical protein
brunen01391	PROKKA_01404 hypothetical protein
brunen01392	PROKKA_01405 Carboxylate-amine ligase YbdK
brunen01411	PROKKA_01424 Deoxyribodipyrimidine photo-lyase
brunen01423	PROKKA_01436 hypothetical protein
brunen01425	PROKKA_01438 hypothetical protein
brunen01426	PROKKA_01439 3-oxoacyl-[acyl-carrier-protein] synthase 2
brunen01451	PROKKA_01464 hypothetical protein
brunen01452	PROKKA_01465 hypothetical protein
brunen01453	PROKKA_01466 hypothetical protein
brunen01456	PROKKA_01469 hypothetical protein
brunen01510	PROKKA_01523 hypothetical protein
brunen01522	PROKKA_01535 hypothetical protein
brunen01574	PROKKA_01588 Ankyrin repeats (3 copies)
brunen01613	PROKKA_01627 hypothetical protein
brunen01623	PROKKA_01637 hypothetical protein
brunen01624	PROKKA_01638 hypothetical protein
brunen01627	PROKKA_01641 Serine/threonine-protein kinase AfsK
brunen01647	PROKKA_01661 hypothetical protein
brunen01677	PROKKA_01692 hypothetical protein

brunen01684	PROKKA_01699 hypothetical protein
brunen01713	PROKKA_01728 Dual specificity phosphatase, catalytic domain
brunen01728	PROKKA_01743 hypothetical protein
brunen01761	PROKKA_01782 hypothetical protein
brunen01788	PROKKA_01812 Peptidase C80 family protein
brunen01826	PROKKA_01851 hypothetical protein
brunen01827	PROKKA_01852 hypothetical protein
brunen01828	PROKKA_01853 hypothetical protein
brunen01830	PROKKA_01855 Ornithine carbamoyltransferase
brunen01831	PROKKA_01856 Arginine-binding extracellular protein ArtP precursor
brunen01833	PROKKA_01858 hypothetical protein
brunen01846	PROKKA_01871 putative esterase of the alpha/beta hydrolase fold protein
brunen01849	PROKKA_01874 Glycine betaine transport ATP-binding protein OpuAA
brunen01850	PROKKA_01875 Glycine betaine transport system permease protein OpuAB
brunen01851	PROKKA_01876 Glycine betaine/carnitine transport binding protein GbuC precursor
brunen01853	PROKKA_01878 hypothetical protein
brunen01860	PROKKA_01885 Enoyl-[acyl-carrier-protein] reductase [NADH] FabI
brunen01907	PROKKA_01932 hypothetical protein
brunen01924	PROKKA_01952 hypothetical protein
brunen01971	PROKKA_01999 hypothetical protein
brunen01979	PROKKA_02007 hypothetical protein
brunen01996	PROKKA_02024 K+-transporting ATPase, F subunit
brunen02010	PROKKA_02038 hypothetical protein
brunen02059	PROKKA_02088 hypothetical protein
brunen02081	PROKKA_02110 Bacterial transferase hexapeptide (six repeats)
brunen02082	PROKKA_02111 hypothetical protein
brunen02090	PROKKA_02119 hypothetical protein
brunen02091	PROKKA_02120 putative RecB family nuclease, family
brunen02102	PROKKA_02132 hypothetical protein
brunen02134	PROKKA_02164 hypothetical protein

brunen02145	PROKKA_02175 hypothetical protein
brunen02190	PROKKA_02221 hypothetical protein
brunen02229	PROKKA_02260 D-alanyl-D-alanine dipeptidase
brunen02249	PROKKA_02281 hypothetical protein
brunen02252	PROKKA_02284 N-acyltransferase YncA
brunen02302	PROKKA_02335 hypothetical protein
brunen02306	PROKKA_02339 hypothetical protein
brunen02307	PROKKA_02340 hypothetical protein
brunen02308	PROKKA_02341 hypothetical protein
brunen02315	PROKKA_02348 hypothetical protein
brunen02316	PROKKA_02349 hypothetical protein
brunen02352	PROKKA_02385 PQ loop repeat
brunen02353	PROKKA_02386 PQ loop repeat
brunen02426	PROKKA_02459 acetolactate synthase 3 regulatory subunit
brunen02451	PROKKA_02484 hypothetical protein
brunen02475	PROKKA_02508 2,4-dichlorophenol 6-monooxygenase
brunen02529	PROKKA_02562 hypothetical protein
brunen02539	PROKKA_02572 Acetophenone carboxylase delta subunit
brunen02541	PROKKA_02574 putative small protein
brunen02543	PROKKA_02576 hypothetical protein
brunen02547	PROKKA_02580 hypothetical protein
brunen02566	PROKKA_02599 hypothetical protein
brunen02569	PROKKA_02602 hypothetical protein
brunen02587	PROKKA_02620 hypothetical protein
brunen02588	PROKKA_02621 hypothetical protein
brunen02593	PROKKA_02626 hypothetical protein
brunen02594	PROKKA_02627 hypothetical protein
brunen02599	PROKKA_02632 hypothetical protein
brunen02615	PROKKA_02648 hypothetical protein
brunen02639	PROKKA_02672 hypothetical protein

brunen02640	PROKKA_02673 Capsule polysaccharide biosynthesis protein
brunen02641	PROKKA_02674 hypothetical protein
brunen02642	PROKKA_02675 Heparinase II/III-like protein
brunen02660	PROKKA_02693 dTDP-fucosamine acetyltransferase
brunen02701	PROKKA_02735 Magnesium transport protein CorA
brunen02725	PROKKA_02759 hypothetical protein
brunen02746	PROKKA_02780 hypothetical protein
brunen02749	PROKKA_02783 hypothetical protein
brunen02764	PROKKA_02798 Ferrochelatase
brunen02772	PROKKA_02806 short chain dehydrogenase
brunen02817	PROKKA_02851 hypothetical protein
brunen02832	PROKKA_02866 hypothetical protein
brunen02840	PROKKA_02874 hypothetical protein
brunen02841	PROKKA_02875 hypothetical protein
brunen02843	PROKKA_02877 Aerobic C4-dicarboxylate transport protein
brunen02870	PROKKA_02904 hypothetical protein
brunen02872	PROKKA_02906 hypothetical protein
brunen02879	PROKKA_02913 Thaumatin family protein
brunen02880	PROKKA_02914 hypothetical protein
brunen02886	PROKKA_02920 hypothetical protein
brunen02889	PROKKA_02923 hypothetical protein
brunen02909	PROKKA_02943 hypothetical protein
brunen02914	PROKKA_02948 hypothetical protein
brunen02920	PROKKA_02954 hypothetical protein
brunen02921	PROKKA_02955 hypothetical protein
brunen02961	PROKKA_02995 hypothetical protein
brunen02964	PROKKA_02998 prevent-host-death family protein
brunen03042	PROKKA_03078 hypothetical protein
brunen03050	PROKKA_03086 succinate dehydrogenase cytochrome b556 large membrane subunit
brunen03053	PROKKA_03089 bifunctional malic enzyme oxidoreductase/phosphotransacetylase

brunen03054	PROKKA_03090 NAD-dependent malic enzyme
brunen03063	PROKKA_03099 hypothetical protein
brunen03105	PROKKA_03141 Guanylyl cyclase
brunen03106	PROKKA_03142 putative amino acid permease YhdG
brunen03110	PROKKA_03146 hypothetical protein
brunen03156	PROKKA_03192 hypothetical protein
brunen03169	PROKKA_03205 ankyrin repeat protein
brunen03215	PROKKA_03252 acyl-CoA thioester hydrolase, YbgC/YbaW family
brunen03255	PROKKA_03292 hypothetical protein
brunen03256	PROKKA_03293 hypothetical protein
brunen03257	PROKKA_03294 hypothetical protein
brunen03259	PROKKA_03296 site-specific tyrosine recombinase XerC
brunen03263	PROKKA_03300 hypothetical protein
brunen03270	PROKKA_03307 hypothetical protein
brunen03275	PROKKA_03312 hypothetical protein
brunen03285	PROKKA_03322 hypothetical protein
erythra00001	PROKKA_00001 putative HTH-type transcriptional regulator
erythra00016	PROKKA_00016 hypothetical protein
erythra00017	PROKKA_00017 hypothetical protein
erythra00023	PROKKA_00026 hypothetical protein
erythra00027	PROKKA_00030 hypothetical protein
erythra00034	PROKKA_00037 hypothetical protein
erythra00064	PROKKA_00067 hypothetical protein
erythra00069	PROKKA_00072 hypothetical protein
erythra00073	PROKKA_00076 hybrid sensory histidine kinase BarA
erythra00075	PROKKA_00078 Pilin
erythra00081	PROKKA_00084 Extreme acid sensitivity protein
erythra00082	PROKKA_00085 Glutamate/gamma-aminobutyrate antiporter
erythra00158	PROKKA_00161 Dna-J like membrane chaperone protein
erythra00168	PROKKA_00171 Aromatic amino acid exporter YddG

erythra00171	PROKKA_00174 hypothetical protein
erythra00173	PROKKA_00176 hypothetical protein
erythra00191	PROKKA_00194 Macrophage killing protein with similarity to conjugation protein
erythra00196	PROKKA_00199 hypothetical protein
erythra00199	PROKKA_00202 hypothetical protein
erythra00240	PROKKA_00243 hypothetical protein
erythra00243	PROKKA_00246 Lactococcin-G-processing and transport ATP-binding protein LagD
erythra00246	PROKKA_00249 hypothetical protein
erythra00247	PROKKA_00250 hypothetical protein
erythra00262	PROKKA_00265 hypothetical protein
erythra00271	PROKKA_00274 hypothetical protein
erythra00296	PROKKA_00299 hypothetical protein
erythra00300	PROKKA_00303 hypothetical protein
erythra00306	PROKKA_00309 Coniferyl aldehyde dehydrogenase
erythra00315	PROKKA_00318 Ribonuclease HII
erythra00318	PROKKA_00321 PAS domain S-box protein
erythra00371	PROKKA_00374 hypothetical protein
erythra00423	PROKKA_00426 hypothetical protein
erythra00424	PROKKA_00427 hypothetical protein
erythra00425	PROKKA_00428 Isotuberculosinol synthase
erythra00426	PROKKA_00429 Type B diterpene cyclase
erythra00427	PROKKA_00430 Type B diterpene cyclase
erythra00452	PROKKA_00455 rickettsial palindromic element RPE4 domain protein
erythra00478	PROKKA_00482 tRNA pseudouridine synthase D
erythra00497	PROKKA_00501 Bacteriophytochrome cph2
erythra00498	PROKKA_00502 hypothetical protein
erythra00499	PROKKA_00503 PilD-dependent protein PddA
erythra00508	PROKKA_00512 hypothetical protein
erythra00521	PROKKA_00525 hypothetical protein
erythra00532	PROKKA_00536 hypothetical protein

erythra00533	PROKKA_00537 hypothetical protein
erythra00546	PROKKA_00550 hypothetical protein
erythra00547	PROKKA_00551 hypothetical protein
erythra00556	PROKKA_00560 Sporulation initiation inhibitor protein soj
erythra00561	PROKKA_00565 hypothetical protein
erythra00589	PROKKA_00593 Endoglucanase precursor
erythra00602	PROKKA_00606 hypothetical protein
erythra00603	PROKKA_00607 hypothetical protein
erythra00624	PROKKA_00628 hypothetical protein
erythra00630	PROKKA_00634 hypothetical protein
erythra00637	PROKKA_00641 putative ubiquinone biosynthesis protein UbiB
erythra00641	PROKKA_00645 putative periplasmic ligand-binding sensor domain protein
erythra00642	PROKKA_00646 putative diguanylate cyclase YegE
erythra00656	PROKKA_00660 hypothetical protein
erythra00658	PROKKA_00663 putative enzyme related to lactoylglutathione lyase
erythra00662	PROKKA_00667 Bacteriophytochrome cph2
erythra00663	PROKKA_00668 phosphate regulon transcriptional regulatory protein PhoB
erythra00664	PROKKA_00669 hypothetical protein
erythra00675	PROKKA_00680 hypothetical protein
erythra00685	PROKKA_00690 hypothetical protein
erythra00710	PROKKA_00715 hypothetical protein
erythra00743	PROKKA_00749 hypothetical protein
erythra00820	PROKKA_00826 Phospholipase A1 precursor
erythra00836	PROKKA_00843 hypothetical protein
erythra00841	PROKKA_00848 Spermidine synthase
erythra00865	PROKKA_00872 hypothetical protein
erythra00928	PROKKA_00936 Uroporphyrinogen decarboxylase
erythra00929	PROKKA_00937 Delta-aminolevulinic acid dehydratase
erythra00931	PROKKA_00939 Blue light- and temperature-regulated antirepressor YcgF
erythra00935	PROKKA_00943 Lipoprotein-releasing system ATP-binding protein LolD

erythra00936	PROKKA_00944 Glutamate-1-semialdehyde 2,1-aminomutase
erythra00939	PROKKA_00947 Aerobic cobaltochelatase subunit CobN
erythra00940	PROKKA_00948 uroporphyrinogen-III synthase
erythra00943	PROKKA_00951 Coproporphyrinogen-III oxidase, aerobic
erythra00945	PROKKA_00953 hypothetical protein
erythra00946	PROKKA_00954 hypothetical protein
erythra00948	PROKKA_00956 Serine/threonine-protein kinase PknB
erythra00949	PROKKA_00957 hypothetical protein
erythra00956	PROKKA_00964 Site-specific recombinase XerC
erythra00963	PROKKA_00971 hypothetical protein
erythra00974	PROKKA_00982 Type IV secretion system protein virB4
erythra01000	PROKKA_01008 hypothetical protein
erythra01005	PROKKA_01013 hypothetical protein
erythra01007	PROKKA_01015 hypothetical protein
erythra01009	PROKKA_01017 hypothetical protein
erythra01010	PROKKA_01018 hypothetical protein
erythra01011	PROKKA_01019 hypothetical protein
erythra01017	PROKKA_01025 hypothetical protein
erythra01045	PROKKA_01053 hypothetical protein
erythra01113	PROKKA_01121 succinic semialdehyde dehydrogenase
erythra01124	PROKKA_01132 hypothetical protein
erythra01138	PROKKA_01148 hypothetical protein
erythra01147	PROKKA_01157 hypothetical protein
erythra01148	PROKKA_01158 hypothetical protein
erythra01153	PROKKA_01163 hypothetical protein
erythra01156	PROKKA_01166 hypothetical protein
erythra01157	PROKKA_01167 hypothetical protein
erythra01159	PROKKA_01169 hypothetical protein
erythra01160	PROKKA_01170 hypothetical protein
erythra01161	PROKKA_01171 hypothetical protein

erythra01193	PROKKA_01203 ankyrin repeat protein
erythra01209	PROKKA_01219 hypothetical protein
erythra01219	PROKKA_01229 Acyl-[acyl-carrier-protein]--UDP-N-acetylglucosamine O-acyltransferase
erythra01240	PROKKA_01250 hypothetical protein
erythra01241	PROKKA_01251 hypothetical protein
erythra01256	PROKKA_01266 hypothetical protein
erythra01271	PROKKA_01281 hypothetical protein
erythra01292	PROKKA_01302 hypothetical protein
erythra01306	PROKKA_01316 hypothetical protein
erythra01311	PROKKA_01321 hypothetical protein
erythra01313	PROKKA_01323 hypothetical protein
erythra01316	PROKKA_01326 hypothetical protein
erythra01330	PROKKA_01340 hypothetical protein
erythra01339	PROKKA_01349 Methyltransferase domain protein
erythra01342	PROKKA_01352 hypothetical protein
erythra01343	PROKKA_01353 hypothetical protein
erythra01344	PROKKA_01354 hypothetical protein
erythra01346	PROKKA_01356 arsenite S-adenosylmethyltransferase
erythra01348	PROKKA_01358 WbqC-like protein family protein
erythra01355	PROKKA_01365 hypothetical protein
erythra01357	PROKKA_01367 UDP-glucose 4-epimerase
erythra01378	PROKKA_01388 Methyltransferase domain protein
erythra01388	PROKKA_01398 hypothetical protein
erythra01400	PROKKA_01410 Glutamate/gamma-aminobutyrate antiporter
erythra01418	PROKKA_01428 hypothetical protein
erythra01450	PROKKA_01460 hypothetical protein
erythra01452	PROKKA_01462 hypothetical protein
erythra01460	PROKKA_01470 Cyanophycin synthetase
erythra01465	PROKKA_01475 glutaredoxin 1
erythra01488	PROKKA_01498 hypothetical protein

erythra01496	PROKKA_01506 ATP synthase subunit alpha
erythra01503	PROKKA_01513 hypothetical protein
erythra01533	PROKKA_01543 hypothetical protein
erythra01574	PROKKA_01584 hypothetical protein
erythra01575	PROKKA_01585 hypothetical protein
erythra01583	PROKKA_01593 hypothetical protein
erythra01588	PROKKA_01598 hypothetical protein
erythra01594	PROKKA_01604 ADP-heptose:LPS heptosyltransferase II
erythra01595	PROKKA_01605 hypothetical protein
erythra01628	PROKKA_01638 hypothetical protein
erythra01629	PROKKA_01639 hypothetical protein
erythra01652	PROKKA_01662 ATP-dependent DNA helicase RecQ
erythra01684	PROKKA_01695 Assimilatory nitrate reductase catalytic subunit
erythra01698	PROKKA_01710 hypothetical protein
erythra01724	PROKKA_01736 hypothetical protein
erythra01725	PROKKA_01737 Histone deacetylase-like amidohydrolase
erythra01733	PROKKA_01745 pyrroloquinoline quinone biosynthesis protein PqqE
erythra01734	PROKKA_01746 hypothetical protein
erythra01735	PROKKA_01747 hypothetical protein
erythra01758	PROKKA_01771 Streptogramin lyase
erythra01761	PROKKA_01774 hypothetical protein
erythra01774	PROKKA_01787 hypothetical protein
erythra01790	PROKKA_01803 hypothetical protein
erythra01796	PROKKA_01809 hypothetical protein
erythra01801	PROKKA_01814 hypothetical protein
erythra01841	PROKKA_01855 hypothetical protein
erythra01844	PROKKA_01858 hypothetical protein
erythra01867	PROKKA_01881 hypothetical protein
erythra01873	PROKKA_01887 hypothetical protein
erythra01894	PROKKA_01908 hypothetical protein

erythra01896	PROKKA_01910 hypothetical protein
erythra01900	PROKKA_01914 Inner membrane transport protein YajR
erythra01905	PROKKA_01919 PQQ-dependent catabolism-associated beta-propeller protein
erythra01906	PROKKA_01920 HTH-type transcriptional activator AllS
erythra01915	PROKKA_01929 Homospermidine synthase
erythra01921	PROKKA_01935 D-galactonate transporter
erythra01927	PROKKA_01941 hypothetical protein
erythra01929	PROKKA_01943 hypothetical protein
erythra01936	PROKKA_01950 hypothetical protein
erythra01954	PROKKA_01968 hypothetical protein
erythra01965	PROKKA_01979 hypothetical protein
erythra01966	PROKKA_01980 hypothetical protein
erythra01969	PROKKA_01983 Gluconate 5-dehydrogenase
erythra01970	PROKKA_01984 hypothetical protein
erythra01973	PROKKA_01987 hypothetical protein
erythra01976	PROKKA_01990 hypothetical protein
erythra01978	PROKKA_01992 hypothetical protein
erythra01985	PROKKA_01999 hypothetical protein
erythra01986	PROKKA_02000 5-methyltetrahydropteroyltriglutamate--homocysteine methyltransferase
erythra01996	PROKKA_02010 Sulfotransferase domain protein
erythra01997	PROKKA_02011 bacteriocin biosynthesis docking scaffold, SagD family
erythra01999	PROKKA_02013 hypothetical protein
erythra02000	PROKKA_02014 hypothetical protein
erythra02001	PROKKA_02015 hypothetical protein
erythra02002	PROKKA_02016 hypothetical protein
erythra02008	PROKKA_02022 hypothetical protein
erythra02010	PROKKA_02024 hypothetical protein
erythra02011	PROKKA_02025 hypothetical protein
erythra02025	PROKKA_02039 Ribonuclease
erythra02056	PROKKA_02070 site-specific tyrosine recombinase XerC

erythra02075	PROKKA_02089 hypothetical protein
erythra02085	PROKKA_02099 hypothetical protein
erythra02090	PROKKA_02104 hypothetical protein
erythra02092	PROKKA_02106 hypothetical protein
erythra02107	PROKKA_02121 hypothetical protein
erythra02111	PROKKA_02125 hypothetical protein
erythra02140	PROKKA_02154 hypothetical protein
erythra02141	PROKKA_02155 Miro-like protein
erythra02142	PROKKA_02156 hypothetical protein
erythra02146	PROKKA_02160 hypothetical protein
erythra02151	PROKKA_02165 hypothetical protein
erythra02153	PROKKA_02167 hypothetical protein
erythra02154	PROKKA_02168 HDOD domain protein
erythra02156	PROKKA_02170 hypothetical protein
erythra02157	PROKKA_02171 hypothetical protein
erythra02163	PROKKA_02177 hypothetical protein
erythra02172	PROKKA_02186 hypothetical protein
erythra02179	PROKKA_02193 Response regulator ArlR
erythra02180	PROKKA_02194 Bacteriophytochrome cph2
erythra02181	PROKKA_02195 hypothetical protein
erythra02182	PROKKA_02196 RTX-I toxin determinant B
erythra02183	PROKKA_02197 RTX-I toxin determinant B
erythra02184	PROKKA_02198 Type I secretion system membrane fusion protein PrsE
erythra02185	PROKKA_02199 hypothetical protein
erythra02202	PROKKA_02216 hypothetical protein
erythra02203	PROKKA_02217 Protein tyrosine kinase
erythra02205	PROKKA_02219 hypothetical protein
erythra02206	PROKKA_02220 hypothetical protein
erythra02209	PROKKA_02223 hypothetical protein
erythra02215	PROKKA_02229 hypothetical protein

erythra02219	PROKKA_02233 Phage-related replication protein
erythra02222	PROKKA_02236 hypothetical protein
erythra02224	PROKKA_02238 hypothetical protein
erythra02228	PROKKA_02242 hypothetical protein
erythra02233	PROKKA_02247 hypothetical protein
erythra02270	PROKKA_02284 hypothetical protein
erythra02277	PROKKA_02291 hypothetical protein
erythra02355	PROKKA_02369 hypothetical protein
erythra02364	PROKKA_02378 hypothetical protein
erythra02371	PROKKA_02385 hypothetical protein
erythra02381	PROKKA_02395 hypothetical protein
erythra02393	PROKKA_02407 hypothetical protein
erythra02403	PROKKA_02417 Intracellular septation protein
erythra02429	PROKKA_02443 D-erythrose-4-phosphate dehydrogenase
erythra02449	PROKKA_02464 hypothetical protein
erythra02468	PROKKA_02483 acylglycerophosphoethanolamine acyltransferase
erythra02480	PROKKA_02495 hypothetical protein
erythra02511	PROKKA_02526 hypothetical protein
erythra02515	PROKKA_02530 hypothetical protein
erythra02528	PROKKA_02543 hypothetical protein
erythra02535	PROKKA_02550 hypothetical protein
erythra02537	PROKKA_02552 putative small periplasmic lipoprotein
erythra02540	PROKKA_02555 hypothetical protein
erythra02542	PROKKA_02557 ATP-dependent DNA helicase RecG
erythra02555	PROKKA_02570 beta-phosphoglucomutase family hydrolase
erythra02577	PROKKA_02593 hypothetical protein
erythra02578	PROKKA_02594 hypothetical protein
erythra02596	PROKKA_02612 hypothetical protein
erythra02599	PROKKA_02615 Glycosyl hydrolases family 18
erythra02603	PROKKA_02619 Bacterial capsule synthesis protein PGA_cap

erythra02604	PROKKA_02620 hypothetical protein
erythra02605	PROKKA_02621 hypothetical protein
erythra02606	PROKKA_02622 hypothetical protein
erythra02607	PROKKA_02623 hypothetical protein
erythra02610	PROKKA_02626 hypothetical protein
erythra02615	PROKKA_02631 ATP-utilizing enzymes of the PP-loop superfamily protein
erythra02616	PROKKA_02632 phosphoribosylaminoimidazole carboxylase, catalytic subunit
erythra02617	PROKKA_02633 hypothetical protein
erythra02618	PROKKA_02634 hypothetical protein
erythra02619	PROKKA_02635 hypothetical protein
erythra02620	PROKKA_02636 Signal transduction histidine kinase regulating C4-dicarboxylate transport system
erythra02621	PROKKA_02637 Sensor protein ZraS
erythra02632	PROKKA_02648 conjugal transfer mating pair stabilization protein TraN
erythra02644	PROKKA_02660 Enterobacterial TraT complement resistance protein
erythra02654	PROKKA_02671 hypothetical protein
erythra02656	PROKKA_02673 hypothetical protein
erythra02658	PROKKA_02675 hypothetical protein
erythra02661	PROKKA_02678 hypothetical protein
erythra02662	PROKKA_02679 Transposase
erythra02663	PROKKA_02680 hypothetical protein
erythra02664	PROKKA_02681 hypothetical protein
erythra02667	PROKKA_02684 CTP pyrophosphohydrolase
erythra02677	PROKKA_02694 hypothetical protein
erythra02692	PROKKA_02710 hypothetical protein
erythra02693	PROKKA_02711 hypothetical protein
erythra02696	PROKKA_02714 Cytochrome d ubiquinol oxidase subunit 2
erythra02703	PROKKA_02721 hypothetical protein
erythra02705	PROKKA_02723 hypothetical protein
erythra02711	PROKKA_02730 Cation efflux system protein CzcB
erythra02715	PROKKA_02734 Sodium/hydrogen exchanger family protein

erythra02723	PROKKA_02742 hypothetical protein
erythra02731	PROKKA_02750 hypothetical protein
erythra02742	PROKKA_02761 hypothetical protein
erythra02746	PROKKA_02765 ATP synthase subunit alpha
erythra02747	PROKKA_02766 ATP synthase
erythra02748	PROKKA_02767 F0F1 ATP synthase subunit gamma
erythra02785	PROKKA_02804 hypothetical protein
erythra02804	PROKKA_02832 Ankyrin repeats (3 copies)
erythra02812	PROKKA_02840 Voltage-gated potassium channel Kch
erythra02813	PROKKA_02841 All-trans-nonaprenyl-diphosphate synthase (geranyl-diphosphate specific)
erythra02826	PROKKA_02854 hypothetical protein
erythra02827	PROKKA_02855 hypothetical protein
erythra02834	PROKKA_02862 hypothetical protein
erythra02842	PROKKA_02870 hypothetical protein
erythra02855	PROKKA_02883 hypothetical protein
erythra02858	PROKKA_02886 hypothetical protein
erythra02863	PROKKA_02891 tRNA(Ile)-lysidine synthase
erythra02870	PROKKA_02898 3-hydroxy-3-methylglutaryl-coenzyme A reductase
erythra02871	PROKKA_02899 mevalonate kinase
erythra02873	PROKKA_02901 hypothetical protein
erythra02874	PROKKA_02902 Integrase core domain protein
erythra02875	PROKKA_02903 hypothetical protein
erythra02888	PROKKA_02917 Cyclic di-GMP phosphodiesterase Gmr
erythra02916	PROKKA_02946 hypothetical protein
erythra02922	PROKKA_02952 hypothetical protein
erythra02929	PROKKA_02959 Response regulator containing a CheY-like receiver domain and an HD-GYP domain protein
erythra02930	PROKKA_02960 Bacteriophytochrome cph2
erythra02931	PROKKA_02961 Bacteriophytochrome cph2
erythra02945	PROKKA_02975 hypothetical protein
erythra02954	PROKKA_02987 hypothetical protein

erythra02955	PROKKA_02988 CTP:phosphocholine cytidylyltransferase involved in choline phosphorylation for cell surface
erythra02961	PROKKA_02995 hypothetical protein
erythra02963	PROKKA_02997 hypothetical protein
erythra02979	PROKKA_03013 hypothetical protein
erythra02988	PROKKA_03022 hypothetical protein
erythra02989	PROKKA_03023 hypothetical protein
erythra02990	PROKKA_03024 hypothetical protein
erythra02992	PROKKA_03026 hypothetical protein
erythra02993	PROKKA_03027 hypothetical protein
erythra02994	PROKKA_03028 DNA protecting protein DprA
erythra02995	PROKKA_03029 hypothetical protein
erythra02996	PROKKA_03030 hypothetical protein
erythra02999	PROKKA_03033 hypothetical protein
erythra03000	PROKKA_03034 hypothetical protein
erythra03003	PROKKA_03037 hypothetical protein
erythra03010	PROKKA_03044 hypothetical protein
erythra03011	PROKKA_03045 hypothetical protein
erythra03017	PROKKA_03051 Isoprenyl transferase
erythra03018	PROKKA_03052 Ditrans,polycis-undecaprenyl-diphosphate synthase ((2E,6E)-farnesyl-diphosphate specific)
erythra03040	PROKKA_03074 Chaperone protein DnaJ
erythra03048	PROKKA_03082 hypothetical protein
erythra03049	PROKKA_03083 hypothetical protein
erythra03063	PROKKA_03097 hypothetical protein
erythra03079	PROKKA_03113 Helix-destabilizing protein
erythra03080	PROKKA_03114 hypothetical protein
erythra03086	PROKKA_03120 hypothetical protein
erythra03099	PROKKA_03133 hypothetical protein
erythra03118	PROKKA_03154 ABC transporter transmembrane region
erythra03134	PROKKA_03170 hypothetical protein
erythra03136	PROKKA_03172 hypothetical protein

erythra03138	PROKKA_03174 MAPEG family protein
erythra03143	PROKKA_03179 Group II intron, maturase-specific domain
erythra03147	PROKKA_03183 hypothetical protein
erythra03154	PROKKA_03190 hypothetical protein
erythra03159	PROKKA_03195 hypothetical protein
erythra03160	PROKKA_03196 Swarming motility protein YbiA
erythra03175	PROKKA_03211 hypothetical protein
erythra03178	PROKKA_03214 hypothetical protein
erythra03179	PROKKA_03215 hypothetical protein
erythra03182	PROKKA_03218 hypothetical protein
erythra03183	PROKKA_03219 hypothetical protein
erythra03187	PROKKA_03223 hypothetical protein
erythra03192	PROKKA_03228 hypothetical protein
erythra03197	PROKKA_03233 hypothetical protein
erythra03201	PROKKA_03237 hypothetical protein
erythra03205	PROKKA_03241 hypothetical protein
erythra03210	PROKKA_03246 hypothetical protein
erythra03213	PROKKA_03249 hypothetical protein
erythra03215	PROKKA_03251 hypothetical protein
erythra03217	PROKKA_03253 F0F1 ATP synthase subunit B
erythra03232	PROKKA_03268 hypothetical protein
erythra03235	PROKKA_03271 hypothetical protein
erythra03236	PROKKA_03272 hypothetical protein
erythra03237	PROKKA_03273 NADP-dependent 3-hydroxy acid dehydrogenase YdfG
erythra03238	PROKKA_03274 hypothetical protein
erythra03239	PROKKA_03275 hypothetical protein
erythra03240	PROKKA_03276 hypothetical protein
erythra03241	PROKKA_03277 hypothetical protein
erythra03250	PROKKA_03286 hypothetical protein
erythra03252	PROKKA_03288 amidase

erythra03253	PROKKA_03289 hypothetical protein
erythra03274	PROKKA_03310 hypothetical protein
erythra03278	PROKKA_03318 hypothetical protein
Jamestown00009	PROKKA_00009 hypothetical protein
Jamestown00046	PROKKA_00046 Ribosomal RNA small subunit methyltransferase H
Jamestown00051	PROKKA_00051 hypothetical protein
Jamestown00052	PROKKA_00052 hypothetical protein
Jamestown00053	PROKKA_00053 hypothetical protein
Jamestown00054	PROKKA_00054 hypothetical protein
Jamestown00055	PROKKA_00055 hypothetical protein
Jamestown00056	PROKKA_00056 hypothetical protein
Jamestown00061	PROKKA_00061 hypothetical protein
Jamestown00062	PROKKA_00062 hypothetical protein
Jamestown00063	PROKKA_00063 hypothetical protein
Jamestown00070	PROKKA_00070 hypothetical protein
Jamestown00072	PROKKA_00072 hypothetical protein
Jamestown00073	PROKKA_00073 Polar organelle development protein
Jamestown00075	PROKKA_00075 hypothetical protein
Jamestown00086	PROKKA_00086 type IV pilus assembly protein PilM
Jamestown00088	PROKKA_00088 hypothetical protein
Jamestown00094	PROKKA_00094 hypothetical protein
Jamestown00116	PROKKA_00116 hypothetical protein
Jamestown00129	PROKKA_00129 hypothetical protein
Jamestown00130	PROKKA_00130 hypothetical protein
Jamestown00134	PROKKA_00134 ornithine cyclodeaminase
Jamestown00144	PROKKA_00144 hypothetical protein
Jamestown00162	PROKKA_00162 Site-specific recombinase XerD
Jamestown00166	PROKKA_00166 hypothetical protein
Jamestown00199	PROKKA_00199 Linear gramicidin synthase subunit B
Jamestown00207	PROKKA_00208 hypothetical protein

Jamestown00219	PROKKA_00220 hypothetical protein
Jamestown00224	PROKKA_00225 hypothetical protein
Jamestown00249	PROKKA_00251 hypothetical protein
Jamestown00271	PROKKA_00273 hypothetical protein
Jamestown00273	PROKKA_00275 acyl-CoA synthetase
Jamestown00278	PROKKA_00280 hypothetical protein
Jamestown00281	PROKKA_00283 hypothetical protein
Jamestown00295	PROKKA_00297 hypothetical protein
Jamestown00304	PROKKA_00306 hypothetical protein
Jamestown00342	PROKKA_00344 hypothetical protein
Jamestown00365	PROKKA_00368 putative efflux pump membrane transporter TtgB
Jamestown00368	PROKKA_00371 hypothetical protein
Jamestown00391	PROKKA_00394 hypothetical protein
Jamestown00393	PROKKA_00396 hypothetical protein
Jamestown00399	PROKKA_00402 hypothetical protein
Jamestown00418	PROKKA_00421 hypothetical protein
Jamestown00423	PROKKA_00426 DNA polymerase V subunit UmuD
Jamestown00448	PROKKA_00451 hypothetical protein
Jamestown00477	PROKKA_00480 Glucose 1-dehydrogenase 1
Jamestown00501	PROKKA_00504 hypothetical protein
Jamestown00507	PROKKA_00510 hypothetical protein
Jamestown00529	PROKKA_00532 hypothetical protein
Jamestown00538	PROKKA_00541 hypothetical protein
Jamestown00542	PROKKA_00545 Multidrug resistance protein MdtF
Jamestown00571	PROKKA_00574 Glucokinase
Jamestown00574	PROKKA_00577 hypothetical protein
Jamestown00592	PROKKA_00595 hypothetical protein
Jamestown00601	PROKKA_00604 hypothetical protein
Jamestown00609	PROKKA_00612 hypothetical protein
Jamestown00639	PROKKA_00642 hypothetical protein

Jamestown00653	PROKKA_00656 hypothetical protein
Jamestown00669	PROKKA_00674 hypothetical protein
Jamestown00678	PROKKA_00683 DNA polymerase V subunit UmuC
Jamestown00681	PROKKA_00686 Poly(A) polymerase I precursor
Jamestown00682	PROKKA_00687 hypothetical protein
Jamestown00689	PROKKA_00694 hypothetical protein
Jamestown00704	PROKKA_00709 TrkA-C domain protein
Jamestown00738	PROKKA_00743 hypothetical protein
Jamestown00745	PROKKA_00750 hypothetical protein
Jamestown00756	PROKKA_00761 hypothetical protein
Jamestown00778	PROKKA_00784 hypothetical protein
Jamestown00792	PROKKA_00798 hypothetical protein
Jamestown00836	PROKKA_00842 hypothetical protein
Jamestown00840	PROKKA_00846 hypothetical protein
Jamestown00845	PROKKA_00851 hypothetical protein
Jamestown00853	PROKKA_00859 hypothetical protein
Jamestown00875	PROKKA_00881 hypothetical protein
Jamestown00876	PROKKA_00882 hypothetical protein
Jamestown00877	PROKKA_00883 putative urate catabolism protein
Jamestown00883	PROKKA_00890 hypothetical protein
Jamestown00899	PROKKA_00906 hypothetical protein
Jamestown00904	PROKKA_00911 hypothetical protein
Jamestown00931	PROKKA_00938 Gluconate 2-dehydrogenase cytochrome c subunit precursor
Jamestown00940	PROKKA_00947 hypothetical protein
Jamestown00947	PROKKA_00954 hypothetical protein
Jamestown00950	PROKKA_00957 hypothetical protein
Jamestown00973	PROKKA_00980 hypothetical protein
Jamestown00975	PROKKA_00982 YhhN-like protein
Jamestown01017	PROKKA_01024 Pentachlorophenol 4-monooxygenase
Jamestown01021	PROKKA_01028 hypothetical protein

Jamestown01024	PROKKA_01031 hypothetical protein
Jamestown01026	PROKKA_01033 hypothetical protein
Jamestown01030	PROKKA_01037 hypothetical protein
Jamestown01034	PROKKA_01041 hypothetical protein
Jamestown01037	PROKKA_01044 Betaine aldehyde dehydrogenase
Jamestown01041	PROKKA_01048 hypothetical protein
Jamestown01045	PROKKA_01052 hypothetical protein
Jamestown01059	PROKKA_01067 hypothetical protein
Jamestown01099	PROKKA_01107 Adaptive-response sensory-kinase SasA
Jamestown01100	PROKKA_01108 hypothetical protein
Jamestown01101	PROKKA_01109 hypothetical protein
Jamestown01104	PROKKA_01112 hypothetical protein
Jamestown01110	PROKKA_01118 hypothetical protein
Jamestown01123	PROKKA_01131 hypothetical protein
Jamestown01131	PROKKA_01139 putative ABC transporter ATP-binding protein
Jamestown01137	PROKKA_01145 Ras family protein
Jamestown01159	PROKKA_01167 hypothetical protein
Jamestown01160	PROKKA_01169 hypothetical protein
Jamestown01194	PROKKA_01207 hypothetical protein
Jamestown01200	PROKKA_01213 putative ATP-dependent helicase DinG
Jamestown01202	PROKKA_01215 hypothetical protein
Jamestown01203	PROKKA_01216 hypothetical protein
Jamestown01204	PROKKA_01217 Serine/threonine-protein kinase PrkC
Jamestown01206	PROKKA_01219 Mlc titration factor A
Jamestown01207	PROKKA_01220 hypothetical protein
Jamestown01208	PROKKA_01221 hypothetical protein
Jamestown01209	PROKKA_01222 hypothetical protein
Jamestown01269	PROKKA_01282 hypothetical protein
Jamestown01312	PROKKA_01325 hypothetical protein
Jamestown01317	PROKKA_01330 Multidrug resistance protein B

Jamestown01347	PROKKA_01360 hypothetical protein
Jamestown01349	PROKKA_01362 prevent-host-death family protein
Jamestown01366	PROKKA_01379 hypothetical protein
Jamestown01433	PROKKA_01455 hypothetical protein
Jamestown01453	PROKKA_01475 hypothetical protein
Jamestown01467	PROKKA_01492 hypothetical protein
Jamestown01470	PROKKA_01495 putative adenylyltransferase/sulfurtransferase MoeZ
Jamestown01472	PROKKA_01497 molybdopterin converting factor, subunit 1
Jamestown01475	PROKKA_01500 hypothetical protein
Jamestown01487	PROKKA_01512 Multiple resistance and pH homeostasis protein D
Jamestown01488	PROKKA_01513 Multiple resistance and pH homeostasis protein D
Jamestown01493	PROKKA_01518 Universal stress protein E
Jamestown01495	PROKKA_01520 Inner membrane protein YbaL
Jamestown01499	PROKKA_01524 putative membrane protein
Jamestown01500	PROKKA_01525 putative membrane protein
Jamestown01501	PROKKA_01526 Cytochrome c oxidase subunit 2 precursor
Jamestown01502	PROKKA_01527 Cytochrome c oxidase subunit 1
Jamestown01503	PROKKA_01528 hypothetical protein
Jamestown01504	PROKKA_01529 cytochrome c oxidase assembly factor CtaG
Jamestown01515	PROKKA_01540 pheromone autoinducer 2 transporter
Jamestown01538	PROKKA_01563 Bacteriophytochrome cph2
Jamestown01539	PROKKA_01564 Cyclic di-GMP phosphodiesterase Gmr
Jamestown01561	PROKKA_01586 hypothetical protein
Jamestown01562	PROKKA_01587 hypothetical protein
Jamestown01571	PROKKA_01596 hypothetical protein
Jamestown01590	PROKKA_01615 4-amino-4-deoxychorismate lyase
Jamestown01593	PROKKA_01618 hypothetical protein
Jamestown01597	PROKKA_01622 hypothetical protein
Jamestown01632	PROKKA_01657 hypothetical protein
Jamestown01635	PROKKA_01660 hypothetical protein

Jamestown01647	PROKKA_01672 hypothetical protein
Jamestown01678	PROKKA_01703 hypothetical protein
Jamestown01689	PROKKA_01714 hypothetical protein
Jamestown01702	PROKKA_01727 hypothetical protein
Jamestown01716	PROKKA_01741 hypothetical protein
Jamestown01728	PROKKA_01754 Gamma-glutamyltranspeptidase precursor
Jamestown01745	PROKKA_01771 hypothetical protein
Jamestown01757	PROKKA_01783 putative protein involved in ubiquinone biosynthesis
Jamestown01758	PROKKA_01784 hypothetical protein
Jamestown01815	PROKKA_01842 hypothetical protein
Jamestown01819	PROKKA_01846 phosphoribosylpyrophosphate synthetase
Jamestown01857	PROKKA_01884 hypothetical protein
Jamestown01874	PROKKA_01901 Pyridoxal 4-dehydrogenase
Jamestown01904	PROKKA_01931 hypothetical protein
Jamestown01922	PROKKA_01949 hypothetical protein
Jamestown01926	PROKKA_01953 hypothetical protein
Jamestown01940	PROKKA_01967 Dipeptide and tripeptide permease A
Jamestown01981	PROKKA_02010 hypothetical protein
Jamestown01997	PROKKA_02026 Bacteriophytochrome cph2
Jamestown01998	PROKKA_02027 Cyclic di-GMP phosphodiesterase Gmr
Jamestown02008	PROKKA_02037 hypothetical protein
Jamestown02015	PROKKA_02044 hypothetical protein
Jamestown02054	PROKKA_02083 sensor protein RstB
Jamestown02071	PROKKA_02100 hypothetical protein
Jamestown02093	PROKKA_02122 hypothetical protein
Jamestown02095	PROKKA_02124 Sodium/proton antiporter NhaA
Jamestown02096	PROKKA_02125 Sodium/proton antiporter NhaA
Jamestown02110	PROKKA_02139 hypothetical protein
Jamestown02135	PROKKA_02164 hypothetical protein
Jamestown02175	PROKKA_02204 hypothetical protein

Jamestown02215	PROKKA_02245 hypothetical protein
Jamestown02239	PROKKA_02269 periplasmic chaperone
Jamestown02249	PROKKA_02280 hypothetical protein
Jamestown02259	PROKKA_02290 hypothetical protein
Jamestown02261	PROKKA_02292 hypothetical protein
Jamestown02262	PROKKA_02293 hypothetical protein
Jamestown02267	PROKKA_02298 hypothetical protein
Jamestown02284	PROKKA_02315 ferrous iron efflux protein F
Jamestown02304	PROKKA_02335 putative N-acetyltransferase YjaB
Jamestown02305	PROKKA_02336 hypothetical protein
Jamestown02308	PROKKA_02339 hypothetical protein
Jamestown02309	PROKKA_02340 Beta-lactamase
Jamestown02310	PROKKA_02341 hypothetical protein
Jamestown02318	PROKKA_02349 O-acetyltransferase OatA
Jamestown02337	PROKKA_02368 Outer membrane protein MIP precursor
Jamestown02351	PROKKA_02383 UDP-N-acetylglucosamine--N-acetylmuramyl-(pentapeptide) pyrophosphoryl-undecaprenol [illegible]
Jamestown02354	PROKKA_02386 hypothetical protein
Jamestown02382	PROKKA_02414 hypothetical protein
Jamestown02392	PROKKA_02424 hypothetical protein
Jamestown02393	PROKKA_02425 hypothetical protein
Jamestown02396	PROKKA_02428 hypothetical protein
Jamestown02403	PROKKA_02435 hypothetical protein
Jamestown02423	PROKKA_02455 hypothetical protein
Jamestown02472	PROKKA_02505 Legionella pneumophila major outer membrane protein precursor
Jamestown02505	PROKKA_02538 hypothetical protein
Jamestown02508	PROKKA_02541 hypothetical protein
Jamestown02552	PROKKA_02585 hypothetical protein
Jamestown02586	PROKKA_02619 molybdopterin converting factor, subunit 1
Jamestown02640	PROKKA_02673 hypothetical protein
Jamestown02642	PROKKA_02675 Heterocyst differentiation ATP-binding protein HepA

Jamestown02652	PROKKA_02685 CAI-1 autoinducer synthase
Jamestown02653	PROKKA_02686 hypothetical protein
Jamestown02658	PROKKA_02691 hypothetical protein
Jamestown02664	PROKKA_02697 RasGEF domain protein
Jamestown02665	PROKKA_02698 hypothetical protein
Jamestown02667	PROKKA_02700 NADH dehydrogenase
Jamestown02678	PROKKA_02711 hypothetical protein
Jamestown02692	PROKKA_02725 Putative Zn-dependent protease, contains TPR repeats
Jamestown02721	PROKKA_02755 hypothetical protein
Jamestown02741	PROKKA_02775 Lysine-specific permease
Jamestown02742	PROKKA_02776 hypothetical protein
Jamestown02749	PROKKA_02783 Soluble lytic murein transglycosylase precursor
Jamestown02764	PROKKA_02798 hypothetical protein
Jamestown02786	PROKKA_02820 hypothetical protein
Jamestown02787	PROKKA_02821 hypothetical protein
Jamestown02788	PROKKA_02822 hypothetical protein
Jamestown02808	PROKKA_02842 hypothetical protein
Jamestown02809	PROKKA_02843 hypothetical protein
Jamestown02811	PROKKA_02845 hypothetical protein
Jamestown02816	PROKKA_02850 homoserine kinase
Jamestown02828	PROKKA_02862 dephospho-CoA kinase/protein folding accessory domain-containing protein
Jamestown02829	PROKKA_02863 Ran GTPase-activating protein (RanGAP) involved in mRNA processing and transport
Jamestown02877	PROKKA_02911 hypothetical protein
Jamestown02878	PROKKA_02912 hypothetical protein
Jamestown02879	PROKKA_02913 Calcineurin-like phosphoesterase
Jamestown02880	PROKKA_02914 hypothetical protein
Jamestown02882	PROKKA_02916 Metal-tetracycline/H(+) antiporter
Jamestown02883	PROKKA_02917 multidrug resistance protein
Jamestown02886	PROKKA_02920 hypothetical protein
Jamestown02887	PROKKA_02921 C signal

Jamestown02890	PROKKA_02924 putative ester cyclase
Jamestown02892	PROKKA_02926 4-hydroxymandelate synthase
Jamestown02897	PROKKA_02931 hypothetical protein
Jamestown02904	PROKKA_02938 hypothetical protein
Jamestown02924	PROKKA_02958 hypothetical protein
Jamestown02928	PROKKA_02962 hypothetical protein
Jamestown02937	PROKKA_02971 hypothetical protein
Jamestown02938	PROKKA_02972 hypothetical protein
Jamestown02939	PROKKA_02973 hypothetical protein
Jamestown02958	PROKKA_02992 hypothetical protein
Jamestown02964	PROKKA_02999 Phenylalanine--tRNA ligase beta subunit
Jamestown02981	PROKKA_03016 Carboxylesterase NlhH
Jamestown02992	PROKKA_03027 hypothetical protein
Jamestown02993	PROKKA_03028 hypothetical protein
Jamestown03006	PROKKA_03041 hypothetical protein
Jamestown03008	PROKKA_03043 hypothetical protein
Jamestown03011	PROKKA_03046 hypothetical protein
Jamestown03021	PROKKA_03056 putative transporter
Jamestown03024	PROKKA_03059 hypothetical protein
Jamestown03046	PROKKA_03081 Synaptobrevin
Jamestown03051	PROKKA_03086 Phosphomannomutase/phosphoglucomutase
Jamestown03071	PROKKA_03106 hypothetical protein
Jamestown03072	PROKKA_03107 hypothetical protein
Jamestown03073	PROKKA_03108 hypothetical protein
Jamestown03075	PROKKA_03110 Carbon storage regulator
Jamestown03079	PROKKA_03114 hypothetical protein
Jamestown03096	PROKKA_03131 hypothetical protein
Jamestown03097	PROKKA_03132 hypothetical protein
Jamestown03098	PROKKA_03133 hypothetical protein
Jamestown03099	PROKKA_03134 hypothetical protein

Jamestown03102	PROKKA_03137 hypothetical protein
Jamestown03104	PROKKA_03139 Transposase, IS30 family
Jamestown03106	PROKKA_03141 hypothetical protein
Jamestown03120	PROKKA_03155 hypothetical protein
Jamestown03122	PROKKA_03157 hypothetical protein
Jamestown03123	PROKKA_03158 DNA polymerase V subunit UmuD
Jamestown03124	PROKKA_03159 DNA polymerase V subunit UmuC
Jamestown03125	PROKKA_03160 hypothetical protein
Jamestown03136	PROKKA_03171 Opacity protein antigens
Jamestown03137	PROKKA_03172 hypothetical protein
Jamestown03138	PROKKA_03173 hypothetical protein
Jamestown03143	PROKKA_03178 hypothetical protein
Jamestown03163	PROKKA_03198 hypothetical protein
Jamestown03167	PROKKA_03202 hypothetical protein
Jamestown03173	PROKKA_03208 Alanine--tRNA ligase
Jamestown03183	PROKKA_03218 Autotransporter assembly factor TamA
Jamestown03189	PROKKA_03224 hypothetical protein
Jamestown03206	PROKKA_03241 H(+)/Cl(-) exchange transporter ClcA
Jamestown03237	PROKKA_03272 hypothetical protein
Jamestown03246	PROKKA_03281 hypothetical protein
Jamestown03252	PROKKA_03287 hypothetical protein
Jamestown03268	PROKKA_03304 DNA adenine methylase
Jamestown03273	PROKKA_03309 potassium transport protein Kup
Jamestown03279	PROKKA_03315 Tyrosine--tRNA ligase
Jamestown03281	PROKKA_03317 hypothetical protein
Jamestown03282	PROKKA_03318 Phytanoyl-CoA dioxygenase (PhyH)
Jamestown03291	PROKKA_03327 hypothetical protein
Jamestown03294	PROKKA_03330 hypothetical protein
Jamestown03295	PROKKA_03331 hypothetical protein
Jamestown03304	PROKKA_03341 hypothetical protein

Jamestown03307	PROKKA_03344 Putative molybdenum carrier
Jamestown03311	PROKKA_03348 hypothetical protein
Jamestown03312	PROKKA_03349 hypothetical protein
Jamestown03313	PROKKA_03350 hypothetical protein
Jamestown03315	PROKKA_03352 hypothetical protein
Jamestown03316	PROKKA_03353 hypothetical protein
Jamestown03320	PROKKA_03357 hypothetical protein
Jamestown03322	PROKKA_03359 hypothetical protein
Jordan00020	Jordan_00020 hypothetical protein
Jordan00095	Jordan_00095 hypothetical protein
Jordan00100	Jordan_00100 hypothetical protein
Jordan00127	Jordan_00127 hypothetical protein
Jordan00134	Jordan_00134 Inosine-uridine preferring nucleoside hydrolase
Jordan00175	Jordan_00175 hypothetical protein
Jordan00193	Jordan_00193 hypothetical protein
Jordan00226	Jordan_00226 hypothetical protein
Jordan00242	Jordan_00242 hypothetical protein
Jordan00280	Jordan_00283 hypothetical protein
Jordan00282	Jordan_00285 hypothetical protein
Jordan00295	Jordan_00298 hypothetical protein
Jordan00320	Jordan_00329 ankyrin repeat protein
Jordan00339	Jordan_00348 hypothetical protein
Jordan00356	Jordan_00365 hypothetical protein
Jordan00391	Jordan_00401 hypothetical protein
Jordan00532	Jordan_00543 hypothetical protein
Jordan00536	Jordan_00547 hypothetical protein
Jordan00569	Jordan_00581 hypothetical protein
Jordan00649	Jordan_00661 hypothetical protein
Jordan00759	Jordan_00772 hypothetical protein
Jordan00819	Jordan_00834 hypothetical protein

Jordan00828	Jordan_00843 hypothetical protein
Jordan00829	Jordan_00844 hypothetical protein
Jordan00831	Jordan_00846 hypothetical protein
Jordan00866	Jordan_00881 hypothetical protein
Jordan00873	Jordan_00888 hypothetical protein
Jordan00879	Jordan_00894 Alpha/beta hydrolase family protein
Jordan00894	Jordan_00909 hypothetical protein
Jordan00895	Jordan_00910 hypothetical protein
Jordan00900	Jordan_00915 RNase III inhibitor
Jordan00901	Jordan_00916 hypothetical protein
Jordan00904	Jordan_00919 hypothetical protein
Jordan00906	Jordan_00921 hypothetical protein
Jordan00910	Jordan_00925 hypothetical protein
Jordan00914	Jordan_00929 hypothetical protein
Jordan00915	Jordan_00930 Cyclic di-GMP phosphodiesterase Gmr
Jordan00916	Jordan_00931 hypothetical protein
Jordan00921	Jordan_00936 hypothetical protein
Jordan00964	Jordan_00979 Mycinamicin III 3"-O-methyltransferase
Jordan00997	Jordan_01012 hypothetical protein
Jordan01055	Jordan_01070 hypothetical protein
Jordan01095	Jordan_01110 hypothetical protein
Jordan01098	Jordan_01114 hypothetical protein
Jordan01115	Jordan_01131 hypothetical protein
Jordan01130	Jordan_01146 hypothetical protein
Jordan01133	Jordan_01149 D-arabitol-phosphate dehydrogenase
Jordan01142	Jordan_01158 Polysaccharide pyruvyl transferase
Jordan01143	Jordan_01159 coenzyme F420-reducing hydrogenase subunit beta
Jordan01144	Jordan_01160 Beta-xylosidase
Jordan01145	Jordan_01161 Phosphoserine phosphatase 1
Jordan01146	Jordan_01162 inositol 1-phosphate synthase

Jordan01147	Jordan_01163 hypothetical protein
Jordan01148	Jordan_01164 hypothetical protein
Jordan01160	Jordan_01177 putative membrane protein
Jordan01161	Jordan_01178 hypothetical protein
Jordan01174	Jordan_01191 hypothetical protein
Jordan01180	Jordan_01197 hypothetical protein
Jordan01203	Jordan_01220 hypothetical protein
Jordan01219	Jordan_01236 hypothetical protein
Jordan01231	Jordan_01248 hypothetical protein
Jordan01245	Jordan_01262 conjugal transfer relaxase TraI
Jordan01248	Jordan_01265 hypothetical protein
Jordan01252	Jordan_01269 hypothetical protein
Jordan01269	Jordan_01286 hypothetical protein
Jordan01297	Jordan_01314 hypothetical protein
Jordan01301	Jordan_01318 hypothetical protein
Jordan01326	Jordan_01343 hypothetical protein
Jordan01332	Jordan_01349 hypothetical protein
Jordan01340	Jordan_01358 hypothetical protein
Jordan01343	Jordan_01361 hypothetical protein
Jordan01344	Jordan_01362 hypothetical protein
Jordan01345	Jordan_01363 hypothetical protein
Jordan01348	Jordan_01366 hypothetical protein
Jordan01360	Jordan_01378 hypothetical protein
Jordan01370	Jordan_01388 hypothetical protein
Jordan01371	Jordan_01389 Putative DNA ligase-like protein/MT0965
Jordan01386	Jordan_01404 hypothetical protein
Jordan01389	Jordan_01407 hypothetical protein
Jordan01390	Jordan_01408 hypothetical protein
Jordan01391	Jordan_01409 hypothetical protein
Jordan01394	Jordan_01412 hypothetical protein

Jordan01395	Jordan_01413 hypothetical protein
Jordan01400	Jordan_01418 site-specific tyrosine recombinase XerC
Jordan01401	Jordan_01419 hypothetical protein
Jordan01402	Jordan_01420 hypothetical protein
Jordan01403	Jordan_01421 hypothetical protein
Jordan01411	Jordan_01429 hypothetical protein
Jordan01424	Jordan_01443 hypothetical protein
Jordan01457	Jordan_01476 hypothetical protein
Jordan01458	Jordan_01477 PAP2 superfamily protein
Jordan01474	Jordan_01493 hypothetical protein
Jordan01571	Jordan_01592 hypothetical protein
Jordan01572	Jordan_01593 hypothetical protein
Jordan01597	Jordan_01618 hypothetical protein
Jordan01598	Jordan_01619 hypothetical protein
Jordan01632	Jordan_01653 hypothetical protein
Jordan01633	Jordan_01654 hypothetical protein
Jordan01634	Jordan_01655 hypothetical protein
Jordan01637	Jordan_01658 hypothetical protein
Jordan01673	Jordan_01694 hypothetical protein
Jordan01677	Jordan_01698 hypothetical protein
Jordan01682	Jordan_01703 trans-aconitate 2-methyltransferase
Jordan01683	Jordan_01704 hypothetical protein
Jordan01686	Jordan_01707 hypothetical protein
Jordan01687	Jordan_01708 His-Xaa-Ser repeat protein HxsA
Jordan01688	Jordan_01709 His-Xaa-Ser system protein HsxD
Jordan01689	Jordan_01710 hypothetical protein
Jordan01690	Jordan_01711 molybdenum cofactor biosynthesis protein A
Jordan01729	Jordan_01750 hypothetical protein
Jordan01748	Jordan_01769 putative acetyltransferase
Jordan01749	Jordan_01770 hypothetical protein

Jordan01812	Jordan_01833 Multidrug transporter MdtC
Jordan01819	Jordan_01840 hypothetical protein
Jordan01868	Jordan_01889 hypothetical protein
Jordan01949	Jordan_01971 hypothetical protein
Jordan01955	Jordan_01977 hypothetical protein
Jordan01969	Jordan_01991 Isoaspartyl peptidase precursor
Jordan01981	Jordan_02003 hypothetical protein
Jordan01985	Jordan_02007 hypothetical protein
Jordan01990	Jordan_02012 DnaK suppressor protein
Jordan02000	Jordan_02022 hypothetical protein
Jordan02001	Jordan_02023 hypothetical protein
Jordan02003	Jordan_02025 hypothetical protein
Jordan02018	Jordan_02041 hypothetical protein
Jordan02022	Jordan_02045 hypothetical protein
Jordan02150	Jordan_02173 hypothetical protein
Jordan02180	Jordan_02203 HNH endonuclease
Jordan02181	Jordan_02204 hypothetical protein
Jordan02187	Jordan_02210 hypothetical protein
Jordan02192	Jordan_02215 hypothetical protein
Jordan02193	Jordan_02216 hypothetical protein
Jordan02195	Jordan_02218 hypothetical protein
Jordan02196	Jordan_02219 hypothetical protein
Jordan02198	Jordan_02221 hypothetical protein
Jordan02202	Jordan_02225 hypothetical protein
Jordan02214	Jordan_02238 hypothetical protein
Jordan02218	Jordan_02242 hypothetical protein
Jordan02221	Jordan_02245 hypothetical protein
Jordan02228	Jordan_02252 hypothetical protein
Jordan02296	Jordan_02320 hypothetical protein
Jordan02311	Jordan_02335 hypothetical protein

Jordan02333	Jordan_02357 hypothetical protein
Jordan02337	Jordan_02361 3-ketoacyl-CoA thiolase
Jordan02345	Jordan_02369 putative membrane protein
Jordan02366	Jordan_02390 hypothetical protein
Jordan02372	Jordan_02396 hypothetical protein
Jordan02380	Jordan_02407 hypothetical protein
Jordan02381	Jordan_02408 hypothetical protein
Jordan02384	Jordan_02411 hypothetical protein
Jordan02385	Jordan_02412 hypothetical protein
Jordan02389	Jordan_02416 hypothetical protein
Jordan02431	Jordan_02458 hypothetical protein
Jordan02446	Jordan_02473 putative enzyme related to lactoylglutathione lyase
Jordan02447	Jordan_02474 HIT-like protein
Jordan02508	Jordan_02537 hypothetical protein
Jordan02527	Jordan_02556 hypothetical protein
Jordan02537	Jordan_02566 hypothetical protein
Jordan02546	Jordan_02575 hypothetical protein
Jordan02592	Jordan_02621 hypothetical protein
Jordan02593	Jordan_02622 hypothetical protein
Jordan02596	Jordan_02625 hypothetical protein
Jordan02598	Jordan_02627 hypothetical protein
Jordan02608	Jordan_02642 hypothetical protein
Jordan02615	Jordan_02649 hypothetical protein
Jordan02646	Jordan_02680 hypothetical protein
Jordan02689	Jordan_02724 hypothetical protein
Jordan02690	Jordan_02725 hypothetical protein
Jordan02719	Jordan_02754 hypothetical protein
Jordan02720	Jordan_02755 hypothetical protein
Jordan02728	Jordan_02763 hypothetical protein
Jordan02748	Jordan_02783 lipoprotein releasing system, transmembrane protein, LolC/E family

Jordan02770	Jordan_02806 hypothetical protein
Jordan02773	Jordan_02809 hypothetical protein
Jordan02785	Jordan_02821 hypothetical protein
Jordan02789	Jordan_02826 dephospho-CoA kinase/protein folding accessory domain-containing protein
Jordan02792	Jordan_02829 hypothetical protein
Jordan02794	Jordan_02831 hypothetical protein
Jordan02796	Jordan_02833 hypothetical protein
Jordan02801	Jordan_02838 hypothetical protein
Jordan02802	Jordan_02839 hypothetical protein
Jordan02803	Jordan_02840 hypothetical protein
Jordan02813	Jordan_02850 hypothetical protein
Jordan02839	Jordan_02876 hypothetical protein
Jordan02854	Jordan_02891 hypothetical protein
Jordan02855	Jordan_02892 hypothetical protein
Jordan02857	Jordan_02894 Cupin domain protein
Jordan02864	Jordan_02901 topology modulation protein
Jordan02893	Jordan_02930 hypothetical protein
Jordan02938	Jordan_02975 hypothetical protein
Jordan02945	Jordan_02982 putative protein tyrosine phosphatase
Jordan02946	Jordan_02983 hypothetical protein
Jordan02947	Jordan_02984 hypothetical protein
Jordan02948	Jordan_02985 hypothetical protein
Jordan02950	Jordan_02987 hypothetical protein
Jordan02960	Jordan_02997 hypothetical protein
Jordan02961	Jordan_02998 hypothetical protein
mac00001	mac_D5800_00001 hypothetical protein
mac00003	mac_D5800_00003 hypothetical protein
mac00004	mac_D5800_00004 hypothetical protein
mac00005	mac_D5800_00005 hypothetical protein
mac00025	mac_D5800_00025 hypothetical protein

mac00042	mac_D5800_00042 hypothetical protein
mac00059	mac_D5800_00059 hypothetical protein
mac00065	mac_D5800_00065 glutathione synthetase
mac00092	mac_D5800_00095 hypothetical protein
mac00134	mac_D5800_00143 hypothetical protein
mac00140	mac_D5800_00149 hypothetical protein
mac00146	mac_D5800_00155 hypothetical protein
mac00152	mac_D5800_00161 hypothetical protein
mac00153	mac_D5800_00162 hypothetical protein
mac00165	mac_D5800_00174 Patatin-like phospholipase
mac00166	mac_D5800_00175 Patatin phospholipase
mac00184	mac_D5800_00193 hypothetical protein
mac00185	mac_D5800_00194 polysaccharide deacetylase family sporulation protein PdaB
mac00191	mac_D5800_00200 Lon protease
mac00192	mac_D5800_00201 Hsp20/alpha crystallin family protein
mac00200	mac_D5800_00209 hypothetical protein
mac00202	mac_D5800_00211 DNA alkylation repair enzyme
mac00206	mac_D5800_00215 hypothetical protein
mac00207	mac_D5800_00216 hypothetical protein
mac00208	mac_D5800_00217 hypothetical protein
mac00209	mac_D5800_00218 hypothetical protein
mac00210	mac_D5800_00219 hypothetical protein
mac00212	mac_D5800_00221 hypothetical protein
mac00214	mac_D5800_00223 hypothetical protein
mac00215	mac_D5800_00224 hypothetical protein
mac00218	mac_D5800_00227 DsrE/DsrF-like family protein
mac00221	mac_D5800_00230 hypothetical protein
mac00223	mac_D5800_00232 hypothetical protein
mac00224	mac_D5800_00233 Transposase
mac00237	mac_D5800_00246 hypothetical protein

mac00238	mac_D5800_00247 hypothetical protein
mac00239	mac_D5800_00248 hypothetical protein
mac00240	mac_D5800_00249 hypothetical protein
mac00249	mac_D5800_00258 Response regulator ArlR
mac00253	mac_D5800_00262 hypothetical protein
mac00254	mac_D5800_00263 hypothetical protein
mac00261	mac_D5800_00270 hypothetical protein
mac00262	mac_D5800_00271 hypothetical protein
mac00272	mac_D5800_00281 peptidase PmbA
mac00288	mac_D5800_00297 hypothetical protein
mac00293	mac_D5800_00302 hypothetical protein
mac00295	mac_D5800_00304 Karyopherin (importin) beta
mac00297	mac_D5800_00306 hypothetical protein
mac00300	mac_D5800_00309 Calcineurin-like phosphoesterase
mac00304	mac_D5800_00313 hypothetical protein
mac00308	mac_D5800_00317 hypothetical protein
mac00318	mac_D5800_00327 Putative Rieske 2Fe-2S iron-sulfur protein/MSMEI_6242
mac00325	mac_D5800_00334 hypothetical protein
mac00328	mac_D5800_00337 extracellular protein
mac00356	mac_D5800_00365 spermidine synthase
mac00369	mac_D5800_00378 Membrane-bound lytic murein transglycosylase B precursor
mac00419	mac_D5800_00431 hypothetical protein
mac00420	mac_D5800_00432 hypothetical protein
mac00421	mac_D5800_00433 hypothetical protein
mac00424	mac_D5800_00436 hypothetical protein
mac00459	mac_D5800_00472 hypothetical protein
mac00503	mac_D5800_00516 hypothetical protein
mac00510	mac_D5800_00523 hypothetical protein
mac00523	mac_D5800_00536 hypothetical protein
mac00527	mac_D5800_00540 Undecaprenyl phosphate-alpha-4-amino-4-deoxy-L-arabinose arabinosyl transferase

mac00529	mac_D5800_00542 hypothetical protein
mac00530	mac_D5800_00543 hypothetical protein
mac00531	mac_D5800_00544 Chaperone protein DnaJ
mac00535	mac_D5800_00548 hypothetical protein
mac00573	mac_D5800_00587 hypothetical protein
mac00577	mac_D5800_00591 O-antigen translocase
mac00603	mac_D5800_00617 hypothetical protein
mac00614	mac_D5800_00628 hypothetical protein
mac00629	mac_D5800_00644 hypothetical protein
mac00630	mac_D5800_00645 hypothetical protein
mac00632	mac_D5800_00647 hypothetical protein
mac00633	mac_D5800_00648 hypothetical protein
mac00639	mac_D5800_00654 Transposase
mac00653	mac_D5800_00668 hypothetical protein
mac00715	mac_D5800_00730 Sensor kinase protein RcsC
mac00718	mac_D5800_00733 hypothetical protein
mac00719	mac_D5800_00734 hypothetical protein
mac00752	mac_D5800_00767 hypothetical protein
mac00755	mac_D5800_00770 hypothetical protein
mac00799	mac_D5800_00814 hypothetical protein
mac00861	mac_D5800_00876 hypothetical protein
mac00864	mac_D5800_00879 Glycerol kinase
mac00921	mac_D5800_00937 Bifunctional protein PutA
mac00924	mac_D5800_00940 3-demethylubiquinone-9 3-methyltransferase
mac00932	mac_D5800_00948 hypothetical protein
mac00975	mac_D5800_00992 hypothetical protein
mac00998	mac_D5800_01016 hypothetical protein
mac01010	mac_D5800_01028 hypothetical protein
mac01011	mac_D5800_01029 hypothetical protein
mac01012	mac_D5800_01030 hypothetical protein

mac01014	mac_D5800_01032 hypothetical protein
mac01015	mac_D5800_01033 3-octaprenyl-4-hydroxybenzoate carboxy-lyase
mac01016	mac_D5800_01034 hypothetical protein
mac01018	mac_D5800_01036 Phenolic acid decarboxylase subunit B
mac01026	mac_D5800_01044 Cation efflux system protein CusA
mac01086	mac_D5800_01106 D,D-heptose 1,7-bisphosphate phosphatase
mac01087	mac_D5800_01107 SNARE associated Golgi protein
mac01090	mac_D5800_01110 Ferredoxin fas2
mac01091	mac_D5800_01111 1-deoxy-D-xylulose-5-phosphate synthase
mac01093	mac_D5800_01113 Glucose-1-phosphate cytidylyltransferase
mac01094	mac_D5800_01114 Phosphoheptose isomerase
mac01102	mac_D5800_01122 hypothetical protein
mac01103	mac_D5800_01123 hypothetical protein
mac01104	mac_D5800_01124 hypothetical protein
mac01137	mac_D5800_01157 hypothetical protein
mac01146	mac_D5800_01166 Ribonuclease R
mac01149	mac_D5800_01169 Superoxide dismutase [Fe]
mac01171	mac_D5800_01191 Multidrug transporter MdtC
mac01172	mac_D5800_01192 Multidrug transporter MdtC
mac01174	mac_D5800_01194 hypothetical protein
mac01188	mac_D5800_01208 hypothetical protein
mac01194	mac_D5800_01214 hypothetical protein
mac01195	mac_D5800_01215 hypothetical protein
mac01198	mac_D5800_01218 hypothetical protein
mac01200	mac_D5800_01220 hypothetical protein
mac01201	mac_D5800_01221 hypothetical protein
mac01216	mac_D5800_01236 hypothetical protein
mac01218	mac_D5800_01238 hypothetical protein
mac01232	mac_D5800_01252 Recombination-associated protein RdgC
mac01246	mac_D5800_01267 hypothetical protein

mac01266	mac_D5800_01287 hypothetical protein
mac01280	mac_D5800_01301 transferase 2, rSAM/selenodomain-associated
mac01291	mac_D5800_01312 Polyketide synthase PksJ
mac01294	mac_D5800_01315 hypothetical protein
mac01300	mac_D5800_01321 hypothetical protein
mac01301	mac_D5800_01322 Site-specific recombinase XerD
mac01305	mac_D5800_01326 hypothetical protein
mac01306	mac_D5800_01327 hypothetical protein
mac01307	mac_D5800_01328 hypothetical protein
mac01308	mac_D5800_01329 hypothetical protein
mac01313	mac_D5800_01334 hypothetical protein
mac01315	mac_D5800_01336 hypothetical protein
mac01321	mac_D5800_01342 glycine oxidase ThiO
mac01336	mac_D5800_01357 putative amino acid permease YhdG
mac01350	mac_D5800_01371 hypothetical protein
mac01360	mac_D5800_01381 hypothetical protein
mac01361	mac_D5800_01382 hypothetical protein
mac01362	mac_D5800_01383 hypothetical protein
mac01363	mac_D5800_01384 hypothetical protein
mac01397	mac_D5800_01418 hypothetical protein
mac01399	mac_D5800_01420 hypothetical protein
mac01400	mac_D5800_01421 4-oxalocrotonate tautomerase
mac01404	mac_D5800_01425 hypothetical protein
mac01407	mac_D5800_01428 hypothetical protein
mac01412	mac_D5800_01434 hypothetical protein
mac01417	mac_D5800_01439 hypothetical protein
mac01420	mac_D5800_01442 hypothetical protein
mac01421	mac_D5800_01443 hypothetical protein
mac01435	mac_D5800_01457 hypothetical protein
mac01437	mac_D5800_01459 hypothetical protein

mac01442	mac_D5800_01464 hypothetical protein
mac01452	mac_D5800_01474 hypothetical protein
mac01455	mac_D5800_01477 ATP-dependent DNA ligase
mac01464	mac_D5800_01486 Karyopherin (importin) beta
mac01465	mac_D5800_01487 Karyopherin (importin) beta
mac01473	mac_D5800_01495 Transposase
mac01475	mac_D5800_01497 hypothetical protein
mac01476	mac_D5800_01498 hypothetical protein
mac01480	mac_D5800_01502 hypothetical protein
mac01483	mac_D5800_01505 hypothetical protein
mac01487	mac_D5800_01509 hypothetical protein
mac01492	mac_D5800_01514 hypothetical protein
mac01520	mac_D5800_01542 Polyphosphate kinase
mac01525	mac_D5800_01547 hypothetical protein
mac01526	mac_D5800_01548 hypothetical protein
mac01527	mac_D5800_01549 hypothetical protein
mac01529	mac_D5800_01551 hypothetical protein
mac01545	mac_D5800_01567 hypothetical protein
mac01561	mac_D5800_01583 Fumarate hydratase class II
mac01563	mac_D5800_01585 hypothetical protein
mac01597	mac_D5800_01619 hypothetical protein
mac01615	mac_D5800_01638 hypothetical protein
mac01646	mac_D5800_01669 hypothetical protein
mac01655	mac_D5800_01678 hypothetical protein
mac01659	mac_D5800_01682 Apolipoprotein N-acyltransferase
mac01662	mac_D5800_01685 hypothetical protein
mac01674	mac_D5800_01697 Mercuric reductase
mac01676	mac_D5800_01699 hypothetical protein
mac01685	mac_D5800_01708 preprotein translocase subunit SecA
mac01688	mac_D5800_01711 hypothetical protein

mac01706	mac_D5800_01729 hypothetical protein
mac01722	mac_D5800_01745 Karyopherin (importin) beta
mac01723	mac_D5800_01746 hypothetical protein
mac01724	mac_D5800_01747 Karyopherin (importin) beta
mac01726	mac_D5800_01749 hypothetical protein
mac01738	mac_D5800_01761 Nicotinamide mononucleotide transporter
mac01760	mac_D5800_01783 hypothetical protein
mac01761	mac_D5800_01784 hypothetical protein
mac01762	mac_D5800_01785 hypothetical protein
mac01763	mac_D5800_01786 Patatin-like phospholipase
mac01766	mac_D5800_01789 hypothetical protein
mac01771	mac_D5800_01794 hypothetical protein
mac01775	mac_D5800_01798 hypothetical protein
mac01776	mac_D5800_01799 hypothetical protein
mac01777	mac_D5800_01800 hypothetical protein
mac01778	mac_D5800_01801 preprotein translocase subunit SecA
mac01790	mac_D5800_01816 diphosphomevalonate decarboxylase
mac01801	mac_D5800_01827 hypothetical protein
mac01842	mac_D5800_01868 hypothetical protein
mac01843	mac_D5800_01869 hypothetical protein
mac01877	mac_D5800_01904 hypothetical protein
mac01894	mac_D5800_01921 Homogentisate 1,2-dioxygenase
mac01911	mac_D5800_01938 Beta(12)glucan export ATP-binding/permease protein NdvA
mac01919	mac_D5800_01946 hypothetical protein
mac01925	mac_D5800_01952 putative glutamine amidotransferase
mac01931	mac_D5800_01958 hypothetical protein
mac01935	mac_D5800_01962 hypothetical protein
mac01938	mac_D5800_01965 Catalase-peroxidase 2 precursor
mac01948	mac_D5800_01975 hypothetical protein
mac01951	mac_D5800_01978 Trafficking protein A

mac01953	mac_D5800_01980 hypothetical protein
mac01954	mac_D5800_01981 hypothetical protein
mac01955	mac_D5800_01982 hypothetical protein
mac01956	mac_D5800_01983 hypothetical protein
mac01959	mac_D5800_01986 hypothetical protein
mac01960	mac_D5800_01987 hypothetical protein
mac01966	mac_D5800_01993 Dimodular nonribosomal peptide synthase
mac01968	mac_D5800_01995 Beta-ketoacyl-acyl-carrier-protein synthase I
mac01970	mac_D5800_01997 hypothetical protein
mac01973	mac_D5800_02000 hypothetical protein
mac01983	mac_D5800_02010 hypothetical protein
mac02019	mac_D5800_02046 hypothetical protein
mac02021	mac_D5800_02048 hypothetical protein
mac02084	mac_D5800_02111 Cyclopropane-fatty-acyl-phospholipid synthase
mac02107	mac_D5800_02134 hypothetical protein
mac02124	mac_D5800_02151 hypothetical protein
mac02125	mac_D5800_02152 hypothetical protein
mac02132	mac_D5800_02159 hypothetical protein
mac02149	mac_D5800_02176 hypothetical protein
mac02173	mac_D5800_02201 hypothetical protein
mac02174	mac_D5800_02202 hypothetical protein
mac02193	mac_D5800_02223 hypothetical protein
mac02196	mac_D5800_02226 hypothetical protein
mac02197	mac_D5800_02227 Abortive infection bacteriophage resistance protein
mac02199	mac_D5800_02229 hypothetical protein
mac02202	mac_D5800_02232 hypothetical protein
mac02203	mac_D5800_02233 hypothetical protein
mac02204	mac_D5800_02234 hypothetical protein
mac02206	mac_D5800_02236 hypothetical protein
mac02210	mac_D5800_02240 hypothetical protein

mac02212	mac_D5800_02242 hypothetical protein
mac02215	mac_D5800_02245 hypothetical protein
mac02218	mac_D5800_02248 hypothetical protein
mac02220	mac_D5800_02250 hypothetical protein
mac02235	mac_D5800_02265 cadmium-translocating P-type ATPase
mac02237	mac_D5800_02267 Universal stress protein family protein
mac02246	mac_D5800_02277 hypothetical protein
mac02247	mac_D5800_02278 Beta-phosphoglucomutase
mac02249	mac_D5800_02280 putative glycosyl hydrolase/MT2062
mac02251	mac_D5800_02282 Glucose-6-phosphate isomerase
mac02254	mac_D5800_02285 hypothetical protein
mac02256	mac_D5800_02287 hypothetical protein
mac02276	mac_D5800_02307 hypothetical protein
mac02313	mac_D5800_02344 hypothetical protein
mac02340	mac_D5800_02372 Sensor protein FixL
mac02354	mac_D5800_02386 Cytochrome d ubiquinol oxidase subunit 1
mac02369	mac_D5800_02401 hypothetical protein
mac02372	mac_D5800_02404 hypothetical protein
mac02375	mac_D5800_02407 hypothetical protein
mac02376	mac_D5800_02408 hypothetical protein
mac02377	mac_D5800_02409 hypothetical protein
mac02379	mac_D5800_02411 Ribosomal protein L16/L10E
mac02381	mac_D5800_02413 hypothetical protein
mac02395	mac_D5800_02428 hypothetical protein
mac02412	mac_D5800_02445 hypothetical protein
mac02418	mac_D5800_02452 hypothetical protein
mac02419	mac_D5800_02453 hypothetical protein
mac02420	mac_D5800_02454 hypothetical protein
mac02422	mac_D5800_02456 hypothetical protein
mac02423	mac_D5800_02457 hypothetical protein

mac02433	mac_D5800_02467 putative cysteine desulfurase
mac02439	mac_D5800_02473 hypothetical protein
mac02446	mac_D5800_02480 hypothetical protein
mac02466	mac_D5800_02500 hypothetical protein
mac02467	mac_D5800_02501 hypothetical protein
mac02481	mac_D5800_02515 hypothetical protein
mac02487	mac_D5800_02521 hypothetical protein
mac02495	mac_D5800_02529 coproporphyrinogen III oxidase
mac02496	mac_D5800_02530 S-methyl-5'-thioadenosine phosphorylase
mac02499	mac_D5800_02533 Methylthioribose-1-phosphate isomerase
mac02500	mac_D5800_02534 S-adenosylmethionine synthase
mac02544	mac_D5800_02578 Ankyrin repeats (3 copies)
mac02546	mac_D5800_02580 hypothetical protein
mac02547	mac_D5800_02581 hypothetical protein
mac02548	mac_D5800_02582 hypothetical protein
mac02550	mac_D5800_02584 hypothetical protein
mac02551	mac_D5800_02585 Virulence sensor protein BvgS precursor
mac02552	mac_D5800_02586 Sensory/regulatory protein RpfC
mac02553	mac_D5800_02587 hypothetical protein
mac02557	mac_D5800_02591 hypothetical protein
mac02558	mac_D5800_02592 hypothetical protein
mac02559	mac_D5800_02593 hypothetical protein
mac02574	mac_D5800_02608 PAP2 superfamily protein
mac02599	mac_D5800_02633 Urocanate hydratase
mac02624	mac_D5800_02658 hypothetical protein
mac02650	mac_D5800_02684 hypothetical protein
mac02711	mac_D5800_02745 putative oxidoreductase/HEAT repeat-containing protein
mac02720	mac_D5800_02755 hypothetical protein
mac02723	mac_D5800_02758 hypothetical protein
mac02728	mac_D5800_02763 hypothetical protein

mac02733	mac_D5800_02768 hypothetical protein
mac02745	mac_D5800_02780 hypothetical protein
mac02747	mac_D5800_02782 hypothetical protein
mac02748	mac_D5800_02783 prevent-host-death family protein
mac02750	mac_D5800_02785 homoserine kinase
mac02754	mac_D5800_02789 hypothetical protein
mac02755	mac_D5800_02790 L-glyceraldehyde 3-phosphate reductase
mac02758	mac_D5800_02793 hypothetical protein
mac02760	mac_D5800_02795 Sensor kinase protein RcsC
mac02763	mac_D5800_02798 hypothetical protein
mac02767	mac_D5800_02802 hypothetical protein
mac02770	mac_D5800_02805 Multifunctional conjugation protein TraI
mac02772	mac_D5800_02807 hypothetical protein
mac02790	mac_D5800_02825 hypothetical protein
mac02791	mac_D5800_02826 hypothetical protein
mac02792	mac_D5800_02827 hypothetical protein
mac02793	mac_D5800_02828 hypothetical protein
mac02794	mac_D5800_02829 hypothetical protein
mac02795	mac_D5800_02830 hypothetical protein
mac02796	mac_D5800_02831 hypothetical protein
mac02797	mac_D5800_02832 hypothetical protein
mac02798	mac_D5800_02833 ParB-like nuclease domain protein
mac02815	mac_D5800_02850 hypothetical protein
mac02816	mac_D5800_02851 hypothetical protein
mac02822	mac_D5800_02857 CRISPR-associated nuclease/helicase Cas3 subtype I-F/YPEST
mac02825	mac_D5800_02860 hypothetical protein
mac02840	mac_D5800_02875 hypothetical protein
mac02928	mac_D5800_02963 hypothetical protein
mac02961	mac_D5800_02996 hypothetical protein
mac02977	mac_D5800_03012 2'-5' RNA ligase

mac02985	mac_D5800_03020 hypothetical protein
mac02990	mac_D5800_03025 hypothetical protein
mac03002	mac_D5800_03037 hypothetical protein
mac03013	mac_D5800_03048 hypothetical protein
mac03038	mac_D5800_03073 hypothetical protein
mac03045	mac_D5800_03080 hypothetical protein
mac03046	mac_D5800_03081 hypothetical protein
mac03047	mac_D5800_03082 hypothetical protein
mac03064	mac_D5800_03099 hypothetical protein
mac03069	mac_D5800_03104 ankyrin repeat protein
mac03092	mac_D5800_03128 Beta-hexosaminidase A precursor
mac03097	mac_D5800_03133 hypothetical protein
mac03113	mac_D5800_03149 hypothetical protein
mac03120	mac_D5800_03156 hypothetical protein
mac03121	mac_D5800_03157 hypothetical protein
mac03127	mac_D5800_03163 Acetyl-/propionyl-coenzyme A carboxylase alpha chain
mac03134	mac_D5800_03170 arylformamidase
mac03136	mac_D5800_03172 hypothetical protein
mac03137	mac_D5800_03173 hypothetical protein
mac03142	mac_D5800_03178 hypothetical protein
mac03152	mac_D5800_03188 hypothetical protein
mac03153	mac_D5800_03189 hypothetical protein
mac03171	mac_D5800_03207 putative inner membrane transporter yiJE
mac03177	mac_D5800_03213 hypothetical protein
mac03185	mac_D5800_03221 conjugative transfer region lipoprotein
mac03187	mac_D5800_03223 hypothetical protein
mac03211	mac_D5800_03248 hypothetical protein
mac03212	mac_D5800_03249 hypothetical protein
mac03228	mac_D5800_03265 hypothetical protein
mac03229	mac_D5800_03266 hypothetical protein

mac03246	mac_D5800_03283 Aerobic respiration control sensor protein ArcB
mac03248	mac_D5800_03285 Autoinducer 2 sensor kinase/phosphatase LuxQ
mac03249	mac_D5800_03286 hypothetical protein
mac03251	mac_D5800_03288 hypothetical protein
mac03253	mac_D5800_03290 hypothetical protein
mac03255	mac_D5800_03292 hypothetical protein
mac03258	mac_D5800_03295 hypothetical protein
mac03261	mac_D5800_03298 Aminoglycoside phosphotransferase
mac03269	mac_D5800_03306 hypothetical protein
mac03273	mac_D5800_03310 hypothetical protein
mac03278	mac_D5800_03315 hypothetical protein
mac03280	mac_D5800_03317 Chaperone protein DnaJ
mac03282	mac_D5800_03319 hypothetical protein
mac03283	mac_D5800_03320 hypothetical protein
mac03289	mac_D5800_03326 Aerobic respiration control sensor protein ArcB
mac03291	mac_D5800_03328 hypothetical protein
mac03292	mac_D5800_03329 Helix-destabilizing protein
mac03293	mac_D5800_03330 hypothetical protein
mac03294	mac_D5800_03331 DNA methylase
mac03295	mac_D5800_03332 hypothetical protein
mac03303	mac_D5800_03340 hypothetical protein
mac03308	mac_D5800_03345 hypothetical protein
mac03309	mac_D5800_03346 hypothetical protein
mac03314	mac_D5800_03351 IS2 transposase TnpB
mac03315	mac_D5800_03352 insertion element IS2 transposase InsD
mac03316	mac_D5800_03353 hypothetical protein
mac03317	mac_D5800_03354 hypothetical protein
mac03325	mac_D5800_03362 hypothetical protein
mac03328	mac_D5800_03369 hypothetical protein
mac03331	mac_D5800_03372 Transposase for transposon Tn5

mac03334	mac_D5800_03376 hypothetical protein
mac03336	mac_D5800_03378 IS2 repressor TnpA
micdadei00023	micdadei_00023 hypothetical protein
micdadei00094	micdadei_00094 hypothetical protein
micdadei00102	micdadei_00102 hypothetical protein
micdadei00103	micdadei_00103 putative oxidoreductase
micdadei00104	micdadei_00104 Tetracycline resistance protein, class C
micdadei00106	micdadei_00106 enterobactin synthase subunit F
micdadei00107	micdadei_00107 Tyrocidine synthase III
micdadei00108	micdadei_00108 protoporphyrinogen oxidase
micdadei00119	micdadei_00119 hypothetical protein
micdadei00133	micdadei_00133 hypothetical protein
micdadei00136	micdadei_00136 hypothetical protein
micdadei00139	micdadei_00139 ankyrin repeat protein
micdadei00140	micdadei_00140 hypothetical protein
micdadei00141	micdadei_00141 hypothetical protein
micdadei00144	micdadei_00144 hypothetical protein
micdadei00146	micdadei_00146 hypothetical protein
micdadei00147	micdadei_00147 hypothetical protein
micdadei00148	micdadei_00148 hypothetical protein
micdadei00149	micdadei_00149 hypothetical protein
micdadei00150	micdadei_00150 Karyopherin (importin) beta
micdadei00152	micdadei_00152 hypothetical protein
micdadei00155	micdadei_00155 hypothetical protein
micdadei00157	micdadei_00157 hypothetical protein
micdadei00160	micdadei_00160 hypothetical protein
micdadei00206	micdadei_00210 hypothetical protein
micdadei00224	micdadei_00228 hypothetical protein
micdadei00241	micdadei_00245 hypothetical protein
micdadei00277	micdadei_00281 hypothetical protein

micdadei00278	micdadei_00282 Transcriptional regulatory protein RstA
micdadei00282	micdadei_00286 hypothetical protein
micdadei00284	micdadei_00288 hypothetical protein
micdadei00285	micdadei_00289 hypothetical protein
micdadei00292	micdadei_00296 hypothetical protein
micdadei00302	micdadei_00306 O-acetyl-ADP-ribose deacetylase
micdadei00305	micdadei_00309 hypothetical protein
micdadei00314	micdadei_00318 hypothetical protein
micdadei00316	micdadei_00320 hypothetical protein
micdadei00318	micdadei_00322 hypothetical protein
micdadei00322	micdadei_00326 hypothetical protein
micdadei00336	micdadei_00340 hypothetical protein
micdadei00360	micdadei_00364 hypothetical protein
micdadei00449	micdadei_00456 DNA-binding transcriptional activator YeiL
micdadei00499	micdadei_00506 hypothetical protein
micdadei00515	micdadei_00522 hypothetical protein
micdadei00567	micdadei_00575 Sensory/regulatory protein RpfC
micdadei00569	micdadei_00577 hypothetical protein
micdadei00573	micdadei_00581 hypothetical protein
micdadei00574	micdadei_00582 hypothetical protein
micdadei00577	micdadei_00585 Type I restriction-modification system methyltransferase subunit
micdadei00597	micdadei_00605 hypothetical protein
micdadei00623	micdadei_00631 hypothetical protein
micdadei00650	micdadei_00658 hypothetical protein
micdadei00655	micdadei_00663 hypothetical protein
micdadei00659	micdadei_00667 hypothetical protein
micdadei00719	micdadei_00727 hypothetical protein
micdadei00731	micdadei_00745 hypothetical protein
micdadei00752	micdadei_00769 hypothetical protein
micdadei00762	micdadei_00779 hypothetical protein

micdadei00780	micdadei_00797 hypothetical protein
micdadei00814	micdadei_00831 Zinc-type alcohol dehydrogenase-like protein
micdadei00908	micdadei_00926 hypothetical protein
micdadei00920	micdadei_00938 hypothetical protein
micdadei00935	micdadei_00953 hypothetical protein
micdadei00970	micdadei_00989 Carboxylate-amine ligase YbdK
micdadei00977	micdadei_00996 hypothetical protein
micdadei01049	micdadei_01068 hypothetical protein
micdadei01061	micdadei_01081 hypothetical protein
micdadei01062	micdadei_01082 hypothetical protein
micdadei01068	micdadei_01088 hypothetical protein
micdadei01093	micdadei_01113 hypothetical protein
micdadei01103	micdadei_01123 hypothetical protein
micdadei01146	micdadei_01166 hypothetical protein
micdadei01147	micdadei_01167 hypothetical protein
micdadei01149	micdadei_01169 hypothetical protein
micdadei01152	micdadei_01172 hypothetical protein
micdadei01153	micdadei_01173 hypothetical protein
micdadei01154	micdadei_01174 Ribosomal-protein-serine acetyltransferase
micdadei01178	micdadei_01198 hypothetical protein
micdadei01181	micdadei_01201 ribosomal-protein-alanine acetyltransferase
micdadei01185	micdadei_01205 hypothetical protein
micdadei01186	micdadei_01206 hypothetical protein
micdadei01231	micdadei_01251 hypothetical protein
micdadei01254	micdadei_01274 hypothetical protein
micdadei01257	micdadei_01277 hypothetical protein
micdadei01414	micdadei_01436 hypothetical protein
micdadei01415	micdadei_01438 Sensor protein EvgS precursor
micdadei01430	micdadei_01453 hypothetical protein
micdadei01530	micdadei_01554 hypothetical protein

micdadei01598	micdadei_01623 hypothetical protein
micdadei01660	micdadei_01686 hypothetical protein
micdadei01680	micdadei_01706 hypothetical protein
micdadei01697	micdadei_01724 hypothetical protein
micdadei01716	micdadei_01743 hypothetical protein
micdadei01718	micdadei_01745 hypothetical protein
micdadei01725	micdadei_01752 hypothetical protein
micdadei01736	micdadei_01763 putative cation-transporting ATPase F
micdadei01743	micdadei_01770 hypothetical protein
micdadei01761	micdadei_01788 homoserine kinase
micdadei01764	micdadei_01791 hypothetical protein
micdadei01787	micdadei_01815 hypothetical protein
micdadei01791	micdadei_01819 hypothetical protein
micdadei01915	micdadei_01947 hypothetical protein
micdadei01961	micdadei_01993 putative siderophore biosynthesis protein SbnA
micdadei01966	micdadei_01998 hypothetical protein
micdadei02004	micdadei_02036 hypothetical protein
micdadei02057	micdadei_02089 hypothetical protein
micdadei02163	micdadei_02197 hypothetical protein
micdadei02262	micdadei_02296 Isochorismatase family protein
micdadei02263	micdadei_02297 hypothetical protein
micdadei02265	micdadei_02299 hypothetical protein
micdadei02268	micdadei_02302 hypothetical protein
micdadei02343	micdadei_02378 hypothetical protein
micdadei02380	micdadei_02416 hypothetical protein
micdadei02415	micdadei_02452 DNA-dependent helicase II
micdadei02529	micdadei_02567 hypothetical protein
micdadei02596	micdadei_02635 hypothetical protein
micdadei02600	micdadei_02639 hypothetical protein
micdadei02605	micdadei_02644 hypothetical protein

micdadei02667	micdadei_02706 hypothetical protein
micdadei02674	micdadei_02713 efflux transporter, outer membrane factor (OMF) lipoprotein, NodT family
micdadei02681	micdadei_02720 hypothetical protein
micdadei02703	micdadei_02742 hypothetical protein
micdadei02704	micdadei_02743 hypothetical protein
micdadei02732	micdadei_02771 hypothetical protein
micdadei02800	micdadei_02839 Cyclic nucleotide-binding domain protein
micdadei02817	micdadei_02856 hypothetical protein
nautram00009	PROKKA_00009 U-box domain protein
nautram00029	PROKKA_00029 hypothetical protein
nautram00072	PROKKA_00075 hypothetical protein
nautram00089	PROKKA_00092 Putative ABC transporter arginine-binding protein 2 precursor
nautram00116	PROKKA_00119 multidrug efflux system subunit MdtA
nautram00119	PROKKA_00122 hypothetical protein
nautram00125	PROKKA_00128 WD domain, G-beta repeat
nautram00126	PROKKA_00129 hypothetical protein
nautram00129	PROKKA_00132 hypothetical protein
nautram00158	PROKKA_00161 hypothetical protein
nautram00162	PROKKA_00165 Retron-type reverse transcriptase
nautram00165	PROKKA_00168 hypothetical protein
nautram00168	PROKKA_00171 hypothetical protein
nautram00169	PROKKA_00172 hypothetical protein
nautram00176	PROKKA_00179 hypothetical protein
nautram00178	PROKKA_00181 hypothetical protein
nautram00179	PROKKA_00182 hypothetical protein
nautram00184	PROKKA_00187 hypothetical protein
nautram00185	PROKKA_00188 hypothetical protein
nautram00186	PROKKA_00189 hypothetical protein
nautram00189	PROKKA_00192 hypothetical protein
nautram00192	PROKKA_00195 Antibiotic biosynthesis monooxygenase

nautram00194	PROKKA_00197 Tyrosine recombinase XerC
nautram00242	PROKKA_00245 hypothetical protein
nautram00265	PROKKA_00268 hypothetical protein
nautram00266	PROKKA_00269 VIT family protein
nautram00310	PROKKA_00313 hypothetical protein
nautram00316	PROKKA_00319 Aspartokinase I/homoserine dehydrogenase I
nautram00317	PROKKA_00320 Homoserine kinase
nautram00324	PROKKA_00327 hypothetical protein
nautram00336	PROKKA_00339 hypothetical protein
nautram00343	PROKKA_00346 hypothetical protein
nautram00354	PROKKA_00357 Dipeptide and tripeptide permease B
nautram00432	PROKKA_00438 hypothetical protein
nautram00461	PROKKA_00467 hypothetical protein
nautram00481	PROKKA_00487 hypothetical protein
nautram00487	PROKKA_00493 hypothetical protein
nautram00489	PROKKA_00495 hypothetical protein
nautram00502	PROKKA_00508 Nicotinate degradation protein S
nautram00503	PROKKA_00509 hypothetical protein
nautram00504	PROKKA_00510 hypothetical protein
nautram00505	PROKKA_00511 RasGEF domain protein
nautram00535	PROKKA_00542 Riboflavin biosynthesis protein RibF
nautram00579	PROKKA_00586 hypothetical protein
nautram00610	PROKKA_00617 hypothetical protein
nautram00620	PROKKA_00627 putative acetyltransferase
nautram00624	PROKKA_00631 ecdysteroid UDP-glucosyltransferase
nautram00629	PROKKA_00636 hypothetical protein
nautram00672	PROKKA_00679 hypothetical protein
nautram00677	PROKKA_00684 hypothetical protein
nautram00690	PROKKA_00697 hypothetical protein
nautram00691	PROKKA_00698 putative RNA methylase

nautram00692	PROKKA_00699 hypothetical protein
nautram00693	PROKKA_00700 Ergosterol biosynthesis ERG4/ERG24 family protein
nautram00696	PROKKA_00703 Beta-ketoacyl-acyl-carrier-protein synthase I
nautram00706	PROKKA_00713 hypothetical protein
nautram00707	PROKKA_00714 hypothetical protein
nautram00762	PROKKA_00770 hypothetical protein
nautram00765	PROKKA_00773 hypothetical protein
nautram00769	PROKKA_00777 hypothetical protein
nautram00770	PROKKA_00778 hypothetical protein
nautram00789	PROKKA_00798 hypothetical protein
nautram00791	PROKKA_00800 Ribosome-binding ATPase YchF
nautram00812	PROKKA_00821 hypothetical protein
nautram00819	PROKKA_00828 hypothetical protein
nautram00831	PROKKA_00840 hypothetical protein
nautram00835	PROKKA_00844 hypothetical protein
nautram00866	PROKKA_00875 hypothetical protein
nautram00883	PROKKA_00893 hypothetical protein
nautram00929	PROKKA_00939 hypothetical protein
nautram00935	PROKKA_00946 hypothetical protein
nautram00958	PROKKA_00969 hypothetical protein
nautram00987	PROKKA_00998 hypothetical protein
nautram00988	PROKKA_00999 hypothetical protein
nautram01011	PROKKA_01023 hypothetical protein
nautram01046	PROKKA_01058 Glycine cleavage system T protein (aminomethyltransferase)
nautram01053	PROKKA_01065 hypothetical protein
nautram01054	PROKKA_01066 hypothetical protein
nautram01055	PROKKA_01067 hypothetical protein
nautram01069	PROKKA_01081 hypothetical protein
nautram01086	PROKKA_01098 hypothetical protein
nautram01088	PROKKA_01100 Transposase, TnpA family

nautram01089	PROKKA_01101 hypothetical protein
nautram01093	PROKKA_01105 hypothetical protein
nautram01094	PROKKA_01106 Nicotinamide nucleotide repair protein
nautram01098	PROKKA_01110 Tyrosine recombinase XerC
nautram01123	PROKKA_01135 Acetolactate synthase isozyme 2 large subunit
nautram01124	PROKKA_01136 hypothetical protein
nautram01143	PROKKA_01156 hypothetical protein
nautram01176	PROKKA_01189 hypothetical protein
nautram01177	PROKKA_01190 hypothetical protein
nautram01182	PROKKA_01195 hypothetical protein
nautram01183	PROKKA_01196 Glycosyl transferases group 1
nautram01212	PROKKA_01225 hypothetical protein
nautram01269	PROKKA_01291 hypothetical protein
nautram01270	PROKKA_01292 hypothetical protein
nautram01285	PROKKA_01307 Acyl-CoA dehydrogenase, middle domain
nautram01286	PROKKA_01308 hypothetical protein
nautram01288	PROKKA_01310 hypothetical protein
nautram01290	PROKKA_01312 hypothetical protein
nautram01291	PROKKA_01313 hypothetical protein
nautram01292	PROKKA_01314 hypothetical protein
nautram01293	PROKKA_01315 hypothetical protein
nautram01309	PROKKA_01331 hypothetical protein
nautram01310	PROKKA_01332 hypothetical protein
nautram01311	PROKKA_01333 hypothetical protein
nautram01312	PROKKA_01334 hypothetical protein
nautram01313	PROKKA_01335 Transposase, TnpA family
nautram01314	PROKKA_01336 hypothetical protein
nautram01358	PROKKA_01381 hypothetical protein
nautram01360	PROKKA_01383 hypothetical protein
nautram01369	PROKKA_01392 D-alanine--poly(phosphoribitol) ligase subunit 1

nautram01372	PROKKA_01395 hypothetical protein
nautram01378	PROKKA_01401 Protein phosphatase 2C
nautram01426	PROKKA_01450 chaperone protein DnaJ
nautram01456	PROKKA_01480 hypothetical protein
nautram01470	PROKKA_01494 hypothetical protein
nautram01551	PROKKA_01577 hypothetical protein
nautram01553	PROKKA_01579 hypothetical protein
nautram01617	PROKKA_01643 hypothetical protein
nautram01618	PROKKA_01644 hypothetical protein
nautram01629	PROKKA_01655 hypothetical protein
nautram01722	PROKKA_01748 hypothetical protein
nautram01728	PROKKA_01754 hypothetical protein
nautram01729	PROKKA_01755 hypothetical protein
nautram01739	PROKKA_01765 Bacteriophytochrome cph2
nautram01744	PROKKA_01770 hypothetical protein
nautram01749	PROKKA_01775 hypothetical protein
nautram01751	PROKKA_01777 hypothetical protein
nautram01752	PROKKA_01778 hypothetical protein
nautram01756	PROKKA_01782 hypothetical protein
nautram01758	PROKKA_01784 Toxin HigB-1
nautram01759	PROKKA_01785 hypothetical protein
nautram01763	PROKKA_01789 hypothetical protein
nautram01771	PROKKA_01797 putative transposase OrfB
nautram01774	PROKKA_01800 hypothetical protein
nautram01779	PROKKA_01805 hypothetical protein
nautram01781	PROKKA_01807 hypothetical protein
nautram01786	PROKKA_01812 hypothetical protein
nautram01788	PROKKA_01814 hypothetical protein
nautram01798	PROKKA_01824 hypothetical protein
nautram01799	PROKKA_01825 hypothetical protein

nautram01800	PROKKA_01826 LD-carboxypeptidase
nautram01801	PROKKA_01827 hypothetical protein
nautram01804	PROKKA_01830 hypothetical protein
nautram01805	PROKKA_01831 hypothetical protein
nautram01806	PROKKA_01832 hypothetical protein
nautram01818	PROKKA_01844 hypothetical protein
nautram01834	PROKKA_01860 Poly(A) polymerase I precursor
nautram01835	PROKKA_01861 hypothetical protein
nautram01836	PROKKA_01862 hypothetical protein
nautram01845	PROKKA_01872 hypothetical protein
nautram01848	PROKKA_01875 hypothetical protein
nautram01859	PROKKA_01886 hypothetical protein
nautram01860	PROKKA_01887 hypothetical protein
nautram01861	PROKKA_01888 hypothetical protein
nautram01862	PROKKA_01889 hypothetical protein
nautram01867	PROKKA_01894 hypothetical protein
nautram01897	PROKKA_01924 hypothetical protein
nautram01898	PROKKA_01925 hypothetical protein
nautram01899	PROKKA_01926 hypothetical protein
nautram01900	PROKKA_01927 hypothetical protein
nautram01909	PROKKA_01936 homoserine kinase
nautram01910	PROKKA_01937 hypothetical protein
nautram01955	PROKKA_01985 Sensor protein ZraS
nautram01956	PROKKA_01986 hypothetical protein
nautram01958	PROKKA_01988 hypothetical protein
nautram01959	PROKKA_01989 hypothetical protein
nautram01960	PROKKA_01990 Response regulator containing a CheY-like receiver domain and an HD-GYP domain protein
nautram01961	PROKKA_01991 CAI-1 autoinducer sensor kinase/phosphatase CqsS
nautram01996	PROKKA_02026 hypothetical protein
nautram02019	PROKKA_02049 hypothetical protein

nautram02049	PROKKA_02079 hypothetical protein
nautram02050	PROKKA_02080 hypothetical protein
nautram02060	PROKKA_02090 hypothetical protein
nautram02061	PROKKA_02091 hypothetical protein
nautram02072	PROKKA_02102 hypothetical protein
nautram02079	PROKKA_02109 hypothetical protein
nautram02080	PROKKA_02110 hypothetical protein
nautram02091	PROKKA_02121 putative cadmium-transporting ATPase
nautram02092	PROKKA_02122 Transcriptional repressor FrmR
nautram02093	PROKKA_02123 hypothetical protein
nautram02113	PROKKA_02143 Silver exporting P-type ATPase
nautram02116	PROKKA_02146 hypothetical protein
nautram02118	PROKKA_02148 hypothetical protein
nautram02238	PROKKA_02268 hypothetical protein
nautram02240	PROKKA_02270 hypothetical protein
nautram02242	PROKKA_02272 hypothetical protein
nautram02253	PROKKA_02283 hypothetical protein
nautram02254	PROKKA_02284 Phosphinothricin N-acetyltransferase
nautram02259	PROKKA_02289 hypothetical protein
nautram02266	PROKKA_02296 hypothetical protein
nautram02275	PROKKA_02305 hypothetical protein
nautram02278	PROKKA_02308 hypothetical protein
nautram02285	PROKKA_02315 5'-methylthioadenosine/S-adenosylhomocysteine nucleosidase
nautram02293	PROKKA_02323 hypothetical protein
nautram02372	PROKKA_02402 GAF domain protein
nautram02373	PROKKA_02403 hypothetical protein
nautram02394	PROKKA_02425 hypothetical protein
nautram02400	PROKKA_02431 hypothetical protein
nautram02437	PROKKA_02468 hypothetical protein
nautram02438	PROKKA_02469 hypothetical protein

nautram02439	PROKKA_02470 phosphonate utilization associated putative membrane protein
nautram02481	PROKKA_02512 hypothetical protein
nautram02490	PROKKA_02521 hypothetical protein
nautram02493	PROKKA_02524 hypothetical protein
nautram02510	PROKKA_02541 lineage-specific thermal regulator protein
nautram02521	PROKKA_02552 hypothetical protein
nautram02522	PROKKA_02553 hypothetical protein
nautram02565	PROKKA_02596 hypothetical protein
nautram02566	PROKKA_02597 hypothetical protein
nautram02572	PROKKA_02603 hypothetical protein
nautram02573	PROKKA_02604 hypothetical protein
nautram02575	PROKKA_02606 hypothetical protein
nautram02576	PROKKA_02607 hypothetical protein
nautram02577	PROKKA_02608 Calcium-transporting ATPase
nautram02580	PROKKA_02611 Alcohol dehydrogenase
nautram02585	PROKKA_02616 hypothetical protein
nautram02594	PROKKA_02625 hypothetical protein
nautram02596	PROKKA_02627 hypothetical protein
nautram02600	PROKKA_02631 hypothetical protein
nautram02601	PROKKA_02632 hypothetical protein
nautram02603	PROKKA_02634 Microcin C7 self-immunity protein MccF
nautram02606	PROKKA_02637 hypothetical protein
nautram02607	PROKKA_02638 hypothetical protein
nautram02619	PROKKA_02650 hypothetical protein
nautram02627	PROKKA_02658 hypothetical protein
nautram02654	PROKKA_02686 bifunctional aldehyde dehydrogenase/enoyl-CoA hydratase
nautram02682	PROKKA_02714 hypothetical protein
nautram02687	PROKKA_02719 hypothetical protein
nautram02689	PROKKA_02721 Antirestriction protein KlcA
nautram02690	PROKKA_02722 hypothetical protein

nautram02692	PROKKA_02724 hypothetical protein
nautram02693	PROKKA_02725 hypothetical protein
nautram02694	PROKKA_02726 type IV secretion system T-DNA border endonuclease VirD2
nautram02695	PROKKA_02727 hypothetical protein
nautram02696	PROKKA_02728 hypothetical protein
nautram02697	PROKKA_02729 hypothetical protein
nautram02700	PROKKA_02732 hypothetical protein
nautram02704	PROKKA_02736 DNA transport protein TraD
nautram02705	PROKKA_02737 hypothetical protein
nautram02706	PROKKA_02738 conjugal transfer mating pair stabilization protein TraG
nautram02708	PROKKA_02740 conjugal transfer protein TrbB
nautram02709	PROKKA_02741 conjugal pilus assembly protein TraF
nautram02710	PROKKA_02742 conjugal transfer mating pair stabilization protein TraN
nautram02711	PROKKA_02743 conjugal transfer pilus assembly protein TrbC
nautram02712	PROKKA_02744 conjugal transfer pilus assembly protein TraU
nautram02713	PROKKA_02745 conjugal transfer pilus assembly protein TraW
nautram02714	PROKKA_02746 conjugal transfer protein TrbI
nautram02715	PROKKA_02747 conjugal transfer ATP-binding protein TraC
nautram02716	PROKKA_02748 hypothetical protein
nautram02717	PROKKA_02749 conjugal transfer protein TraV
nautram02718	PROKKA_02750 conjugal transfer pilus assembly protein TraB
nautram02719	PROKKA_02751 conjugal transfer protein TraK
nautram02720	PROKKA_02752 conjugal transfer pilus assembly protein TraE
nautram02721	PROKKA_02753 conjugal transfer pilus assembly protein TraL
nautram02722	PROKKA_02754 hypothetical protein
nautram02728	PROKKA_02760 hypothetical protein
nautram02729	PROKKA_02761 hypothetical protein
nautram02730	PROKKA_02762 TDP-fucosamine acetyltransferase
nautram02731	PROKKA_02763 Sensor protein CreC
nautram02732	PROKKA_02764 hypothetical protein

nautram02733	PROKKA_02765 hypothetical protein
nautram02734	PROKKA_02766 hypothetical protein
nautram02794	PROKKA_02826 hypothetical protein
nautram02811	PROKKA_02844 hypothetical protein
nautram02872	PROKKA_02906 hypothetical protein
nautram02898	PROKKA_02932 Pyrimidine deaminase
nautram02899	PROKKA_02933 hypothetical protein
nautram02909	PROKKA_02943 ubiquinone/menaquinone biosynthesis methyltransferase
nautram02917	PROKKA_02951 hypothetical protein
nautram02918	PROKKA_02952 hypothetical protein
nautram02934	PROKKA_02968 hypothetical protein
nautram02944	PROKKA_02978 hypothetical protein
nautram02948	PROKKA_02982 hypothetical protein
nautram02949	PROKKA_02983 hypothetical protein
nautram02950	PROKKA_02984 hypothetical protein
nautram02958	PROKKA_02992 conjugal transfer ATP-binding protein TraC
nautram02961	PROKKA_02995 Transposase, TnpA family
nautram02964	PROKKA_02998 hypothetical protein
nautram02965	PROKKA_02999 hypothetical protein
nautram02967	PROKKA_03001 hypothetical protein
nautram03029	PROKKA_03063 Uridine kinase
nautram03055	PROKKA_03089 hypothetical protein
nautram03059	PROKKA_03093 hypothetical protein
nautram03060	PROKKA_03094 putative protein containing caspase domain protein
nautram03062	PROKKA_03096 hypothetical protein
nautram03064	PROKKA_03098 hypothetical protein
nautram03065	PROKKA_03099 hypothetical protein
nautram03066	PROKKA_03100 hypothetical protein
nautram03067	PROKKA_03101 hypothetical protein
nautram03068	PROKKA_03102 hypothetical protein

nautram03125	PROKKA_03159 hypothetical protein
nautram03127	PROKKA_03161 hypothetical protein
nautram03128	PROKKA_03162 hypothetical protein
nautram03138	PROKKA_03172 hypothetical protein
nautram03154	PROKKA_03188 Histone methylation protein DOT1
nautram03155	PROKKA_03189 putative integral membrane sensor domain protein
nautram03156	PROKKA_03190 hypothetical protein
nautram03167	PROKKA_03201 hypothetical protein
nautram03174	PROKKA_03208 hypothetical protein
nautram03188	PROKKA_03222 hypothetical protein
nautram03200	PROKKA_03234 hypothetical protein
nautram03212	PROKKA_03246 hypothetical protein
nautram03215	PROKKA_03249 hypothetical protein
nautram03223	PROKKA_03257 hypothetical protein
nautram03225	PROKKA_03259 hypothetical protein
nautram03231	PROKKA_03265 hypothetical protein
nautram03250	PROKKA_03285 hypothetical protein
nautram03252	PROKKA_03287 Transposase IS66 family protein
nautram03259	PROKKA_03294 hypothetical protein
nautram03260	PROKKA_03295 hypothetical protein
nautram03263	PROKKA_03298 hypothetical protein
nautram03269	PROKKA_03304 hypothetical protein
nautram03281	PROKKA_03316 hypothetical protein
nautram03283	PROKKA_03318 Toxin HigB-2
nautram03285	PROKKA_03320 hypothetical protein
nautram03301	PROKKA_03336 hypothetical protein
nautram03302	PROKKA_03337 hypothetical protein
nautram03304	PROKKA_03339 Proline porter II
nautram03306	PROKKA_03341 hypothetical protein
nautram03327	PROKKA_03362 Ribbon-helix-helix protein, copG family

nautram03338	PROKKA_03374 hypothetical protein
nautram03341	PROKKA_03377 hypothetical protein
nautram03343	PROKKA_03379 hypothetical protein
nautram03344	PROKKA_03380 hypothetical protein
nautram03349	PROKKA_03385 hypothetical protein
nautram03350	PROKKA_03386 hypothetical protein
nautram03354	PROKKA_03390 hypothetical protein
nautram03362	PROKKA_03398 putative nucleotide-binding protein
nautram03364	PROKKA_03400 hypothetical protein
nautram03368	PROKKA_03404 hypothetical protein
nautram03369	PROKKA_03405 Bifunctional polymyxin resistance protein ArnA
nautram03373	PROKKA_03409 hypothetical protein

**Supplementary Table 3c. Unique genes/gene clusters found only in the Clade 3 *Legionella* species**	
Anisa02186	PROKKA_02186 hypothetical protein
Anisa00104	PROKKA_00104 hypothetical protein
Anisa00175	PROKKA_00175 putative inner membrane transporter yiJE
Anisa00801	PROKKA_00801 hypothetical protein
Anisa00968	PROKKA_00968 hypothetical protein
Anisa01185	PROKKA_01185 hypothetical protein
Anisa01621	PROKKA_01621 Acyl carrier protein
Anisa01681	PROKKA_01681 Carboxylesterase NlhH
Anisa01692	PROKKA_01692 putative membrane protein
Anisa01786	PROKKA_01786 hypothetical protein
Anisa01796	PROKKA_01796 hypothetical protein
Anisa01883	PROKKA_01883 Small integral membrane protein
Anisa02014	PROKKA_02014 hypothetical protein

Anisa02038	PROKKA_02038 hypothetical protein
Anisa02187	PROKKA_02187 hypothetical protein
Anisa02474	PROKKA_02474 hypothetical protein
Anisa02478	PROKKA_02478 hypothetical protein
Anisa02558	PROKKA_02558 L-aspartate transporter
Anisa02733	PROKKA_02733 2-polyprenylphenol 6-hydroxylase
Anisa02762	PROKKA_02762 hypothetical protein
Anisa02783	PROKKA_02783 Mitochondrial carrier protein
Anisa02853	PROKKA_02853 hypothetical protein
Anisa02975	PROKKA_02975 hypothetical protein
Anisa02982	PROKKA_02982 hypothetical protein
Anisa03411	PROKKA_03411 hypothetical protein
Anisa03589	PROKKA_03589 hypothetical protein
Anisa00130	PROKKA_00130 hypothetical protein
Anisa00471	PROKKA_00471 Multifunctional CCA protein
Anisa00568	PROKKA_00568 carbamoyl phosphate synthase-like protein
Anisa01060	PROKKA_01060 Multidrug resistance protein D
Anisa01141	PROKKA_01141 hypothetical protein
Anisa01688	PROKKA_01688 biotin biosynthesis protein BioC
Anisa01936	PROKKA_01936 Spore protein SP21
Anisa02003	PROKKA_02003 GtrA-like protein
Anisa02335	PROKKA_02335 hypothetical protein
Anisa02611	PROKKA_02611 3-oxoacyl-[acyl-carrier-protein] reductase FabG
Anisa03051	PROKKA_03051 hypothetical protein
Anisa03695	PROKKA_03695 ATP-dependent zinc metalloprotease FtsH 4
Cincinnati00155	PROKKA_00155 hypothetical protein
Cincinnati00369	PROKKA_00369 hypothetical protein
Cincinnati00671	PROKKA_00677 Pseudouridine kinase
Anisa00107	PROKKA_00107 hypothetical protein
Anisa00422	PROKKA_00422 hypothetical protein

Anisa00464	PROKKA_00464 hypothetical protein
Anisa00755	PROKKA_00755 hypothetical protein
Anisa01126	PROKKA_01126 SET domain protein
Anisa01230	PROKKA_01230 Spectinomycin tetracycline efflux pump
Anisa01581	PROKKA_01581 hypothetical protein
Anisa01613	PROKKA_01613 ABC exporter transmembrane subunit, DevC protein
Anisa01707	PROKKA_01707 cellulose synthase operon protein YhjU
Anisa01708	PROKKA_01708 Cellulose synthase operon protein C precursor
Anisa01715	PROKKA_01715 Cellulose synthase catalytic subunit [UDP-forming]
Anisa01716	PROKKA_01716 Cellulose synthase regulatory subunit
Anisa01724	PROKKA_01724 hypothetical protein
Anisa01848	PROKKA_01848 hypothetical protein
Anisa01891	PROKKA_01891 Glucosamine-6-phosphate deaminase 1
Anisa01971	PROKKA_01971 hypothetical protein
Anisa01993	PROKKA_01993 Mitochondrial carrier protein
Anisa02001	PROKKA_02001 hypothetical protein
Anisa02316	PROKKA_02316 Exopolysaccharide biosynthesis protein related to N-acetylglucosamine-1-phosphodiester alp
Anisa02317	PROKKA_02317 hypothetical protein
Anisa02440	PROKKA_02440 hypothetical protein
Anisa02486	PROKKA_02486 hypothetical protein
Anisa02570	PROKKA_02570 hypothetical protein
Anisa02845	PROKKA_02845 putative transporter
Anisa03422	PROKKA_03422 hypothetical protein
Anisa03478	PROKKA_03478 hypothetical protein
Anisa03555	PROKKA_03555 hypothetical protein
Anisa03690	PROKKA_03690 Putative universal stress protein
Cincinnati02421	PROKKA_02443 hypothetical protein
Cincinnati03216	PROKKA_03252 Epimerase family protein
NSW150_02302	NSW150_02339 Outer membrane protein OprM precursor
Anisa00093	PROKKA_00093 Riboflavin biosynthesis protein RibBA

Anisa00094	PROKKA_00094 Galactitol-1-phosphate 5-dehydrogenase
Anisa00095	PROKKA_00095 queuosine biosynthesis protein QueD
Anisa00189	PROKKA_00189 E3 ubiquitin-protein ligase SlrP
Anisa00485	PROKKA_00485 hypothetical protein
Anisa00486	PROKKA_00486 hypothetical protein
Anisa00487	PROKKA_00487 hypothetical protein
Anisa00488	PROKKA_00488 hypothetical protein
Anisa00489	PROKKA_00489 hypothetical protein
Anisa00490	PROKKA_00490 putative oxidoreductase
Anisa00491	PROKKA_00491 Putative protein-S-isoprenylcysteine methyltransferase
Anisa00702	PROKKA_00702 FmtA-like protein
Anisa00868	PROKKA_00868 hypothetical protein
Anisa00940	PROKKA_00940 hypothetical protein
Anisa00980	PROKKA_00980 Isochorismatase family protein
Anisa01620	PROKKA_01620 hypothetical protein
Anisa01694	PROKKA_01694 putative secreted hydrolase
Anisa01714	PROKKA_01714 cell division protein
Anisa01717	PROKKA_01717 Endoglucanase precursor
Anisa01807	PROKKA_01807 Cytosol aminopeptidase
Anisa01826	PROKKA_01826 Transcriptional regulatory protein FixJ
Anisa01832	PROKKA_01832 hypothetical protein
Anisa01834	PROKKA_01834 hypothetical protein
Anisa01836	PROKKA_01836 hypothetical protein
Anisa01840	PROKKA_01840 Adenine deaminase
Anisa01854	PROKKA_01854 hypothetical protein
Anisa01860	PROKKA_01860 hypothetical protein
Anisa01893	PROKKA_01893 hypothetical protein
Anisa01896	PROKKA_01896 hypothetical protein
Anisa01912	PROKKA_01912 hypothetical protein
Anisa01930	PROKKA_01930 Mycobacterial persistence regulator A

Anisa02020	PROKKA_02020 hypothetical protein
Anisa02060	PROKKA_02060 family membrane protein
Anisa02148	PROKKA_02148 Prolyl tripeptidyl peptidase precursor
Anisa02149	PROKKA_02149 putative ester cyclase
Anisa02168	PROKKA_02168 hypothetical protein
Anisa02190	PROKKA_02190 hypothetical protein
Anisa02436	PROKKA_02436 Putative DNA ligase-like protein/MT0965
Anisa02597	PROKKA_02597 hypothetical protein
Anisa02829	PROKKA_02829 hypothetical protein
Anisa02830	PROKKA_02830 hypothetical protein
Anisa02867	PROKKA_02867 hypothetical protein
Anisa02984	PROKKA_02984 Diaminopimelate decarboxylase
Anisa02998	PROKKA_02998 Chondroitin polymerase
Anisa03744	PROKKA_03744 Inner membrane transport protein YdhC
Cincinnati00105	PROKKA_00105 hypothetical protein
Cincinnati02530	PROKKA_02555 hypothetical protein
Cincinnati02531	PROKKA_02556 hypothetical protein
Anisa00243	PROKKA_00243 hypothetical protein
Anisa00421	PROKKA_00421 hypothetical protein
Anisa00549	PROKKA_00549 hypothetical protein
Anisa00591	PROKKA_00591 hypothetical protein
Anisa00884	PROKKA_00884 Outer membrane protein OprM precursor
Anisa00917	PROKKA_00917 hypothetical protein
Anisa00931	PROKKA_00931 hypothetical protein
Anisa01067	PROKKA_01067 hypothetical protein
Anisa01181	PROKKA_01181 TVP38/TMEM64 family inner membrane protein YdjZ
Anisa01614	PROKKA_01614 Lipoprotein-releasing system ATP-binding protein LolD
Anisa01615	PROKKA_01615 p-hydroxybenzoic acid efflux pump subunit AaeA
Anisa01616	PROKKA_01616 Toluene efflux pump outer membrane protein TtgF precursor
Anisa01623	PROKKA_01623 PAP2 superfamily protein

Anisa01628	PROKKA_01628 Putative glycosyltransferase EpsE
Anisa01816	PROKKA_01816 ribosomal-protein-alanine acetyltransferase
Anisa01853	PROKKA_01853 hypothetical protein
Anisa01901	PROKKA_01901 hypothetical protein
Anisa01926	PROKKA_01926 putative copper-binding protein
Anisa02004	PROKKA_02004 Morphology and auto-aggregation control protein
Anisa02311	PROKKA_02311 Polar-differentiation response regulator DivK
Anisa02312	PROKKA_02312 Autoinducer 2 sensor kinase/phosphatase LuxQ
Anisa02313	PROKKA_02313 Leucine-specific-binding protein precursor
Anisa02314	PROKKA_02314 hypothetical protein
Anisa02557	PROKKA_02557 hypothetical protein
Anisa02618	PROKKA_02618 hypothetical protein
Anisa02749	PROKKA_02749 hypothetical protein
Anisa02795	PROKKA_02795 hypothetical protein
Anisa02877	PROKKA_02877 hypothetical protein
Anisa03444	PROKKA_03444 hypothetical protein
Anisa03790	PROKKA_03790 hypothetical protein
Anisa03819	PROKKA_03819 hypothetical protein
Cincinnati00195	PROKKA_00195 hypothetical protein
Cincinnati00609	PROKKA_00614 Isopenicillin N epimerase
Cincinnati01538	PROKKA_01554 Esterase EstA precursor
Cincinnati02005	PROKKA_02024 Angiotensin-converting enzyme
NSW150_00765	NSW150_00780 putative distant relative of cell wall-associated hydrolases
NSW150_02303	NSW150_02340 2,3-dihydro-2,3-dihydroxybenzoate dehydrogenase
boze1_00295	PROKKA_00296 hypothetical protein
boze1_01084	PROKKA_01091 hypothetical protein
boze1_03267	PROKKA_03296 hypothetical protein
boze1_03293	PROKKA_03322 hypothetical protein
Anisa00098	PROKKA_00098 hypothetical protein
Anisa00156	PROKKA_00156 hypothetical protein

Anisa00201	PROKKA_00201 hypothetical protein
Anisa00383	PROKKA_00383 hypothetical protein
Anisa00457	PROKKA_00457 putative inner membrane transporter yiJE
Anisa00458	PROKKA_00458 hypothetical protein
Anisa00461	PROKKA_00461 Fluorothreonine transaldolase
Anisa00462	PROKKA_00462 hypothetical protein
Anisa00473	PROKKA_00473 S-adenosylmethionine:diacylglycerol 3-amino-3-carboxypropyl transferase
Anisa00482	PROKKA_00482 ribonuclease Z
Anisa00492	PROKKA_00492 Putative ABC transporter arginine-binding protein 2 precursor
Anisa00567	PROKKA_00567 Tyrocidine synthase III
Anisa00585	PROKKA_00585 hypothetical protein
Anisa00768	PROKKA_00768 Polar organelle development protein
Anisa00880	PROKKA_00880 hypothetical protein
Anisa00927	PROKKA_00927 hypothetical protein
Anisa01015	PROKKA_01015 putative integral membrane protein
Anisa01500	PROKKA_01500 hypothetical protein
Anisa01666	PROKKA_01666 hypothetical protein
Anisa01861	PROKKA_01861 hypothetical protein
Anisa01871	PROKKA_01871 Pyrimidine-specific ribonucleoside hydrolase RihB
Anisa01879	PROKKA_01879 hypothetical protein
Anisa01881	PROKKA_01881 hypothetical protein
Anisa01894	PROKKA_01894 hypothetical protein
Anisa01913	PROKKA_01913 Cyclopentanol dehydrogenase
Anisa01945	PROKKA_01945 Proline porter II
Anisa01967	PROKKA_01967 hypothetical protein
Anisa02047	PROKKA_02047 hypothetical protein
Anisa02174	PROKKA_02174 hypothetical protein
Anisa02361	PROKKA_02361 Phosphoglycerol transferase, alkaline phosphatase superfamily
Anisa02398	PROKKA_02398 hypothetical protein
Anisa02444	PROKKA_02444 hypothetical protein

Anisa02445	PROKKA_02445 hypothetical protein
Anisa02446	PROKKA_02446 Serine/threonine-protein kinase PknH
Anisa02489	PROKKA_02489 hypothetical protein
Anisa02555	PROKKA_02555 Small integral membrane protein
Anisa02564	PROKKA_02564 ankyrin repeat protein
Anisa02566	PROKKA_02566 hypothetical protein
Anisa02758	PROKKA_02758 hypothetical protein
Anisa02842	PROKKA_02842 Aspartate 1-decarboxylase precursor
Anisa02843	PROKKA_02843 D-alanine--D-alanine ligase
Anisa02844	PROKKA_02844 Aspartate racemase
Anisa02876	PROKKA_02876 aspartate aminotransferase
Anisa03284	PROKKA_03284 hypothetical protein
Anisa03317	PROKKA_03317 anthranilate synthase component II
Anisa03461	PROKKA_03461 hypothetical protein
Anisa03463	PROKKA_03463 hypothetical protein
Anisa03464	PROKKA_03464 hypothetical protein
Anisa03567	PROKKA_03567 hypothetical protein
Anisa03585	PROKKA_03585 ankyrin repeat protein
Anisa03666	PROKKA_03666 hypothetical protein
Anisa03672	PROKKA_03672 hypothetical protein
Anisa03750	PROKKA_03750 hypothetical protein
Cincinnati00334	PROKKA_00334 hypothetical protein
Cincinnati00802	PROKKA_00808 hypothetical protein
Cincinnati01152	PROKKA_01165 hypothetical protein
Cincinnati01216	PROKKA_01229 hypothetical protein
Cincinnati01537	PROKKA_01553 Outer membrane flp protein
Cincinnati01544	PROKKA_01560 hypothetical protein
Cincinnati01621	PROKKA_01637 Streptogramin lyase
Cincinnati02426	PROKKA_02448 Protein kinase domain protein
Cincinnati02471	PROKKA_02494 hypothetical protein

Cincinnati03087	PROKKA_03122 hypothetical protein
Cincinnati03125	PROKKA_03160 dihydroorotate dehydrogenase electron transfer subunit
Cincinnati03190	PROKKA_03226 putative DNA-binding protein with PD1-like DNA-binding motif
NSW150_02189	NSW150_02226 hypothetical protein
NSW150_02521	NSW150_02561 hypothetical protein
NSW150_03192	NSW150_03235 8-amino-7-oxononanoate synthase
boze1_00078	PROKKA_00079 hypothetical protein
boze1_00273	PROKKA_00274 trimethyllysine dioxygenase
boze1_00848	PROKKA_00852 Pimelyl-[acyl-carrier protein] methyl ester esterase
boze1_00850	PROKKA_00854 hypothetical protein
boze1_01258	PROKKA_01266 hypothetical protein
boze1_01270	PROKKA_01278 putative ABC transporter ATP-binding protein
boze1_01479	PROKKA_01488 RasGEF domain protein
boze1_01504	PROKKA_01514 Lysine decarboxylase, inducible
boze1_01752	PROKKA_01763 hypothetical protein
boze1_01785	PROKKA_01796 hypothetical protein
boze1_02202	PROKKA_02225 Serine 3-dehydrogenase
boze1_02937	PROKKA_02966 Putative protein-S-isoprenylcysteine methyltransferase
boze1_03028	PROKKA_03057 hypothetical protein
boze1_03233	PROKKA_03262 hypothetical protein
Anisa00004	PROKKA_00004 Lipoprotein-releasing system transmembrane protein LolE
Anisa00006	PROKKA_00006 hypothetical protein
Anisa00118	PROKKA_00118 hypothetical protein
Anisa00122	PROKKA_00122 hypothetical protein
Anisa00474	PROKKA_00474 hypothetical protein
Anisa00475	PROKKA_00475 Pyrroloquinoline quinone (Coenzyme PQQ) biosynthesis protein C
Anisa00476	PROKKA_00476 S-adenosylmethionine:diacylglycerol 3-amino-3-carboxypropyl transferase
Anisa00617	PROKKA_00617 hypothetical protein
Anisa00640	PROKKA_00640 hypothetical protein
Anisa00641	PROKKA_00641 hypothetical protein

Anisa00682	PROKKA_00682 hypothetical protein
Anisa00873	PROKKA_00873 hypothetical protein
Anisa01013	PROKKA_01013 hypothetical protein
Anisa01112	PROKKA_01112 hypothetical protein
Anisa01113	PROKKA_01113 hypothetical protein
Anisa01114	PROKKA_01114 hypothetical protein
Anisa01279	PROKKA_01279 hypothetical protein
Anisa01510	PROKKA_01510 hypothetical protein
Anisa01511	PROKKA_01511 dTDP-glucose 4,6-dehydratase
Anisa01521	PROKKA_01521 Putative glutamine amidotransferasec
Anisa01587	PROKKA_01587 hypothetical protein
Anisa01654	PROKKA_01654 hypothetical protein
Anisa01870	PROKKA_01870 hypothetical protein
Anisa01875	PROKKA_01875 hypothetical protein
Anisa01882	PROKKA_01882 hypothetical protein
Anisa01976	PROKKA_01976 hypothetical protein
Anisa02275	PROKKA_02275 hypothetical protein
Anisa02318	PROKKA_02318 hypothetical protein
Anisa02589	PROKKA_02589 hypothetical protein
Anisa02710	PROKKA_02710 carboxylate-amine ligase
Anisa02712	PROKKA_02712 aerobic sulfatase maturase family protein
Anisa02734	PROKKA_02734 hypothetical protein
Anisa02739	PROKKA_02739 Sulfatase
Anisa02740	PROKKA_02740 Ribosomal protein L11 methylase
Anisa02741	PROKKA_02741 hypothetical protein
Anisa02753	PROKKA_02753 hypothetical protein
Anisa02851	PROKKA_02851 hypothetical protein
Anisa02854	PROKKA_02854 3-oxo-5-alpha-steroid 4-dehydrogenase
Anisa02857	PROKKA_02857 hypothetical protein
Anisa02885	PROKKA_02885 Formamidase

Anisa02999	PROKKA_02999 hypothetical protein
Anisa03204	PROKKA_03204 hypothetical protein
Anisa03472	PROKKA_03472 hypothetical protein
Anisa03568	PROKKA_03568 hypothetical protein
Anisa03624	PROKKA_03624 Cell cycle control protein
Anisa03628	PROKKA_03628 hypothetical protein
Anisa03665	PROKKA_03665 Putative penicillin-binding protein PbpX
Anisa03773	PROKKA_03773 hypothetical protein
Anisa03798	PROKKA_03798 hypothetical protein
Cincinnati00544	PROKKA_00548 hypothetical protein
Cincinnati01215	PROKKA_01228 Exo-beta-1,3-glucanase
Cincinnati01376	PROKKA_01390 hypothetical protein
Cincinnati01578	PROKKA_01594 Aspartyl/Asparaginyl beta-hydroxylase
Cincinnati01599	PROKKA_01615 hypothetical protein
Cincinnati01709	PROKKA_01725 hypothetical protein
Cincinnati02048	PROKKA_02067 Exopolysaccharide synthesis, ExoD
Cincinnati02420	PROKKA_02442 universal stress protein UspE
Cincinnati02807	PROKKA_02834 Ribonuclease Z
Cincinnati03061	PROKKA_03096 hypothetical protein
Cincinnati03089	PROKKA_03124 phosphoglycerol transferase I
Cincinnati03201	PROKKA_03237 putative metal-dependent hydrolase of the TIM-barrel fold protein
Cincinnati03248	PROKKA_03284 hypothetical protein
Cincinnati03470	PROKKA_03506 hypothetical protein
NSW150_00025	NSW150_00025 Transposase DDE domain protein
NSW150_00221	NSW150_00221 hypothetical protein
NSW150_01689	NSW150_01719 hypothetical protein
NSW150_01931	NSW150_01963 hypothetical protein
NSW150_01949	NSW150_01981 hypothetical protein
NSW150_02008	NSW150_02041 Opacity protein antigens
NSW150_02114	NSW150_02151 hypothetical protein

NSW150_02163	NSW150_02200 TM2 domain-containing protein
NSW150_02987	NSW150_03030 MgtC family protein
NSW150_03274	NSW150_03317 hypothetical protein
NSW150_03381	NSW150_03427 L-asparagine oxygenase
NSW150_03383	NSW150_03429 aspartate racemase
NSW150_03384	NSW150_03430 hypothetical protein
NSW150_03385	NSW150_03431 Alanine-anticapsin ligase BacD
boze1_00259	PROKKA_00260 hypothetical protein
boze1_00274	PROKKA_00275 hypothetical protein
boze1_00276	PROKKA_00277 Phenylacetate-coenzyme A ligase
boze1_00536	PROKKA_00537 Universal stress protein family protein
boze1_00605	PROKKA_00606 Putative DNA ligase-like protein/MT0965
boze1_01861	PROKKA_01872 hypothetical protein
boze1_02106	PROKKA_02129 hypothetical protein
boze1_02163	PROKKA_02186 hypothetical protein
boze1_02190	PROKKA_02213 hypothetical protein
boze1_02209	PROKKA_02232 Malonyl-CoA O-methyltransferase BioC
boze1_02284	PROKKA_02307 multidrug efflux protein
boze1_02690	PROKKA_02718 Undecaprenyl-phosphate 4-deoxy-4-formamido-L-arabinose transferase
boze1_02691	PROKKA_02719 Acyl-[acyl-carrier-protein]--UDP-N-acetylglucosamine O-acyltransferase
boze1_02943	PROKKA_02972 hypothetical protein
boze1_03113	PROKKA_03142 Phosphate-selective porin
boze1_03446	PROKKA_03476 hypothetical protein
boze1_03447	PROKKA_03477 hypothetical protein
boze1_03518	PROKKA_03553 hypothetical protein
boze1_03520	PROKKA_03555 Cellulase (glycosyl hydrolase family 5)
boze1_03556	PROKKA_03591 Phthiotriol/phenolphthiotriol dimycocerosates methyltransferase
boze2_03239	bozeman_2_D4398_03277 hypothetical protein
cherii00020	PROKKA_00020 Putative fatty-acid--CoA ligase fadD21
cherii00173	PROKKA_00176 carbamoyl phosphate synthase small subunit

cherii00192	PROKKA_00195 hypothetical protein
cherii00455	PROKKA_00461 Benzene 1,2-dioxygenase subunit alpha
cherii01091	PROKKA_01103 hypothetical protein
cherii01910	PROKKA_01928 hypothetical protein
cherii02248	PROKKA_02280 hypothetical protein
cherii02249	PROKKA_02281 hypothetical protein
cherii02380	PROKKA_02412 putative GTPase
cherii02631	PROKKA_02665 hypothetical protein
cherii02745	PROKKA_02780 Restriction endonuclease
wadswor01374	wadswor_01401 hypothetical protein
Anisa00367	PROKKA_00367 hypothetical protein
Anisa00456	PROKKA_00456 hypothetical protein
Anisa00508	PROKKA_00508 hypothetical protein
Anisa00526	PROKKA_00526 hypothetical protein
Anisa00715	PROKKA_00715 HAD hydrolase
Anisa00876	PROKKA_00876 Sel1 repeat
Anisa00928	PROKKA_00928 hypothetical protein
Anisa01605	PROKKA_01605 hypothetical protein
Anisa01771	PROKKA_01771 ankyrin repeat protein
Anisa01874	PROKKA_01874 hypothetical protein
Anisa01877	PROKKA_01877 hypothetical protein
Anisa02066	PROKKA_02066 Cold acclimation protein B
Anisa02078	PROKKA_02078 Hin recombinational enhancer-binding protein
Anisa02084	PROKKA_02084 Exodeoxyribonuclease V alpha chain
Anisa02160	PROKKA_02160 hypothetical protein
Anisa02172	PROKKA_02172 small GTP-binding protein domain protein
Anisa02332	PROKKA_02332 hypothetical protein
Anisa02362	PROKKA_02362 hypothetical protein
Anisa02371	PROKKA_02371 hypothetical protein
Anisa02568	PROKKA_02568 hypothetical protein

Anisa02588	PROKKA_02588 hypothetical protein
Anisa02628	PROKKA_02628 putative PEP-CTERM system TPR-repeat lipoprotein
Anisa02631	PROKKA_02631 Phosphomannomutase/phosphoglucomutase
Anisa02633	PROKKA_02633 Endoglucanase A precursor
Anisa02693	PROKKA_02693 ATP-dependent dethiobiotin synthetase BioD 1
Anisa02707	PROKKA_02707 Lipoprotein p23
Anisa02824	PROKKA_02824 hypothetical protein
Anisa02852	PROKKA_02852 hypothetical protein
Anisa02858	PROKKA_02858 hypothetical protein
Anisa03174	PROKKA_03174 hypothetical protein
Anisa03175	PROKKA_03175 hypothetical protein
Anisa03179	PROKKA_03179 hypothetical protein
Anisa03202	PROKKA_03202 hypothetical protein
Anisa03250	PROKKA_03250 hypothetical protein
Anisa03287	PROKKA_03287 hypothetical protein
Anisa03288	PROKKA_03288 Phthiotriol/phenolphthiotriol dimycocerosates methyltransferase
Anisa03307	PROKKA_03307 hypothetical protein
Anisa03394	PROKKA_03394 hypothetical protein
Anisa03402	PROKKA_03402 2-hydroxy-6-oxononadienedioate/2-hydroxy-6-oxononatrienedioate hydrolase
Anisa03446	PROKKA_03446 hypothetical protein
Anisa03473	PROKKA_03473 Organic hydroperoxide resistance protein OhrA
Anisa03481	PROKKA_03481 hypothetical protein
Anisa03488	PROKKA_03488 hypothetical protein
Anisa03569	PROKKA_03569 hypothetical protein
Anisa03613	PROKKA_03613 hypothetical protein
Anisa03614	PROKKA_03614 hypothetical protein
Anisa03653	PROKKA_03653 hypothetical protein
Anisa03782	PROKKA_03782 Cytochrome c oxidase subunit III
Anisa03824	PROKKA_03824 Transposase DDE domain protein
Cincinnati00020	PROKKA_00020 hypothetical protein

Cincinnati00033	PROKKA_00033 hypothetical protein
Cincinnati00034	PROKKA_00034 hypothetical protein
Cincinnati00035	PROKKA_00035 hypothetical protein
Cincinnati00036	PROKKA_00036 hypothetical protein
Cincinnati00094	PROKKA_00094 hypothetical protein
Cincinnati00179	PROKKA_00179 murein transglycosylase A
Cincinnati00191	PROKKA_00191 hypothetical protein
Cincinnati00193	PROKKA_00193 hypothetical protein
Cincinnati00228	PROKKA_00228 hypothetical protein
Cincinnati00261	PROKKA_00261 hypothetical protein
Cincinnati00271	PROKKA_00271 hypothetical protein
Cincinnati00273	PROKKA_00273 hypothetical protein
Cincinnati00277	PROKKA_00277 hypothetical protein
Cincinnati00313	PROKKA_00313 hypothetical protein
Cincinnati00418	PROKKA_00418 hypothetical protein
Cincinnati00541	PROKKA_00545 hypothetical protein
Cincinnati00602	PROKKA_00607 hypothetical protein
Cincinnati00608	PROKKA_00613 hypothetical protein
Cincinnati00613	PROKKA_00618 hypothetical protein
Cincinnati00778	PROKKA_00784 Adenylate cyclase 1
Cincinnati00779	PROKKA_00785 hypothetical protein
Cincinnati00781	PROKKA_00787 hypothetical protein
Cincinnati00866	PROKKA_00875 hypothetical protein
Cincinnati00891	PROKKA_00900 hypothetical protein
Cincinnati01007	PROKKA_01018 Phosphocholine transferase AnkX
Cincinnati01015	PROKKA_01026 Rubredoxin
Cincinnati01062	PROKKA_01073 hypothetical protein
Cincinnati01075	PROKKA_01086 hypothetical protein
Cincinnati01076	PROKKA_01087 Chaperone protein DnaJ
Cincinnati01089	PROKKA_01100 L-2,4-diaminobutyrate decarboxylase

Cincinnati01125	PROKKA_01137 hypothetical protein
Cincinnati01187	PROKKA_01200 hypothetical protein
Cincinnati01207	PROKKA_01220 hypothetical protein
Cincinnati01229	PROKKA_01242 Sensor protein EvgS precursor
Cincinnati01261	PROKKA_01274 T-complex protein 11
Cincinnati01278	PROKKA_01291 hypothetical protein
Cincinnati01401	PROKKA_01415 hypothetical protein
Cincinnati01474	PROKKA_01490 hypothetical protein
Cincinnati01504	PROKKA_01520 hypothetical protein
Cincinnati01518	PROKKA_01534 hypothetical protein
Cincinnati01527	PROKKA_01543 hypothetical protein
Cincinnati01579	PROKKA_01595 Formyltetrahydrofolate deformylase
Cincinnati01673	PROKKA_01689 hypothetical protein
Cincinnati01674	PROKKA_01690 hypothetical protein
Cincinnati01712	PROKKA_01728 ankyrin repeat protein
Cincinnati01731	PROKKA_01748 hypothetical protein
Cincinnati01732	PROKKA_01749 hypothetical protein
Cincinnati01733	PROKKA_01750 hypothetical protein
Cincinnati01734	PROKKA_01751 Zeta toxin
Cincinnati01743	PROKKA_01760 Opacity protein antigens
Cincinnati01774	PROKKA_01791 Bicarbonate transporter BicA
Cincinnati01827	PROKKA_01844 hypothetical protein
Cincinnati01852	PROKKA_01869 hypothetical protein
Cincinnati01871	PROKKA_01889 hypothetical protein
Cincinnati01873	PROKKA_01891 hypothetical protein
Cincinnati01921	PROKKA_01939 hypothetical protein
Cincinnati01943	PROKKA_01961 Tyrocidine synthase III
Cincinnati01946	PROKKA_01964 Putative ABC transporter substrate-binding lipoprotein YhfQ precursor
Cincinnati01948	PROKKA_01966 Iron(III)-hydroxamate import system permease protein FhuB
Cincinnati01949	PROKKA_01967 putative siderophore transport system ATP-binding protein YusV

Cincinnati01976	PROKKA_01994 pentatricopeptide repeat domain protein
Cincinnati01980	PROKKA_01998 hypothetical protein
Cincinnati01998	PROKKA_02017 hypothetical protein
Cincinnati02029	PROKKA_02048 hypothetical protein
Cincinnati02030	PROKKA_02049 ankyrin repeat protein
Cincinnati02139	PROKKA_02158 hypothetical protein
Cincinnati02241	PROKKA_02263 hypothetical protein
Cincinnati02255	PROKKA_02277 hypothetical protein
Cincinnati02258	PROKKA_02280 hypothetical protein
Cincinnati02264	PROKKA_02286 HTH-type transcriptional regulator GltC
Cincinnati02265	PROKKA_02287 FkbH domain protein
Cincinnati02266	PROKKA_02288 hypothetical protein
Cincinnati02284	PROKKA_02306 Zeta toxin
Cincinnati02285	PROKKA_02307 hypothetical protein
Cincinnati02296	PROKKA_02318 hypothetical protein
Cincinnati02322	PROKKA_02344 hypothetical protein
Cincinnati02324	PROKKA_02346 Retron-type reverse transcriptase
Cincinnati02331	PROKKA_02353 hypothetical protein
Cincinnati02408	PROKKA_02430 Cyclic di-GMP phosphodiesterase Gmr
Cincinnati02409	PROKKA_02431 hypothetical protein
Cincinnati02472	PROKKA_02495 hypothetical protein
Cincinnati02473	PROKKA_02496 ABC transporter arginine-binding protein 1 precursor
Cincinnati02515	PROKKA_02540 Protein kinase domain protein
Cincinnati02528	PROKKA_02553 hypothetical protein
Cincinnati02563	PROKKA_02588 hypothetical protein
Cincinnati02577	PROKKA_02602 Putative oxidoreductasec/MT0587
Cincinnati02590	PROKKA_02616 hypothetical protein
Cincinnati02619	PROKKA_02645 hypothetical protein
Cincinnati02638	PROKKA_02664 Trans-aconitate 2-methyltransferase
Cincinnati02669	PROKKA_02695 Pyoverdine/dityrosine biosynthesis protein

Cincinnati02770	PROKKA_02796 hypothetical protein
Cincinnati02772	PROKKA_02798 hypothetical protein
Cincinnati02773	PROKKA_02799 hypothetical protein
Cincinnati02805	PROKKA_02832 hypothetical protein
Cincinnati02808	PROKKA_02835 hypothetical protein
Cincinnati02810	PROKKA_02837 hypothetical protein
Cincinnati02823	PROKKA_02850 hypothetical protein
Cincinnati02856	PROKKA_02883 hypothetical protein
Cincinnati02864	PROKKA_02891 hypothetical protein
Cincinnati02881	PROKKA_02909 hypothetical protein
Cincinnati02882	PROKKA_02910 hypothetical protein
Cincinnati02883	PROKKA_02911 YciI-like protein
Cincinnati02885	PROKKA_02913 hypothetical protein
Cincinnati02886	PROKKA_02914 Ankyrin repeats (3 copies)
Cincinnati02887	PROKKA_02915 hypothetical protein
Cincinnati02890	PROKKA_02918 hypothetical protein
Cincinnati02975	PROKKA_03003 hypothetical protein
Cincinnati02997	PROKKA_03025 hypothetical protein
Cincinnati03026	PROKKA_03061 hypothetical protein
Cincinnati03029	PROKKA_03064 hypothetical protein
Cincinnati03075	PROKKA_03110 hypothetical protein
Cincinnati03093	PROKKA_03128 hypothetical protein
Cincinnati03132	PROKKA_03167 chromosome segregation protein
Cincinnati03143	PROKKA_03178 Phosphocholine transferase AnkX
Cincinnati03155	PROKKA_03190 hypothetical protein
Cincinnati03156	PROKKA_03191 hypothetical protein
Cincinnati03185	PROKKA_03221 hypothetical protein
Cincinnati03200	PROKKA_03236 L-arabinolactonase
Cincinnati03206	PROKKA_03242 Stress responsive A/B Barrel Domain protein
Cincinnati03222	PROKKA_03258 Alanine racemase

Cincinnati03223	PROKKA_03259 hypothetical protein
Cincinnati03294	PROKKA_03330 hypothetical protein
Cincinnati03302	PROKKA_03338 hypothetical protein
Cincinnati03359	PROKKA_03395 aerobic sulfatase maturase family protein
Cincinnati03384	PROKKA_03420 hypothetical protein
Cincinnati03395	PROKKA_03431 hypothetical protein
Cincinnati03397	PROKKA_03433 hypothetical protein
Cincinnati03411	PROKKA_03447 hypothetical protein
Cincinnati03439	PROKKA_03475 hypothetical protein
Cincinnati03469	PROKKA_03505 hypothetical protein
Cincinnati03471	PROKKA_03507 pentatricopeptide repeat domain protein
Cincinnati03476	PROKKA_03512 hypothetical protein
Cincinnati03480	PROKKA_03516 hypothetical protein
NSW150_00022	NSW150_00022 Integrase core domain protein
NSW150_00023	NSW150_00023 hypothetical protein
NSW150_00225	NSW150_00225 Demethylrebeccamycin-D-glucose O-methyltransferase
NSW150_00227	NSW150_00227 hypothetical protein
NSW150_00228	NSW150_00228 Oxidoreductase family, NAD-binding Rossmann fold
NSW150_01189	NSW150_01206 hypothetical protein
NSW150_01191	NSW150_01208 hypothetical protein
NSW150_01345	NSW150_01374 hypothetical protein
NSW150_01368	NSW150_01397 hypothetical protein
NSW150_01637	NSW150_01667 hypothetical protein
NSW150_01655	NSW150_01685 ankyrin repeat protein
NSW150_01893	NSW150_01925 Transposase DDE domain protein
NSW150_02000	NSW150_02033 Protein tyrosine/serine phosphatase
NSW150_02260	NSW150_02297 hypothetical protein
NSW150_02419	NSW150_02457 hypothetical protein
NSW150_02882	NSW150_02925 hypothetical protein
NSW150_02937	NSW150_02980 hypothetical protein

NSW150_02944	NSW150_02987 Glutathionyl-hydroquinone reductase YqjG
NSW150_02946	NSW150_02989 Inner membrane protein YbaL
NSW150_02947	NSW150_02990 LigB family dioxygenase
NSW150_02949	NSW150_02992 hypothetical protein
NSW150_02957	NSW150_03000 hypothetical protein
NSW150_02986	NSW150_03029 hypothetical protein
NSW150_03127	NSW150_03170 Ran GTPase-activating protein (RanGAP) involved in mRNA processing and transport
NSW150_03128	NSW150_03171 hypothetical protein
NSW150_03173	NSW150_03216 hypothetical protein
NSW150_03246	NSW150_03289 Mannosyltransferase OCH1
boze1_00271	PROKKA_00272 hypothetical protein
boze1_00323	PROKKA_00324 hypothetical protein
boze1_00385	PROKKA_00386 hypothetical protein
boze1_00436	PROKKA_00437 hypothetical protein
boze1_00468	PROKKA_00469 Zinc-type alcohol dehydrogenase-like protein
boze1_00471	PROKKA_00472 putative sulfate transport protein CysZ
boze1_00514	PROKKA_00515 hypothetical protein
boze1_00521	PROKKA_00522 hypothetical protein
boze1_00578	PROKKA_00579 hypothetical protein
boze1_00596	PROKKA_00597 hypothetical protein
boze1_00621	PROKKA_00623 hypothetical protein
boze1_00650	PROKKA_00652 hypothetical protein
boze1_00651	PROKKA_00653 hypothetical protein
boze1_00728	PROKKA_00731 hypothetical protein
boze1_00801	PROKKA_00804 hypothetical protein
boze1_00833	PROKKA_00837 hypothetical protein
boze1_00838	PROKKA_00842 hypothetical protein
boze1_00922	PROKKA_00928 hypothetical protein
boze1_01048	PROKKA_01054 hypothetical protein
boze1_01136	PROKKA_01143 hypothetical protein

boze1_01138	PROKKA_01145 hypothetical protein
boze1_01139	PROKKA_01146 hypothetical protein
boze1_01183	PROKKA_01191 hypothetical protein
boze1_01277	PROKKA_01285 hypothetical protein
boze1_01302	PROKKA_01310 hypothetical protein
boze1_01311	PROKKA_01319 hypothetical protein
boze1_01326	PROKKA_01334 hypothetical protein
boze1_01347	PROKKA_01355 hypothetical protein
boze1_01348	PROKKA_01356 hypothetical protein
boze1_01354	PROKKA_01362 hypothetical protein
boze1_01361	PROKKA_01369 putative manganese-dependent inorganic pyrophosphatase
boze1_01373	PROKKA_01381 hypothetical protein
boze1_01446	PROKKA_01455 Ras family protein
boze1_01506	PROKKA_01516 polyhydroxyalkanoate depolymerase, intracellular
boze1_01521	PROKKA_01532 hypothetical protein
boze1_01523	PROKKA_01534 hypothetical protein
boze1_01528	PROKKA_01539 hypothetical protein
boze1_01564	PROKKA_01575 hypothetical protein
boze1_01616	PROKKA_01627 Opacity protein antigens
boze1_01653	PROKKA_01664 hypothetical protein
boze1_01675	PROKKA_01686 Pyridine nucleotide-disulphide oxidoreductase
boze1_01681	PROKKA_01692 hypothetical protein
boze1_01699	PROKKA_01710 putative ATP-grasp enzyme
boze1_01751	PROKKA_01762 hypothetical protein
boze1_01786	PROKKA_01797 hypothetical protein
boze1_01951	PROKKA_01962 hypothetical protein
boze1_01969	PROKKA_01982 hypothetical protein
boze1_01991	PROKKA_02004 hypothetical protein
boze1_02036	PROKKA_02052 hypothetical protein
boze1_02137	PROKKA_02160 hypothetical protein

boze1_02157	PROKKA_02180 hypothetical protein
boze1_02166	PROKKA_02189 disulfide isomerase/thiol-disulfide oxidase
boze1_02168	PROKKA_02191 hypothetical protein
boze1_02173	PROKKA_02196 hypothetical protein
boze1_02175	PROKKA_02198 hypothetical protein
boze1_02176	PROKKA_02199 DNA-binding transcriptional activator PspC
boze1_02184	PROKKA_02207 hypothetical protein
boze1_02189	PROKKA_02212 hypothetical protein
boze1_02195	PROKKA_02218 hypothetical protein
boze1_02207	PROKKA_02230 hypothetical protein
boze1_02211	PROKKA_02234 hypothetical protein
boze1_02238	PROKKA_02261 hypothetical protein
boze1_02270	PROKKA_02293 hypothetical protein
boze1_02299	PROKKA_02322 hypothetical protein
boze1_02334	PROKKA_02357 hypothetical protein
boze1_02337	PROKKA_02360 hypothetical protein
boze1_02381	PROKKA_02405 hypothetical protein
boze1_02446	PROKKA_02473 hypothetical protein
boze1_02530	PROKKA_02558 Type III restriction enzyme, res subunit
boze1_02531	PROKKA_02559 Adenine specific DNA methylase Mod
boze1_02547	PROKKA_02575 hypothetical protein
boze1_02555	PROKKA_02583 hypothetical protein
boze1_02558	PROKKA_02586 hypothetical protein
boze1_02561	PROKKA_02589 hypothetical protein
boze1_02617	PROKKA_02645 hypothetical protein
boze1_02625	PROKKA_02653 hypothetical protein
boze1_02626	PROKKA_02654 hypothetical protein
boze1_02631	PROKKA_02659 hypothetical protein
boze1_02633	PROKKA_02661 Na(+)-translocating NADH-quinone reductase subunit F
boze1_02638	PROKKA_02666 hypothetical protein

boze1_02644	PROKKA_02672 Glutamyl-tRNA reductase
boze1_02645	PROKKA_02673 Group 3 truncated hemoglobin ctb
boze1_02652	PROKKA_02680 hypothetical protein
boze1_02654	PROKKA_02682 hypothetical protein
boze1_02667	PROKKA_02695 Superfamily II helicase
boze1_02668	PROKKA_02696 hypothetical protein
boze1_02670	PROKKA_02698 hypothetical protein
boze1_02672	PROKKA_02700 hypothetical protein
boze1_02674	PROKKA_02702 hypothetical protein
boze1_02687	PROKKA_02715 hypothetical protein
boze1_02688	PROKKA_02716 hypothetical protein
boze1_02738	PROKKA_02767 hypothetical protein
boze1_02739	PROKKA_02768 hypothetical protein
boze1_02758	PROKKA_02787 hypothetical protein
boze1_02770	PROKKA_02799 hypothetical protein
boze1_02783	PROKKA_02812 hypothetical protein
boze1_02790	PROKKA_02819 hypothetical protein
boze1_02866	PROKKA_02895 Linear gramicidin synthase subunit D
boze1_02879	PROKKA_02908 hypothetical protein
boze1_02976	PROKKA_03005 bifunctional 3-demethylubiquinone-9 3-methyltransferase/ 2-octaprenyl-6-hydroxy phenol m[illegible]
boze1_03021	PROKKA_03050 arylsulfatase
boze1_03022	PROKKA_03051 Phosphoglycerol transferase, alkaline phosphatase superfamily
boze1_03031	PROKKA_03060 hypothetical protein
boze1_03032	PROKKA_03061 hypothetical protein
boze1_03071	PROKKA_03100 U-box domain protein
boze1_03076	PROKKA_03105 Exoenzyme S synthesis regulatory protein ExsA
boze1_03078	PROKKA_03107 hypothetical protein
boze1_03109	PROKKA_03138 Pseudomonalisin precursor
boze1_03110	PROKKA_03139 Methionine aminopeptidase
boze1_03112	PROKKA_03141 hypothetical protein

boze1_03117	PROKKA_03146 hypothetical protein
boze1_03126	PROKKA_03155 tellurite resistance protein TehB
boze1_03140	PROKKA_03169 hypothetical protein
boze1_03143	PROKKA_03172 hypothetical protein
boze1_03145	PROKKA_03174 hypothetical protein
boze1_03146	PROKKA_03175 Nodulation protein D 2
boze1_03153	PROKKA_03182 hypothetical protein
boze1_03170	PROKKA_03199 hypothetical protein
boze1_03237	PROKKA_03266 hypothetical protein
boze1_03279	PROKKA_03308 hypothetical protein
boze1_03284	PROKKA_03313 hypothetical protein
boze1_03290	PROKKA_03319 hypothetical protein
boze1_03294	PROKKA_03323 hypothetical protein
boze1_03346	PROKKA_03376 hypothetical protein
boze1_03347	PROKKA_03377 hypothetical protein
boze1_03383	PROKKA_03413 FRG domain protein
boze1_03385	PROKKA_03415 hypothetical protein
boze1_03386	PROKKA_03416 hypothetical protein
boze1_03388	PROKKA_03418 hypothetical protein
boze1_03389	PROKKA_03419 hypothetical protein
boze1_03416	PROKKA_03446 hypothetical protein
boze1_03418	PROKKA_03448 hypothetical protein
boze1_03421	PROKKA_03451 hypothetical protein
boze1_03422	PROKKA_03452 hypothetical protein
boze1_03425	PROKKA_03455 Superfamily II helicase
boze1_03427	PROKKA_03457 hypothetical protein
boze1_03442	PROKKA_03472 hypothetical protein
boze1_03449	PROKKA_03479 conjugal transfer pilus assembly protein TraL
boze1_03492	PROKKA_03524 hypothetical protein
boze1_03494	PROKKA_03526 ATP-dependent DNA helicase PcrA

boze1_03541	PROKKA_03576 hypothetical protein
boze1_03542	PROKKA_03577 hypothetical protein
boze1_03546	PROKKA_03581 hypothetical protein
boze1_03565	PROKKA_03600 hypothetical protein
boze1_03569	PROKKA_03604 putative integral membrane protein
boze1_03590	PROKKA_03625 hypothetical protein
boze1_03600	PROKKA_03635 hypothetical protein
boze1_03602	PROKKA_03637 hypothetical protein
boze1_03607	PROKKA_03642 hypothetical protein
boze1_03611	PROKKA_03646 conjugal transfer protein TraV
boze1_03616	PROKKA_03651 flagellar biosynthesis regulator FlhF
boze2_00138	bozeman_2_D4398_00138 hypothetical protein
boze2_00795	bozeman_2_D4398_00798 hypothetical protein
boze2_02232	bozeman_2_D4398_02255 hypothetical protein
boze2_02262	bozeman_2_D4398_02285 Leucine--tRNA ligase
boze2_03182	bozeman_2_D4398_03220 hypothetical protein
boze2_03238	bozeman_2_D4398_03276 hypothetical protein
cherii00185	PROKKA_00188 hypothetical protein
cherii00387	PROKKA_00393 hypothetical protein
cherii00454	PROKKA_00460 hypothetical protein
cherii00487	PROKKA_00493 bifunctional proline dehydrogenase/pyrroline-5-carboxylate dehydrogenase
cherii00589	PROKKA_00595 hypothetical protein
cherii00902	PROKKA_00913 hypothetical protein
cherii01267	PROKKA_01279 hypothetical protein
cherii01270	PROKKA_01282 hypothetical protein
cherii01367	PROKKA_01379 hypothetical protein
cherii01437	PROKKA_01451 hypothetical protein
cherii01626	PROKKA_01642 hypothetical protein
cherii01772	PROKKA_01789 Antibiotic efflux pump outer membrane protein ArpC precursor
cherii01773	PROKKA_01790 p-hydroxybenzoic acid efflux pump subunit AaeA

cherii01774	PROKKA_01791 Lipoprotein-releasing system ATP-binding protein LolD
cherii01900	PROKKA_01918 hypothetical protein
cherii02222	PROKKA_02254 adenosine deaminase
cherii02353	PROKKA_02385 ankyrin repeat protein
cherii02371	PROKKA_02403 Protein kinase domain protein
cherii02423	PROKKA_02455 hypothetical protein
cherii02564	PROKKA_02598 hypothetical protein
cherii02569	PROKKA_02603 Stalked cell differentiation-controlling protein
cherii02646	PROKKA_02681 hypothetical protein
cherii02652	PROKKA_02687 hypothetical protein
cherii02658	PROKKA_02693 hypothetical protein
cherii03213	PROKKA_03250 hypothetical protein
dumof00147	PROKKA_00149 hypothetical protein
dumof00494	PROKKA_00508 hypothetical protein
dumof00525	PROKKA_00539 hypothetical protein
dumof00621	PROKKA_00635 putative murein peptide carboxypeptidase
dumof00732	PROKKA_00746 Peptidase C80 family protein
dumof01753	PROKKA_01775 hypothetical protein
dumof02045	PROKKA_02069 hypothetical protein
dumof02202	PROKKA_02226 hypothetical protein
dumof02219	PROKKA_02243 hypothetical protein
dumof02638	PROKKA_02664 hypothetical protein
dumof02651	PROKKA_02677 hypothetical protein
dumof03249	PROKKA_03285 hypothetical protein
Tucson01067	PROKKA_01086 hypothetical protein
Tucson01896	PROKKA_01920 L,D-transpeptidase catalytic domain
Tucson02605	PROKKA_02638 hypothetical protein
wadswor00826	wadswor_00835 hypothetical protein
Anisa00046	PROKKA_00046 putative acetyltransferase
Anisa00062	PROKKA_00062 hypothetical protein

Anisa00085	PROKKA_00085 hypothetical protein
Anisa00092	PROKKA_00092 hypothetical protein
Anisa00186	PROKKA_00186 hypothetical protein
Anisa00187	PROKKA_00187 hypothetical protein
Anisa00191	PROKKA_00191 hypothetical protein
Anisa00204	PROKKA_00204 hypothetical protein
Anisa00207	PROKKA_00207 hypothetical protein
Anisa00215	PROKKA_00215 hypothetical protein
Anisa00231	PROKKA_00231 hypothetical protein
Anisa00257	PROKKA_00257 hypothetical protein
Anisa00299	PROKKA_00299 hypothetical protein
Anisa00300	PROKKA_00300 Kua-ubiquitin conjugating enzyme hybrid localisation domain protein
Anisa00369	PROKKA_00369 hypothetical protein
Anisa00478	PROKKA_00478 hypothetical protein
Anisa00494	PROKKA_00494 hypothetical protein
Anisa00496	PROKKA_00496 hypothetical protein
Anisa00527	PROKKA_00527 hypothetical protein
Anisa00572	PROKKA_00572 Opacity protein antigens
Anisa00584	PROKKA_00584 hypothetical protein
Anisa00594	PROKKA_00594 Copper resistance protein B precursor
Anisa00604	PROKKA_00604 Proclavaminate amidinohydrolase
Anisa00616	PROKKA_00616 hypothetical protein
Anisa00621	PROKKA_00621 hypothetical protein
Anisa00626	PROKKA_00626 hypothetical protein
Anisa00627	PROKKA_00627 hypothetical protein
Anisa00725	PROKKA_00725 hypothetical protein
Anisa00765	PROKKA_00765 hypothetical protein
Anisa00769	PROKKA_00769 hypothetical protein
Anisa00770	PROKKA_00770 hypothetical protein
Anisa00771	PROKKA_00771 hypothetical protein

Anisa00784	PROKKA_00784 Putative beta-lactamase HcpC precursor
Anisa00785	PROKKA_00785 hypothetical protein
Anisa00871	PROKKA_00871 ribosomal-protein-alanine acetyltransferase
Anisa00885	PROKKA_00885 hypothetical protein
Anisa00886	PROKKA_00886 hypothetical protein
Anisa00888	PROKKA_00888 hypothetical protein
Anisa00889	PROKKA_00889 hypothetical protein
Anisa00933	PROKKA_00933 hypothetical protein
Anisa00936	PROKKA_00936 hypothetical protein
Anisa01011	PROKKA_01011 hypothetical protein
Anisa01012	PROKKA_01012 thiamine kinase
Anisa01155	PROKKA_01155 hypothetical protein
Anisa01278	PROKKA_01278 hypothetical protein
Anisa01280	PROKKA_01280 hypothetical protein
Anisa01503	PROKKA_01503 hypothetical protein
Anisa01504	PROKKA_01504 hypothetical protein
Anisa01584	PROKKA_01584 Tfp pilus assembly protein FimV
Anisa01606	PROKKA_01606 hypothetical protein
Anisa01608	PROKKA_01608 hypothetical protein
Anisa01624	PROKKA_01624 hypothetical protein
Anisa01627	PROKKA_01627 Fatty acid desaturase
Anisa01651	PROKKA_01651 D-tyrosyl-tRNA(Tyr) deacylase
Anisa01655	PROKKA_01655 hypothetical protein
Anisa01656	PROKKA_01656 hypothetical protein
Anisa01679	PROKKA_01679 hypothetical protein
Anisa01709	PROKKA_01709 hypothetical protein
Anisa01744	PROKKA_01744 hypothetical protein
Anisa01763	PROKKA_01763 Chemotaxis protein MotA
Anisa01817	PROKKA_01817 hypothetical protein
Anisa01858	PROKKA_01858 ankyrin repeat protein

Anisa01859	PROKKA_01859 hypothetical protein
Anisa01863	PROKKA_01863 hypothetical protein
Anisa01873	PROKKA_01873 hypothetical protein
Anisa01918	PROKKA_01918 putative urate catabolism protein
Anisa01919	PROKKA_01919 lactoylglutathione lyase
Anisa01920	PROKKA_01920 2-keto-3-deoxy-L-fuconate dehydrogenase
Anisa01921	PROKKA_01921 Carbon starvation induced regulator
Anisa01928	PROKKA_01928 chemotaxis-specific methylesterase
Anisa01929	PROKKA_01929 hypothetical protein
Anisa01958	PROKKA_01958 hypothetical protein
Anisa01965	PROKKA_01965 serine/threonine protein kinase
Anisa01982	PROKKA_01982 hypothetical protein
Anisa01989	PROKKA_01989 hypothetical protein
Anisa01999	PROKKA_01999 hypothetical protein
Anisa02056	PROKKA_02056 hypothetical protein
Anisa02069	PROKKA_02069 Recombination-associated protein RdgC
Anisa02070	PROKKA_02070 Helix-destabilizing protein
Anisa02071	PROKKA_02071 hypothetical protein
Anisa02121	PROKKA_02121 hypothetical protein
Anisa02124	PROKKA_02124 hypothetical protein
Anisa02130	PROKKA_02130 hypothetical protein
Anisa02134	PROKKA_02134 putative lyase
Anisa02170	PROKKA_02170 hypothetical protein
Anisa02219	PROKKA_02219 hypothetical protein
Anisa02278	PROKKA_02278 hypothetical protein
Anisa02308	PROKKA_02308 hypothetical protein
Anisa02309	PROKKA_02309 hypothetical protein
Anisa02319	PROKKA_02319 hypothetical protein
Anisa02321	PROKKA_02321 Cysteine desulfurase
Anisa02334	PROKKA_02334 hypothetical protein

Anisa02356	PROKKA_02356 hypothetical protein
Anisa02363	PROKKA_02363 Cholesterol oxidase
Anisa02364	PROKKA_02364 Endonuclease/Exonuclease/phosphatase family protein
Anisa02365	PROKKA_02365 hypothetical protein
Anisa02376	PROKKA_02376 hypothetical protein
Anisa02377	PROKKA_02377 hypothetical protein
Anisa02381	PROKKA_02381 hypothetical protein
Anisa02382	PROKKA_02382 hypothetical protein
Anisa02389	PROKKA_02389 hypothetical protein
Anisa02397	PROKKA_02397 hypothetical protein
Anisa02399	PROKKA_02399 hypothetical protein
Anisa02431	PROKKA_02431 hypothetical protein
Anisa02447	PROKKA_02447 type 2 lantibiotic biosynthesis protein LanM
Anisa02493	PROKKA_02493 hypothetical protein
Anisa02494	PROKKA_02494 hypothetical protein
Anisa02496	PROKKA_02496 hypothetical protein
Anisa02498	PROKKA_02498 hypothetical protein
Anisa02501	PROKKA_02501 hypothetical protein
Anisa02503	PROKKA_02503 Carbon storage regulator
Anisa02512	PROKKA_02512 conjugal transfer protein TrbG
Anisa02514	PROKKA_02514 conjugal transfer protein TrbJ
Anisa02519	PROKKA_02519 hypothetical protein
Anisa02520	PROKKA_02520 glycosyltransferase, MGT family
Anisa02521	PROKKA_02521 3-oxoacyl-[acyl-carrier-protein] synthase 3
Anisa02522	PROKKA_02522 putative oxidoreductase
Anisa02523	PROKKA_02523 hypothetical protein
Anisa02525	PROKKA_02525 hypothetical protein
Anisa02526	PROKKA_02526 hypothetical protein
Anisa02528	PROKKA_02528 hypothetical protein
Anisa02535	PROKKA_02535 Transposase

Anisa02536	PROKKA_02536 Transposase
Anisa02538	PROKKA_02538 putative membrane protein
Anisa02539	PROKKA_02539 Macrolide export ATP-binding/permease protein MacB
Anisa02540	PROKKA_02540 Macrolide export ATP-binding/permease protein MacB
Anisa02541	PROKKA_02541 Macrolide-specific efflux protein MacA precursor
Anisa02542	PROKKA_02542 hypothetical protein
Anisa02544	PROKKA_02544 hypothetical protein
Anisa02579	PROKKA_02579 hypothetical protein
Anisa02580	PROKKA_02580 ankyrin repeat protein
Anisa02581	PROKKA_02581 hypothetical protein
Anisa02586	PROKKA_02586 hypothetical protein
Anisa02621	PROKKA_02621 hypothetical protein
Anisa02622	PROKKA_02622 Transposase
Anisa02627	PROKKA_02627 hypothetical protein
Anisa02655	PROKKA_02655 Peptidase S24-like protein
Anisa02695	PROKKA_02695 Phosphogluconate dehydratase
Anisa02706	PROKKA_02706 putative diguanylate cyclase YegE
Anisa02708	PROKKA_02708 Ferrochelatase
Anisa02709	PROKKA_02709 hypothetical protein
Anisa02714	PROKKA_02714 hypothetical protein
Anisa02715	PROKKA_02715 hypothetical protein
Anisa02747	PROKKA_02747 hypothetical protein
Anisa02756	PROKKA_02756 hypothetical protein
Anisa02757	PROKKA_02757 hypothetical protein
Anisa02761	PROKKA_02761 hypothetical protein
Anisa02771	PROKKA_02771 hypothetical protein
Anisa02772	PROKKA_02772 hypothetical protein
Anisa02773	PROKKA_02773 hypothetical protein
Anisa02774	PROKKA_02774 Adenylate and Guanylate cyclase catalytic domain protein
Anisa02778	PROKKA_02778 hypothetical protein

Anisa02799	PROKKA_02799 hypothetical protein
Anisa02821	PROKKA_02821 hypothetical protein
Anisa02822	PROKKA_02822 hypothetical protein
Anisa02823	PROKKA_02823 hypothetical protein
Anisa02826	PROKKA_02826 hypothetical protein
Anisa02828	PROKKA_02828 putative hydrolase
Anisa02831	PROKKA_02831 hypothetical protein
Anisa02837	PROKKA_02837 serine/threonine kinase US3
Anisa02847	PROKKA_02847 hypothetical protein
Anisa02856	PROKKA_02856 hypothetical protein
Anisa02859	PROKKA_02859 Cold shock protein CspV
Anisa02860	PROKKA_02860 Cold shock protein CspV
Anisa02861	PROKKA_02861 hypothetical protein
Anisa02873	PROKKA_02873 Alkaline phosphatase precursor
Anisa02874	PROKKA_02874 hypothetical protein
Anisa02878	PROKKA_02878 L-Ala-D/L-Glu epimerase
Anisa02888	PROKKA_02888 hypothetical protein
Anisa02889	PROKKA_02889 hypothetical protein
Anisa02890	PROKKA_02890 putative diguanylate cyclase YegE
Anisa02898	PROKKA_02898 hypothetical protein
Anisa02936	PROKKA_02936 Dipeptide and tripeptide permease A
Anisa02943	PROKKA_02943 hypothetical protein
Anisa03006	PROKKA_03006 hypothetical protein
Anisa03007	PROKKA_03007 hypothetical protein
Anisa03009	PROKKA_03009 putative cadmium-transporting ATPase
Anisa03037	PROKKA_03037 hypothetical protein
Anisa03056	PROKKA_03056 hypothetical protein
Anisa03078	PROKKA_03078 hypothetical protein
Anisa03178	PROKKA_03178 hypothetical protein
Anisa03195	PROKKA_03195 hypothetical protein

Anisa03197	PROKKA_03197 hypothetical protein
Anisa03219	PROKKA_03219 Cation efflux system protein CusA
Anisa03222	PROKKA_03222 hypothetical protein
Anisa03303	PROKKA_03303 Putative NADH-flavin reductase
Anisa03304	PROKKA_03304 Cupin domain protein
Anisa03310	PROKKA_03310 Penicillin-binding protein E
Anisa03336	PROKKA_03336 Xylulose-5-phosphate phosphoketolase
Anisa03350	PROKKA_03350 hypothetical protein
Anisa03399	PROKKA_03399 FRG domain protein
Anisa03403	PROKKA_03403 Acyltransferase family protein
Anisa03447	PROKKA_03447 F-box-like protein
Anisa03462	PROKKA_03462 Putative epoxidase LasC
Anisa03474	PROKKA_03474 hypothetical protein
Anisa03475	PROKKA_03475 hypothetical protein
Anisa03479	PROKKA_03479 hypothetical protein
Anisa03482	PROKKA_03482 conjugative transfer relaxase protein TraI
Anisa03532	PROKKA_03532 cryptochrome, DASH family
Anisa03558	PROKKA_03558 ABC transporter ATP-binding protein uup
Anisa03560	PROKKA_03560 hypothetical protein
Anisa03562	PROKKA_03562 Ankyrin repeat
Anisa03565	PROKKA_03565 hypothetical protein
Anisa03566	PROKKA_03566 hypothetical protein
Anisa03570	PROKKA_03570 hypothetical protein
Anisa03577	PROKKA_03577 hypothetical protein
Anisa03581	PROKKA_03581 hypothetical protein
Anisa03582	PROKKA_03582 hypothetical protein
Anisa03584	PROKKA_03584 hypothetical protein
Anisa03615	PROKKA_03615 hypothetical protein
Anisa03616	PROKKA_03616 multidrug resistance protein D
Anisa03626	PROKKA_03626 hypothetical protein

Anisa03640	PROKKA_03640 F-type ATPase subunit b
Anisa03641	PROKKA_03641 Lipid-binding protein
Anisa03642	PROKKA_03642 F-ATPase subunit 6
Anisa03643	PROKKA_03643 F1/F0 ATPase, Methanosarcina type, subunit 2
Anisa03644	PROKKA_03644 putative F0F1-ATPase subunit
Anisa03645	PROKKA_03645 F0F1 ATP synthase subunit epsilon
Anisa03646	PROKKA_03646 ATP synthase subunit beta
Anisa03650	PROKKA_03650 Ribonuclease
Anisa03651	PROKKA_03651 hypothetical protein
Anisa03656	PROKKA_03656 hypothetical protein
Anisa03662	PROKKA_03662 Methionine aminopeptidase
Anisa03664	PROKKA_03664 hypothetical protein
Anisa03673	PROKKA_03673 hypothetical protein
Anisa03675	PROKKA_03675 F-ATPase gamma subunit
Anisa03676	PROKKA_03676 putative bifunctional cbb3-type cytochrome c oxidase subunit II/cytochrome c
Anisa03678	PROKKA_03678 Cation efflux system protein CusB precursor
Anisa03682	PROKKA_03682 Propionate kinase
Anisa03683	PROKKA_03683 Enoyl-[acyl-carrier-protein] reductase [NADH] FabI
Anisa03686	PROKKA_03686 hypothetical protein
Anisa03699	PROKKA_03699 hypothetical protein
Anisa03705	PROKKA_03705 hypothetical protein
Anisa03707	PROKKA_03707 hypothetical protein
Anisa03721	PROKKA_03721 hypothetical protein
Anisa03722	PROKKA_03722 hypothetical protein
Anisa03729	PROKKA_03729 type I secretion outer membrane protein, TolC family
Anisa03736	PROKKA_03736 hypothetical protein
Anisa03738	PROKKA_03738 Phosphogluconate dehydratase
Anisa03741	PROKKA_03741 hypothetical protein
Anisa03747	PROKKA_03747 hypothetical protein
Anisa03770	PROKKA_03770 Soluble cytochrome b558

Anisa03771	PROKKA_03771 Adenosylhomocysteinase
Anisa03772	PROKKA_03772 hypothetical protein
Anisa03776	PROKKA_03776 Copper resistance protein A precursor
Anisa03777	PROKKA_03777 Copper resistance protein B precursor (CopB)
Anisa03779	PROKKA_03779 hypothetical protein
Anisa03780	PROKKA_03780 hypothetical protein
Anisa03781	PROKKA_03781 hypothetical protein
Anisa03784	PROKKA_03784 Nicotinate dehydrogenase subunit B
Anisa03785	PROKKA_03785 hypothetical protein
Anisa03786	PROKKA_03786 hypothetical protein
Anisa03787	PROKKA_03787 hypothetical protein
Anisa03797	PROKKA_03797 hypothetical protein
Anisa03810	PROKKA_03810 hypothetical protein
Anisa03813	PROKKA_03813 hypothetical protein
Anisa03820	PROKKA_03820 L-lactate dehydrogenase
Anisa03828	PROKKA_03828 hypothetical protein
Anisa03833	PROKKA_03833 hypothetical protein
Anisa03843	PROKKA_03843 hypothetical protein
Anisa03849	PROKKA_03849 Relaxosome protein
Anisa03851	PROKKA_03851 hypothetical protein
Anisa03857	PROKKA_03857 Transposase, TnpA family
Anisa03859	PROKKA_03859 hypothetical protein
Anisa03867	PROKKA_03867 hypothetical protein
Anisa03868	PROKKA_03868 Doubled CXXCH motif (Paired_CXXCH_1)
Anisa03869	PROKKA_03869 hypothetical protein
Anisa03883	PROKKA_03883 Retron-type reverse transcriptase
Anisa03884	PROKKA_03884 hypothetical protein
Anisa03887	PROKKA_03887 hypothetical protein
Anisa03890	PROKKA_03890 hypothetical protein
boze1_00013	PROKKA_00013 hypothetical protein

boze1_00028	PROKKA_00028 hypothetical protein
boze1_00066	PROKKA_00067 hypothetical protein
boze1_00080	PROKKA_00081 hypothetical protein
boze1_00101	PROKKA_00102 hypothetical protein
boze1_00143	PROKKA_00144 carbonic anhydrase
boze1_00388	PROKKA_00389 hypothetical protein
boze1_00424	PROKKA_00425 hypothetical protein
boze1_00482	PROKKA_00483 Lhr-like helicases
boze1_00483	PROKKA_00484 ATP-independent RNA helicase DbpA
boze1_00524	PROKKA_00525 hypothetical protein
boze1_00544	PROKKA_00545 ATP-dependent zinc metalloprotease FtsH 4
boze1_00550	PROKKA_00551 D-3-phosphoglycerate dehydrogenase
boze1_00640	PROKKA_00642 Ferrous iron transport protein B
boze1_00648	PROKKA_00650 hypothetical protein
boze1_00658	PROKKA_00660 Signal recognition particle receptor FtsY
boze1_00740	PROKKA_00743 DNA-binding transcriptional regulator CpxR
boze1_00754	PROKKA_00757 UDP-2-acetamido-2-deoxy-3-oxo-D-glucuronate aminotransferase
boze1_00777	PROKKA_00780 hypothetical protein
boze1_00824	PROKKA_00827 Putative beta-lactamase HcpD precursor
boze1_00836	PROKKA_00840 multidrug resistance outer membrane protein MdtQ
boze1_00939	PROKKA_00945 hypothetical protein
boze1_00943	PROKKA_00949 putative glycosyl transferase
boze1_00955	PROKKA_00961 hypothetical protein
boze1_00993	PROKKA_00999 Lipid A core - O-antigen ligase
boze1_01023	PROKKA_01029 Flagellar biosynthetic protein FlhB
boze1_01172	PROKKA_01180 hypothetical protein
boze1_01216	PROKKA_01224 hypothetical protein
boze1_01273	PROKKA_01281 Patatin phospholipase
boze1_01380	PROKKA_01388 hypothetical protein
boze1_01381	PROKKA_01389 hypothetical protein

boze1_01431	PROKKA_01440 Hpt domain protein
boze1_01482	PROKKA_01491 hypothetical protein
boze1_01498	PROKKA_01508 Major Facilitator Superfamily protein
boze1_01530	PROKKA_01541 hypothetical protein
boze1_01560	PROKKA_01571 hypothetical protein
boze1_01589	PROKKA_01600 peroxidase
boze1_01691	PROKKA_01702 D-alanyl-D-alanine carboxypeptidase DacC precursor
boze1_01735	PROKKA_01746 Pyruvate dehydrogenase E1 component subunit alpha
boze1_01759	PROKKA_01770 O-acetyltransferase OatA
boze1_01784	PROKKA_01795 hypothetical protein
boze1_01890	PROKKA_01901 GTP pyrophosphokinase
boze1_01893	PROKKA_01904 hypothetical protein
boze1_01901	PROKKA_01912 sn-glycerol-3-phosphate-binding periplasmic protein UgpB precursor
boze1_01939	PROKKA_01950 16S rRNA methyltransferase B
boze1_01958	PROKKA_01970 hypothetical protein
boze1_01967	PROKKA_01979 Transposase
boze1_01986	PROKKA_01999 hypothetical protein
boze1_02011	PROKKA_02024 hypothetical protein
boze1_02021	PROKKA_02037 Elongation factor 4
boze1_02088	PROKKA_02111 hypothetical protein
boze1_02093	PROKKA_02116 Serine carboxypeptidase S28
boze1_02226	PROKKA_02249 hypothetical protein
boze1_02227	PROKKA_02250 hypothetical protein
boze1_02233	PROKKA_02256 hypothetical protein
boze1_02373	PROKKA_02397 hypothetical protein
boze1_02377	PROKKA_02401 hypothetical protein
boze1_02378	PROKKA_02402 putative zinc finger/helix-turn-helix protein, YgiT family
boze1_02379	PROKKA_02403 hypothetical protein
boze1_02393	PROKKA_02417 Aspartokinase I/homoserine dehydrogenase I
boze1_02405	PROKKA_02429 GDSL-like Lipase/Acylhydrolase

boze1_02447	PROKKA_02474 hypothetical protein
boze1_02496	PROKKA_02524 Alpha-ketoglutarate permease
boze1_02504	PROKKA_02532 hypothetical protein
boze1_02508	PROKKA_02536 hypothetical protein
boze1_02511	PROKKA_02539 integrating conjugative element protein, family
boze1_02513	PROKKA_02541 conjugative transfer region lipoprotein
boze1_02515	PROKKA_02543 integrating conjugative element protein, family
boze1_02517	PROKKA_02545 conjugative transfer region protein
boze1_02525	PROKKA_02553 integrating conjugative element protein PilL, PFGI-1 class
boze1_02532	PROKKA_02560 RNA polymerase-associated protein RapA
boze1_02579	PROKKA_02607 hypothetical protein
boze1_02614	PROKKA_02642 hypothetical protein
boze1_02630	PROKKA_02658 putative hemoglobin and hemoglobin-haptoglobin-binding protein 1 precursor
boze1_02673	PROKKA_02701 hypothetical protein
boze1_02677	PROKKA_02705 DNA polymerase V subunit UmuC
boze1_02699	PROKKA_02727 hypothetical protein
boze1_02744	PROKKA_02773 hypothetical protein
boze1_02766	PROKKA_02795 hypothetical protein
boze1_02772	PROKKA_02801 Glycolipid anchored surface protein (GAS1)
boze1_02795	PROKKA_02824 conjugal transfer ATP-binding protein TraC
boze1_02808	PROKKA_02837 hypothetical protein
boze1_02822	PROKKA_02851 hypothetical protein
boze1_02823	PROKKA_02852 hypothetical protein
boze1_02829	PROKKA_02858 hypothetical protein
boze1_02830	PROKKA_02859 hypothetical protein
boze1_02832	PROKKA_02861 hypothetical protein
boze1_02856	PROKKA_02885 hypothetical protein
boze1_02890	PROKKA_02919 Response regulator
boze1_02921	PROKKA_02950 Proline iminopeptidase
boze1_02955	PROKKA_02984 hypothetical protein

boze1_02959	PROKKA_02988 hypothetical protein
boze1_02962	PROKKA_02991 efflux transporter, RND family, MFP subunit
boze1_02969	PROKKA_02998 hypothetical protein
boze1_02977	PROKKA_03006 hypothetical protein
boze1_02990	PROKKA_03019 hypothetical protein
boze1_03003	PROKKA_03032 hypothetical protein
boze1_03023	PROKKA_03052 hypothetical protein
boze1_03033	PROKKA_03062 hypothetical protein
boze1_03038	PROKKA_03067 hypothetical protein
boze1_03057	PROKKA_03086 Pertussis toxin liberation protein F
boze1_03061	PROKKA_03090 hypothetical protein
boze1_03065	PROKKA_03094 hypothetical protein
boze1_03159	PROKKA_03188 putative DMT superfamily transporter inner membrane protein
boze1_03168	PROKKA_03197 Transposase IS116/IS110/IS902 family protein
boze1_03188	PROKKA_03217 hypothetical protein
boze1_03194	PROKKA_03223 ABC transporter arginine-binding protein 1 precursor
boze1_03209	PROKKA_03238 Acyl-coenzyme A dehydrogenase
boze1_03252	PROKKA_03281 hypothetical protein
boze1_03272	PROKKA_03301 putative diguanylate cyclase YedQ
boze1_03275	PROKKA_03304 ATP-dependent DNA helicase RecQ
boze1_03379	PROKKA_03409 Elongation factor Tu
boze1_03382	PROKKA_03412 Putative prophage CPS-53 integrase
boze1_03477	PROKKA_03507 hypothetical protein
boze1_03493	PROKKA_03525 ATP-dependent DNA helicase rep
boze1_03521	PROKKA_03556 Terephthalate 1,2-dioxygenase, reductase component 1
boze1_03522	PROKKA_03557 Na(+)-translocating NADH-quinone reductase subunit F
boze1_03558	PROKKA_03593 hypothetical protein
boze1_03562	PROKKA_03597 LexA repressor
boze1_03578	PROKKA_03613 hypothetical protein
boze1_03628	PROKKA_03667 hypothetical protein

boze2_00092	bozeman_2_D4398_00092 hypothetical protein
boze2_00097	bozeman_2_D4398_00097 hypothetical protein
boze2_00115	bozeman_2_D4398_00115 D-alanyl-lipoteichoic acid biosynthesis protein DltB
boze2_00139	bozeman_2_D4398_00139 hypothetical protein
boze2_00140	bozeman_2_D4398_00140 hypothetical protein
boze2_00142	bozeman_2_D4398_00142 hypothetical protein
boze2_00159	bozeman_2_D4398_00159 hypothetical protein
boze2_00231	bozeman_2_D4398_00231 hypothetical protein
boze2_00468	bozeman_2_D4398_00470 hypothetical protein
boze2_00499	bozeman_2_D4398_00502 Inner membrane transport permease YbhS
boze2_00512	bozeman_2_D4398_00515 hypothetical protein
boze2_00855	bozeman_2_D4398_00859 Phosphocholine hydrolase Lem3
boze2_00992	bozeman_2_D4398_00998 hypothetical protein
boze2_01038	bozeman_2_D4398_01044 hypothetical protein
boze2_01098	bozeman_2_D4398_01104 hypothetical protein
boze2_01246	bozeman_2_D4398_01253 hypothetical protein
boze2_01529	bozeman_2_D4398_01542 hypothetical protein
boze2_01530	bozeman_2_D4398_01543 hypothetical protein
boze2_01551	bozeman_2_D4398_01564 hypothetical protein
boze2_01552	bozeman_2_D4398_01565 hypothetical protein
boze2_02214	bozeman_2_D4398_02237 hypothetical protein
boze2_02413	bozeman_2_D4398_02438 hypothetical protein
boze2_02459	bozeman_2_D4398_02484 hypothetical protein
boze2_02547	bozeman_2_D4398_02572 putative O-methyltransferase
boze2_02617	bozeman_2_D4398_02644 5,6-dimethylbenzimidazole synthase
boze2_02669	bozeman_2_D4398_02696 hypothetical protein
boze2_02739	bozeman_2_D4398_02767 hypothetical protein
boze2_02797	bozeman_2_D4398_02825 hypothetical protein
boze2_02859	bozeman_2_D4398_02890 hypothetical protein
boze2_02878	bozeman_2_D4398_02909 hypothetical protein

boze2_02915	bozeman_2_D4398_02946 hypothetical protein
boze2_02936	bozeman_2_D4398_02967 hypothetical protein
boze2_02960	bozeman_2_D4398_02991 Phenylalanine-4-hydroxylase
boze2_02997	bozeman_2_D4398_03029 hypothetical protein
boze2_03085	bozeman_2_D4398_03123 hypothetical protein
boze2_03087	bozeman_2_D4398_03125 hypothetical protein
boze2_03088	bozeman_2_D4398_03126 hypothetical protein
boze2_03089	bozeman_2_D4398_03127 hypothetical protein
boze2_03155	bozeman_2_D4398_03193 hypothetical protein
boze2_03160	bozeman_2_D4398_03198 hypothetical protein
boze2_03169	bozeman_2_D4398_03207 hypothetical protein
boze2_03177	bozeman_2_D4398_03215 hypothetical protein
boze2_03180	bozeman_2_D4398_03218 hypothetical protein
boze2_03259	bozeman_2_D4398_03297 hypothetical protein
boze2_03260	bozeman_2_D4398_03298 hypothetical protein
boze2_03282	bozeman_2_D4398_03320 hypothetical protein
boze2_03301	bozeman_2_D4398_03339 hypothetical protein
boze2_03410	bozeman_2_D4398_03451 hypothetical protein
boze2_03432	bozeman_2_D4398_03473 hypothetical protein
boze2_03448	bozeman_2_D4398_03489 hypothetical protein
cherii00007	PROKKA_00007 hypothetical protein
cherii00019	PROKKA_00019 Polyketide synthase PksJ
cherii00031	PROKKA_00031 hypothetical protein
cherii00035	PROKKA_00036 hypothetical protein
cherii00044	PROKKA_00045 hypothetical protein
cherii00073	PROKKA_00074 Alpha/beta hydrolase family protein
cherii00084	PROKKA_00085 Frizzy aggregation protein FrzCD
cherii00085	PROKKA_00086 hypothetical protein
cherii00126	PROKKA_00129 hypothetical protein
cherii00128	PROKKA_00131 gamma-butyrobetaine hydroxylase

cherii00131	PROKKA_00134 YHS domain protein
cherii00132	PROKKA_00135 hypothetical protein
cherii00137	PROKKA_00140 hypothetical protein
cherii00157	PROKKA_00160 ankyrin repeat protein
cherii00158	PROKKA_00161 hypothetical protein
cherii00166	PROKKA_00169 hypothetical protein
cherii00174	PROKKA_00177 anthranilate synthase component II
cherii00180	PROKKA_00183 SH2 domain protein
cherii00196	PROKKA_00199 hypothetical protein
cherii00199	PROKKA_00202 hypothetical protein
cherii00208	PROKKA_00211 Agmatinase
cherii00221	PROKKA_00224 hypothetical protein
cherii00267	PROKKA_00272 Polyketide synthase PksM
cherii00268	PROKKA_00273 Beta-ketoacyl-acyl-carrier-protein synthase I
cherii00269	PROKKA_00274 hypothetical protein
cherii00272	PROKKA_00277 hypothetical protein
cherii00282	PROKKA_00287 hypothetical protein
cherii00288	PROKKA_00293 hypothetical protein
cherii00289	PROKKA_00294 hypothetical protein
cherii00290	PROKKA_00295 hypothetical protein
cherii00300	PROKKA_00306 hypothetical protein
cherii00302	PROKKA_00308 Ankyrin repeats (3 copies)
cherii00363	PROKKA_00369 hypothetical protein
cherii00365	PROKKA_00371 hypothetical protein
cherii00420	PROKKA_00426 hypothetical protein
cherii00447	PROKKA_00453 hypothetical protein
cherii00463	PROKKA_00469 hypothetical protein
cherii00466	PROKKA_00472 ankyrin repeat protein
cherii00467	PROKKA_00473 hypothetical protein
cherii00480	PROKKA_00486 hypothetical protein

cherii00584	PROKKA_00590 Chloramphenicol resistance pump Cmr
cherii00587	PROKKA_00593 hypothetical protein
cherii00591	PROKKA_00597 hypothetical protein
cherii00644	PROKKA_00650 hypothetical protein
cherii00657	PROKKA_00664 hypothetical protein
cherii00699	PROKKA_00709 Capsule polysaccharide biosynthesis protein
cherii00701	PROKKA_00711 hypothetical protein
cherii00711	PROKKA_00721 hypothetical protein
cherii00724	PROKKA_00735 ATP-dependent helicase/nuclease subunit A
cherii00725	PROKKA_00736 Inactivated superfamily I helicase
cherii00726	PROKKA_00737 hypothetical protein
cherii00727	PROKKA_00738 hypothetical protein
cherii00730	PROKKA_00741 hypothetical protein
cherii00731	PROKKA_00742 hypothetical protein
cherii00732	PROKKA_00743 hypothetical protein
cherii00733	PROKKA_00744 hypothetical protein
cherii00735	PROKKA_00746 hypothetical protein
cherii00765	PROKKA_00776 hypothetical protein
cherii00774	PROKKA_00785 hypothetical protein
cherii00775	PROKKA_00786 hypothetical protein
cherii00779	PROKKA_00790 hypothetical protein
cherii00780	PROKKA_00791 Bacteriophytochrome cph2
cherii00783	PROKKA_00794 Ras family protein
cherii00789	PROKKA_00800 hypothetical protein
cherii00790	PROKKA_00801 hypothetical protein
cherii00805	PROKKA_00816 chaperone protein DnaJ
cherii00824	PROKKA_00835 hypothetical protein
cherii00825	PROKKA_00836 hypothetical protein
cherii00892	PROKKA_00903 hypothetical protein
cherii00893	PROKKA_00904 hypothetical protein

cherii00963	PROKKA_00975 Transcriptional repressor SdpR
cherii00964	PROKKA_00976 hypothetical protein
cherii00983	PROKKA_00995 hypothetical protein
cherii00984	PROKKA_00996 hypothetical protein
cherii00992	PROKKA_01004 Bacteriophytochrome cph2
cherii01009	PROKKA_01021 hypothetical protein
cherii01022	PROKKA_01034 hypothetical protein
cherii01061	PROKKA_01073 hypothetical protein
cherii01062	PROKKA_01074 Polysaccharide biosynthesis protein
cherii01076	PROKKA_01088 Asparagine synthetase [glutamine-hydrolyzing] 1
cherii01077	PROKKA_01089 hypothetical protein
cherii01078	PROKKA_01090 ubiquinone/menaquinone biosynthesis methyltransferase
cherii01079	PROKKA_01091 Putative glycosyltransferase CsbB
cherii01080	PROKKA_01092 4-amino-4-deoxy-L-arabinose-phosphoundecaprenol flippase subunit ArnF
cherii01081	PROKKA_01093 biotin biosynthesis protein BioC
cherii01082	PROKKA_01094 hypothetical protein
cherii01083	PROKKA_01095 tRNA mo(5)U34 methyltransferase
cherii01113	PROKKA_01125 hypothetical protein
cherii01128	PROKKA_01140 hypothetical protein
cherii01161	PROKKA_01173 hypothetical protein
cherii01164	PROKKA_01176 NADP-dependent malic enzyme
cherii01174	PROKKA_01186 hypothetical protein
cherii01198	PROKKA_01210 cyanophycinase
cherii01248	PROKKA_01260 Stage 0 sporulation protein KE
cherii01302	PROKKA_01314 hypothetical protein
cherii01313	PROKKA_01325 hypothetical protein
cherii01362	PROKKA_01374 hypothetical protein
cherii01363	PROKKA_01375 hypothetical protein
cherii01431	PROKKA_01445 hypothetical protein
cherii01435	PROKKA_01449 hypothetical protein

cherii01436	PROKKA_01450 hypothetical protein
cherii01459	PROKKA_01473 hypothetical protein
cherii01467	PROKKA_01481 hypothetical protein
cherii01493	PROKKA_01507 Poly-beta-hydroxybutyrate polymerase
cherii01503	PROKKA_01517 hypothetical protein
cherii01515	PROKKA_01529 DNA helicase II
cherii01560	PROKKA_01574 hypothetical protein
cherii01561	PROKKA_01575 hypothetical protein
cherii01633	PROKKA_01649 hypothetical protein
cherii01636	PROKKA_01652 Cytochrome P450
cherii01637	PROKKA_01653 hypothetical protein
cherii01640	PROKKA_01656 hypothetical protein
cherii01644	PROKKA_01660 hypothetical protein
cherii01667	PROKKA_01683 hypothetical protein
cherii01668	PROKKA_01684 hypothetical protein
cherii01709	PROKKA_01725 hypothetical protein
cherii01781	PROKKA_01798 F0F1 ATP synthase subunit B
cherii01788	PROKKA_01805 hypothetical protein
cherii01799	PROKKA_01816 hypothetical protein
cherii01826	PROKKA_01843 hypothetical protein
cherii01864	PROKKA_01881 hypothetical protein
cherii01874	PROKKA_01891 hypothetical protein
cherii01886	PROKKA_01903 Phosphoenolpyruvate synthase
cherii01915	PROKKA_01933 hypothetical protein
cherii01933	PROKKA_01954 Cold shock-like protein CspE
cherii01966	PROKKA_01987 hypothetical protein
cherii01988	PROKKA_02009 hypothetical protein
cherii02007	PROKKA_02028 type VI secretion protein, family
cherii02023	PROKKA_02044 hypothetical protein
cherii02067	PROKKA_02089 Rhodocoxin reductase

cherii02116	PROKKA_02148 hypothetical protein
cherii02127	PROKKA_02159 Multidrug transporter MdtC
cherii02223	PROKKA_02255 hypothetical protein
cherii02224	PROKKA_02256 hypothetical protein
cherii02225	PROKKA_02257 hypothetical protein
cherii02226	PROKKA_02258 hypothetical protein
cherii02232	PROKKA_02264 putative membrane protein
cherii02233	PROKKA_02265 putative membrane protein
cherii02236	PROKKA_02268 hypothetical protein
cherii02241	PROKKA_02273 hypothetical protein
cherii02246	PROKKA_02278 CheW-like domain protein
cherii02247	PROKKA_02279 Chemotaxis protein CheC, inhibitor of MCP methylation
cherii02257	PROKKA_02289 hypothetical protein
cherii02269	PROKKA_02301 hypothetical protein
cherii02284	PROKKA_02316 hypothetical protein
cherii02285	PROKKA_02317 hypothetical protein
cherii02290	PROKKA_02322 hypothetical protein
cherii02295	PROKKA_02327 GH3 auxin-responsive promoter
cherii02310	PROKKA_02342 hypothetical protein
cherii02330	PROKKA_02362 hypothetical protein
cherii02334	PROKKA_02366 hypothetical protein
cherii02355	PROKKA_02387 hypothetical protein
cherii02374	PROKKA_02406 putative lysine decarboxylase
cherii02385	PROKKA_02417 hypothetical protein
cherii02417	PROKKA_02449 hypothetical protein
cherii02435	PROKKA_02467 hypothetical protein
cherii02439	PROKKA_02471 hypothetical protein
cherii02442	PROKKA_02474 hypothetical protein
cherii02482	PROKKA_02514 hypothetical protein
cherii02498	PROKKA_02531 hypothetical protein

cherii02508	PROKKA_02542 hypothetical protein
cherii02549	PROKKA_02583 4-cresol dehydrogenase [hydroxylating] flavoprotein subunit
cherii02559	PROKKA_02593 hypothetical protein
cherii02568	PROKKA_02602 putative dienelactone hydrolase
cherii02572	PROKKA_02606 hypothetical protein
cherii02573	PROKKA_02607 hypothetical protein
cherii02579	PROKKA_02613 2-hydroxy-3-keto-5-methylthiopentenyl-1-phosphate phosphatase
cherii02580	PROKKA_02614 Acetylornithine aminotransferase
cherii02581	PROKKA_02615 hypothetical protein
cherii02582	PROKKA_02616 L-glutamine:2-deoxy-scyllo-inosose aminotransferase
cherii02583	PROKKA_02617 Arginase family protein
cherii02584	PROKKA_02618 4-amino-4-deoxy-L-arabinose-phosphoundecaprenol flippase subunit ArnE
cherii02585	PROKKA_02619 EamA-like transporter family protein
cherii02590	PROKKA_02624 hypothetical protein
cherii02591	PROKKA_02625 Enoyl-[acyl-carrier-protein] reductase [NADPH] FabL
cherii02592	PROKKA_02626 hypothetical protein
cherii02595	PROKKA_02629 Cyclopentanol dehydrogenase
cherii02596	PROKKA_02630 D-beta-hydroxybutyrate dehydrogenase
cherii02604	PROKKA_02638 hypothetical protein
cherii02606	PROKKA_02640 Bacteriophytochrome cph2
cherii02613	PROKKA_02647 hypothetical protein
cherii02617	PROKKA_02651 hypothetical protein
cherii02641	PROKKA_02676 hypothetical protein
cherii02643	PROKKA_02678 hypothetical protein
cherii02644	PROKKA_02679 hypothetical protein
cherii02645	PROKKA_02680 hypothetical protein
cherii02654	PROKKA_02689 hypothetical protein
cherii02656	PROKKA_02691 Cholesterol dehydrogenase
cherii02663	PROKKA_02698 Bleomycin-binding protein
cherii02664	PROKKA_02699 hypothetical protein

cherii02703	PROKKA_02738 polysaccharide deacetylase family sporulation protein PdaB
cherii02704	PROKKA_02739 hypothetical protein
cherii02722	PROKKA_02757 hypothetical protein
cherii02740	PROKKA_02775 hypothetical protein
cherii02742	PROKKA_02777 hypothetical protein
cherii02762	PROKKA_02797 hypothetical protein
cherii02766	PROKKA_02801 hypothetical protein
cherii02772	PROKKA_02807 diguanylate cyclase (GGDEF) domain protein
cherii02786	PROKKA_02821 hypothetical protein
cherii02839	PROKKA_02874 hypothetical protein
cherii02854	PROKKA_02889 hypothetical protein
cherii02876	PROKKA_02911 hypothetical protein
cherii02890	PROKKA_02925 hypothetical protein
cherii02910	PROKKA_02945 hypothetical protein
cherii02914	PROKKA_02949 hypothetical protein
cherii02954	PROKKA_02989 hypothetical protein
cherii02957	PROKKA_02992 hypothetical protein
cherii02978	PROKKA_03013 Alkaline phosphatase precursor
cherii03026	PROKKA_03061 NAD-specific glutamate dehydrogenase
cherii03100	PROKKA_03135 hypothetical protein
cherii03102	PROKKA_03137 ADP-ribosyltransferase exoenzyme
cherii03209	PROKKA_03246 signal peptide peptidase SppA, 67K type
cherii03219	PROKKA_03256 RES domain protein
cherii03220	PROKKA_03257 hypothetical protein
cherii03221	PROKKA_03258 hypothetical protein
cherii03222	PROKKA_03259 hypothetical protein
Cincinnati00012	PROKKA_00012 hypothetical protein
Cincinnati00014	PROKKA_00014 hypothetical protein
Cincinnati00015	PROKKA_00015 hypothetical protein
Cincinnati00021	PROKKA_00021 hypothetical protein

Cincinnati00023	PROKKA_00023 hypothetical protein
Cincinnati00025	PROKKA_00025 hypothetical protein
Cincinnati00026	PROKKA_00026 DNA polymerase III subunit epsilon
Cincinnati00067	PROKKA_00067 Extracellular basic protease precursor
Cincinnati00085	PROKKA_00085 hypothetical protein
Cincinnati00127	PROKKA_00127 hypothetical protein
Cincinnati00145	PROKKA_00145 putative membrane protein
Cincinnati00146	PROKKA_00146 hypothetical protein
Cincinnati00147	PROKKA_00147 biotin biosynthesis protein BioC
Cincinnati00148	PROKKA_00148 hypothetical protein
Cincinnati00150	PROKKA_00150 hypothetical protein
Cincinnati00213	PROKKA_00213 hypothetical protein
Cincinnati00214	PROKKA_00214 hypothetical protein
Cincinnati00215	PROKKA_00215 hypothetical protein
Cincinnati00222	PROKKA_00222 Glucose-6-phosphate isomerase
Cincinnati00236	PROKKA_00236 3-phenylpropionate dioxygenase ferredoxin subunit
Cincinnati00238	PROKKA_00238 hypothetical protein
Cincinnati00240	PROKKA_00240 Mitochondrial carrier protein
Cincinnati00268	PROKKA_00268 hypothetical protein
Cincinnati00272	PROKKA_00272 hypothetical protein
Cincinnati00321	PROKKA_00321 Electron transfer flavoprotein-ubiquinone oxidoreductase
Cincinnati00339	PROKKA_00339 hypothetical protein
Cincinnati00359	PROKKA_00359 putative HTH-type transcriptional regulator YfiR
Cincinnati00365	PROKKA_00365 hypothetical protein
Cincinnati00379	PROKKA_00379 hypothetical protein
Cincinnati00386	PROKKA_00386 GTP-binding protein EngA
Cincinnati00401	PROKKA_00401 hypothetical protein
Cincinnati00491	PROKKA_00492 hypothetical protein
Cincinnati00492	PROKKA_00493 hypothetical protein
Cincinnati00500	PROKKA_00501 hypothetical protein

Cincinnati00503	PROKKA_00504 hypothetical protein
Cincinnati00524	PROKKA_00528 Ribulose-5-phosphate 4-epimerase and aldolases
Cincinnati00543	PROKKA_00547 hypothetical protein
Cincinnati00561	PROKKA_00566 N-carbamoylsarcosine amidase
Cincinnati00593	PROKKA_00598 hypothetical protein
Cincinnati00604	PROKKA_00609 hypothetical protein
Cincinnati00640	PROKKA_00646 Dot/Icm substrate protein
Cincinnati00666	PROKKA_00672 hypothetical protein
Cincinnati00713	PROKKA_00719 hypothetical protein
Cincinnati00770	PROKKA_00776 hypothetical protein
Cincinnati00780	PROKKA_00786 hypothetical protein
Cincinnati00822	PROKKA_00828 PAP2 superfamily protein
Cincinnati00865	PROKKA_00874 hypothetical protein
Cincinnati00868	PROKKA_00877 hypothetical protein
Cincinnati00870	PROKKA_00879 hypothetical protein
Cincinnati00873	PROKKA_00882 Uridine kinase
Cincinnati00884	PROKKA_00893 hypothetical protein
Cincinnati00885	PROKKA_00894 hypothetical protein
Cincinnati00920	PROKKA_00929 hypothetical protein
Cincinnati00980	PROKKA_00990 primosomal protein DnaI
Cincinnati00991	PROKKA_01001 hypothetical protein
Cincinnati00999	PROKKA_01009 hypothetical protein
Cincinnati01001	PROKKA_01012 hypothetical protein
Cincinnati01024	PROKKA_01035 hypothetical protein
Cincinnati01039	PROKKA_01050 Ribonuclease R
Cincinnati01052	PROKKA_01063 hypothetical protein
Cincinnati01053	PROKKA_01064 hypothetical protein
Cincinnati01054	PROKKA_01065 Transposase
Cincinnati01061	PROKKA_01072 hypothetical protein
Cincinnati01095	PROKKA_01106 hypothetical protein

Cincinnati01126	PROKKA_01138 Ankyrin repeats (3 copies)
Cincinnati01127	PROKKA_01139 hypothetical protein
Cincinnati01128	PROKKA_01140 hypothetical protein
Cincinnati01134	PROKKA_01146 Superfamily II helicase
Cincinnati01176	PROKKA_01189 hypothetical protein
Cincinnati01214	PROKKA_01227 hypothetical protein
Cincinnati01290	PROKKA_01304 hypothetical protein
Cincinnati01295	PROKKA_01309 hypothetical protein
Cincinnati01313	PROKKA_01327 hypothetical protein
Cincinnati01314	PROKKA_01328 hypothetical protein
Cincinnati01315	PROKKA_01329 hypothetical protein
Cincinnati01389	PROKKA_01403 hypothetical protein
Cincinnati01427	PROKKA_01441 hypothetical protein
Cincinnati01462	PROKKA_01478 hypothetical protein
Cincinnati01463	PROKKA_01479 hypothetical protein
Cincinnati01472	PROKKA_01488 hypothetical protein
Cincinnati01500	PROKKA_01516 hypothetical protein
Cincinnati01516	PROKKA_01532 hypothetical protein
Cincinnati01519	PROKKA_01535 hypothetical protein
Cincinnati01522	PROKKA_01538 hypothetical protein
Cincinnati01535	PROKKA_01551 copper/silver efflux system outer membrane protein CusC
Cincinnati01549	PROKKA_01565 exodeoxyribonuclease III
Cincinnati01567	PROKKA_01583 Tyrocidine synthase III
Cincinnati01572	PROKKA_01588 hypothetical protein
Cincinnati01631	PROKKA_01647 hypothetical protein
Cincinnati01632	PROKKA_01648 hypothetical protein
Cincinnati01643	PROKKA_01659 hypothetical protein
Cincinnati01701	PROKKA_01717 Efflux pump membrane transporter BepE
Cincinnati01703	PROKKA_01719 hypothetical protein
Cincinnati01714	PROKKA_01730 hypothetical protein

Cincinnati01735	PROKKA_01752 hypothetical protein
Cincinnati01738	PROKKA_01755 hypothetical protein
Cincinnati01744	PROKKA_01761 hypothetical protein
Cincinnati01814	PROKKA_01831 hypothetical protein
Cincinnati01821	PROKKA_01838 Protease HtpX
Cincinnati01856	PROKKA_01873 Putative prophage CPS-53 integrase
Cincinnati01877	PROKKA_01895 hypothetical protein
Cincinnati01899	PROKKA_01917 type-F conjugative transfer system protein TraW
Cincinnati01912	PROKKA_01930 hypothetical protein
Cincinnati01913	PROKKA_01931 hypothetical protein
Cincinnati01931	PROKKA_01949 hypothetical protein
Cincinnati01932	PROKKA_01950 hypothetical protein
Cincinnati01937	PROKKA_01955 hypothetical protein
Cincinnati01938	PROKKA_01956 hypothetical protein
Cincinnati01939	PROKKA_01957 Inosamine-phosphate amidinotransferase 1
Cincinnati01940	PROKKA_01958 L-ornithine 5-monooxygenase
Cincinnati01941	PROKKA_01959 ABC transporter ATP-binding protein YojI
Cincinnati01942	PROKKA_01960 N(6)-hydroxylysine O-acetyltransferase
Cincinnati01947	PROKKA_01965 Ferric iron reductase protein FhuF
Cincinnati01951	PROKKA_01969 MbtH-like protein
Cincinnati01952	PROKKA_01970 putative RNA polymerase sigma factor FecI
Cincinnati01953	PROKKA_01971 hypothetical protein
Cincinnati01954	PROKKA_01972 hypothetical protein
Cincinnati01960	PROKKA_01978 hypothetical protein
Cincinnati01961	PROKKA_01979 hypothetical protein
Cincinnati01962	PROKKA_01980 hypothetical protein
Cincinnati01968	PROKKA_01986 multifunctional aminopeptidase A
Cincinnati01977	PROKKA_01995 hypothetical protein
Cincinnati01997	PROKKA_02016 hypothetical protein
Cincinnati02006	PROKKA_02025 hypothetical protein

Cincinnati02011	PROKKA_02030 hypothetical protein
Cincinnati02028	PROKKA_02047 inositol phosphate phosphatase SopB
Cincinnati02032	PROKKA_02051 hypothetical protein
Cincinnati02034	PROKKA_02053 hypothetical protein
Cincinnati02039	PROKKA_02058 hypothetical protein
Cincinnati02070	PROKKA_02089 hypothetical protein
Cincinnati02114	PROKKA_02133 Nitroreductase family protein
Cincinnati02140	PROKKA_02159 hypothetical protein
Cincinnati02174	PROKKA_02193 hypothetical protein
Cincinnati02206	PROKKA_02225 hypothetical protein
Cincinnati02207	PROKKA_02226 hypothetical protein
Cincinnati02212	PROKKA_02231 hypothetical protein
Cincinnati02219	PROKKA_02241 hypothetical protein
Cincinnati02228	PROKKA_02250 hypothetical protein
Cincinnati02231	PROKKA_02253 Putative protein-S-isoprenylcysteine methyltransferase
Cincinnati02232	PROKKA_02254 Condensation domain protein
Cincinnati02233	PROKKA_02255 hypothetical protein
Cincinnati02260	PROKKA_02282 hypothetical protein
Cincinnati02316	PROKKA_02338 hypothetical protein
Cincinnati02328	PROKKA_02350 hypothetical protein
Cincinnati02346	PROKKA_02368 hypothetical protein
Cincinnati02375	PROKKA_02397 haloacid dehalogenase-like hydrolase
Cincinnati02380	PROKKA_02402 poly(R)-hydroxyalkanoic acid synthase, class I
Cincinnati02382	PROKKA_02404 hypothetical protein
Cincinnati02384	PROKKA_02406 Enolase
Cincinnati02393	PROKKA_02415 Cold shock-like protein CspG
Cincinnati02394	PROKKA_02416 Cold-shock DEAD box protein A
Cincinnati02399	PROKKA_02421 hypothetical protein
Cincinnati02400	PROKKA_02422 hypothetical protein
Cincinnati02401	PROKKA_02423 hypothetical protein

Cincinnati02402	PROKKA_02424 hypothetical protein
Cincinnati02403	PROKKA_02425 hypothetical protein
Cincinnati02404	PROKKA_02426 hypothetical protein
Cincinnati02405	PROKKA_02427 hypothetical protein
Cincinnati02406	PROKKA_02428 hypothetical protein
Cincinnati02407	PROKKA_02429 hypothetical protein
Cincinnati02411	PROKKA_02433 hypothetical protein
Cincinnati02414	PROKKA_02436 hypothetical protein
Cincinnati02415	PROKKA_02437 hypothetical protein
Cincinnati02422	PROKKA_02444 hypothetical protein
Cincinnati02423	PROKKA_02445 hypothetical protein
Cincinnati02430	PROKKA_02452 hypothetical protein
Cincinnati02433	PROKKA_02455 hypothetical protein
Cincinnati02459	PROKKA_02481 Tyrosine phenol-lyase
Cincinnati02460	PROKKA_02482 hypothetical protein
Cincinnati02480	PROKKA_02503 putative peptidoglycan biosynthesis protein MurJ
Cincinnati02481	PROKKA_02504 hypothetical protein
Cincinnati02492	PROKKA_02515 hypothetical protein
Cincinnati02493	PROKKA_02516 hypothetical protein
Cincinnati02519	PROKKA_02544 hypothetical protein
Cincinnati02535	PROKKA_02560 N-acyl homoserine lactonase AttM
Cincinnati02536	PROKKA_02561 Maleamate amidohydrolase
Cincinnati02559	PROKKA_02584 hypothetical protein
Cincinnati02576	PROKKA_02601 hypothetical protein
Cincinnati02586	PROKKA_02611 hypothetical protein
Cincinnati02613	PROKKA_02639 hypothetical protein
Cincinnati02653	PROKKA_02679 putative dipeptide and tripeptide permease YjdL
Cincinnati02686	PROKKA_02712 ribose-phosphate pyrophosphokinase
Cincinnati02695	PROKKA_02721 hypothetical protein
Cincinnati02720	PROKKA_02746 hypothetical protein

Cincinnati02797	PROKKA_02824 2-deoxyglucose-6-phosphatase
Cincinnati02809	PROKKA_02836 hypothetical protein
Cincinnati02816	PROKKA_02843 hypothetical protein
Cincinnati02817	PROKKA_02844 hypothetical protein
Cincinnati02827	PROKKA_02854 hypothetical protein
Cincinnati02828	PROKKA_02855 putative monovalent cation/H+ antiporter subunit F
Cincinnati02829	PROKKA_02856 putative monovalent cation/H+ antiporter subunit G
Cincinnati02830	PROKKA_02857 putative monovalent cation/H+ antiporter subunit B
Cincinnati02831	PROKKA_02858 putative monovalent cation/H+ antiporter subunit B
Cincinnati02835	PROKKA_02862 Ferredoxin-dependent glutamate synthase 1
Cincinnati02842	PROKKA_02869 hypothetical protein
Cincinnati02843	PROKKA_02870 hypothetical protein
Cincinnati02844	PROKKA_02871 Glyoxal reductase
Cincinnati02846	PROKKA_02873 hypothetical protein
Cincinnati02847	PROKKA_02874 hypothetical protein
Cincinnati02848	PROKKA_02875 hypothetical protein
Cincinnati02849	PROKKA_02876 Transposase, IS30 family
Cincinnati02850	PROKKA_02877 hypothetical protein
Cincinnati02889	PROKKA_02917 hypothetical protein
Cincinnati02898	PROKKA_02926 hypothetical protein
Cincinnati02899	PROKKA_02927 hypothetical protein
Cincinnati02918	PROKKA_02946 hypothetical protein
Cincinnati02928	PROKKA_02956 hypothetical protein
Cincinnati02940	PROKKA_02968 hypothetical protein
Cincinnati02943	PROKKA_02971 hypothetical protein
Cincinnati02947	PROKKA_02975 hypothetical protein
Cincinnati02948	PROKKA_02976 Sensor protein CpxA
Cincinnati02953	PROKKA_02981 hypothetical protein
Cincinnati02955	PROKKA_02983 hypothetical protein
Cincinnati02959	PROKKA_02987 hypothetical protein

Cincinnati02965	PROKKA_02993 hypothetical protein
Cincinnati02973	PROKKA_03001 hypothetical protein
Cincinnati02988	PROKKA_03016 hypothetical protein
Cincinnati03021	PROKKA_03056 Putative DNA ligase-like protein/MT0965
Cincinnati03025	PROKKA_03060 hypothetical protein
Cincinnati03057	PROKKA_03092 multidrug efflux system subunit MdtC
Cincinnati03058	PROKKA_03093 Multidrug resistance protein MdtE precursor
Cincinnati03059	PROKKA_03094 efflux transporter, RND family, MFP subunit
Cincinnati03081	PROKKA_03116 Putative transposase
Cincinnati03083	PROKKA_03118 3-oxoacyl-[acyl-carrier-protein] synthase 2
Cincinnati03101	PROKKA_03136 hypothetical protein
Cincinnati03102	PROKKA_03137 hypothetical protein
Cincinnati03105	PROKKA_03140 hypothetical protein
Cincinnati03114	PROKKA_03149 hypothetical protein
Cincinnati03117	PROKKA_03152 hypothetical protein
Cincinnati03118	PROKKA_03153 Superfamily II helicase
Cincinnati03119	PROKKA_03154 Superfamily II helicase
Cincinnati03121	PROKKA_03156 Glutamyl-tRNA(Gln) amidotransferase subunit A
Cincinnati03122	PROKKA_03157 hypothetical protein
Cincinnati03134	PROKKA_03169 Decaprenyl-phosphate phosphoribosyltransferase
Cincinnati03135	PROKKA_03170 putative decaprenylphosphoryl-beta-D-ribose oxidase
Cincinnati03136	PROKKA_03171 putative oxidoreductase
Cincinnati03137	PROKKA_03172 hypothetical protein
Cincinnati03151	PROKKA_03186 hypothetical protein
Cincinnati03169	PROKKA_03204 Legionella pneumophila major outer membrane protein precursor
Cincinnati03181	PROKKA_03217 hypothetical protein
Cincinnati03186	PROKKA_03222 phosphoglycolate phosphatase
Cincinnati03187	PROKKA_03223 hypothetical protein
Cincinnati03192	PROKKA_03228 hypothetical protein
Cincinnati03207	PROKKA_03243 hypothetical protein

Cincinnati03211	PROKKA_03247 hypothetical protein
Cincinnati03213	PROKKA_03249 hypothetical protein
Cincinnati03214	PROKKA_03250 hypothetical protein
Cincinnati03217	PROKKA_03253 hypothetical protein
Cincinnati03218	PROKKA_03254 Chitinase
Cincinnati03224	PROKKA_03260 hypothetical protein
Cincinnati03228	PROKKA_03264 putative transcriptional regulator
Cincinnati03231	PROKKA_03267 RNase III inhibitor
Cincinnati03232	PROKKA_03268 hypothetical protein
Cincinnati03243	PROKKA_03279 hypothetical protein
Cincinnati03249	PROKKA_03285 hypothetical protein
Cincinnati03266	PROKKA_03302 Transposase
Cincinnati03267	PROKKA_03303 hypothetical protein
Cincinnati03269	PROKKA_03305 hypothetical protein
Cincinnati03270	PROKKA_03306 hypothetical protein
Cincinnati03271	PROKKA_03307 hypothetical protein
Cincinnati03272	PROKKA_03308 hypothetical protein
Cincinnati03273	PROKKA_03309 hypothetical protein
Cincinnati03274	PROKKA_03310 Abortive infection bacteriophage resistance protein
Cincinnati03276	PROKKA_03312 Putative multidrug export ATP-binding/permease protein
Cincinnati03307	PROKKA_03343 Transposase
Cincinnati03308	PROKKA_03344 hypothetical protein
Cincinnati03323	PROKKA_03359 hypothetical protein
Cincinnati03337	PROKKA_03373 hypothetical protein
Cincinnati03346	PROKKA_03382 cation diffusion facilitator family transporter
Cincinnati03360	PROKKA_03396 hypothetical protein
Cincinnati03374	PROKKA_03410 hypothetical protein
Cincinnati03375	PROKKA_03411 hypothetical protein
Cincinnati03381	PROKKA_03417 hypothetical protein
Cincinnati03393	PROKKA_03429 hypothetical protein

Cincinnati03423	PROKKA_03459 hypothetical protein
Cincinnati03425	PROKKA_03461 hypothetical protein
Cincinnati03438	PROKKA_03474 hypothetical protein
Cincinnati03455	PROKKA_03491 hypothetical protein
Cincinnati03464	PROKKA_03500 diadenosine tetraphosphatase
Cincinnati03465	PROKKA_03501 hypothetical protein
Cincinnati03472	PROKKA_03508 hypothetical protein
Cincinnati03478	PROKKA_03514 hypothetical protein
Cincinnati03479	PROKKA_03515 hypothetical protein
Cincinnati03482	PROKKA_03518 hypothetical protein
Cincinnati03486	PROKKA_03522 Tyrocidine synthase III
Cincinnati03491	PROKKA_03527 hypothetical protein
Cincinnati03495	PROKKA_03533 hypothetical protein
Cincinnati03498	PROKKA_03536 hypothetical protein
Cincinnati03499	PROKKA_03537 hypothetical protein
dumof00011	PROKKA_00011 hypothetical protein
dumof00012	PROKKA_00012 hypothetical protein
dumof00013	PROKKA_00013 hypothetical protein
dumof00124	PROKKA_00126 Serine 3-dehydrogenase
dumof00134	PROKKA_00136 hypothetical protein
dumof00168	PROKKA_00170 putative acetyltransferase
dumof00207	PROKKA_00209 hypothetical protein
dumof00265	PROKKA_00267 hypothetical protein
dumof00276	PROKKA_00278 Cytidylate kinase
dumof00285	PROKKA_00287 Adenylate kinase
dumof00291	PROKKA_00293 hypothetical protein
dumof00355	PROKKA_00358 Regulator of competence-specific genes
dumof00357	PROKKA_00360 KDP operon transcriptional regulatory protein KdpE
dumof00358	PROKKA_00361 Sensor protein KdpD
dumof00361	PROKKA_00364 hypothetical protein

dumof00427	PROKKA_00430 hypothetical protein
dumof00504	PROKKA_00518 Hsp20/alpha crystallin family protein
dumof00515	PROKKA_00529 hypothetical protein
dumof00516	PROKKA_00530 hypothetical protein
dumof00542	PROKKA_00556 phosphate:H+ symporter
dumof00562	PROKKA_00576 hypothetical protein
dumof00608	PROKKA_00622 hypothetical protein
dumof00610	PROKKA_00624 hypothetical protein
dumof00611	PROKKA_00625 hypothetical protein
dumof00669	PROKKA_00683 hypothetical protein
dumof00761	PROKKA_00775 Penicillin-binding protein 1A
dumof00789	PROKKA_00803 hypothetical protein
dumof00901	PROKKA_00916 Copper resistance protein A precursor
dumof00936	PROKKA_00951 hypothetical protein
dumof00948	PROKKA_00963 hypothetical protein
dumof00975	PROKKA_00992 ankyrin repeat protein
dumof01037	PROKKA_01055 hypothetical protein
dumof01039	PROKKA_01057 hypothetical protein
dumof01047	PROKKA_01065 hypothetical protein
dumof01053	PROKKA_01071 hypothetical protein
dumof01102	PROKKA_01121 hypothetical protein
dumof01124	PROKKA_01143 hypothetical protein
dumof01137	PROKKA_01156 hypothetical protein
dumof01240	PROKKA_01259 hypothetical protein
dumof01263	PROKKA_01282 hypothetical protein
dumof01271	PROKKA_01290 hypothetical protein
dumof01272	PROKKA_01291 Polysaccharide biosynthesis protein
dumof01273	PROKKA_01292 hypothetical protein
dumof01274	PROKKA_01293 hypothetical protein
dumof01275	PROKKA_01294 hypothetical protein

dumof01276	PROKKA_01295 Lipid A core - O-antigen ligase
dumof01280	PROKKA_01299 hypothetical protein
dumof01301	PROKKA_01320 hypothetical protein
dumof01312	PROKKA_01331 PrkA family serine protein kinase
dumof01366	PROKKA_01387 hypothetical protein
dumof01397	PROKKA_01418 hypothetical protein
dumof01418	PROKKA_01439 hypothetical protein
dumof01532	PROKKA_01554 hypothetical protein
dumof01681	PROKKA_01703 hypothetical protein
dumof01685	PROKKA_01707 hypothetical protein
dumof01692	PROKKA_01714 Formamidopyrimidine-DNA glycosylase
dumof01693	PROKKA_01715 hypothetical protein
dumof01696	PROKKA_01718 hypothetical protein
dumof01697	PROKKA_01719 hypothetical protein
dumof01728	PROKKA_01750 hypothetical protein
dumof01742	PROKKA_01764 hypothetical protein
dumof01743	PROKKA_01765 hypothetical protein
dumof01809	PROKKA_01831 hypothetical protein
dumof01837	PROKKA_01859 hypothetical protein
dumof01852	PROKKA_01874 hypothetical protein
dumof01884	PROKKA_01906 hypothetical protein
dumof01887	PROKKA_01909 hypothetical protein
dumof01934	PROKKA_01956 Potassium-transporting ATPase B chain
dumof01990	PROKKA_02013 putative cadmium-transporting ATPase
dumof01999	PROKKA_02022 hypothetical protein
dumof02011	PROKKA_02034 hypothetical protein
dumof02046	PROKKA_02070 hypothetical protein
dumof02061	PROKKA_02085 hypothetical protein
dumof02082	PROKKA_02106 3-hydroxy-3-methylglutaryl-coenzyme A reductase
dumof02083	PROKKA_02107 hypothetical protein

dumof02085	PROKKA_02109 mevalonate kinase
dumof02087	PROKKA_02111 prenyltransferase
dumof02089	PROKKA_02113 Ditrans,polycis-undecaprenyl-diphosphate synthase ((2E,6E)-farnesyl-diphosphate specific)
dumof02091	PROKKA_02115 hypothetical protein
dumof02130	PROKKA_02154 putative acetyltransferase
dumof02168	PROKKA_02192 hypothetical protein
dumof02180	PROKKA_02204 hypothetical protein
dumof02184	PROKKA_02208 hypothetical protein
dumof02188	PROKKA_02212 hypothetical protein
dumof02190	PROKKA_02214 hypothetical protein
dumof02196	PROKKA_02220 hypothetical protein
dumof02198	PROKKA_02222 2-isopropylmalate synthase
dumof02201	PROKKA_02225 hypothetical protein
dumof02214	PROKKA_02238 PhoP regulatory network protein YrbL
dumof02226	PROKKA_02250 hypothetical protein
dumof02237	PROKKA_02261 hypothetical protein
dumof02247	PROKKA_02271 hypothetical protein
dumof02250	PROKKA_02274 hypothetical protein
dumof02253	PROKKA_02277 hypothetical protein
dumof02281	PROKKA_02305 hypothetical protein
dumof02296	PROKKA_02320 3D-(3,5/4)-trihydroxycyclohexane-1,2-dione hydrolase
dumof02297	PROKKA_02321 3D-(3,5/4)-trihydroxycyclohexane-1,2-dione hydrolase
dumof02311	PROKKA_02335 hypothetical protein
dumof02333	PROKKA_02357 hypothetical protein
dumof02555	PROKKA_02581 aerobic respiration control sensor protein ArcB
dumof02608	PROKKA_02634 hypothetical protein
dumof02613	PROKKA_02639 hypothetical protein
dumof02628	PROKKA_02654 hypothetical protein
dumof02642	PROKKA_02668 hypothetical protein
dumof02646	PROKKA_02672 hypothetical protein

dumof02649	PROKKA_02675 hypothetical protein
dumof02653	PROKKA_02679 hypothetical protein
dumof02671	PROKKA_02697 hypothetical protein
dumof02682	PROKKA_02708 hypothetical protein
dumof02685	PROKKA_02711 hypothetical protein
dumof02690	PROKKA_02716 hypothetical protein
dumof02747	PROKKA_02773 hypothetical protein
dumof02940	PROKKA_02966 High-affinity nickel transport protein
dumof02946	PROKKA_02972 hypothetical protein
dumof03005	PROKKA_03035 hypothetical protein
dumof03077	PROKKA_03108 hypothetical protein
dumof03084	PROKKA_03115 hypothetical protein
dumof03093	PROKKA_03124 hypothetical protein
dumof03127	PROKKA_03158 hypothetical protein
dumof03179	PROKKA_03211 hypothetical protein
dumof03182	PROKKA_03214 hypothetical protein
dumof03183	PROKKA_03215 hypothetical protein
dumof03210	PROKKA_03245 hypothetical protein
dumof03226	PROKKA_03261 putative cysteine protease (OTU family)
dumof03248	PROKKA_03284 hypothetical protein
dumof03261	PROKKA_03297 Multidrug resistance protein B
dumof03313	PROKKA_03349 hypothetical protein
dumof03413	PROKKA_03449 hypothetical protein
dumof03429	PROKKA_03466 hypothetical protein
dumof03436	PROKKA_03473 hypothetical protein
dumof03437	PROKKA_03474 hypothetical protein
dumof03438	PROKKA_03475 Site-specific recombinase XerC
dumof03442	PROKKA_03479 hypothetical protein
NSW150_00006	NSW150_00006 hypothetical protein
NSW150_00024	NSW150_00024 putative acyl-CoA thioester hydrolase

NSW150_00030	NSW150_00030 hypothetical protein
NSW150_00032	NSW150_00032 hypothetical protein
NSW150_00033	NSW150_00033 hypothetical protein
NSW150_00050	NSW150_00050 hypothetical protein
NSW150_00055	NSW150_00055 hypothetical protein
NSW150_00056	NSW150_00056 hypothetical protein
NSW150_00060	NSW150_00060 hypothetical protein
NSW150_00096	NSW150_00096 hypothetical protein
NSW150_00098	NSW150_00098 hypothetical protein
NSW150_00099	NSW150_00099 hypothetical protein
NSW150_00110	NSW150_00110 L-rhamnose operon regulatory protein RhaS
NSW150_00111	NSW150_00111 RNA polymerase factor sigma-70
NSW150_00112	NSW150_00112 hypothetical protein
NSW150_00118	NSW150_00118 hypothetical protein
NSW150_00121	NSW150_00121 DNA mismatch repair protein
NSW150_00241	NSW150_00241 hypothetical protein
NSW150_00256	NSW150_00256 RasGEF domain protein
NSW150_00571	NSW150_00582 hypothetical protein
NSW150_00598	NSW150_00612 hypothetical protein
NSW150_00660	NSW150_00675 DNA polymerase IV
NSW150_00678	NSW150_00693 Phenylalanine-specific permease
NSW150_00679	NSW150_00694 Amino-acid permease RocE
NSW150_00684	NSW150_00699 hypothetical protein
NSW150_00686	NSW150_00701 hypothetical protein
NSW150_00687	NSW150_00702 hypothetical protein
NSW150_00688	NSW150_00703 hypothetical protein
NSW150_00689	NSW150_00704 hypothetical protein
NSW150_00693	NSW150_00708 Cupin domain protein
NSW150_00697	NSW150_00712 putative endonuclease containing a URI domain protein
NSW150_00704	NSW150_00719 Outer membrane protein transport protein (OMPP1/FadL/TodX)

NSW150_00709	NSW150_00724 hypothetical protein
NSW150_00713	NSW150_00728 hypothetical protein
NSW150_00724	NSW150_00739 hypothetical protein
NSW150_00743	NSW150_00758 hypothetical protein
NSW150_00750	NSW150_00765 hypothetical protein
NSW150_00794	NSW150_00809 hypothetical protein
NSW150_00799	NSW150_00814 putative endonuclease containing a URI domain protein
NSW150_00800	NSW150_00815 tRNA(1-methyladenosine) methyltransferase
NSW150_00806	NSW150_00821 hypothetical protein
NSW150_00810	NSW150_00825 hypothetical protein
NSW150_00889	NSW150_00904 hypothetical protein
NSW150_00890	NSW150_00905 hypothetical protein
NSW150_00909	NSW150_00924 GSCFA family protein
NSW150_00923	NSW150_00938 hypothetical protein
NSW150_00947	NSW150_00963 hypothetical protein
NSW150_00969	NSW150_00985 3-oxoacyl-[acyl-carrier-protein] reductase FabG
NSW150_00971	NSW150_00987 hypothetical protein
NSW150_00972	NSW150_00988 hypothetical protein
NSW150_00975	NSW150_00992 hypothetical protein
NSW150_00979	NSW150_00996 hypothetical protein
NSW150_00980	NSW150_00997 hypothetical protein
NSW150_00982	NSW150_00999 Crotonyl-CoA reductase
NSW150_00985	NSW150_01002 putative long-chain-fatty-acid--CoA ligase FadD23
NSW150_01003	NSW150_01020 hypothetical protein
NSW150_01062	NSW150_01079 hypothetical protein
NSW150_01071	NSW150_01088 hypothetical protein
NSW150_01072	NSW150_01089 hypothetical protein
NSW150_01098	NSW150_01115 hypothetical protein
NSW150_01099	NSW150_01116 hypothetical protein
NSW150_01100	NSW150_01117 hypothetical protein

NSW150_01139	NSW150_01156 hypothetical protein
NSW150_01169	NSW150_01186 Endo-polygalacturonase precursor
NSW150_01257	NSW150_01274 hypothetical protein
NSW150_01277	NSW150_01294 hypothetical protein
NSW150_01296	NSW150_01324 hypothetical protein
NSW150_01313	NSW150_01342 hypothetical protein
NSW150_01315	NSW150_01344 hypothetical protein
NSW150_01344	NSW150_01373 hypothetical protein
NSW150_01364	NSW150_01393 hypothetical protein
NSW150_01376	NSW150_01405 hypothetical protein
NSW150_01378	NSW150_01407 hypothetical protein
NSW150_01379	NSW150_01408 NH(3)-dependent NAD(+) synthetase
NSW150_01380	NSW150_01409 Cell-division control histidine kinase PdhS
NSW150_01381	NSW150_01410 hypothetical protein
NSW150_01382	NSW150_01411 DNA-binding response regulator MtrA
NSW150_01398	NSW150_01427 hypothetical protein
NSW150_01400	NSW150_01429 hypothetical protein
NSW150_01431	NSW150_01460 hypothetical protein
NSW150_01435	NSW150_01464 hypothetical protein
NSW150_01459	NSW150_01489 hypothetical protein
NSW150_01478	NSW150_01508 hypothetical protein
NSW150_01511	NSW150_01541 hypothetical protein
NSW150_01548	NSW150_01578 hypothetical protein
NSW150_01558	NSW150_01588 hypothetical protein
NSW150_01571	NSW150_01601 hypothetical protein
NSW150_01583	NSW150_01613 hypothetical protein
NSW150_01601	NSW150_01631 hypothetical protein
NSW150_01602	NSW150_01632 hypothetical protein
NSW150_01610	NSW150_01640 hypothetical protein
NSW150_01621	NSW150_01651 hypothetical protein

NSW150_01643	NSW150_01673 hypothetical protein
NSW150_01646	NSW150_01676 hypothetical protein
NSW150_01648	NSW150_01678 biotin biosynthesis protein BioC
NSW150_01652	NSW150_01682 hypothetical protein
NSW150_01654	NSW150_01684 hypothetical protein
NSW150_01690	NSW150_01720 hypothetical protein
NSW150_01703	NSW150_01733 Glycosyl hydrolases family 28
NSW150_01712	NSW150_01742 hypothetical protein
NSW150_01725	NSW150_01755 hypothetical protein
NSW150_01804	NSW150_01834 hypothetical protein
NSW150_01835	NSW150_01867 bifunctional 3-demethylubiquinone-9 3-methyltransferase/ 2-octaprenyl-6-hydroxy phenol me
NSW150_01836	NSW150_01868 hypothetical protein
NSW150_01837	NSW150_01869 hypothetical protein
NSW150_01838	NSW150_01870 hypothetical protein
NSW150_01840	NSW150_01872 hypothetical protein
NSW150_01841	NSW150_01873 hypothetical protein
NSW150_01867	NSW150_01899 hypothetical protein
NSW150_01877	NSW150_01909 hypothetical protein
NSW150_01878	NSW150_01910 hypothetical protein
NSW150_01897	NSW150_01929 putative cyanobacterial protein
NSW150_01898	NSW150_01930 hypothetical protein
NSW150_01899	NSW150_01931 Bacterial leucyl aminopeptidase precursor
NSW150_01900	NSW150_01932 Bacterial leucyl aminopeptidase precursor
NSW150_01902	NSW150_01934 Prolyl endopeptidase
NSW150_01904	NSW150_01936 hypothetical protein
NSW150_01905	NSW150_01937 hypothetical protein
NSW150_01906	NSW150_01938 putative signal-transduction protein containing cAMP-binding and CBS domains
NSW150_01912	NSW150_01944 hypothetical protein
NSW150_01928	NSW150_01960 hypothetical protein
NSW150_01929	NSW150_01961 hypothetical protein

NSW150_01935	NSW150_01967 hypothetical protein
NSW150_01936	NSW150_01968 hypothetical protein
NSW150_01948	NSW150_01980 hypothetical protein
NSW150_01950	NSW150_01982 hypothetical protein
NSW150_01951	NSW150_01983 hypothetical protein
NSW150_01986	NSW150_02019 hypothetical protein
NSW150_01987	NSW150_02020 hypothetical protein
NSW150_01989	NSW150_02022 hypothetical protein
NSW150_01992	NSW150_02025 hypothetical protein
NSW150_01994	NSW150_02027 hypothetical protein
NSW150_01995	NSW150_02028 hypothetical protein
NSW150_02003	NSW150_02036 Isopenicillin N epimerase
NSW150_02004	NSW150_02037 Kynurenine 3-monooxygenase
NSW150_02005	NSW150_02038 hypothetical protein
NSW150_02009	NSW150_02042 Calcineurin-like phosphoesterase
NSW150_02013	NSW150_02046 hypothetical protein
NSW150_02018	NSW150_02051 hypothetical protein
NSW150_02021	NSW150_02054 hypothetical protein
NSW150_02056	NSW150_02089 hypothetical protein
NSW150_02067	NSW150_02100 hypothetical protein
NSW150_02108	NSW150_02145 hypothetical protein
NSW150_02109	NSW150_02146 hypothetical protein
NSW150_02115	NSW150_02152 Putative multidrug export ATP-binding/permease protein
NSW150_02123	NSW150_02160 hypothetical protein
NSW150_02146	NSW150_02183 hypothetical protein
NSW150_02147	NSW150_02184 hypothetical protein
NSW150_02149	NSW150_02186 hypothetical protein
NSW150_02162	NSW150_02199 hypothetical protein
NSW150_02165	NSW150_02202 hypothetical protein
NSW150_02174	NSW150_02211 hypothetical protein

NSW150_02186	NSW150_02223 hypothetical protein
NSW150_02187	NSW150_02224 hypothetical protein
NSW150_02188	NSW150_02225 hypothetical protein
NSW150_02196	NSW150_02233 hypothetical protein
NSW150_02199	NSW150_02236 hypothetical protein
NSW150_02215	NSW150_02252 hypothetical protein
NSW150_02216	NSW150_02253 hypothetical protein
NSW150_02220	NSW150_02257 hypothetical protein
NSW150_02228	NSW150_02265 hypothetical protein
NSW150_02238	NSW150_02275 Putative NADH-flavin reductase
NSW150_02240	NSW150_02277 L-lactate dehydrogenase [cytochrome]
NSW150_02259	NSW150_02296 hypothetical protein
NSW150_02268	NSW150_02305 Cyn operon transcriptional activator
NSW150_02282	NSW150_02319 hypothetical protein
NSW150_02284	NSW150_02321 (S)-2-haloacid dehalogenase 4A
NSW150_02287	NSW150_02324 hypothetical protein
NSW150_02304	NSW150_02341 short chain dehydrogenase
NSW150_02307	NSW150_02344 hypothetical protein
NSW150_02311	NSW150_02348 hypothetical protein
NSW150_02340	NSW150_02377 small GTP-binding protein domain protein
NSW150_02361	NSW150_02399 Transposase for transposon Tn5
NSW150_02365	NSW150_02403 lysine-arginine-ornithine-binding periplasmic protein
NSW150_02370	NSW150_02408 hypothetical protein
NSW150_02385	NSW150_02423 Opine dehydrogenase
NSW150_02387	NSW150_02425 ParB-like nuclease domain protein
NSW150_02388	NSW150_02426 putative siderophore biosynthesis protein SbnA
NSW150_02389	NSW150_02427 Iron-uptake system permease protein FeuC
NSW150_02392	NSW150_02430 High-copy suppressor of rspA
NSW150_02433	NSW150_02472 hypothetical protein
NSW150_02438	NSW150_02477 hypothetical protein

NSW150_02487	NSW150_02527 hypothetical protein
NSW150_02523	NSW150_02563 hypothetical protein
NSW150_02545	NSW150_02585 hypothetical protein
NSW150_02554	NSW150_02594 hypothetical protein
NSW150_02555	NSW150_02595 hypothetical protein
NSW150_02568	NSW150_02608 hypothetical protein
NSW150_02668	NSW150_02709 hypothetical protein
NSW150_02682	NSW150_02724 Virulence metalloprotease precursor
NSW150_02727	NSW150_02769 hypothetical protein
NSW150_02778	NSW150_02820 hypothetical protein
NSW150_02779	NSW150_02821 hypothetical protein
NSW150_02783	NSW150_02825 hypothetical protein
NSW150_02784	NSW150_02826 hypothetical protein
NSW150_02786	NSW150_02828 hypothetical protein
NSW150_02787	NSW150_02829 hypothetical protein
NSW150_02813	NSW150_02855 hypothetical protein
NSW150_02835	NSW150_02877 hypothetical protein
NSW150_02840	NSW150_02882 hypothetical protein
NSW150_02846	NSW150_02888 hypothetical protein
NSW150_02847	NSW150_02889 hypothetical protein
NSW150_02851	NSW150_02893 hypothetical protein
NSW150_02860	NSW150_02902 hypothetical protein
NSW150_02883	NSW150_02926 putative metallophosphoesterase YhaO
NSW150_02884	NSW150_02927 chromosome segregation protein
NSW150_02905	NSW150_02948 hypothetical protein
NSW150_02920	NSW150_02963 cytochrome c biogenesis protein CcmA
NSW150_02921	NSW150_02964 alpha-L-glutamate ligases, RimK family
NSW150_02922	NSW150_02965 hypothetical protein
NSW150_02932	NSW150_02975 hypothetical protein
NSW150_02933	NSW150_02976 Inner membrane transport protein YdhC

NSW150_02934	NSW150_02977 hypothetical protein
NSW150_02952	NSW150_02995 hypothetical protein
NSW150_02962	NSW150_03005 hypothetical protein
NSW150_02967	NSW150_03010 hypothetical protein
NSW150_02969	NSW150_03012 hypothetical protein
NSW150_02970	NSW150_03013 Universal stress protein family protein
NSW150_02975	NSW150_03018 Site-specific recombinase XerD
NSW150_02979	NSW150_03022 hypothetical protein
NSW150_02981	NSW150_03024 hypothetical protein
NSW150_02983	NSW150_03026 Raf kinase inhibitor-like protein, YbhB/YbcL family
NSW150_02984	NSW150_03027 High-affinity zinc uptake system membrane protein ZnuB
NSW150_02985	NSW150_03028 Zinc import ATP-binding protein ZnuC
NSW150_02993	NSW150_03036 hypothetical protein
NSW150_02994	NSW150_03037 Phosphoserine phosphatase 1
NSW150_03000	NSW150_03043 hypothetical protein
NSW150_03019	NSW150_03062 hypothetical protein
NSW150_03023	NSW150_03066 Chondramide synthase cmdD
NSW150_03024	NSW150_03067 Beta-ketoacyl-acyl-carrier-protein synthase I
NSW150_03056	NSW150_03099 hypothetical protein
NSW150_03061	NSW150_03104 hypothetical protein
NSW150_03091	NSW150_03134 Patatin
NSW150_03104	NSW150_03147 hypothetical protein
NSW150_03117	NSW150_03160 hypothetical protein
NSW150_03158	NSW150_03201 hypothetical protein
NSW150_03161	NSW150_03204 hypothetical protein
NSW150_03162	NSW150_03205 polysaccharide export protein Wza
NSW150_03164	NSW150_03207 Polysialic acid transport protein KpsM
NSW150_03169	NSW150_03212 Capsular polysaccharide biosynthesis protein
NSW150_03170	NSW150_03213 hypothetical protein
NSW150_03172	NSW150_03215 hypothetical protein

NSW150_03174	NSW150_03217 putative glycosyl transferase
NSW150_03177	NSW150_03220 Gluconate 5-dehydrogenase
NSW150_03181	NSW150_03224 hypothetical protein
NSW150_03182	NSW150_03225 hypothetical protein
NSW150_03183	NSW150_03226 L-arabinolactonase
NSW150_03190	NSW150_03233 hypothetical protein
NSW150_03191	NSW150_03234 diacylglycerol glucosyltransferase
NSW150_03235	NSW150_03278 hypothetical protein
NSW150_03242	NSW150_03285 hypothetical protein
NSW150_03247	NSW150_03290 hypothetical protein
NSW150_03260	NSW150_03303 Bacteriophytochrome cph2
NSW150_03261	NSW150_03304 hypothetical protein
NSW150_03262	NSW150_03305 hypothetical protein
NSW150_03263	NSW150_03306 hypothetical protein
NSW150_03264	NSW150_03307 hypothetical protein
NSW150_03272	NSW150_03315 hypothetical protein
NSW150_03275	NSW150_03318 hypothetical protein
NSW150_03276	NSW150_03319 hypothetical protein
NSW150_03300	NSW150_03345 Adenosine monophosphate-protein transferase VbhT
NSW150_03347	NSW150_03392 Putative ABC transporter arginine-binding protein 2 precursor
NSW150_03355	NSW150_03400 hypothetical protein
NSW150_03356	NSW150_03401 hypothetical protein
NSW150_03372	NSW150_03418 hypothetical protein
NSW150_03376	NSW150_03422 hypothetical protein
NSW150_03413	NSW150_03460 hypothetical protein
NSW150_03448	NSW150_03495 hypothetical protein
NSW150_03452	NSW150_03499 hypothetical protein
NSW150_03467	NSW150_03514 hypothetical protein
NSW150_03468	NSW150_03515 hypothetical protein
Tucson00038	PROKKA_00038 hypothetical protein

Tucson00059	PROKKA_00059 hypothetical protein
Tucson00073	PROKKA_00073 hypothetical protein
Tucson00081	PROKKA_00081 Hydroxylaminobenzene mutase HabB
Tucson00082	PROKKA_00082 hypothetical protein
Tucson00131	PROKKA_00131 hypothetical protein
Tucson00144	PROKKA_00144 hypothetical protein
Tucson00147	PROKKA_00147 hypothetical protein
Tucson00148	PROKKA_00148 hypothetical protein
Tucson00152	PROKKA_00152 hypothetical protein
Tucson00196	PROKKA_00196 16S ribosomal RNA methyltransferase KsgA/Dim1 family protein
Tucson00224	PROKKA_00224 hypothetical protein
Tucson00225	PROKKA_00225 Putative universal stress protein
Tucson00234	PROKKA_00234 hypothetical protein
Tucson00236	PROKKA_00236 Putative multidrug export ATP-binding/permease protein
Tucson00237	PROKKA_00237 SH2 domain protein
Tucson00245	PROKKA_00248 hypothetical protein
Tucson00246	PROKKA_00249 hypothetical protein
Tucson00298	PROKKA_00302 hypothetical protein
Tucson00299	PROKKA_00303 hypothetical protein
Tucson00410	PROKKA_00414 hypothetical protein
Tucson00411	PROKKA_00415 hypothetical protein
Tucson00412	PROKKA_00416 hypothetical protein
Tucson00528	PROKKA_00543 hypothetical protein
Tucson00617	PROKKA_00633 hypothetical protein
Tucson00644	PROKKA_00660 putative HKD family nuclease
Tucson00655	PROKKA_00671 hypothetical protein
Tucson00657	PROKKA_00673 DNA-dependent helicase II
Tucson00660	PROKKA_00676 hypothetical protein
Tucson00665	PROKKA_00681 hypothetical protein
Tucson00666	PROKKA_00682 hypothetical protein

Tucson00670	PROKKA_00686 hypothetical protein
Tucson00677	PROKKA_00693 hypothetical protein
Tucson00788	PROKKA_00804 hypothetical protein
Tucson00818	PROKKA_00834 hypothetical protein
Tucson00853	PROKKA_00869 hypothetical protein
Tucson00880	PROKKA_00896 drug resistance MFS transporter, drug:H+ antiporter-2 (14 Spanner) (DHA2) family
Tucson00889	PROKKA_00905 hypothetical protein
Tucson00986	PROKKA_01003 hypothetical protein
Tucson01002	PROKKA_01019 hypothetical protein
Tucson01003	PROKKA_01020 putative ABC transporter ATP-binding protein
Tucson01014	PROKKA_01031 hypothetical protein
Tucson01016	PROKKA_01033 hypothetical protein
Tucson01020	PROKKA_01037 hypothetical protein
Tucson01028	PROKKA_01045 hypothetical protein
Tucson01031	PROKKA_01048 hypothetical protein
Tucson01032	PROKKA_01049 hypothetical protein
Tucson01033	PROKKA_01050 hypothetical protein
Tucson01034	PROKKA_01051 hypothetical protein
Tucson01035	PROKKA_01052 Major Facilitator Superfamily protein
Tucson01039	PROKKA_01056 hypothetical protein
Tucson01068	PROKKA_01087 hypothetical protein
Tucson01121	PROKKA_01140 hypothetical protein
Tucson01150	PROKKA_01169 mercuric reductase
Tucson01159	PROKKA_01178 hypothetical protein
Tucson01160	PROKKA_01179 hypothetical protein
Tucson01173	PROKKA_01192 amidase
Tucson01186	PROKKA_01205 hypothetical protein
Tucson01187	PROKKA_01206 hypothetical protein
Tucson01245	PROKKA_01264 hypothetical protein
Tucson01290	PROKKA_01309 hypothetical protein

Tucson01308	PROKKA_01327 High-copy suppressor of rspA
Tucson01398	PROKKA_01417 hypothetical protein
Tucson01418	PROKKA_01437 Glutamate-aspartate carrier protein
Tucson01440	PROKKA_01459 hypothetical protein
Tucson01441	PROKKA_01460 hypothetical protein
Tucson01442	PROKKA_01461 hypothetical protein
Tucson01443	PROKKA_01462 hypothetical protein
Tucson01444	PROKKA_01463 hypothetical protein
Tucson01445	PROKKA_01464 hypothetical protein
Tucson01487	PROKKA_01507 putative ABC transporter ATP-binding protein
Tucson01533	PROKKA_01554 hypothetical protein
Tucson01566	PROKKA_01587 hypothetical protein
Tucson01580	PROKKA_01601 hypothetical protein
Tucson01630	PROKKA_01651 hypothetical protein
Tucson01631	PROKKA_01652 hypothetical protein
Tucson01638	PROKKA_01659 hypothetical protein
Tucson01671	PROKKA_01692 hypothetical protein
Tucson01758	PROKKA_01780 hypothetical protein
Tucson01759	PROKKA_01781 hypothetical protein
Tucson01761	PROKKA_01783 hypothetical protein
Tucson01769	PROKKA_01791 hypothetical protein
Tucson01773	PROKKA_01795 hypothetical protein
Tucson01787	PROKKA_01809 hypothetical protein
Tucson01794	PROKKA_01816 hypothetical protein
Tucson01802	PROKKA_01824 hypothetical protein
Tucson01807	PROKKA_01830 hypothetical protein
Tucson01840	PROKKA_01863 hypothetical protein
Tucson01856	PROKKA_01880 hypothetical protein
Tucson01857	PROKKA_01881 ATP-dependent DNA ligase
Tucson01868	PROKKA_01892 hypothetical protein

Tucson01873	PROKKA_01897 hypothetical protein
Tucson01883	PROKKA_01907 hypothetical protein
Tucson01885	PROKKA_01909 hypothetical protein
Tucson01886	PROKKA_01910 hypothetical protein
Tucson01910	PROKKA_01934 hypothetical protein
Tucson01964	PROKKA_01988 hypothetical protein
Tucson01978	PROKKA_02002 Universal stress protein E
Tucson01994	PROKKA_02018 hypothetical protein
Tucson02008	PROKKA_02032 hypothetical protein
Tucson02057	PROKKA_02081 hypothetical protein
Tucson02071	PROKKA_02095 hypothetical protein
Tucson02086	PROKKA_02110 Adenine deaminase
Tucson02147	PROKKA_02171 hypothetical protein
Tucson02151	PROKKA_02175 hypothetical protein
Tucson02202	PROKKA_02227 hypothetical protein
Tucson02207	PROKKA_02235 hypothetical protein
Tucson02211	PROKKA_02239 hypothetical protein
Tucson02271	PROKKA_02299 hypothetical protein
Tucson02299	PROKKA_02328 hypothetical protein
Tucson02385	PROKKA_02414 hypothetical protein
Tucson02386	PROKKA_02415 hypothetical protein
Tucson02395	PROKKA_02424 integral membrane protein MviN
Tucson02396	PROKKA_02425 Integrase core domain protein
Tucson02402	PROKKA_02431 hypothetical protein
Tucson02403	PROKKA_02432 hypothetical protein
Tucson02465	PROKKA_02496 hypothetical protein
Tucson02480	PROKKA_02511 hypothetical protein
Tucson02503	PROKKA_02535 Prophage CP4-57 integrase
Tucson02526	PROKKA_02559 hypothetical protein
Tucson02527	PROKKA_02560 hypothetical protein

Tucson02565	PROKKA_02598 Chromosomal replication initiator protein DnaA
Tucson02590	PROKKA_02623 hypothetical protein
Tucson02607	PROKKA_02640 Glycosyl transferases group 1
Tucson02609	PROKKA_02642 hypothetical protein
Tucson02614	PROKKA_02647 O-acetyltransferase OatA
Tucson02617	PROKKA_02650 hypothetical protein
Tucson02643	PROKKA_02676 hypothetical protein
Tucson02644	PROKKA_02677 polysaccharide deacetylase family sporulation protein PdaB
Tucson02702	PROKKA_02737 hypothetical protein
Tucson02726	PROKKA_02761 hypothetical protein
Tucson02735	PROKKA_02770 Cell division ATP-binding protein FtsE
Tucson02762	PROKKA_02797 hypothetical protein
Tucson02790	PROKKA_02826 hypothetical protein
Tucson02791	PROKKA_02827 hypothetical protein
Tucson02795	PROKKA_02831 hypothetical protein
Tucson02797	PROKKA_02833 putative thiol peroxidase
Tucson02798	PROKKA_02834 hypothetical protein
Tucson02801	PROKKA_02837 hypothetical protein
Tucson02822	PROKKA_02858 hypothetical protein
Tucson02824	PROKKA_02860 hypothetical protein
Tucson02828	PROKKA_02864 hypothetical protein
Tucson02846	PROKKA_02882 hypothetical protein
Tucson02855	PROKKA_02891 hypothetical protein
Tucson02922	PROKKA_02958 hypothetical protein
Tucson02924	PROKKA_02960 hypothetical protein
Tucson02926	PROKKA_02962 Laccase domain protein YfiH
Tucson02954	PROKKA_02990 hypothetical protein
Tucson02956	PROKKA_02992 hypothetical protein
Tucson02958	PROKKA_02994 hypothetical protein
Tucson02967	PROKKA_03003 hypothetical protein

Tucson02973	PROKKA_03009 Formamidase
Tucson02994	PROKKA_03031 hypothetical protein
Tucson02996	PROKKA_03033 hypothetical protein
wadswor00004	wadswor_00004 hypothetical protein
wadswor00039	wadswor_00041 hypothetical protein
wadswor00061	wadswor_00063 hypothetical protein
wadswor00089	wadswor_00092 hypothetical protein
wadswor00116	wadswor_00119 hypothetical protein
wadswor00172	wadswor_00176 hypothetical protein
wadswor00185	wadswor_00189 hypothetical protein
wadswor00197	wadswor_00201 hypothetical protein
wadswor00236	wadswor_00241 hypothetical protein
wadswor00237	wadswor_00242 Phosphoglucosamine mutase
wadswor00238	wadswor_00243 Glutamine--fructose-6-phosphate aminotransferase [isomerizing]
wadswor00281	wadswor_00286 hypothetical protein
wadswor00304	wadswor_00309 hypothetical protein
wadswor00306	wadswor_00311 hypothetical protein
wadswor00309	wadswor_00314 hypothetical protein
wadswor00310	wadswor_00315 Membrane protein of unknown function
wadswor00311	wadswor_00316 hypothetical protein
wadswor00318	wadswor_00323 Sel1 repeat
wadswor00319	wadswor_00324 hypothetical protein
wadswor00369	wadswor_00374 hypothetical protein
wadswor00380	wadswor_00385 hypothetical protein
wadswor00399	wadswor_00404 hypothetical protein
wadswor00401	wadswor_00406 hypothetical protein
wadswor00407	wadswor_00412 hypothetical protein
wadswor00413	wadswor_00418 hypothetical protein
wadswor00426	wadswor_00431 hypothetical protein
wadswor00505	wadswor_00510 Chaperone protein DnaJ

wadswor00506	wadswor_00511 hypothetical protein
wadswor00509	wadswor_00514 hypothetical protein
wadswor00518	wadswor_00523 hypothetical protein
wadswor00689	wadswor_00695 hypothetical protein
wadswor00702	wadswor_00708 Glutathione-independent formaldehyde dehydrogenase
wadswor00721	wadswor_00727 Glucan endo-1,3-beta-glucosidase A1 precursor
wadswor00727	wadswor_00733 hypothetical protein
wadswor00739	wadswor_00746 hypothetical protein
wadswor00766	wadswor_00774 putative membrane-bound dolichyl-phosphate-mannose-protein mannosyltransferase
wadswor00770	wadswor_00778 hypothetical protein
wadswor00772	wadswor_00780 TspO/MBR family protein
wadswor00791	wadswor_00800 Putative fatty-acid--CoA ligase fadD21
wadswor00792	wadswor_00801 8-amino-7-oxononanoate synthase
wadswor00809	wadswor_00818 hypothetical protein
wadswor00811	wadswor_00820 hypothetical protein
wadswor00812	wadswor_00821 hypothetical protein
wadswor00813	wadswor_00822 hypothetical protein
wadswor00816	wadswor_00825 Pur regulon 18 kDa protein
wadswor00840	wadswor_00850 hypothetical protein
wadswor00871	wadswor_00888 hypothetical protein
wadswor00909	wadswor_00926 Cold acclimation protein B
wadswor00910	wadswor_00927 queuosine biosynthesis protein QueD
wadswor00914	wadswor_00931 hypothetical protein
wadswor00917	wadswor_00934 Putative glycosyltransferase CsbB
wadswor00919	wadswor_00936 Putative acetyltransferase
wadswor00920	wadswor_00937 hypothetical protein
wadswor00990	wadswor_01008 hypothetical protein
wadswor01010	wadswor_01028 hypothetical protein
wadswor01053	wadswor_01071 hypothetical protein
wadswor01060	wadswor_01078 hypothetical protein

wadswor01076	wadswor_01095 hypothetical protein
wadswor01113	wadswor_01132 hypothetical protein
wadswor01114	wadswor_01133 hypothetical protein
wadswor01148	wadswor_01167 hypothetical protein
wadswor01244	wadswor_01270 hypothetical protein
wadswor01266	wadswor_01293 hypothetical protein
wadswor01364	wadswor_01391 hypothetical protein
wadswor01436	wadswor_01463 hypothetical protein
wadswor01438	wadswor_01465 ADP-glyceromanno-heptose 6-epimerase
wadswor01443	wadswor_01470 hypothetical protein
wadswor01444	wadswor_01471 putative peptidoglycan biosynthesis protein MurJ
wadswor01479	wadswor_01506 hypothetical protein
wadswor01575	wadswor_01604 hypothetical protein
wadswor01683	wadswor_01712 hypothetical protein
wadswor01751	wadswor_01780 hypothetical protein
wadswor01767	wadswor_01796 hypothetical protein
wadswor01777	wadswor_01806 hypothetical protein
wadswor01818	wadswor_01847 hypothetical protein
wadswor01897	wadswor_01926 hypothetical protein
wadswor01902	wadswor_01931 hypothetical protein
wadswor01917	wadswor_01946 hypothetical protein
wadswor01945	wadswor_01975 hypothetical protein
wadswor01947	wadswor_01977 antitoxin HipB
wadswor01952	wadswor_01982 hypothetical protein
wadswor01954	wadswor_01984 hypothetical protein
wadswor01963	wadswor_01993 hypothetical protein
wadswor01964	wadswor_01994 hypothetical protein
wadswor01965	wadswor_01995 hypothetical protein
wadswor01966	wadswor_01996 hypothetical protein
wadswor01985	wadswor_02015 hypothetical protein

wadswor02008	wadswor_02038 hypothetical protein
wadswor02074	wadswor_02104 hypothetical protein
wadswor02082	wadswor_02112 hypothetical protein
wadswor02096	wadswor_02126 N-acetyl-alpha-D-glucosaminyl L-malate synthase BshA
wadswor02099	wadswor_02129 hypothetical protein
wadswor02155	wadswor_02185 hypothetical protein
wadswor02190	wadswor_02220 hypothetical protein
wadswor02194	wadswor_02224 hypothetical protein
wadswor02205	wadswor_02235 Divalent metal cation transporter MntH
wadswor02211	wadswor_02241 hypothetical protein
wadswor02286	wadswor_02316 hypothetical protein
wadswor02288	wadswor_02318 hypothetical protein
wadswor02331	wadswor_02361 Amidinotransferase
wadswor02332	wadswor_02362 hypothetical protein
wadswor02366	wadswor_02396 hypothetical protein
wadswor02395	wadswor_02425 2-dehydropantoate 2-reductase
wadswor02401	wadswor_02431 Frizzy aggregation protein FrzCD
wadswor02404	wadswor_02434 F-box domain protein
wadswor02412	wadswor_02442 hypothetical protein
wadswor02416	wadswor_02446 Protein-tyrosine phosphatase
wadswor02421	wadswor_02451 hypothetical protein
wadswor02426	wadswor_02456 hypothetical protein
wadswor02429	wadswor_02459 Polar organelle development protein
wadswor02431	wadswor_02461 DNA-invertase hin
wadswor02432	wadswor_02462 hypothetical protein
wadswor02550	wadswor_02581 Chemotaxis response regulator protein-glutamate methylesterase
wadswor02551	wadswor_02582 Chemotaxis response regulator protein-glutamate methylesterase of group 1 operon
wadswor02607	wadswor_02641 hypothetical protein
wadswor02615	wadswor_02650 hypothetical protein
wadswor02622	wadswor_02657 hypothetical protein

wadswor02627	wadswor_02662 hypothetical protein
wadswor02636	wadswor_02671 hypothetical protein
wadswor02639	wadswor_02674 hypothetical protein
wadswor02648	wadswor_02683 hypothetical protein
wadswor02649	wadswor_02684 hypothetical protein
wadswor02652	wadswor_02687 hypothetical protein
wadswor02653	wadswor_02688 hypothetical protein
wadswor02672	wadswor_02707 hypothetical protein
wadswor02683	wadswor_02718 N-ethylmaleimide reductase
wadswor02689	wadswor_02724 hypothetical protein
wadswor02692	wadswor_02727 Acetyltransferase (GNAT) family protein
wadswor02694	wadswor_02729 hypothetical protein
wadswor02698	wadswor_02733 hypothetical protein
wadswor02702	wadswor_02737 hypothetical protein
wadswor02703	wadswor_02738 hypothetical protein
wadswor02720	wadswor_02755 hypothetical protein
wadswor02721	wadswor_02756 Site-specific recombinase XerD
wadswor02736	wadswor_02771 hypothetical protein
wadswor02757	wadswor_02792 hypothetical protein
wadswor02765	wadswor_02800 hypothetical protein
wadswor02766	wadswor_02801 hypothetical protein
wadswor02778	wadswor_02813 hypothetical protein
wadswor02799	wadswor_02834 hypothetical protein
wadswor02808	wadswor_02843 hypothetical protein
wadswor02880	wadswor_02916 hypothetical protein
wadswor02940	wadswor_02977 hypothetical protein
wadswor02990	wadswor_03027 hypothetical protein
wadswor02998	wadswor_03035 hypothetical protein
wadswor03004	wadswor_03041 hypothetical protein
wadswor03018	wadswor_03055 hypothetical protein

wadswor03020	wadswor_03057 hypothetical protein
wadswor03027	wadswor_03064 hypothetical protein
wadswor03035	wadswor_03072 hypothetical protein
wadswor03042	wadswor_03079 hypothetical protein
wadswor03044	wadswor_03081 hypothetical protein
wadswor03045	wadswor_03082 3-oxoacyl-[acyl-carrier-protein] synthase 3
wadswor03046	wadswor_03083 Acyl carrier protein
wadswor03062	wadswor_03099 hypothetical protein
wadswor03066	wadswor_03103 hypothetical protein
wadswor03067	wadswor_03104 hypothetical protein
wadswor03072	wadswor_03109 Chemotaxis response regulator protein-glutamate methylesterase
wadswor03073	wadswor_03110 Chemotaxis protein methyltransferase
wadswor03081	wadswor_03118 hypothetical protein
wadswor03095	wadswor_03132 hypothetical protein
wadswor03100	wadswor_03137 hypothetical protein
wadswor03104	wadswor_03141 hypothetical protein
wadswor03107	wadswor_03144 hypothetical protein
wadswor03119	wadswor_03156 hypothetical protein
wadswor03121	wadswor_03158 NlpC/P60 family protein
wadswor03130	wadswor_03167 hypothetical protein
wadswor03131	wadswor_03168 hypothetical protein
wadswor03133	wadswor_03170 hypothetical protein
wadswor03134	wadswor_03171 Acetyltransferase (GNAT) family protein
wadswor03147	wadswor_03184 hypothetical protein
wadswor03148	wadswor_03185 hypothetical protein
wadswor03152	wadswor_03189 hypothetical protein
wadswor03160	wadswor_03197 hypothetical protein
wadswor03161	wadswor_03198 hypothetical protein
wadswor03162	wadswor_03199 LemA family protein
wadswor03165	wadswor_03202 hypothetical protein

wadswor03174	wadswor_03211 Ankyrin repeats (3 copies)
wadswor03181	wadswor_03218 hypothetical protein
wadswor03184	wadswor_03221 hypothetical protein
wadswor03191	wadswor_03228 Site-specific recombinase XerD
wadswor03192	wadswor_03229 hypothetical protein
wadswor03194	wadswor_03231 hypothetical protein
wadswor03195	wadswor_03232 hypothetical protein
wadswor03198	wadswor_03235 hypothetical protein
wadswor03209	wadswor_03246 putative DNA-binding transcriptional regulator

Supplementary Table 3d. Unique genes/gene clusters found only in the Clade 4 *Legionella* species

rowbo00003	PROKKA_00003 hypothetical protein
rowbo00004	PROKKA_00004 hypothetical protein
rowbo00005	PROKKA_00005 hypothetical protein
rowbo00008	PROKKA_00008 hypothetical protein
rowbo00014	PROKKA_00014 ankyrin repeat protein
rowbo00015	PROKKA_00015 Multidrug resistance protein MdtL
rowbo00020	PROKKA_00020 hypothetical protein
rowbo00032	PROKKA_00032 hypothetical protein
rowbo00033	PROKKA_00033 hypothetical protein
rowbo00041	PROKKA_00041 hypothetical protein
rowbo00045	PROKKA_00045 Tyrosine recombinase XerC
rowbo00046	PROKKA_00046 hypothetical protein
rowbo00047	PROKKA_00047 hypothetical protein
rowbo00048	PROKKA_00048 hypothetical protein
rowbo00051	PROKKA_00051 hypothetical protein
rowbo00056	PROKKA_00056 hypothetical protein
rowbo00077	PROKKA_00077 hypothetical protein
rowbo00081	PROKKA_00081 hypothetical protein
rowbo00087	PROKKA_00087 hypothetical protein

rowbo00092	PROKKA_00092 CsbD-like protein
rowbo00100	PROKKA_00100 outer membrane channel protein
rowbo00125	PROKKA_00125 hypothetical protein
rowbo00145	PROKKA_00146 Outer membrane protein OmpH
rowbo00146	PROKKA_00147 hypothetical protein
rowbo00156	PROKKA_00157 Alcohol dehydrogenase
rowbo00159	PROKKA_00160 Guanine/hypoxanthine permease PbuG
rowbo00171	PROKKA_00172 hypothetical protein
rowbo00174	PROKKA_00175 hypothetical protein
rowbo00194	PROKKA_00195 L-gulono-1,4-lactone dehydrogenase
rowbo00201	PROKKA_00202 hypothetical protein
rowbo00210	PROKKA_00211 hypothetical protein
rowbo00221	PROKKA_00222 hypothetical protein
rowbo00224	PROKKA_00225 hypothetical protein
rowbo00231	PROKKA_00232 hypothetical protein
rowbo00232	PROKKA_00233 hypothetical protein
rowbo00233	PROKKA_00234 hypothetical protein
rowbo00235	PROKKA_00236 hypothetical protein
rowbo00241	PROKKA_00243 hypothetical protein
rowbo00280	PROKKA_00282 hypothetical protein
rowbo00282	PROKKA_00284 hypothetical protein
rowbo00289	PROKKA_00291 Serine/threonine-protein kinase AfsK
rowbo00304	PROKKA_00306 hypothetical protein
rowbo00324	PROKKA_00326 hypothetical protein
rowbo00327	PROKKA_00329 hypothetical protein
rowbo00336	PROKKA_00338 hypothetical protein
rowbo00345	PROKKA_00347 DNA polymerase III subunit alpha
rowbo00351	PROKKA_00353 hypothetical protein
rowbo00377	PROKKA_00380 Glutathione S-transferase GST-6.0
rowbo00379	PROKKA_00382 Glutathione S-transferase GstA

rowbo00403	PROKKA_00406 Flagellar biosynthetic protein FliP precursor
rowbo00404	PROKKA_00407 Flagellar biosynthetic protein FliP precursor
rowbo00421	PROKKA_00424 flagellar basal body P-ring biosynthesis protein FlgA
rowbo00430	PROKKA_00436 S1/P1 Nuclease
rowbo00438	PROKKA_00444 hypothetical protein
rowbo00443	PROKKA_00449 hypothetical protein
rowbo00458	PROKKA_00473 hypothetical protein
rowbo00497	PROKKA_00512 putative periplasmic ligand-binding sensor domain protein
rowbo00498	PROKKA_00513 GAF domain protein
rowbo00499	PROKKA_00514 Diguanylate cyclase DosC
rowbo00504	PROKKA_00519 hypothetical protein
rowbo00510	PROKKA_00525 hypothetical protein
rowbo00521	PROKKA_00536 hypothetical protein
rowbo00523	PROKKA_00538 hypothetical protein
rowbo00535	PROKKA_00550 hypothetical protein
rowbo00536	PROKKA_00551 hypothetical protein
rowbo00538	PROKKA_00553 hypothetical protein
rowbo00548	PROKKA_00563 hypothetical protein
rowbo00573	PROKKA_00589 hypothetical protein
rowbo00574	PROKKA_00590 hypothetical protein
rowbo00584	PROKKA_00600 hypothetical protein
rowbo00643	PROKKA_00659 hypothetical protein
rowbo00644	PROKKA_00660 hypothetical protein
rowbo00645	PROKKA_00661 hypothetical protein
rowbo00647	PROKKA_00663 hypothetical protein
rowbo00671	PROKKA_00687 hypothetical protein
rowbo00673	PROKKA_00689 adenine phosphoribosyltransferase
rowbo00675	PROKKA_00691 hypothetical protein
rowbo00706	PROKKA_00722 hypothetical protein
rowbo00712	PROKKA_00728 hypothetical protein

rowbo00714	PROKKA_00730 hypothetical protein
rowbo00727	PROKKA_00743 Putative universal stress protein
rowbo00734	PROKKA_00750 hypothetical protein
rowbo00736	PROKKA_00752 3-hydroxy-3-methylglutaryl-coenzyme A reductase
rowbo00737	PROKKA_00753 6,7-dimethyl-8-ribityllumazine synthase
rowbo00746	PROKKA_00762 hypothetical protein
rowbo00756	PROKKA_00772 hypothetical protein
rowbo00757	PROKKA_00773 putative periplasmic ligand-binding sensor domain protein
rowbo00772	PROKKA_00788 hypothetical protein
rowbo00774	PROKKA_00790 hypothetical protein
rowbo00775	PROKKA_00791 hypothetical protein
rowbo00776	PROKKA_00792 hypothetical protein
rowbo00779	PROKKA_00795 hypothetical protein
rowbo00782	PROKKA_00798 hypothetical protein
rowbo00783	PROKKA_00799 hypothetical protein
rowbo00784	PROKKA_00800 hypothetical protein
rowbo00797	PROKKA_00813 hypothetical protein
rowbo00798	PROKKA_00814 hypothetical protein
rowbo00799	PROKKA_00815 hypothetical protein
rowbo00803	PROKKA_00819 hypothetical protein
rowbo00810	PROKKA_00826 hypothetical protein
rowbo00816	PROKKA_00832 hypothetical protein
rowbo00818	PROKKA_00834 hypothetical protein
rowbo00819	PROKKA_00835 hypothetical protein
rowbo00820	PROKKA_00836 hypothetical protein
rowbo00822	PROKKA_00838 hypothetical protein
rowbo00825	PROKKA_00841 hypothetical protein
rowbo00828	PROKKA_00844 hypothetical protein
rowbo00831	PROKKA_00847 hypothetical protein
rowbo00832	PROKKA_00848 3-oxoacyl-[acyl-carrier-protein] synthase 3

rowbo00835	PROKKA_00851 hypothetical protein
rowbo00836	PROKKA_00852 Putative ribosomal N-acetyltransferase YdaF
rowbo00842	PROKKA_00858 hypothetical protein
rowbo00850	PROKKA_00866 hypothetical protein
rowbo00857	PROKKA_00873 hypothetical protein
rowbo00862	PROKKA_00878 hypothetical protein
rowbo00863	PROKKA_00879 hypothetical protein
rowbo00865	PROKKA_00881 hypothetical protein
rowbo00872	PROKKA_00888 ankyrin repeat protein
rowbo00875	PROKKA_00891 hypothetical protein
rowbo00876	PROKKA_00892 hypothetical protein
rowbo00877	PROKKA_00893 hypothetical protein
rowbo00878	PROKKA_00894 Integrase core domain protein
rowbo00892	PROKKA_00908 hypothetical protein
rowbo00893	PROKKA_00909 hypothetical protein
rowbo00894	PROKKA_00910 alkylhydroperoxidase AhpD family core domain protein
rowbo00898	PROKKA_00914 hypothetical protein
rowbo00909	PROKKA_00925 N-succinylglutamate 5-semialdehyde dehydrogenase
rowbo00944	PROKKA_00960 hypothetical protein
rowbo00956	PROKKA_00972 hypothetical protein
rowbo00979	PROKKA_00995 hypothetical protein
rowbo00995	PROKKA_01011 UDP-2,3-diacylglucosamine hydrolase
rowbo00998	PROKKA_01014 hypothetical protein
rowbo01007	PROKKA_01023 hypothetical protein
rowbo01016	PROKKA_01032 hypothetical protein
rowbo01026	PROKKA_01042 hypothetical protein
rowbo01027	PROKKA_01043 hypothetical protein
rowbo01030	PROKKA_01046 Transposase DDE domain protein
rowbo01032	PROKKA_01048 hypothetical protein
rowbo01035	PROKKA_01051 hypothetical protein

rowbo01039	PROKKA_01055 hypothetical protein
rowbo01040	PROKKA_01056 putative chitinase
rowbo01047	PROKKA_01063 hypothetical protein
rowbo01052	PROKKA_01068 hypothetical protein
rowbo01053	PROKKA_01069 hypothetical protein
rowbo01054	PROKKA_01070 N-acyl amino acid synthase, PEP-CTERM/exosortase system-associated
rowbo01064	PROKKA_01080 hypothetical protein
rowbo01068	PROKKA_01084 hypothetical protein
rowbo01072	PROKKA_01088 Lipase (class 3)
rowbo01116	PROKKA_01133 hypothetical protein
rowbo01122	PROKKA_01139 hypothetical protein
rowbo01124	PROKKA_01141 putative ATPase
rowbo01125	PROKKA_01142 hypothetical protein
rowbo01132	PROKKA_01149 hypothetical protein
rowbo01144	PROKKA_01162 Excinuclease ABC subunit B
rowbo01146	PROKKA_01164 hypothetical protein
rowbo01152	PROKKA_01170 efflux transporter, outer membrane factor (OMF) lipoprotein, NodT family
rowbo01180	PROKKA_01198 hypothetical protein
rowbo01186	PROKKA_01204 Autoinducer 2 sensor kinase/phosphatase LuxQ
rowbo01205	PROKKA_01223 hypothetical protein
rowbo01244	PROKKA_01262 UDP-N-acetyl-alpha-D-glucosamine C6 dehydratase
rowbo01268	PROKKA_01286 hypothetical protein
rowbo01285	PROKKA_01305 hypothetical protein
rowbo01320	PROKKA_01340 hypothetical protein
rowbo01321	PROKKA_01341 hypothetical protein
rowbo01331	PROKKA_01351 Excinuclease ABC subunit C
rowbo01335	PROKKA_01355 GTP-binding protein Obg
rowbo01355	PROKKA_01375 hypothetical protein
rowbo01357	PROKKA_01377 Septal ring factor
rowbo01403	PROKKA_01423 hypothetical protein

rowbo01404	PROKKA_01424 ankyrin repeat protein
rowbo01405	PROKKA_01425 hypothetical protein
rowbo01458	PROKKA_01478 Dipeptide and tripeptide permease B
rowbo01462	PROKKA_01482 hypothetical protein
rowbo01477	PROKKA_01497 hypothetical protein
rowbo01504	PROKKA_01524 hypothetical protein
rowbo01516	PROKKA_01536 hypothetical protein
rowbo01522	PROKKA_01542 putative adenylate-forming enzyme
rowbo01531	PROKKA_01551 hypothetical protein
rowbo01544	PROKKA_01564 putative small periplasmic lipoprotein
rowbo01594	PROKKA_01614 hypothetical protein
rowbo01607	PROKKA_01627 Proline porter II
rowbo01669	PROKKA_01690 Tyrocidine synthase I
rowbo01680	PROKKA_01701 Cyanophycin synthetase
rowbo01697	PROKKA_01718 hypothetical protein
rowbo01720	PROKKA_01742 hypothetical protein
rowbo01729	PROKKA_01751 flavoprotein, family
rowbo01736	PROKKA_01758 hypothetical protein
rowbo01741	PROKKA_01763 hypothetical protein
rowbo01757	PROKKA_01779 hypothetical protein
rowbo01762	PROKKA_01784 hypothetical protein
rowbo01776	PROKKA_01798 Ankyrin repeats (3 copies)
rowbo01777	PROKKA_01799 Alkane 1-monooxygenase 2
rowbo01778	PROKKA_01800 HAD hydrolase, family IB
rowbo01780	PROKKA_01802 Putative fatty-acid--CoA ligase fadD21
rowbo01781	PROKKA_01803 Long-chain-fatty-acid--AMP ligase FadD26
rowbo01782	PROKKA_01804 hypothetical protein
rowbo01783	PROKKA_01805 1-acyl-sn-glycerol-3-phosphate acyltransferase
rowbo01802	PROKKA_01824 hypothetical protein
rowbo01805	PROKKA_01827 hypothetical protein

rowbo01810	PROKKA_01832 hypothetical protein
rowbo01815	PROKKA_01837 hypothetical protein
rowbo01824	PROKKA_01846 Inner membrane transport permease YadH
rowbo01825	PROKKA_01847 Daunorubicin/doxorubicin resistance ATP-binding protein DrrA
rowbo01826	PROKKA_01848 Doxorubicin resistance ATP-binding protein DrrA
rowbo01843	PROKKA_01865 hypothetical protein
rowbo01846	PROKKA_01868 hypothetical protein
rowbo01859	PROKKA_01881 hypothetical protein
rowbo01908	PROKKA_01930 conjugative coupling factor TraD, PFGI-1 class
rowbo01910	PROKKA_01932 Nuclease SbcCD subunit D
rowbo01911	PROKKA_01933 Nuclease SbcCD subunit C
rowbo01912	PROKKA_01934 Nuclease SbcCD subunit C
rowbo01920	PROKKA_01942 hypothetical protein
rowbo01927	PROKKA_01949 hypothetical protein
rowbo01930	PROKKA_01952 hypothetical protein
rowbo01940	PROKKA_01962 hypothetical protein
rowbo01941	PROKKA_01963 hypothetical protein
rowbo01944	PROKKA_01966 hypothetical protein
rowbo01949	PROKKA_01971 hypothetical protein
rowbo01953	PROKKA_01975 ankyrin repeat protein
rowbo01991	PROKKA_02013 lipopolysaccharide 1,2-N-acetylglucosaminetransferase
rowbo01992	PROKKA_02014 hypothetical protein
rowbo01994	PROKKA_02016 hypothetical protein
rowbo01995	PROKKA_02017 hypothetical protein
rowbo02011	PROKKA_02033 hypothetical protein
rowbo02025	PROKKA_02047 hypothetical protein
rowbo02052	PROKKA_02074 hypothetical protein
rowbo02065	PROKKA_02087 hypothetical protein
rowbo02075	PROKKA_02097 hypothetical protein
rowbo02076	PROKKA_02098 Pyridoxamine-phosphate oxidase

rowbo02077	PROKKA_02099 Pyridoxine/pyridoxamine 5'-phosphate oxidase
rowbo02083	PROKKA_02105 von Willebrand factor type D domain protein
rowbo02084	PROKKA_02106 hypothetical protein
rowbo02108	PROKKA_02130 ankyrin repeat protein
rowbo02112	PROKKA_02134 Fibronectin type III domain protein
rowbo02152	PROKKA_02174 hypothetical protein
rowbo02161	PROKKA_02183 putative lipase
rowbo02162	PROKKA_02184 Putative cyclic-di-GMP phosphodiesterase AdrB
rowbo02173	PROKKA_02195 hypothetical protein
rowbo02183	PROKKA_02205 Legionella pneumophila major outer membrane protein precursor
rowbo02186	PROKKA_02208 hypothetical protein
rowbo02207	PROKKA_02229 hypothetical protein
rowbo02220	PROKKA_02242 hypothetical protein
rowbo02221	PROKKA_02243 hypothetical protein
rowbo02226	PROKKA_02248 hypothetical protein
rowbo02228	PROKKA_02250 hypothetical protein
rowbo02229	PROKKA_02251 hypothetical protein
rowbo02236	PROKKA_02258 hypothetical protein
rowbo02238	PROKKA_02260 Alginate biosynthesis protein AlgA
rowbo02239	PROKKA_02261 hypothetical protein
rowbo02255	PROKKA_02277 hypothetical protein
rowbo02260	PROKKA_02282 hypothetical protein
rowbo02264	PROKKA_02286 hypothetical protein
rowbo02266	PROKKA_02288 Mandelamide hydrolase
rowbo02267	PROKKA_02289 hypothetical protein
rowbo02268	PROKKA_02290 hypothetical protein
rowbo02269	PROKKA_02291 Putative Ig domain protein
rowbo02276	PROKKA_02298 Beta-lactamase
rowbo02280	PROKKA_02302 hypothetical protein
rowbo02286	PROKKA_02308 hypothetical protein

rowbo02291	PROKKA_02313 hypothetical protein
rowbo02297	PROKKA_02319 hypothetical protein
rowbo02298	PROKKA_02320 hypothetical protein
rowbo02299	PROKKA_02321 hypothetical protein
rowbo02300	PROKKA_02322 hypothetical protein
rowbo02303	PROKKA_02325 hypothetical protein
rowbo02304	PROKKA_02326 hypothetical protein
rowbo02309	PROKKA_02331 hypothetical protein
rowbo02317	PROKKA_02339 hypothetical protein
rowbo02322	PROKKA_02344 hypothetical protein
rowbo02325	PROKKA_02347 Cold acclimation protein B
rowbo02328	PROKKA_02350 hypothetical protein
rowbo02330	PROKKA_02352 hypothetical protein
rowbo02331	PROKKA_02353 hypothetical protein
rowbo02337	PROKKA_02359 Outer membrane protein W precursor
rowbo02346	PROKKA_02368 hypothetical protein
rowbo02355	PROKKA_02377 LigB family dioxygenase
rowbo02358	PROKKA_02380 hypothetical protein
rowbo02360	PROKKA_02382 hypothetical protein
rowbo02361	PROKKA_02383 hypothetical protein
rowbo02372	PROKKA_02394 Kua-ubiquitin conjugating enzyme hybrid localisation domain protein
rowbo02374	PROKKA_02396 hypothetical protein
rowbo02377	PROKKA_02399 hypothetical protein
rowbo02382	PROKKA_02404 hypothetical protein
rowbo02385	PROKKA_02407 putative enzyme related to lactoylglutathione lyase
rowbo02386	PROKKA_02408 hypothetical protein
rowbo02388	PROKKA_02410 hypothetical protein
rowbo02390	PROKKA_02412 hypothetical protein
rowbo02399	PROKKA_02421 hypothetical protein
rowbo02402	PROKKA_02424 hypothetical protein

rowbo02404	PROKKA_02426 hypothetical protein
rowbo02409	PROKKA_02431 hypothetical protein
rowbo02411	PROKKA_02433 hypothetical protein
rowbo02415	PROKKA_02437 DNA-invertase hin
rowbo02420	PROKKA_02442 Nodulation protein S (NodS)
rowbo02460	PROKKA_02482 Peptide chain release factor 2
rowbo02482	PROKKA_02504 hypothetical protein
rowbo02483	PROKKA_02505 poly(A) polymerase
rowbo02501	PROKKA_02523 hypothetical protein
rowbo02506	PROKKA_02528 hypothetical protein
rowbo02508	PROKKA_02530 hypothetical protein
rowbo02516	PROKKA_02538 hypothetical protein
rowbo02524	PROKKA_02546 Acetyl-coenzyme A synthetase
rowbo02535	PROKKA_02557 Glycine--tRNA ligase beta subunit
rowbo02550	PROKKA_02572 hypothetical protein
rowbo02579	PROKKA_02601 hypothetical protein
rowbo02581	PROKKA_02603 Catalase C
rowbo02584	PROKKA_02606 hypothetical protein
rowbo02587	PROKKA_02609 hypothetical protein
rowbo02593	PROKKA_02615 hypothetical protein
rowbo02594	PROKKA_02616 hypothetical protein
rowbo02595	PROKKA_02617 hypothetical protein
rowbo02610	PROKKA_02633 hypothetical protein
rowbo02620	PROKKA_02643 nicotinic acid mononucleotide adenylyltransferase
rowbo02630	PROKKA_02653 hypothetical protein
rowbo02638	PROKKA_02661 hypothetical protein
rowbo02652	PROKKA_02675 Heat shock protein F84.1
rowbo02654	PROKKA_02677 hypothetical protein
rowbo02671	PROKKA_02694 hypothetical protein
rowbo02672	PROKKA_02695 Legionella pneumophila major outer membrane protein precursor

rowbo02677	PROKKA_02700 hypothetical protein
rowbo02757	PROKKA_02782 hypothetical protein
rowbo02760	PROKKA_02785 hypothetical protein
rowbo02762	PROKKA_02787 hypothetical protein
rowbo02767	PROKKA_02793 hypothetical protein
rowbo02776	PROKKA_02802 hypothetical protein
rowbo02791	PROKKA_02817 DnaJ domain protein
rowbo02799	PROKKA_02825 hypothetical protein
rowbo02810	PROKKA_02836 fused phosphoenolpyruvate-protein phosphotransferase PtsP/GAF domain protein
rowbo02815	PROKKA_02841 hypothetical protein
rowbo02817	PROKKA_02843 Ribonuclease
rowbo02818	PROKKA_02844 Ribonuclease
rowbo02831	PROKKA_02857 hypothetical protein
rowbo02838	PROKKA_02864 Zinc-type alcohol dehydrogenase-like protein
rowbo02839	PROKKA_02865 hypothetical protein
rowbo02841	PROKKA_02867 hypothetical protein
rowbo02846	PROKKA_02872 hypothetical protein
rowbo02847	PROKKA_02873 hypothetical protein
rowbo02850	PROKKA_02876 hypothetical protein
rowbo02853	PROKKA_02879 hypothetical protein
rowbo02854	PROKKA_02880 hypothetical protein
rowbo02856	PROKKA_02882 hypothetical protein
rowbo02859	PROKKA_02885 homoserine/Threonine efflux protein
rowbo02860	PROKKA_02886 Putative pyridoxal phosphate-dependent acyltransferase
rowbo02862	PROKKA_02888 putative 2-phosphosulfolactate phosphatase
rowbo02867	PROKKA_02893 hypothetical protein
rowbo02872	PROKKA_02898 hypothetical protein
rowbo02873	PROKKA_02899 Calcium-transporting ATPase
rowbo02880	PROKKA_02906 Putative oxidoreductase SadH
rowbo02881	PROKKA_02907 hypothetical protein

rowbo02882	PROKKA_02908 hypothetical protein
rowbo02889	PROKKA_02915 Glucose-6-phosphate isomerase
rowbo02890	PROKKA_02916 ribosomal-protein-alanine acetyltransferase
rowbo02892	PROKKA_02918 hypothetical protein
rowbo02897	PROKKA_02923 Transposase
rowbo02899	PROKKA_02925 hypothetical protein
rowbo02900	PROKKA_02926 hypothetical protein
rowbo02904	PROKKA_02930 hypothetical protein
rowbo02913	PROKKA_02939 bifunctional ATP-dependent DNA helicase/DNA polymerase III subunit epsilon
rowbo02915	PROKKA_02941 hypothetical protein
rowbo02922	PROKKA_02948 hypothetical protein
rowbo02930	PROKKA_02956 hypothetical protein
rowbo02933	PROKKA_02959 hypothetical protein
rowbo02934	PROKKA_02960 hypothetical protein
rowbo02965	PROKKA_02991 hypothetical protein
rowbo02967	PROKKA_02993 hypothetical protein
rowbo02993	PROKKA_03022 Ubiquitin fusion degradation protein 2
rowbo03000	PROKKA_03029 hypothetical protein
rowbo03012	PROKKA_03041 hypothetical protein
rowbo03017	PROKKA_03046 hypothetical protein
rowbo03024	PROKKA_03053 hypothetical protein
rowbo03043	PROKKA_03072 hypothetical protein
rowbo03061	PROKKA_03090 3-oxoacyl-[acyl-carrier-protein] reductase FabG
rowbo03062	PROKKA_03091 acetyacetyl-CoA reductase
rowbo03066	PROKKA_03095 hypothetical protein
rowbo03068	PROKKA_03097 hypothetical protein
rowbo03085	PROKKA_03114 Region found in RelA / SpoT proteins
rowbo03089	PROKKA_03118 hypothetical protein
rowbo03095	PROKKA_03124 hypothetical protein
rowbo03118	PROKKA_03147 Carbon storage regulator

rowbo03127	PROKKA_03159 hypothetical protein
rowbo03140	PROKKA_03172 hypothetical protein
rowbo03157	PROKKA_03189 hypothetical protein
rowbo03176	PROKKA_03208 hypothetical protein
rowbo03184	PROKKA_03216 hypothetical protein
rowbo03185	PROKKA_03217 hypothetical protein
rowbo03187	PROKKA_03219 hypothetical protein
rowbo03188	PROKKA_03220 hypothetical protein
rowbo03192	PROKKA_03224 hypothetical protein
rowbo03193	PROKKA_03225 hypothetical protein
rowbo03194	PROKKA_03226 hypothetical protein
rowbo03195	PROKKA_03227 Transposase, TnpA family
rowbo03196	PROKKA_03228 hypothetical protein
rowbo03201	PROKKA_03233 hypothetical protein
rowbo03205	PROKKA_03237 hypothetical protein
rowbo03206	PROKKA_03238 hypothetical protein
rowbo03207	PROKKA_03239 Chemotaxis protein methyltransferase
rowbo03210	PROKKA_03242 Chemotaxis regulator BdlA
rowbo03212	PROKKA_03244 Chemotaxis protein CheA
rowbo03215	PROKKA_03247 DNA polymerase IV
rowbo03217	PROKKA_03249 Tyrosine recombinase XerC
rowbo03228	PROKKA_03260 hypothetical protein
rowbo03230	PROKKA_03262 hypothetical protein
rowbo03236	PROKKA_03268 hypothetical protein
rowbo03238	PROKKA_03270 hypothetical protein
rowbo03239	PROKKA_03271 hypothetical protein
rowbo03240	PROKKA_03272 hypothetical protein
rowbo03241	PROKKA_03273 Undecaprenyl-phosphate 4-deoxy-4-formamido-L-arabinose transferase
rowbo03245	PROKKA_03277 Poly-beta-hydroxybutyrate polymerase
rowbo03250	PROKKA_03282 hypothetical protein

rowbo03251	PROKKA_03283 hypothetical protein
rowbo03278	PROKKA_03310 hypothetical protein
rowbo03280	PROKKA_03312 hypothetical protein
rowbo03293	PROKKA_03325 Gliding motility regulatory protein
rowbo03294	PROKKA_03326 CheW-like domain protein
rowbo03295	PROKKA_03327 Chemotaxis protein CheA
rowbo03296	PROKKA_03328 Heme-based aerotactic transducer HemAT
rowbo03298	PROKKA_03330 hypothetical protein
rowbo03304	PROKKA_03336 hypothetical protein
rowbo03305	PROKKA_03337 hypothetical protein
rowbo03308	PROKKA_03340 hypothetical protein
rowbo03314	PROKKA_03346 hypothetical protein
rowbo03316	PROKKA_03348 hypothetical protein
rowbo03317	PROKKA_03349 hypothetical protein
rowbo03318	PROKKA_03350 hypothetical protein
rowbo03320	PROKKA_03352 hypothetical protein
rowbo03321	PROKKA_03353 hypothetical protein
rowbo03322	PROKKA_03354 hypothetical protein
rowbo03326	PROKKA_03358 hypothetical protein
rowbo03328	PROKKA_03360 hypothetical protein
rowbo03330	PROKKA_03362 hypothetical protein
rowbo03331	PROKKA_03363 hypothetical protein
rowbo03332	PROKKA_03364 hypothetical protein
rowbo03347	PROKKA_03379 hypothetical protein
rowbo03350	PROKKA_03382 hypothetical protein
rowbo03352	PROKKA_03384 hypothetical protein
rowbo03355	PROKKA_03387 hypothetical protein
rowbo03366	PROKKA_03398 hypothetical protein
rowbo03368	PROKKA_03400 hypothetical protein
rowbo03369	PROKKA_03401 hypothetical protein

rowbo03370	PROKKA_03402 hypothetical protein
rowbo03371	PROKKA_03403 hypothetical protein
rowbo03374	PROKKA_03406 hypothetical protein
rowbo03375	PROKKA_03407 hypothetical protein
rowbo03376	PROKKA_03408 hypothetical protein
rowbo03381	PROKKA_03413 Ankyrin repeats (3 copies)
rowbo03383	PROKKA_03415 hypothetical protein
rowbo03385	PROKKA_03417 hypothetical protein
rowbo03386	PROKKA_03418 hypothetical protein
rowbo03387	PROKKA_03419 Alpha/beta hydrolase family protein
rowbo03388	PROKKA_03420 hypothetical protein
rowbo03390	PROKKA_03422 hypothetical protein
rowbo03391	PROKKA_03423 hypothetical protein
rowbo03392	PROKKA_03424 ankyrin repeat protein
rowbo03393	PROKKA_03425 hypothetical protein
rowbo03396	PROKKA_03428 hypothetical protein
rowbo03399	PROKKA_03431 hypothetical protein
rowbo03403	PROKKA_03435 potassium transport protein Kup
rowbo03404	PROKKA_03436 potassium transport protein Kup
rowbo03408	PROKKA_03440 putative DNA repair protein YkoV
rowbo03412	PROKKA_03444 ATP-dependent zinc metalloprotease FtsH 4
rowbo03416	PROKKA_03448 hypothetical protein
rowbo03418	PROKKA_03450 hypothetical protein
rowbo03425	PROKKA_03457 CheB methylesterase
rowbo03429	PROKKA_03461 Transcriptional regulatory protein TdiR
rowbo03430	PROKKA_03462 hypothetical protein
rowbo03431	PROKKA_03463 hypothetical protein
rowbo03444	PROKKA_03477 hypothetical protein
rowbo03445	PROKKA_03478 Cation efflux family protein
rowbo03446	PROKKA_03479 hypothetical protein

rowbo03451	PROKKA_03484 hypothetical protein
rowbo03454	PROKKA_03487 hypothetical protein
rowbo03456	PROKKA_03489 hypothetical protein
rowbo03458	PROKKA_03491 ankyrin repeat protein
rowbo03459	PROKKA_03492 hypothetical protein
rowbo03462	PROKKA_03495 hypothetical protein
rowbo03463	PROKKA_03496 hypothetical protein
rowbo03465	PROKKA_03498 hypothetical protein
rowbo03467	PROKKA_03500 hypothetical protein
rowbo03468	PROKKA_03501 DNA repair protein RadC
rowbo03470	PROKKA_03503 hypothetical protein
rowbo03471	PROKKA_03504 hypothetical protein
rowbo03472	PROKKA_03505 hypothetical protein
rowbo03473	PROKKA_03506 hypothetical protein
rowbo03474	PROKKA_03507 hypothetical protein
rowbo03475	PROKKA_03508 hypothetical protein
rowbo03476	PROKKA_03509 hypothetical protein
rowbo03477	PROKKA_03510 hypothetical protein
rowbo03478	PROKKA_03511 putative P-loop ATPase
rowbo03481	PROKKA_03514 DNA-invertase hin
rowbo03482	PROKKA_03515 DNA-invertase hin
rowbo03488	PROKKA_03521 Hsp20/alpha crystallin family protein
rowbo03496	PROKKA_03529 hypothetical protein
rowbo03499	PROKKA_03532 hypothetical protein
rowbo03500	PROKKA_03533 hypothetical protein
rowbo03501	PROKKA_03534 hypothetical protein
rowbo03502	PROKKA_03535 hypothetical protein
rowbo03506	PROKKA_03539 hypothetical protein
rowbo03518	PROKKA_03551 N-glycosyltransferase
rowbo03519	PROKKA_03552 hypothetical protein

rowbo03522	PROKKA_03555 Cation efflux system protein CzcA
rowbo03523	PROKKA_03556 Transposase, TnpA family
rowbo03537	PROKKA_03571 Erythromycin esterase
rowbo03540	PROKKA_03574 hypothetical protein
rowbo03546	PROKKA_03580 hypothetical protein
rowbo03548	PROKKA_03582 hypothetical protein
rowbo03549	PROKKA_03583 hypothetical protein
rowbo03559	PROKKA_03597 hypothetical protein

Supplementary Table 4. Details of the core genes predicted to be under positive selection. (**a**) Core gene clusters predicted to be under positive selection among the clade 1 (*L. pneumophila*) strains compared to the rest of the *Legionella* clades. (**b**) Results of the GO term enrichment analysis of the core genes predicted to be under positive selection in clade 1 (*L. pneumophila*) compared to other *Legionella* clades. Performed hypergeometric probability distribution Test. (**c**) Core gene clusters predicted to be under positive selection among the clade 2 strains compared to the rest of *Legionella* clades. (**d**) Results of the GO term enrichment analysis of the core genes predicted to be under positive selection in clade 2 compared to other *Legionella* clades. Performed hypergeometric probability distribution Test. (**e**) Core gene clusters predicted to be under positive selection among the clade 3 strains compared to the rest of *Legionella* clades. (**f**) Results of the GO term enrichment analysis of the core genes predicted to be under positive selection in clade 3 compared to other *Legionella* clades. Performed hypergeometric probability distribution Test.

4a: Core gene clusters predicted to be under positive selection among the clade 1 strains compared to the rest of *Legionella* clades

**Cluster No.**	**Cluster representative internal Gene id**	**GI Accession Id**	**LOCUS Accession id**	**Gene Annotation**	**LnL H0***	**LnL H1***	**LnLH1-LnLH0**	**2*(LnLH1-LnLH0)**	**Chi p-values**	**q-value**	**sig if a[illegible]**
1149	Anisa02408	654929847	WP_028380217.1	6-phosphofructokinase [Legionella cherrii]	-12673.16338	-12630.61306	42.550322	85.100644	2.83558E-20	2.74E-17	[illegible]
940	Anisa01530	498339205	WP_010653361.1	recombinase RecA [Fluoribacter dumoffii]	-8844.786117	-8808.181304	36.604813	73.209626	1.16586E-17	5.63E-15	[illegible]
1330	Anisa03590	654932071	WP_028382433.1	tRNA uridine 5-carboxymethylaminomethyl modification protein [Legionella cherrii]	-19816.65959	-19792.1107	24.548893	49.097786	2.43515E-12	7.84E-10	[illegible]

699	Anisa01092	654930590	WP_028380959.1	cell division protein FtsA [Legionella cherrii]	-10516.53308	-10493.73524	22.79784	45.59568	1.45362E-11	3.51E-09	[illegible]
1358	Anisa03717	671589527	WP_031565333	histidinol-phosphate aminotransferase [Legionella wadsworthii]	-15045.6055	-15032.23023	13.375273	26.750546	2.31486E-07	3.73E-05	[illegible]
397	Anisa00388	654929751	WP_028380121.1	Lipopolysaccharide export system protein LptA precursor	-6174.045982	-6161.484488	12.561494	25.122988	5.37878E-07	7.42E-05	[illegible]
655	Anisa01031	671587466	WP_031563691.1	Outer membrane protein assembly factor BamA precursor	-23298.39135	-23290.10644	8.284909	16.569818	4.68914E-05	0.00503301	[illegible]
681	Anisa01071	654930612	WP_028380981.1	4-amino-4-deoxychorismate lyase [Legionella cherrii]	-7568.832764	-7561.244574	7.58819	15.17638	9.79206E-05	0.009093046	[illegible]
1197	Anisa03075	518063563	WP_019233771	oligopeptide transporter, OPT family	-22154.09931	-22146.56383	7.53548	15.07096	0.000103544	0.009093046	[illegible]
989	Anisa01646	654931643	WP_028382006.1	glycyl-tRNA synthetase subunit beta [Legionella cherrii]	-19517.28243	-19509.89527	7.387161	14.774322	0.000121174	0.009754507	[illegible]
1033	Anisa01738	654931783	WP_028382146.1	ABC transporter ATPase [Legionella cherrii]	-15100.1486	-15093.39219	6.756403	13.512806	0.000236941	0.01760654	[illegible]

*LnLH0 - estimate of the log likelihood of the null hypothesis; LnLH1 - estimate of the log likelihood of the alternate hypothesis

4b: Results of the GO term enrichment analysis of the core genes predicted to be under positive selection in Clade 1 compared to other Legionella clades. Performed hypergeometric probability distribution Test

**GOID**	**Ontology**	**Term**	**Level**	**q**	**m**	**t**	**k**	log_odds_ratio	p
GO:0008150	biological_process	biological_process	0	10	5467	12977	10	1.247135622	0.012185317
GO:0009987	biological_process	cellular process	1	8	2986	12977	10	1.797742737	0.012185317
GO:0005575	cellular_component	cellular_component	0	6	2157	12977	10	1.851893226	0.02325305
GO:0005622	cellular_component	intracellular	3	4	931	12977	10	2.479103829	0.027504649
GO:0005623	cellular_component	cell	1	6	1249	12977	10	2.640145926	0.012185317
GO:0005737	cellular_component	cytoplasm	5	4	572	12977	10	3.18186985	0.016876011
GO:0044424	cellular_component	intracellular part	4	4	680	12977	10	2.932350251	0.022493545
GO:0044464	cellular_component	cell part	2	6	1200	12977	10	2.697884997	0.012185317
GO:0005488	molecular_function	binding	1	6	2476	12977	10	1.652908088	0.031861224
GO:0043167	molecular_function	ion binding	2	5	1450	12977	10	2.161832097	0.02325305
GO:0044699	biological_process	single-organism process	1	5	1366	12977	10	2.247927513	0.02325305
GO:0043168	molecular_function	anion binding	3	4	1011	12977	10	2.360173905	0.031861224

GO:0003697	molecular_function	single-stranded DNA binding	6	1	11	12977	10	6.882309568	0.044046081
GO:0006399	biological_process	tRNA metabolic process	8	2	86	12977	10	4.915476432	0.02325305
GO:0034660	biological_process	ncRNA metabolic process	7	2	108	12977	10	4.586853685	0.02325305
GO:0002097	biological_process	tRNA wobble base modification	11	1	5	12977	10	8.019813092	0.027504649
GO:0033036	biological_process	macromolecule localization	2	2	149	12977	10	4.122572666	0.03197036
GO:1901264	biological_process	carbohydrate derivative transport	5	1	9	12977	10	7.171816185	0.036942275
GO:0051205	biological_process	protein insertion into membrane	6	1	6	12977	10	7.756778686	0.030630496
GO:0051668	biological_process	localization within membrane	4	1	6	12977	10	7.756778686	0.030630496
GO:0043163	biological_process	cell envelope organization	4	1	7	12977	10	7.534386265	0.031861224

GO:0043165	biological_process	Gram-negative-bacterium-type cell outer membrane assembly	6	1	7	12977	10	7.534386265	0.031861224
GO:0044091	biological_process	membrane biogenesis	3	1	8	12977	10	7.341741187	0.033670189
GO:0071709	biological_process	membrane assembly	5	1	8	12977	10	7.341741187	0.033670189

4c: Core gene clusters predicted to be under positive selection among the Clade 2 strains compared to the rest of Legionella Clades

Cluster number	Cluster representative internal Gene id	GI Accession Id	LOCUS Accession id	Gene Annotation	LnL H0*	LnL H1*	LnL H1-LnL H0	2*(LnLH1-LnL H0)	Chi p-values	p-value	q-value	sig ica[illegible]
202	Anisa00073	654930769	WP_028381137.1	DNA-binding protein [Legionella cherrii]	-10914.0314	-10910.00387	4.027526	8.055052	0.004537694	0.004537694	0.01959459	[illegible]
205	Anisa00077	654930773	WP_028381141.1	16S rRNA methyltransferase [Legionella cherrii]	-14594.65761	-14589.17103	5.486581	10.973162	0.000924409	0.000924409	0.006465192	[illegible]
206	Anisa00080	654930776	WP_028381144.1	membrane protein [Legionella cherrii]	-10029.38897	-10026.51375	2.875221	5.750442	0.016484509	0.0164 8451	0.04868482	[illegible]

208	Anisa00082	654930778	WP_028381146.1	dihydrodipicolinate synthase [Legionella cherrii]	-8858.148276	-8855.209868	2.938408	5.876816	0.01534154	0.01534154	0.04693899	[illegible]
210	Anisa00089	654930785	WP_028381153.1	molecular chaperone GroEL [Legionella cherrii]	-11073.9848	-11070.82784	3.156955	6.31391	0.011979437	0.01197944	0.03924299	[illegible]
213	Anisa00097	654930788	WP_028381156.1	ribosomal protein S12 methylthiotransferase [Legionella cherrii]	-12752.73199	-12743.64263	9.089363	18.178726	2.01113E-05	0.0000201	0.000402226	[illegible]
215	Anisa00102	654930792	WP_028381160.1	ribulose-phosphate 3-epimerase [Legionella cherrii]	-7498.50524	-7483.082812	15.422428	30.844856	2.79502E-08	0.000000028	0.0000029	[illegible]
218	Anisa00111	654930800	WP_028381168.1	glycerophosphodiester phosphodiesterase [Legionella cherrii]	-7686.486634	-7683.025501	3.461133	6.922266	0.008512904	0.008512904	0.03052072	[illegible]
219	Anisa00112	671587614	WP_031563799.1	hypothetical protein [Legionella wadsworthii]	-7530.626062	-7524.628929	5.997133	11.994266	0.000533645	0.000533645	0.004614523	[illegible]
220	Anisa00113	671587613	WP_031563798.1	amino acid dehydrogenase [Legionella wadsworthii]	-10774.54868	-10770.69632	3.852366	7.704732	0.005507625	0.005507625	0.02189242	[illegible]
221	Anisa00114	654930803	WP_028381171.1	SAM-dependent methyltransferase	-7104.59756	-7100.743746	3.853814	7.707628	0.005498796	0.005498796	0.02189242	[illegible]

				[Legionella cherrii]								
223	Anisa00116	654930806	WP_028381174.1	2-keto-4-pentenoate hydratase [Legionella cherrii]	-10790.18643	-10785.55747	4.628965	9.25793	0.002344779	0.002344779	0.01229905	[illegible]
224	Anisa00117	654930807	WP_028381175.1	maleylacetoacetate isomerase [Legionella cherrii]	-6941.214772	-6937.132869	4.081903	8.163806	0.00427346	0.00427346	0.0189562	[illegible]
230	Anisa00129	654930817	WP_028381185.1	pseudouridine synthase [Legionella cherrii]	-9480.666077	-9451.272467	29.39361	58.78722	1.75679E-14	1.76E-14	1E-11	[illegible]
231	Anisa00131	671587589	WP_031563779.1	hypothetical protein, partial [Legionella wadsworthii]	-15835.20359	-15831.28426	3.919337	7.838674	0.005114011	0.005114011	0.0210153	[illegible]
233	Anisa00134	654930821	WP_028381189.1	ankyrin [Legionella cherrii]	-15201.45603	-15198.23957	3.216455	6.43291	0.011202489	0.0112020249	0.03690994	[illegible]
236	Anisa00137	671587581	WP_031563773.1	chorismate synthase [Legionella wadsworthii]	-5753.671447	-5750.388916	3.282531	6.565062	0.010400011	0.0104000001	0.03497349	[illegible]
237	Anisa00138	498338568	WP_010652724.1	N5-glutamine S-adenosyl-L-methionine-dependent methyltransferase [Fluoribacter dumoffii]	-9904.270873	-9898.437661	5.833212	11.666424	0.000636382	0.000636382	0.005108982	[illegible]

239	Anisa00140	498338566	WP_010652722.1	sulfurtransferase [Fluoribacter dumoffii]	-4735.792166	-4731.91256	3.879606	7.759212	0.005343927	0.005343927	0.02167999	[illegible]
240	Anisa00141	654930828	WP_028381196.1	glutaredoxin [Legionella cherrii]	-2542.828128	-2539.144114	3.684014	7.368028	0.00663937	0.006639371	0.02539894	[illegible]
244	Anisa00151	671587567	WP_031563762.1	DNA repair protein RadA [Legionella wadsworthii]	-13178.62807	-13174.76271	3.865356	7.730712	0.005428934	0.005428934	0.0218692	[illegible]
246	Anisa00153	498338554	WP_010652710.1	geranyltranstransferase [Fluoribacter dumoffii]	-11227.01226	-11214.85736	12.154908	24.309816	8.202E-07	0.00000082	0.0000346	[illegible]
248	Anisa00155	502743836	WP_012978820.1	competence protein ComF [Legionella longbeachae]	-7930.681105	-7926.48064	4.200465	8.40093	0.003750291	0.003750291	0.01730904	[illegible]
249	Anisa00157	671586084	WP_031562600.1	peptidase [Legionella wadsworthii]	-10790.51939	-10782.53113	7.988256	15.976512	6.41333E-05	0.0000641	0.000937333	[illegible]
253	Anisa00161	654930847	WP_028381215.1	SAM-dependent methyltransferase [Legionella cherrii]	-9137.090577	-9130.217316	6.873261	13.746522	0.000209208	0.000209208	0.002271401	[illegible]
255	Anisa00163	654930849	WP_028381217.1	3-deoxy-D-manno-octulosonic acid transferase [Legionella cherrii]	-15111.2856	-15103.48023	7.805367	15.610734	7.78116E-05	0.0000778	0.001090258	[illegible]
256	Anisa00164	671586100	WP_031562614.1	molecular chaperone DnaJ	-7284.822734	-7281.637407	3.185327	6.370654	0.011602283	0.0116022 28	0.03811701	[illegible]

				[Legionella wadsworthii]								
258	Anisa00173	654930854	WP_028381222.1	RNA helicase [Legionella cherrii]	-15002.15676	-14998.5035	3.653262	7.306524	0.00687047	0.00687047	0.02602534	[illegible]
259	Anisa00174	654930855	WP_028381223.1	2,4-dienoyl-CoA reductase [Legionella cherrii]	-21034.65308	-21020.50983	14.143253	28.286506	1.04623E-07	0.000000105	0.00000795	[illegible]
260	Anisa00177	654930859	WP_028381227.1	malate dehydrogenase [Legionella cherrii]	-9409.196746	-9406.136448	3.060298	6.120596	0.013361574	0.01336158	0.04231166	[illegible]
261	Anisa00178	654930860	WP_028381228.1	radical SAM protein [Legionella cherrii]	-13617.28102	-13608.43422	8.846807	17.693614	2.59496E-05	0.0000259	0.000477645	[illegible]
263	Anisa00180	654930863	WP_028381231.1	tRNA threonylcarbamoyladenosine modification protein TsaD [Legionella cherrii]	-11250.65779	-11242.87997	7.777819	15.555638	8.01124E-05	0.0000801	0.001100339	[illegible]
267	Anisa00184	518179724	WP_019349932.1	RNA polymerase sigma factor RpoD [Fluoribacter dumoffii]	-16099.82883	-16096.91206	2.916771	5.833542	0.015723435	0.0157234	0.04751194	[illegible]
269	Anisa00208	654930141	WP_028380511.1	DNA mismatch repair protein [Legionella cherrii]	-8652.179359	-8649.077154	3.102205	6.20441	0.012743242	0.0127434	0.04072261	[illegible]

271	Anisa00210	654930139	WP_028380509.1	aspartyl-tRNA synthetase [Legionella cherrii]	-18517.24918	-18511.82971	5.419467	10.838934	0.000993879	0.000993879	0.006744179	[illegible]
273	Anisa00213	671589997	WP_031565728.1	membrane protein [Legionella wadsworthii]	-9806.351791	-9800.502999	5.848792	11.697584	0.000625813	0.000625813	0.005059765	[illegible]
274	Anisa00218	654930131	WP_028380501.1	oxidoreductase [Legionella cherrii]	-9555.713958	-9552.611378	3.10258	6.20516	0.012737843	0.01273784	0.04072261	[illegible]
276	Anisa00224	654930124	WP_028380494.1	integrase [Legionella cherrii]	-20581.59681	-20572.21458	9.382235	18.76447	1.47897E-05	0.0000148	0.000324236	[illegible]
279	Anisa00227	654930121	WP_028380491.1	3-demethylubiquinone-9 3-methyltransferase [Legionella cherrii]	-5735.725708	-5731.726829	3.998879	7.997758	0.004683531	0.004683531	0.01977491	[illegible]
280	Anisa00228	657688538	WP_029488989.1	uracil-DNA glycosylase [Fluoribacter dumoffii]	-8829.363584	-8826.03011	3.333474	6.666948	0.009821724	0.009821725	0.0337252	[illegible]
283	Anisa00236	518179477	WP_019349685.1	hypothetical protein [Fluoribacter dumoffii]	-2785.276933	-2780.296516	4.980417	9.960834	0.001599056	0.001599056	0.009517878	[illegible]
286	Anisa00240	654930108	WP_028380478.1	elongation factor Ts [Legionella cherrii]	-7948.192902	-7938.653169	9.539733	19.079466	1.25387E-05	0.0000125	0.000291717	[illegible]
287	Anisa00241	654930107	WP_028380477.1	30S ribosomal protein S2 [Legionella	-5829.024187	-5825.922632	3.101555	6.20311	0.012752604	0.01275261	0.04072261	[illegible]

				cherrii]								
291	Anisa00249	498339633	WP_010653789.1	ATP-dependent dethiobiotin synthetase BioD [Fluoribacter dumoffii]	-8297.3607	-8288.364015	8.996685	17.99337	2.21676E-05	0.0000222	0.000435708	[illegible]
296	Anisa00256	654930093	WP_028380463.1	dephospho-CoA kinase [Legionella cherrii]	-8509.113259	-8497.965357	11.147902	22.295804	2.33717E-06	0.00000234	0.0000727	[illegible]
300	Anisa00262	671589904	WP_031565650.1	ssDNA exonuclease RecJ [Legionella wadsworthii]	-19416.05372	-19407.93806	8.115659	16.231318	5.60597E-05	0.0000561	0.000840896	[illegible]
301	Anisa00263	654930084	WP_028380454.1	hypothetical protein [Legionella cherrii]	-8576.118925	-8572.624778	3.494147	6.988294	0.008204452	0.008204452	0.02988203	[illegible]
302	Anisa00264	654930083	WP_028380453.1	glutamate--pyruvate aminotransferase [Legionella cherrii]	-10661.65591	-10656.22604	5.429876	10.859752	0.000982769	0.000982769	0.006708723	[illegible]
306	Anisa00270	654930078	WP_028380448.1	dihydrolipoamide dehydrogenase [Legionella cherrii]	-12723.50153	-12716.26312	7.238407	14.476814	0.000141895	0.000141895	0.001720854	[illegible]
307	Anisa00271	654930077	WP_028380447.1	dihydrolipoamide acetyltransferase [Legionella cherrii]	-19384.50507	-19365.73014	18.774927	37.549854	8.91061E-10	8.91E-10	0.000000145	[illegible]
308	Anisa00	4983396	WP_010	acyltransferase	-4176.290928	-4169.095949	7.19	14.38	0.00014	0.0001	0.001764	[illegible]

	273	57	653813.1	[Fluoribacter dumoffii]			4979	9958	8593	48593	542	
318	Anisa00285	654930063	WP_028380433.1	segregation and condensation protein A [Legionella cherrii]	-9679.761233	-9672.609311	7.151922	14.303844	0.000155547	0.000155547	0.001828078	[illegible]
327	Anisa00296	498339680	WP_010653836.1	purine nucleoside phosphorylase [Fluoribacter dumoffii]	-8883.66789	-8878.379157	5.288733	10.577466	0.001144745	0.001144745	0.007432502	[illegible]
328	Anisa00297	654930052	WP_028380422.1	deoxyribose-phosphate aldolase [Legionella cherrii]	-11613.78567	-11610.17686	3.608814	7.217628	0.007219104	0.007219104	0.0268071	[illegible]
329	Anisa00298	654930051	WP_028380421.1	hypothetical protein [Legionella cherrii]	-2449.843804	-2446.896798	2.947006	5.894012	0.01519245	0.01519245	0.046683	[illegible]
330	Anisa00301	657688530	WP_029488981.1	4-hydroxybenzoate polyprenyltransferase [Fluoribacter dumoffii]	-8980.565346	-8975.906305	4.659041	9.318082	0.002269034	0.002269034	0.01220141	[illegible]
332	Anisa00306	654930046	WP_028380416.1	orotidine 5'-phosphate decarboxylase [Legionella cherrii]	-9177.871477	-9173.245298	4.626179	9.252358	0.002351924	0.002351924	0.01229905	[illegible]
333	Anisa00307	654930045	WP_028380415.1	aminotransferase DegT [Legionella cherrii]	-9139.401038	-9134.700232	4.700806	9.401612	0.002167948	0.002167948	0.01176886	[illegible]

339	Anisa00314	654930038	WP_028380408.1	DNA gyrase subunit A [Legionella cherrii]	-23624.38479	-23612.57575	11.809038	23.618076	1.17479E-06	0.00000117	0.0000446	[illegible]
341	Anisa00316	654930036	WP_028380406.1	glycerol kinase [Legionella cherrii]	-16705.30094	-16701.38675	3.91419	7.82838	0.005143218	0.005143218	0.0210153	[illegible]
342	Anisa00317	654930035	WP_028380405.1	glycerol-3-phosphate dehydrogenase [Legionella cherrii]	-14184.57641	-14181.64982	2.926589	5.853178	0.015548947	0.01554895	0.04714309	[illegible]
345	Anisa00320	671589785	WP_031565549.1	hypothetical protein [Legionella wadsworthii]	-4648.599078	-4640.864382	7.734696	15.469392	8.38522E-05	0.0000839	0.001137994	[illegible]
350	Anisa00330	654930021	WP_028380391.1	deoxyribonuclease [Legionella cherrii]	-9026.310006	-9022.881125	3.428881	6.857762	0.008825702	0.008825703	0.03089388	[illegible]
352	Anisa00333	654930018	WP_028380388.1	thymidylate kinase [Legionella cherrii]	-5021.575373	-5017.986304	3.589069	7.178138	0.007379726	0.007379726	0.02731457	[illegible]
371	Anisa00354	654929722	WP_028380092.1	L-aspartate oxidase [Legionella cherrii]	-17399.78439	-17394.85283	4.931556	9.863112	0.001686256	0.001686256	0.009815359	[illegible]
374	Anisa00357	654929725	WP_028380095.1	phosphoenolpyruvate synthase [Legionella cherrii]	-21365.91413	-21358.65924	7.254893	14.509786	0.000139433	0.000139433	0.001709179	[illegible]
378	Anisa00361	654929729	WP_028380099.1	peptidase M20 [Legionella	-15868.10414	-15862.80292	5.301219	10.602438	0.001129386	0.001129386	0.007432502	[illegible]

				cherrii]								
379	Anisa00362	671589072	WP_031564960.1	competence protein ComEA [Legionella wadsworthii]	-4371.191759	-4367.931064	3.260695	6.52139	0.010658467	0.01065847	0.03563242	[illegible]
388	Anisa00374	518179396	WP_019349604.1	glycosyl transferase [Fluoribacter dumoffii]	-7520.29645	-7512.748936	7.547514	15.095028	0.000102232	0.000102232	0.001355168	[illegible]
389	Anisa00377	654929741	WP_028380111.1	neurogenic locus notch [Legionella cherrii]	-4234.054497	-4230.79078	3.263717	6.527434	0.010622309	0.01062231	0.03561598	[illegible]
391	Anisa00379	654929743	WP_028380113.1	exodeoxyribonuclease VII large subunit [Legionella cherrii]	-10301.40842	-10289.0884	12.320024	24.640048	6.91007E-07	0.000000691	0.0000313	[illegible]
394	Anisa00385	654929748	WP_028380118.1	anthranilate phosphoribosyltransferase [Legionella cherrii]	-12940.80787	-12935.52133	5.286532	10.573064	0.001147474	0.001147474	0.007432502	[illegible]
397	Anisa00388	654929751	WP_028380121.1	hypothetical protein [Legionella cherrii]	-6155.048193	-6152.096885	2.951308	5.902616	0.015118414	0.01511841	0.04658106	[illegible]
407	Anisa00399	518179402	WP_019349610.1	metal-binding protein [Fluoribacter dumoffii]	-9636.274656	-9633.318006	2.95665	5.9133	0.015026996	0.01502 7	0.04655102	[illegible]
410	Anisa00407	654929768	WP_028380138.1	cytochrome C biogenesis protein CcmA	-8014.596368	-7988.715643	25.880725	51.76145	6.26703E-13	6.27E-13	1.79E-10	[illegible]

				[Legionella cherrii]								
418	Anisa00418	654929779	WP_028380149.1	FMN adenylyltransferase [Legionella cherrii]	-6362.117839	-6359.202774	2.915065	5.83013	0.015753959	0.01575396	0.04751194	[illegible]
419	Anisa00419	654929780	WP_028380150.1	isoleucyl-tRNA synthetase [Legionella cherrii]	-16848.94667	-16844.03017	4.916504	9.833008	0.001714078	0.0017 14078	0.009868934	[illegible]
422	Anisa00425	654929788	WP_028380158.1	coiled-coil protein [Legionella cherrii]	-41001.50149	-40992.94611	8.555381	17.110762	3.52619E-05	0.0000353	0.000591155	[illegible]
428	Anisa00434	498339925	WP_010654081.1	protease TldD [Fluoribacter dumoffii]	-12416.14226	-12412.59431	3.547949	7.095898	0.007726057	0.007726057	0.02832059	[illegible]
429	Anisa00443	654929805	WP_028380175.1	peptide ABC transporter substrate-binding protein [Legionella cherrii]	-20128.25419	-20124.02673	4.227455	8.45491	0.003640593	0.003640593	0.01687104	[illegible]
431	Anisa00446	654929808	WP_028380178.1	DNA polymerase III subunit alpha [Legionella cherrii]	-19878.15849	-19866.27576	11.882731	23.765462	1.08818E-06	0.00000109	0.0000443	[illegible]
432	Anisa00447	654929809	WP_028380179.1	pterin-4-alpha-carbinolamine dehydratase [Legionella cherrii]	-3156.561429	-3150.371673	6.189756	12.379512	0.000434071	0.000434071	0.004023097	[illegible]

434	Anisa00450	654929813	WP_028380183.1	prepilin cleavage protein [Legionella cherrii]	-7651.21455	-7646.357719	4.856831	9.713662	0.001829032	0.001829032	0.01042548	[illegible]
435	Anisa00451	654929814	WP_028380184.1	hypothetical protein [Legionella cherrii]	-4844.539153	-4840.535781	4.003372	8.006744	0.004660346	0.004660346	0.01975017	[illegible]
436	Anisa00452	654929817	WP_028380187.1	membrane protein [Legionella cherrii]	-13562.35434	-13556.75673	5.59761	11.19522	0.000820083	0.000820083	0.006006521	[illegible]
439	Anisa00500	654930303	WP_028380673.1	crotonase [Legionella cherrii]	-15191.42446	-15184.1453	7.279159	14.558318	0.000135887	0.000135887	0.001692776	[illegible]
447	Anisa00514	498339452	WP_010653608.1	glycine cleavage system protein T [Fluoribacter dumoffii]	-14262.50091	-14253.55917	8.941749	17.883498	2.34849E-05	0.0000235	0.000453776	[illegible]
449	Anisa00520	653017810	WP_027269773.1	esterase [Legionella sainthelensi]	-1958.79397	-1955.167054	3.626916	7.253832	0.007074987	0.007074988	0.0263578	[illegible]
451	Anisa00522	654930284	WP_028380654.1	biopolymer transporter TolR [Legionella cherrii]	-3886.625218	-3882.256745	4.368473	8.736946	0.003118263	0.003118264	0.01519154	[illegible]
452	Anisa00524	498339462	WP_010653618.1	translocation protein TolB [Fluoribacter dumoffii]	-12861.99561	-12855.39984	6.595773	13.191546	0.000281215	0.000281215	0.002837036	[illegible]
454	Anisa00530	654930279	WP_028380649.1	branched-chain alpha-keto acid dehydrogenase subunit E2	-12022.7442	-12019.88757	2.856625	5.71325	0.01683734	0.01683734	0.04909097	[illegible]

				[Legionella cherrii]								
456	Anisa00532	654930277	WP_028380647.1	ABC transporter permease [Legionella cherrii]	-8121.507853	-8116.211963	5.29589	10.59178	0.001135916	0.001135916	0.007432502	[illegible]
461	Anisa00539	654930270	WP_028380640.1	tRNA hydroxylase [Legionella cherrii]	-4429.919038	-4425.194737	4.724301	9.448602	0.002113101	0.002113101	0.01158142	[illegible]
481	Anisa00562	654930246	WP_028380616.1	2-methylcitrate dehydratase [Legionella cherrii]	-14594.63587	-14586.0432	8.59267	17.18534	3.39043E-05	0.0000339	0.000576879	[illegible]
483	Anisa00566	654930241	WP_028380611.1	hypothetical protein [Legionella cherrii]	-4080.468096	-4075.741555	4.726541	9.453082	0.002107946	0.002107946	0.01158142	[illegible]
492	Anisa00629	654930177	WP_028380547.1	alpha-2-macroglobulin [Legionella cherrii]	-55617.66615	-55613.29599	4.370162	8.740324	0.003112492	0.003112492	0.01519154	[illegible]
494	Anisa00657	654929505	WP_028379875.1	adenosylhomocysteinase [Legionella cherrii]	-12507.41083	-12503.26978	4.141045	8.28209	0.004003802	0.004003802	0.01833066	[illegible]
496	Anisa00659	654929503	WP_028379873.1	carbamoyl phosphate synthase small subunit [Legionella cherrii]	-11377.72227	-11374.41966	3.30261	6.60522	0.010168024	0.0101 6803	0.03429452	[illegible]

500	Anisa00666	498340275	WP_010654431.1	phospho-2-dehydro-3-deoxyheptonate aldolase [Fluoribacter dumoffii]	-13062.11316	-13057.50677	4.606382	9.212764	0.002403336	0.002403336	0.01245365	[illegible]
501	Anisa00672	654929494	WP_028379864.1	uroporphyrinogen decarboxylase [Legionella cherrii]	-12300.50822	-12293.49731	7.010908	14.021816	0.000180702	0.000180702	0.002080811	[illegible]
504	Anisa00676	654929489	WP_028379859.1	ATP-dependent DNA helicase RecG [Legionella cherrii]	-21844.49826	-21841.07041	3.427846	6.855692	0.008835933	0.008835933	0.03089388	[illegible]
505	Anisa00678	671588499	WP_031564480.1	zinc transporter [Legionella wadsworthii]	-5410.335185	-5406.979229	3.355956	6.711912	0.009577098	0.009577099	0.03308452	[illegible]
509	Anisa00687	654929479	WP_028379849.1	endonuclease [Legionella cherrii]	-35781.91515	-35778.26203	3.653114	7.306228	0.006871602	0.006871602	0.02602534	[illegible]
510	Anisa00689	671588473	WP_031564458.1	membrane protein [Legionella wadsworthii]	-7913.345341	-7904.69072	8.654621	17.309242	3.17638E-05	0.0000318	0.000557088	[illegible]
512	Anisa00691	498340295	WP_010654451.1	tyrosine-specific transporter [Fluoribacter dumoffii]	-9111.690079	-9106.727513	4.962566	9.925132	0.001630372	0.001630372	0.009630177	[illegible]
523	Anisa00742	654929432	WP_028379802.1	phosphopentomutase [Legionella cherrii]	-14114.0556	-14110.91797	3.137634	6.275268	0.01224346	0.01224346	0.0398787	[illegible]
524	Anisa00744	671588368	WP_031564369.1	MFS transporter [Legionella wadsworthii]	-13265.80471	-13258.73365	7.071066	14.142132	0.000169505	0.000169505	0.001971793	[illegible]

525	Anisa00747	654929427	WP_028379797.1	polysaccharide deacetylase [Legionella cherrii]	-4663.523671	-4658.14969	5.373981	10.747962	0.001043942	0.001043942	0.007041976	[illegible]
527	Anisa00774	498339280	WP_010653436.1	protease [Fluoribacter dumoffii]	-13425.70644	-13414.93261	10.773834	21.547668	3.45142E-06	0.00000345	0.000100888	[illegible]
532	Anisa00786	654931517	WP_028381880.1	acetyl-CoA carboxylase subunit beta [Legionella cherrii]	-6707.133312	-6703.142621	3.990691	7.981382	0.004726085	0.004726085	0.01988095	[illegible]
533	Anisa00787	654931516	WP_028381879.1	folylpolyglutamate synthase [Legionella cherrii]	-16978.57186	-16969.28426	9.287609	18.575218	0.000016333	0.0000163	0.000344808	[illegible]
534	Anisa00788	671590558	WP_031566200.1	sporulation protein [Legionella wadsworthii]	-8679.384764	-8672.016696	7.368068	14.736136	0.000123653	0.000123653	0.00158387	[illegible]
540	Anisa00796	671590539	WP_031566184.1	saccharopine dehydrogenase [Legionella wadsworthii]	-9670.094169	-9661.744066	8.350103	16.700206	4.37762E-05	0.0000438	0.000703669	[illegible]
543	Anisa00802	654931502	WP_028381865.1	leucyl/phenylalanyl-tRNA--protein transferase [Legionella cherrii]	-4101.375187	-4097.031425	4.343762	8.687524	0.003203956	0.003203956	0.01547674	[illegible]
550	Anisa00819	654931486	WP_028381849.1	UDP-N-acetylmuramate:L-alanyl-gamma-D-glutamyl-	-14909.52974	-14904.20469	5.32505	10.6501	0.00110065	0.00110065	0.007295006	[illegible]

				meso-diaminopimelate ligase [Legionella cherrii]								
554	Anisa00829	654931476	WP_028381839.1	cysteine desulfurase [Legionella cherrii]	-13074.75891	-13069.21474	5.544177	11.088354	0.000868716	0.000868716	0.006208632	[illegible]
558	Anisa00835	654931470	WP_028381833.1	glutamyl-tRNA amidotransferase [Legionella cherrii]	-12045.30819	-12040.12761	5.180577	10.361154	0.001286947	0.001286947	0.008150664	[illegible]
559	Anisa00836	654931469	WP_028381832.1	glutamyl-tRNA amidotransferase [Legionella cherrii]	-17029.96765	-17020.65265	9.315006	18.630012	1.58702E-05	0.0000159	0.000341359	[illegible]
569	Anisa00851	654931451	WP_028381814.1	hydrolase [Legionella cherrii]	-9016.892118	-9013.250694	3.641424	7.282848	0.006961608	0.006961608	0.02610603	[illegible]
573	Anisa00856	498339362	WP_010653518.1	inosine-5-monophosphate dehydrogenase [Fluoribacter dumoffii]	-13514.09961	-13509.22868	4.870932	9.741864	0.001801182	0.001801182	0.01031833	[illegible]
580	Anisa00893	654931099	WP_028381466.1	ATP F0F1 synthase subunit alpha [Legionella cherrii]	-12941.57161	-12938.14517	3.42644	6.85288	0.00884985	0.008849851	0.03089388	[illegible]
581	Anisa00894	498341133	WP_010655289.1	F0F1 ATP synthase subunit delta [Fluoribacter dumoffii]	-5231.829465	-5227.913759	3.915706	7.831412	0.005134597	0.005134598	0.0210153	[illegible]

586	Anisa00901	654931092	WP_028381459.1	hemolysin [Legionella cherrii]	-6460.390998	-6455.538784	4.852214	9.704428	0.001838245	0.001838245	0.01042587	[illegible]
587	Anisa00902	671591227	WP_031566748.1	hypothetical protein [Legionella wadsworthii]	-5955.488008	-5952.446787	3.041221	6.082442	0.013653191	0.01365319	0.04299624	[illegible]
590	Anisa00905	654931088	WP_028381455.1	hypothetical protein [Legionella cherrii]	-22207.8149	-22203.43262	4.382277	8.764554	0.003071412	0.003071412	0.01509228	[illegible]
591	Anisa00906	654931087	WP_028381454.1	tetrapyrrole methylase [Legionella cherrii]	-10954.27736	-10944.31925	9.958118	19.916236	8.09103E-06	0.00000809	0.000192162	[illegible]
592	Anisa00907	671591237	WP_031566756.1	membrane protein, partial [Legionella wadsworthii]	-14945.61968	-14941.82408	3.795592	7.591184	0.005865442	0.005865442	0.02297802	[illegible]
594	Anisa00909	518180157	WP_019350365.1	insertase [Fluoribacter dumoffii]	-8665.043449	-8651.783956	13.259493	26.518986	2.60961E-07	0.000000261	0.0000149	[illegible]
596	Anisa00914	654931068	WP_028381435.1	DNA polymerase III subunit beta [Legionella cherrii]	-12712.7879	-12706.86146	5.926442	11.852884	0.000575721	0.000575721	0.004830733	[illegible]
601	Anisa00921	654931062	WP_028381429.1	hydrolase [Legionella cherrii]	-8602.889357	-8598.96438	3.924977	7.849954	0.0050822	0.0050822	0.0209917	[illegible]
603	Anisa00924	654931058	WP_028381425.1	GTPase HflX [Legionella cherrii]	-12961.95159	-12958.71589	3.235698	6.471396	0.010962439	0.01096244	0.03643493	[illegible]

604	Anisa00925	671591284	WP_031566795.1	thiol-disulfide oxidoreductase [Legionella wadsworthii]	-5665.30809	-5661.02449	4.2836	8.5672	0.003422731	0.003422731	0.01625797	[illegible]
605	Anisa00929	654931053	WP_028381420.1	quercetin 2,3-dioxygenase [Legionella cherrii]	-4122.14231	-4118.434999	3.707311	7.414622	0.006469591	0.006469591	0.02491667	[illegible]
606	Anisa00937	654931048	WP_028381415.1	membrane protein [Legionella cherrii]	-6800.732592	-6796.846762	3.88583	7.77166	0.005307225	0.005307226	0.02160799	[illegible]
608	Anisa00939	654931046	WP_028381413.1	23S rRNA methyltransferase [Legionella cherrii]	-23862.13528	-23853.5266	8.608676	17.217352	3.33377E-05	0.0000333	0.000575833	[illegible]
610	Anisa00943	671591312	WP_031566819.1	amino acid permease [Legionella wadsworthii]	-10750.99931	-10745.3869	5.612407	11.224814	0.000807109	0.000807109	0.006006521	[illegible]
618	Anisa00954	498338008	WP_010652164.1	dihydroorotase [Fluoribacter dumoffii]	-14556.07365	-14552.50421	3.569442	7.138884	0.007543009	0.007543009	0.02773881	[illegible]
620	Anisa00960	654932016	WP_028382378.1	membrane protein [Legionella cherrii]	-11776.35134	-11762.99094	13.360399	26.720798	2.35078E-07	0.000000235	0.0000141	[illegible]
624	Anisa00966	654932022	WP_028382384.1	hypothetical protein [Legionella cherrii]	-7739.113994	-7722.291968	16.822026	33.644052	6.61769E-09	6.62E-09	0.000000838	[illegible]
626	Anisa00969	493924554	WP_006869417.1	cystathionine beta-lyase [Legionella drancourtii]	-9787.783656	-9780.223518	7.560138	15.120276	0.000100874	0.000100874	0.001352898	[illegible]

627	Anisa00972	654932027	WP_028382389.1	fumarate hydratase [Legionella cherrii]	-13906.35077	-13900.7154	5.635372	11.270744	0.000787382	0.000787382	0.005984103	[illegible]
631	Anisa00976	498338056	WP_010652212.1	3-polyprenyl-4-hydroxybenzoate decarboxylase [Fluoribacter dumoffii]	-13862.70925	-13859.46979	3.239467	6.478934	0.010916043	0.01091604	0.03638681	[illegible]
632	Anisa00977	654932031	WP_028382393.1	flavin oxidoreductase [Legionella cherrii]	-8385.573006	-8377.429095	8.143911	16.287822	5.44125E-05	0.0000544	0.00082707	[illegible]
635	Anisa00982	671591399	WP_031566891.1	preprotein translocase subunit TatC [Legionella wadsworthii]	-8732.754324	-8729.651969	3.102355	6.20471	0.012741082	0.01274108	0.04072261	[illegible]
636	Anisa00984	654932039	WP_028382401.1	dimethyladenosine transferase [Legionella cherrii]	-7535.817193	-7528.929119	6.888074	13.776148	0.000205934	0.000205934	0.002257353	[illegible]
637	Anisa00985	671591406	WP_031566897.1	helicase [Legionella wadsworthii]	-15509.98144	-15503.36011	6.621339	13.242678	0.000273647	0.000273647	0.002835978	[illegible]
638	Anisa00986	654932041	WP_028382403.1	diadenosine tetraphosphatase [Legionella cherrii]	-9489.047168	-9477.216577	11.830591	23.661182	1.14876E-06	0.00000115	0.0000446	[illegible]
643	Anisa00995	654932047	WP_028382409.1	methionine sulfoxide reductase [Legionella	-9765.338236	-9759.409374	5.928862	11.857724	0.000574227	0.000574227	0.004830733	[illegible]

				cherrii]								
649	Anisa01020	654930661	WP_028381030.1	hypothetical protein [Legionella cherrii]	-11181.89106	-11178.77728	3.113783	6.227566	0.012577651	0.01257765	0.04072261	[illegible]
650	Anisa01026	654930658	WP_028381027.1	seryl-tRNA synthetase [Legionella cherrii]	-13223.37854	-13219.04062	4.337929	8.675858	0.00322453	0.00322453	0.0155104	[illegible]
653	Anisa01029	654930655	WP_028381024.1	UDP-3-O-(3-hydroxymyristoyl) glucosamine N-acyltransferase [Legionella cherrii]	-12824.66672	-12820.03014	4.636582	9.273164	0.002325357	0.0023253557	0.01229905	[illegible]
655	Anisa01031	671587466	WP_031563691.1	membrane protein [Legionella wadsworthii]	-23273.06064	-23260.66558	12.395061	24.790122	6.39242E-07	0.000000639	0.0000304	[illegible]
659	Anisa01036	654930648	WP_028381017.1	peptidase M24 [Legionella cherrii]	-4643.541741	-4640.142221	3.39952	6.79904	0.00912069	0.009120691	0.03169996	[illegible]
661	Anisa01040	498340472	WP_010654628.1	adenylosuccinate synthetase [Fluoribacter dumoffii]	-12789.86845	-12783.6394	6.229052	12.458104	0.000416182	0.000416182	0.003921054	[illegible]
664	Anisa01045	654930638	WP_028381007.1	ankyrin [Legionella cherrii]	-14324.63574	-14321.79279	2.842947	5.685894	0.017101824	0.01710183	0.0497349	[illegible]
675	Anisa01058	654930625	WP_028380994.1	lipase [Legionella cherrii]	-10776.8343	-10772.0918	4.742498	9.484996	0.002071591	0.002071591	0.01156444	[illegible]
676	Anisa01059	654930624	WP_028380993.1	zinc protease [Legionella	-17502.69594	-17496.55582	6.140116	12.280232	0.000457781	0.000457781	0.004078323	[illegible]

				cherrii]								
681	Anisa01071	654930612	WP_028380981.1	4-amino-4-deoxychorismate lyase [Legionella cherrii]	-7553.258819	-7547.363114	5.895705	11.79141	0.000595046	0.000595047	0.004889698	[illegible]
683	Anisa01073	654930610	WP_028380979.1	5-formyltetrahydrofolate cyclo-ligase [Legionella cherrii]	-6762.975531	-6756.95182	6.023711	12.047422	0.000518641	0.000518641	0.004548083	[illegible]
687	Anisa01078	498340510	WP_010654666.1	aspartate carbamoyltransferase [Fluoribacter dumoffii]	-10939.69902	-10934.96005	4.738971	9.477942	0.002079571	0.002079571	0.01156444	[illegible]
696	Anisa01089	654930593	WP_028380962.1	metallopeptidase [Legionella cherrii]	-19328.99466	-19320.20186	8.792806	17.585612	2.74659E-05	0.0000275	0.000494735	[illegible]
703	Anisa01096	671587313	WP_031563574.1	UDP-N-acetylmuramate--alanine ligase [Legionella wadsworthii]	-12910.71792	-12907.84125	2.876675	5.75335	0.016457245	0.01645725	0.04868482	[illegible]
705	Anisa01098	654930584	WP_028380953.1	UDP-N-acetylmuramoyl-L-alanyl-D-glutamate synthetase [Legionella cherrii]	-14394.15302	-14380.9645	13.188518	26.377036	2.8086E-07	0.000000281	0.0000152	[illegible]
706	Anisa01099	654930583	WP_028380952.1	phospho-N-acetylmuramoyl-pentapeptide transferase	-9483.213741	-9478.461966	4.751775	9.50355	0.002050748	0.002050748	0.01151652	[illegible]

				[Legionella cherrii]								
707	Anisa01100	653017650	WP_027269614.1	UDP-N-acetylmuramoyl-tripeptide--D-alanyl-D-alanine ligase [Legionella sainthelensi]	-15355.16956	-15345.78469	9.384873	18.769746	1.47488E-05	0.0000147	0.000324236	[illegible]
709	Anisa01104	654930579	WP_028380948.1	acid phosphatase [Legionella cherrii]	-8776.671785	-8771.677804	4.993981	9.987962	0.001575669	0.001575669	0.009454014	[illegible]
715	Anisa01119	654931152	WP_028381519.1	hypothetical protein [Legionella cherrii]	-6531.500303	-6525.989034	5.511269	11.022538	0.000900107	0.000900107	0.006334086	[illegible]
716	Anisa01120	654931153	WP_028381520.1	proline aminopeptidase P II [Legionella cherrii]	-9631.685106	-9626.608095	5.077011	10.154022	0.001439868	0.001439868	0.008903215	[illegible]
717	Anisa01121	654931154	WP_028381521.1	2-octaprenyl-6-methoxyphenyl hydroxylase [Legionella cherrii]	-14293.49789	-14279.67126	13.826628	27.653256	1.45128E-07	0.000000145	0.00000973	[illegible]
718	Anisa01122	654931155	WP_028381522.1	2-polyprenyl-6-methoxyphenol hydroxylase [Legionella cherrii]	-13324.34823	-13321.20137	3.146862	6.293724	0.012116621	0.01211662	0.03957865	[illegible]
721	Anisa01129	498341071	WP_010655227.1	hypothetical protein [Fluoribacter dumoffii]	-2275.802314	-2271.074984	4.72733	9.45466	0.002106133	0.002106133	0.01158142	[illegible]

727	Anisa01147	654931176	WP_028381543.1	DNA polymerase I [Legionella cherrii]	-26837.21647	-26810.24634	26.970135	53.94027	2.06678E-13	2.07E-13	7.85E-11	[illegible]
729	Anisa01151	654931180	WP_028381547.1	3-oxoacyl-ACP synthase [Legionella cherrii]	-12751.78289	-12748.94534	2.837542	5.675084	0.017207516	0.01720752	0.04991493	[illegible]
733	Anisa01161	654931190	WP_028381557.1	3-deoxy-manno-octulosonate cytidylyltransferase [Legionella cherrii]	-9414.574583	-9398.807215	15.767368	31.534736	1.95904E-08	1.96E-08	0.00000223	[illegible]
735	Anisa01173	654931201	WP_028381568.1	glycine dehydrogenase [Legionella cherrii]	-14821.5261	-14817.88323	3.642867	7.285734	0.006950433	0.006950433	0.02610603	[illegible]
737	Anisa01175	654931203	WP_028381570.1	glycine cleavage system protein T [Legionella cherrii]	-11042.99422	-11038.60128	4.392935	8.78587	0.003035727	0.003035728	0.01504665	[illegible]
739	Anisa01177	489730068	WP_003634184.1	type IV secretion system protein IcmL [Legionella longbeachae]	-6472.175624	-6468.453546	3.722078	7.444156	0.006364276	0.006364276	0.02459415	[illegible]
743	Anisa01183	653018590	WP_027270548.1	cytochrome C [Legionella sainthelensi]	-6704.065163	-6699.878762	4.186401	8.372802	0.003808779	0.003808779	0.0175081	[illegible]
744	Anisa01184	671590990	WP_031566552.1	GTP-binding protein [Legionella wadsworthii]	-3668.222466	-3663.963017	4.259449	8.518898	0.003514773	0.003514773	0.0166259	[illegible]
745	Anisa01194	654931220	WP_028381587.1	acetyl-CoA synthetase	-21883.09975	-21880.08738	3.012366	6.024732	0.014106774	0.01410678	0.04418056	[illegible]

				[Legionella cherrii]								
746	Anisa01196	671590965	WP_031566531.1	methylmalonate-semialdehyde dehydrogenase [Legionella wadsworthii]	-16278.25366	-16272.13073	6.122935	12.24587	0.000466289	0.000466289	0.004120693	[illegible]
747	Anisa01199	654931224	WP_028381591.1	dihydrodipicolinate reductase [Legionella cherrii]	-9062.039935	-9054.193829	7.846106	15.692212	7.45304E-05	0.0000745	0.001062058	[illegible]
751	Anisa01206	654931230	WP_028381597.1	transketolase [Legionella cherrii]	-21731.18744	-21727.23968	3.947754	7.895508	0.004955772	0.004955772	0.02054393	[illegible]
752	Anisa01208	654931232	WP_028381599.1	oligopeptidase A [Legionella cherrii]	-24302.51015	-24296.52122	5.988934	11.977868	0.000538361	0.000538361	0.004614523	[illegible]
753	Anisa01217	654930546	WP_028380915.1	ferrochelatase [Legionella cherrii]	-13378.96426	-13375.33209	3.632162	7.264324	0.007033774	0.007033774	0.02629017	[illegible]
758	Anisa01234	654930527	WP_028380896.1	cytochrome B561 [Legionella cherrii]	-11305.38059	-11298.62443	6.756161	13.512322	0.000237002	0.000237002	0.002525068	[illegible]
765	Anisa01241	654930940	WP_028381308.1	transporter [Legionella cherrii]	-12465.59955	-12461.27126	4.328295	8.65659	0.003258806	0.003258806	0.01560941	[illegible]
768	Anisa01244	654930943	WP_028381311.1	16S rRNA processing protein RimM [Legionella cherrii]	-5409.960825	-5406.516632	3.444193	6.888386	0.008675757	0.008675757	0.03071541	[illegible]
771	Anisa01250	498340604	WP_010654760.1	hypothetical protein	-2984.308497	-2979.7795	4.528997	9.057994	0.002615488	0.002615488	0.01325181	[illegible]

				[Fluoribacter dumoffii]								
772	Anisa01251	654930954	WP_028381322.1	zinc metalloprotease [Legionella cherrii]	-8340.14783	-8335.564306	4.583524	9.167048	0.002464117	0.002464117	0.01265357	[illegible]
773	Anisa01252	654930955	WP_028381323.1	hypothetical protein [Legionella cherrii]	-7761.665128	-7757.081281	4.583847	9.167694	0.002463248	0.002463248	0.01265357	[illegible]
784	Anisa01287	654930990	WP_028381358.1	carboxylesterase [Legionella cherrii]	-7254.995603	-7249.882257	5.113346	10.226692	0.001384227	0.001384227	0.008623053	[illegible]
785	Anisa01289	654930992	WP_028381360.1	diaminopimelate epimerase [Legionella cherrii]	-6983.58444	-6977.4446	6.13984	12.27968	0.000457917	0.000457917	0.004078323	[illegible]
788	Anisa01292	654930995	WP_028381363.1	3-oxoacyl-ACP synthase [Legionella cherrii]	-14266.46767	-14261.39382	5.073846	10.147692	0.00144482	0.00144482	0.008903215	[illegible]
802	Anisa01313	498340661	WP_010654817.1	30S ribosomal protein S5 [Fluoribacter dumoffii]	-3774.91878	-3769.323274	5.595506	11.191012	0.000821945	0.000821945	0.006006521	[illegible]
803	Anisa01314	498340662	WP_010654818.1	50S ribosomal protein L18 [Fluoribacter dumoffii]	-3477.733682	-3474.501303	3.232379	6.464758	0.011003463	0.0110034 6	0.03646496	[illegible]
807	Anisa01318	498340666	WP_010654822.1	50S ribosomal protein L5 [Fluoribacter dumoffii]	-3891.60451	-3888.598346	3.006164	6.012328	0.014206273	0.0142 0628	0.04437028	[illegible]

820	Anisa01332	654930404	WP_028380773.1	gamma-glutamyltransferase [Legionella cherrii]	-14448.48444	-14434.25025	14.23419 1	28.468382	9.52412E-08	9.52E-08	0.00000776	[illegible]
824	Anisa01337	654930400	WP_028380769.1	hypothetical protein [Legionella cherrii]	-17011.50692	-17008.16365	3.343265	6.68653	0.009714411	0.009714412	0.03345749	[illegible]
825	Anisa01338	498338347	WP_010652503.1	MFS transporter [Fluoribacter dumoffii]	-10559.5939	-10555.72221	3.871696	7.743392	0.005390944	0.005390945	0.02179318	[illegible]
834	Anisa01351	671586698	WP_031563094.1	virulence factor [Legionella wadsworthii]	-7022.751431	-7019.524942	3.226489	6.452978	0.011076653	0.0110 7665	0.03660112	[illegible]
836	Anisa01353	654930384	WP_028380753.1	iron transporter FeoB [Legionella cherrii]	-23682.91492	-23677.87639	5.038531	10.077062	0.001501265	0.001501265	0.009103415	[illegible]
848	Anisa01368	654930368	WP_028380737.1	zinc protease [Legionella cherrii]	-16513.71304	-16506.80475	6.908291	13.816582	0.00020155	0.00020155	0.002257353	[illegible]
856	Anisa01378	671586766	WP_031563150.1	phosphoesterase [Legionella wadsworthii]	-19944.88606	-19932.1068	12.77926	25.55852	4.29167E-07	0.000000429	0.0000222	[illegible]
858	Anisa01383	654930355	WP_028380724.1	tRNA dimethylallyltransferase [Legionella cherrii]	-11258.43928	-11249.56967	8.869604	17.739208	2.53351E-05	0.0000253	0.000477645	[illegible]
859	Anisa01384	654930354	WP_028380723.1	DNA mismatch repair protein MutL [Legionella cherrii]	-15930.86539	-15924.55091	6.314488	12.628976	0.000379813	0.000379813	0.00366938	[illegible]
862	Anisa01387	654930351	WP_028380720.1	sugar kinase [Legionella	-17564.73471	-17559.2725	5.462213	10.924426	0.000949046	0.000949046	0.006517545	[illegible]

				cherrii]								
871	Anisa01397	654930341	WP_028380710.1	phenylalanyl-tRNA synthetase subunit beta [Legionella cherrii]	-16134.44153	-16127.84002	6.601519	13.203038	0.000279496	0.000279496	0.002837036	[illegible]
876	Anisa01402	654930337	WP_028380706.1	threonyl-tRNA synthetase [Legionella cherrii]	-9567.00722	-9563.520141	3.487079	6.974158	0.008269512	0.008269513	0.03002307	[illegible]
879	Anisa01409	671586862	WP_031563224.1	queuine tRNA-ribosyltransferase [Legionella wadsworthii]	-11258.81189	-11253.76962	5.042277	10.084554	0.001495173	0.001495174	0.009103415	[illegible]
880	Anisa01411	498338277	WP_010652433.1	disulfide bond formation protein DsbB [Fluoribacter dumoffii]	-6445.855906	-6435.502725	10.3531811	20.706362	5.35378E-06	0.00000535	0.000138712	[illegible]
883	Anisa01418	654930323	WP_028380692.1	uroporphyrin-III methyltransferase [Legionella cherrii]	-15469.42585	-15463.53195	5.893902	11.787804	0.0005962	0.0005962	0.004889698	[illegible]
884	Anisa01419	654930322	WP_028380691.1	protoporphyrinogen oxidase [Legionella cherrii]	-7224.15028	-7220.13164	4.01864	8.03728	0.004582429	0.004582430	0.01962097	[illegible]
886	Anisa01421	671586897	WP_031563254.1	hypothetical protein [Legionella wadsworthii]	-3368.760851	-3365.012652	3.748199	7.496398	0.006182252	0.006182253	0.02405166	[illegible]
888	Anisa01424	654930317	WP_028380686.1	ribosome biogenesis	-11159.21535	-11148.83342	10.38192	20.763856	5.19543E-06	0.0000052	0.000138712	[illegible]

				GTPase RsgA [Legionella cherrii]			8					
897	Anisa01439	654931391	WP_028381757.1	polynucleotide phosphorylase/polyadenylase [Legionella cherrii]	-22196.74109	-22192.60822	4.132871	8.265742	0.004040015	0.004040015	0.01838173	[illegible]
901	Anisa01443	654931387	WP_028381753.1	translation initiation factor IF-2 [Legionella cherrii]	-5428.622801	-5422.984817	5.637984	11.275968	0.000785169	0.000785169	0.005984103	[illegible]
904	Anisa01447	654931383	WP_028381749.1	NADH:ubiquinone oxidoreductase subunit M [Legionella cherrii]	-14333.79449	-14330.71923	3.07526	6.15052	0.013137347	0.0131373735	0.04171748	[illegible]
905	Anisa01448	654931382	WP_028381748.1	NADH:ubiquinone oxidoreductase subunit L [Legionella cherrii]	-21095.03496	-21083.89619	11.138779	22.277558	2.35948E-06	0.00000236	0.0000727	[illegible]
910	Anisa01453	654931377	WP_028381743.1	NADH dehydrogenase [Legionella cherrii]	-13835.90501	-13830.14433	5.760679	11.521358	0.000688011	0.000688011	0.005446754	[illegible]
913	Anisa01456	654931374	WP_028381740.1	NADH dehydrogenase [Legionella cherrii]	-11079.35577	-11075.87921	3.476558	6.953116	0.008367337	0.008367337	0.03028179	[illegible]
918	Anisa01463	654931367	WP_028381733.1	dihydropteroate synthase [Legionella	-11324.4682	-11320.82319	3.645007	7.290014	0.006933894	0.006933894	0.02610603	[illegible]

				cherrii]								
920	Anisa01465	511303846	WP_016357037.1	ribosomal RNA large subunit methyltransferase E [Legionella pneumophila]	-5527.911331	-5523.956863	3.954468	7.908936	0.004919119	0.00491912	0.02054138	[illegible]
926	Anisa01479	654931356	WP_028381722.1	shikimate dehydrogenase [Legionella cherrii]	-10568.06429	-10564.6383	3.425995	6.85199	0.00885426	0.00885426	0.03089388	[illegible]
933	Anisa01492	654931342	WP_028381708.1	GTP-binding protein [Legionella cherrii]	-16268.13202	-16259.70498	8.427037	16.854074	4.03666E-05	0.0000404	0.000666926	[illegible]
937	Anisa01520	654931607	WP_028381970.1	membrane protein [Legionella cherrii]	-15234.75482	-15230.89378	3.861036	7.722072	0.005454976	0.0054 54976	0.02189242	[illegible]
938	Anisa01522	654931606	WP_028381969.1	DNA mismatch repair protein MutS [Legionella cherrii]	-24506.79577	-24499.59461	7.201152	14.402304	0.000147622	0.000147622	0.001764542	[illegible]
940	Anisa01530	498339205	WP_010653361.1	recombinase RecA [Fluoribacter dumoffii]	-8825.920147	-8788.357811	37.562336	75.124672	4.4191E-18	4.42E-18	5.04E-15	[illegible]
941	Anisa01531	653019466	WP_027271416.1	recombinase RecX [Legionella sainthelensi]	-5565.866827	-5546.466714	19.400113	38.800226	4.69473E-10	4.69E-10	8.92E-08	[illegible]
942	Anisa01532	654931597	WP_028381960.1	alanyl-tRNA synthetase [Legionella cherrii]	-24695.34556	-24681.30179	14.044377	28.08754	1.1595E-07	0.000000116	0.00000826	[illegible]

943	Anisa01536	654931594	WP_028381957.1	short-chain dehydrogenase [Legionella cherrii]	-10089.45537	-10082.48234	6.973024	13.946048	0.000188133	0.000188133	0.002144716	[illegible]
944	Anisa01537	654931593	WP_028381956.1	L-gululonolactone oxidase [Legionella cherrii]	-9477.001599	-9472.584931	4.416668	8.833336	0.002957772	0.002957772	0.01478886	[illegible]
949	Anisa01554	654931577	WP_028381940.1	lysyl-tRNA synthetase [Legionella cherrii]	-14708.37358	-14703.44741	4.926168	9.852336	0.001696162	0.001696163	0.009815359	[illegible]
951	Anisa01562	654931568	WP_028381931.1	hypothetical protein [Legionella cherrii]	-4986.391877	-4981.700267	4.69161	9.38322	0.002189806	0.002189806	0.01183118	[illegible]
952	Anisa01563	498339238	WP_010653394.1	malate dehydrogenase [Fluoribacter dumoffii]	-16682.24718	-16679.27911	2.968076	5.936152	0.01483337	0.01483337	0.04609022	[illegible]
956	Anisa01569	654931561	WP_028381924.1	homogentisate 1,2-dioxygenase [Legionella cherrii]	-12040.09366	-12034.97839	5.115273	10.230546	0.001381338	0.001381338	0.008623053	[illegible]
960	Anisa01573	671591892	WP_031567302.1	histidine kinase [Legionella wadsworthii]	-10642.35606	-10636.20797	6.148095	12.29619	0.000453884	0.000453884	0.004078323	[illegible]
962	Anisa01575	498339249	WP_010653405.1	septation protein A [Fluoribacter dumoffii]	-2829.922292	-2826.220844	3.701448	7.402896	0.006511896	0.006511896	0.02499516	[illegible]
964	Anisa01577	654931554	WP_028381917.1	hydroxyacylglutathione hydrolase	-10312.89188	-10303.47611	9.415773	18.831546	1.42786E-05	0.0000143	0.000324236	[illegible]

				[Legionella cherrii]								
970	Anisa01591	498339264	WP_010653420.1	tryptophan synthase subunit alpha [Fluoribacter dumoffii]	-11739.34484	-11735.91959	3.425248	6.850496	0.008861667	0.008861667	0.03089388	[illegible]
974	Anisa01600	654931535	WP_028381898.1	type II protein secretion LspD [Legionella cherrii]	-24587.42832	-24583.74769	3.680631	7.361262	0.0066644	0.0066644	0.02540942	[illegible]
977	Anisa01632	654931629	WP_028381992.1	acetyl-CoA acetyltransferase [Legionella cherrii]	-12010.33395	-12001.55177	8.782179	17.564358	2.77746E-05	0.0000278	0.000494735	[illegible]
979	Anisa01634	654931631	WP_028381994.1	methylcrotonoyl-CoA carboxylase [Legionella cherrii]	-11019.50332	-11015.21235	4.290974	8.581948	0.00339512	0.00339512	0.0161943	[illegible]
980	Anisa01635	654931632	WP_028381995.1	gamma-carboxygeranoyl-CoA hydratase [Legionella cherrii]	-7920.93639	-7914.26796	6.66843	13.33686	0.00026024	0.00026024	0.002746978	[illegible]
981	Anisa01636	654931633	WP_028381996.1	3-methylcrotonyl-CoA carboxylase [Legionella cherrii]	-11407.62696	-11387.98447	19.642498	39.284996	3.66245E-10	3.66E-10	8.35E-08	[illegible]
983	Anisa01638	654931635	WP_028381998.1	acetoacetyl-CoA synthetase [Legionella cherrii]	-12522.42414	-12518.16868	4.255467	8.510934	0.003530188	0.003530188	0.01662981	[illegible]
986	Anisa01	6549316	WP_028	hypothetical	-8523.099213	-8520.019803	3.07	6.158	0.01307	0.0130	0.041638	[illegible]

	641	40	382003.1	protein [Legionella cherrii]			941	82	5841	7584	16	
987	Anisa01642	654931641	WP_028382004.1	hypothetical protein [Legionella cherrii]	-14956.40571	-14953.37048	3.035237	6.070474	0.013746007	0.01374601	0.04316928	[illegible]
988	Anisa01644	654931642	WP_028382005.1	SAM-dependent methyltransferase [Legionella cherrii]	-9878.439339	-9867.213432	11.225907	22.451814	2.15482E-06	0.00000215	0.0000702	[illegible]
989	Anisa01646	654931643	WP_028382006.1	glycyl-tRNA synthetase subunit beta [Legionella cherrii]	-19499.66556	-19490.42685	9.238715	18.47743	1.71928E-05	0.0000172	0.00035636	[illegible]
991	Anisa01648	654931645	WP_028382008.1	hypothetical protein [Legionella cherrii]	-7512.490069	-7507.847446	4.642623	9.285246	0.002310069	0.002310069	0.01229905	[illegible]
992	Anisa01650	654931647	WP_028382010.1	proline iminopeptidase [Legionella cherrii]	-9063.075871	-9055.779886	7.295985	14.59197	0.000133482	0.000133482	0.001690772	[illegible]
1002	Anisa01667	498339143	WP_010653299.1	2-dehydro-3-deoxyphosphooctonate aldolase [Fluoribacter dumoffii]	-5401.150431	-5395.91077	5.239661	10.479322	0.00120718	0.001207181	0.007731384	[illegible]
1005	Anisa01671	654931664	WP_028382027.1	Zn-dependent protease [Legionella cherrii]	-8947.453699	-8943.88156	3.572139	7.144278	0.007520354	0.007520355	0.02773881	[illegible]

1020	Anisa01721	489728341	WP_003632457.1	acyl-CoA dehydrogenase [Legionella longbeachae]	-11663.66891	-11659.5495	4.119413	8.238826	0.004100365	0.004100365	0.01847595	[illegible]
1028	Anisa01733	654931787	WP_028382150.1	stress-induced protein [Legionella cherrii]	-10594.95348	-10589.41171	5.541772	11.083544	0.000870972	0.000870972	0.006208632	[illegible]
1033	Anisa01738	654931783	WP_028382146.1	ABC transporter ATPase [Legionella cherrii]	-15084.69141	-15080.67872	4.012687	8.025374	0.00461265	0.00461265	0.01962097	[illegible]
1034	Anisa01742	654931779	WP_028382142.1	S-adenosylmethionine tRNA ribosyltransferase [Legionella cherrii]	-11504.24799	-11494.06871	10.179276	20.358552	6.42055E-06	0.00000642	0.000159118	[illegible]
1035	Anisa01743	498339022	WP_010653178.1	queuine tRNA-ribosyltransferase [Fluoribacter dumoffii]	-10554.41093	-10540.13294	14.277992	28.555984	9.10281E-08	0.000000091	0.00000776	[illegible]
1040	Anisa01757	654931753	WP_028382116.1	multidrug MFS transporter [Legionella cherrii]	-21308.41144	-21301.93632	6.475116	12.950232	0.000319882	0.000319882	0.003143668	[illegible]
1043	Anisa01773	654932319	WP_028382681.1	4-hydroxythreonine-4-phosphate dehydrogenase [Legionella cherrii]	-8517.61604	-8513.08412	4.53192	9.06384	0.002607139	0.002607139	0.01325181	[illegible]

1044	Anisa01774	671587781	WP_031563922.1	molecular chaperone SurA [Legionella wadsworthii]	-13695.29236	-13689.76431	5.528043	11.056086	0.000883966	0.000883966	0.006259138	[illegible]
1045	Anisa01775	654932317	WP_028382679.1	LPS biosynthesis protein [Legionella cherrii]	-29264.55935	-29259.41401	5.145333	10.290666	0.001337048	0.001337048	0.008421186	[illegible]
1046	Anisa01776	654932316	WP_028382678.1	phosphotransferase [Legionella cherrii]	-11202.83555	-11198.74407	4.091485	8.18297	0.004228547	0.004228547	0.0189562	[illegible]
1049	Anisa01779	498340700	WP_010654856.1	acyl-CoA dehydrogenase [Fluoribacter dumoffii]	-24404.9349	-24399.00939	5.92551	11.85102	0.000576298	0.000576298	0.004830733	[illegible]
1051	Anisa01781	653020757	WP_027272660.1	chromate transporter [Legionella sainthelensi]	-4855.85232	-4851.615762	4.236558	8.473116	0.003604335	0.003604335	0.01683993	[illegible]
1053	Anisa01784	654932307	WP_028382669.1	EF-P beta-lysylation protein EpmB [Legionella cherrii]	-12005.0103	-12001.181	3.829293	7.658586	0.005650277	0.005650278	0.02236568	[illegible]
1067	Anisa02042	654931943	WP_028382306.1	ABC transporter ATP-binding protein [Legionella cherrii]	-13178.41111	-13175.10039	3.310715	6.62143	0.01007589	0.01007589	0.03408462	[illegible]
1068	Anisa02043	654931944	WP_028382307.1	DNA topoisomerase IV subunit B [Legionella	-9682.03702	-9678.928928	3.108092	6.216184	0.012658766	0.0126587 7	0.04072261	[illegible]

				cherrii]								
1073	Anisa02178	654931825	WP_028382188.1	electron transfer flavoprotein subunit beta [Legionella cherrii]	-9975.164368	-9968.646625	6.517743	13.035486	0.000305644	0.000305644	0.003029862	[illegible]
1074	Anisa02179	654931826	WP_028382189.1	electron transfer flavoprotein subunit beta [Legionella cherrii]	-8119.193711	-8112.825777	6.367934	12.735868	0.000358709	0.000358709	0.003495113	[illegible]
1076	Anisa02188	654931834	WP_028382197.1	peptidase M50 [Legionella cherrii]	-7223.177784	-7217.484871	5.692913	11.385826	0.000740066	0.000740066	0.005743553	[illegible]
1077	Anisa02189	654931835	WP_028382198.1	erythronate-4-phosphate dehydrogenase [Legionella cherrii]	-15242.67011	-15239.14159	3.52852	7.05704	0.007895435	0.007895435	0.0288487	[illegible]
1078	Anisa02192	654931838	WP_028382201.1	UDP-N-acetylmuramoylalanyl-D-glutamate--2, 6-diaminopimelate ligase [Legionella cherrii]	-16838.91759	-16827.68766	11.229938	22.459876	2.14579E-06	0.00000215	0.0000702	[illegible]
1081	Anisa02195	654931841	WP_028382204.1	16S rRNA methyltransferase [Legionella cherrii]	-5982.41107	-5976.86974	5.54133	11.08266	0.000871387	0.000871387	0.006208632	[illegible]
1090	Anisa02215	654931859	WP_028382222.1	cystathionine beta-lyase	-12269.71397	-12262.81719	6.896781	13.793562	0.000204034	0.000204034	0.002257353	[illegible]

				[Legionella cherrii]								
1094	Anisa02221	654931864	WP_028382227.1	alanine racemase [Legionella cherrii]	-11230.9521	-11223.67794	7.274161	14.548322	0.00013661	0.00013661	0.001692776	[illegible]
1095	Anisa02222	654931865	WP_028382228.1	DNA helicase [Legionella cherrii]	-12608.97843	-12605.00912	3.969319	7.938638	0.004839024	0.004839025	0.02028121	[illegible]
1097	Anisa02224	498338931	WP_010653087.1	helicase [Fluoribacter dumoffii]	-24782.95364	-24776.04785	6.905794	13.811588	0.000202086	0.000202086	0.002257353	[illegible]
1099	Anisa02229	671592657	WP_031567934.1	phosphatidylglycerophosphatase [Legionella wadsworthii]	-5667.821896	-5663.191243	4.630653	9.261306	0.002340461	0.002340461	0.01229905	[illegible]
1100	Anisa02230	654931873	WP_028382236.1	thiamine monophosphate kinase [Legionella cherrii]	-12590.52287	-12579.25035	11.272522	22.545044	2.05273E-06	0.00000205	0.0000702	[illegible]
1103	Anisa02233	654931877	WP_028382240.1	serine hydroxymethyltransferase [Legionella cherrii]	-10703.45706	-10699.37272	4.084341	8.168682	0.004261987	0.004261987	0.0189562	[illegible]
1105	Anisa02237	654931881	WP_028382244.1	MexH family multidrug efflux RND transporter periplasmic adaptor subunit [Legionella cherrii]	-9509.303514	-9503.705323	5.598191	11.196382	0.00081957	0.00081957	0.006006521	[illegible]

1106	Anisa02238	654931882	WP_028382245.1	acriflavine resistance protein B [Legionella cherrii]	-20819.38196	-20813.91022	5.471747	10.943494	0.000939329	0.000939329	0.006513684	[illegible]
1108	Anisa02247	654932116	WP_028382478.1	acyl-CoA thioester hydrolase [Legionella cherrii]	-3727.932792	-3721.730076	6.202716	12.405432	0.000428087	0.000428087	0.004000157	[illegible]
1110	Anisa02249	654932114	WP_028382476.1	glucosamine--fructose-6-phosphate aminotransferase [Legionella cherrii]	-10129.03765	-10125.71807	3.319582	6.639164	0.009976074	0.009976074	0.03389755	[illegible]
1111	Anisa02251	671591574	WP_031567037.1	hypothetical protein [Legionella wadsworthii]	-8565.419092	-8557.126771	8.292321	16.584642	4.65263E-05	0.0000465	0.000726575	[illegible]
1118	Anisa02259	654932105	WP_028382467.1	ribonuclease T [Legionella cherrii]	-7547.325652	-7542.613326	4.712326	9.424652	0.002140877	0.002140877	0.01167751	[illegible]
1121	Anisa02266	654932099	WP_028382461.1	endonuclease III [Legionella cherrii]	-7355.046663	-7349.698871	5.347792	10.695584	0.001073916	0.001073916	0.007186867	[illegible]
1122	Anisa02270	654932094	WP_028382456.1	bifunctional N-acetylglucosamine-1-phosphate uridyltransferase/glucosamine-1-phosphate acetyltransferase [Legionella	-15174.68983	-15166.67273	8.017099	16.034198	6.22086E-05	0.0000622	0.00092101	[illegible]

				cherrii]								
1130	Anisa02 282	6549320 84	WP_028 382446.1	hypothetical protein [Legionella cherrii]	-13542.42078	-13537.79912	4.62 1665	9.243 33	0.00236 3547	0.0023 63548	0.012303 4	[illegible]
1137	Anisa02 292	6549320 75	WP_028 382437.1	hypothetical protein [Legionella cherrii]	-12630.81702	-12627.90955	2.90 7469	5.814 938	0.01589 0614	0.0158 9061	0.047561 11	[illegible]
1138	Anisa02 293	6530205 11	WP_027 272416.1	hypothetical protein [Legionella sainthelensi]	-11944.86565	-11938.20679	6.65 8854	13.31 7708	0.00026 2911	0.0002 62911	0.002749 711	[illegible]
1145	Anisa02 402	6549298 40	WP_028 380210.1	ATP-dependent DNA helicase DinG [Legionella cherrii]	-15789.08363	-15783.48235	5.60 1286	11.20 2572	0.00081 684	0.0008 1684	0.006006 521	[illegible]
1146	Anisa02 403	6549298 41	WP_028 380211.1	glycoprotease [Legionella cherrii]	-9029.551028	-9026.089976	3.46 1052	6.922 104	0.00851 3675	0.0085 13675	0.030520 72	[illegible]
1148	Anisa02 406	6549298 45	WP_028 380215.1	BolA family transcriptional regulator [Legionella cherrii]	-4536.393911	-4533.456453	2.93 7458	5.874 916	0.01535 8106	0.0153 5811	0.046938 99	[illegible]
1149	Anisa02 408	6549298 47	WP_028 380217.1	6-phosphofructokin ase [Legionella cherrii]	-12619.05328	-12615.59723	3.45 6052	6.912 104	0.00856 1419	0.0085 61419	0.030595 67	[illegible]

1151	Anisa02410	654929849	WP_028380219.1	glutamyl-tRNA synthetase [Legionella cherrii]	-14478.8036	-14474.7729	4.030702	8.061404	0.004521813	0.004521813	0.01959459	[illegible]
1152	Anisa02412	671589421	WP_031565243.1	hypothetical protein, partial [Legionella wadsworthii]	-13871.9305	-13857.07411	14.856381	29.712762	5.01039E-08	5.01E-08	0.00000476	[illegible]
1153	Anisa02419	654929860	WP_028380230.1	multidrug DMT transporter permease [Legionella cherrii]	-9156.808721	-9153.063109	3.745612	7.491224	0.00620004	0.006200041	0.02405166	[illegible]
1154	Anisa02423	498339851	WP_010654007.1	preprotein translocase SecA [Fluoribacter dumoffii]	-6295.674583	-6284.33769	11.336893	22.673786	1.91969E-06	0.00000192	0.0000702	[illegible]
1159	Anisa02461	654929542	WP_028379912.1	NAD(P) transhydrogenase subunit alpha [Legionella cherrii]	-13314.67195	-13309.20359	5.468359	10.936718	0.00094277	0.00094277	0.006513684	[illegible]
1161	Anisa02471	654929551	WP_028379921.1	glycosyl hydrolase [Legionella cherrii]	-12932.57592	-12922.53828	10.037643	20.075286	7.44526E-06	0.00000745	0.000180587	[illegible]
1162	Anisa02472	654929552	WP_028379922.1	transporter [Legionella cherrii]	-13464.69433	-13461.76515	2.92918	5.85836	0.015503233	0.0155030323	0.04714309	[illegible]
1173	Anisa02968	498340394	WP_010654550.1	chromosome partitioning protein ParB [Fluoribacter	-7507.525001	-7504.072779	3.452222	6.904444	0.008598176	0.008598176	0.030631	[illegible]

				dumoffii]								
1176	Anisa02973	498340401	WP_010654557.1	anhydrase [Fluoribacter dumoffii]	-6606.323098	-6602.878868	3.44423	6.88846	0.008675398	0.008675398	0.03071541	[illegible]
1177	Anisa02977	498339190	WP_010653346.1	long-chain fatty acid transporter [Fluoribacter dumoffii]	-18259.29142	-18254.04905	5.242371	10.484742	0.001203644	0.001203644	0.007731384	[illegible]
1179	Anisa02980	654931614	WP_028381977.1	ATPase [Legionella cherrii]	-13171.42101	-13165.4315	5.98951	11.97902	0.000538029	0.000538029	0.004614523	[illegible]
1181	Anisa02989	654931623	WP_028381986.1	tetraacyldisaccharide 4'-kinase [Legionella cherrii]	-10853.59196	-10849.57838	4.013577	8.027154	0.004608119	0.004608119	0.01962097	[illegible]
1184	Anisa02993	654931627	WP_028381990.1	hypothetical protein [Legionella cherrii]	-5742.891852	-5739.994735	2.897117	5.794234	0.016078819	0.01607882	0.04798391	[illegible]
1185	Anisa03060	654931901	WP_028382264.1	carbonic anhydrase [Legionella cherrii]	-24173.80818	-24167.54709	6.261089	12.522178	0.00040215	0.00040215	0.003852529	[illegible]
1186	Anisa03061	654931900	WP_028382263.1	tRNA 2-thiocytidine biosynthesis protein TtcA [Legionella cherrii]	-6807.383374	-6799.134445	8.248929	16.497858	4.87051E-05	0.0000487	0.000750322	[illegible]
1188	Anisa03063	657688564	WP_029489015.1	protein-L-isoaspartate O-methyltransferase	-7102.984407	-7096.826167	6.15824	12.31648	0.000448977	0.000448977	0.004078323	[illegible]

				[Fluoribacter dumoffii]								
1189	Anisa03064	654931897	WP_028382260.1	2-amino-3-ketobutyrate CoA ligase [Legionella cherrii]	-11498.62679	-11493.97335	4.653438	9.306876	0.002282954	0.002282954	0.01221863	[illegible]
1190	Anisa03065	498338904	WP_010653060.1	l-threonine 3-dehydrogenase [Fluoribacter dumoffii]	-9107.367189	-9101.484849	5.88234	11.76468	0.000603653	0.000603653	0.00491546	[illegible]
1194	Anisa03072	654931887	WP_028382250.1	sepiapterin reductase [Legionella cherrii]	-9719.780375	-9714.741735	5.03864	10.07728	0.001501088	0.001501088	0.009103415	[illegible]
1200	Anisa03082	2231682	AAC45669.1	macrophage infectivity potentiator [Legionella anisa]	-3810.119705	-3799.077074	11.042631	22.085262	2.60804E-06	0.00000261	0.0000782	[illegible]
1202	Anisa03085	654929711	WP_028380081.1	tRNA(Ile)-lysidine synthetase [Legionella cherrii]	-14853.09711	-14850.1293	2.967812	5.935624	0.014837814	0.01483782	0.04609022	[illegible]
1203	Anisa03086	498340010	WP_010654166.1	acetyl-CoA carboxylase subunit alpha [Fluoribacter dumoffii]	-3265.56112	-3261.737945	3.823175	7.64635	0.005688731	0.005688731	0.02243998	[illegible]
1205	Anisa03088	654929708	WP_028380078.1	bifunctional biotin--[acetyl-CoA-carboxylase] synthetase/biotin operon repressor	-17118.68518	-17112.94719	5.737999	11.475998	0.000705008	0.000705008	0.005542822	[illegible]

				[Legionella cherrii]								
1208	Anisa03091	654929706	WP_028380076.1	IraAB [Legionella cherrii]	-15993.81033	-15990.93846	2.871869	5.743738	0.01654754	0.01654754	0.04874469	[illegible]
1214	Anisa03100	498340027	WP_010654183.1	mevalonate kinase [Fluoribacter dumoffii]	-4323.23789	-4317.000343	6.237547	12.475094	0.000412414	0.000412414	0.003917933	[illegible]
1216	Anisa03102	671588996	WP_031564899.1	membrane protein [Legionella wadsworthii]	-9957.259715	-9953.463453	3.796262	7.592524	0.005861083	0.005861084	0.02297802	[illegible]
1224	Anisa03124	498341116	WP_010655272.1	translation factor Sua5 [Fluoribacter dumoffii]	-12602.64682	-12598.59721	4.049611	8.099222	0.004428426	0.004428427	0.01949192	[illegible]
1226	Anisa03128	671591180	WP_031566708.1	phosphatidylserine decarboxylase [Legionella wadsworthii]	-10650.22377	-10645.98235	4.241421	8.482842	0.003585116	0.003585116	0.01681906	[illegible]
1227	Anisa03131	489729832	WP_003633948.1	hypothetical protein [Legionella longbeachae]	-13483.17576	-13469.41776	13.758001	27.516002	1.558E-07	0.000000156	0.00000987	[illegible]
1229	Anisa03138	699016736	AIU36101.1	LglB [Legionella parisiensis]	-16468.03107	-16462.84351	5.187555	10.37511	0.001277255	0.001277255	0.008134473	[illegible]
1230	Anisa03139	699016735	AIU36100.1	LglA [Legionella parisiensis]	-7163.84645	-7159.485178	4.361272	8.722544	0.003142992	0.003142992	0.01524685	[illegible]
1233	Anisa03152	654930000	WP_028380370.1	ribosome silencing factor RsfS [Legionella cherrii]	-3386.719217	-3383.853786	2.865431	5.730862	0.016669298	0.0166693	0.04889766	[illegible]

1238	Anisa03157	671589711	WP_031565488.1	UDP-N-acetyl-D-glucosamine dehydrogenase [Legionella wadsworthii]	-6558.211968	-6553.790866	4.421102	8.842204	0.002943435	0.002943435	0.01478201	[illegible]
1239	Anisa03158	654929994	WP_028380364.1	lipid-A-disaccharide synthase [Legionella cherrii]	-14089.40399	-14078.16622	11.237772	22.475544	2.12836E-06	0.00000213	0.0000702	[illegible]
1241	Anisa03162	654929991	WP_028380361.1	heat-shock protein Hsp90 [Legionella cherrii]	-14286.41763	-14275.86515	10.5524777	21.104954	4.34803E-06	0.00000435	0.000120896	[illegible]
1250	Anisa03176	654929977	WP_028380347.1	peptidase PMbA [Legionella cherrii]	-11294.46464	-11291.51265	2.951991	5.903982	0.015106694	0.01510669	0.04658106	[illegible]
1253	Anisa03181	654929974	WP_028380344.1	ribonucleotide-diphosphate reductase subunit beta [Legionella cherrii]	-8676.036088	-8671.505863	4.530225	9.06045	0.002611977	0.002611977	0.01325181	[illegible]
1256	Anisa03223	498340408	WP_010654564.1	spore maturation protein [Fluoribacter dumoffii]	-2123.174815	-2119.856552	3.318263	6.636526	0.009990857	0.009990858	0.03389755	[illegible]
1258	Anisa03225	654930699	WP_028381068.1	hypothetical protein [Legionella cherrii]	-11846.77532	-11839.28111	7.494206	14.988412	0.000108173	0.000108173	0.001417439	[illegible]
1262	Anisa03229	654930695	WP_028381064.1	cytochrome C oxidase subunit II [Legionella	-17056.16502	-17051.23299	4.932031	9.864062	0.001685386	0.001685386	0.009815359	[illegible]

				cherrii]								
1265	Anisa03237	654930686	WP_028381055.1	endopeptidase IV [Legionella cherrii]	-6192.13575	-6184.282966	7.852784	15.705568	0.000074006	0.000074	0.001062058	[illegible]
1266	Anisa03238	671587537	WP_031563742.1	endopeptidase IV [Legionella wadsworthii]	-4051.494096	-4047.106193	4.387903	8.775806	0.003052522	0.003052523	0.0150644	[illegible]
1267	Anisa03239	654930684	WP_028381053.1	excinuclease ABC subunit C [Legionella cherrii]	-15467.63654	-15459.33378	8.30276	16.60552	4.60169E-05	0.000046	0.000726575	[illegible]
1272	Anisa03246	671587524	WP_031563733.1	50S ribosomal protein L25 [Legionella wadsworthii]	-5475.651104	-5466.535286	9.115818	18.231636	1.95603E-05	0.0000196	0.000398192	[illegible]
1279	Anisa03256	671586571	WP_031562988.1	cysteine sulfinate desulfinase [Legionella wadsworthii]	-7984.60447	-7979.833348	4.771122	9.542244	0.002007961	0.002007961	0.01133206	[illegible]
1280	Anisa03257	654930433	WP_028380802.1	Fe-S cluster assembly protein SufD [Legionella cherrii]	-16130.99009	-16122.14429	8.845798	17.691596	2.59772E-05	0.000026	0.000477645	[illegible]
1282	Anisa03259	489730537	WP_003634653.1	cysteine desulfurase activator complex subunit SufB [Legionella longbeachae]	-11742.16941	-11738.4242	3.745212	7.490424	0.006202795	0.006202796	0.02405166	[illegible]
1294	Anisa03278	654930406	WP_028380775.1	5-hydroxymethyluracil DNA	-11071.43933	-11067.31788	4.121452	8.242904	0.004091162	0.004091163	0.01847595	[illegible]

				glycosylase [Legionella cherrii]								
1298	Anisa03354	654929884	WP_028380254.1	general secretion pathway protein GspL [Legionella cherrii]	-13479.05485	-13474.07671	4.978143	9.956286	0.001603011	0.001603011	0.009517878	[illegible]
1303	Anisa03363	654929891	WP_028380261.1	integrase [Legionella cherrii]	-13260.5753	-13257.19029	3.385009	6.770018	0.00927019	0.00927019	0.03212163	[illegible]
1316	Anisa03389	518063275	WP_019233483.1	phosphoesterase [Legionella anisa]	-3556.388024	-3551.043769	5.344255	10.68851	0.00107803	0.00107803	0.007186867	[illegible]
1318	Anisa03391	498340560	WP_010654716.1	phosphoesterase [Fluoribacter dumoffii]	-19394.80933	-19388.0201	6.789233	13.578466	0.000228795	0.000228795	0.002460625	[illegible]
1319	Anisa03392	498340561	WP_010654717.1	phosphoesterase [Fluoribacter dumoffii]	-11267.92115	-11264.06943	3.851729	7.703458	0.005511514	0.005511514	0.02189242	[illegible]
1324	Anisa03534	654929912	WP_028380282.1	orotate phosphoribosyltransferase [Legionella cherrii]	-6429.720887	-6424.704498	5.016389	10.032778	0.001537789	0.001537789	0.009275553	[illegible]
1329	Anisa03588	654932073	WP_028382435.1	hypothetical protein [Legionella cherrii]	-23032.69311	-23026.53573	6.157381	12.314762	0.00044939	0.00044939	0.004078323	[illegible]
1332	Anisa03592	498338108	WP_010652264.1	cobyric acid synthase CobQ [Fluoribacter dumoffii]	-7336.205294	-7332.141827	4.063467	8.126934	0.004361244	0.004361244	0.01927061	[illegible]
1333	Anisa03593	671591479	WP_031566958.1	chromosome partitioning	-10862.65013	-10856.95791	5.692223	11.384446	0.000740616	0.000740616	0.005743553	[illegible]

				protein ParB [Legionella wadsworthii]								
1335	Anisa03597	498338103	WP_010652259.1	cytochrome oxidase subunit I [Fluoribacter dumoffii]	-14020.11658	-14014.53399	5.582589	11.165178	0.00083347	0.00083347	0.006051948	[illegible]
1339	Anisa03629	654931413	WP_028381778.1	elongation factor G [Legionella cherrii]	-17065.36057	-17061.8935	3.467067	6.934134	0.0084566	0.0084566	0.03050799	[illegible]
1347	Anisa03637	498340688	WP_010654844.1	MULTISPECIES: 50S ribosomal protein L11 [Legionellaceae]	-3371.700763	-3368.816339	2.884424	5.768848	0.016312725	0.01631273	0.04850497	[illegible]
1355	Anisa03714	498339810	WP_010653966.1	1-(5-phosphoribosyl)-5-[(5-phosphoribosylamino)methylideneamino] imidazole-4-carboxamide isomerase [Fluoribacter dumoffii]	-10125.19091	-10117.39293	7.797979	15.595958	0.000078422	0.0000784	0.001090258	[illegible]
1359	Anisa03718	654929909	WP_028380279.1	histidinol dehydrogenase [Legionella cherrii]	-17825.78941	-17818.34272	7.446691	14.893382	0.000113761	0.000113761	0.001473722	[illegible]
1360	Anisa03719	654929910	WP_028380280.1	ATP phosphoribosyltransferase [Legionella	-8073.647898	-8063.286478	10.36142	20.72284	5.30791E-06	0.00000531	0.000138712	[illegible]

				cherrii]								
1363	Anisa03 814	6549311 05	WP_028 381472.1	DNA topoisomerase IV subunit A [Legionella cherrii]	-22895.35456	-22891.40506	3.94 9493	7.898 986	0.00494 6252	0.0049 46252	0.020543 93	[illegible]
1365	Anisa03 817	6990167 47	AIU3611 2.1	LglM [Legionella parisiensis]	-14352.69306	-14344.34401	8.34 9048	16.69 8096	0.00004 3825	0.0000 438	0.000703 669	[illegible]

4d: Results of the GO term enrichment analysis of the core genes predicted to be under positive selection in Clade 2 compared to other Legionella clades. Performed hypergeometric probability distribution Test

**GOID**	**Ontology**	**Term**	**Level**	**q**	**m**	**t**	**k**	**log_odds_ratio**	**FDR corrected p-value at significance level of 0.05**
GO:0003674	molecular_function	molecular_function	0	286	5235	12977	362	0.96972091	1.10E-49
GO:0003676	molecular_function	nucleic acid binding	3	50	1016	12977	362	0.818994898	0.00038868
GO:0003684	molecular_function	damaged DNA binding	5	4	15	12977	362	3.256932799	0.003110314
GO:0005488	molecular_function	binding	1	159	2476	12977	362	1.202910751	2.67E-26
GO:0097159	molecular_function	organic cyclic compound binding	2	117	1879	12977	362	1.158438763	8.14E-17
GO:1901363	molecular_function	heterocyclic compound binding	2	117	1879	12977	362	1.158438763	8.14E-17
GO:0003824	molecular_function	catalytic activity	1	247	3902	12977	362	1.182192564	1.69E-49

GO:0008094	molecular_function	DNA-dependent ATPase activity	9	6	38	12977	362	2.500858382	0.002915011
GO:0016462	molecular_function	pyrophosphatase activity	5	22	323	12977	362	1.287864659	0.000666244
GO:0016787	molecular_function	hydrolase activity	2	71	1220	12977	362	1.060905082	1.86E-08
GO:0016817	molecular_function	hydrolase activity, acting on acid anhydrides	3	22	329	12977	362	1.26131124	0.000821326
GO:0016818	molecular_function	hydrolase activity, acting on acid anhydrides, in phosphorus-containing anhydrides	4	22	326	12977	362	1.274526859	0.000745039
GO:0017111	molecular_function	nucleoside-triphosphatase activity	6	21	314	12977	362	1.261520069	0.00111621
GO:0042623	molecular_function	ATPase activity, coupled	8	10	153	12977	362	1.228363647	0.033559805
GO:0006139	biological_process	nucleobase-containing compound metabolic process	4	84	1118	12977	362	1.429436345	3.91E-16

GO:0006259	biological_process	DNA metabolic process	6	24	531	12977	362	0.696217845	0.04221412
GO:0006725	biological_process	cellular aromatic compound metabolic process	3	105	1244	12977	362	1.597298142	1.15E-24
GO:0006807	biological_process	nitrogen compound metabolic process	2	130	1493	12977	362	1.642192758	4.57E-33
GO:0008150	biological_process	biological_process	0	297	5467	12977	362	0.961608855	1.74E-53
GO:0008152	biological_process	metabolic process	1	271	4493	12977	362	1.112509089	1.74E-53
GO:0009987	biological_process	cellular process	1	203	2986	12977	362	1.285160862	7.18E-41
GO:0034641	biological_process	cellular nitrogen compound metabolic process	3	103	1271	12977	362	1.538575607	9.15E-23
GO:0043170	biological_process	macromolecule metabolic process	3	105	1597	12977	362	1.236920315	1.80E-16
GO:0044237	biological_process	cellular metabolic process	2	182	2220	12977	362	1.555274074	1.37E-46

GO:0044238	biological_process	primary metabolic process	2	165	2261	12977	362	1.387400332	2.10E-34
GO:0044260	biological_process	cellular macromolecule metabolic process	4	88	1235	12977	362	1.352959687	1.36E-15
GO:0046483	biological_process	heterocycle metabolic process	3	104	1260	12977	362	1.565055094	1.19E-23
GO:0071704	biological_process	organic substance metabolic process	2	191	2451	12977	362	1.482097454	2.07E-46
GO:0090304	biological_process	nucleic acid metabolic process	5	59	902	12977	362	1.229482821	8.56E-09
GO:1901360	biological_process	organic cyclic compound metabolic process	3	109	1288	12977	362	1.601090841	7.41E-26
GO:0006950	biological_process	response to stress	2	14	177	12977	362	1.503572767	0.002313249
GO:0006974	biological_process	response to DNA damage stimulus	5	11	98	12977	362	2.008545169	0.000564158
GO:0033554	biological_process	cellular response to stress	4	12	114	12977	362	1.915895881	0.000528234

GO:0044699	biological_process	single-organism process	1	55	1366	12977	362	0.52944134	0.012171682
GO:0000166	molecular_function	nucleotide binding	4	85	946	12977	362	1.687517957	3.13E-21
GO:0036094	molecular_function	small molecule binding	2	87	986	12977	362	1.661323054	3.13E-21
GO:1901265	molecular_function	nucleoside phosphate binding	3	85	946	12977	362	1.687517957	3.13E-21
GO:0005575	cellular_component	cellular_component	0	142	2157	12977	362	1.238760053	8.75E-24
GO:0005622	cellular_component	intracellular	3	93	931	12977	362	1.840344848	7.95E-27
GO:0005623	cellular_component	cell	1	118	1249	12977	362	1.759908682	7.91E-33
GO:0005737	cellular_component	cytoplasm	5	86	572	12977	362	2.430216813	1.19E-37
GO:0044424	cellular_component	intracellular part	4	91	680	12977	362	2.262227099	6.72E-36
GO:0044464	cellular_component	cell part	2	116	1200	12977	362	1.792985699	4.57E-33
GO:0001882	molecular_function	nucleoside binding	3	68	776	12977	362	1.651373394	3.91E-16
GO:0001883	molecular_function	purine nucleoside binding	4	67	772	12977	362	1.637455548	1.09E-15
GO:0005524	molecular_function	ATP binding	8	57	713	12977	362	1.518955142	8.06E-12
GO:0017076	molecular_function	purine nucleotide binding	5	67	778	12977	362	1.62628624	1.49E-15

GO:0030554	molecular_function	adenyl nucleotide binding	6	58	715	12977	362	1.540004958	2.80E-12
GO:0032549	molecular_function	ribonucleoside binding	4	67	773	12977	362	1.635587981	1.14E-15
GO:0032550	molecular_function	purine ribonucleoside binding	5	67	772	12977	362	1.637455548	1.09E-15
GO:0032553	molecular_function	ribonucleotide binding	5	70	801	12977	362	1.647447979	1.46E-16
GO:0032555	molecular_function	purine ribonucleotide binding	6	67	777	12977	362	1.628141797	1.43E-15
GO:0032559	molecular_function	adenyl ribonucleotide binding	7	58	714	12977	362	1.542024126	2.68E-12
GO:0035639	molecular_function	purine ribonucleoside triphosphate binding	4	66	771	12977	362	1.617630464	3.54E-15
GO:0043167	molecular_function	ion binding	2	121	1450	12977	362	1.580849447	1.18E-28
GO:0043168	molecular_function	anion binding	3	88	1011	12977	362	1.641687731	3.90E-21
GO:0006281	biological_process	DNA repair	7	11	98	12977	362	2.008545169	0.000564158
GO:0043566	molecular_function	structure-specific DNA binding	5	3	17	12977	362	2.661323054	0.033766134
GO:0003723	molecular_function	RNA binding	4	20	162	12977	362	2.145901487	3.06E-07
GO:0009451	biological_process	RNA modification	7	15	61	12977	362	3.139976653	1.34E-09

GO:0016070	biological_process	RNA metabolic process	6	34	318	12977	362	1.938403281	2.91E-10
GO:0043412	biological_process	macromolecule modification	4	26	314	12977	362	1.569642364	7.51E-06
GO:0016853	molecular_function	isomerase activity	2	13	121	12977	362	1.945399876	0.000262592
GO:0016740	molecular_function	transferase activity	2	79	1400	12977	362	1.016393031	8.05E-09
GO:0090305	biological_process	nucleic acid phosphodiester bond hydrolysis	6	11	149	12977	362	1.404086493	0.011428985
GO:0009058	biological_process	biosynthetic process	2	104	1051	12977	362	1.826716159	4.42E-30
GO:0009059	biological_process	macromolecule biosynthetic process	4	39	502	12977	362	1.47768206	8.73E-08
GO:0034645	biological_process	cellular macromolecule biosynthetic process	5	37	432	12977	362	1.618389258	1.70E-08
GO:0044249	biological_process	cellular biosynthetic process	3	97	891	12977	362	1.964454615	4.04E-31

GO:1901576	biological_process	organic substance biosynthetic process	3	99	961	12977	362	1.884787394	6.95E-30
GO:0004518	molecular_function	nuclease activity	4	12	155	12977	362	1.47266149	0.005705944
GO:0016788	molecular_function	hydrolase activity, acting on ester bonds	3	19	285	12977	362	1.256932799	0.002169897
GO:0018130	biological_process	heterocycle biosynthetic process	4	44	458	12977	362	1.784051225	1.10E-11
GO:0019438	biological_process	aromatic compound biosynthetic process	4	43	426	12977	362	1.855378529	3.97E-12
GO:0034654	biological_process	nucleobase-containing compound biosynthetic process	5	26	342	12977	362	1.446410598	3.41E-05
GO:0044271	biological_process	cellular nitrogen compound biosynthetic process	4	41	460	12977	362	1.675885348	7.03E-10

GO:1901362	biological_process	organic cyclic compound biosynthetic process	4	47	476	12977	362	1.823594483	6.98E-13
GO:0004527	molecular_function	exonuclease activity	5	7	48	12977	362	2.386215816	0.001847115
GO:0008408	molecular_function	3'-5' exonuclease activity	6	4	13	12977	362	3.463383677	0.00186796
GO:0016043	biological_process	cellular component organization	2	20	133	12977	362	2.430469054	1.12E-08
GO:0022607	biological_process	cellular component assembly	3	10	61	12977	362	2.555014152	5.56E-05
GO:0034622	biological_process	cellular macromolecular complex assembly	5	3	17	12977	362	2.661323054	0.033766134
GO:0044085	biological_process	cellular component biogenesis	2	25	119	12977	362	2.912861821	4.78E-14
GO:0071840	biological_process	cellular component organization or biogenesis	1	28	176	12977	362	2.511746698	1.46E-12
GO:0032991	cellular_component	macromolecular complex	1	20	224	12977	362	1.678396567	4.29E-05
GO:0043234	cellular_component	protein complex	2	13	150	12977	362	1.635444422	0.001598804

GO:0030312	cellular_component	external encapsulating structure	4	6	35	12977	362	2.619502878	0.001958677
GO:0030313	cellular_component	cell envelope	4	6	29	12977	362	2.8908049	0.000749056
GO:0031975	cellular_component	envelope	3	6	29	12977	362	2.8908049	0.000749056
GO:0042597	cellular_component	periplasmic space	3	5	24	12977	362	2.900788989	0.002351218
GO:0044462	cellular_component	external encapsulating structure part	5	5	28	12977	362	2.678396567	0.004353078
GO:0071944	cellular_component	cell periphery	3	23	273	12977	362	1.59462821	2.35E-05
GO:0071702	biological_process	organic substance transport	4	15	240	12977	362	1.163823395	0.011428985
GO:0005886	cellular_component	plasma membrane	4	18	240	12977	362	1.4268578	0.000813603
GO:0005975	biological_process	carbohydrate metabolic process	3	16	264	12977	362	1.119429275	0.011428985
GO:0005996	biological_process	monosaccharide metabolic process	5	5	30	12977	362	2.578860894	0.005655508
GO:0006006	biological_process	glucose metabolic process	7	4	27	12977	362	2.408935892	0.020947093
GO:0006091	biological_process	generation of precursor metabolites and energy	3	8	70	12977	362	2.034540378	0.003366195

GO:0009056	biological_process	catabolic process	2	22	191	12977	362	2.045826185	2.24E-07
GO:0016052	biological_process	carbohydrate catabolic process	4	4	35	12977	362	2.034540378	0.046070771
GO:0019318	biological_process	hexose metabolic process	6	4	27	12977	362	2.408935892	0.020947093
GO:0044723	biological_process	single-organism carbohydrate metabolic process	4	11	131	12977	362	1.589832012	0.004750734
GO:0044724	biological_process	single-organism carbohydrate catabolic process	5	4	29	12977	362	2.3058424	0.02619071
GO:1901575	biological_process	organic substance catabolic process	3	22	177	12977	362	2.155649463	5.73E-08
GO:0044444	cellular_component	cytoplasmic part	6	12	125	12977	362	1.783001611	0.00109191
GO:0008168	molecular_function	methyltransferase activity	4	16	195	12977	362	1.556493081	0.000698717
GO:0008170	molecular_function	N-methyltransferase activity	5	6	21	12977	362	3.356468473	0.000145834

GO:0008276	molecular_function	protein methyltransferase activity	5	4	14	12977	362	3.356468473	0.002444193
GO:0008757	molecular_function	S-adenosylmethionine-dependent methyltransferase activity	5	11	53	12977	362	2.895334559	2.10E-06
GO:0016741	molecular_function	transferase activity, transferring one-carbon groups	3	18	212	12977	362	1.605827942	0.000226665
GO:0006479	biological_process	protein methylation	8	4	15	12977	362	3.256932799	0.003110314
GO:0008213	biological_process	protein alkylation	7	4	15	12977	362	3.256932799	0.003110314
GO:0019538	biological_process	protein metabolic process	4	47	586	12977	362	1.523655392	9.07E-10
GO:0032259	biological_process	methylation	2	16	195	12977	362	1.556493081	0.000698717
GO:0043414	biological_process	macromolecule methylation	5	11	53	12977	362	2.895334559	2.10E-06
GO:0044267	biological_process	cellular protein metabolic process	5	33	382	12977	362	1.630788686	1.04E-07

GO:0006753	biological_process	nucleoside phosphate metabolic process	6	23	189	12977	362	2.125142926	3.91E-08
GO:0006793	biological_process	phosphorus metabolic process	3	44	592	12977	362	1.413801648	3.57E-08
GO:0006796	biological_process	phosphate-containing compound metabolic process	4	43	588	12977	362	1.390415805	8.61E-08
GO:0009117	biological_process	nucleotide metabolic process	7	22	184	12977	362	2.099693057	1.14E-07
GO:0009165	biological_process	nucleotide biosynthetic process	8	18	133	12977	362	2.278465961	3.58E-07
GO:0009226	biological_process	nucleotide-sugar biosynthetic process	9	2	7	12977	362	3.356468473	0.04357665
GO:0019637	biological_process	organophosphate metabolic process	4	34	255	12977	362	2.256932799	6.24E-13
GO:0044281	biological_process	small molecule metabolic process	3	88	658	12977	362	2.26131124	1.36E-34

GO:0044710	biological_process	single-organism metabolic process	2	133	1380	12977	362	1.788653278	1.21E-38
GO:0055086	biological_process	nucleobase-containing small molecule metabolic process	5	31	227	12977	362	2.291471218	4.03E-12
GO:0090407	biological_process	organophosphate biosynthetic process	5	27	193	12977	362	2.32625386	8.02E-11
GO:1901135	biological_process	carbohydrate derivative metabolic process	3	42	259	12977	362	2.53933253	4.10E-19
GO:1901137	biological_process	carbohydrate derivative biosynthetic process	4	32	166	12977	362	2.788783963	9.38E-17
GO:1901293	biological_process	nucleoside phosphate biosynthetic process	7	18	136	12977	362	2.246285555	5.05E-07
GO:0006629	biological_process	lipid metabolic process	3	16	188	12977	362	1.609234543	0.000504933

GO:0008610	biological_process	lipid biosynthetic process	4	11	97	12977	362	2.023342171	0.000528234
GO:0008653	biological_process	lipopolysaccharide metabolic process	6	3	14	12977	362	2.941430973	0.020857606
GO:0009103	biological_process	lipopolysaccharide biosynthetic process	8	3	12	12977	362	3.163823395	0.014168081
GO:0044255	biological_process	cellular lipid metabolic process	4	14	120	12977	362	2.064287721	5.41E-05
GO:0044262	biological_process	cellular carbohydrate metabolic process	4	6	63	12977	362	1.771505972	0.026096623
GO:0043169	molecular_function	cation binding	3	55	612	12977	362	1.687795265	2.17E-13
GO:0046872	molecular_function	metal ion binding	4	55	596	12977	362	1.726014588	7.10E-14
GO:0006732	biological_process	coenzyme metabolic process	4	19	101	12977	362	2.753539425	6.54E-10
GO:0006733	biological_process	oxidoreduction coenzyme metabolic process	5	7	35	12977	362	2.8418953	0.000299634

GO:0051186	biological_process	cofactor metabolic process	3	26	143	12977	362	2.704391776	4.71E-13
GO:0055114	biological_process	oxidation-reduction process	3	51	712	12977	362	1.360515306	6.88E-09
GO:0072524	biological_process	pyridine-containing compound metabolic process	4	4	32	12977	362	2.163823395	0.035463766
GO:1901564	biological_process	organonitrogen compound metabolic process	3	81	587	12977	362	2.306456704	1.07E-32
GO:0006082	biological_process	organic acid metabolic process	4	52	375	12977	362	2.313516327	1.23E-20
GO:0006520	biological_process	cellular amino acid metabolic process	7	40	269	12977	362	2.414289127	8.24E-17
GO:0006575	biological_process	cellular modified amino acid metabolic process	8	7	47	12977	362	2.416589465	0.001628664

GO:0006790	biological_process	sulfur compound metabolic process	3	6	50	12977	362	2.104929706	0.009954243
GO:0019752	biological_process	carboxylic acid metabolic process	6	52	370	12977	362	2.332881652	6.98E-21
GO:0043436	biological_process	oxoacid metabolic process	5	52	372	12977	362	2.325104302	8.72E-21
GO:0016757	molecular_function	transferase activity, transferring glycosyl groups	3	7	79	12977	362	1.667397569	0.021364469
GO:0016763	molecular_function	transferase activity, transferring pentosyl groups	4	6	31	12977	362	2.794589585	0.001052709
GO:0006396	biological_process	RNA processing	7	20	83	12977	362	3.110712058	1.77E-12
GO:0006399	biological_process	tRNA metabolic process	8	18	86	12977	362	2.907483641	3.43E-10
GO:0006400	biological_process	tRNA modification	10	6	37	12977	362	2.53933253	0.002568433
GO:0008033	biological_process	tRNA processing	9	10	51	12977	362	2.813326148	1.16E-05

GO:0010467	biological_process	gene expression	4	41	358	12977	362	2.037559622	2.55E-13
GO:0034470	biological_process	ncRNA processing	8	18	73	12977	362	3.143923837	1.79E-11
GO:0034660	biological_process	ncRNA metabolic process	7	26	108	12977	362	3.109375611	4.17E-16
GO:0008616	biological_process	queuosine biosynthetic process	11	3	8	12977	362	3.748785895	0.004734987
GO:0009116	biological_process	nucleoside metabolic process	6	21	123	12977	362	2.613626312	4.22E-10
GO:0009119	biological_process	ribonucleoside metabolic process	7	18	102	12977	362	2.661323054	5.91E-09
GO:0009163	biological_process	nucleoside biosynthetic process	7	16	76	12977	362	2.915895881	3.71E-09
GO:0042455	biological_process	ribonucleoside biosynthetic process	8	16	76	12977	362	2.915895881	3.71E-09
GO:0046116	biological_process	queuosine metabolic process	8	3	8	12977	362	3.748785895	0.004734987
GO:1901566	biological_process	organonitrogen compound biosynthetic process	4	55	359	12977	362	2.457343074	6.31E-24

GO:1901657	biological_process	glycosyl compound metabolic process	3	21	123	12977	362	2.613626312	4.22E-10
GO:1901659	biological_process	glycosyl compound biosynthetic process	4	16	76	12977	362	2.915895881	3.71E-09
GO:0016491	molecular_function	oxidoreductase activity	2	48	680	12977	362	1.339394959	3.47E-08
GO:0016627	molecular_function	oxidoreductase activity, acting on the CH-CH group of donors	3	6	49	12977	362	2.134076051	0.009120825
GO:0048037	molecular_function	cofactor binding	2	25	281	12977	362	1.673253264	3.46E-06
GO:0050662	molecular_function	coenzyme binding	3	16	188	12977	362	1.609234543	0.000504933
GO:0050660	molecular_function	flavin adenine dinucleotide binding	5	8	73	12977	362	1.973998836	0.004310523
GO:0016874	molecular_function	ligase activity	2	29	197	12977	362	2.39975257	3.65E-12
GO:0006412	biological_process	translation	6	20	131	12977	362	2.452328488	8.59E-09
GO:0006418	biological_process	tRNA aminoacylation for protein translation	10	9	36	12977	362	3.163823395	4.80E-06

GO:0043038	biological_process	amino acid activation	8	9	37	12977	362	3.12429503	6.05E-06
GO:0043039	biological_process	tRNA aminoacylation	9	9	37	12977	362	3.12429503	6.05E-06
GO:0008270	molecular_function	zinc ion binding	6	10	129	12977	362	1.474524234	0.012171682
GO:0046914	molecular_function	transition metal ion binding	5	18	240	12977	362	1.4268578	0.000813603
GO:0000049	molecular_function	tRNA binding	5	4	21	12977	362	2.771505972	0.009660596
GO:0004812	molecular_function	aminoacyl-tRNA ligase activity	5	9	37	12977	362	3.12429503	6.05E-06
GO:0016875	molecular_function	ligase activity, forming carbon-oxygen bonds	3	9	38	12977	362	3.085820883	7.51E-06
GO:0016876	molecular_function	ligase activity, forming aminoacyl-tRNA and related compounds	4	9	38	12977	362	3.085820883	7.51E-06
GO:0006743	biological_process	ubiquinone metabolic process	6	5	15	12977	362	3.578860894	0.000287995

GO:0006744	biological_process	ubiquinone biosynthetic process	7	5	15	12977	362	3.578860894	0.000287995
GO:0009108	biological_process	coenzyme biosynthetic process	5	17	80	12977	362	2.929358141	9.07E-10
GO:0042180	biological_process	cellular ketone metabolic process	4	5	21	12977	362	3.093434067	0.001283371
GO:0042181	biological_process	ketone biosynthetic process	5	5	16	12977	362	3.48575149	0.00038868
GO:0042375	biological_process	quinone cofactor metabolic process	4	5	15	12977	362	3.578860894	0.000287995
GO:0044283	biological_process	small molecule biosynthetic process	4	33	248	12977	362	2.254021204	1.54E-12
GO:0044711	biological_process	single-organism biosynthetic process	3	33	255	12977	362	2.213864077	3.23E-12
GO:0045426	biological_process	quinone cofactor biosynthetic process	6	5	15	12977	362	3.578860894	0.000287995

GO:0051188	biological_process	cofactor biosynthetic process	4	22	114	12977	362	2.790364999	1.15E-11
GO:1901661	biological_process	quinone metabolic process	5	5	15	12977	362	3.578860894	0.000287995
GO:1901663	biological_process	quinone biosynthetic process	6	5	15	12977	362	3.578860894	0.000287995
GO:0016044	biological_process	cellular membrane organization	4	4	14	12977	362	3.356468473	0.002444193
GO:0051205	biological_process	protein insertion into membrane	6	2	6	12977	362	3.578860894	0.033559805
GO:0051668	biological_process	localization within membrane	4	2	6	12977	362	3.578860894	0.033559805
GO:0061024	biological_process	membrane organization	3	4	14	12977	362	3.356468473	0.002444193
GO:0008360	biological_process	regulation of cell shape	6	6	27	12977	362	2.993898393	0.000535905
GO:0022603	biological_process	regulation of anatomical structure morphogenesis	4	6	28	12977	362	2.941430973	0.000641724

GO:0022604	biological_process	regulation of cell morphogenesis	5	6	28	12977	362	2.941430973	0.000641724
GO:0050793	biological_process	regulation of developmental process	3	6	28	12977	362	2.941430973	0.000641724
GO:0051128	biological_process	regulation of cellular component organization	4	6	36	12977	362	2.578860894	0.002278413
GO:0065008	biological_process	regulation of biological quality	2	10	91	12977	362	1.977956849	0.001211314
GO:0051301	biological_process	cell division	3	8	43	12977	362	2.73755864	0.000174372
GO:0016879	molecular_function	ligase activity, forming carbon-nitrogen bonds	3	15	101	12977	362	2.412502508	1.44E-06
GO:0016881	molecular_function	acid-amino acid ligase activity	4	7	46	12977	362	2.447616361	0.001430935
GO:0045229	biological_process	external encapsulating structure organization	3	11	37	12977	362	3.413801648	4.18E-08

GO:0071554	biological_process	cell wall organization or biogenesis	3	8	41	12977	362	2.80627139	0.000124374
GO:0071555	biological_process	cell wall organization	4	8	30	12977	362	3.256932799	1.16E-05
GO:0000270	biological_process	peptidoglycan metabolic process	6	8	44	12977	362	2.704391776	0.000206753
GO:0006022	biological_process	aminoglycan metabolic process	4	8	49	12977	362	2.549113551	0.000390967
GO:0006023	biological_process	aminoglycan biosynthetic process	5	7	26	12977	362	3.270738599	4.54E-05
GO:0006024	biological_process	glycosaminoglycan biosynthetic process	6	7	26	12977	362	3.270738599	4.54E-05
GO:0009252	biological_process	peptidoglycan biosynthetic process	8	7	26	12977	362	3.270738599	4.54E-05
GO:0009273	biological_process	peptidoglycan-based cell wall biogenesis	5	7	26	12977	362	3.270738599	4.54E-05
GO:0030203	biological_process	glycosaminoglycan metabolic process	5	8	45	12977	362	2.671970298	0.000242509

GO:0042546	biological_process	cell wall biogenesis	4	7	26	12977	362	3.270738599	4.54E-05
GO:0044036	biological_process	cell wall macromolecule metabolic process	5	7	35	12977	362	2.8418953	0.000299634
GO:0044038	biological_process	cell wall macromolecule biosynthetic process	7	7	26	12977	362	3.270738599	4.54E-05
GO:0070589	biological_process	cellular component macromolecule biosynthetic process	6	7	26	12977	362	3.270738599	4.54E-05
GO:0007049	biological_process	cell cycle	3	8	47	12977	362	2.609234543	0.000297919
GO:0019867	cellular_component	outer membrane	2	5	48	12977	362	1.900788989	0.033441989
GO:0043163	biological_process	cell envelope organization	4	3	7	12977	362	3.941430973	0.003303069
GO:0043165	biological_process	Gram-negative-bacterium-type cell outer membrane assembly	6	3	7	12977	362	3.941430973	0.003303069

GO:0044091	biological_process	membrane biogenesis	3	3	8	12977	362	3.748785895	0.004734987
GO:0071709	biological_process	membrane assembly	5	3	8	12977	362	3.748785895	0.004734987
GO:0004536	molecular_function	deoxyribonuclease activity	5	3	19	12977	362	2.500858382	0.043831872
GO:0019439	biological_process	aromatic compound catabolic process	4	7	73	12977	362	1.781353758	0.014794781
GO:0034655	biological_process	nucleobase-containing compound catabolic process	5	6	44	12977	362	2.289354277	0.005609317
GO:0044248	biological_process	cellular catabolic process	3	17	136	12977	362	2.163823395	2.64E-06
GO:0044270	biological_process	cellular nitrogen compound catabolic process	4	6	68	12977	362	1.661323054	0.035463766
GO:0046700	biological_process	heterocycle catabolic process	4	7	71	12977	362	1.821431197	0.013097886
GO:1901361	biological_process	organic cyclic compound catabolic process	4	8	75	12977	362	1.935004704	0.004849483

GO:0003916	molecular_function	DNA topoisomerase activity	5	3	11	12977	362	3.289354277	0.011383731
GO:0003918	molecular_function	DNA topoisomerase type II (ATP-hydrolyzing) activity	10	3	7	12977	362	3.941430973	0.003303069
GO:0061505	molecular_function	DNA topoisomerase II activity	6	3	7	12977	362	3.941430973	0.003303069
GO:0006265	biological_process	DNA topological change	8	3	11	12977	362	3.289354277	0.011383731
GO:0071103	biological_process	DNA conformation change	7	6	51	12977	362	2.076360553	0.010929177
GO:0005694	cellular_component	chromosome	7	3	17	12977	362	2.661323054	0.033766134
GO:0043226	cellular_component	organelle	1	10	149	12977	362	1.266582969	0.028601916
GO:0043228	cellular_component	non-membrane-bounded organelle	2	10	130	12977	362	1.463383677	0.012799425
GO:0043229	cellular_component	intracellular organelle	5	10	148	12977	362	1.276298124	0.027400937

GO:0043232	cellular_component	intracellular non-membrane-bounded organelle	6	10	130	12977	362	1.463383677	0.012799425
GO:0006364	biological_process	rRNA processing	9	8	25	12977	362	3.519967205	2.72E-06
GO:0016072	biological_process	rRNA metabolic process	8	8	25	12977	362	3.519967205	2.72E-06
GO:0022613	biological_process	ribonucleoprotein complex biogenesis	3	10	35	12977	362	3.356468473	3.07E-07
GO:0042254	biological_process	ribosome biogenesis	4	10	35	12977	362	3.356468473	3.07E-07
GO:0008173	molecular_function	RNA methyltransferase activity	5	7	24	12977	362	3.386215816	2.72E-05
GO:0008649	molecular_function	rRNA methyltransferase activity	6	7	18	12977	362	3.801253315	3.32E-06
GO:0016435	molecular_function	rRNA (guanine) methyltransferase activity	7	2	6	12977	362	3.578860894	0.033559805
GO:0000154	biological_process	rRNA modification	10	7	18	12977	362	3.801253315	3.32E-06
GO:0001510	biological_process	RNA methylation	8	7	25	12977	362	3.327322127	3.58E-05

GO:0031167	biological_process	rRNA methylation	11	7	18	12977	362	3.801253315	3.32E-06
GO:0000287	molecular_function	magnesium ion binding	5	16	109	12977	362	2.39563907	6.54E-07
GO:0072527	biological_process	pyrimidine-containing compound metabolic process	4	7	42	12977	362	2.578860894	0.000840554
GO:0072528	biological_process	pyrimidine-containing compound biosynthetic process	5	7	34	12977	362	2.883715475	0.00028143
GO:0006766	biological_process	vitamin metabolic process	4	7	53	12977	362	2.243257862	0.003110314
GO:0006767	biological_process	water-soluble vitamin metabolic process	5	7	53	12977	362	2.243257862	0.003110314
GO:0009110	biological_process	vitamin biosynthetic process	5	6	50	12977	362	2.104929706	0.009954243
GO:0042364	biological_process	water-soluble vitamin biosynthetic process	6	6	50	12977	362	2.104929706	0.009954243

GO:0006643	biological_process	membrane lipid metabolic process	5	4	19	12977	362	2.915895881	0.007019192
GO:0006644	biological_process	phospholipid metabolic process	5	6	37	12977	362	2.53933253	0.002568433
GO:0006664	biological_process	glycolipid metabolic process	6	4	17	12977	362	3.076360553	0.004706451
GO:0008654	biological_process	phospholipid biosynthetic process	6	6	35	12977	362	2.619502878	0.001958677
GO:0009245	biological_process	lipid A biosynthetic process	8	4	17	12977	362	3.076360553	0.004706451
GO:0009247	biological_process	glycolipid biosynthetic process	7	4	17	12977	362	3.076360553	0.004706451
GO:0009311	biological_process	oligosaccharide metabolic process	5	4	26	12977	362	2.463383677	0.018505789
GO:0009312	biological_process	oligosaccharide biosynthetic process	6	4	24	12977	362	2.578860894	0.014523367
GO:0046467	biological_process	membrane lipid biosynthetic process	6	4	18	12977	362	2.993898393	0.005699908

GO:0046493	biological_process	lipid A metabolic process	7	4	17	12977	362	3.076360553	0.004706451
GO:1901269	biological_process	lipooligosaccharide metabolic process	6	4	17	12977	362	3.076360553	0.004706451
GO:1901271	biological_process	lipooligosaccharide biosynthetic process	7	4	17	12977	362	3.076360553	0.004706451
GO:0006163	biological_process	purine nucleotide metabolic process	8	9	92	12977	362	1.81018644	0.004706451
GO:0006164	biological_process	purine nucleotide biosynthetic process	9	8	67	12977	362	2.097734204	0.002645845
GO:0009150	biological_process	purine ribonucleotide metabolic process	9	8	88	12977	362	1.704391776	0.011692597
GO:0009152	biological_process	purine ribonucleotide biosynthetic process	10	8	66	12977	362	2.119429275	0.002444193
GO:0009259	biological_process	ribonucleotide metabolic process	8	14	109	12977	362	2.202993992	1.90E-05

GO:0009260	biological_process	ribonucleotide biosynthetic process	9	14	87	12977	362	2.528234821	1.37E-06
GO:0015936	biological_process	coenzyme A metabolic process	10	4	13	12977	362	3.463383677	0.00186796
GO:0015937	biological_process	coenzyme A biosynthetic process	11	4	8	12977	362	4.163823395	0.000287995
GO:0019693	biological_process	ribose phosphate metabolic process	5	15	111	12977	362	2.276298124	4.65E-06
GO:0033865	biological_process	nucleoside bisphosphate metabolic process	7	4	22	12977	362	2.704391776	0.011198823
GO:0033866	biological_process	nucleoside bisphosphate biosynthetic process	8	4	8	12977	362	4.163823395	0.000287995
GO:0033875	biological_process	ribonucleoside bisphosphate metabolic process	8	4	22	12977	362	2.704391776	0.011198823

GO:0034030	biological_process	ribonucleoside bisphosphate biosynthetic process	9	4	8	12977	362	4.163823395	0.000287995
GO:0034032	biological_process	purine nucleoside bisphosphate metabolic process	8	4	22	12977	362	2.704391776	0.011198823
GO:0034033	biological_process	purine nucleoside bisphosphate biosynthetic process	9	4	8	12977	362	4.163823395	0.000287995
GO:0042278	biological_process	purine nucleoside metabolic process	7	10	75	12977	362	2.256932799	0.000297919
GO:0042451	biological_process	purine nucleoside biosynthetic process	8	8	51	12977	362	2.491398053	0.000507991
GO:0046128	biological_process	purine ribonucleoside metabolic process	8	10	75	12977	362	2.256932799	0.000297919

GO:0046129	biological_process	purine ribonucleoside biosynthetic process	9	8	51	12977	362	2.491398053	0.000507991
GO:0046390	biological_process	ribose phosphate biosynthetic process	6	15	89	12977	362	2.594980559	2.68E-07
GO:0072521	biological_process	purine-containing compound metabolic process	4	11	104	12977	362	1.922815295	0.000870139
GO:0072522	biological_process	purine-containing compound biosynthetic process	5	8	73	12977	362	1.973998836	0.004310523
GO:0006119	biological_process	oxidative phosphorylation	6	3	12	12977	362	3.163823395	0.014168081
GO:0015980	biological_process	energy derivation by oxidation of organic compounds	4	6	51	12977	362	2.076360553	0.010929177
GO:0022900	biological_process	electron transport chain	4	4	24	12977	362	2.578860894	0.014523367

GO:0022904	biological_process	respiratory electron transport chain	6	3	18	12977	362	2.578860894	0.039261507
GO:0042773	biological_process	ATP synthesis coupled electron transport	7	3	12	12977	362	3.163823395	0.014168081
GO:0045333	biological_process	cellular respiration	5	6	50	12977	362	2.104929706	0.009954243
GO:0003954	molecular_function	NADH dehydrogenase activity	4	4	17	12977	362	3.076360553	0.004706451
GO:0008137	molecular_function	NADH dehydrogenase (ubiquinone) activity	6	3	13	12977	362	3.048346177	0.017018983
GO:0016651	molecular_function	oxidoreductase activity, acting on NAD(P)H	3	5	43	12977	362	2.059486735	0.021901628
GO:0016655	molecular_function	oxidoreductase activity, acting on NAD(P)H, quinone or similar compound as acceptor	4	4	22	12977	362	2.704391776	0.011198823

GO:0050136	molecular_function	NADH dehydrogenase (quinone) activity	5	4	17	12977	362	3.076360553	0.004706451
GO:0006457	biological_process	protein folding	6	4	29	12977	362	2.3058424	0.02619071
GO:0006508	biological_process	proteolysis	5	13	208	12977	362	1.163823395	0.018958065
GO:0004175	molecular_function	endopeptidase activity	5	8	83	12977	362	1.788783963	0.009042664
GO:0008233	molecular_function	peptidase activity	3	13	203	12977	362	1.198927196	0.015796009
GO:0070011	molecular_function	peptidase activity, acting on L-amino acid peptides	4	12	177	12977	362	1.281180345	0.014523367
GO:0009055	molecular_function	electron carrier activity	1	7	58	12977	362	2.113197322	0.004750891
GO:0015035	molecular_function	protein disulfide oxidoreductase activity	5	3	12	12977	362	3.163823395	0.014168081
GO:0015036	molecular_function	disulfide oxidoreductase activity	4	3	14	12977	362	2.941430973	0.020857606

GO:0016667	molecular_function	oxidoreductase activity, acting on a sulfur group of donors	3	5	32	12977	362	2.48575149	0.007472922
GO:0005525	molecular_function	GTP binding	8	9	60	12977	362	2.4268578	0.000287995
GO:0019001	molecular_function	guanyl nucleotide binding	6	9	65	12977	362	2.311380583	0.000504933
GO:0032561	molecular_function	guanyl ribonucleotide binding	7	9	65	12977	362	2.311380583	0.000504933
GO:0003924	molecular_function	GTPase activity	7	6	30	12977	362	2.8418953	0.000880089
GO:0000105	biological_process	histidine biosynthetic process	10	3	15	12977	362	2.8418953	0.024759823
GO:0008652	biological_process	cellular amino acid biosynthetic process	8	20	135	12977	362	2.408935892	1.42E-08
GO:0009076	biological_process	histidine family amino acid biosynthetic process	9	3	15	12977	362	2.8418953	0.024759823
GO:0016053	biological_process	organic acid biosynthetic process	5	24	194	12977	362	2.148873053	1.33E-08

GO:0046394	biological_process	carboxylic acid biosynthetic process	7	24	194	12977	362	2.148873053	1.33E-08
GO:1901605	biological_process	alpha-amino acid metabolic process	8	23	171	12977	362	2.269532836	6.09E-09
GO:1901607	biological_process	alpha-amino acid biosynthetic process	9	16	98	12977	362	2.549113551	1.48E-07
GO:0008171	molecular_function	O-methyltransferase activity	5	4	20	12977	362	2.8418953	0.008478901
GO:0009063	biological_process	cellular amino acid catabolic process	8	7	44	12977	362	2.511746698	0.001105486
GO:0016054	biological_process	organic acid catabolic process	5	10	57	12977	362	2.652861475	3.18E-05
GO:0043648	biological_process	dicarboxylic acid metabolic process	7	11	68	12977	362	2.535792172	2.42E-05
GO:0044282	biological_process	small molecule catabolic process	4	11	66	12977	362	2.578860894	1.82E-05

GO:0044712	biological_process	single-organism catabolic process	3	11	66	12977	362	2.578860894	1.82E-05
GO:0046395	biological_process	carboxylic acid catabolic process	7	10	57	12977	362	2.652861475	3.18E-05
GO:1901565	biological_process	organonitrogen compound catabolic process	4	7	69	12977	362	1.86265386	0.011428985
GO:1901606	biological_process	alpha-amino acid catabolic process	9	6	42	12977	362	2.356468473	0.004627518
GO:0042398	biological_process	cellular modified amino acid biosynthetic process	9	4	31	12977	362	2.209627084	0.032911673
GO:0006760	biological_process	folic acid-containing compound metabolic process	9	3	12	12977	362	3.163823395	0.014168081
GO:0042558	biological_process	pteridine-containing compound metabolic process	4	5	15	12977	362	3.578860894	0.000287995

GO:0042559	biological_process	pteridine-containing compound biosynthetic process	5	4	10	12977	362	3.8418953	0.000672388
GO:0043650	biological_process	dicarboxylic acid biosynthetic process	8	6	27	12977	362	2.993898393	0.000535905
GO:0003735	molecular_function	structural constituent of ribosome	2	6	58	12977	362	1.8908049	0.018277463
GO:0005198	molecular_function	structural molecule activity	1	6	68	12977	362	1.661323054	0.035463766
GO:0019843	molecular_function	rRNA binding	5	5	43	12977	362	2.059486735	0.021901628
GO:0030529	cellular_component	ribonucleoprotein complex	5	7	73	12977	362	1.781353758	0.014794781
GO:0005840	cellular_component	ribosome	7	7	71	12977	362	1.821431197	0.013097886
GO:0032787	biological_process	monocarboxylic acid metabolic process	7	9	79	12977	362	2.029967648	0.001847115
GO:0006778	biological_process	porphyrin-containing compound metabolic process	5	5	32	12977	362	2.48575149	0.007472922

GO:0006779	biological_process	porphyrin-containing compound biosynthetic process	6	4	29	12977	362	2.3058424	0.02619071
GO:0033013	biological_process	tetrapyrrole metabolic process	4	5	34	12977	362	2.398288648	0.009300588
GO:0033014	biological_process	tetrapyrrole biosynthetic process	5	4	31	12977	362	2.209627084	0.032911673
GO:0016765	molecular_function	transferase activity, transferring alkyl or aryl (other than methyl) groups	3	6	39	12977	362	2.463383677	0.003294102
GO:0016226	biological_process	iron-sulfur cluster assembly	5	2	6	12977	362	3.578860894	0.033559805
GO:0031163	biological_process	metallo-sulfur cluster assembly	4	2	6	12977	362	3.578860894	0.033559805
GO:0008237	molecular_function	metallopeptidase activity	5	9	66	12977	362	2.289354277	0.000551581
GO:0004222	molecular_function	metalloendopeptidase activity	6	7	35	12977	362	2.8418953	0.000299634

GO:0016453	molecular_function	C-acetyltransferase activity	6	2	5	12977	362	3.8418953	0.023977261
GO:0070475	biological_process	rRNA base methylation	12	2	7	12977	362	3.356468473	0.04357665
GO:0009062	biological_process	fatty acid catabolic process	9	3	11	12977	362	3.289354277	0.011383731
GO:0016042	biological_process	lipid catabolic process	4	4	14	12977	362	3.356468473	0.002444193
GO:0044242	biological_process	cellular lipid catabolic process	5	4	12	12977	362	3.578860894	0.001364494
GO:0072329	biological_process	monocarboxylic acid catabolic process	8	3	12	12977	362	3.163823395	0.014168081
GO:0016829	molecular_function	lyase activity	2	22	213	12977	362	1.888545393	1.47E-06
GO:0016835	molecular_function	carbon-oxygen lyase activity	3	12	72	12977	362	2.578860894	6.55E-06
GO:0016836	molecular_function	hydro-lyase activity	4	8	52	12977	362	2.463383677	0.000564158
GO:0016830	molecular_function	carbon-carbon lyase activity	3	7	67	12977	362	1.905089126	0.00997249
GO:0016831	molecular_function	carboxy-lyase activity	4	6	38	12977	362	2.500858382	0.002915011

GO:0019877	biological_process	diaminopimelate biosynthetic process	9	2	5	12977	362	3.8418953	0.023977261
GO:0046451	biological_process	diaminopimelate metabolic process	8	3	13	12977	362	3.048346177	0.017018983
GO:0006553	biological_process	lysine metabolic process	9	3	14	12977	362	2.941430973	0.020857606
GO:0009066	biological_process	aspartate family amino acid metabolic process	8	5	32	12977	362	2.48575149	0.007472922
GO:0009085	biological_process	lysine biosynthetic process	10	3	13	12977	362	3.048346177	0.017018983
GO:0009089	biological_process	lysine biosynthetic process via diaminopimelate	11	3	13	12977	362	3.048346177	0.017018983
GO:0006563	biological_process	L-serine metabolic process	9	2	7	12977	362	3.356468473	0.04357665
GO:0009069	biological_process	serine family amino acid metabolic process	8	6	21	12977	362	3.356468473	0.000145834

GO:0009070	biological_process	serine family amino acid biosynthetic process	9	3	11	12977	362	3.289354277	0.011383731
GO:0051287	molecular_function	NAD binding	5	5	41	12977	362	2.128199485	0.018277463
GO:0016614	molecular_function	oxidoreductase activity, acting on CH-OH group of donors	3	12	139	12977	362	1.629844823	0.002595245
GO:0016616	molecular_function	oxidoreductase activity, acting on the CH-OH group of donors, NAD or NADP as acceptor	4	11	120	12977	362	1.716364418	0.002645845
GO:0006635	biological_process	fatty acid beta-oxidation	10	2	7	12977	362	3.356468473	0.04357665
GO:0019395	biological_process	fatty acid oxidation	9	2	7	12977	362	3.356468473	0.04357665
GO:0034440	biological_process	lipid oxidation	6	2	7	12977	362	3.356468473	0.04357665
GO:0030170	molecular_function	pyridoxal phosphate binding	4	9	70	12977	362	2.204465379	0.000802625
GO:0003690	molecular_function	double-stranded DNA binding	6	2	6	12977	362	3.578860894	0.033559805

GO:0006298	biological_process	mismatch repair	8	3	5	12977	362	4.4268578	0.001155324
GO:0007059	biological_process	chromosome segregation	3	3	12	12977	362	3.163823395	0.014168081
GO:0042168	biological_process	heme metabolic process	6	4	24	12977	362	2.578860894	0.014523367
GO:0042440	biological_process	pigment metabolic process	3	4	33	12977	362	2.119429275	0.039261507
GO:0006820	biological_process	anion transport	6	6	74	12977	362	1.53933253	0.049748417
GO:0009123	biological_process	nucleoside monophosphate metabolic process	7	8	47	12977	362	2.609234543	0.000297919
GO:0009124	biological_process	nucleoside monophosphate biosynthetic process	8	8	46	12977	362	2.640261439	0.000281514
GO:0009156	biological_process	ribonucleoside monophosphate biosynthetic process	9	8	42	12977	362	2.771505972	0.000146309

GO:0009161	biological_process	ribonucleoside monophosphate metabolic process	8	8	42	12977	362	2.771505972	0.000146309
GO:0015935	cellular_component	small ribosomal subunit	9	2	7	12977	362	3.356468473	0.04357665
GO:0000162	biological_process	tryptophan biosynthetic process	10	2	7	12977	362	3.356468473	0.04357665
GO:0009072	biological_process	aromatic amino acid family metabolic process	8	7	39	12977	362	2.685776098	0.000567618
GO:0009073	biological_process	aromatic amino acid family biosynthetic process	9	5	25	12977	362	2.8418953	0.002757952
GO:0042435	biological_process	indole-containing compound biosynthetic process	5	2	7	12977	362	3.356468473	0.04357665
GO:0046219	biological_process	indolalkylamine biosynthetic process	8	2	7	12977	362	3.356468473	0.04357665

GO:0006213	biological_process	pyrimidine nucleoside metabolic process	7	5	23	12977	362	2.962189533	0.001948683
GO:0006220	biological_process	pyrimidine nucleotide metabolic process	8	6	26	12977	362	3.048346177	0.000448101
GO:0006221	biological_process	pyrimidine nucleotide biosynthetic process	9	6	24	12977	362	3.163823395	0.000290613
GO:0006222	biological_process	UMP biosynthetic process	11	5	15	12977	362	3.578860894	0.000287995
GO:0009129	biological_process	pyrimidine nucleoside monophosphate metabolic process	8	5	18	12977	362	3.315826488	0.000649374
GO:0009130	biological_process	pyrimidine nucleoside monophosphate biosynthetic process	9	5	18	12977	362	3.315826488	0.000649374

GO:0009173	biological_process	pyrimidine ribonucleoside monophosphate metabolic process	9	5	15	12977	362	3.578860894	0.000287995
GO:0009174	biological_process	pyrimidine ribonucleoside monophosphate biosynthetic process	10	5	15	12977	362	3.578860894	0.000287995
GO:0009218	biological_process	pyrimidine ribonucleotide metabolic process	9	5	19	12977	362	3.237823976	0.000813603
GO:0009220	biological_process	pyrimidine ribonucleotide biosynthetic process	10	5	19	12977	362	3.237823976	0.000813603
GO:0044205	biological_process	'de novo' UMP biosynthetic process	12	5	8	12977	362	4.48575149	8.66E-06
GO:0046049	biological_process	UMP metabolic process	10	5	15	12977	362	3.578860894	0.000287995

GO:0046131	biological_process	pyrimidine ribonucleoside metabolic process	8	5	20	12977	362	3.163823395	0.001033749
GO:0046132	biological_process	pyrimidine ribonucleoside biosynthetic process	9	5	18	12977	362	3.315826488	0.000649374
GO:0046134	biological_process	pyrimidine nucleoside biosynthetic process	8	5	18	12977	362	3.315826488	0.000649374
GO:0006206	biological_process	pyrimidine nucleobase metabolic process	7	4	10	12977	362	3.8418953	0.000672388
GO:0006207	biological_process	'de novo' pyrimidine nucleobase biosynthetic process	9	3	5	12977	362	4.4268578	0.001155324
GO:0009112	biological_process	nucleobase metabolic process	6	4	18	12977	362	2.993898393	0.005699908
GO:0019856	biological_process	pyrimidine nucleobase biosynthetic process	8	4	6	12977	362	4.578860894	7.38E-05

GO:0046112	biological_process	nucleobase biosynthetic process	7	4	11	12977	362	3.704391776	0.00096882
GO:0006650	biological_process	glycerophospholipid metabolic process	6	2	7	12977	362	3.356468473	0.04357665
GO:0046486	biological_process	glycerolipid metabolic process	5	2	7	12977	362	3.356468473	0.04357665
GO:0046434	biological_process	organophosphate catabolic process	5	4	13	12977	362	3.463383677	0.00186796
GO:0006544	biological_process	glycine metabolic process	9	3	7	12977	362	3.941430973	0.003303069
GO:1901292	biological_process	nucleoside phosphate catabolic process	7	3	8	12977	362	3.748785895	0.004734987
GO:0016878	molecular_function	acid-thiol ligase activity	4	2	6	12977	362	3.578860894	0.033559805
GO:0046653	biological_process	tetrahydrofolate metabolic process	10	2	7	12977	362	3.356468473	0.04357665
GO:0003989	molecular_function	acetyl-CoA carboxylase activity	5	2	5	12977	362	3.8418953	0.023977261

GO:0016421	molecular_function	CoA carboxylase activity	4	2	7	12977	362	3.356468473	0.04357665
GO:0006072	biological_process	glycerol-3-phosphate metabolic process	6	2	7	12977	362	3.356468473	0.04357665
GO:0052646	biological_process	alditol phosphate metabolic process	5	2	7	12977	362	3.356468473	0.04357665
GO:0006071	biological_process	glycerol metabolic process	7	2	5	12977	362	3.8418953	0.023977261
GO:0019400	biological_process	alditol metabolic process	6	2	5	12977	362	3.8418953	0.023977261
GO:1901615	biological_process	organic hydroxy compound metabolic process	3	3	15	12977	362	2.8418953	0.024759823
GO:0019692	biological_process	deoxyribose phosphate metabolic process	5	2	6	12977	362	3.578860894	0.033559805
GO:0009166	biological_process	nucleotide catabolic process	8	2	6	12977	362	3.578860894	0.033559805

GO:0009262	biological_process	deoxyribonucleotide metabolic process	8	3	7	12977	362	3.941430973	0.003303069
GO:0006108	biological_process	malate metabolic process	8	2	7	12977	362	3.356468473	0.04357665

Supplementary Table 4e: Core gene clusters predicted to be under positive selection among the Clade 3 strains compared to the rest of Legionella Clades

Cluster No.	**Cluster representative internal Gene id**	**GI Accession Id**	**LOCUS Accession id**	**Gene Annotation**	**InL_H0**	**InL_Ha**	**Ha-H0**	**2*(Ha-H0)**	**Chi p-values**	**p-value**	**q-value**	**signiica[illegible]**
199	Anisa00068	671587667	WP_031563838.1	MFS transporter [Legionella wadsworthii]	-12816.13744	-12811.13271	5.00473	10.00946	0.001557382	0.001557382	0.01994849	1
200	Anisa00071	654930767	WP_028381135.1	DNA topoisomerase I [Legionella cherrii]	-11019.42428	-11015.33649	4.087793	8.175586	0.004245795	0.004245795	0.0345729	1
210	Anisa00089	654930785	WP_028381153.1	molecular chaperone GroEL [Legionella cherrii]	-11071.37064	-11067.26712	4.103513	8.207026	0.004172849	0.004172849	0.03448645	1
213	Anisa00097	654930788	WP_028381156.1	ribosomal protein S12 methylthiotransferase [Legionella	-12759.811	-12756.18922	3.621782	7.243564	0.00711556	0.0071556	0.04611212	1

				cherrii]								
218	Anisa00111	654930800	WP_028381168.1	glycerophosphodiester phosphodiesterase [Legionella cherrii]	-7688.462448	-7682.903766	5.558682	11.117364	0.000855232	0.000855233	0.01299954	1
223	Anisa00116	654930806	WP_028381174.1	2-keto-4-pentenoate hydratase [Legionella cherrii]	-10797.07008	-10786.27399	10.796095	21.59219	3.37222E-06	0.00000337	0.000256289	1
233	Anisa00134	654930821	WP_028381189.1	ankyrin [Legionella cherrii]	-15211.11199	-15207.00887	4.103116	8.206232	0.004174675	0.004174676	0.03448645	1
246	Anisa00153	498338554	WP_010652710.1	geranyltranstransferase [Fluoribacter dumoffii]	-11236.22226	-11224.7888	11.433466	22.866932	1.73613E-06	0.00000174	0.000143878	1
248	Anisa00155	502743836	WP_012978820.1	competence protein ComF [Legionella longbeachae]	-7934.981424	-7931.322167	3.659257	7.318514	0.006824782	0.006824782	0.04541557	1
249	Anisa00157	671586084	WP_031562600.1	peptidase [Legionella wadsworthii]	-10789.81273	-10780.58479	9.22794	18.45588	1.73883E-05	0.0000174	0.000792907	1
259	Anisa00174	654930855	WP_028381223.1	2,4-dienoyl-CoA reductase [Legionella cherrii]	-21030.76024	-21013.59754	17.162706	34.325412	4.66254E-09	4.66E-09	0.000000664	1
262	Anisa00179	671586124	WP_031562633.1	hypothetical protein, partial [Legionella	-8845.05165	-8841.0264	4.025214	8.050428	0.004549291	0.004549291	0.03576684	1

				wadsworthii]		36						
266	Anisa0 0183	654930865	WP_028381233.1	DNA primase [Legionella cherrii]	- 11319. 83701	- 1131 5.192 71	4.64 4302	9.2886 04	0.002305838	0.0023 05838	0.02367 333	1
270	Anisa0 0209	498339594	WP_010653750.1	mechanosensitive ion channel protein MscS [Fluoribacter dumoffii]	- 16896. 91878	- 1689 2.912 27	4.00 6503	8.0130 06	0.004644258	0.0046 44258	0.03626 338	1
274	Anisa0 0218	654930131	WP_028380501.1	oxidoreductase [Legionella cherrii]	- 9553.7 87325	- 9549. 5783 78	4.20 8947	8.4178 94	0.003715459	0.0037 15459	0.03233 3	1
276	Anisa0 0224	654930124	WP_028380494.1	integrase [Legionella cherrii]	- 20600. 94698	- 2059 6.124 29	4.82 2694	9.6453 88	0.001898276	0.0018 98276	0.02164 035	1
286	Anisa0 0240	654930108	WP_028380478.1	elongation factor Ts [Legionella cherrii]	- 7950.3 44248	- 7946. 7902 73	3.55 3975	7.1079 5	0.007674282	0.0076 74283	0.04788 042	1
291	Anisa0 0249	498339633	WP_010653789.1	ATP-dependent dethiobiotin synthetase BioD [Fluoribacter dumoffii]	- 8304.7 33954	- 8301. 0687 04	3.66 525	7.3305	0.006779419	0.0067 79419	0.04541 557	1
293	Anisa0 0251	654930098	WP_028380468.1	8-amino-7-oxononanoate synthase [Legionella cherrii]	- 14040. 19431	- 1403 5.557 9	4.63 6407	9.2728 14	0.002325801	0.0023 25801	0.02367 333	1

295	Anisa00253	657688536	WP_029488987.1	adenosylmethionine--8-amino-7-oxononanoate aminotransferase BioA [Fluoribacter dumoffii]	-15898.76616	-15890.7041	8.062053	16.124106	5.93241E-05	0.0000593	0.002049378	1
301	Anisa00263	654930084	WP_028380454.1	hypothetical protein [Legionella cherrii]	-8587.659326	-8580.158541	7.500785	15.00157	0.000107422	0.000107422	0.003061527	1
304	Anisa00266	654930081	WP_028380451.1	(p)ppGpp synthetase [Legionella cherrii]	-13228.4749	-13223.6791	4.795801	9.591602	0.001954693	0.001954693	0.02206287	1
307	Anisa00271	654930077	WP_028380447.1	dihydrolipoamide acetyltransferase [Legionella cherrii]	-19392.49748	-19386.98755	5.509929	11.019858	0.00090141	0.00090141	0.01321025	1
317	Anisa00284	654930064	WP_028380434.1	SMC-Scp complex subunit ScpB [Legionella cherrii]	-6818.832497	-6813.961697	4.8708	9.7416	0.00180144	0.00180144	0.02137073	1
327	Anisa00296	498339680	WP_010653836.1	purine nucleoside phosphorylase [Fluoribacter dumoffii]	-8890.794531	-8887.25735	3.537181	7.074362	0.007819466	0.007819466	0.04792576	1
339	Anisa00314	654930038	WP_028380408.1	DNA gyrase subunit A [Legionella cherrii]	-23650.7055	-23646.06539	4.64011	9.28022	0.002316416	0.002316416	0.02367333	1
349	Anisa00329	654930022	WP_028380392.1	anhydro-N-acetylmuramic acid kinase [Legionella	-14472.02139	-14468.131	3.890262	7.780524	0.005281248	0.005281248	0.03935048	1

				cherrii]		13						
352	Anisa00333	654930018	WP_028380388.1	thymidylate kinase [Legionella cherrii]	-5024.393889	-5020.782269	3.61162	7.22324	0.00719657	0.00719657	0.04611212	1
364	Anisa00345	653017774	WP_027269737.1	endopeptidase IV [Legionella sainthelensi]	-5757.69772	-5753.146016	4.551704	9.103408	0.002551337	0.002551337	0.02529151	1
369	Anisa00351	654929718	WP_028380088.1	hypothetical protein [Legionella cherrii]	-4104.992879	-4101.372967	3.619912	7.239824	0.007130397	0.007130397	0.04611212	1
374	Anisa00357	654929725	WP_028380095.1	phosphoenolpyruvate synthase [Legionella cherrii]	-21355.06139	-21349.05226	6.009131	12.018262	0.000526818	0.000526818	0.009383946	1
378	Anisa00361	654929729	WP_028380099.1	peptidase M20 [Legionella cherrii]	-15881.54041	-15877.20225	4.33816	8.67632	0.003223713	0.003223713	0.02893727	1
394	Anisa00385	654929748	WP_028380118.1	anthranilate phosphoribosyltransferase [Legionella cherrii]	-12948.54922	-12944.85819	3.691022	7.382044	0.006587826	0.006587826	0.04451803	1
408	Anisa00402	654929764	WP_028380134.1	hypothetical protein [Legionella cherrii]	-5922.904447	-5917.964667	4.93978	9.87956	0.001671249	0.001671249	0.02070965	1
410	Anisa00407	654929768	WP_028380138.1	cytochrome C biogenesis protein CcmA [Legionella	-8010.804175	-8003.1466	7.657533	15.315066	9.09879E-05	0.000091	0.002729637	1

				cherrii]		42						
415	Anisa0 0413	654929774	WP_028380144.1	thiol:disulfide interchange protein [Legionella cherrii]	- 6801.2 87466	- 6791. 4790 25	9.80 8441	19.616 882	9.46294E-06	0.0000 0946	0.00049 0352	1
422	Anisa0 0425	654929788	WP_028380158.1	coiled-coil protein [Legionella cherrii]	- 41014. 14215	- 4100 8.192 54	5.94 9611	11.899 222	0.000561574	0.0005 61574	0.00969 9915	1
431	Anisa0 0446	654929808	WP_028380178.1	DNA polymerase III subunit alpha [Legionella cherrii]	- 19918. 36308	- 1991 2.869 2	5.49 3881	10.987 762	0.000917155	0.0009 17155	0.01322 367	1
436	Anisa0 0452	654929817	WP_028380187.1	membrane protein [Legionella cherrii]	- 13568. 34259	- 1356 4.290 12	4.05 2476	8.1049 52	0.004414449	0.0044 14449	0.03519 211	1
439	Anisa0 0500	654930303	WP_028380673.1	crotonase [Legionella cherrii]	- 15198. 55811	- 1519 3.644 24	4.91 387	9.8277 4	0.001718994	0.0017 18994	0.02071 999	1
447	Anisa0 0514	498339452	WP_010653608.1	glycine cleavage system protein T [Fluoribacter dumoffii]	- 14265. 04017	- 1426 0.860 27	4.17 9906	8.3598 12	0.003836103	0.0038 36103	0.03312 998	1
462	Anisa0 0540	654930269	WP_028380639.1	hypothetical protein [Legionella cherrii]	- 6095.6 44081	- 6088. 6829 4	6.96 1141	13.922 282	0.000190526	0.0001 90526	0.00452 4992	1
483	Anisa0 0566	654930241	WP_028380611.1	hypothetical protein [Legionella cherrii]	- 4090.2 72422	- 4085. 3626	4.90 9775	9.8195 5	0.001726666	0.0017 26666	0.02071 999	1

						47						
490	Anisa00620	671590076	WP_031565795.1	hypoxanthine-guanine phosphoribosyltransferase [Legionella wadsworthii]	-5633.824995	-5615.535539	18.289456	36.578912	1.46609E-09	1.47E-09	0.000000279	1
492	Anisa00629	654930177	WP_028380547.1	alpha-2-macroglobulin [Legionella cherrii]	-55628.71052	-55623.17623	5.534288	11.068576	0.000878031	0.000878031	0.01317046	1
497	Anisa00660	498340272	WP_010654428.1	molecular chaperone DnaJ [Fluoribacter dumoffii]	-10223.6327	-10219.18261	4.450096	8.900192	0.002851407	0.002851407	0.02664429	1
501	Anisa00672	654929494	WP_028379864.1	uroporphyrinogen decarboxylase [Legionella cherrii]	-12303.894	-12300.40058	3.493426	6.986852	0.008211064	0.008211065	0.04952706	1
504	Anisa00676	654929489	WP_028379859.1	ATP-dependent DNA helicase RecG [Legionella cherrii]	-21839.97866	-21833.45427	6.524391	13.048782	0.000303482	0.000303482	0.006290354	1
508	Anisa00686	498340290	WP_010654446.1	hypothetical protein [Fluoribacter dumoffii]	-7157.294637	-7150.270715	7.023922	14.047844	0.000178218	0.000178218	0.004387981	1
509	Anisa00687	654929479	WP_028379849.1	endonuclease [Legionella cherrii]	-35789.06854	-35778.57211	10.496435	20.99287	4.60996E-06	0.00000461	0.000291964	1
510	Anisa00689	671588473	WP_031564458.1	membrane protein [Legionella wadsworthii]	-7909.64853	-7900.8579	8.790556	17.581112	2.75309E-05	0.0000275	0.001082249	1

						74						
511	Anisa00690	498340294	WP_010654450.1	tyrosine-specific transporter [Fluoribacter dumoffii]	-7615.577528	-7610.992603	4.584925	9.16985	0.002460347	0.002460347	0.0247225	1
512	Anisa00691	498340295	WP_010654451.1	tyrosine-specific transporter [Fluoribacter dumoffii]	-9117.587619	-9111.726822	5.860797	11.721594	0.00061779	0.00061779	0.01049353	1
519	Anisa00737	654929437	WP_028379807.1	DNA-(apurinic or apyrimidinic site) lyase [Legionella cherrii]	-8080.150719	-8073.536934	6.613785	13.22757	0.000275861	0.000275861	0.005823732	1
520	Anisa00738	671588382	WP_031564381.1	NAD(P)H-quinone oxidoreductase [Legionella wadsworthii]	-6870.03917	-6866.476966	3.562204	7.124408	0.007604152	0.007604152	0.04788042	1
537	Anisa00791	654931512	WP_028381875.1	DNA polymerase III subunit delta [Legionella cherrii]	-7985.430392	-7981.2764	4.153992	8.307984	0.003947118	0.003947118	0.03383244	1
539	Anisa00793	654931510	WP_028381873.1	leucyl-tRNA synthetase [Legionella cherrii]	-18896.00484	-18889.50304	6.501805	13.00361	0.000310891	0.000310891	0.006328852	1
545	Anisa00805	498339310	WP_010653466.1	cell division protein FtsK [Fluoribacter dumoffii]	-25123.71995	-25120.13058	3.589369	7.178738	0.007377258	0.007377258	0.04698365	1
574	Anisa00857	654931446	WP_028381809.1	GMP synthase [Legionella cherrii]	-14883.55592	-14875.85762	7.698298	15.396596	8.71451E-05	0.0000871	0.002729637	1

581	Anisa00894	498341133	WP_010655289.1	F0F1 ATP synthase subunit delta [Fluoribacter dumoffii]	-5230.756998	-5227.209282	3.547716	7.095432	0.007728066	0.007728067	0.04788042	1
586	Anisa00901	654931092	WP_028381459.1	hemolysin [Legionella cherrii]	-6469.676518	-6465.829069	3.847449	7.694898	0.005537714	0.005537714	0.04013312	1
604	Anisa00925	671591284	WP_031566795.1	thiol-disulfide oxidoreductase [Legionella wadsworthii]	-5667.962964	-5664.21722	3.745744	7.491488	0.006199131	0.006199132	0.04257235	1
612	Anisa00945	671591317	WP_031566823.1	2-octaprenyl-3-methyl-6-methoxy-1,4-benzoquinol hydroxylase [Legionella wadsworthii]	-5596.187066	-5592.131617	4.055449	8.110898	0.004399992	0.004399993	0.03519211	1
618	Anisa00954	498338008	WP_010652164.1	dihydroorotase [Fluoribacter dumoffii]	-14555.69701	-14546.25858	9.43843	18.87686	1.39434E-05	0.0000139	0.000662312	1
627	Anisa00972	654932027	WP_028382389.1	fumarate hydratase [Legionella cherrii]	-13921.66189	-13918.09844	3.563446	7.126892	0.007593624	0.007593624	0.04788042	1
637	Anisa00985	671591406	WP_031566897.1	helicase [Legionella wadsworthii]	-15525.92287	-15521.80751	4.115357	8.230714	0.004118732	0.004118732	0.03448645	1
638	Anisa00986	654932041	WP_028382403.1	diadenosine tetraphosphatase [Legionella cherrii]	-9496.324481	-9488.4648	7.859604	15.719208	7.34743E-05	0.0000735	0.00246355	1

						77						
649	Anisa0<br>1020	654930661	WP_028381030.1	hypothetical protein<br>[Legionella cherrii]	-<br>11180.<br>08952	-<br>1117<br>2.639<br>53	7.44<br>9986	14.899<br>972	0.000113365	0.0001<br>13365	0.00315<br>1313	1
656	Anisa0<br>1032	654930652	WP_028381021.1	peptidase<br>[Legionella cherrii]	-<br>15934.<br>05283	-<br>1593<br>0.078<br>19	3.97<br>4649	7.9492<br>98	0.004810603	0.0048<br>10604	0.03680<br>596	1
664	Anisa0<br>1045	654930638	WP_028381007.1	ankyrin [Legionella<br>cherrii]	-<br>14330.<br>83735	-<br>1432<br>6.914<br>09	3.92<br>3253	7.8465<br>06	0.005091902	0.0050<br>91903	0.03818<br>927	1
672	Anisa0<br>1055	654930629	WP_028380998.1	phenol hydroxylase<br>[Legionella cherrii]	-<br>7571.5<br>20431	-<br>7567.<br>0986<br>75	4.42<br>1756	8.8435<br>12	0.002941326	0.0029<br>41326	0.02704<br>122	1
676	Anisa0<br>1059	654930624	WP_028380993.1	zinc protease<br>[Legionella cherrii]	-<br>17500.<br>34657	-<br>1747<br>8.470<br>08	21.8<br>7648<br>7	43.752<br>974	3.7255E-11	3.73E-<br>11	8.49E-<br>09	1
686	Anisa0<br>1076	654930607	WP_028380976.1	ATP-dependent<br>protease<br>[Legionella cherrii]	-<br>18241.<br>74734	-<br>1823<br>7.294<br>83	4.45<br>2504	8.9050<br>08	0.002843896	0.0028<br>43896	0.02664<br>429	1
696	Anisa0<br>1089	654930593	WP_028380962.1	metallopeptidase<br>[Legionella cherrii]	-<br>19382.<br>6048	-<br>1934<br>2.322<br>82	40.2<br>8198<br>2	80.563<br>964	2.81446E-19	2.81E-<br>19	1.6E-16	1
699	Anisa0<br>1092	654930590	WP_028380959.1	cell division protein<br>FtsA [Legionella<br>cherrii]	-<br>10513.<br>2968	-<br>1048<br>8.568	24.7<br>2783<br>9	49.455<br>678	2.02904E-12	2.03E-<br>12	5.78E-<br>10	1

						96						
701	Anisa01094	654930588	WP_028380957.1	D-alanine--D-alanine ligase [Legionella cherrii]	-11427.85726	-11423.74417	4.11309	8.22618	0.004129035	0.004129035	0.03448645	1
712	Anisa01111	654931148	WP_028381515.1	excinuclease ABC subunit B [Legionella cherrii]	-11417.24715	-11412.94144	4.305708	8.611416	0.003340625	0.003340625	0.02966108	1
717	Anisa01121	654931154	WP_028381521.1	2-octaprenyl-6-methoxyphenyl hydroxylase [Legionella cherrii]	-14303.26225	-14297.77924	5.483012	10.966024	0.000927977	0.000927977	0.01322367	1
719	Anisa01127	498341073	WP_010655229.1	alpha-L-glutamate ligase [Fluoribacter dumoffii]	-9670.208999	-9666.562637	3.646362	7.292724	0.006923442	0.006923442	0.04562268	1
720	Anisa01128	671591086	WP_031566630.1	membrane protein [Legionella wadsworthii]	-14411.51563	-14407.72641	3.78922	7.57844	0.005907054	0.005907054	0.04127918	1
723	Anisa01131	654931161	WP_028381528.1	hypothetical protein [Legionella cherrii]	-6952.085803	-6947.323164	4.762639	9.525278	0.002026609	0.002026609	0.02252732	1
725	Anisa01136	498341065	WP_010655221.1	ribonuclease R [Fluoribacter dumoffii]	-15925.89509	-15921.14527	4.749819	9.499638	0.002055124	0.002055124	0.02252732	1
739	Anisa01177	489730068	WP_003634184.1	type IV secretion system protein IcmL [Legionella	-6472.285959	-6466.9891	5.296834	10.593668	0.001134756	0.001134756	0.01544716	1

				longbeachae]		25						
742	Anisa01182	498341025	WP_010655181.1	DSBA oxidoreductase [Fluoribacter dumoffii]	-7096.468486	-7091.235583	5.232903	10.465806	0.001216045	0.001216045	0.01575331	1
747	Anisa01199	654931224	WP_028381591.1	dihydrodipicolinate reductase [Legionella cherrii]	-9066.011356	-9060.717333	5.294023	10.588046	0.001138212	0.001138212	0.01544716	1
751	Anisa01206	654931230	WP_028381597.1	transketolase [Legionella cherrii]	-21752.70499	-21746.9698	5.73519	11.47038	0.000707142	0.000707142	0.01151631	1
772	Anisa01251	654930954	WP_028381322.1	zinc metalloprotease [Legionella cherrii]	-8341.426925	-8337.357872	4.069053	8.138106	0.004334453	0.004334454	0.03504452	1
797	Anisa01307	671586985	WP_031563323.1	30S ribosomal protein S4 [Legionella wadsworthii]	-4844.06657	-4837.65721	6.40936	12.81872	0.000343168	0.000343168	0.006745026	1
834	Anisa01351	671586698	WP_031563094.1	virulence factor [Legionella wadsworthii]	-7021.948337	-7013.483512	8.464825	16.92965	3.87909E-05	0.0000388	0.001426504	1
838	Anisa01355	654930382	WP_028380751.1	ATPase [Legionella cherrii]	-8670.295859	-8666.201932	4.093927	8.187854	0.004217178	0.004217178	0.0345729	1
842	Anisa01361	654930375	WP_028380744.1	DeoR family transcriptional regulator	-8044.385639	-8036.2983	8.087306	16.174612	5.77631E-05	0.0000578	0.002049378	1

				[Legionella cherrii]		33						
845	Anisa01364	654930372	WP_028380741.1	cell division protein [Legionella cherrii]	-10174.33661	-10167.7102	6.626415	13.25283	0.000272169	0.000272169	0.005823732	1
846	Anisa01365	498338318	WP_010652474.1	cell division protein FtsE [Fluoribacter dumoffii]	-3998.196076	-3994.324547	3.871529	7.743058	0.005391941	0.005391942	0.03991438	1
856	Anisa01378	671586766	WP_031563150.1	phosphoesterase [Legionella wadsworthii]	-19948.24326	-19942.10495	6.138303	12.276606	0.000458672	0.000458672	0.008503334	1
858	Anisa01383	654930355	WP_028380724.1	tRNA dimethylallyltransferase [Legionella cherrii]	-11265.62851	-11258.20102	7.427488	14.854976	0.000116101	0.000116101	0.003151313	1
859	Anisa01384	654930354	WP_028380723.1	DNA mismatch repair protein MutL [Legionella cherrii]	-15935.95847	-15931.2577	4.700768	9.401536	0.002168037	0.002168037	0.02309871	1
866	Anisa01391	498338294	WP_010652450.1	cytochrome B [Fluoribacter dumoffii]	-11045.44827	-11040.61862	4.829647	9.659294	0.00188396	0.00188396	0.02164035	1
881	Anisa01412	654930328	WP_028380697.1	cytochrome C [Legionella cherrii]	-4360.843544	-4357.000094	3.84345	7.6869	0.005562309	0.005562309	0.04013312	1
883	Anisa01418	654930323	WP_028380692.1	uroporphyrin-III methyltransferase [Legionella cherrii]	-15472.78162	-15468.649	4.132119	8.264238	0.004043363	0.004043363	0.03439876	1

						5						
885	Anisa01420	654930321	WP_028380690.1	cobalt transporter [Legionella cherrii]	-10120.15352	-10116.34311	3.810412	7.620824	0.005769812	0.005769812	0.04060238	1
923	Anisa01468	654931362	WP_028381728.1	DNA replication initiation factor [Legionella cherrii]	-7957.15456	-7952.29474	4.85982	9.71964	0.001823092	0.001823092	0.02137073	1
929	Anisa01484	654931353	WP_028381719.1	hypothetical protein [Legionella cherrii]	-6660.066931	-6655.78535	4.281581	8.563162	0.003430331	0.003430331	0.03008136	1
932	Anisa01491	654931343	WP_028381709.1	inorganic polyphosphate kinase [Legionella cherrii]	-8950.246769	-8943.035751	7.211018	14.422036	0.000146083	0.000146083	0.003700769	1
937	Anisa01520	654931607	WP_028381970.1	membrane protein [Legionella cherrii]	-15238.91923	-15232.07055	6.848683	13.697366	0.000214755	0.000214755	0.004896414	1
938	Anisa01522	654931606	WP_028381969.1	DNA mismatch repair protein MutS [Legionella cherrii]	-24515.46462	-24503.25375	12.210872	24.421744	7.73903E-07	0.000000774	0.0000802	1
941	Anisa01531	653019466	WP_027271416.1	recombinase RecX [Legionella sainthelensi]	-5581.806885	-5564.184888	17.621997	35.243994	2.90878E-09	2.91E-09	0.000000474	1
942	Anisa01532	654931597	WP_028381960.1	alanyl-tRNA synthetase [Legionella cherrii]	-24776.03923	-24712.614	63.425132	126.850264	2.00332E-29	2E-29	2.28E-26	1

						1						
943	Anisa01536	654931594	WP_028381957.1	short-chain dehydrogenase [Legionella cherrii]	-10093.60628	-10089.26487	4.341415	8.68283	0.003212218	0.003212218	0.02893727	1
956	Anisa01569	654931561	WP_028381924.1	homogentisate 1,2-dioxygenase [Legionella cherrii]	-12053.30944	-12048.38439	4.925054	9.850108	0.001698218	0.001698218	0.02071999	1
960	Anisa01573	671591892	WP_031567302.1	histidine kinase [Legionella wadsworthii]	-10654.12489	-10648.13437	5.990525	11.98105	0.000537443	0.000537443	0.009425923	1
971	Anisa01593	654931541	WP_028381904.1	glutamate--tRNA ligase [Legionella cherrii]	-9038.820297	-9033.556615	5.263682	10.527364	0.001176199	0.001176199	0.01559148	1
976	Anisa01631	654931628	WP_028381991.1	isovaleryl-CoA dehydrogenase [Legionella cherrii]	-9992.764089	-9984.134884	8.629205	17.25841	0.000032625	0.0000326	0.00123975	1
979	Anisa01634	654931631	WP_028381994.1	methylcrotonoyl-CoA carboxylase [Legionella cherrii]	-11044.12368	-11027.12761	16.996074	33.992148	5.53349E-09	5.53E-09	0.000000701	1
980	Anisa01635	654931632	WP_028381995.1	gamma-carboxygeranoyl-CoA hydratase [Legionella cherrii]	-7929.141347	-7925.192293	3.949054	7.898108	0.004948653	0.004948654	0.03760977	1
987	Anisa01642	654931641	WP_028382004.1	hypothetical protein [Legionella cherrii]	-14957.65841	-14954.039	3.618799	7.237598	0.007139243	0.007139243	0.04611212	1

						61						
989	Anisa01646	654931643	WP_028382006.1	glycyl-tRNA synthetase subunit beta [Legionella cherrii]	-19507.13832	-19503.35387	3.784449	7.568898	0.005938408	0.005938409	0.04127918	1
1003	Anisa01668	654931661	WP_028382024.1	CTP synthase [Legionella cherrii]	-15160.58992	-15154.96828	5.621646	11.243292	0.000799113	0.000799113	0.0124793	1
1005	Anisa01671	654931664	WP_028382027.1	Zn-dependent protease [Legionella cherrii]	-8954.13479	-8949.700286	4.434504	8.869008	0.002900527	0.002900527	0.02688293	1
1020	Anisa01721	489728341	WP_003632457.1	acyl-CoA dehydrogenase [Legionella longbeachae]	-11669.77233	-11665.2717	4.500626	9.001252	0.002697947	0.002697947	0.02606491	1
1022	Anisa01723	654931797	WP_028382160.1	3-hydroxyisobutyryl-CoA hydrolase [Legionella cherrii]	-13128.30872	-13123.45595	4.85277	9.70554	0.001837133	0.001837133	0.02137073	1
1023	Anisa01726	654931794	WP_028382157.1	hypothetical protein [Legionella cherrii]	-2303.796018	-2299.859137	3.936881	7.873762	0.00501572	0.0050 1572	0.03786703	1
1033	Anisa01738	654931783	WP_028382146.1	ABC transporter ATPase [Legionella cherrii]	-15094.63774	-15085.69615	8.941588	17.883176	2.34888E-05	0.0000235	0.00095633	1
1043	Anisa01773	654932319	WP_028382681.1	4-hydroxythreonine-4-phosphate dehydrogenase	-8523.839989	-8513.209977	10.630012	21.260024	4.01008E-06	0.00000401	0.000285718	1

				[Legionella cherrii]								
104<br>5	Anisa0<br>1775	654932317	WP_028382679.1	LPS biosynthesis protein [Legionella cherrii]	-<br>29265.<br>36206	-<br>2925<br>4.802<br>45	10.5<br>5961<br>2	21.119<br>224	4.31577E-06	0.0000<br>0432	0.00028<br>9411	1
104<br>9	Anisa0<br>1779	498340700	WP_010654856.1	acyl-CoA dehydrogenase [Fluoribacter dumoffii]	-<br>24417.<br>54484	-<br>2441<br>3.995<br>54	3.54<br>9298	7.0985<br>96	0.007714436	0.0077<br>14436	0.04788<br>042	1
106<br>7	Anisa0<br>2042	654931943	WP_028382306.1	ABC transporter ATP-binding protein [Legionella cherrii]	-<br>13182.<br>70668	-<br>1317<br>7.002<br>6	5.70<br>4084	11.408<br>168	0.000731219	0.0007<br>31219	0.01174<br>07	1
107<br>2	Anisa0<br>2177	654931824	WP_028382187.1	alanine dehydrogenase [Legionella cherrii]	-<br>10202.<br>99087	-<br>1019<br>8.051<br>12	4.93<br>975	9.8795	0.001671303	0.0016<br>71304	0.02070<br>965	1
107<br>7	Anisa0<br>2189	654931835	WP_028382198.1	erythronate-4-phosphate dehydrogenase [Legionella cherrii]	-<br>15243.<br>5124	-<br>1523<br>9.657<br>14	3.85<br>5263	7.7105<br>26	0.005489975	0.0054<br>89976	0.04013<br>312	1
107<br>8	Anisa0<br>2192	654931838	WP_028382201.1	UDP-N-acetylmuramoylalanyl-D-glutamate--2,6-diaminopimelate ligase [Legionella cherrii]	-<br>16855.<br>28959	-<br>1684<br>8.073<br>11	7.21<br>6487	14.432<br>974	0.000145237	0.0001<br>45237	0.00370<br>0769	1
107<br>9	Anisa0<br>2193	671592591	WP_031567880.1	cell division protein [Legionella wadsworthii]	-<br>14648.<br>24813	-<br>1464<br>4.636<br>93	3.61<br>1197	7.2223<br>94	0.007199962	0.0071<br>99962	0.04611<br>212	1

1082	Anisa02196	654931842	WP_028382205.1	cell division protein MraZ [Legionella cherrii]	-4032.755461	-4028.003226	4.752235	9.50447	0.00204972	0.00204972	0.02252732	1
1094	Anisa02221	654931864	WP_028382227.1	alanine racemase [Legionella cherrii]	-11239.21574	-11231.53334	7.682397	15.364794	8.86243E-05	0.0000886	0.002729637	1
1097	Anisa02224	498338931	WP_010653087.1	helicase [Fluoribacter dumoffii]	-24800.26745	-24795.88337	4.384075	8.76815	0.003065362	0.003065362	0.0279561	1
1103	Anisa02233	654931877	WP_028382240.1	serine hydroxymethyltransferase [Legionella cherrii]	-10711.01206	-10704.31471	6.697348	13.394696	0.000252337	0.000252337	0.005532003	1
1109	Anisa02248	498338157	WP_010652313.1	RNA-binding protein [Fluoribacter dumoffii]	-13606.6266	-13600.41522	6.21138	12.42276	0.000424133	0.000424133	0.008195112	1
1126	Anisa02276	671591525	WP_031566996.1	diacylglyceryl transferase [Legionella wadsworthii]	-8048.052844	-8044.077508	3.975336	7.950672	0.004806952	0.004806953	0.03680596	1
1130	Anisa02282	654932084	WP_028382446.1	hypothetical protein [Legionella cherrii]	-13547.66617	-13543.68208	3.984089	7.968178	0.004760683	0.004760684	0.03680596	1
1142	Anisa02394	653018305	WP_027270265.1	iron transporter [Legionella sainthelensi]	-2615.615229	-2610.16119	5.454039	10.908078	0.000957458	0.000957458	0.0134521	1

1154	Anisa02423	498339851	WP_010654007.1	preprotein translocase SecA [Fluoribacter dumoffii]	-6302.906463	-6299.132015	3.774448	7.548896	0.006004685	0.006004686	0.04148692	1
1174	Anisa02969	654929380	WP_028379750.1	D-alanyl-D-alanine carboxypeptidase [Legionella cherrii]	-14360.77805	-14356.32735	4.450693	8.901386	0.002849543	0.002849543	0.02664429	1
1175	Anisa02972	654929376	WP_028379746.1	glutathione reductase [Legionella cherrii]	-16052.60334	-16044.85256	7.750776	15.501552	8.24375E-05	0.0000824	0.002685107	1
1177	Anisa02977	498339190	WP_010653346.1	long-chain fatty acid transporter [Fluoribacter dumoffii]	-18253.25566	-18246.34416	6.911498	13.822996	0.000200863	0.000200863	0.004673139	1
1180	Anisa02988	654931622	WP_028381985.1	hypothetical protein [Legionella cherrii]	-11115.51242	-11111.85676	3.655658	7.311316	0.006852173	0.006852173	0.04541557	1
1185	Anisa03060	654931901	WP_028382264.1	carbonic anhydrase [Legionella cherrii]	-24182.59939	-24178.7625	3.836892	7.673784	0.005602884	0.005602884	0.04017162	1
1202	Anisa03085	654929711	WP_028380081.1	tRNA(Ile)-lysidine synthetase [Legionella cherrii]	-14852.74328	-14847.23586	5.507414	11.014828	0.000903859	0.000903859	0.01321025	1
1220	Anisa03106	498340033	WP_010654189.1	iron ABC transporter ATP-binding protein [Fluoribacter dumoffii]	-7436.979488	-7431.711363	5.268125	10.53625	0.001170557	0.001170557	0.01559148	1

122<br>5	Anisa0<br>3126	654931114	WP_028381481.1	nucleoside-<br>diphosphate sugar<br>epimerase<br>[Legionella cherrii]	-<br>5945.2<br>86621	-<br>5937.<br>9014<br>02	7.38<br>5219	14.770<br>438	0.000121424	0.0001<br>21424	0.00321<br>9148	1
122<br>6	Anisa0<br>3128	671591180	WP_031566708.1	phosphatidylserine<br>decarboxylase<br>[Legionella<br>wadsworthii]	-<br>10660.<br>98425	-<br>1065<br>7.171<br>54	3.81<br>2713	7.6254<br>26	0.005755107	0.0057<br>55108	0.04060<br>238	1
122<br>7	Anisa0<br>3131	489729832	WP_003633948.1	hypothetical protein<br>[Legionella<br>longbeachae]	-<br>13499.<br>633	-<br>1349<br>5.052<br>5	4.58<br>0509	9.1610<br>18	0.00247225	0.0024<br>7225	0.02472<br>25	1
122<br>9	Anisa0<br>3138	699016736	AIU36101.1	LglB [Legionella<br>parisiensis]	-<br>16479.<br>13272	-<br>1647<br>5.404<br>84	3.72<br>7882	7.4557<br>64	0.006323363	0.0063<br>23364	0.04316<br>548	1
123<br>9	Anisa0<br>3158	654929994	WP_028380364.1	lipid-A-<br>disaccharide<br>synthase<br>[Legionella cherrii]	-<br>14098.<br>39949	-<br>1408<br>8.884<br>67	9.51<br>4818	19.029<br>636	1.28704E-05	0.0000<br>129	0.00063<br>7924	1
124<br>7	Anisa0<br>3171	654929982	WP_028380352.1	type II secretory<br>pathway protein<br>LspJ [Legionella<br>cherrii]	-<br>7836.8<br>38738	-<br>7832.<br>3239<br>18	4.51<br>482	9.0296<br>4	0.002656368	0.0026<br>56368	0.02588<br>256	1
124<br>8	Anisa0<br>3172	671589679	WP_031565461.1	type II secretory<br>pathway protein<br>[Legionella<br>wadsworthii]	-<br>7330.3<br>67888	-<br>7323.<br>3580<br>53	7.00<br>9835	14.019<br>67	0.000180908	0.0001<br>80908	0.00438<br>7981	1
125<br>0	Anisa0<br>3176	654929977	WP_028380347.1	peptidase PMbA<br>[Legionella cherrii]	-<br>11303.<br>10891	-<br>1129<br>8.579<br>86	4.52<br>9054	9.0581<br>08	0.002615325	0.0026<br>15325	0.02570<br>233	1
125	Anisa0	654930699	WP_028381068.1	hypothetical protein	-	-	9.81	19.628	9.40749E-06	0.0000	0.00049	1

8	3225			[Legionella cherrii]	11855. 66649	1184 5.852 44	4055	11		0941	0352	
126 2	Anisa0 3229	654930695	WP_028381064.1	cytochrome C oxidase subunit II [Legionella cherrii]	- 17058. 22371	- 1705 2.779 43	5.44 4274	10.888 548	0.000967607	0.0009 67607	0.01345 21	1
127 8	Anisa0 3255	498338382	WP_010652538.1	nitrogen fixation protein NifU [Fluoribacter dumoffii]	- 4608.4 5769	- 4603. 2204 29	5.23 7261	10.474 522	0.001210321	0.0012 10321	0.01575 331	1
128 2	Anisa0 3259	489730537	WP_003634653.1	cysteine desulfurase activator complex subunit SufB [Legionella longbeachae]	- 11738. 01046	- 1173 3.333 19	4.67 7273	9.3545 46	0.002224331	0.0022 24331	0.02347 905	1
129 4	Anisa0 3278	654930406	WP_028380775.1	5- hydroxymethylurac il DNA glycosylase [Legionella cherrii]	- 11077. 49774	- 1107 3.645 43	3.85 2309	7.7046 18	0.005507973	0.0055 07973	0.04013 312	1
130 3	Anisa0 3363	654929891	WP_028380261.1	integrase [Legionella cherrii]	- 13262. 90819	- 1325 6.462 33	6.44 5854	12.891 708	0.000330041	0.0003 30041	0.00660 082	1
130 9	Anisa0 3381	498340549	WP_010654705.1	hypothetical protein [Fluoribacter dumoffii]	- 27959. 00281	- 2795 4.539	4.46 3812	8.9276 24	0.002808895	0.0028 08895	0.02664 429	1
131 2	Anisa0 3384	671587258	WP_031563532.1	type IV secretion protein IcmC [Legionella wadsworthii]	- 3417.6 18047	- 3413. 3166 25	4.30 1422	8.6028 44	0.003356385	0.0033 56385	0.02966 108	1
131 3	Anisa0 3385	654930560	WP_028380929.1	type IV secretion protein IcmG	- 9996.7	- 9991.	4.72 1009	9.4420 18	0.0021207	0.0021 207	0.02297 231	1

				[Legionella cherrii]	04812	983803						
1317	Anisa03390	654930555	WP_028380924.1	protein LphA [Legionella cherrii]	-6214.810462	-6208.649436	6.161026	12.322052	0.000447638	0.000447638	0.008503334	1
1318	Anisa03391	498340560	WP_010654716.1	phosphoesterase [Fluoribacter dumoffii]	-19413.74254	-19404.71149	9.031049	18.062098	2.13816E-05	0.0000214	0.000902779	1
1329	Anisa03588	654932073	WP_028382435.1	hypothetical protein [Legionella cherrii]	-23039.98781	-23036.48833	3.499481	6.998962	0.008155699	0.0081557	0.04945478	1
1330	Anisa03590	654932071	WP_028382433.1	tRNA uridine 5-carboxymethylaminomethyl modification protein [Legionella cherrii]	-19784.10605	-19778.27101	5.835037	11.670074	0.000635135	0.000635135	0.01049353	1
1332	Anisa03592	498338108	WP_010652264.1	cobyric acid synthase CobQ [Fluoribacter dumoffii]	-7352.972277	-7341.555706	11.416571	22.833142	1.76692E-06	0.00000177	0.000143878	1
1352	Anisa03710	654929901	WP_028380271.1	potassium transporter Kup [Legionella cherrii]	-18043.66774	-18039.0172	4.650533	9.301066	0.002290206	0.002290206	0.02367333	1
1359	Anisa03718	654929909	WP_028380279.1	histidinol dehydrogenase [Legionella cherrii]	-17828.07372	-17824.57361	3.500107	7.000214	0.008149997	0.008149998	0.04945478	1

4f: Results of the GO term enrichment analysis of the core genes predicted to be under positive selection in Clade 3 compared to other Legionella clades. Performed hypergeometric probability distribution Test

GOID	Ontology	Term	Level	q	m	t	k	log_odds_ratio	FDR corrected p-value at significance level of 0.05
GO:0003674	molecular_function	molecular_function	0	124	5235	12977	170	0.854500834	1.05E-15
GO:0003824	molecular_function	catalytic activity	1	102	3902	12977	170	0.996705625	5.77E-14
GO:0016874	molecular_function	ligase activity	2	12	197	12977	170	2.217189027	0.000172522
GO:0006082	biological_process	organic acid metabolic process	4	22	375	12977	170	2.162963179	9.04E-08
GO:0006139	biological_process	nucleobase-containing compound metabolic process	4	35	1118	12977	170	1.25685689	1.69E-05
GO:0006399	biological_process	tRNA metabolic process	8	7	86	12977	170	2.635368513	0.001663067
GO:0006412	biological_process	translation	6	6	131	12977	170	1.805817845	0.039436397
GO:0006418	biological_process	tRNA aminoacylation for protein translation	10	4	36	12977	170	3.084353344	0.011242015
GO:0006520	biological_process	cellular amino acid metabolic process	7	15	269	12977	170	2.089706579	4.42E-05
GO:0006725	biological_process	cellular aromatic compound metabolic process	3	41	1244	12977	170	1.33105958	3.83E-07
GO:0006807	biological_process	nitrogen compound metabolic process	2	54	1493	12977	170	1.465167398	5.25E-11
GO:0008150	biological_process	biological_process	0	130	5467	12977	170	0.860112498	4.85E-17
GO:0008152	biological_process	metabolic process	1	115	4493	12977	170	0.96630505	3.63E-16
GO:0009058	biological_process	biosynthetic process	2	43	1051	12977	170	1.642996146	3.78E-10
GO:0009059	biological_process	macromolecule biosynthetic process	4	16	502	12977	170	1.282734792	0.008892399
GO:0009987	biological_process	cellular process	1	88	2986	12977	170	1.169711514	3.68E-14
GO:0016070	biological_process	RNA metabolic process	6	11	318	12977	170	1.400827009	0.022968587
GO:0019538	biological_process	protein metabolic process	4	21	586	12977	170	1.451838914	0.000389738
GO:0019752	biological_process	carboxylic acid metabolic process	6	22	370	12977	170	2.182328504	7.63E-08
GO:0034641	biological_process	cellular nitrogen compound metabolic	3	44	1271	12977	170	1.401961649	2.90E-08

		process							
GO:0034645	biological_process	cellular macromolecule biosynthetic process	5	15	432	12977	170	1.406281439	0.005747919
GO:0034660	biological_process	ncRNA metabolic process	7	7	108	12977	170	2.306745765	0.005605344
GO:0043038	biological_process	amino acid activation	8	4	37	12977	170	3.04482498	0.011899589
GO:0043039	biological_process	tRNA aminoacylation	9	4	37	12977	170	3.04482498	0.011899589
GO:0043170	biological_process	macromolecule metabolic process	3	44	1597	12977	170	1.072561367	1.69E-05
GO:0043436	biological_process	oxoacid metabolic process	5	22	372	12977	170	2.174551153	8.21E-08
GO:0044237	biological_process	cellular metabolic process	2	72	2220	12977	170	1.307859386	6.39E-13
GO:0044238	biological_process	primary metabolic process	2	70	2261	12977	170	1.240816086	1.49E-11
GO:0044249	biological_process	cellular biosynthetic process	3	39	891	12977	170	1.740398943	5.64E-10
GO:0044260	biological_process	cellular macromolecule metabolic process	4	31	1235	12977	170	0.93817933	0.003339897
GO:0044267	biological_process	cellular protein metabolic process	5	12	382	12977	170	1.261812018	0.027883069
GO:0044281	biological_process	small molecule metabolic process	3	35	658	12977	170	2.021617589	4.45E-11
GO:0044710	biological_process	single-organism metabolic process	2	55	1380	12977	170	1.605185507	9.21E-13
GO:0046483	biological_process	heterocycle metabolic process	3	45	1260	12977	170	1.446923424	8.52E-09
GO:0071704	biological_process	organic substance metabolic process	2	80	2451	12977	170	1.317051672	8.03E-15
GO:0090304	biological_process	nucleic acid metabolic process	5	24	902	12977	170	1.022257223	0.006905543
GO:1901360	biological_process	organic cyclic compound metabolic process	3	46	1288	12977	170	1.446923424	5.23E-09
GO:1901564	biological_process	organonitrogen compound metabolic process	3	33	587	12977	170	2.101455772	4.66E-11
GO:1901576	biological_process	organic substance biosynthetic process	3	40	961	12977	170	1.66781382	1.32E-09
GO:0005488	molecular_function	binding	1	75	2476	12977	170	1.209301437	4.12E-12
GO:0097159	molecular_function	organic cyclic compound binding	2	57	1879	12977	170	1.211419008	8.70E-09
GO:1901363	molecular_function	heterocyclic compound binding	2	57	1879	12977	170	1.211419008	8.70E-09
GO:0000166	molecular_function	nucleotide binding	4	43	946	12977	170	1.794846727	1.51E-11
GO:0036094	molecular_function	small molecule binding	2	43	986	12977	170	1.735099264	5.20E-11

GO:1901265	molecular_function	nucleoside phosphate binding	3	43	946	12977	170	1.794846727	1.51E-11
GO:0005575	cellular_component	cellular_component	0	62	2157	12977	170	1.133664195	8.74E-09
GO:0005622	cellular_component	intracellular	3	37	931	12977	170	1.601094354	2.21E-08
GO:0005623	cellular_component	cell	1	53	1249	12977	170	1.695641039	3.09E-13
GO:0005737	cellular_component	cytoplasm	5	30	572	12977	170	2.001297604	2.39E-09
GO:0044424	cellular_component	intracellular part	4	35	680	12977	170	1.974170426	9.30E-11
GO:0044464	cellular_component	cell part	2	52	1200	12977	170	1.725899373	2.95E-13
GO:0008270	molecular_function	zinc ion binding	6	8	129	12977	170	2.24305109	0.003336814
GO:0043167	molecular_function	ion binding	2	64	1450	12977	170	1.752441161	5.80E-17
GO:0043169	molecular_function	cation binding	3	28	612	12977	170	1.804245425	1.26E-07
GO:0046872	molecular_function	metal ion binding	4	28	596	12977	170	1.842464747	8.64E-08
GO:0046914	molecular_function	transition metal ion binding	5	14	240	12977	170	2.154742672	5.64E-05
GO:0001882	molecular_function	nucleoside binding	3	32	776	12977	170	1.654365503	1.20E-07
GO:0001883	molecular_function	purine nucleoside binding	4	32	772	12977	170	1.661821308	1.11E-07
GO:0005524	molecular_function	ATP binding	8	32	713	12977	170	1.776520079	2.17E-08
GO:0017076	molecular_function	purine nucleotide binding	5	32	778	12977	170	1.650652001	1.22E-07
GO:0030554	molecular_function	adenyl nucleotide binding	6	32	715	12977	170	1.772478914	2.19E-08
GO:0032549	molecular_function	ribonucleoside binding	4	32	773	12977	170	1.659953742	1.12E-07
GO:0032550	molecular_function	purine ribonucleoside binding	5	32	772	12977	170	1.661821308	1.11E-07
GO:0032553	molecular_function	ribonucleotide binding	5	34	801	12977	170	1.696082754	2.21E-08
GO:0032555	molecular_function	purine ribonucleotide binding	6	32	777	12977	170	1.652507557	1.21E-07
GO:0032559	molecular_function	adenyl ribonucleotide binding	7	32	714	12977	170	1.774498082	2.18E-08
GO:0035639	molecular_function	purine ribonucleoside triphosphate binding	4	32	771	12977	170	1.663691296	1.11E-07
GO:0043168	molecular_function	anion binding	3	45	1011	12977	170	1.76456416	9.35E-12
GO:0004812	molecular_function	aminoacyl-tRNA ligase activity	5	4	37	12977	170	3.04482498	0.011899589
GO:0016875	molecular_function	ligase activity, forming carbon-oxygen bonds	3	4	38	12977	170	3.006350832	0.012809311

GO:0016876	molecular_function	ligase activity, forming aminoacyl-tRNA and related compounds	4	4	38	12977	170	3.006350832	0.012809311
GO:0006508	biological_process	proteolysis	5	8	208	12977	170	1.553838627	0.033635178
GO:0008233	molecular_function	peptidase activity	3	8	203	12977	170	1.588942428	0.03091915
GO:0008237	molecular_function	metallopeptidase activity	5	7	66	12977	170	3.017239148	0.000356423
GO:0016787	molecular_function	hydrolase activity	2	31	1220	12977	170	0.955809224	0.002758017
GO:0070011	molecular_function	peptidase activity, acting on L-amino acid peptides	4	8	177	12977	170	1.786672796	0.017934185
GO:0004175	molecular_function	endopeptidase activity	5	5	83	12977	170	2.201167009	0.028161517
GO:0004222	molecular_function	metalloendopeptidase activity	6	5	35	12977	170	3.446923424	0.001176807
GO:0008360	biological_process	regulation of cell shape	6	3	27	12977	170	3.084353344	0.030169499
GO:0022603	biological_process	regulation of anatomical structure morphogenesis	4	3	28	12977	170	3.031885924	0.032235886
GO:0022604	biological_process	regulation of cell morphogenesis	5	3	28	12977	170	3.031885924	0.032235886
GO:0050793	biological_process	regulation of developmental process	3	3	28	12977	170	3.031885924	0.032235886
GO:0051128	biological_process	regulation of cellular component organization	4	3	36	12977	170	2.669315845	0.04502866
GO:0065008	biological_process	regulation of biological quality	2	6	91	12977	170	2.331446206	0.011344581
GO:0007049	biological_process	cell cycle	3	5	47	12977	170	3.021617589	0.003962378
GO:0044699	biological_process	single-organism process	1	29	1366	12977	170	0.696517572	0.033228914
GO:0044763	biological_process	single-organism cellular process	2	27	1252	12977	170	0.719147001	0.03553222
GO:0051301	biological_process	cell division	3	6	43	12977	170	3.412976092	0.000287343
GO:0009116	biological_process	nucleoside metabolic process	6	8	123	12977	170	2.31176384	0.002561556
GO:0055086	biological_process	nucleobase-containing small molecule metabolic process	5	10	227	12977	170	1.749657953	0.00798277
GO:1901135	biological_process	carbohydrate derivative metabolic process	3	14	259	12977	170	2.04482498	0.00012907
GO:1901657	biological_process	glycosyl compound metabolic process	3	8	123	12977	170	2.31176384	0.002561556
GO:0016740	molecular_function	transferase activity	2	28	1400	12977	170	0.610422156	0.049867357
GO:0016757	molecular_function	transferase activity, transferring	3	5	79	12977	170	2.272425692	0.025630943

		glycosyl groups							
GO:0016763	molecular_function	transferase activity, transferring pentosyl groups	4	3	31	12977	170	2.885044536	0.03953231
GO:0051052	biological_process	regulation of DNA metabolic process	6	2	7	12977	170	4.446923424	0.024374639
GO:0055114	biological_process	oxidation-reduction process	3	25	712	12977	170	1.422401104	0.000103515
GO:0016491	molecular_function	oxidoreductase activity	2	23	680	12977	170	1.368449366	0.000389738
GO:0016627	molecular_function	oxidoreductase activity, acting on the CH-CH group of donors	3	5	49	12977	170	2.961496596	0.004743139
GO:0016628	molecular_function	oxidoreductase activity, acting on the CH-CH group of donors, NAD or NADP as acceptor	4	2	12	12977	170	3.669315845	0.04502866
GO:0048037	molecular_function	cofactor binding	2	16	281	12977	170	2.119852025	1.62E-05
GO:0050662	molecular_function	coenzyme binding	3	12	188	12977	170	2.284651995	0.000112803
GO:0050660	molecular_function	flavin adenine dinucleotide binding	5	7	73	12977	170	2.871808709	0.000659634
GO:0006950	biological_process	response to stress	2	7	177	12977	170	1.594027718	0.04296169
GO:0006974	biological_process	response to DNA damage stimulus	5	6	98	12977	170	2.224531002	0.014404168
GO:0033554	biological_process	cellular response to stress	4	6	114	12977	170	2.006350832	0.025630943
GO:0006281	biological_process	DNA repair	7	6	98	12977	170	2.224531002	0.014404168
GO:0003690	molecular_function	double-stranded DNA binding	6	2	6	12977	170	4.669315845	0.018815807
GO:0006298	biological_process	mismatch repair	8	2	5	12977	170	4.932350251	0.013626039
GO:0006629	biological_process	lipid metabolic process	3	7	188	12977	170	1.507044416	0.04502866
GO:0044255	biological_process	cellular lipid metabolic process	4	6	120	12977	170	1.932350251	0.029636691
GO:0006793	biological_process	phosphorus metabolic process	3	16	592	12977	170	1.04482498	0.029001364
GO:0006796	biological_process	phosphate-containing compound metabolic process	4	16	588	12977	170	1.054606001	0.027883069
GO:0006766	biological_process	vitamin metabolic process	4	4	53	12977	170	2.526357891	0.029911844
GO:0006767	biological_process	water-soluble vitamin metabolic process	5	4	53	12977	170	2.526357891	0.029911844
GO:0008614	biological_process	pyridoxine metabolic process	7	2	12	12977	170	3.669315845	0.04502866
GO:0008615	biological_process	pyridoxine biosynthetic process	8	2	12	12977	170	3.669315845	0.04502866

GO:0009110	biological_process	vitamin biosynthetic process	5	4	50	12977	170	2.610422156	0.026188467
GO:0018130	biological_process	heterocycle biosynthetic process	4	19	458	12977	170	1.663002071	0.000143578
GO:0042364	biological_process	water-soluble vitamin biosynthetic process	6	4	50	12977	170	2.610422156	0.026188467
GO:0042816	biological_process	vitamin B6 metabolic process	6	2	12	12977	170	3.669315845	0.04502866
GO:0042819	biological_process	vitamin B6 biosynthetic process	7	2	12	12977	170	3.669315845	0.04502866
GO:0044271	biological_process	cellular nitrogen compound biosynthetic process	4	17	460	12977	170	1.496251136	0.0014448
GO:0044283	biological_process	small molecule biosynthetic process	4	12	248	12977	170	1.885044536	0.001356614
GO:0044711	biological_process	single-organism biosynthetic process	3	12	255	12977	170	1.844887409	0.001672363
GO:0072524	biological_process	pyridine-containing compound metabolic process	4	3	32	12977	170	2.839240846	0.042514755
GO:0072525	biological_process	pyridine-containing compound biosynthetic process	5	3	24	12977	170	3.254278346	0.025193905
GO:1901362	biological_process	organic cyclic compound biosynthetic process	4	19	476	12977	170	1.607388096	0.000235221
GO:1901566	biological_process	organonitrogen compound biosynthetic process	4	20	359	12977	170	2.088366407	9.26E-07
GO:0006732	biological_process	coenzyme metabolic process	4	5	101	12977	170	1.917994958	0.04502866
GO:0019637	biological_process	organophosphate metabolic process	4	11	255	12977	170	1.719356527	0.005605344
GO:0051186	biological_process	cofactor metabolic process	3	9	143	12977	170	2.26433201	0.001415468
GO:0051188	biological_process	cofactor biosynthetic process	4	6	114	12977	170	2.006350832	0.025630943
GO:0090407	biological_process	organophosphate biosynthetic process	5	10	193	12977	170	1.983749403	0.002656601
GO:0000287	molecular_function	magnesium ion binding	5	7	109	12977	170	2.293448943	0.00579895
GO:0016043	biological_process	cellular component organization	2	8	133	12977	170	2.19899591	0.003962378
GO:0071840	biological_process	cellular component organization or biogenesis	1	8	176	12977	170	1.794846727	0.017454537
GO:0030312	cellular_component	external encapsulating structure	4	3	35	12977	170	2.709957829	0.04502866
GO:0030313	cellular_component	cell envelope	4	3	29	12977	170	2.981259851	0.03424409
GO:0031975	cellular_component	envelope	3	3	29	12977	170	2.981259851	0.03424409

GO:0044462	cellular_component	external encapsulating structure part	5	3	28	12977	170	3.031885924	0.032235886
GO:0071944	cellular_component	cell periphery	3	14	273	12977	170	1.968876127	0.000222969
GO:0022607	biological_process	cellular component assembly	3	5	61	12977	170	2.645469103	0.011242015
GO:0044085	biological_process	cellular component biogenesis	2	7	119	12977	170	2.166815504	0.009189266
GO:0045229	biological_process	external encapsulating structure organization	3	4	37	12977	170	3.04482498	0.011899589
GO:0015036	molecular_function	disulfide oxidoreductase activity	4	2	14	12977	170	3.446923424	0.047275926
GO:0017004	biological_process	cytochrome complex assembly	7	2	13	12977	170	3.553838627	0.04502866
GO:0043623	biological_process	cellular protein complex assembly	6	2	14	12977	170	3.446923424	0.047275926
GO:0030288	cellular_component	outer membrane-bounded periplasmic space	6	2	8	12977	170	4.254278346	0.027883069
GO:0042597	cellular_component	periplasmic space	3	3	24	12977	170	3.254278346	0.025193905
GO:0006644	biological_process	phospholipid metabolic process	5	3	37	12977	170	2.629787481	0.04502866
GO:0008654	biological_process	phospholipid biosynthetic process	6	3	35	12977	170	2.709957829	0.04502866
GO:1901137	biological_process	carbohydrate derivative biosynthetic process	4	8	166	12977	170	1.879238914	0.013159514
GO:0006220	biological_process	pyrimidine nucleotide metabolic process	8	3	26	12977	170	3.138801128	0.028109786
GO:0006221	biological_process	pyrimidine nucleotide biosynthetic process	9	3	24	12977	170	3.254278346	0.025193905
GO:0006753	biological_process	nucleoside phosphate metabolic process	6	7	189	12977	170	1.499390843	0.04502866
GO:0009117	biological_process	nucleotide metabolic process	7	7	184	12977	170	1.538071312	0.04502866
GO:0009119	biological_process	ribonucleoside metabolic process	7	5	102	12977	170	1.903781099	0.04502866
GO:0009165	biological_process	nucleotide biosynthetic process	8	6	133	12977	170	1.783958411	0.041673303
GO:0009259	biological_process	ribonucleotide metabolic process	8	5	109	12977	170	1.808022116	0.047951144
GO:0019438	biological_process	aromatic compound biosynthetic process	4	14	426	12977	170	1.326923647	0.012680159
GO:0034654	biological_process	nucleobase-containing compound biosynthetic process	5	10	342	12977	170	1.158353926	0.049167606

GO:0072528	biological_process	pyrimidine-containing compound biosynthetic process	5	3	34	12977	170	2.751778005	0.04502866
GO:1901293	biological_process	nucleoside phosphate biosynthetic process	7	6	136	12977	170	1.751778005	0.043682196
GO:0005886	cellular_component	plasma membrane	4	12	240	12977	170	1.932350251	0.001065633
GO:0016462	molecular_function	pyrophosphatase activity	5	11	323	12977	170	1.37831961	0.024906259
GO:0016817	molecular_function	hydrolase activity, acting on acid anhydrides	3	11	329	12977	170	1.35176619	0.026188467
GO:0016818	molecular_function	hydrolase activity, acting on acid anhydrides, in phosphorus-containing anhydrides	4	11	326	12977	170	1.36498181	0.025630943
GO:0016887	molecular_function	ATPase activity	7	8	217	12977	170	1.492727113	0.040470026
GO:0017111	molecular_function	nucleoside-triphosphatase activity	6	10	314	12977	170	1.281585692	0.042883538
GO:0003995	molecular_function	acyl-CoA dehydrogenase activity	4	3	16	12977	170	3.839240846	0.010359402
GO:0006768	biological_process	biotin metabolic process	8	2	5	12977	170	4.932350251	0.013626039
GO:0009102	biological_process	biotin biosynthetic process	9	2	5	12977	170	4.932350251	0.013626039
GO:0016053	biological_process	organic acid biosynthetic process	5	8	194	12977	170	1.654365503	0.026188467
GO:0032787	biological_process	monocarboxylic acid metabolic process	7	6	79	12977	170	2.535460098	0.005891291
GO:0046394	biological_process	carboxylic acid biosynthetic process	7	8	194	12977	170	1.654365503	0.026188467
GO:0030170	molecular_function	pyridoxal phosphate binding	4	4	70	12977	170	2.124995329	0.04567503
GO:0032991	cellular_component	macromolecular complex	1	9	224	12977	170	1.616848425	0.020563739
GO:0043234	cellular_component	protein complex	2	8	150	12977	170	2.025459655	0.007905898
GO:0016879	molecular_function	ligase activity, forming carbon-nitrogen bonds	3	6	101	12977	170	2.181029364	0.01665551
GO:1901605	biological_process	alpha-amino acid metabolic process	8	10	171	12977	170	2.158353926	0.001167337
GO:0006522	biological_process	alanine metabolic process	9	2	5	12977	170	4.932350251	0.013626039
GO:0009078	biological_process	pyruvate family amino acid metabolic process	8	2	5	12977	170	4.932350251	0.013626039
GO:0008652	biological_process	cellular amino acid biosynthetic process	8	6	135	12977	170	1.762425249	0.04296169

GO:1901607	biological_process	alpha-amino acid biosynthetic process	9	6	98	12977	170	2.224531002	0.014404168
GO:0006733	biological_process	oxidoreduction coenzyme metabolic process	5	3	35	12977	170	2.709957829	0.04502866
GO:0071555	biological_process	cell wall organization	4	3	30	12977	170	2.932350251	0.037264023
GO:0000270	biological_process	peptidoglycan metabolic process	6	4	44	12977	170	2.794846727	0.019222313
GO:0006022	biological_process	aminoglycan metabolic process	4	4	49	12977	170	2.639568501	0.025630943
GO:0030203	biological_process	glycosaminoglycan metabolic process	5	4	45	12977	170	2.762425249	0.020563739
GO:0009147	biological_process	pyrimidine nucleoside triphosphate metabolic process	8	2	12	12977	170	3.669315845	0.04502866
GO:0009148	biological_process	pyrimidine nucleoside triphosphate biosynthetic process	9	2	11	12977	170	3.794846727	0.04296169
GO:0009056	biological_process	catabolic process	2	7	191	12977	170	1.48420444	0.04502866
GO:1901575	biological_process	organic substance catabolic process	3	7	177	12977	170	1.594027718	0.04296169
GO:0006260	biological_process	DNA replication	7	5	80	12977	170	2.254278346	0.026188467
GO:0008408	molecular_function	3'-5' exonuclease activity	6	2	13	12977	170	3.553838627	0.04502866
GO:0005506	molecular_function	iron ion binding	6	4	71	12977	170	2.104531226	0.047275926
GO:0016226	biological_process	iron-sulfur cluster assembly	5	2	6	12977	170	4.669315845	0.018815807
GO:0031163	biological_process	metallo-sulfur cluster assembly	4	2	6	12977	170	4.669315845	0.018815807
GO:0016054	biological_process	organic acid catabolic process	5	6	57	12977	170	3.006350832	0.001286309
GO:0044248	biological_process	cellular catabolic process	3	6	136	12977	170	1.751778005	0.043682196
GO:0044282	biological_process	small molecule catabolic process	4	6	66	12977	170	2.794846727	0.002597871
GO:0044712	biological_process	single-organism catabolic process	3	6	66	12977	170	2.794846727	0.002597871
GO:0046395	biological_process	carboxylic acid catabolic process	7	6	57	12977	170	3.006350832	0.001286309
GO:0006635	biological_process	fatty acid beta-oxidation	10	2	7	12977	170	4.446923424	0.024374639
GO:0009062	biological_process	fatty acid catabolic process	9	2	11	12977	170	3.794846727	0.04296169
GO:0016042	biological_process	lipid catabolic process	4	2	14	12977	170	3.446923424	0.047275926
GO:0019395	biological_process	fatty acid oxidation	9	2	7	12977	170	4.446923424	0.024374639
GO:0030258	biological_process	lipid modification	5	2	13	12977	170	3.553838627	0.04502866
GO:0034440	biological_process	lipid oxidation	6	2	7	12977	170	4.446923424	0.024374639

GO:0044242	biological_process	cellular lipid catabolic process	5	2	12	12977	170	3.669315845	0.04502866
GO:0072329	biological_process	monocarboxylic acid catabolic process	8	3	12	12977	170	4.254278346	0.004800061
GO:0016829	molecular_function	lyase activity	2	8	213	12977	170	1.519568725	0.03755786
GO:0007059	biological_process	chromosome segregation	3	2	12	12977	170	3.669315845	0.04502866
GO:0009890	biological_process	negative regulation of biosynthetic process	5	2	9	12977	170	4.084353344	0.032235886
GO:0009892	biological_process	negative regulation of metabolic process	4	2	10	12977	170	3.932350251	0.037754528
GO:0010558	biological_process	negative regulation of macromolecule biosynthetic process	6	2	9	12977	170	4.084353344	0.032235886
GO:0010605	biological_process	negative regulation of macromolecule metabolic process	5	2	10	12977	170	3.932350251	0.037754528
GO:0031324	biological_process	negative regulation of cellular metabolic process	5	2	10	12977	170	3.932350251	0.037754528
GO:0031327	biological_process	negative regulation of cellular biosynthetic process	6	2	9	12977	170	4.084353344	0.032235886
GO:0045934	biological_process	negative regulation of nucleobase-containing compound metabolic process	6	2	8	12977	170	4.254278346	0.027883069
GO:0048519	biological_process	negative regulation of biological process	3	2	13	12977	170	3.553838627	0.04502866
GO:0048523	biological_process	negative regulation of cellular process	4	2	13	12977	170	3.553838627	0.04502866
GO:0051172	biological_process	negative regulation of nitrogen compound metabolic process	5	2	8	12977	170	4.254278346	0.027883069
GO:2000113	biological_process	negative regulation of cellular macromolecule biosynthetic process	7	2	9	12977	170	4.084353344	0.032235886
GO:0003916	molecular_function	DNA topoisomerase activity	5	2	11	12977	170	3.794846727	0.04296169
GO:0006265	biological_process	DNA topological change	8	2	11	12977	170	3.794846727	0.04296169
GO:0006289	biological_process	nucleotide-excision repair	8	2	13	12977	170	3.553838627	0.04502866
GO:0016835	molecular_function	carbon-oxygen lyase activity	3	5	72	12977	170	2.406281439	0.018940079
GO:0009254	biological_process	peptidoglycan turnover	7	2	8	12977	170	4.254278346	0.027883069

Supplementary Table 5. GO term enrichment analysis of all the predicted genes gained over the *Legionella* phylogeny over time by gainLoss program. (**a**) Results of the GO term enrichment analysis done for the genes gained in clade 1 (*L. pneumophila*). (**b**) Results of the GO term enrichment analysis done for the genes gained in clade 2. (**c**) Results of the GO term enrichment analysis done for the genes gained in clade 3. (**d**) Results of the GO term enrichment analysis done for the genes gained in clade 4.

5a. Results of the GO term enrichment analysis done for the genes gained in clade 1

GOID	Ontology	Term	Level	q	m	t	k	log_odds_ratio	p
GO:0006139	biological_process	nucleobase-containing compound metabolic process	4	137	1118	12977	1014	0.649154955	4.47E-06
GO:0006259	biological_process	DNA metabolic process	6	83	531	12977	1014	1.000298725	8.66E-08
GO:0006725	biological_process	cellular aromatic compound metabolic process	3	145	1244	12977	1014	0.576965665	3.23E-05
GO:0006807	biological_process	nitrogen compound metabolic process	2	156	1493	12977	1014	0.419231113	0.003102809
GO:0008150	biological_process	biological_process	0	478	5467	12977	1014	0.162160493	0.017951638
GO:0009987	biological_process	cellular process	1	273	2986	12977	1014	0.226586036	0.038590658
GO:0015074	biological_process	DNA integration	7	24	134	12977	1014	1.196700655	0.004639739
GO:0034641	biological_process	cellular nitrogen compound metabolic process	3	146	1271	12977	1014	0.555903589	5.60E-05
GO:0043170	biological_process	macromolecule metabolic process	3	176	1597	12977	1014	0.496110366	5.60E-05
GO:0044238	biological_process	primary metabolic process	2	215	2261	12977	1014	0.283274918	0.023465721
GO:0044260	biological_process	cellular macromolecule metabolic process	4	140	1235	12977	1014	0.536815035	0.00017128
GO:0046483	biological_process	heterocycle metabolic process	3	146	1260	12977	1014	0.568443885	3.85E-05
GO:0071704	biological_process	organic substance metabolic process	2	227	2451	12977	1014	0.245221063	0.049483425

GO:0090304	biological_process	nucleic acid metabolic process	5	120	902	12977	1014	0.767734317	3.56E-07
GO:1901360	biological_process	organic cyclic compound metabolic process	3	147	1288	12977	1014	0.546582811	6.58E-05
GO:0006310	biological_process	DNA recombination	7	38	265	12977	1014	0.875906309	0.007813603
GO:0010468	biological_process	regulation of gene expression	5	32	248	12977	1014	0.723631034	0.068729444
GO:0019222	biological_process	regulation of metabolic process	3	44	339	12977	1014	0.7321175	0.019872792
GO:0006401	biological_process	RNA catabolic process	7	7	28	12977	1014	1.677827345	0.084728338
GO:0006402	biological_process	mRNA catabolic process	8	7	21	12977	1014	2.092864844	0.024109702
GO:0016071	biological_process	mRNA metabolic process	7	7	25	12977	1014	1.841326077	0.054226456
GO:0044265	biological_process	cellular macromolecule catabolic process	5	9	40	12977	1014	1.525824251	0.064942648
GO:0006109	biological_process	regulation of carbohydrate metabolic process	5	7	21	12977	1014	2.092864844	0.024109702
GO:0006313	biological_process	transposition, DNA-mediated	8	22	132	12977	1014	1.092864844	0.019346967
GO:0032196	biological_process	transposition	3	22	132	12977	1014	1.092864844	0.019346967
GO:0042120	biological_process	alginic acid metabolic process	7	5	12	12977	1014	2.414792939	0.039113482
GO:0042121	biological_process	alginic acid biosynthetic process	8	5	12	12977	1014	2.414792939	0.039113482
GO:0006754	biological_process	ATP biosynthetic process	11	8	30	12977	1014	1.770936749	0.044618485
GO:0009145	biological_process	purine nucleoside triphosphate biosynthetic process	9	8	31	12977	1014	1.723631034	0.049483425
GO:0009206	biological_process	purine ribonucleoside triphosphate biosynthetic process	10	8	31	12977	1014	1.723631034	0.049483425
GO:0015985	biological_process	energy coupled proton transport, down electrochemical gradient	9	8	29	12977	1014	1.81984635	0.038513434
GO:0015986	biological_process	ATP synthesis coupled proton transport	12	8	29	12977	1014	1.81984635	0.038513434

GO:0046034	biological_process	ATP metabolic process	10	8	32	12977	1014	1.677827345	0.056038421
GO:0051704	biological_process	multi-organism process	1	27	85	12977	1014	2.023323911	2.10E-08
GO:0000746	biological_process	conjugation	3	16	28	12977	1014	2.870472423	4.27E-09
GO:0044764	biological_process	multi-organism cellular process	2	19	39	12977	1014	2.640352639	2.87E-09
GO:0006304	biological_process	DNA modification	7	11	37	12977	1014	1.927805598	0.003871707
GO:0006305	biological_process	DNA alkylation	8	7	14	12977	1014	2.677827345	0.001912482
GO:0006306	biological_process	DNA methylation	9	7	14	12977	1014	2.677827345	0.001912482
GO:0040029	biological_process	regulation of gene expression, epigenetic	6	7	14	12977	1014	2.677827345	0.001912482
GO:0044728	biological_process	DNA methylation or demethylation	8	7	14	12977	1014	2.677827345	0.001912482
GO:0006952	biological_process	defense response	3	6	19	12977	1014	2.014862332	0.0557122
GO:0043570	biological_process	maintenance of DNA repeat elements	7	4	5	12977	1014	3.35589925	0.007449851
GO:0043571	biological_process	maintenance of CRISPR repeat elements	8	4	5	12977	1014	3.35589925	0.007449851
GO:0002252	biological_process	immune effector process	2	3	5	12977	1014	2.940861751	0.075563879
GO:0009607	biological_process	response to biotic stimulus	2	3	5	12977	1014	2.940861751	0.075563879
GO:0009615	biological_process	response to virus	4	3	5	12977	1014	2.940861751	0.075563879
GO:0051607	biological_process	defense response to virus	5	3	5	12977	1014	2.940861751	0.075563879
GO:0051707	biological_process	response to other organism	3	3	5	12977	1014	2.940861751	0.075563879
GO:0016469	cellular_component	proton-transporting two-sector ATPase complex	3	8	32	12977	1014	1.677827345	0.056038421
GO:0045259	cellular_component	proton-transporting ATP synthase complex	5	7	27	12977	1014	1.730294765	0.074640918
GO:0003674	molecular_function	molecular_function	0	477	5235	12977	1014	0.221698979	0.000248809
GO:0003676	molecular_function	nucleic acid binding	3	147	1016	12977	1014	0.888815003	4.94E-11
GO:0003677	molecular_function	DNA binding	4	111	691	12977	1014	1.039701311	5.10E-11
GO:0005488	molecular_function	binding	1	260	2476	12977	1014	0.426399559	5.83E-06
GO:0097159	molecular_function	organic cyclic compound	2	225	1879	12977	1014	0.615859185	1.22E-09

		binding							
GO:1901363	molecular_function	heterocyclic compound binding	2	225	1879	12977	1014	0.615859185	1.22E-09
GO:0043565	molecular_function	sequence-specific DNA binding	5	21	82	12977	1014	1.712592763	6.30E-05
GO:0004803	molecular_function	transposase activity	2	22	131	12977	1014	1.103835962	0.018460315
GO:0019829	molecular_function	cation-transporting ATPase activity	12	11	52	12977	1014	1.436819245	0.049314308
GO:0042623	molecular_function	ATPase activity, coupled	8	22	153	12977	1014	0.879871121	0.074640918
GO:0042625	molecular_function	ATPase activity, coupled to transmembrane movement of ions	11	11	53	12977	1014	1.409338509	0.052354642
GO:0044769	molecular_function	ATPase activity, coupled to transmembrane movement of ions, rotational mechanism	12	7	27	12977	1014	1.730294765	0.074640918
GO:0046933	molecular_function	proton-transporting ATP synthase activity, rotational mechanism	13	7	26	12977	1014	1.784742549	0.064413092
GO:0009008	molecular_function	DNA-methyltransferase activity	5	4	8	12977	1014	2.677827345	0.049483425
GO:0003857	molecular_function	3-hydroxyacyl-CoA dehydrogenase activity	5	4	7	12977	1014	2.870472423	0.032939542

5b. Results of the GO term enrichment analysis done for the genes gained in clade 2

GOID	Ontology	Term	Level	q	m	t	k	log_odds_ratio	p
GO:0004803	molecular_function	transposase activity	2	40	131	12977	1985	0.997251083	0.00130407
GO:0003676	molecular_function	nucleic acid binding	3	226	1016	12977	1985	0.540240265	4.95E-07
GO:0097159	molecular_function	organic cyclic compound binding	2	340	1879	12977	1985	0.242387574	0.01395028
GO:1901363	molecular_function	heterocyclic compound binding	2	340	1879	12977	1985	0.242387574	0.01395028

GO:0006139	biological_process	nucleobase-containing compound metabolic process	4	208	1118	12977	1985	0.282481235	0.054230393
GO:0006259	biological_process	DNA metabolic process	6	131	531	12977	1985	0.68960094	3.19E-06
GO:0006310	biological_process	DNA recombination	7	70	265	12977	1985	0.788180457	0.000380042
GO:0006313	biological_process	transposition, DNA-mediated	8	40	132	12977	1985	0.986279965	0.001308016
GO:0032196	biological_process	transposition	3	40	132	12977	1985	0.986279965	0.001308016
GO:0090304	biological_process	nucleic acid metabolic process	5	181	902	12977	1985	0.391608254	0.005176305
GO:0003677	molecular_function	DNA binding	4	172	691	12977	1985	0.702468844	1.57E-08
GO:0043565	molecular_function	sequence-specific DNA binding	5	29	82	12977	1985	1.20917498	0.00113021
GO:0000746	biological_process	conjugation	3	17	28	12977	1985	1.988853909	2.00E-05
GO:0044764	biological_process	multi-organism cellular process	2	19	39	12977	1985	1.671271284	0.000291731
GO:0051704	biological_process	multi-organism process	1	31	85	12977	1985	1.253551364	0.000340357
GO:0006304	biological_process	DNA modification	7	15	37	12977	1985	1.40618322	0.01395028
GO:0006305	biological_process	DNA alkylation	8	8	14	12977	1985	1.901391068	0.022562288
GO:0006306	biological_process	DNA methylation	9	8	14	12977	1985	1.901391068	0.022562288
GO:0040029	biological_process	regulation of gene expression, epigenetic	6	8	14	12977	1985	1.901391068	0.022562288
GO:0044728	biological_process	DNA methylation or demethylation	8	8	14	12977	1985	1.901391068	0.022562288
GO:0006952	biological_process	defense response	3	9	19	12977	1985	1.630743478	0.054230393
GO:0043570	biological_process	maintenance of DNA repeat elements	7	5	5	12977	1985	2.70874599	0.006903214
GO:0043571	biological_process	maintenance of CRISPR repeat elements	8	5	5	12977	1985	2.70874599	0.006903214
GO:0002252	biological_process	immune effector process	2	5	5	12977	1985	2.70874599	0.006903214
GO:0002376	biological_process	immune system process	1	5	7	12977	1985	2.223319163	0.072396578
GO:0009607	biological_process	response to biotic stimulus	2	5	5	12977	1985	2.70874599	0.006903214
GO:0009615	biological_process	response to virus	4	5	5	12977	1985	2.70874599	0.006903214

GO:0051607	biological_process	defense response to virus	5	5	5	12977	1985	2.70874599	0.006903214
GO:0051707	biological_process	response to other organism	3	5	5	12977	1985	2.70874599	0.006903214

5c. Results of the GO term enrichment analysis done for the genes gained in clade 3

GOID	Ontology	Term	Level	q	m	t	k	log_odds_ratio	p
GO:0003676	molecular_function	nucleic acid binding	3	125	1016	12977	1168	0.450944321	0.010243167
GO:0015074	biological_process	DNA integration	7	24	134	12977	1168	0.992718033	0.044043874
GO:0006402	biological_process	mRNA catabolic process	8	7	21	12977	1168	1.888882222	0.081048572
GO:0006109	biological_process	regulation of carbohydrate metabolic process	5	7	21	12977	1168	1.888882222	0.081048572
GO:0003677	molecular_function	DNA binding	4	95	691	12977	1168	0.611158431	0.001453128
GO:0043565	molecular_function	sequence-specific DNA binding	5	20	82	12977	1168	1.438220813	0.002369856
GO:0000746	biological_process	conjugation	3	16	28	12977	1168	2.666489801	1.66E-07
GO:0044764	biological_process	multi-organism cellular process	2	18	39	12977	1168	2.358367505	6.06E-07
GO:0051704	biological_process	multi-organism process	1	26	85	12977	1168	1.764893505	4.16E-06
GO:0006304	biological_process	DNA modification	7	12	37	12977	1168	1.849353858	0.004504838
GO:0006305	biological_process	DNA alkylation	8	8	14	12977	1168	2.666489801	0.000786165
GO:0006306	biological_process	DNA methylation	9	8	14	12977	1168	2.666489801	0.000786165
GO:0040029	biological_process	regulation of gene expression, epigenetic	6	8	14	12977	1168	2.666489801	0.000786165
GO:0044728	biological_process	DNA methylation or demethylation	8	8	14	12977	1168	2.666489801	0.000786165
GO:0034637	biological_process	cellular carbohydrate biosynthetic process	6	10	36	12977	1168	1.625847816	0.049359401
GO:0005984	biological_process	disaccharide metabolic process	6	5	5	12977	1168	3.473844723	0.000786165
GO:0005991	biological_process	trehalose metabolic process	7	5	5	12977	1168	3.473844723	0.000786165

5d. Results of the GO term enrichment analysis done for the genes gained in clade 4

GOID	Ontology	Term	Level	q	m	t	k	log_odds_ratio	p
GO:0003674	molecular_function	molecular_function	0	325	5235	12977	714	0.174211104	0.032555567
GO:0006812	biological_process	cation transport	6	15	142	12977	714	0.941032494	0.097975413
GO:0006139	biological_process	nucleobase-containing compound metabolic process	4	106	1118	12977	714	0.785104999	9.16E-07
GO:0006725	biological_process	cellular aromatic compound metabolic process	3	108	1244	12977	714	0.65800575	4.84E-05
GO:0006807	biological_process	nitrogen compound metabolic process	2	115	1493	12977	714	0.485380619	0.002617587
GO:0009987	biological_process	cellular process	1	196	2986	12977	714	0.254600412	0.03508273
GO:0034641	biological_process	cellular nitrogen compound metabolic process	3	107	1271	12977	714	0.613607689	0.000154539
GO:0044238	biological_process	primary metabolic process	2	148	2261	12977	714	0.250597107	0.095944027
GO:0046483	biological_process	heterocycle metabolic process	3	108	1260	12977	714	0.639568501	7.81E-05
GO:1901360	biological_process	organic cyclic compound metabolic process	3	108	1288	12977	714	0.607859642	0.000158433
GO:0005488	molecular_function	binding	1	190	2476	12977	714	0.479949027	1.75E-05
GO:0006259	biological_process	DNA metabolic process	6	69	531	12977	714	1.239845424	3.26E-09
GO:0006310	biological_process	DNA recombination	7	31	265	12977	714	1.088236779	0.001816809
GO:0043170	biological_process	macromolecule metabolic process	3	125	1597	12977	714	0.508524705	0.000730793
GO:0044260	biological_process	cellular macromolecule metabolic process	4	107	1235	12977	714	0.655060678	5.65E-05
GO:0090304	biological_process	nucleic acid metabolic process	5	94	902	12977	714	0.921494246	7.53E-08
GO:0000150	molecular_function	recombinase activity	2	6	18	12977	714	2.598926517	0.007249711
GO:0003676	molecular_function	nucleic acid binding	3	125	1016	12977	714	1.160988616	1.66E-15
GO:0003677	molecular_function	DNA binding	4	96	691	12977	714	1.336309618	4.86E-15
GO:0097159	molecular_function	organic cyclic compound binding	2	167	1879	12977	714	0.691843959	5.31E-09
GO:1901363	molecular_function	heterocyclic compound	2	167	1879	12977	714	0.691843959	5.31E-09

		binding							
GO:0008168	molecular_function	methyltransferase activity	4	19	195	12977	714	0.824486217	0.097316815
GO:0032259	biological_process	methylation	2	19	195	12977	714	0.824486217	0.097316815
GO:0004803	molecular_function	transposase activity	2	15	131	12977	714	1.057356612	0.06025436
GO:0006313	biological_process	transposition, DNA-mediated	8	15	132	12977	714	1.046385494	0.062956356
GO:0032196	biological_process	transposition	3	15	132	12977	714	1.046385494	0.062956356
GO:0015074	biological_process	DNA integration	7	19	134	12977	714	1.365727341	0.003586365
GO:0043565	molecular_function	sequence-specific DNA binding	5	20	82	12977	714	2.148265108	9.16E-07
GO:0000746	biological_process	conjugation	3	14	28	12977	714	3.183889018	5.31E-09
GO:0044764	biological_process	multi-organism cellular process	2	16	39	12977	714	2.898486799	6.98E-09
GO:0051704	biological_process	multi-organism process	1	25	85	12977	714	2.418354271	5.54E-10
GO:0065007	biological_process	biological regulation	1	58	683	12977	714	0.626128245	0.013104678
GO:0030255	biological_process	protein secretion by the type IV secretion system	8	3	8	12977	714	2.768851518	0.073074752
GO:0044097	biological_process	secretion by the type IV secretion system	7	3	8	12977	714	2.768851518	0.073074752
GO:0010468	biological_process	regulation of gene expression	5	24	248	12977	714	0.814655208	0.056366312
GO:0019222	biological_process	regulation of metabolic process	3	35	339	12977	714	0.908030572	0.006520433
GO:0050789	biological_process	regulation of biological process	2	56	658	12977	714	0.629300166	0.014631517
GO:0060255	biological_process	regulation of macromolecule metabolic process	4	24	264	12977	714	0.724457399	0.096909863
GO:0080090	biological_process	regulation of primary metabolic process	4	28	311	12977	714	0.71047317	0.070579771
GO:0006401	biological_process	RNA catabolic process	7	7	28	12977	714	2.183889018	0.013252522
GO:0006402	biological_process	mRNA catabolic process	8	7	21	12977	714	2.598926517	0.002540271
GO:0009057	biological_process	macromolecule catabolic process	4	9	64	12977	714	1.353814019	0.076646879
GO:0016071	biological_process	mRNA metabolic process	7	7	25	12977	714	2.34738775	0.007345932

GO:0034655	biological_process	nucleobase-containing compound catabolic process	5	7	44	12977	714	1.531812321	0.08864059
GO:0044265	biological_process	cellular macromolecule catabolic process	5	8	40	12977	714	1.861960923	0.022876557
GO:0006109	biological_process	regulation of carbohydrate metabolic process	5	7	21	12977	714	2.598926517	0.002540271
GO:0015672	biological_process	monovalent inorganic cation transport	7	10	73	12977	714	1.315992554	0.065124507
GO:0034220	biological_process	ion transmembrane transport	6	13	116	12977	714	1.026347741	0.097316815
GO:0042623	molecular_function	ATPase activity, coupled	8	18	153	12977	714	1.096426176	0.028093213
GO:0006754	biological_process	ATP biosynthetic process	11	7	30	12977	714	2.084353344	0.018774377
GO:0006818	biological_process	hydrogen transport	5	7	37	12977	714	1.781790574	0.043606581
GO:0009142	biological_process	nucleoside triphosphate biosynthetic process	8	7	41	12977	714	1.633691935	0.065124507
GO:0009144	biological_process	purine nucleoside triphosphate metabolic process	8	7	37	12977	714	1.781790574	0.043606581
GO:0009145	biological_process	purine nucleoside triphosphate biosynthetic process	9	7	31	12977	714	2.037047629	0.022087784
GO:0009199	biological_process	ribonucleoside triphosphate metabolic process	8	7	43	12977	714	1.564979185	0.079967721
GO:0009201	biological_process	ribonucleoside triphosphate biosynthetic process	9	7	37	12977	714	1.781790574	0.043606581
GO:0009205	biological_process	purine ribonucleoside triphosphate metabolic process	9	7	37	12977	714	1.781790574	0.043606581
GO:0009206	biological_process	purine ribonucleoside triphosphate biosynthetic process	10	7	31	12977	714	2.037047629	0.022087784
GO:0015985	biological_process	energy coupled proton transport, down electrochemical gradient	9	7	29	12977	714	2.133262945	0.01546075
GO:0015986	biological_process	ATP synthesis coupled proton transport	12	7	29	12977	714	2.133262945	0.01546075

GO:0015992	biological_process	proton transport	8	7	37	12977	714	1.781790574	0.043606581
GO:0046034	biological_process	ATP metabolic process	10	7	32	12977	714	1.99124394	0.023431018
GO:0016469	cellular_component	proton-transporting two-sector ATPase complex	3	7	32	12977	714	1.99124394	0.023431018
GO:0045259	cellular_component	proton-transporting ATP synthase complex	5	6	27	12977	714	2.013964016	0.040349436
GO:0015399	molecular_function	primary active transmembrane transporter activity	4	13	108	12977	714	1.129441234	0.064594937
GO:0015405	molecular_function	P-P-bond-hydrolysis-driven transmembrane transporter activity	5	13	108	12977	714	1.129441234	0.064594937
GO:0016820	molecular_function	hydrolase activity, acting on acid anhydrides, catalyzing transmembrane movement of substances	4	13	105	12977	714	1.170083218	0.055067899
GO:0019829	molecular_function	cation-transporting ATPase activity	12	10	52	12977	714	1.805377394	0.010708235
GO:0042625	molecular_function	ATPase activity, coupled to transmembrane movement of ions	11	10	53	12977	714	1.777896658	0.011880199
GO:0042626	molecular_function	ATPase activity, coupled to transmembrane movement of substances	10	13	104	12977	714	1.183889018	0.053668032
GO:0043492	molecular_function	ATPase activity, coupled to movement of substances	9	13	104	12977	714	1.183889018	0.053668032
GO:0044769	molecular_function	ATPase activity, coupled to transmembrane movement of ions, rotational mechanism	12	6	27	12977	714	2.013964016	0.040349436
GO:0046933	molecular_function	proton-transporting ATP synthase activity, rotational mechanism	13	6	26	12977	714	2.0684118	0.034620303
GO:0006996	biological_process	organelle organization	3	5	25	12977	714	1.861960923	0.095944027
GO:0051276	biological_process	chromosome organization	4	5	18	12977	714	2.335892111	0.033975276

GO:0009007	molecular_function	site-specific DNA-methyltransferase (adenine-specific) activity	6	3	7	12977	714	2.961496596	0.055067899
GO:0009008	molecular_function	DNA-methyltransferase activity	5	4	8	12977	714	3.183889018	0.011880199
GO:0006304	biological_process	DNA modification	7	12	37	12977	714	2.559398153	2.28E-05
GO:0006305	biological_process	DNA alkylation	8	7	14	12977	714	3.183889018	0.000147389
GO:0006306	biological_process	DNA methylation	9	7	14	12977	714	3.183889018	0.000147389
GO:0032775	biological_process	DNA methylation on adenine	10	3	7	12977	714	2.961496596	0.055067899
GO:0040029	biological_process	regulation of gene expression, epigenetic	6	7	14	12977	714	3.183889018	0.000147389
GO:0044728	biological_process	DNA methylation or demethylation	8	7	14	12977	714	3.183889018	0.000147389
GO:0042120	biological_process	alginic acid metabolic process	7	4	12	12977	714	2.598926517	0.041397839
GO:0042121	biological_process	alginic acid biosynthetic process	8	4	12	12977	714	2.598926517	0.041397839
GO:0006952	biological_process	defense response	3	6	19	12977	714	2.520924005	0.009511539
GO:0043570	biological_process	maintenance of DNA repeat elements	7	4	5	12977	714	3.861960923	0.00141531
GO:0043571	biological_process	maintenance of CRISPR repeat elements	8	4	5	12977	714	3.861960923	0.00141531
GO:0002252	biological_process	immune effector process	2	3	5	12977	714	3.446923424	0.023431018
GO:0002376	biological_process	immune system process	1	3	7	12977	714	2.961496596	0.055067899
GO:0009607	biological_process	response to biotic stimulus	2	3	5	12977	714	3.446923424	0.023431018
GO:0009615	biological_process	response to virus	4	3	5	12977	714	3.446923424	0.023431018
GO:0051607	biological_process	defense response to virus	5	3	5	12977	714	3.446923424	0.023431018
GO:0051707	biological_process	response to other organism	3	3	5	12977	714	3.446923424	0.023431018

Supplementary Table 6. Details of CRISPRs identified in the *Legionella* genomes.

Legionella Species	Confirmed CRISPR Counts	Questionable CRISPR Counts	No. of spacers	Best hit on CRISPR database	No. of spacers	Best hit on CRISPR database	No. of spacers	Best h on CRISI databa
L. pneumophila Alcoy	1	1	56	CRISPR-associated protein Cas1 tax=Proteobacteria	-	-	-	-
L. pneumophila Corby	0	2	--	--				
L. pneumophila Lens	2	1	52	Cas3_family CRISPR-associated helicase tax=Gammaproteobacteria	12	Cas3_family CRISPR-associated helicase tax=Gammaproteobacteria	-	-
L. pneumophila Philadelphia (Hextuple mutant)	0	0	-	-	-	-	-	-
L. pneumophila Paris	1	1	33	putative CRISPR-associated large protein (Provisional) tax=gamma	-	-	-	-
L. pneumophila Philadelphia	0	0	-	-	-	-	-	-
L .pneumophila Lorraine	0	1	-	-	-	-	-	-
L. pneumophila LPE509	0	0	-	-	-	-	-	-
L. pneumophila Thunderbay	0	0	-	-	-	-	-	-
L. pneumophila - 2 (D5762)	0	3	-	-	-	-	-	-
L. pneumophila - 3 (D5517)	0	0	-	-	-	-	-	-

L. pneumophila - 4 (D5739)	1	2	33	putative CRISPR-associated large protein (Provisional) tax=gamma proteobacterium HTCC5015	-	-	-	-
L. pneumophila - 5 (D5698)	2	1	24	CRISPR-associated helicase Cas3 tax=Legionella drancourtii LLAP12	70	Cas3_family CRISPR-associated helicase tax=Gammaproteobacteria	-	-
L. pneumophila - 6 (D5864)	0	0	-	-	-	-	-	-
L. pneumophila - 7 (D5178)	2	2	60	Cas3_family CRISPR-associated helicase tax=Gammaproteobacteria	32	putative CRISPR-associated large protein (Provisional) tax=gammaproteobacteria	-	-
L. pneumophila - 8 (D5744)	1	2	37	putative CRISPR-associated large protein (Provisional) tax=gammaproteobacteria	-	-	-	-
L. pneumophila - 9 (D4366)	0	2	-	-	-	-	-	-
L. pneumophila - 10 (D5602)	0	1	-	-	-	-	-	-
L. pneumophila - 11 (D797)	2	0	37	No hit	37	Crispr-associated protein Cas1 tax=Proteobacteria	-	-
L. pneumophila - 12 (D4955)	0	0		-	-	-	-	-
L. pneumophila - 12 (ATCC_43290)	0	0	-	-	-	-	-	-

L. pneumophila - 13 (D5677)	3	1	8	CRISPR-associated RAMP Csd1 family protein tax=Legionella drancourtii	32	CRISPR-associated helicase Cas3 tax=Legionella drancourtii LLAP12	55	Cas3_fa ly CRISP associa helicas tax=Ga maprote acteri
L. pneumophila - 14 (D4677)	3	1	7	putative CRISPR-associated large protein (Provisional) tax=gammaproteobacteria	5	No hits	14	No Hi
L. pneumophila - 15 (D4613)	0	0		-	-	-	-	-
L. pneumophila - 16 (D5564)	0	2		-	-	-	-	-
L. pneumophila - 17 (D4954)	1	1	25	putative CRISPR-associated large protein (Provisional) tax=gamma proteobacterium	-	-	-	-
L. anisa (D5641)	0	2	-	-	-	-	-	-
L. birminghamensis (D1407)	1	3	17	CRISPR-associated helicase Cas3 tax=Desulfonatronospira thiodismutans	-	-	-	-
L. erythra (D3308)	0	2	-	-	-	-	-	-
L. jamestowniensis (D4855)	0	1	-	-	-	-	-	-
L. wadsworthii (D4735)	0	1	-	-	-	-	-	-
L. nautarum (D4144)	0	1	-	-	-	-	-	-
L. micdadei (D5727)	0	1	-	-	-	-	-	-
L. cherii (D3084)	1	1	8	No Hits	-	-	-	-

L. bozemanii - 1 (D5751)	0	4	-	-	-	-	-	-
L. bozemanii - 2 (D4398)	1	4	6	No Hits	-	-	-	-
L. maceachernii (D5800)	1	10	22	Cas3_family CRISPR-associated helicase tax=Gammaproteobacteria	-	-	-	-
L. jordanis (D5875)	0	0	-	-	-	-	-	-
L. rowbothamii (D5054)	0	1	-	-	-	-	-	-
L. cincinnatiensis (D3325)	0	0	-	-	-	-	-	-
L. tusconensis (D1087)	0	0	-	-	-	-	-	-
L. brunensis (D1635)	1	1	19	CRISPR-associated protein Cas1 tax=Proteobacteria				
L. dumoffii (D5637)	0	1	--	--	-	-	-	-
*L. longbeacha*e NSW150	0	2	--	--	-	-	-	-

Supplementary Table 7. Core genes identified to have undergone homologous recombination. (**a**) Core genes that had undergone recombination identified by both NSS and MaxChi square methods. (**b**) Core genes that are predicted to have undergone recombination by PHI method only. (**c**) Results of the GO term enrichment analysis done on the core genes that showed evidence of recombination.

7a: Core genes that had undergone recombination identified by both NSS and MaxChi methods

Cluster No.	p-value of MaxChi	q-value of MaxChi	p-value of NSS	q-value of NSS	Gene Annotation
202	0	0	0	0	DNA protecting protein DprA
204	0.001	0.00890625	0	0	Methionyl-tRNA formyltransferase

206	0.001	0.00890625	0.008	0.009956332	hypothetical protein
215	0	0	0	0	Ribulose-phosphate 3-epimerase
216	0	0	0	0	Soluble lytic murein transglycosylase precursor
219	0	0	0	0	hypothetical protein
220	0	0	0	0	Leucine dehydrogenase
222	0.001	0.00890625	0	0	4-hydroxyphenylpyruvate dioxygenase
225	0	0	0.003	0.004061758	hypothetical protein
233	0.001	0.00890625	0	0	ankyrin repeat protein
235	0	0	0.001	0.001444867	Aspartate-semialdehyde dehydrogenase 2
236	0	0	0	0	Chorismate synthase
242	0	0	0	0	Glycerol-3-phosphate dehydrogenase [NAD(P)+]
246	0.001	0.00890625	0.002	0.002780488	Farnesyl diphosphate synthase
248	0.001	0.00890625	0	0	DNA utilization protein GntX
255	0	0	0	0	3-deoxy-D-manno-octulosonic acid transferase
259	0	0	0	0	2,4-dienoyl-CoA reductase [NADPH]
261	0	0	0.001	0.001444867	Oxygen-independent coproporphyrinogen-III oxidase 1
271	0	0	0	0	Aspartate--tRNA ligase
273	0.001	0.00890625	0	0	hypothetical protein
274	0.001	0.00890625	0	0	3-oxoacyl-[acyl-carrier-protein] reductase FabG
285	0.001	0.00890625	0	0	Uridylate kinase
293	0.001	0.00890625	0	0	8-amino-7-oxononanoate synthase
294	0.001	0.00890625	0	0	Biotin synthase
295	0	0	0	0	Adenosylmethionine-8-amino-7-oxononanoate aminotransferase
300	0	0	0	0	Single-stranded-DNA-specific exonuclease RecJ
301	0	0	0	0	hypothetical protein
305	0	0	0	0	ribosomal-protein-alanine N-acetyltransferase
308	0.001	0.00890625	0	0	hypothetical protein

321	0	0	0.002	0.002780488	Error-prone DNA polymerase
322	0	0	0	0	PhoH-like protein
331	0	0	0	0	chorismate pyruvate lyase
334	0	0	0	0	tetratricopeptide repeat protein
338	0.001	0.00890625	0	0	3-phosphoshikimate 1-carboxyvinyltransferase
341	0.001	0.00890625	0	0	Glycerol kinase
344	0	0	0	0	Adenylate kinase
347	0.001	0.00890625	0	0	Chloramphenicol resistance pump Cmr
348	0.001	0.00890625	0	0	putative sulfoacetate transporter SauU
349	0	0	0	0	Anhydro-N-acetylmuramic acid kinase
350	0	0	0	0	putative deoxyribonuclease YcfH
353	0	0	0.006	0.007616927	putative aminodeoxychorismate lyase
356	0	0	0.001	0.001444867	3-oxoacyl-[acyl-carrier-protein] reductase FabG
357	0	0	0	0	Malonyl CoA-acyl carrier protein transacylase
362	0	0	0	0	tRNA-specific 2-thiouridylase MnmA
364	0	0	0	0	L,D-transpeptidase catalytic domain
365	0.001	0.00890625	0	0	DNA polymerase III subunit epsilon
368	0	0	0	0	Urocanate hydratase
370	0	0	0	0	Quinolinate synthase A
371	0	0	0.008	0.009956332	L-aspartate oxidase
373	0	0	0	0	Dicarboxylic acid uptake system A
378	0	0	0	0	diaminopimelate aminotransferase
379	0.001	0.00890625	0	0	ComE operon protein 1
380	0	0	0.005	0.006426156	Rod shape-determining protein MreB
388	0	0	0	0	Putative glycosyltransferase EpsH
389	0.001	0.00890625	0	0	hypothetical protein
401	0	0	0.001	0.001444867	putative phospholipid ABC transporter permease protein MlaE

404	0	0	0	0	anti-anti-sigma factor
416	0.001	0.00890625	0	0	Cytochrome c-type biogenesis protein CcmH precursor
417	0	0	0	0	hypothetical protein
421	0	0	0	0	KHG/KDPG aldolase
426	0.001	0.00890625	0	0	Modulator of FtsH protease YccA
427	0.001	0.00890625	0.001	0.001444867	N-carbamoyl-D-amino acid hydrolase
430	0	0	0	0	Glutathione transport system permease protein GsiD
432	0	0	0	0	Putative pterin-4-alpha-carbinolamine dehydratase
438	0	0	0.002	0.002780488	Beta-ketoadipyl-CoA thiolase
439	0.001	0.00890625	0	0	Fatty acid oxidation complex subunit alpha
440	0	0	0.001	0.001444867	30S ribosomal protein S6
444	0	0	0.001	0.001444867	hypothetical protein
447	0.001	0.00890625	0	0	tRNA-modifying protein YgfZ
449	0	0	0	0	Acyl-CoA thioester hydrolase YbgC
452	0	0	0	0	translocation protein TolB
456	0	0	0	0	Pyruvate dehydrogenase E1 component subunit alpha
460	0	0	0	0	UDP-2,3-diacylglucosamine hydrolase
463	0	0	0	0	Nucleoside diphosphate kinase
464	0	0	0	0	Dual-specificity RNA methyltransferase RlmN
466	0	0	0	0	Cytoskeleton protein RodZ
467	0.001	0.00890625	0	0	Histidine--tRNA ligase
473	0.001	0.00890625	0	0	Poly(A) polymerase I precursor
474	0.001	0.00890625	0	0	integral membrane protein, YkoY family
477	0	0	0	0	Glutamate-1-semialdehyde 2,1-aminomutase
483	0.001	0.00890625	0.005	0.006426156	hypothetical protein
484	0	0	0.001	0.001444867	Alcohol dehydrogenase
486	0	0	0	0	General secretion pathway protein F
490	0	0	0.003	0.004061758	Hypoxanthine-guanine phosphoribosyltransferase

493	0	0	0	0	Penicillin-binding protein F
495	0	0	0	0	S-adenosylmethionine synthase
501	0	0	0.001	0.001444867	Uroporphyrinogen decarboxylase
506	0	0	0	0	Septum formation protein Maf
508	0	0	0	0	hypothetical protein
512	0	0	0	0	Tyrosine permease
513	0.001	0.00890625	0	0	DNA adenine methylase
514	0	0	0	0	putative sulfoacetate transporter SauU
515	0	0	0	0	putative sulfoacetate transporter SauU
517	0.001	0.00890625	0	0	50S ribosomal protein L31
521	0	0	0	0	Unfoldase HslU
523	0	0	0.001	0.001444867	Phosphopentomutase
528	0	0	0.001	0.001444867	Ribosomal large subunit pseudouridine synthase D
536	0	0	0	0	Nicotinate-nucleotide adenylyltransferase
548	0	0	0	0	Quorum-sensing regulator protein F
555	0	0	0	0	NifU-like protein
561	0	0	0	0	Arginine repressor
562	0	0	0.001	0.001444867	Arginine transport system permease protein ArtQ
563	0	0	0.003	0.004061758	Glutamine transport ATP-binding protein GlnQ

7b: Core genes that are predicted to have undergone recombination by PHI method only

Cluster number	p-value	q-value	Gene Annotation
304	0	0	GTP pyrophosphokinase
336	0	0	30S ribosomal protein S1
439	0	0	Fatty acid oxidation complex subunit alpha
570	0	0	Acyl-coenzyme A dehydrogenase
727	0	0	DNA polymerase I

731	0	0	hypothetical protein
737	0	0	Aminomethyltransferase
905	0	0	NADH-quinone oxidoreductase subunit L
1097	0	0	ATP-dependent helicase HepA
1151	0	0	Glutamate--tRNA ligase
1205	0	0	Bifunctional protein BirA
1318	0	0	conjugative coupling factor TraD, SXT/TOL subfamily
1329	0.001	0.07125	hypothetical protein
1336	0.001	0.07125	Cytochrome c oxidase subunit 2 precursor
1342	0.001	0.07125	DNA-directed RNA polymerase subunit beta'
1343	0.001	0.07125	DNA-directed RNA polymerase subunit beta

7c: Results of the GO term enrichment analysis done on the core genes that showed evidence of recombination

GOID	Ontology	Term	Level	q	m	t	k	log_odds_ratio	p
GO:0003674	molecular_function	molecular_function	0	95	5235	12977	129	0.868323813	2.94E-12
GO:0003824	molecular_function	catalytic activity	1	56	3902	12977	129	0.529798886	0.006148155
GO:0008150	biological_process	biological_process	0	96	5467	12977	129	0.820870867	1.03E-11
GO:0008152	biological_process	metabolic process	1	79	4493	12977	129	0.822759428	1.43E-08
GO:0044238	biological_process	primary metabolic process	2	44	2261	12977	129	0.969128368	5.35E-05
GO:0071704	biological_process	organic substance metabolic process	2	51	2451	12977	129	1.065712599	7.21E-07
GO:0009987	biological_process	cellular process	1	61	2986	12977	129	1.039180914	2.64E-08
GO:0016043	biological_process	cellular component organization	2	9	133	12977	129	2.767084592	0.000107375
GO:0022607	biological_process	cellular component assembly	3	7	61	12977	129	3.529059611	3.71E-05
GO:0030031	biological_process	cell projection assembly	4	2	14	12977	129	3.845087104	0.041034286

GO:0044085	biological_process	cellular component biogenesis	2	8	119	12977	129	2.757624263	0.000326032
GO:0071840	biological_process	cellular component organization or biogenesis	1	10	176	12977	129	2.514938503	0.000151644
GO:0071702	biological_process	organic substance transport	4	7	240	12977	129	1.552906353	0.041089704
GO:0005575	cellular_component	cellular_component	0	51	2157	12977	129	1.250056907	1.20E-08
GO:0005623	cellular_component	cell	1	42	1249	12977	129	1.758201687	2.44E-11
GO:0019867	cellular_component	outer membrane	2	3	48	12977	129	2.652442026	0.046771952
GO:0044464	cellular_component	cell part	2	42	1200	12977	129	1.815940759	1.08E-11
GO:0071944	cellular_component	cell periphery	3	11	273	12977	129	2.019116504	0.001033795
GO:0005215	molecular_function	transporter activity	1	12	488	12977	129	1.306667189	0.018909201
GO:0022892	molecular_function	substrate-specific transporter activity	2	8	293	12977	129	1.457685172	0.041034286
GO:0016860	molecular_function	intramolecular oxidoreductase activity	3	2	17	12977	129	3.564979185	0.046771952
GO:0005622	cellular_component	intracellular	3	31	931	12977	129	1.744000979	3.50E-08
GO:0005737	cellular_component	cytoplasm	5	28	572	12977	129	2.299925612	6.85E-11
GO:0044424	cellular_component	intracellular part	4	28	680	12977	129	2.050406012	3.22E-09
GO:0009056	biological_process	catabolic process	2	8	191	12977	129	2.075013198	0.005125094
GO:0044237	biological_process	cellular metabolic process	2	52	2220	12977	129	1.236537783	1.07E-08
GO:1901575	biological_process	organic substance catabolic process	3	8	177	12977	129	2.184836476	0.003467288
GO:0009058	biological_process	biosynthetic process	2	38	1051	12977	129	1.862822586	4.68E-11
GO:1901576	biological_process	organic substance biosynthetic process	3	36	961	12977	129	1.913974407	6.85E-11
GO:0006807	biological_process	nitrogen compound metabolic process	2	28	1493	12977	129	0.915798498	0.005125094
GO:0044281	biological_process	small molecule metabolic	3	16	658	12977	129	1.290498253	0.005948956

		process							
GO:0044710	biological_process	single-organism metabolic process	2	34	1380	12977	129	1.309452316	6.63E-06
GO:1901564	biological_process	organonitrogen compound metabolic process	3	18	587	12977	129	1.625150335	0.000279653
GO:0005488	molecular_function	binding	1	55	2476	12977	129	1.160006141	1.48E-08
GO:0043167	molecular_function	ion binding	2	26	1450	12977	129	0.85104456	0.012011927
GO:0043169	molecular_function	cation binding	3	16	612	12977	129	1.395054184	0.003430715
GO:0046872	molecular_function	metal ion binding	4	15	596	12977	129	1.340164101	0.006033956
GO:0016491	molecular_function	oxidoreductase activity	2	17	680	12977	129	1.330513931	0.00358055
GO:0055114	biological_process	oxidation-reduction process	3	18	712	12977	129	1.346633597	0.002478917
GO:0006082	biological_process	organic acid metabolic process	4	12	375	12977	129	1.686657742	0.003399423
GO:0019752	biological_process	carboxylic acid metabolic process	6	12	370	12977	129	1.706023067	0.003098852
GO:0043436	biological_process	oxoacid metabolic process	5	12	372	12977	129	1.698245716	0.003206965
GO:0048037	molecular_function	cofactor binding	2	9	281	12977	129	1.687940708	0.012011927
GO:0097159	molecular_function	organic cyclic compound binding	2	45	1879	12977	129	1.268545771	1.03E-07
GO:1901363	molecular_function	heterocyclic compound binding	2	45	1879	12977	129	1.268545771	1.03E-07
GO:0043170	biological_process	macromolecule metabolic process	3	30	1597	12977	129	0.918184025	0.003467288
GO:1901565	biological_process	organonitrogen compound catabolic process	4	4	69	12977	129	2.54391757	0.026407197
GO:0016651	molecular_function	oxidoreductase activity, acting on NAD(P)H	3	5	43	12977	129	3.548105366	0.00083767

GO:0016655	molecular_function	oxidoreductase activity, acting on NAD(P)H, quinone or similar compound as acceptor	4	5	22	12977	129	4.514938503	3.45E-05
GO:0046914	molecular_function	transition metal ion binding	5	9	240	12977	129	1.915476432	0.005125094
GO:0006520	biological_process	cellular amino acid metabolic process	7	9	269	12977	129	1.750904665	0.009346934
GO:0009064	biological_process	glutamine family amino acid metabolic process	8	6	70	12977	129	3.10812151	0.000844395
GO:1901605	biological_process	alpha-amino acid metabolic process	8	7	171	12977	129	2.041944433	0.010104
GO:0018130	biological_process	heterocycle biosynthetic process	4	12	458	12977	129	1.398200739	0.012055854
GO:0044249	biological_process	cellular biosynthetic process	3	36	891	12977	129	2.023085406	1.25E-11
GO:0044271	biological_process	cellular nitrogen compound biosynthetic process	4	14	460	12977	129	1.614306897	0.002056681
GO:1901362	biological_process	organic cyclic compound biosynthetic process	4	12	476	12977	129	1.342586764	0.015716449
GO:1901566	biological_process	organonitrogen compound biosynthetic process	4	10	359	12977	129	1.486530087	0.017357964
GO:0006412	biological_process	translation	6	16	131	12977	129	3.619019025	1.25E-11
GO:0009059	biological_process	macromolecule biosynthetic process	4	24	502	12977	129	2.265860973	4.13E-09
GO:0010467	biological_process	gene expression	4	24	358	12977	129	2.75358875	1.04E-11
GO:0016070	biological_process	RNA metabolic process	6	12	318	12977	129	1.924521572	0.000934491
GO:0019538	biological_process	protein metabolic process	4	17	586	12977	129	1.545148013	0.000844395
GO:0034645	biological_process	cellular macromolecule biosynthetic process	5	23	432	12977	129	2.42111648	1.39E-09

GO:0044260	biological_process	cellular macromolecule metabolic process	4	27	1235	12977	129	1.137034202	0.000844395
GO:0044267	biological_process	cellular protein metabolic process	5	16	382	12977	129	2.075013198	2.04E-05
GO:0006355	biological_process	regulation of transcription, DNA-dependent	8	9	212	12977	129	2.094446573	0.002632447
GO:0009889	biological_process	regulation of biosynthetic process	4	9	237	12977	129	1.933623779	0.004914073
GO:0010468	biological_process	regulation of gene expression	5	9	248	12977	129	1.868170717	0.005981529
GO:0010556	biological_process	regulation of macromolecule biosynthetic process	5	9	233	12977	129	1.958180883	0.004514031
GO:0019219	biological_process	regulation of nucleobase-containing compound metabolic process	5	9	261	12977	129	1.794461031	0.007882772
GO:0019222	biological_process	regulation of metabolic process	3	9	339	12977	129	1.417225565	0.034038916
GO:0031323	biological_process	regulation of cellular metabolic process	4	9	290	12977	129	1.642457938	0.014131788
GO:0031326	biological_process	regulation of cellular biosynthetic process	5	9	235	12977	129	1.945850081	0.004740367
GO:0051171	biological_process	regulation of nitrogen compound metabolic process	4	9	261	12977	129	1.794461031	0.007882772
GO:0051252	biological_process	regulation of RNA metabolic process	6	9	212	12977	129	2.094446573	0.002632447
GO:0060255	biological_process	regulation of macromolecule metabolic process	4	9	264	12977	129	1.777972908	0.008451261
GO:0080090	biological_process	regulation of primary metabolic process	4	9	311	12977	129	1.541596258	0.021330924

GO:2000112	biological_process	regulation of cellular macromolecule biosynthetic process	6	9	233	12977	129	1.958180883	0.004514031
GO:2001141	biological_process	regulation of RNA biosynthetic process	7	9	212	12977	129	2.094446573	0.002632447
GO:0003676	molecular_function	nucleic acid binding	3	29	1016	12977	129	1.521738335	3.25E-06
GO:0001071	molecular_function	nucleic acid binding transcription factor activity	1	6	109	12977	129	2.469220202	0.005603269
GO:0003700	molecular_function	sequence-specific DNA binding transcription factor activity	2	6	109	12977	129	2.469220202	0.005603269
GO:0003723	molecular_function	RNA binding	4	18	162	12977	129	3.482517025	4.67E-12
GO:0043933	biological_process	macromolecular complex subunit organization	3	4	27	12977	129	3.897554524	0.001502386
GO:0071822	biological_process	protein complex subunit organization	4	3	24	12977	129	3.652442026	0.010424403
GO:0006664	biological_process	glycolipid metabolic process	6	2	17	12977	129	3.564979185	0.046771952
GO:0009245	biological_process	lipid A biosynthetic process	8	2	17	12977	129	3.564979185	0.046771952
GO:0009247	biological_process	glycolipid biosynthetic process	7	2	17	12977	129	3.564979185	0.046771952
GO:0046493	biological_process	lipid A metabolic process	7	2	17	12977	129	3.564979185	0.046771952
GO:1901269	biological_process	lipooligosaccharide metabolic process	6	2	17	12977	129	3.564979185	0.046771952
GO:1901271	biological_process	lipooligosaccharide biosynthetic process	7	2	17	12977	129	3.564979185	0.046771952
GO:0006820	biological_process	anion transport	6	4	74	12977	129	2.442988661	0.032029922
GO:0015698	biological_process	inorganic anion transport	7	3	24	12977	129	3.652442026	0.010424403
GO:0008509	molecular_function	anion transmembrane transporter activity	5	4	58	12977	129	2.794461031	0.014934554

GO:0005886	cellular_component	plasma membrane	4	9	240	12977	129	1.915476432	0.005125094
GO:0090502	biological_process	RNA phosphodiester bond hydrolysis, endonucleolytic	8	2	11	12977	129	4.193010408	0.027001789
GO:0004521	molecular_function	endoribonuclease activity	6	2	11	12977	129	4.193010408	0.027001789
GO:0016891	molecular_function	endoribonuclease activity, producing 5'-phosphomonoesters	7	2	6	12977	129	5.067479526	0.009306897
GO:0016893	molecular_function	endonuclease activity, active with either ribo- or deoxyribonucleic acids and producing 5'-phosphomonoesters	6	2	12	12977	129	4.067479526	0.031201645
GO:0044248	biological_process	cellular catabolic process	3	6	136	12977	129	2.149941686	0.013937526
GO:0003735	molecular_function	structural constituent of ribosome	2	13	58	12977	129	4.494900749	2.94E-12
GO:0005198	molecular_function	structural molecule activity	1	14	68	12977	129	4.372334107	2.35E-12
GO:0030529	cellular_component	ribonucleoprotein complex	5	13	73	12977	129	4.163057186	1.58E-11
GO:0032991	cellular_component	macromolecular complex	1	13	224	12977	129	2.545526822	6.38E-06
GO:0005840	cellular_component	ribosome	7	13	71	12977	129	4.203134625	1.25E-11
GO:0043226	cellular_component	organelle	1	13	149	12977	129	3.133713224	5.96E-08
GO:0043228	cellular_component	non-membrane-bounded organelle	2	13	130	12977	129	3.330513931	1.37E-08
GO:0043229	cellular_component	intracellular organelle	5	13	148	12977	129	3.143428379	5.68E-08
GO:0043232	cellular_component	intracellular non-membrane-bounded organelle	6	13	130	12977	129	3.330513931	1.37E-08
GO:0044444	cellular_component	cytoplasmic part	6	14	125	12977	129	3.494012664	8.53E-10
GO:0019843	molecular_function	rRNA binding	5	10	43	12977	129	4.548105366	4.02E-10

GO:0006351	biological_process	transcription, DNA-dependent	8	7	148	12977	129	2.250343583	0.005309335
GO:0019438	biological_process	aromatic compound biosynthetic process	4	10	426	12977	129	1.239660501	0.041034286
GO:0032774	biological_process	RNA biosynthetic process	7	7	161	12977	129	2.12888007	0.007643556
GO:0034654	biological_process	nucleobase-containing compound biosynthetic process	5	9	342	12977	129	1.404514513	0.035710768
GO:0015935	cellular_component	small ribosomal subunit	9	2	7	12977	129	4.845087104	0.012011927
GO:0044391	cellular_component	ribosomal subunit	8	5	15	12977	129	5.067479526	4.76E-06
GO:0044422	cellular_component	organelle part	2	5	62	12977	129	3.020173811	0.003430715
GO:0044446	cellular_component	intracellular organelle part	6	5	34	12977	129	3.88690728	0.000279653
GO:0006525	biological_process	arginine metabolic process	9	5	16	12977	129	4.974370121	6.50E-06
GO:0006527	biological_process	arginine catabolic process	10	2	6	12977	129	5.067479526	0.009306897
GO:0009063	biological_process	cellular amino acid catabolic process	8	3	44	12977	129	2.777972908	0.041034286
GO:0009065	biological_process	glutamine family amino acid catabolic process	9	2	12	12977	129	4.067479526	0.031201645
GO:0016054	biological_process	organic acid catabolic process	5	4	57	12977	129	2.819552012	0.014131788
GO:0044282	biological_process	small molecule catabolic process	4	4	66	12977	129	2.608047907	0.022706149
GO:0044712	biological_process	single-organism catabolic process	3	4	66	12977	129	2.608047907	0.022706149
GO:0046395	biological_process	carboxylic acid catabolic process	7	4	57	12977	129	2.819552012	0.014131788
GO:1901606	biological_process	alpha-amino acid catabolic process	9	3	42	12977	129	2.845087104	0.041034286

GO:0006105	biological_process	succinate metabolic process	8	2	5	12977	129	5.330513931	0.00670064
GO:0019545	biological_process	arginine catabolic process to succinate	11	2	5	12977	129	5.330513931	0.00670064
GO:0005506	molecular_function	iron ion binding	6	4	71	12977	129	2.502694907	0.028541753
GO:0051536	molecular_function	iron-sulfur cluster binding	3	5	72	12977	129	2.80444512	0.005603269
GO:0051539	molecular_function	4 iron, 4 sulfur cluster binding	4	4	26	12977	129	3.952002308	0.001311188
GO:0051540	molecular_function	metal cluster binding	2	5	72	12977	129	2.80444512	0.005603269
GO:0015934	cellular_component	large ribosomal subunit	9	3	8	12977	129	5.237404527	0.000693369
GO:1901682	molecular_function	sulfur compound transmembrane transporter activity	3	2	12	12977	129	4.067479526	0.031201645
GO:0034622	biological_process	cellular macromolecular complex assembly	5	2	17	12977	129	3.564979185	0.046771952
GO:0065003	biological_process	macromolecular complex assembly	4	3	21	12977	129	3.845087104	0.007643556
GO:0006526	biological_process	arginine biosynthetic process	10	3	10	12977	129	4.915476432	0.001240911
GO:0009084	biological_process	glutamine family amino acid biosynthetic process	9	3	25	12977	129	3.593548337	0.011651528
GO:0003954	molecular_function	NADH dehydrogenase activity	4	4	17	12977	129	4.564979185	0.000279653
GO:0050136	molecular_function	NADH dehydrogenase (quinone) activity	5	4	17	12977	129	4.564979185	0.000279653
GO:0048038	molecular_function	quinone binding	3	3	16	12977	129	4.237404527	0.004303433
GO:0008137	molecular_function	NADH dehydrogenase (ubiquinone) activity	6	3	13	12977	129	4.536964809	0.002632447

Supplementary Data 1. CRISPR (consensus DR and spacer) sequences Identified in *Legionella* Genomes.

L. anisa (D5641)	**DR (consensus)**	**DR lengt h**	**No. of spacer s**	**Spacer sequences**	**Results of BLASTN search against the nr.nt database**	**Searching similarities between upstream and downstrea m regions and cas genes**	**putative confirm CRISPR**
1	TCAAGGTAGTGTTTT CTGCTAAC	23	1	>spacer3			
				GCCTCGAGCTCTTCCTTCACAAGTGAACATGCG CACAGATTGAGCTTTT	No hits	No hits	Putative
2	ATGTGAACCATTTGT GAACCATT	23	4	>spacer2			
				AATCACCACAAACAGAACTATTCTTGTGAGTCAT TAAAAATCACAAAATCGCATA	No hits	No hits	Putative
				>spacer3			
				GCTTTTATTTTATATAAATAATATCATGGTA	No hits		
				>spacer4			
				TGTGAACCATTTAATAGTGTTTTACGAACTACTA ACAACCAGTTTTAATAATACAATAAAACAAAT	No hits		
				>spacer5			
				TCTACAAATTAAATCGAACATATTAATAAATAAA GATGTTATAAAAATGGCAAGA	No hits		
L. pneumophila - 2 (D5762)	**DR (consensus)**	**DR lengt h**	**No. of spacer s**	**Spacer sequences**	**Results of BLASTN search against the nr.nt database**	**Searching similarities between upstream and downstrea m regions and cas**	**putative confirm CRISPR**

						genes	
1	AAATTACTGTTATGG CGGTAAATTT	25	2	>spacer1	No hits	No hits	Putative
				GATAAAAATAAATTTATTATTGCATTTCTTTGCAT TAATTCACCAT			
				>spacer2			
				GATTGATTTCCTGATTTTCCCATATTTATTTATTA TAAATTCCTAA			
2	CCTGGCAGCAGCGC TGATCAGCATTCCAA AAAGC	34	1	>spacer1			
				GTCACCACGCTTAACTTGAGCGACAATGGCCTT TATCAACTAAGCGCTGATCG	No hits	No hits	Putative
3	ATTTTCCAATTTTATC GACATTTTTTA	27	1	>spacer1			
				AGAATATGTCGATGCGGTAGACATGTCAAGGAA ATGTGTCGAT	No hits	No hits	Putative
L. pneumophila - 3 (D5517)	**NO CRISPR SEQUENCES IDENTIFIED**						
L. pneumophila - 4 (D5739)	**DR (consensus)**	**DR length**	**No. of spacers**	**Spacer sequences**	**Results of BLASTN search against the nr.nt database**	**Searching similarities between upstream and downstream regions and cas genes**	**putative confirm CRISPR**
1	GGCTCTTCCCCATTT CCGGGGGCACTAAT CAAGAAC	36	1	>spacer1			
				ACAAAACTTTAACCCTTAACCTATAA	No hits	No hits	Putative

2	TAGATTAATTTGTTTG ACTGTTGTTTGATT	30	1	>spacer1			
				GGTTGACAACTTTTTTATTGCATTTTCTGTAAAA AGCTAGGTTTTACAAGATGTTAGT	No hits	No hits	Putative
3	GTTTCAGTGGTTGGA TTTTTAGATGAGGGA TTATTGG	37	33	>spacer1		putative CRISPR-associated large protein (Provisional) tax=gamma proteobacterium HTCC5015	Confirme
				AGTCACACAAATTAATTCAAATGCTATGTGCA			
				>spacer2			
				ATCGTTAGAAACGCTAGAGGCGGCAATCAATAC CA			
				>spacer3			
				GGCAAAACCAAAGAGGTATGATTCATGACTGTA TA			
				>spacer4			
				ATGTCAAAGAGATGCCCAATAGCGTTAACTTAA C			
				>spacer5			
				AAAAACTGGTTTGATAGATCACGTTAATCGTTTT TC			
				>spacer6			
				TCTTTGCGCTTCATAGCAGTATCAGCAAGCAAG TT			
				>spacer7			
				CCGGAGGCGTTTCAGGAAACGTCACAGGTTATA CTTA			
				>spacer8			
				ATGTGCTAATATGTTCAGTGGCAGCGATATCTA CATA			

				>spacer9			
				GGTGTGTAAGCGACATCGAATAACACTTCAGTAGCAAT			
				>spacer10			
				TGTATTGCTAACAGGCAATCCGAATCCGTTATAGC			
				>spacer11			
				GGTTTAACTGTTGCAAATAGTGGAACTGCGACTAA			
				>spacer12			
				GATCAGGTTAAAGGATATATTTAAATGGAAATCAC			
				>spacer13			
				GTGGCGTGGTGATTAATGCGTCACCGTAGCCTGC			
				>spacer14			
				TTGTTGGTAGATTGACATCAATTTGTAAGTTAGA			
				>spacer15			
				CAAAGCAGAACAATTAAATCAAAGACGGATCTTGA			
				>spacer16			
				GCCGACGGAGGTTGATTCGGACGCTTTGCAAT			
				>spacer17			
				TTGTTGGTAGATTGACATCAATTTGTAAGTTAGA			
				>spacer18			
				GGCAGCTAATTCCGAGTATAACCCTAAATATTGTG			
				>spacer19			
				TTAGGCGCGGGAGTAGATACTTCTATTACAATAAACG			
				>spacer20			
				TTGATAAATTAATGTCAGGTTATGAGCCATCGAA			
				>spacer21			

				CTTTATCAAGGTGGTAATTGATTGAGGTTTTTA			
				>spacer22			
				TTATAATACTTGTCCACGTTGTGAACTTACTATTG			
				>spacer23			
				CAACCCTATTAAATTACTAAAAACTAGCCCTTTA			
				>spacer24			
				CTATGATCCTAACCTGGGTAGAGAAGTCACTAGT			
				>spacer25			
				GCTTAGAACTAGATGCAAAGCGTAAGCTTATTGGA			
				>spacer26			
				GTTGGACTTGTGTACAGATTGACTTTAGCCAGTTAG			
				>spacer27			
				ACTGAAACGATGACCTTGGAGCAATATCTAAAAT			
				>spacer28			
				TAAGACGTTTATTCAGAATGAACGGCAATATCAAA			
				>spacer29			
				AAAGAATATTATGAAATTACTAAAAAACGAATAG			
				>spacer30			
				GTTACAATACCCGACTTTCATTTATCACCTGTATTTCAAA			
				>spacer31			
				GGTATTTCAATCATTTTAGTGGATTTGATTTTTA			
				>spacer32			
				ACCGTCCACGCTGTCTTGCGTGAATCGACACTCCA			
				>spacer33			
				TAGAAAGTGCCCAAGGAACACGAGAGACAGCC			

				GA			
L. pneumophila - 5 (D5698)	**DR (consensus)**	**DR length**	**No. of spacers**	**Spacer sequences**	**Results of BLASTN search against the nr.nt database**	**Searching similarities between upstream and downstream regions and cas genes**	**putative confirm CRISPR**
1	GTCGCGCCCCGCGCGGGCGCGTGGATTGAAAC	32	24	>spacer1		CRISPR-associated helicase Cas3 tax=Legionella drancourtii LLAP12	
				TTTAGCTCTTGATTGTGACTATCCTCTGTTTGTCGG			
				>spacer2			
				CAGTTGCTTGTAAAGTGCCTAGTCCAACGTCTGG			
				>spacer3			
				TCTATTGTAAGAAAATACTAACAAATGCCTGTTG			
				>spacer4			
				ATTAAAAAGCTGTCAACTGGAAATTGATATTTT			
				>spacer5			
				TAAGCCTAAATTAATCGTAAAATTGTTACAAGAAA			
				>spacer6			
				TATATAAAAATATGGTGTGTCTTGCACTTGGCC			
				>spacer7			
				ATTATACGGACCTAATAATACAGTTGCACCAGC			

				AC			
				>spacer8			
				CAGTTCGTATGGTAGCGGGGGACGCCCTAGACGC			
				>spacer9			
				ATTTAAACGATGATGAGTTATTGCTTTTAGACTT			
				>spacer10			
				AAAGCTCGAAGCAATCAGTTAAACATTGACATCAA			
				>spacer11			
				AATTTATCTTTTATTGCGTAAGTGTTACCTAAGACT			
				>spacer12			
				TCTATTGTAAGAAAATACTAACAAATGCCTGTTGAT			
				>spacer13			
				TTATAAAAGCAGGGATGCAGACGTATAAAGATGCTA			
				>spacer14			
				AAATGAATTTCATCATTTGATTGAGAGTAGAAGGCA			
				>spacer15			
				ATAAAGCATCTATAGCAACCCAATCCTTGTTTAT			
				>spacer16			
				ATCGAATAGCTCATTTACAACCCGGGCAGTTTT			
				>spacer17			
				CTTGCTCTGTTGAAATGCAAAAGGCTGTGTCATT			
				>spacer18			
				CACAAAGGGCATTCAATACCGGCATGATGCTTTT			
				>spacer19			
				ACTTTGATTGGGTTCTCTGGCTAGTAAGTGCTTA			

				>spacer20			
				ACTTTGATTGGGTTCTCTGGCTAGTAAGTGCTTA			
				>spacer21			
				TAAATTCTACATTTTGTAAATAAGTTTCTAAAAT			
				>spacer22			
				TTTAGGAGACTGTATTGCTCCTGGAGACGGATA TA			
				>spacer23			
				AGCTTTTGTATGTGCGCTGAATAAAGCGAAAAC AGC			
				>spacer24			
				TGCCACTGGATTACCAGCAATTGGAACAGGAGA G			
2	GTTCACTGCCGTACA GGCAGCTTAGAAA	28	70	>spacer1		Cas3_family CRISPR-associated helicase tax=Gamma proteobacter ia	
				ATATTGGCGTTTTAAAATAAACCGTTACGTCC			
				>spacer2			
				AAAACTGCCACCATCATTAAACTGTACTTGTG			
				>spacer3			
				AATTGCCGGTAATCCTGTAGCAGATGTTAAAG			
				>spacer4			
				AGGATATGCTGAATTTAAAGAAGACAGCGATA			
				>spacer5			
				TATAGCAGCAGCAACTACTCCGACAAATGGCT			
				>spacer6			
				CCTGATAATAGAACGCTTAATCAACATCGTTC			

				>spacer7				
				ATCAATTAATTAGCCTATTAGAATCGAAACCA				
				>spacer8				
				GCTAACCGTGGAAAAAGAACTGTCATCATCGA				
				>spacer9				
				TTTGAGAAAAACCCGCTGCCGCTTGTAGACGG				
				>spacer10				
				TAAGCAGGCTTTTTAGTTCTTTAATTGCGTCC				
				>spacer11				
				AGGAAATGCTTAACATGACCCTAAAACGTCT				
				>spacer12				
				TTGCCATCGCCTTCCGATACGGGCAAGCGTGA				
				>spacer13				
				CACGCATTAAAACCCAACGCCGCATGAAAA				
				>spacer14				
				AAGCGCGAAAGACCTAGGGTTACGGATGTTTT				
				>spacer15				
				ACCAAATATAAATGCTCAACGGTGCAAGATGT				
				>spacer16				
				CACTAAGTTAGGGGATTGCTGGTTACTTGGCG				
				>spacer17				
				GCGAAACTTCCAAAGAATTTGAAGACCTAGGG				
				>spacer18				
				CCGGTAAGCAATGCCGCCAATAGATACAGAAT				
				>spacer19				
				GATTTCAGGTGTTCCGGCATTGTTCGACACGA				
				>spacer20				
				ATATACGGTAAAGACCAATATGGTAATGACGT				

				>spacer21			
				ATGCCAAACCGCGCACCCGTCAACGGCGTTGC			
				>spacer22			
				TCTTTAAATAGCAAGTCGCCGTTTTCATCAAC			
				>spacer23			
				CCCTGTACCCGAAGCCGCCTCGCCTATGCTTAT			
				>spacer24			
				ACCACCGCTTTAGCGAATATGAATGTCGAGGA			
				>spacer25			
				GCACAAGGAGTAAGTACAACTTTTCGTTTGG			
				>spacer26			
				TTGCAGAGCGGCTCGGCAAAACCTTAAAGCAG			
				>spacer27			
				TTGCAGAGCGGCTCGGCAAAACCTTAAAGCAG			
				>spacer28			
				AAGATACACAGGTCGGCGGCATTCAAAGCGAT			
				>spacer29			
				GAACAGCTGGTATAATTTTTATAAACCTAGAT			
				>spacer30			
				TATCCAGTTTGTGATTCGTGGTGTTCCTTCGT			
				>spacer31			
				TCAAAAAGGTCCTGCTGTTGAGTTGCCTTTGG			
				>spacer32			
				CGCCGCCTAAAGGGTCAATAAAACCAACATCT			
				>spacer33			
				ATACAGATAAAAGTGATGCTAAAGTTCACGGC			
				>spacer34			
				TGTAGGATTCGGCTCTAATTCAATTAAACTATG			

				>spacer35			
				CAAGTACCTTTTGATGCCTGAAATACGGAGTG			
				>spacer36			
				TATTTGATGAGCTTCTTCAAATTCTGGATATT			
				>spacer37			
				TTTGTGCTTAGCTTCTAATTCATTTCTTATACT			
				>spacer38			
				TTGGATATCTTGCTCGCTTTGATTATGTACAT			
				>spacer39			
				GGCAAGGTCTTTAATGCCTTTGCCTTTGCTGA			
				>spacer40			
				TTGTGGTCAGTGTATTGGTTGTAAGTTACGCA			
				>spacer41			
				TCCTATTGATCGTATTAAAGCTCTTGGTGATG			
				>spacer42			
				TCATCCAAGTCAACGACTAATATGGGTAAGCC			
				>spacer43			
				CTACAATGGCCTGCGATAATTGTTCCTGCCGA			
				>spacer44			
				TGCTGGATTGTGCCCCAAGGCAAACAGAGACA			
				>spacer45			
				TATTGGTGTAACTTTAAACACCATATCAGAAT			
				>spacer46			
				TAAGAAAATATAAAATAGCTGGCTGTGTAACG			
				>spacer47			
				TTGGTATAGACGAAAGTAGCTGGCGATATCAT			
				>spacer48			
				AATTCATCATTAAATAAACAGTTGGCTCTTG			

				>spacer49			
				AACTCACGCCCAACAATATCAGCAGCTTTATA			
				>spacer50			
				ATCGTCACCGGACCTAAAATACCATCCACTGC			
				>spacer51			
				GTGGGGAGTTAATCTAATGAAACTATTACCCT			
				>spacer52			
				TCAACTATTTGTTCAATCGGCTTAGGCTTGTC			
				>spacer53			
				ATAGAGACAAGAAACTTCAATCATTGCTTTAT			
				>spacer54			
				TTGCACGCTGGACACCTAATCATTTCCATTTC			
				>spacer55			
				TTGAGTCATGATGTATGATTTATGATGAATAA			
				>spacer56			
				AGAAGCAAAAACAACACGGCCTTGAGTATCT			
				>spacer57			
				AAGTGTACGGAGATAGACCGCAAGACGACAC			
				>spacer58			
				AGTAGTGCTGGTCAAGCTCGTCATATGTTAAG			
				>spacer59			
				CATACATCTGGCCTGTATAATAATTATTGCTC			
				>spacer60			
				CAGAAAGCCCGATAATTGCGTTTAAAGCATCG			
				>spacer61			
				TTGTTTCCATTTCCTAAATGTACATTCTCCTT			
				>spacer62			
				AGTGTTCAATAAAAATGAACAAGTTAAACAAA			

				>spacer63			
				CAGCATGGACAGAGAAGAATCTGTACATACAA			
				>spacer64			
				CAGCATGGACAGAGAAGAATCTGTACATACAA			
				>spacer65			
				TTCTCAAGTTGGTGCTCCTATGCAAACTGGTG			
				>spacer66			
				TTATACTGGACCTGATAAGCGTATTAAAAAGA			
				>spacer67			
				TCTAATATTAGCTAGTATGTGGGCAATAACGT			
				>spacer68			
				TTTGCACACTGGACATCTAATCATTTCCATTT			
				>spacer69			
				GGTTGTAAAGACTGGTAAAAGAGCTATATATATA<br>TA			
				>spacer70			
				AATGTGCCAAGTAACGTGCAGAATACAGTTAA			
3	AAATTTACCGCGATA<br>ACAGTAATTT	25	2	>spacer1			
				TATGAATTTAACATAAATAAATACACTGAAAATTA<br>GAAAGCCGAGT			
				>spacer2			
				ATGGCGAATTAATGCAAAAAAATGCAATAATAAA<br>TTTATTTTAATC			
L. pneumophila - 6 (D5864)	**NO CRISPR SEQUENCES IDENTIFIED**						
L. pneumophila - 7 (D5178)	**DR (consensus)**	**DR lengt h**	**No. of spacer s**	**Spacer sequences**	**Results of BLASTN search against**	**Searching similarities between upstream**	**putative confirm CRISPR**

					**the nr.nt database**	**and downstream regions and cas genes**	
1	AAATTACTGTTATGG CGGTAAATTT	25	2	>spacer1		No Hits	hypothet protein
				GATAAAAATAAATTTATTATTGCATTTCTTTGCAT TAATTCACCAT			
				>spacer2			
				GATTGATTTCCTGATTTTCCCATATTTATTTATTA TAAATTCCTAA			
2	TAAGACGTCTCACTC TGCTCAAATGAAATC TGACTCCTCGTCGAC C	46	1	>spacer1		No Hits	Copper Oxidase
				TCAATGAACAAGAGCATGGCCATGCCGGGCAT GAATCATAGCGCAATGGGCCAGAA			
3	TTTCTAAGCTGCCTG TGCGGCAGTGAAC	28	60	>spacer1		Cas3_family CRISPR-associated helicase tax=Gammaproteobacteria	
				GCATAAAACGCCCAGCGTATTCTATCGCAAAA			
				>spacer2			
				GATGAGCATAAAATGCAGTTAGCTGCTTATCA			
				>spacer3			
				CAAAGCCATGTCATGAACTTGTGGGGGCGAAT			
				>spacer4			
				CTGGGCTTTCTGGGTCGAGTCCCGCGCGAACT			
				>spacer5			
				GACTATCATTTTCGCTGCCTTTATCTCCCTTT			

				>spacer6			
				GCTTTCACACAAACTAAGAACTATCCGATGCA			
				>spacer7			
				AGTCGTCAGGGAATTGGAATAGGCACGTCCAA			
				>spacer8			
				AGCTATTCTATAAAGTAGCTCGTCTCCTTCCT			
				>spacer9			
				TGTATGGAATAGCGGCTAGACAAGCTCCTTGC			
				>spacer10			
				TACAGATAATTGTCTAGTGTCTCTTTTGCAGA			
				>spacer11			
				AAACTGTGGCGGAGTGGGGAGAAACATGTGGA			
				>spacer12			
				TACCATCTCGTTCATGCTACGGAAAGATTAAG			
				>spacer13			
				TAATTGACAATCCCACAGTTTGCCCCCTCTTA			
				>spacer14			
				TGTTAAATAAATATTTAGCAATTGGCTATTTG			
				>spacer15			
				GCTCGCCAAGTTGCCAAACCCAAGCTTTTAGA			
				>spacer16			
				GGCAACTTAAAAATGTTATGCACCGATTACA			
				>spacer17			
				AAGAGCATCATTGCCCCCAGTAGTTGGGATGA			
				>spacer18			
				TTTTCGCAAATGTCCGTTGTTTTTCCAATAA			
				>spacer19			
				AAGTGGGCTTCATGCATCTCATTTAACTTATT			

				>spacer20			
				AAAACTCTATGGCAATCGGGTGCTGGTCTAT			
				>spacer21			
				ATTTTATAAGCGGCCATATCTAAAAGTTTTTTG			
				>spacer22			
				TAGTTAATGGTGTTTGCGTGGTTTTTTTA			
				>spacer23			
				TCATGGTTTAAACATTTATTATTGTCTATTAA			
				>spacer24			
				AGGAGATAGAATTAATGTGAATTGTGCACTGT			
				>spacer25			
				TTGAACCGTTCTTGCCCTTTTTTGACTTTCA			
				>spacer26			
				CTCCACAAGATTTCTAATCTCGTCCCCAATTTG			
				>spacer27			
				AAAAACGAGTTGTGGGCATGGCGTCCCACGAA			
				>spacer28			
				AAATTATAACTAATTAATTATAAAACGTCAAG			
				>spacer29			
				TAAAAGATAAAAATGGAAAAGAGATTTATGAA			
				>spacer30			
				ATAATACACACATATACAAAAGTTGTGCTACA			
				>spacer31			
				CATTTCCTACCCTTTCTCTTATAAATCATTGT			
				>spacer32			
				AATACACTCGACACAATCCCAATTTTTTTGT			
				>spacer33			
				AATGCCCCATTGTTGGAAATTTTCTACAAGTT			

				>spacer34				
				AATCTAGGCGGGGGACGATTTAACTATACTAG				
				>spacer35				
				AAAATATGGCGTGTCCTGCACTTGGCCAGTTT				
				>spacer36				
				TAGACAATACTGTTATATTAGCCCCAGATGCA				
				>spacer37				
				AACATCTAACTCTTTTACAACACTTTCTATGT				
				>spacer38				
				ATTTTACAAATTTAACCAGAGTTGGTTTAACA				
				>spacer39				
				ATCAGCACTACAGACAGCGGTTTTATTCAGTG				
				>spacer40				
				TCTCAGCAATTTTTTGATACACTTCGTCGATT				
				>spacer41				
				ATTATTTAAGAAGCCTAAATAAATTAAAAGAT				
				>spacer42				
				ATAAGAGGCTGTCGCTGTTCACTTCTGTGCGT				
				>spacer43				
				TGCTACTTTAAGAGTAACTGGTGAACCAGCGT				
				>spacer44				
				GCCTTACCAGCAATCACTGGCGGGGGAAATGA T				
				>spacer45				
				TGCAGAAGAATTAATGTCCACAGATTACATTG				
				>spacer46				
				AGAGTTTTTAGGTGCGGTTCGCCAAGGAAATA				
				>spacer47				
				GTCCAACGTAAGTTGGTTCGTAATCTTGATGA				

				>spacer48			
				AGTAAAAAGAGTTAAATTTTCAGGCGAAGAGT			
				>spacer49			
				GAGATAGTGCTCTCGTTGGATTACAGTGGTGA			
				>spacer50			
				GTGAAGGTGTACATATTTCATCTACAGACCAA			
				>spacer51			
				CTATTCAAGAGTCCCTAGTTCTTGATTCTTTA			
				>spacer52			
				AAGATCCCCATGGGATTTGGATCGCAAATGGT			
				>spacer53			
				TTAGCAATCCACAAGCCGTATGGATCTTTATT			
				>spacer54			
				ATTCTTTGTTTAATTCATCAAATAACCAATCT			
				>spacer55			
				AACGTATTAGGCAATTGAGATACAGAGAGACT			
				>spacer56			
				CTGCGCTGGCTTCTCCGTTTAATCCATATGAT			
				>spacer57			
				AATGGCATGTTGAGACTGTCTCTGCTCGAGTG			
				>spacer58			
				TTATCAATGTGTATGGTACGGGGTCTGGTGAA			
				>spacer59			
				TTTTCTCGTATGCGATATTCCAGTAAGGACAA			
				>spacer60			
				AGGTCGGCGACATTTACTAATTGCTTCGTTAA			
4	CCAATAATCCCTCAT CTAAAAATCCAACCA	37	32	>spacer1		putative CRISPR-	

	CTGAAAC					associated large protein (Provisional) tax=gamma	
				CTTTATCAATTGTAAGATTGAAATATTCGTCCAT T		proteobacter ium HTCC5015	
				>spacer2			
				AATAGCCCAGACCCTGAAAAAGTTAGAGATCGA G			
				>spacer3			
				CGGATCCATTTTCCTGTTAGCTGGACAGTATGT TT			
				>spacer4			
				GAAAAACATGTCTTAAAATCCGTTGACGAGGCT A			
				>spacer5			
				ATAGCTCCAGGTAGGGAACTTAAAAAACTAAAC AA			
				>spacer6			
				ATCATACACTAAAGCCATTGTCTATTAATCTCTT A			
				>spacer7			
				ATTGCGGATTTGAAAGACCAAACAATGTTTTTAA			
				>spacer8			
				TCTAAAAAACTTCGTTTTGTGTCTTTTGACATC			
				>spacer9			
				TGAAGAGAAAGGCTGGACACATTGTTGTATGTT			
				>spacer10			
				TAATTTTCACTACCCCTCCCTGATTTACAGACA			
				>spacer11			
				GCAATTACTCTCCGGCGGCCAGTAATGCAATTG AT			
				>spacer12			

				TCTGGTAACTTACAGTTTTCGCAAATGTCCGTTG TT			
				>spacer13			
				AAGAGAATTAAGTTTGATCCACCTGTTGGACTA G			
				>spacer14			
				GACAAGTAGCTATGTTTTTTGTCTTAAGAGTTTA TC			
				>spacer15			
				AGACCAAAAAATTATATTATAATAACCTTCTGGC TT			
				>spacer16			
				ACTTTTTATGCTTATTTTCCAAGTGCTTTAGATTT			
				>spacer17			
				TTCTTAAATCGCCCATCTTCAACTTTTTGTTCTAT			
				>spacer18			
				TTGACATTATCGTTGAGACCTTCACACTGATTGC T			
				>spacer19			
				TTATGAGTTGCCAACTAACTTTCGGAATATGGAC T			
				>spacer20			
				TATTACCAAAAAGGCGGTAAGTCATGGAACTGA GA			
				>spacer21			
				TTCCAAGAATCATGGCATCAGTTTCTTTCTCTGG			
				>spacer22			
				CTTATCAATCATTTAATCGGTGTGCAAAGACAAA ATAC			
				>spacer23			
				TTTCCAACCATGGAGTGGTTTATGCAATCAGAT GA			
				>spacer24			

				ACCTCCGGTTTAATTCTATAATCCTTTTTGAG			
				>spacer25			
				AATTATGGAAAATTTGAGAGAGTTGGCACTGTTTTG			
				>spacer26			
				TAAATTACACCTAGATTGTCATGCATTCTAGTATAT			
				>spacer27			
				CTTTATTGCAGTTTTTAACCGCGCAATAGTGCGCG			
				>spacer28			
				AACTAGAAGAACTAACGCATCCTAAGCCTAAGTATAA			
				>spacer29			
				GTTAAATCAACACGGTTACATCTAATCAGTATACTCTA			
				>spacer30			
				CACTAGATGTAAAATTTGCTGGTCACTAATAAA			
				>spacer31			
				TCAAGATCGGCTGTTGTGCCTGATTCTCCAAGTC			
				>spacer32			
				GTCGCATCGTTTTCATGGTCGTAGCCTGACTTCTT			
L. pneumophila - 8 (D5744)	**DR (consensus)**	**DR length**	**No. of spacers**	**Spacer sequences**	**Results of BLASTN search against the nr.nt database**	**Searching similarities between upstream and downstream regions and cas genes**	**putative confirm CRISPR**
1	GTTTCAGTGGTTGGA	37	9	>spacer1		putative	

	TTTTTAGATGAGGGA TTATTGG					CRISPR-associated large protein (Provisional) tax=gamma proteobacter ium	
				AAGAAGTCAGGCTACGACCATGAAAACGATGCG AC		HTCC5015	
				>spacer2			
				TTTCTTTAAGCTTTTAAAACGCTTTTCACATACA			
				>spacer3			
				CTTTTAGCATTCGAGATTTTGATGATGAAATGGA AGT			
				>spacer4			
				TTCAAAGAAAGTTAATCCCGTTGAGTGGGTTAG GAT			
				>spacer5			
				ATACTTTGTGGGTTTTAGTAGAGGAGCCTTTCTT GTC			
				>spacer6			
				GTTGAGATCTTTTGAAGTAGTGGAAAAGGGGGA TGT			
				>spacer7			
				TTAAGGATAAACAGACAAGCCTTTTTTGCTGAGA AA			
				>spacer8			
				TTCAAGAATGCGGGCAATGTGTCAATTGCATTA C			
				>spacer9			
				TGTAGATGATGGTTTTCAAGTGGCAATTGTTCAA GA			
2	CCTGGCAGCAGCGC TGATCAGCATTCCAA AAAGC	34	1	>spacer1			

				GTCACCACGCTTAACTTGAGCGACAATGGCTTT TATCAACTAAGCGCTGATCG			
3	AAATTACTGTTATGG CGGTAAATTTACTCG A	31	2	>spacer1			
				AATAAATTTATTATTGCATTTTTTTGCATTAATTC ACCAT			
				>spacer2			
				TTTTCTAATTTTCTGTGTATTTATTTATGTTAAATT TATA			
L. pneumophila - 9 (D4366)	**DR (consensus)**	**DR lengt h**	**No. of spacer s**	**Spacer sequences**	**Results of BLASTN search against the nr.nt database**	**Searching similarities between upstream and downstrea m regions and cas genes**	**putative confirm CRISPR**
1	AAATTTACCGCGATA ACAGTAATTT	25	2	>spacer1			
				TATGAATTTAACATAAATAAATACACTGAAAATTA GAAAGCCGAGT			
				>spacer2			
				ATGGCGAATTAATGCAAAAAAATGCAATAATAAA TTTATTTTAATC			
2	CCTGGCAGCAGCGC TGATCAGCATTCCAA AAAGC	34	1	>spacer1			
				GTCACCACGCTTAACTTGAGCGACAATGGCCTT TATCAACTAAGCGCTGATCG			
L. pneumophila - 10 (D5602)	**DR (consensus)**	**DR lengt**	**No. of spacer**	**Spacer sequences**	**Results of BLASTN**	**Searching similarities**	**putative confirm**

		h	s		search against the nr.nt database	between upstream and downstream regions and cas genes	CRISPR
1	CAGGTTAATGATGCTGGTGGTGTGTTGGG	29	1	>spacer1			
				CCAGCAGTACCAGCAGCATTTGACGGCTTAT			
L. pneumophila* - 11 *(D797)	**DR (consensus)**	**DR length**	**No. of spacers**	**Spacer sequences**	**Results of BLASTN search against the nr.nt database**	**Searching similarities between upstream and downstream regions and cas genes**	**putative confirm CRISPR**
1	CCAATAATCCCTCATCTAAAAATCCAACCACTGAAAC	37	14	>spacer1		No hit	
				TCTAAATTCGACAAAAATAACAAAAAACAAATTTGGA			
				>spacer2			
				AAAATTGTGTTCCGCTGCTCGACAAAATTATTTAA			
				>spacer3			
				GGTAAGTAAAAGGTGAATTAATACCATAAATGCA			
				>spacer4			
				TGGGACTGTCCATCCATAATCAATTGGGCCAATT			
				>spacer5			
				TTCTACCGATATTTCCCACTATACCTATATCATAAAG			
				>spacer6			
				TTCGAAATTAGTGTATTAGTCATAGCTAATAGTG			

				T			
				>spacer7			
				GACTTAGTAGAAGAGGAAACTAGAGGAAAAACTAAAT			
				>spacer8			
				CACTTAGAACATAGCTTTGATTCTGTTGGTGTG			
				>spacer9			
				AGACATGCTAATCCTTTTAGGCCATATCAATGCC			
				>spacer10			
				ACAAAACTAGTCAAATGCCCCGCACCAGACATGGCA			
				>spacer11			
				TTTTTATATAATAATCCTATTCTGCTAGGTTTAATT			
				>spacer12			
				ATCCTGTAATATTGTCGAATACGTAATTAATCTCAGG			
				>spacer13			
				ACTTAGCCTCAATTCCCAAACCTCTGTGGCCGATAC			
				>spacer14			
				ACTTTATTAGATATGACTAATATATTAATAGCCAA			
2	CCAATAATCCCTCATCTAAAAATCCAACCACTGAAAC	37	6	>spacer1		Crispr-associated protein Cas1 tax=Proteobacteria	
				GGGGATACCAACGATCTAGAATTTTTTGTACTTTAA			
				>spacer2			
				CACCATAGATGTTACGTTAGATCAAAGTCATTTAGG			
				>spacer3			

				AAAAGCTTGGGTCTGGCAACTTGGCGAGCAGTATG			
				>spacer4			
				TAGTAATACATCCGGCCCATTATGTCACAAATAAT			
				>spacer5			
				GTTTGTCACTCGCGGCACTCCTTCATTGTCGTAGTT			
				>spacer6			
				TTACATCTAAGGCAGTCTACCCCAGTACACGGTGA			
L. pneumophila - 12 (D4955)	**NO CRISPR SEQUENCES IDENTIFIED**						
L. pneumophila - 13 (D5677)	**DR (consensus)**	**DR length**	**No. of spacers**	**Spacer sequences**	**Results of BLASTN search against the nr.nt database**	**Searching similarities between upstream and downstream regions and cas genes**	**putative confirm CRISPR**
1	GTTTCAATCCACGCGCCCGCACGGGGCGCGAC	32	8	>spacer1			
				CAGGACGCACAGAAGAATTAAAGAAGTGTATTTG		CRISPR-associated RAMP Csd1 family protein tax=Legionella drancourtii	
				>spacer2		LLAP12	

				CGATTGAGCCTTGCCTAGCGGCGGACGAACAT CT			
				>spacer3			
				GCACTGCCAATAAAAAGACTATACATATAAGAGT A			
				>spacer4			
				ATTGGTTAGGAAAACAAGTTAGAATATTTATTAA AA			
				>spacer5			
				TTATATCATGTTTTTGTGACATCATCCCGTTCGG			
				>spacer6			
				TTATCGCAGGGACGATCTGCCCCCAAACGAATA CGGC			
				>spacer7			
				GAAAAGTTTTATACCGAGTATGAAAAACTAAGTG			
				>spacer8			
				ATTTGTAAATAGGAGTTAGTTATGTCATTGATGC T			
2	GTTTCAATCCACGCG CCCGCACGGGGCGC GAC	32	48	>spacer1		CRISPR-associated helicase Cas3 tax=Legionella drancourtii LLAP12	
				ACCTTGCAGAAGTGTGTTTATTCCTTACAAAAAT			
				>spacer2			
				AAGTAATATTTATGAAAACCCTGAACTATTAACT			
				>spacer3			
				GTAGAGTTAACTAGATGCAGTAATACAATGAGC			

				>spacer4			
				TTTAATTTTTGTACTAACAATTAGTTTAATTAAT			
				>spacer5			
				AAAACACTACAAGATATAATCACCCATCTTGAGG			
				>spacer6			
				CTTGCGCACATGCACTAAAAGTTAACAAGTATAT AA			
				>spacer7			
				GCACAGAGTTCTTTACCATCAAAACCAATATAGC TA			
				>spacer8			
				GTTTAGAGTGTTGTGTACCTTTAAATGATAAACA T			
				>spacer9			
				CGCTCATGTACTTCTAAGCCGCCTACCGCAGCA			
				>spacer10			
				CTTTGATAACTTGTATCGTCAAAATGTACACCAT A			
				>spacer11			
				TTGCCCAAGTTCCATCCCCTCGCCAAAATGTGC TA			
				>spacer12			
				AGCCTAAATCATGTAAATCTGCTATGTGGTCATA A			
				>spacer13			
				ATCAACATAATAATTAATCTGTTTTAACGATAAT			
				>spacer14			
				TGCAGTGCCAAAATAATTATTAGCACCTGATATT			
				>spacer15			
				AAGTTATGATGATTATAATGGTTGGAACCCTGC G			
				>spacer16			

				GCGTATCACATGGGGCTACAGTTGCCGATGTGT GCTA			
				>spacer17			
				ACAGTAAAGAAGCAAATGTTTATATATACGCTCC T			
				>spacer18			
				ATGGTCAAGGGTGGGTTTTTGGCATACATTATC CA			
				>spacer19			
				TATTAGCTTTTGGATTACTTGGATTTTTAGTGTTT			
				>spacer20			
				ACGGCTAAAGGCCACGAAGGAGAATATAATTTA GTA			
				>spacer21			
				AGCAAATCGCACAACATAATTGTAGATTTTAAAA C			
				>spacer22			
				TATATGAAAAGTACACATCTATTAATAATATTTTA			
				>spacer23			
				TTCTGTACTTAAAAAGCCAGTGCTAGGTAAAGA AA			
				>spacer24			
				ATTTCAACATCTGTATTAAAAAATCCATACTAATC			
				>spacer25			
				ATTTAGTGGTGGAGTTAGACCACCTTATGTTATA TT			
				>spacer26			
				TAGAATTAATAGGCAATTAGGAGTAGATTATGAA T			
				>spacer27			
				GTTGTGTCTGCATATAAGCGCATAAAATCAATG CTT			
				>spacer28			

				ATTATGGATGCATGCACGGCGGTATAAATGCAACA			
				>spacer29			
				AAAGCAGAAAAGGCGTTAAGTACGGCATATCAAA			
				>spacer30			
				GTGATTAATCCGCAGCATCTTTTCAAAGAATCTT			
				>spacer31			
				AAAAGACTACGAACAGAACCTATAAACTAAACAA			
				>spacer32			
				AAGACAAATTGAGAGACTTATAAATAATTCGGTT			
				>spacer33			
				CCCGTTTTTGTTTTATTGGTATTCACATTTTCAT			
				>spacer34			
				GCACAAAAAGCGGTAAAGTTGAGCCTTTTGTCAT			
				>spacer35			
				TTAATCAGGCTTATGAAGTATTAAAAGACTTAAGC			
				>spacer36			
				CAGTGATTACAGTTTTCATACCAGCAGACGCGAT			
				>spacer37			
				AGAGCTTGGCAGTTATTTCAAATACAATCAATCA			
				>spacer38			
				ATGCTAGCTAACTACATTGTTAACGTAACACAGG			
				>spacer39			
				AATAAAAGTTGCAAAAGTATTAAACAAAACTAAT			
				>spacer40			
				ATGGCTTAGTGGCGTTTAGCGTTACGTTTAATGG			

				>spacer41			
				CTTCCTACTCCACCGATGTCATCAGTGAATTCAA AA			
				>spacer42			
				TGCACCAGCTCTCGAATGTCACGCTCATCAGTA A			
				>spacer43			
				ATGCTAGCTAACTACATTGTTAACGTAACACAG G			
				>spacer44			
				CCAGTTTGGCAATCAAGCCTATAGAACGTTTGT T			
				>spacer45			
				TGATAGAATTGACATCAGCACCAACAACCAAGA TT			
				>spacer46			
				TTGAGAATAGATGCAAAAAATGCAGAAGGTATG TGT			
				>spacer47			
				GGCAATACCGCCCGTTATCTCGCAGACCTTGCC GA			
				>spacer48			
				TGCAAAAATTCCTTATAGGATATACATCGTCATT CA			
3	ATTGATCTAAATCAA GAATTAAAT	24	4	>spacer1		NO hits	Hypothe Protein
				ATCAATCTAAATCAAGCATTAAAT			
				>spacer2			
				ATTGATCTAAATCAAGAATTAAAT			
				>spacer3			
				ATTGATCCAGATCAAAGA			
				>spacer4			
				ATTAATAGATACCGGTTTGGTTTATTTTCTAAAC CAGTGAAT			

4	TTTCTAAGCTGCCTG TACGGCAGTGAAC	28	55	>spacer1		Cas3_family CRISPR-associated helicase tax=Gammaproteobacteria	
				TTAACTGTATTCTGCACGTTACTTGGCACATT			
				>spacer2			
				TATATATATATAGCTCTTTTACCAGTCTTTACAACC			
				>spacer3			
				AAATGGAAATGATTAGATGTCCAGTGTGCAAA			
				>spacer4			
				ACGTTATTGCCCACATACTAGCTAATATTAGA			
				>spacer5			
				TCTTTTTAATACGCTTATCAGGTCCAGTATAA			
				>spacer6			
				CACCAGTTTGCATAGGAGCACCAACTTGAGAA			
				>spacer7			
				TTGTATGTACAGATTCTTCTCTGTCCATGCTG			
				>spacer8			
				TTTGTTTAACTTGTTCATTTTTATTGAACACT			
				>spacer9			
				AAGGAGAATGTACATTTAGGAAATGGAAACAA			
				>spacer10			
				CGATGCTTTAAACGCAATTATCGGGCTTTCTG			
				>spacer11			
				GAGCAATAATTATTATACAGGCCAGATGTATG			
				>spacer12			

				CTTAACATATGACGAGCTTGACCAGCACTACT			
				>spacer13			
				GTGTCGTCTTGCGGTCTATCTCCGTACACTTT			
				>spacer14			
				AGATACTCAAGGCCGTGTTGTTTTTTGCTTCT			
				>spacer15			
				TTATTCATCATAAATCATACATCATGACTCAA			
				>spacer16			
				GAAATGGAAATGATTAGGTGTCCAGCGTGCAA			
				>spacer17			
				ATAAAGCAATGATTGAAGTTTCTTGTCTCTAT			
				>spacer18			
				GACAAGCCTAAGCCGATTGAACAAATAGTTGA			
				>spacer19			
				TTTGTCTCTCCTTATTTCTAAAATATTCTTAG			
				>spacer20			
				CAATATAAATCACATTTACTATTTTCTTGAGT			
				>spacer21			
				ATATTGAAAAAGAGATTGAGTTACGATTAGCT			
				>spacer22			
				TTTTAAGTACAGAAATGTAACCTTTATTTTT			
				>spacer23			
				GTGATACAACTGAGCGTGGGATTGGTGGATTT			
				>spacer24			
				TAAGAATTATCGAACATATTTTGGTACTATTG			
				>spacer25			
				AATATGATGTATAGCGTTCCCACTCTGAGAAA			
				>spacer26			

				ATTCTCCTAAAGCTAAAAAGCCCTCGCAATGA				
				>spacer27				
				AGAGCAGCCCAAGTTTAGAACACGGATGGAGT				
				>spacer28				
				CAGGGTTTGAATATATGTTTATATGGAGCAAA				
				>spacer29				
				TATGTACGGCGGTATAAATGCAACAGAAGAAT				
				>spacer30				
				TTTATTTAGGCAATCATGAACGTAAGTATAAA				
				>spacer31				
				GGTGCTATGGATATGCTGGTGGCTCCGTTACA				
				>spacer32				
				AGTACATTTTGTCAATAACTTTTCATCTTATA				
				>spacer33				
				ACTTTATTATAAGATAATCAGTCAACTATTAA				
				>spacer34				
				TTTTAGCACTATAATTCATGTTTCTTGAATT				
				>spacer35				
				AAAGAAACCCAGCCAGTAACAATGATCCAGCA				
				>spacer36				
				TTCTGTAATTCCTACAACTCCAAATGGCTATA				
				>spacer37				
				ATAGTTTTGCATCAGTAAGGATTCCATACACT				
				>spacer38				
				TCTGATGGTCGAACAAATGACATATCTCTAAA				
				>spacer39				
				TAATAAAATAGCAGTAGGGTAGCATAGGGGAA				
				>spacer40				

				TAGATTATATAGCTAGAGCTTTAGGATTTGGA			
				>spacer41			
				TTAGCTCTGATCTTGGAGTTACACAATTAGCAT			
				>spacer42			
				AAATTTAGATGTGCGCCATGCTGCATATATGCT			
				>spacer43			
				ACATAGCAAGCCAAGCATGTAAAGGTACTGCT			
				>spacer44			
				CGTTGGATAAGTGCAGACATGCTGGTGAGATA			
				>spacer45			
				GGATTAGTGAAGAAAGTTTCGCATCGTCATAT			
				>spacer46			
				ATTTCCCCCATTGAAAATACACAAAAGGAGCA			
				>spacer47			
				TTTCATAATCCTCCACTGTGGTGTAGACCTGG			
				>spacer48			
				GCAAGCCTAGATATGAAGTAATTGGGGCAAAT			
				>spacer49			
				ATATTAACTGTATCCGATGTTAGTGGGTCTAT			
				>spacer50			
				TGAATGTGCTGGTATTTCTTGCTCTATTTCAC			
				>spacer51			
				GGGATAATGTTGGTAACTATGTGCCAGCAGAC			
				>spacer52			
				GTATGATTAAAGCATTTGCAAATAGCCCTAGC			
				>spacer53			
				GATTTTCTCCTGTATTAGTTAAATCTGTTTTA			
				>spacer54			

				ATCTTCTTCCATATTTCGCAAACTTATAGAAT			
				>spacer55			
				ACTTTAGCAAGTACAGCTAAAGAATATCGCAT			
L. pneumophila - 14 (D4677)	**DR (consensus)**	**DR length**	**No. of spacers**	**Spacer sequences**	**Results of BLASTN search against the nr.nt database**	**Searching similarities between upstream and downstream regions and cas genes**	**putative confirm CRISPR**
1	GTTCTTGATTAGTGCCCCCGGAAATGGGGAAGAGCC	36	1	>spacer1		putative CRISPR-associated large protein (Provisional) tax=gamma	
				TTATAGGTTAAGGGTTAAAGTTTTGT		proteobacterium HTCC5015	
2	GTTTCAGTGGTTGGATTTTTAGATGAGGGATTATTGG	37	7	>spacer1			
				CAACCCTATTAAATTACTAAAAACTAGCCCTTTA			
				>spacer2			
				CTATGATCCTAACCTGGGTAGAGAAGTCACTAGT			
				>spacer3			
				GCTTAGAACTAGATGCAAAGCGTAAGCTTATTGGA			
				>spacer4			
				GTTGGACTTGTGTACAGATTGACTTTAGCCAGTTAG			
				>spacer5			

				ACTGAAACGATGACCTTGGAGCAATATCTAAAA T			
				>spacer6			
				TAAGACGTTTATTCAGAATGAACGGCAATATCAA A			
				>spacer7			
				CAGATTTAGATGCTTTATACGATTGCTTAGCATA			
3	GTTTCAGTGGTTGGA TTTTTAGATGAGGGA TTATTGGA	38	5	>spacer1		No Hits	
				GTCACACAAATTAATTCAAATGCTATGTGCA			
				>spacer2			
				TCGTTAGAAACGCTAGAGGCGGCAATCAATACC A			
				>spacer3			
				GCAAAACCAAAGAGGTATGATTCATGACTGTAT A			
				>spacer4			
				TGTCAAAGAGATGCCCAATAGCGTTAACTTAAC			
				>spacer5			
				AAAACTGGTTTGATAGATCACGTTAATCGTTTTT C			
4	GTTTCAGTGGTTGGA TTTTTAGATGAGGGA TTATTGG	37	14	>spacer1		No Hits	
				CCGGAGGCGTTTCAGGAAACGTCACAGGTTATA CTTA			
				>spacer2			
				ATGTGCTAATATGTTCAGTGGCAGCGATATCTA CATA			
				>spacer3			
				GGTGTGTAAGCGACATCGAATAACACTTCAGTA			

				GCAAT			
				>spacer4			
				TGTATTGCTAACAGGCAATCCGAATCCGTTATAGC			
				>spacer5			
				GGTTTAACTGTTGCAAATAGTGGAACTGCGACTAA			
				>spacer6			
				GATCAGGTTAAAGGATATATTTAAATGGAAATCAC			
				>spacer7			
				GTGGCGTGGTGATTAATGCGTCACCGTAGCCTGC			
				>spacer8			
				TTGTTGGTAGATTGACATCAATTTGTAAGTTAGA			
				>spacer9			
				CAAAGCAGAACAATTAAATCAAAGACGGATCTTGA			
				>spacer10			
				GCCGACGGAGGTTGATTCGGACGCTTTGCAAT			
				>spacer11			
				TTGTTGGTAGATTGACATCAATTTGTAAGTTAGA			
				>spacer12			
				GGCAGCTAATTCCGAGTATAACCCTAAATATTGTG			
				>spacer13			
				TTAGGCGCGGGAGTAGATACTTCTATTACAATAAACG			
				>spacer14			
				TTGATAAATTAATGTCAGGTTATGAGCCATCGAA			
L. pneumophila - 15 (D4613)	**NO CRISPR SEQUENCES**						

	IDENTIFIED						
***L. pneumophila* - 16 (D5564)**	**DR (consensus)**	**DR length**	**No. of spacers**	**Spacer sequences**	**Results of BLASTN search against the nr.nt database**	**Searching similarities between upstream and downstream regions and cas genes**	**putative confirm CRISPR**
1	CGAGAGCATTGATGAACAGGATTTGAATGTTCGCAC	36	1	>spacer1		No hits	
				TTTATAAAGCAAGAGACACAATACAAGCTACAAC			
2	TCCCAAGCAAATTATATATAATTATAGCCATATTTTTG	38	1	>spacer1			
				GATTTACTCAGGCCGGTGCACGTGCTATCAATA			
***L. pneumophila* - 17 (D4954)**	**DR (consensus)**	**DR length**	**No. of spacers**	**Spacer sequences**	**Results of BLASTN search against the nr.nt database**	**Searching similarities between upstream and downstream regions and cas genes**	**putative confirm CRISPR**
1	CTGATTGAGAGGTGATACCCAAATTGCCTGTAAGAT	36	1	>spacer1			
				ATATTCTACATTTGGGACTTCGATTGTTCTTATGTG			
2	GTTTCAGTGGTTGGA	36	25	>spacer1		putative	

	TTTTTAGATGAGGGA TTATTG					CRISPR-associated large protein (Provisional) tax=gamma proteobacterium	
				GGCCTACACAGAGATATACAGAGTTATGCCGAC AG		HTCC5015	
				>spacer2			
				GACCCCATTCGCTGCGCTTCGAATCATTTCTTT CGA			
				>spacer3			
				TTCATTATCGTAAATTACCTACATATAATCCTATT A			
				>spacer4			
				TGTGCGTGGAAATGCTAAATTTTGATTTTCGGG AT			
				>spacer5			
				TTGTCTTTATAATTTATATACTTTGTGCTTAATTC T			
				>spacer6			
				ATATACAAAATTAACCTTAGAGGTTATTTACTTG			
				>spacer7			
				TTTTACTAAAAATTTAGAGGCTGGATATGAGAT			
				>spacer8			
				ATACATTATACCTTTCCATGTAGGTTTTAATACC G			
				>spacer9			
				AGCAGAACTAGGTGTAAATAAGCCCGTTTAAAC			
				>spacer10			
				TTTTGCTGCACAAAATGCAACTATTCATTAATAA AC			
				>spacer11			

				TATAGCTTTGGAGCCATGGTTTCTAATATGTCAC			
				>spacer12			
				AGTTTTGGCGTGATTTGGGCAAGTTCCGACACG GT			
				>spacer13			
				GTAGCACGGTAAGTTAGTAACCATGTTGTATTTA C			
				>spacer14			
				ATCTGGGCAATTTCCATCTTGATGGGGCTTATG G			
				>spacer15			
				GTCAATAATAAATTGGAAAACTGAACATAGAAA			
				>spacer16			
				TTTAACTGTCAATAAAGCCTTACCTGCAATTACA GG			
				>spacer17			
				ACCACTCTAAAGCAGATATCCTCATCATACCTT			
				>spacer18			
				TGCGTAGAGAAAGACGCCTATCAATATGTGTTA AA			
				>spacer19			
				GTGTCGTCGTCTGCTTGTATGCGCCATACATCA AG			
				>spacer20			
				CATTAAAGCTACGGGCTTTTGTGTAGGGTGAAC AGA			
				>spacer21			
				TTGAAATGGGGAGAGAACAGTTCCAAGCAGAGA CAT			
				>spacer22			
				AATTGAAACGGCGTTACGTAATCATTTATTTACA T			
				>spacer23			

				ATAGTTTATTGTGTTGTGGAAATTATAGTTTAT			
				>spacer24			
				TTAAAGCGATTAGAGCAACGATAATAAAACAGTAGT			
				>spacer25			
				AATCAATTAGGAGCGTGACCATGATTCTTATTTTT			
L. birminghamensis (D1407)	**DR (consensus)**	**DR length**	**No. of spacers**	**Spacer sequences**	**Results of BLASTN search against the nr.nt database**	**Searching similarities between upstream and downstream regions and cas genes**	**putative confirm CRISPR**
1	GCGGTCGCAGCCGCGGGAATTCGAGCGGTCGCAGCCGCGGGAATTCGA	48	1	>spacer1			
				GCGGTCGCAGCCGCGGGAATTCGATGCGGTCGCAGCCGCGGGAATTCGAGCGGTCGCAGCCGCGGGAATTCGATGCGGTCGCAGCCGCGGGAATTCGAT			
2	GTTTTCCCGCTCCGAATCCCCGCGG	25	1	>spacer1			
				TCGCAGCCCATAGCTATCTACTCAAATG			
3	TCGTTGCCGCGGGGATTCGGATG	23	1	>spacer1			
				GTCGTTGCCGCGGGGATTCGGATGGTCGTTGCCACGGGATTCGGATGA			
4	GTCGCGCCCCATGCGGGCGCGTGGATTGAAAC	32	17	>spacer1		CRISPR-associated helicase	

						Cas3 tax=Desulfo natronospira thiodismuta ns	
				TTTTGTAAATATTGGTATTCGAATCAAAAGAATTT		ASO3-1	
				>spacer2			
				CCAGTTTTCAGTATCAAACTCGCTGGCTACACT AAT			
				>spacer3			
				ATTTTTAACTCCCCCCAGACTATAGATAACCATA AT			
				>spacer4			
				TGTAGCATCTGATAAAACCCTTACTTTGCCAGA			
				>spacer5			
				ATGTCATAGTGAATTAATATCATGAGTATATGCC AA			
				>spacer6			
				GCTACTCCTTAATCTCTTCTCTCATTTTAAGTA			
				>spacer7			
				AACGATGAAACTAGGGCGTTGCCCGTCTGAGG TT			
				>spacer8			
				AGTATCCGCTCAAAATAGAGTTAGATATTATTG			
				>spacer9			
				AAGTTAAAAATGCCTGAAGGTACCAAAGACCAG C			
				>spacer10			
				AAGAATGTGCCACAGAATAGTACTTTTTCAGGA			
				>spacer11			
				TTGTTCTGTCAGTGTCACGCTATATAAGTCGGC			
				>spacer12			
				TTTTTGCTTGAACAAATTAAGTGCTGCAGCTTG			

				>spacer13			
				AAATTTGGAACTCTGAATGTACCAACTCCACCA G			
				>spacer14			
				TAAAAAACCAATGGTATTTGCAAGACTATATCGA ATA			
				>spacer15			
				ATTATAATGTACACCGTCATAATGAAAATCAAGG			
				>spacer16			
				AAGCTGTGGCGTGTTCTGCTGTTGCTCACCTAA AAG			
				>spacer17			
				GTTTAATTTTAACTTTACATTCGTGCCCATTATGT C			
L. erythra (D3308)	**DR (consensus)**	**DR lengt h**	**No. of spacer s**	**Spacer sequences**	**Results of BLASTN search against the nr.nt database**	**Searching similarities between upstream and downstrea m regions and cas genes**	**putative confirm CRISPR**
1	TTGTTGGGAAGTAGA GCCAAATT	23	1	>spacer1			
				GACTGAAAGCGCCCCCCTCGGACAAATCAAGC TCATCCTCCGAATCAAGGCC			
2	GTAGCAAAAGTGTCC CGCCGCTTTCATATT CTCGCG	36	1	>spacer1			
				CACCATTGGTGTGTTGATTCATGCACGTATGTAT TCTGAACAAGTCGTGGCAT			
L. jamestowniensis	**DR (consensus)**	**DR**	**No. of**	**Spacer sequences**	**Results of**	**Searching**	**putative**

(D4855)		**lengt h**	**spacer s**		**BLASTN search against the nr.nt database**	**similarities between upstream and downstrea m regions and cas genes**	**confirm CRISPR**
1	CCCCGTTACCGCCGT GAAAGGGCGATGTC CTAGGCCTCTAGACG ATGGGGACC	53	1	>spacer1			
				TGGAACTTAAATTATATACTATTTTTACGCCAATA ATGGCGTCCCCAAGGGGATTCGAAC			
L. wadsworthii (D4735)	**DR (consensus)**	**DR lengt h**	**No. of spacer s**	**Spacer sequences**	**Results of BLASTN search against the nr.nt database**	**Searching similarities between upstream and downstrea m regions and cas genes**	**putative confirm CRISPR**
1	AAATTTACCGCGATA ACAGTAAT	23	2	>spacer1			
				TTTTAGGAATTTATAATGAATAAATAGGGAAAAA TCAGGAAATCAATC			
				>spacer2			
				ATATAGTGAAATAATCCAAAGAAATGCAATAATA AATTTATTATTTTG			
L. nautarum (D4144)	**DR (consensus)**	**DR lengt h**	**No. of spacer s**	**Spacer sequences**	**Results of BLASTN search against the nr.nt database**	**Searching similarities between upstream and downstrea m regions**	**putative confirm CRISPR**

						and cas genes	
1	CTTAACTTTTTCCTGTAAACGTCACAT	27	1	>spacer1			
				CTCATCAAATATTTTTAAATATAAACTAATCGATCGAAAAGGAGAGTTAAGTGA			
L. micdadei (D5727)	**DR (consensus)**	**DR length**	**No. of spacers**	**Spacer sequences**	**Results of BLASTN search against the nr.nt database**	**Searching similarities between upstream and downstream regions and cas genes**	**putative confirm CRISPR**
1	CTCTCCCCCGCACAAAGTGTGGGGGAGAGGGTTAGGGAGAGGGGG	45	1	>spacer1			
				GAACTTCTCTCCTCCCACGTACAAAGTACGGAGAAAAAAATCAGAAAAAACCTTTCCC			
L. cherii (D3084)	**DR (consensus)**	**DR length**	**No. of spacers**	**Spacer sequences**	**Results of BLASTN search against the nr.nt database**	**Searching similarities between upstream and downstream regions and cas genes**	**putative confirm CRISPR**
1	ATCTGAATCCGATTTATCGAAAAAAACCCACGCGTTCAAGGATGCACTCGAAG	53	1	>spacer1		No Hits	
				CGGCTAGTGCGGGTACTGGCATAGCAGCAAAAAA			
2	GGGCATTTAACGATT	25	8	>spacer2		No hits	

	GCAGTAGTTT						
				ACAAACCTGTACCCTTCCGCAAAGCCTCAAATC GATTAACAAGG			
				>spacer3			
				ACAAACACTCGACATCCCTGAAGGGGTCACCTC TATTGGGGCAA			
				>spacer4			
				GCGAAGCATCAACGTCCCTGTAGGGGTTACCTC TATTAAGGAAT			
				>spacer5			
				ACAAAACATCAGCATCCCTGAAGGGGTCGAGTC GATTGAAGATG			
				>spacer6			
				ACAAAACATCACCCTTCCCCAAAGCCTCAAGTA TATTAGTCGGG			
				>spacer7			
				GCAAAACATTACCATCCCCGACGGGATTACTCG TATCAACGTTA			
				>spacer8			
				GCAAAACATCACTCTCCCCGAAGGAGTAACCAT TATCTTAGACG			
				>spacer9			
				GAAAACTTTGATCGTCCCTGAAACGGTCAACAG GATTGATTCTG			
L. bozemanii - 1 (D5751)	**DR (consensus)**	**DR lengt h**	**No. of spacer s**	**Spacer sequences**	**Results of BLASTN search against the nr.nt database**	**Searching similarities between upstream and downstrea m regions and cas genes**	**putative confirm CRISPR**
1	CGTGTTAGGGTGCTG TGTTGTTAA	24	1	>spacer1			

				CTGGTGAGTGCCCCCGTAATTGGGAAAGACCC CAGAACT			
2	TCACCATTTGAAGTA GTAAAACAA	24	1	>spacer2			
				TCACTGAAAACTACGAAGCAACCATCTGTGTGG AGCATACCTTCATGGTAC			
3	GCGCGTCAAGAAGTA ACAAATGCTGAAGCC CAAATTCA	38	1	>spacer1			
				CTCATTGACCACATTGCTCACTGAA			
4	CAAATTTACCGCGAT AACAGTAATTT	26	2	>spacer1			
				TTACGAATTTATAATAAATAAATATGGGAAAATC AGGAAATCAAT			
				>spacer2			
				ATGGTGAATTAATGCAAAGAATGCAATAATAAA TTTATTTTTAT			
L. bozemanii - 2 (D4398)	**DR (consensus)**	**DR length**	**No. of spacers**	**Spacer sequences**	**Results of BLASTN search against the nr.nt database**	**Searching similarities between upstream and downstream regions and cas genes**	**putative confirm CRISPR**
1	GCGCGTCAAGAAGTA ACAAATGCTGAAGCC CAAATTCA	38	1	>spacer1			
				CTCATTGACCACATTGCTCACTGAA			
2	TCACCATTTGAAGTA GTAAAACAA	24	1	>spacer2			
				TCACTGAAAACTACGAAGCAACCATCTGTGTGG AGCATACCTTCATGGTAC			

3	TTAACAGCACAGCACCCTAACACG	24	1	>spacer1			
				AGTTCTGGGGTCTTTCCCAATTACGGGGGCACTCACCAG			
4	CAAATTTACCGCGATAACAGTAATTT	26	2	>spacer1			
				TTACGAATTTATAATAAATAAATATGGGAAAATCAGGAAATCAAT			
				>spacer2			
				ATGGTGAATTAATGCAAAGAAATGCAATAATAAATTTATTTTTAT			
5	TTAATGGTTCACAAACGGTTCACT	24	6	>spacer2			
				TTAAGCTTGTTGGTGCATCC			
				>spacer3			
				AAAGGTAGACAAACGGTTCACTAATGTTTGAGCTAACAAAT			
				>spacer4			
				GAAATTTTGATCTGGCTAATTGATTTGTTA			
				>spacer5			
				AATGGTTCACATATAAATTAGTATGTATATT			
				>spacer6			
				AACGGTTCACGATCGGTTCAATGATGGTTTTTG			
				>spacer7			
				ATTGGTTCAATTATTTGAAATTAGAAACGATTCATAATTAAGTTAAGTTATTGGGTT			
L. maceachernii (D5800)	**DR (consensus)**	**DR length**	**No. of spacers**	**Spacer sequences**	**Results of BLASTN search against the nr.nt**	**Searching similarities between upstream and**	**putative confirm CRISPR**

					**database**	**downstream regions and cas genes**	
1	CCACCCAGGAGATCCCTCACTACGTTCGGGACGACAA	37	1	>spacer1			
				CACCACCGTCATCCTGAACAAAAGCGAAGGACCTCCCAACTCTTGGCAGTAACC			
2	CGCTCAGGACGACAACACTCACTGTCATCCTGAGCAA	37	1	>spacer1			
				TGCGAAGGACCTCCCGACTCTTGGCAATAACCCAACCAAGGAGATCCCTCACTA			
3	GTGGAGTTACTGCCAAGAGTCGGGAGGTCC	30	1	>spacer1			
				TTCGCGTTGCTCAGGATGACAGGGGTATTGTCGTCCTGAGCGAAGCGAAGGATCTCCTTG			
4	ACATCATTCGGCTGTAGAGCTAACGCGGCATTTAAATCCGTTAAGGCAT	49	1	>spacer1			
				TATCGAATTGGTTCGATTGTCGATAAACCTCTCCCCGTCTTGCTAAAGCAAAT			
5	GGTATTGTCGTCCCGAACGAAGTGAGGGATCTCCTGAAGGAGGG	44	1	>spacer1			
				CACTGCCAAGAGTTGGGAGGTCCTTCGCTTTCGCTTAAC			
6	TTCATGGTTGATATACGGAGTATCTTCTTTTG	32	1	>spacer1			

				AATTATAAAACCTACCTTCCGCATTGAATGGCTT GAAATCTTGTTTCGCCTCTTC			
7	GGGTTACTGCCAAGA GTTGGGAGGTCCCT CGCTTTTGCTCG	41	1	>spacer1			
				GGATGACAGAGGTTGTTGTCGTCCCGAACGCA GTGAGGGATCCCCTGGATG			
8	TCATCCTGAGCAAAA GCGAAGGACCTCTC GACTC	34	1	>spacer1			
				CTGGCAGTAGCTTGCCACAGGAGATCCTTCGCT TCGCTCAGGACGACAATCAACACG			
9	AGGAGATCCTTCGCT TCGCTCAGGACGACA ACACAACT	38	1	>spacer1			
				GTCATCCCGAGCAAAGCGAGGGACCTCCCGAC TCTTGGCACTAACCCCACCA			
10	TTTAGAGAGCTTATG AATTTACC	23	1	>spacer1			
				CCCAATAACCTCTCAGGATGCCCCCAAAATAAC CGCTGTTCAACCACGTAAGACT			
11	TTTCTAAGCTGCCTG TGCGGCAGTGAAC	28	22	>spacer1		Cas3_family CRISPR-associated helicase tax=Gamma proteobacter ia	
				GGCTTTTAGCAAGACATCTAAAGACAAGCAT			
				>spacer2			
				GATCATTATCAAATTTTGCCCTAACTTTAGCA			

				>spacer3				
				TTATATGACCTGTTATTATCCCCTGCAAGCTT				
				>spacer4				
				TGCGTTAATCATAAAGGCTTCCTTAGTTTTTT				
				>spacer5				
				CTAAGGTTCGCGCAAAGTTTGATAATGATCCT				
				>spacer6				
				GAACGAGAAAAATCATGTGCACGAATCATGCC				
				>spacer7				
				CGAAAATATATGCTCATATTCTTTGTCTGAT				
				>spacer8				
				TAATCATAGCATTTGAATTAAAATGTATTT				
				>spacer9				
				GATACGCACTTAAAACCAAGAGGAGATGTAAA				
				>spacer10				
				TCTGCGGAACAATATAATCAGTCGAATCATCA				
				>spacer11				
				TAAAAGCTTAGGTTTAGCTCTTGATTTTGTTG				
				>spacer12				
				TTCGAGGAGCTTTGTTGTAATTTTCTCGTCAA				
				>spacer13				
				CATCATCAACTTTAATATCACCGTATTTTTTA				
				>spacer14				
				ATTAGGGGATTTATGCGCGGAAAATCCGCATT				
				>spacer15				
				TTCGAGGAGCTTTGTTGTAATTTTCTCGTCAA				
				>spacer16				
				GCTCATCAACAACAGCTTTAACACGCTCCATA				

				>spacer17			
				GTAACTCTACAGCAGGACCCTTCTGAGGCCAG G			
				>spacer18			
				GTTGTTTTTTAGAATCTTTTGATATTCTTTC			
				>spacer19			
				CCATAACTGCTCAAGAATATCCGAGCGATACA			
				>spacer20			
				TTAATGATGTTTCCTGGCAGACTAACCCAGTC			
				>spacer21			
				TCCTGTTAATGATGTCTCCTGGCAGACTAACC			
				>spacer22			
				TTATTACTGGTCGTTTTCGTTCTAATGCTACC			
L. jordanis (D5875)	**NO CRISPR SEQUENCES IDENTIFIED**						
L. rowbothamii (D5054)	**DR (consensus)**	**DR length**	**No. of spacers**	**Spacer sequences**	**Results of BLASTN search against the nr.nt database**	**Searching similarities between upstream and downstream regions and cas genes**	**putative confirm CRISPR**
1	GCTGGTTAAATCAGC TATATAACTCCAGCC	30	1	>spacer1			
				CATTTCTAATTCTTGGAGTTCTTTCTTTAATGTAT TGGCACGCTGCAGGCGTTCATT			

L. cincinnatiensis (D3325)	**NO CRISPR SEQUENCES IDENTIFIED**						
L. tusconensis (D1087)	**NO CRISPR SEQUENCES IDENTIFIED**						
L. brunensis (D1635)	**DR (consensus)**	**DR length**	**No. of spacers**	**Spacer sequences**	**Results of BLASTN search against the nr.nt database**	**Searching similarities between upstream and downstream regions and cas genes**	**putative confirm CRISPR**
1	GTTCACTGCCGTATAGGCAGCTTAGAAA	28	19	>spacer1		CRISPR-associated protein Cas1 tax=Proteobacteria	
				ATGAAGCAGTGACAATAGGTTCTGATGTACTT			
				>spacer2			
				TATATTACTTGACTACAAAACAATAAAGCGTA			
				>spacer3			
				TTTTCATAATACACCTGCCATAAGTTCTTCCA			
				>spacer4			
				AAGAACTCACGCAGGAAAAGCCAAAACCTAAA			
				>spacer5			
				AAGGTAAAGGTTTAGGTTTATTCAGAATTGCG			
				>spacer6			
				TCAGATTTCGCTCTAAGGTGAGTACTTGTAAC			

				>spacer7			
				AAGTGGCACTACAGCGCAGCGTCCGGGGATGT			
				>spacer8			
				AAGGTGCAAACGCTCATTATATAGACACTGGC			
				>spacer9			
				TTTCACGCAGGTCAGTTTGCAATTTTCTTAAA			
				>spacer10			
				TTCTATTTCTATCTTGTGAACACCCCAGAAGC			
				>spacer11			
				TGAGCAATCAACGGCTTTCTTTTATAGACAAG			
				>spacer12			
				TAGCTATTTCTCCTTCTGGATTAACGCTAGGC			
				>spacer13			
				TTGTAGCATCTGCCGCTGTTATCCCTGTTGCT			
				>spacer14			
				TCTGCCTCAATTTCCGCTTCTTCTCGTTTTAG			
				>spacer15			
				TTAAACGTTTCATCGTCAATGCTCTTATCACT			
				>spacer16			
				AGACAGCTAAGATACGTTCAGCACTTGTAAAA			
				>spacer17			
				ATGGTACTGGCAAGACAATTACATCGACAACT			
				>spacer18			
				GTTTAAGTTCAATAGAGCGCAAGAATATATTC			
				>spacer19			
				ATGATTGAAAATTATTGTGAGCATGACCCAAG			

2	GTTTTGTTTTCACTTCATCAACAT	24	1	>spacer2			
				TGCTGTAACTGGTATTAGTTTTTTGTTGTTCATCTGTTTTTTGGGGGGGAGGTGAAAGTA			
L. dumoffii (D5637)	**DR (consensus)**	**DR length**	**No. of spacers**	**Spacer sequences**	**Results of BLASTN search against the nr.nt database**	**Searching similarities between upstream and downstream regions and cas genes**	**putative confirm CRISPR**
1	TAAGACGTCTCACTCTGCTCAAATGAAATCTGACTCCTCGTCGACC	46	1	>spacer1			
				TCAATGAACAAGAGCATGGCCATGCCGGGCATGAATCATAGCGCAATGGGCCAGAA			
***L. pneumophila* Alcoy**	**DR (consensus)**	**DR length**	**No. of spacers**	**Spacer sequences**	**Results of BLASTN search against the nr.nt database**	**Searching similarities between upstream and downstream regions and cas genes**	**putative confirm CRISPR**
1	GTTAACTGCCGCACAGGCAGCTTAGAAG	28	56	>spacer1		CRISPR-associated protein Cas1 tax=Proteobacteria	
				TATTAAATTACTAAATACAAGTCCTTTTGATC			
				>spacer2			

				ACCTTATGTTATATTAAATAGTGTAAAGATTT			
				>spacer3			
				TATCTATTTAGATATAGTCTTCTTTCTATATA			
				>spacer4			
				TTAGCTTTGTGTACAAAGTGTGCTAGTGATTA			
				>spacer5			
				TATAAGATGAAAAGTTATTGACAAAATGTACT			
				>spacer6			
				TGTATTTGCTGTTAGTTGCCAGCCACCTGCTC			
				>spacer7			
				TTTTGTTCCTAGCCCAGATCTAGGAATTATTA			
				>spacer8			
				TTGGGAAATGTCTAATACAGTCACTTTTTATG			
				>spacer9			
				CAGTAGACGGCACTAAATGCTATGGATATGCT			
				>spacer10			
				AGTTTGCCGCTTAAAGATGCAAATGAAATGCT			
				>spacer11			
				GTAACTTTCCGTTTACACCAGTTACTTCATAT			
				>spacer12			
				TTTGTAATTGTCTCCACCATGCTTTTTTATCC			
				>spacer13			
				AAAGACACTGCACCATCCCAAGGACAACCTCT			
				>spacer14			
				AAACAACCAGCATATCGTTCTCATCAGCAACT			
				>spacer15			
				AAGCGCAAGCGATCAAGGTCGTTGGTCTCAAA			
				>spacer16			

				AACAGCGGGTGCGACATCAAATTTAATAATAA			
				>spacer17			
				AACTACAGAAATCCGATTGGCTACAACATCAG			
				>spacer18			
				ACTATCTGGTAGAAGGATCGCATTTAACTAAG			
				>spacer19			
				CTTAGCGTATCTACCATTATAATACCTTTTCC			
				>spacer20			
				TGTTTTTAAATCTTTATACTGTAAATTTTGCA			
				>spacer21			
				AAGTTGTGTTTGCTGTAATGCAAATAACTTCA			
				>spacer22			
				ATTAGCACCACTTGCCCCCGCAGTCGATGTAG			
				>spacer23			
				TACAGATAACACTGAATATGAGGCTTACGGAT			
				>spacer24			
				TGACGTTAACAATGAAATTAAAGTCAAAAGTG			
				>spacer25			
				GTGCGACATAGGAGTAAAGTTAAAATCGCAAA			
				>spacer26			
				ACTAAGTTCTATATATCCAAGTCCTTTGCATT			
				>spacer27			
				AAAGATTGCACGTAAATTTAAATTGTTTTCTA			
				>spacer28			
				AAAACACCAGGATTATGAATGTGTCTTTTTAA			
				>spacer29			
				ATAATCTCTGCGAATTGCTTGAGCTAAATCTT			
				>spacer30			

				ATAATTCTCAGTTTCAGTAAGTATACCATATC			
				>spacer31			
				TGAAACACTTTCAAAAGTGGCTTCGTATTCTT			
				>spacer32			
				TGCTTTGAGTCATATTGGAGAGCAAGCTGTTC			
				>spacer33			
				AAATGCCTGCGCTATTTGAGCATTGCGCGCAG			
				>spacer34			
				GTCCAGAAGATAGATTGCTCGTACTCAGCATC			
				>spacer35			
				AAAACGCCATCAATTCAACACCATCACTAATG			
				>spacer36			
				AATGTTCTTTTGAAAAAATTAATTAATAGTCA			
				>spacer37			
				TGTATACTGCATAATGGGGGTATTATTGCACA			
				>spacer38			
				TCAATCTGGGGTACTGACCGGCTCATATCATA			
				>spacer39			
				AGCGTGGGGTAAACAAATATTTGATAGCTTAA			
				>spacer40			
				TCTATATGCTCTCGCTTAATGTCTTTTAATGT			
				>spacer41			
				TTTGAACGATCAAAGGCTCAATCTTGTTTCCC			
				>spacer42			
				ATTCGTCCTTGGTAACGTAGAGTTAATCTATA			
				>spacer43			
				CCAATTAATCACCCTACTAGTACAAATTTTATAC T			
				>spacer44			

				TTCTTAGCCCCTAACACTTGCATTTAAAATAT			
				>spacer45			
				TAAAGTGATGATAGGTTCCCAACCATTGCGAG			
				>spacer46			
				TTTTGGGGTAATTAGTGTGTGTTTGCAAATGC			
				>spacer47			
				TAAACGCTGGATCTCTATCCTGTGTTTGATGT			
				>spacer48			
				ATTCGAGGACAATAAATCCCAGTTTAACCCTT			
				>spacer49			
				AAATACAATTGGTATGGTGTTTTGTAGCTAGA			
				>spacer50			
				AGCAGATATCTCATCCCATTCATTATCTGATT			
				>spacer51			
				CAGTACCCGCTACCAAACAAAGTGCCAAACCAT			
				>spacer52			
				ATTTGGAGAAATGGATATCCCCATTTGGTGAC			
				>spacer53			
				AAAACGAATCATTAATGGAATTGCGCATGAAA			
				>spacer54			
				AAACCTAAAATAAGTATATACTGAATTTGAAT			
				>spacer55			
				AGTGATTTAGATAATTCTCAGCTTCCTGTTTT			
				>spacer56			
				TGTACTTATTTCATTTCCTGTAATATGTAATA			
2	CAGGTTAATGATGCT GGTGGTGTGTTGGG	29	1	>spacer1			
				CCAGCAGTACCAGCAGCATTTGACGGCTTAT			

L. pneumophila Corby	**DR (consensus)**	**DR length**	**No. of spacers**	**Spacer sequences**	**Results of BLASTN search against the nr.nt database**	**Searching similarities between upstream and downstream regions and cas genes**	**putative confirm CRISPR**
	TAAGACGTCTCACTCTGCTCAAATGAAATCTGACTCCTCGTCGACC	46	1	>spacer1			
				TCAATGAACAAGAGCATGGCCATGCCGGGCATGAATCATAGCGCAATGGGCCAGAA			
2	CAGGTTAATGATGCTGGTGGTGTGTTGGG	29	1	>spacer1			
				CCAGCAGTACCAGCAGCATTTGACGGCTTAT			
***Legionella longbeachae*_NSW150**	**DR (consensus)**	**DR length**	**No. of spacers**	**Spacer sequences**	**Results of BLASTN search against the nr.nt database**	**Searching similarities between upstream and downstream regions and cas genes**	**putative confirm CRISPR**
1	TATTAATGCTAAAAAAAGACTG	23	1	>spacer1			
				AAATCCATAACGTATTCGGAAATAATGCCAGTAACCAATGTAAGGT			
2	GTATTTGAGCCTTTT	27	1	>spacer1			

	GTAAATAAAATT						
				GTGAAAAACAAAGCAACTTTGATATTTAAGGGT GAAGACAAAAAA			
***L. pneumophila* Lens**	**DR (consensus)**	**DR lengt h**	**No. of spacer s**	**Spacer sequences**	**Results of BLASTN search against the nr.nt database**	**Searching similarities between upstream and downstrea m regions and cas genes**	**putative confirm CRISPR**
1	GTTCACTGCCGCACA GGCAGCTTAGAAA	28	52	>spacer1			
				TTTTAAAAAACTTTAAGTTCTTTTCTGAAACA		Cas3_family CRISPR-associated helicase tax=Gamma proteobacter ia	
				>spacer2			
				CGATGAATCAACTATCAATAGATGGCGTTTAC			
				>spacer3			
				AATAAACAAAATTTGGAGTTAAATGTGAATGA			
				>spacer4			
				TTCAGCACATCATATGCGCAGTTAATCAACTT			
				>spacer5			
				ATACCGACACATTTTAATGGGATGGGGTGTCT			
				>spacer6			
				ACTTAATATTACCGAGCTAACATCAGCTAAAA			
				>spacer7			
				TATCGCAAGGCGTGTTCTTTGTATCATCCAGA			

				>spacer8			
				TGAAAAAACATTACACAATGATTCTACTATAC			
				>spacer9			
				ATTACACATATGACACTTAATCATTATCAATC			
				>spacer10			
				ATCCTAAGCACAAGCTCGCGCACTGTAGCCGT			
				>spacer11			
				TGTATCCACTGGAGACTCTCTTCTAATTCTTA			
				>spacer12			
				TGCTGAATCAAATTGACGCCTATAAGTTAAAT			
				>spacer13			
				TCTTTAACTTCTCTAATTATAGTACTGTTATC			
				>spacer14			
				AGCAAAAAAACGAATCGTCGTCAAGCGCAAGT			
				>spacer15			
				ATCTGCTTCATAAATTAAAAATGGAGAACTAG			
				>spacer16			
				CACCATTAACAGTTCCTGCGAGTACCGGCACCA			
				>spacer17			
				ATAACAGCGCAAGAGATTAAGGGATAGATAAA			
				>spacer18			
				TTCTCAACACAGTCTTTTTTGCCATCTCTTCA			
				>spacer19			
				TATACCGACTGCGCTTCCGGTGGTTCTTTGTG			
				>spacer20			
				AAAAGCCAACCGAGACAGCCAAAAAGAGCTTA			
				>spacer21			
				ATTGTGCCCAAGCCAAGGTTGGTTCTTGCGTC			

				>spacer22			
				ATAGTGAAAGTAACATAACCACGAGCAAAAAG			
				>spacer23			
				TGATTGGTTTCTTTTTCTTGACTGTTCTTTGA			
				>spacer24			
				TCTGATAGCGCAACGGCACTGGCCACCCAGCA			
				>spacer25			
				AGACAAGACTGGAACAATATCTCCAGCCTGTA			
				>spacer26			
				ATGAGCCAGTCGTATTTCATCTTGTTTTAGTA			
				>spacer27			
				AGGCAGTCGGAGAGCAGGAAAACACGGAAATC			
				>spacer28			
				GCTTGCTGTTGCTTTGCTTGTGCGTATTGCAC			
				>spacer29			
				ATTTGAAAAATCTATCCCTAAATCTTATGTAC			
				>spacer30			
				TCTTTGAAATGACAAAAAGGTATTATGAGTAC			
				>spacer31			
				ACAAGAAAGCGTGGACTTAATAGAGTTACTAG			
				>spacer32			
				TTCCCACGATGCCAGCGAGCAAGAAACAGAGG			
				>spacer33			
				GTCCATCCTTGCGTTTCCTTCCTTGTCGTATG			
				>spacer34			
				ATTTCGCCTTCAACGTCATGCGATAAATTTTT			
				>spacer35			
				GCTTGAACTTCTGCGACTGCTCTATCGGATTG			

				>spacer36			
				TTTGGCTTCCAATGGCGTTGTATTAACAAAAA			
				>spacer37			
				AGTCGCAACATTCCCGATTGACAAGTAAAGAC			
				>spacer38			
				ATATTCTGTTTGACCAAAGCAATCATTTGAAT			
				>spacer39			
				TACAAGTCAAAATTTTCTATTTCTTTGACAGC			
				>spacer40			
				ATAGATGTTACGTTAGACCAAAGTCATTTGGG			
				>spacer41			
				TTGCAGCGAAGGAGAAGTGCCATAATGTACAA			
				>spacer42			
				TTCACAACGCCTGGGTTAGTGCTAGGAGCAGA			
				>spacer43			
				TTCGCGTCTTTAATCTGTATATTCGGGTGTTA			
				>spacer44			
				AATAAGGTCGAACTGCTACACTAAGTTTACACC			
				>spacer45			
				ACTACATTCTTTAATACTTGTGCCATTTCTTA			
				>spacer46			
				AAATAACCGGATTGAACCTTCCCGAATAACAC			
				>spacer47			
				TAATGCAATTATCATGAATAATACTATGCACT			
				>spacer48			
				ATTACGACAACATAGAAGGCATAACCACAAGG			
				>spacer49			
				TGTGCAGCTATTGGAGGCTTAGGGGCTAAGTT			

				>spacer50			
				GCTGCTTATATTGGCGGCGACCTTTAAGATTT			
				>spacer51			
				TTACAAAGCCTGCTGTATGCGTCTTTTAATAA			
				>spacer52			
				TTGATGTTCGTGCGCCCTATTCGCAGTTATTA			
2	GTTCACTGCCGCACA GGCAGCTTAGAAA	28	12	>spacer54		Cas3_family CRISPR-associated helicase tax=Gamma proteobacter ia	
				AGTACCACGGCACAAACACCGCCTGAAAGTTG			
				>spacer55			
				TACTACGTCCTTAGCTCTTAAGTCTTTTGTGC			
				>spacer56			
				TCTTGCGGCGCGTCTTTGTCTTCCATTAATTC			
				>spacer57			
				CAACAGACACGACAGGGTAGGGTTGCCCACAA A			
				>spacer58			
				GCCATCATGATTACTAAAGTCGATTGTGACCC			
				>spacer59			
				CAAAGTGTACGGAGATAGACCGCAAGACGACA			
				>spacer60			
				AATATAAAGCATCGGGAAAATGTAGACAAAAT			
				>spacer61			
				AAATGCACGGCGCAAAAGTCAGAAAACAGCCT			
				>spacer62			

				AGACAGAAATCCGGCTGGGCTAGAAATGATGA			
				>spacer63			
				TCTTGCCTGCTCTTAACTACAACTCCAGACCA			
				>spacer64			
				AAACAACCACACCTCCCTTTTTAGTTAATCTA			
				>spacer65			
				GATTCGATACATTTTTTAACTCGTCGCCCCAGT			
3	AAATTTACCGCGATA ACAGTAATTT	25	2	>spacer1			
				TTAGGAATTTATAATAAATAAATATGGGAAAATC AGGAAATCAATT			
				>spacer2			
				ATGGTGAATTAATGCAAGGAAATGCAATAATAAA TTTATTTTTATC			
**Mutant_Legionella_p _Hextuple_3a.fasta**	**NO CRISPR SEQUENCES IDENTIFIED**						
Legionella pneumophila **Paris**	**DR (consensus)**	**DR length**	**No. of spacers**	**Spacer sequences**	**Results of BLASTN search against the nr.nt database**	**Searching similarities between upstream and downstream regions and cas genes**	**putative confirm CRISPR**
1	CCAATAATCCCTCAT CTAAAAATCCAACCA CTGAAAC	37	33	>spacer1		:putative CRISPR-associated large protein (Provisional) tax=gamma	

				TAGATATAAAAAGATTAAATCTTCTAGCGCACAT			
				>spacer2			
				TCACTACTCCTGAAGGTTATAATTTTTGCTATAA			
				>spacer3			
				TTCGAATACAATCCTAGTGTCTCTGTGTGAATTAAG			
				>spacer4			
				CAGGCACTGGTTCACTAGACACTGTAACATCTAT			
				>spacer5			
				CAATAACAAGCGAGCCTTTTGTACTAGAAGGTTTA			
				>spacer6			
				CTACCAGTTAATCGTAACTCAATCTCTTTTTCAA			
				>spacer7			
				ATAGAATACATAAGTGCAAATTATTAAATGTTAC			
				>spacer8			
				TGAATGTAGAAACCAGATGCCACGAATTATTAGA			
				>spacer9			
				TTTGTATAAACGTTCTGATATGACTTAGGTAATCT			
				>spacer10			
				CTAACCTGATTGCTCAACAAATAATGCTATTGGC			
				>spacer11			
				TCACTTTAGGCCAACGCCGATCCTCCGCTTCGAA			
				>spacer12			
				TGACGCAAAGGATTTATTAAAAACGCCTTGTAAT			
				>spacer13			
				ATTTTACCTTTTAACACATATTGATAGGCGT			
				>spacer14			

				TCCATAACTGAAACGTCCTTATGCCTCAACATAATG				
				>spacer15				
				GAATTTGTCGGCCGCATAGACCGCTTTTATCAAA				
				>spacer16				
				CTATTGCAAGCTAGTTTGATCGTGTTATTATAAGAA				
				>spacer17				
				TGACAAACGTTTGTTTTTTAGACACAACACTAAAAG				
				>spacer18				
				CCTGAAAACCCGCCACAACCCGCGCCAGACTTGAA				
				>spacer19				
				ACCAAGTCGAAACAACATACCGAGACCGTGTTGA				
				>spacer20				
				TACATTGTTACGTTCATTTCACTCAGTTTTTCATA				
				>spacer21				
				AGCAATAACCCAAAGTTTCGCGCGCGTGCGCGGG				
				>spacer22				
				TTATAACATCGGGATGGCGGTTTATTGGTTAAGTAAC				
				>spacer23				
				TTCTTTTTTCAGATTTCATTTCCTTTTCCTTGTG				
				>spacer24				
				AATCTTAATTTATGCGCCTTACCTTCTGCTTCATC				
				>spacer25				
				TATACTTAGACAATATCATCATTTCCTATGTTTCGA				
				>spacer26				

				GCTATTCCAGATAGGTAGTTGTTTAGAGCATTTT GT			
				>spacer27			
				TTCGATACTCCTTAGCGGTACTTGCTAAAGTAGT T			
				>spacer28			
				AAATAATTCTTGTGACTCACGTGCCGCCATTTGA A			
				>spacer29			
				TAGGAATTGATTGGGGTAACGCCATCGCCATAG AAG			
				>spacer30			
				TTACTTCATGACCCGGACATTTTACTAAAAAAT			
				>spacer31			
				TCCTATACCTTGTCACAATTTCCTCATATGGCTC			
				>spacer32			
				CATAATTGGGAATTGGTGTGAAATGCTCACCGT CCG			
				>spacer33			
				TTAATTGCGCCAGAAACAGCACCATTTATGGTTA CAGC			
2	CTATTCTTAATGCTCA ACAGAATAAAAAACA ACAGA	36	1	>spacer1			
				CAAATAGTCCATCTAAAGAGATAAAAAAATTTGA TATGTCAGAAGCTCAATTATGGG			
L. pneumophila **Philadelphia**	**NO CRISPR SEQUENCES IDENTIFIED**						
**pneumophila_ATCC_**	**NO CRISPR**						

**43290**	**SEQUENCES IDENTIFIED**						
**pneumophila_str_HL 06041035**	**NO CRISPR SEQUENCES IDENTIFIED**						
***L. pneumophila* Lorraine**	**DR (consensus)**	**DR lengt h**	**No. of spacer s**	**Spacer sequences**	**Results of BLASTN search against the nr.nt database**	**Searching similarities between upstream and downstrea m regions and cas genes**	**putative or confirm d CRISP**
	CAAAAATATGGCTAT AATTATATATAATTTG CTTGGGA	38	1	>spacer1			
				TATTGATAGCACGTGCACCGGCCTGAGTAAATC			
***L. pneumophila* LPE509**	**NO CRISPR SEQUENCES IDENTIFIED**						
***L. pneumophila* Thunder Bay**	**NO CRISPR SEQUENCES IDENTIFIED**						
